# Genome-Wide Identification of Mitogen-Activated Protein Kinase Gene Family across Fungal Lineage Shows Presence of Novel and Diverse Activation Loop Motifs

**DOI:** 10.1371/journal.pone.0149861

**Published:** 2016-02-26

**Authors:** Tapan Kumar Mohanta, Nibedita Mohanta, Pratap Parida, Sujogya Kumar Panda, Lakshmi Narayanan Ponpandian, Hanhong Bae

**Affiliations:** 1 Free Major of Natural Science, College of Basic Studies, Yeungnam University, Gyeongsan, Gyeongsangbuk-do, 712749, Republic of Korea; 2 Department Of Biotechnology, North Orissa University, Takatpur, Baripada, 757003, India; 3 Regional Medical Research Centre, NE Region, Indian Council of Medical Research, Dibrugarh, 786001, Assam, India; 4 Department of Zoology; North Orissa University; Baripada, Odisha, 757003, India; 5 School of Biotechnology, Yeungnam University, Gyeongsan, Gyeongsangbuk-do, 712749, Republic of Korea; Yonsei University, REPUBLIC OF KOREA

## Abstract

The mitogen-activated protein kinase (MAPK) is characterized by the presence of the T-E-Y, T-D-Y, and T-G-Y motifs in its activation loop region and plays a significant role in regulating diverse cellular responses in eukaryotic organisms. Availability of large-scale genome data in the fungal kingdom encouraged us to identify and analyse the fungal MAPK gene family consisting of 173 fungal species. The analysis of the MAPK gene family resulted in the discovery of several novel activation loop motifs (T-T-Y, T-I-Y, T-N-Y, T-H-Y, T-S-Y, K-G-Y, T-Q-Y, S-E-Y and S-D-Y) in fungal MAPKs. The phylogenetic analysis suggests that fungal MAPKs are non-polymorphic, had evolved from their common ancestors around 1500 million years ago, and are distantly related to plant MAPKs. We are the first to report the presence of nine novel activation loop motifs in fungal MAPKs. The specificity of the activation loop motif plays a significant role in controlling different growth and stress related pathways in fungi. Hence, the presences of these nine novel activation loop motifs in fungi are of special interest.

## Introduction

Among the several signal transduction pathways present in an eukaryotic system, the mitogen-activated protein kinase (MAPK) pathway is one of the most important signaling pathways [1–4]. It is considered as one of the evolutionary conserved signal transduction pathways that transduces an extracellular signal to the nucleus and maintains proper adjustment of cellular responses [2]. The signaling pathways consist of myriads of cascades that are induced in response to environmental cues. A recent development in the study of MAPK shows that, this pathway is involved in diverse cellular responses [5–7]. The MAPK cascade consists of a three-kinase signaling module; MAP kinase kinase kinase (MAP3Ks), MAP kinase kinase (MAP2Ks), and MAP kinases (MAPKs) [1,2,8] which are connected to each other by a process of sequential phosphorylation events [1,5]. Upon extra-cellular responses, the signalling molecules activate upstream MAP kinase kinase kinase kinase (MAP4Ks) or MAP kinase kinase kinase (MAP3Ks) making them as adaptor-signaling molecules [8] which phosphorylates downstream MAP2Ks at S/T-X_3-5_-S/T motif of the activation loop region [9]. Subsequently, MAP2Ks phosphorylates MAPKs at conserved T-x-Y (T-E-Y/T-D-Y) motifs in the activation loop region [1,10]. Upon activation, the MAPKs are able to phosphorylate a large number of downstream targets, including other kinases and transcription factors that regulate growth, development and stress responses. The MAPK pathway module present in fungi is activated by a member of the p21-activated protein kinase (PAK) that activates Ste20. Protein p21 is a monomeric Ras-related GTPase Cdc42 that activates Ste20 [11]. Upon activation by Cdc42, Ste20 modulates phosphorylation of Ste11 [11]. The Ste20 acts as an upstream MAPKKK kinase in MAPK module [12]. Later Ste20 phosphorylates downstream protein Ste7 (MAPKK) and Fus3 (MAPK). A scaffold protein Ste5, binds all three kinase module cascade together [11,12].

The MAPKs contain characteristic T-E-Y/T-D-Y/T-P-Y and the T-G-Y motif in the activation loop region [4,13–16]. Reports suggest that, activation loop motif T-E-Y and T-D-Y are common to plants and animals, whereas presence of T-E-Y, and T-G-Y motifs are unique to animals and fungi only [13]. The MAPK motif specificity in fungi plays distinct roles in maintaining different pathways. The Fus3 (T-E-Y) mediates cellular response to peptide pheromone, Kss1 (T-E-Y) helps in adjustment to nutrient limiting conditions, and Hog1 (T-G-Y) controls hyperosmotic condition in fungi [11]. The phosphorylation at both threonine and tyrosine residues in the T-x-Y motif of MAPK is important for locking the kinase domain in a catalytic competent conformation. The phosphorylation of tyrosine is usually followed by phosphorylation of threonine, although phosphorylation of any one of the two residues can occur in the absence of the other [17]. This sequential activation of the MAPK cascade controls different aspects of cellular activities and regulates proper growth and development of the organisms [18,19]. The fungi are very important microorganisms with a diverse genomic organization [20]. The MAPK gene family plays significant roles in fungal growth, development as well as different signaling and stress responses [20]. The MAPK research in fungal biology is in its infantile stage and required in-depth investigations. Therefore, to know more detail about fungal MAPKs, we conducted genome-wide identification of the MAPK gene family of 173 fungal species, and analysed their diversity and evolutionary aspects.

## Materials and Methods

### Identification of fungal MAPKs

To identify the MAPK sequences of fungal species, the MAPK protein sequences of *Saccharomyces cerevisiae* [P16892 (Fus3), P14681 (Kss1), P32485 (Hog1), P41808 (Smk1), and Q00772 (Slt2)], *Oryza sativa* [LOC_Os03g17700 (OsMPK3), LOC_Os10g38950 (OsMPK4-1), LOC_Os05g05160 (OsMPK4-2), LOC_Os06g06090 (OsMPK6), LOC_Os06g48590 (OsMPK7), LOC_Os02g05480 (OsMPK14), LOC_Os11g17080 (OsMPK16-1), LOC_Os08g06060 (OsMPK16-2), LOC_Os06g49430 (OsMPK17-1), LOC_Os02g04230 (OsMPK17-2), LOC_Os01g43910 (OsMPK20-1), LOC_Os05g50560 (OsMPK20-2), LOC_Os06g26340 (OsMPK20-3), LOC_Os01g47530 (OsMPK20-4), LOC_Os05g49140 (OsMPK20-5), LOC_Os05g50120 (OsMPK21-1), and LOC_Os01g45620 (OsMPK21-2)] and *Arabidopsis thaliana* [At1g10210 (AtMPK1), At1g59580 (AtMPK2), At3g45640 (AtMPK3), At4g01370 (AtMPK4), At4g11330 (AtMPK5), At2g43790 (AtMPK6), At2g18170 (AtMPK7), At1g18150 (AtMPK8), At3g18040 (AtMPK9), At3g59790 (AtMPK10), At1g01560 (AtMPK11), At2g46070 (AtMPK12), At1g07880 (AtMPK13), At4g36450 (AtMPK14), At1g73670 (AtMPK15), At5g19010 (AtMPK16), At2g01450 (AtMPK17), At1g53510 (AtMPK18), At3g14720 (AtMPK19), and At2g42880 (AtMPK20)] were used as the search query. The MAPK sequences of *Saccharomyces cerevisiae* were downloaded from Uniprot (http://www.uniprot.org/) database. The MAPK sequences of *O*. *sativa* and *A*. *thaliana* were downloaded from “Rice Genome Annotation Project (http://rice.plantbiology.msu.edu/)” and “The Arabidopsis Information Resource (TAIR) (http://www.arabidopsis.org/),” respectively [21,22]. The MAPK sequences from all the studied fungal species were downloaded from the Mycocosm database [23,24]. The MAPK sequences of plants were included in this study to broaden the diversity of search of fungal MAPK. Fungi has closer evolutionary role with plants which helped the photosynthetic plant lineage to acquire the land habitat. Therefore, their inclusion in this study was important. In total, 173 fungal species were taken during this study, and the names of all these species are listed in Table 1. The BLASTP program was used to identify the MAPK sequences of fungal species. Different statistical parameters used during BLASTP searches were: E- value: 1.0E-5, and scoring matrix: BLOSUM62. All the identified sequences were subjected to scanprosite software in FASTA format to check for the presence of serine/threonine protein kinase domain and activation loop motifs within it using the default parameters [25]. Scanprosite analysis of sequences which gave hit to prosite accession of serine/threonine protein kinase or mitogen activated protein kinase such as PS01351, PS00107, PS00108, PS50011 were considered as positive prediction for MAP kinase. The details of all the fungal MAPKs and their JGI accessions are provided in S1 appendix. The resulting protein sequences were again subjected to a BLASTP search against the TAIR and rice genome annotation database using default BLASTP parameters. The sequences that resulted in MAPK hits in the TAIR and rice genome annotation databases were considered as the fungal MAPK sequences.

**Table 1:** MAPK gene family of fungi. The MAPK gene family of 173 fungal species (including three Oomycota species) shows the presence of several novel activation loop motifs (T-T-Y, T-I-Y, T-N-Y, T-H-Y, T-S-Y, K-G-Y, T-Q-Y, S-E-Y and S-D-Y) in fungi.

Sl. No	Name of Fungal Species	Division	No. of Identified MAPKs	Activation Loop Motifs
1	*Acidomyces richmondensis*	Ascomycota	6	TEY, TGY
2	*Acremonium alcalophilum*	Ascomycota	5	TEY, TGY
3	*Amorphotheca resinae*	Ascomycota	3	TEY, TGY
4	*Anthostoma avocetta*	Ascomycota	6	TEY, TGY
5	*Apiospora montagnei*	Ascomycota	6	TEY, TGY
6	*Aplosporella prunicola*	Ascomycota	6	TEY, TGY
7	*Ascobolus immerses*	Ascomycota	6	TEY, TGY
8	*Ascoidea rubescens*	Ascomycota	4	TEY, TNY
9	*Aspergillus acidus*	Ascomycota	8	TEY, TGY
10	*Aspergillus niger*	Ascomycota	7	TEY, TGY
11	*Aulographum hederae*	Ascomycota	6	TEY, TGY
12	*Aureobasidium pullulans*	Ascomycota	4	TEY, TGY
13	*Babjeviella inositovora*	Ascomycota	6	TEY, TGY, TNY
14	*Baudoinia compniacensis*	Ascomycota	4	TEY, TGY
15	*Candida caseinolytica*	Ascomycota	3	TEY, TGY
16	*Cenococcum geophilum*	Ascomycota	5	TEY, TGY
17	*Cercospora zeae-maydis*	Ascomycota	4	TEY, TGY
18	*Chalara longipes*	Ascomycota	6	TEY, TGY
19	*Choiromyces venosus*	Ascomycota	8	TEY, TGY
20	*Cochliobolus sativus*	Ascomycota	6	TEY, TGY
21	*Cryphonectria parasitica*	Ascomycota	4	TEY, TGY
22	*Cucurbitaria berberidis*	Ascomycota	6	TEY, TGY
23	*Cyberlindnera jadinii*	Ascomycota	6	TEY, TGY, TNY
24	*Daldinia eschscholzii*	Ascomycota	4	TEY, TGY
25	*Dekkera bruxellensis*	Ascomycota	8	TEY, TGY, THY
26	*Didymella exigua*	Ascomycota	4	TEY, TGY
27	*Dissoconium aciculare*	Ascomycota	5	TEY, TGY
28	*Dothidotthia symphoricarpi*	Ascomycota	6	TEY, TGY
29	*Eurotium rubrum*	Ascomycota	7	TEY, TGY
30	*Glomerella acutata*	Ascomycota	7	TEY
31	*Glomerella cingulata*	Ascomycota	6	TEY, TGY
32	*Gymnascella aurantiaca*	Ascomycota	6	TEY, TGY
33	*Gymnascella citrina*	Ascomycota	8	TEY, TGY
34	*Hanseniaspora valbyensis*	Ascomycota	4	TEY, TGY, TSY
35	*Hansenula polymorpha*	Ascomycota	6	TEY, TNY
36	*Hyphopichia burtonii*	Ascomycota	5	TEY, TGY, TNY
37	*Hypoxylon sp*.	Ascomycota	6	TEY, TGY
38	*Lipomyces starkeyi*	Ascomycota	4	TEY, TGY
39	*Lophiostoma macrostomum*	Ascomycota	5	TEY, TGY
40	*Melanconium sp*.	Ascomycota	6	TEY, TGY
41	*Melanomma pulvis-pyrius*	Ascomycota	4	TEY
42	*Metschnikowia bicuspidata*	Ascomycota	6	TEY, TGY, TNY
43	*Monascus purpureus*	Ascomycota	5	TEY, TGY
44	*Monascus ruber*	Ascomycota	8	TEY, TGY
45	*Myceliophthora thermophila*	Ascomycota	3	TEY, TGY
46	*Mycosphaerella graminicola*	Ascomycota	3	TEY, TGY
47	*Myriangium duriaei*	Ascomycota	6	TEY, TGY
48	*Nadsonia fulvescens*	Ascomycota	5	TEY, TGY, THY
49	*Neurospora discreta*	Ascomycota	3	TEY
50	*Neurospora tetrasperma*	Ascomycota	5	TEY, TGY
51	*Oidiodendron maius*	Ascomycota	7	TEY, TGY
52	*Pachysolen tannophilus*	Ascomycota	8	TEY, TGY, TNY
53	*Patellaria atrata*	Ascomycota	4	TEY, TGY
54	*Penicillium brevicompactum*	Ascomycota	8	TEY, TGY
55	*Penicillium canescens*	Ascomycota	8	TEY, TGY
56	*Penicillium janthinellum*	Ascomycota	6	TEY, TGY
57	*Penicillium raistrickii*	Ascomycota	5	TEY, TGY
58	*Pichia stipitis*	Ascomycota	5	TEY, TGY, TNY
59	*Piedraia hortae*	Ascomycota	7	TEY, TGY
60	*Pleomassaria siparia*	Ascomycota	5	TEY, TGY
61	*Polychaeton citri*	Ascomycota	6	TEY, TGY
62	*Saccharata proteae*	Ascomycota	2	TEY, TGY
63	*Saccharomyces cerevisiae*	Ascomycota	6	TEY, TGY, TNY, KGY
64	*Saitoella complicata*	Ascomycota	4	TEY, TGY
65	*Septoria musiva*	Ascomycota	3	TEY, TGY
66	*Sodiomyces alkalinus*	Ascomycota	7	TEY, TGY
67	*Spathaspora passalidarum*	Ascomycota	6	TEY, TGY
68	*Sporormia fimetaria*	Ascomycota	9	TEY, TGY
69	*Talaromyces aculeatus*	Ascomycota	5	TEY, TGY
70	*Terfezia boudieri*	Ascomycota	5	TEY, TGY
71	*Thermoascus aurantiacus*	Ascomycota	4	TEY, TGY
72	*Thielavia appendiculata*	Ascomycota	10	TEY, TGY
73	*Thielavia arenaria*	Ascomycota	6	TEY, TGY
74	*Thielavia hyrcaniae*	Ascomycota	7	TEY, TGY
75	*Trichoderma citrinoviride*	Ascomycota	3	TEY, TGY
76	*Trypethelium eluteriae*	Ascomycota	7	TEY, TGY
77	*Wickerhamomyces anomalus*	Ascomycota	6	TEY, TGY, TNY
78	*Wilcoxina mikolae*	Ascomycota	6	TEY, TGY
79	*Xanthoria parietina*	Ascomycota	7	TEY, TGY, TNY, KGY
80	*Xylona heveae*	Ascomycota	6	TEY, TGY
81	*Zasmidium cellare*	Ascomycota	3	TEY, TGY
82	*Zopfia rhizophila*	Ascomycota	5	TEY, TGY
83	*Agaricus bisporus*	Basidiomycota	8	TEY
84	*Amanita muscaria*	Basidiomycota	15	TEY, TGY
85	*Antrodia sinuosa*	Basidiomycota	14	TEY
86	*Atractiellales sp*.	Basidiomycota	7	TEY, TGY
87	*Auricularia subglabra*	Basidiomycota	8	TEY, TGY
88	*Bjerkandera adusta*	Basidiomycota	9	TEY, TGY
89	*Boletus edulis*	Basidiomycota	13	TEY, TGY
90	*Botryobasidium botryosum*	Basidiomycota	7	TEY, TGY
91	*Calocera cornea*	Basidiomycota	6	TEY, TGY
92	*Calocera viscose*	Basidiomycota	9	TEY, TGY
93	*Coniophora puteana*	Basidiomycota	6	TEY, TGY
94	*Coprinopsis cinerea*	Basidiomycota	12	TEY, TGY
95	*Cortinarius glaucopus*	Basidiomycota	10	TEY
96	*Cronartium quercuum*	Basidiomycota	7	TEY, TGY
97	*Cryptococcus vishniacii*	Basidiomycota	7	TEY, TGY
98	*Cylindrobasidium torrendii*	Basidiomycota	10	TEY, TGY
99	*Dacryopinax sp*.	Basidiomycota	7	TEY, TGY
100	*Daedalea quercina*	Basidiomycota	15	TEY
101	*Dendrothele bispora*	Basidiomycota	10	TEY
102	*Dichomitus squalens*	Basidiomycota	9	TEY
103	*Dioszegia cryoxerica*	Basidiomycota	7	TEY, TGY
104	*Exidia glandulosa*	Basidiomycota	7	TEY
105	*Exobasidium vaccinii*	Basidiomycota	10	TEY, TGY
106	*Fibulorhizoctonia sp*.	Basidiomycota	8	TEY, TGY
107	*Fomitiporia mediterranea*	Basidiomycota	8	TEY, TGY
108	*Fomitopsis pinicola*	Basidiomycota	7	TEY, TGY
109	*Galerina marginata*	Basidiomycota	5	TEY
110	*Ganoderma sp*.	Basidiomycota	5	TEY
111	*Gloeophyllum trabeum*	Basidiomycota	11	TEY
112	*Gyrodon lividus*	Basidiomycota	10	TEY
113	*Hebeloma cylindrosporum*	Basidiomycota	13	TEY, TGY
114	*Heterobasidion annosum*	Basidiomycota	6	TEY
115	*Hydnomerulius pinastri*	Basidiomycota	7	TEY
116	*Hypholoma sublateritium*	Basidiomycota	11	TEY, TGY
117	*Jaapia argillacea*	Basidiomycota	11	TEY, TGY
118	*Laccaria amethystina*	Basidiomycota	20	TEY, TGY
119	*Laccaria bicolour*	Basidiomycota	7	TEY, TGY
120	*Laetiporus sulphureus*	Basidiomycota	12	TEY, TGY
121	*Lentinus tigrinus*	Basidiomycota	6	TEY
122	*Leucogyrophana mollusca*	Basidiomycota	9	TEY
123	*Macrolepiota fuliginosa*	Basidiomycota	14	TEY, TGY
124	*Melampsora laricis-populina*	Basidiomycota	6	TEY, TGY
125	*Meliniomyces bicolour*	Basidiomycota	8	TEY, TGY, SDY
126	*Mixia osmundae*	Basidiomycota	6	TEY, TGY
127	*Neolentinus lepideus*	Basidiomycota	13	TEY
128	*Paxillus rubicundulus*	Basidiomycota	18	TEY, TGY
129	*Phlebia brevispora*	Basidiomycota	8	TEY, TGY
130	*Phlebiopsis gigantean*	Basidiomycota	10	TEY, TGY
131	*Piloderma croceum*	Basidiomycota	11	TEY, TGY
132	*Pisolithus microcarpus*	Basidiomycota	8	TEY, TGY
133	*Pleurotus ostreatus*	Basidiomycota	10	TEY, TGY
134	*Polyporus arcularius*	Basidiomycota	7	TEY
135	*Punctularia strigosozonata*	Basidiomycota	10	TEY, TGY
136	*Pycnoporus sanguineus*	Basidiomycota	9	TEY, TGY
137	*Ramaria rubella*	Basidiomycota	11	TEY
138	*Rhodotorula graminis*	Basidiomycota	6	TEY, TGY
139	*Rickenella mellea*	Basidiomycota	6	TEY
140	*Schizophyllum commune Loenen*	Basidiomycota	9	TEY
141	*Schizophyllum commune Tattone*	Basidiomycota	9	TEY
142	*Schizopora paradoxa*	Basidiomycota	10	TEY, TGY
143	*Scleroderma citrinum*	Basidiomycota	11	TEY, TGY
144	*Sebacina vermifera*	Basidiomycota	10	TEY, TGY
145	*Serpula lacrymans*	Basidiomycota	7	TEY, TGY
146	*Sistotrema brinkmannii*	Basidiomycota	9	TEY, TGY
147	*Sistotremastrum niveocremeum*	Basidiomycota	8	TEY, TGY
148	*Sporobolomyces roseus*	Basidiomycota	5	TEY, TGY
149	*Stereum hirsutum*	Basidiomycota	7	TEY, TGY
150	*Suillus brevipes*	Basidiomycota	9	TEY
151	*Trametes versicolor*	Basidiomycota	4	TEY
152	*Trichaptum abietinum*	Basidiomycota	10	TEY, TGY
153	*Tulasnella calospora*	Basidiomycota	10	TEY, TGY
154	*Wallemia sebi*	Basidiomycota	6	TEY, TGY
155	*Wolfiporia cocos*	Basidiomycota	8	TEY, TGY
156	*Catenaria anguillulae*	Blastocladiomycota	4	TEY, TGY
157	*Conidiobolus coronatus*	Entomophthoromycota	6	TEY, TGY
158	*Rhizophagus irregularis*	Glomeromycota	6	TEY, TGY
159	*Coemansia reversa*	Kickellomycotina	4	TEY, TGY
160	*Gonapodya prolifera*	Monoblepharidomycetes	8	TEY, TGY
161	*Backusella circina*	Mucoromycotina	5	TEY, TGY
162	*Lichtheimia hyalospora*	Mucoromycotina	9	TEY, TGY, THY
163	*Mortierella elongate*	Mucoromycotina	5	TEY, TGY
164	*Mucor circinelloides*	Mucoromycotina	8	TEY, TGY, THY
165	*Phycomyces blakesleeanus*	Mucoromycotina	6	TEY, TGY, THY
166	*Rhizopus microsporus*	Mucoromycotina	8	TEY, TGY, THY
167	*Umbelopsis ramanniana*	Mucoromycotina	5	TEY, TGY, TQY
168	*Piromyces sp*.	Neocallimastigomycota	2	TEY
169	*Phytophthora capsici*	Oomycota	12	TEY, TGY
170	*Phytophthora cinnamomi*	Oomycota	6	TEY
171	*Phytophthora sojae*	Oomycota	8	TEY
172	*Aplanochytrium kerguelense*	Stramenopiles	4	TEY, TTY
173	*Aurantiochytrium limacinum*	Stramenopiles	7	TEY, TIY, SEY

### Nomenclature of fungal MAPKs

Nomenclature of a gene is very important to know its exact identity. But, it was a very difficult task to name all the identified fungal MAPKs. Therefore we named the fungal MAPKs which contained only novel activation loop motif. Name was provided by taking first letter of genus name in upper case and first letter of specie name in lower case followed by MPK. Functional characterizations of all the novel activation loop motifs are yet to be done; therefore the naming of fungal MAPKs which contains novel activation loop motifs were not done according to gene *HOG*, *SLT2*, *KSS1*, *FUS3* and *SMK1* as found in *Saccharomyces cerevisiae*.

### Molecular modeling of fungal MAPKs

During the identification of fungal MAPKs, we identified several MAPKs that contains novel activation loop motif. Therefore, representative molecular structures for all the MAPKs were modelled which contained the novel activation loop motif. The fungal MAPK sequences that contained novel activation loop motif was used to build the molecular structure with the help of Swiss-model workspace [26]. After building the models, the models were analysed for the presence of novel motifs using Pymol software.

### Multiple sequence alignment

Multiple sequence alignments of all the identified MAPKs of fungi were conducted using Multalin software (http://multalin.toulouse.inra.fr/multalin/) [S1 Fig]. Owing to large data files of multiple sequence alignments, it was difficult to incorporate them all in the manuscript for a conceptual figure. Therefore, we again conducted another multiple sequence alignment of few selected fungal MAPK sequences those that contained the novel activation loop motif. Different statistical parameters used to run the Multalin program were as follows. Protein weight matrix: Blosum62-12-12; gap penalty at the opening: default; gap penalty at extension: default; gap penalty at the extremities: none; one iteration only: no; high consensus level: 90%; and low consensus level: 50%.

### Phylogenetic analysis

Different phylogenetic trees were constructed to infer the phylogenetic relationship of fungal MAPKs. In the first case, all the MAPK sequences of the fungal species were taken to construct a phylogenetic tree. In the second case, a phylogenetic tree was constructed by taking fungal MAPK sequences that contained only novel activation loop motifs and AtMPK4, AtMPK16, OsMPK6, and OsMPK14 from *A*. *thaliana* and *O*. *sativa* as the representative of the T-E-Y and T-D-Y motifs. In the third case, a phylogenetic tree was constructed using all representative MAPKs of *A*. *thaliana*, and *O*. *sativa* with the fungal MAPKs that contained the novel activation loop motifs. In all the three cases, first of all, a clustal file was generated using clustalw or clustal omega program using the default parameters [27,28]. The resulting clustal files were downloaded and converted to MEGA file format using MEGA5 software [29]. Resulting MEGA files of MAPKs were subjected to construct the phylogenetic tree. Different statistical parameters used to construct the phylogenetic trees were as follows. Analysis: phylogeny reconstruction; scope: all selected taxa; statistical parameters: maximum likelihood; test of phylogeny: bootstrap method; number of bootstrap replications: 1000; substitution type: amino acids; model/methods: Jones-Taylor-Thornton (JTT); rates among sites: uniform rates; pattern among lineage: homogenous; gaps/missing data treatment: pair wise deletion/use all sites; and swap filter: very strong.

### Statistical analysis

Tajima’s neutrality test and Tajima’s relative rate test were conducted in order to understand the statistical significance and the rate of evolution of fungal MAPKs [30]. Different statistical parameters used for Tajima’s neutrality test were; analysis, Tajima’s test of neutrality; scope, all selected taxas; substitution type, amino acids; gaps/missing data treatment, pairwise deletion and different statistical parameters that were used to carry out Tajima’s relative rate tests were; analysis, Tajima’s relative rate test; scope, for three chosen sequences; substitution type, amino acids; and gaps/missing data treatment, complete deletion.

### Gene duplication analysis

The duplication event of all the *Saccharomyces cerevisiae* MAPKs were studied using Notung 2.6 software [31,32]. Due to lack of species tree of 173 species, study of gene duplication/loss of all the fungal MAPKs was not feasible. Therefore, we conducted the duplication analysis of fungal MAPKs that contained the novel activation loop motifs, by using the online server Pinda (http://orion.mbg.duth.gr/Pinda) [33]. The fungal MAPKs sequences that resulted in z-score below four were considered as non-duplicated while those resulted in a z-score above four were considered to be duplicated MAPKs [33].

## Results

### Identification of novel activation loop motifs

**Fig 1:**
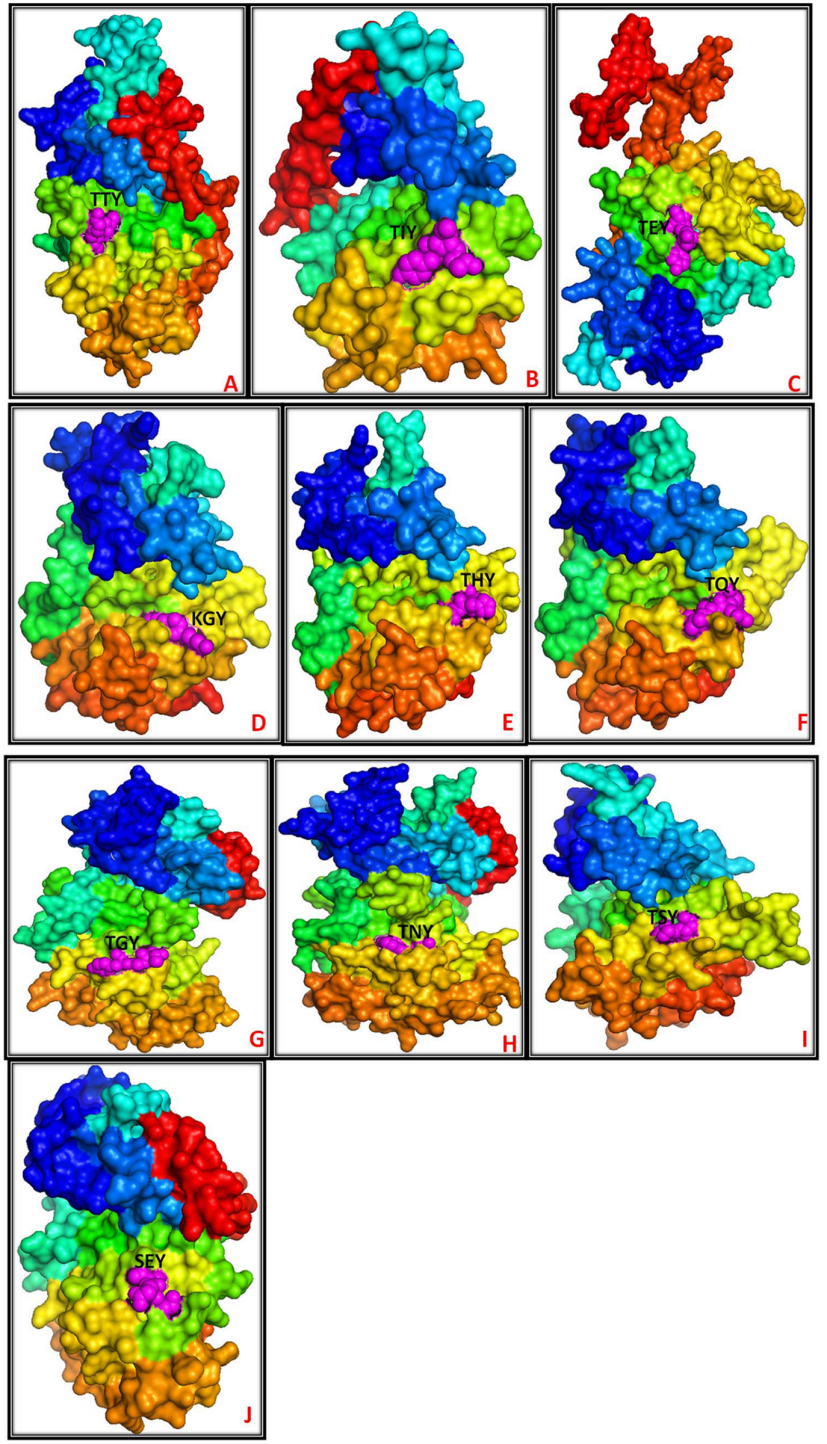
Representative molecular structure of fungal MAPKs with their activation loop motifs. Different activation loop motifs shown in the figure are T-T-Y (AkMAPK), T-I-Y (AlMAPK-A), T-E-Y (CjMAPK), K-G-Y (ScMAPK-A), T-H-Y (LhMAPK), T-Q-Y (UrMAPK), T-G-Y (DeMAPK), T-N-Y (BiMAPK), T-S-Y (HvMAPK) and S-E-Y (AlMAPK-B). The items within the bracket represent MAPKs from different fungal species. The first letter represents the genus name in upper case, and the second letter represents the species name in lower case followed by MPK.

Commonly, the fungal MAPKs contain either a T-E-Y or a T-G-Y motif in the activation loop region [19, 20]. Besides the presence of the activation loop T-E-Y or T-G-Y motif in fungal MAPKs, they were also found to contain several new activation loop motifs (Tables 1 and 2). These newly identified activation loop motifs of fungal MAPKs are T-T-Y, T-I-Y, T-N-Y, T-H-Y, T-S-Y, K-G-Y, T-Q-Y, S-E-Y and S-D-Y (Fig 1) which were never reported before. The newly identified activation loop motifs found among different species are; T-T-Y (*Aplanochytrium kerguelense*), T-I-Y (*Aurantiochytrium limacinum*), T-N-Y (*Ascoidea rubescens*, *Babjeviella inositovora*, *Cyberlindnera jadinii*, *Hansenula polymorpha*, *Hyphopichia burtonii*, *Metschnikowia bicuspidata*, *Pachysolen tannophilus*, *Pichia stipitis*, *Saccharomyces cerevisiae*, *Wickerhamomyces anomalus*, *Xanthoria parietina*), T-H-Y (*Dekkera bruxellensis*, *Lichtheimia hyalospora*, *Mucor circinelloides*, *Nadsonia fulvescens*, *Phycomyces blakesleeanus*, *Rhizopus microsporus*), T-S-Y (*Hanseniaspora valbyensis*), K-G-Y (*Saccharomyces cerevisiae*, *Xanthoria parietina*), T-Q-Y (*Umbelopsis ramanniana*), S-E-Y (*Aurantiochytrium limacinum*), and S-D-Y (*Meliniomyces bicolor*). The novel activation loop motif T-N-Y, K-G-Y, and T-S-Y is only present in the species belonged to the Ascomycota group while the novel activation loop motif T-Q-Y was only found in the Mucoromycotina group. The novel activation loop motif T-H-Y is present in the Ascomycota and Mucoromycotina and absent in all other groups (Table 1). The novel activation loop motif S-D-Y is only present in Basidiomycota group. Earlier, it was thought that the “x” in T-x-Y motif is restricted to G (glycine), P (proline), D (aspartate), and E (glutamate) which are belonged to polar, non-polar and negatively charged amino acids respectively. In our study we found that, the “x” amino acid in T-x-Y motif is very dynamic and can be either polar (T-T-Y), non-polar (T-I-Y), positively charged (T-H-Y) and negatively charged (T-E-Y/T-D-Y) amino acid. From the studied 82 species of Ascomycota group, 66 species have MAPKs that contains only two types of motifs i.e. T-E-Y and T-G-Y while seven species (*Babjeviella inositovora*, *Cyberlindnera jadinii*, *Hyphopichia burtonii*, *Metschnikowia bicuspidate*, *Pachysolen tannophilus*, *Pichia stipitis*, and *Wickerhamomyces anomalus*) have MAPKs contains three types of motifs i.e. T-E-Y, T-G-Y and T-N-Y. Along with T-E-Y, T-G-Y, and T-N-Y motifs, K-G-Y motif is present in MAPKs of only two species (*Saccharomyces cerevisiae*, and *Xanthoria parietina*). MAPKs of only one species (*Hanseniaspora valbyensis*) encodes T-E-Y, T-G-Y and T-S-Y motifs while another two species (*Ascoidea rubescens*, *Hansenula polymorpha*) encodes T-E-Y and T-N-Y motif. The activation loop motif T-E-Y, T-G-Y and T-H-Y is encoded by only two species (*Dekkera bruxellensis*, *Nadsonia fulvescens*). The fungal species *Glomerella acutata*, *Melanomma pulvis-pyrius*, and *Neurospora discrete* encodes only T-E-Y motif. Unlike Ascomycota species, from a total of 73 species studied from Basidiomycota group, 23 species encodes only T-E-Y motif in their MAPKs and 49 species encodes T-E-Y and T-G-Y motifs (Table 1). Only one species (*Meliniomyces bicolour*) of Basidiomycota group contains T-E-Y, T-G-Y, and S-D-Y motifs. From the studied 7 species of Mucoromycotina, four species encodes T-E-Y, T-G-Y, T-H-Y motifs, two species encodes T-E-Y and T-G-Y motifs while only one species encodes T-E-Y, T-G-Y, and T-Q-Y motifs. The presence of T-Q-Y motif is unique to Mucoromycotina only. From this analysis, it is clear that, the variability’s of activation loop motifs are much more in Ascomycota and Mucoromycotina.

**Table 2:** Distribution of the different MAPK activation loop motifs in different groups of fungal MAPKs. The activation loop T-E-Y and T-G-Y motifs are distributed in all four groups. The activation loop motif T-S-Y is unique to group A. The activation loop motif T-H-Y is distributed in groups A and B. The activation loop motifs S-D-Y, S-E-Y, T-T-Y and T-I-Y are unique to group D, while the K-G-Y motif is unique to groups A and C.

Fungal MAPK Groups	Activation loop motifs
Group A (red)	TEY, TGY, TNY, KGY, THY, TSY
Group B (lime)	TEY, TGY, TQY, THY,
Group C (magenta)	TEY, TGY, TNY, KGY
Group D (blue)	TEY, TGY, TIY, TTY, SEY, SDY

The numbers of MAPK gene members in fungi are diverse and varies from species to species (Table 1). The species *Laccaria amethystine* encodes for a maximum of 20 MAPKs in its genome. From the studied 173 species, 31 species encode for 10 or more than 10 MAPKs in their genome (Table 1). A majority of the fungal species putatively encodes six (40 species), seven (23 species) or eight (20 species) MAPKs in their genomes (Table 1). From 73 species of Basidiomycota, 29 species encodes 10 or more than 10 MAPKs in their genome. Similarly, from 82 species of Ascomycota, only one species (*Thielavia appendiculata*) encodes 10 MAPKs in its genome. The MAPK gene family size of Basidiomycota is bigger than Ascomycota.

### Conserved domains and motifs of MAPKs

**Fig 2:**
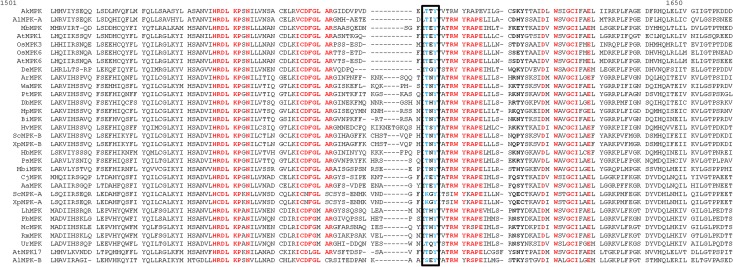
Multiple sequence alignments of fungal MAPKs sequences that show diverse activation loop motifs. The representative fungal MAPKs sequence taken for multiple sequence alignment were from following species; AkMPK (*Aplanochytrium kerguelense*, JGI Sequence ID: 16589); ArMPK *(Ascoidea rubescens*, JGI Sequence ID: 14766); AlMPK-A (*Aurantiochytrium limacinum*-A, JGI Sequence ID: 147180); AlMPK-B (*Aurantiochytrium limacinum*-B JGI Sequence ID: 56966); BiMPK (*Babjeviella inositovora*, JGI Sequence ID: 113741); CjMPK (*Cyberlindnera jadinii*, JGI sequence ID: 176564); DbMPK (*Dekkera bruxellensis*, JGI sequence ID: 5078); HvMPK (*Hanseniaspora valbyensis*, JGI sequence ID: 114746); HpMPK (*Hansenula polymorpha*, JGI sequence ID: 65523); LhMPK (*Lichtheimia hyalospora*, JGI sequence ID: 60177); MbMPK (*Meliniomyces bicolor*, JGI sequence ID: 727596); MbiMPK (*Metschnikowia bicuspidata*, sequence ID: 219362); McMPK (*Mucor circinelloides*, JGI sequence ID: 167765); PtMPK (*Pachysolen tannophilus*, sequence ID: 1060); PbMPK (*Phycomyces blakesleeanus*, JGI sequence ID: 29226); PsMPK (*Pichia stipitis*, JGI sequence ID: 62843); RmMPK (*Rhizopus microsporus*, JGI sequence ID: 57923); ScMPK-A (*Saccharomyces cerevisiae*-A, JGI sequence ID: 28309); ScMPK-B (*Saccharomyces cerevisiae*-B, JGI sequence ID: 645859/SLT2); UmMPK (*Umbelopsis ramanniana*, JGI sequence ID: 230955); WaMPK (*Wickerhamomyces anomalus*, JGI sequence ID: 68008); XpMPK (*Xanthoria parietina*-A, JGI sequence ID: 767958); XpMPK (*Xanthoria parietina*-B, JGI sequence ID: 599559); AaMPK (*Acremonium alcalophilum*, JGI sequence ID: 1074946); and DeMPK (*Daldinia eschscholzii*, JGI sequence ID: 376076). Among several MAPKs that contain the novel activation loop motifs, a representative of only one MAPK sequence was taken for multiple sequence alignments. In the naming system of fungal MAPKs, the first letter indicates the genus name and is given the upper case and second letter indicates the first letter of the species and is given lower case followed by MAPK.

In the multiple sequence alignment studies, we found that, the newly identified T-T-Y, T-I-Y, T-N-Y, T-H-Y, T-S-Y, K-G-Y, T-Q-Y, S-E-Y and S-D-Y activation loop motifs of MAPKs are aligned with the commonly found T-E-Y and T-D-Y motifs of *A*. *thaliana*, and *O*. *sativa* (Fig 2). This reflects that the newly identified activation loop motifs are true activation loop motifs of fungal MAPKs. Besides the presence of a conserved activation loop motif, fungal MAPKs also show the presence of N-terminal-and C-terminal-conserved domains. The N-terminal conserved domain of MAPK is H-R-D-L-K-P-N whereas the C-terminal conserved domain is T-R-W-Y-R-A-P [Fig 2, S1 Fig]. The N-terminal-conserved domain is present before the activation loop motif, and the C-terminal-conserved domain is present after the activation loop motif. The ScMPK-A (*S*. *cerevisiae*-A, JGI sequence ID: 151941649 (M3837 strain) and XpMPK (*X*. *parietina*-A, JGI sequence ID: 767958) contains lysine amino acid in K-G-Y motif instead of the threonine amino acid. This shows that, threonine amino acid is replaced by lysine amino acid in the activation loop motif. Lysine amino acid is a positively charged amino acid and a suitable target phosphorylation site of different kinases [34–38]. Presence of lysine amino acid in the activation loop motif in MAPK shows the activation loop motifs are expanding to increase their diversity to get phosphorylated by diverse kinases.

### Phylogeny

**Fig 3:**
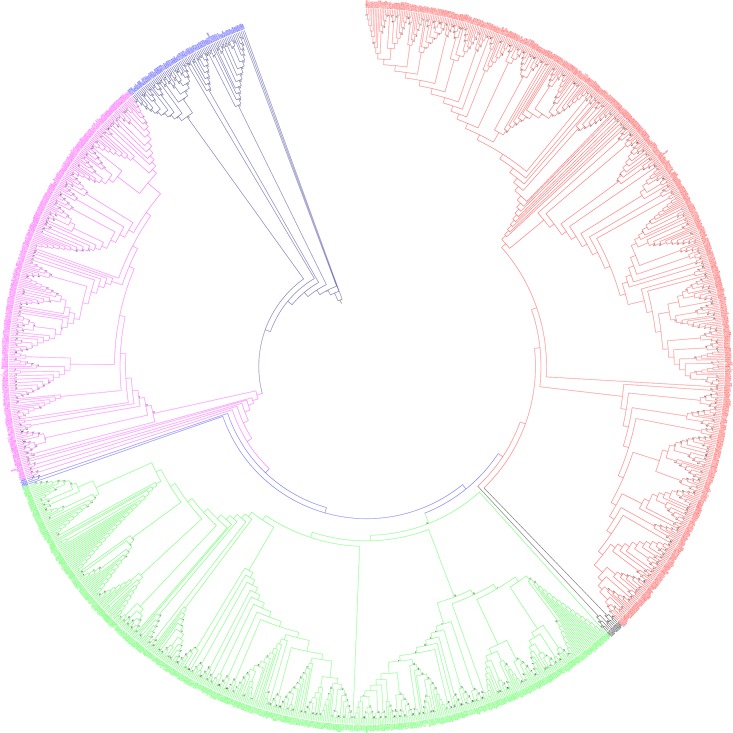
Phylogenetic tree of fungal MAPKs. The phylogenetic tree shows the presence of four major and two minor groups. The major groups are named as group A (red), B (lime), C (magenta) and D (deep blue) and minor groups are named as group E (black) and F (light blue). Group E is present in between group A and B while group F is present in between groups B and C. The majority of MAPK sequences of fungi-like organisms (Oomycetes) are fall in group D (deep blue). The phylogenetic tree was constructed using maximum likelihood statistical method and Jones-Taylor-Thornton model with 1000 bootstrap replicates. The MAPKs sequences of all fungal species can be found in S1 Appendix.

The grouping and classification of different genes in a gene family is very important in order to understand the functional aspects of a gene family. So, grouping of fungal MAPKs was conducted by constructing a phylogenetic tree. The phylogenetic analysis shows that fungal MAPKs are grouped into four major groups and two minor groups. The major groups are named as group A (red), B (lime), C (magenta), and D (deep blue) (Fig 3). The minor groups are named as group E (black) and F (light blue). Group E is present in between groups A and B, and group F is present in between groups B and C. The phylogenetic tree shows that, the fungal MAPK groups are originated polyphyletically. This could have happened due to the fact that the study was conducted with 173 species, which included diverse species, including slime molds. Slime molds are not considered as true fungi, and hence, their common ancestor could be different from other fungi. The classification of the fungi kingdom is changing very rapidly and is yet to acquire a specified classification. Therefore, this may be the reason for the presence of polyphyletic groups in fungal MAPKs.

**Fig 4:**
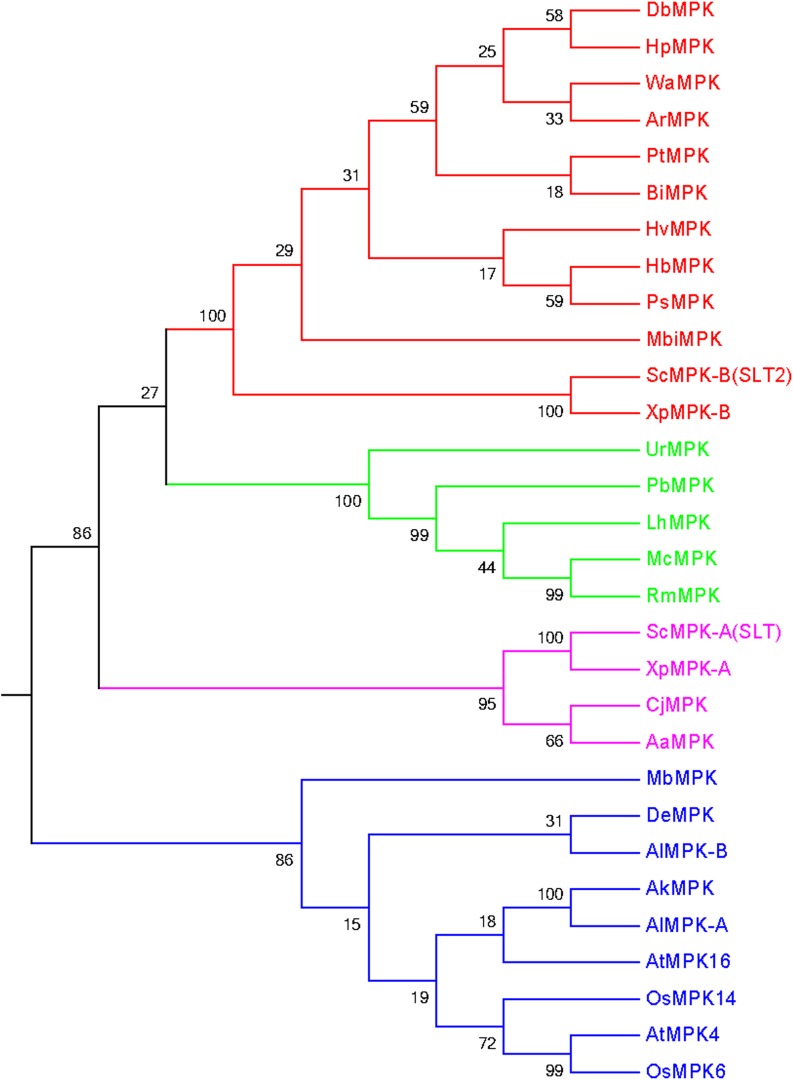
Grouping of fungal MAPKs that contain the novel activation loop motif. The OsMAPKs (OsMPK6, OsMPK14) and AtMAPKs (AtMPK4 and AtMPK16) from four different MAPK groups (one sequence from each group) were taken to provide a proper grouping system to fungal MAPKs that contained the novel activation loop motif. The phylogenetic tree shows that MAPKs are grouped into four different groups. The fungal MAPKs, MbMPK (T-E-Y), DeMPK (T-G-Y), AkMPK (T-T-Y), AlMPK-A (T-I-Y) and ALMPK-B (S-E-Y) are grouped with OsMPK and AtMPK. This reflects that, the MAPKs with this novel activation loop motifs might also be present in plant kingdom too which are yet to be identified. ScMPK in the figure represents SLT2 of *Sachharomyces cerevisiae*.

**Fig 5:**
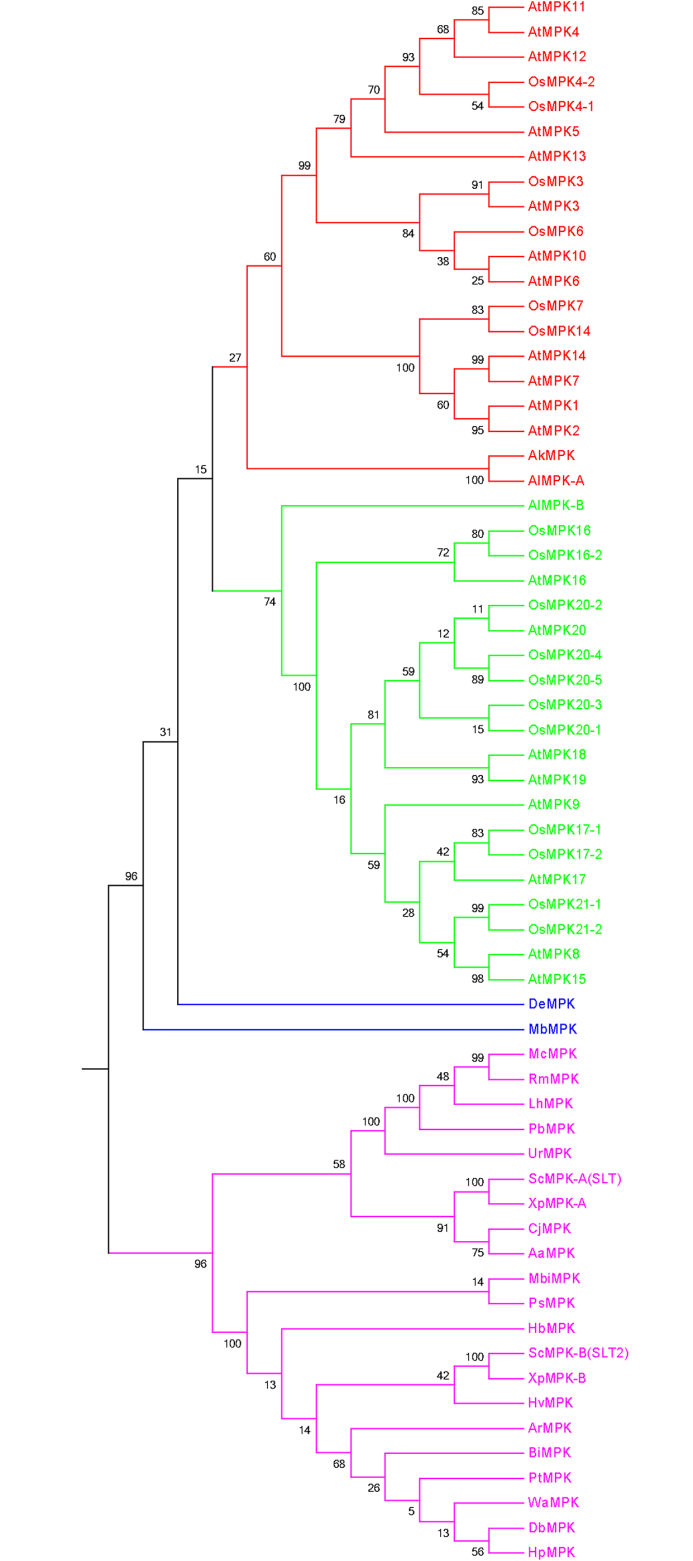
The phylogenetic tree of fungal MAPKs those contain a novel activation loop motif and all representative MAPKs of *A*. *thaliana* and *O*. *sativa*. The phylogenetic tree was constructed by taking all MAPKs of *O*. *sativa* and *A*. *thaliana* with MAPKs of fungi that contains only a “novel” activation loop motif [except one, i.e MbMPK (it contains a T-E-Y motif)]. The phylogenetic tree resulted into four different groups (red and lime color groups are plant-specific and magenta and blue colors are fungi-specific). OsMPKs and AtMPKs were grouped with AkMPK (T-T-Y motif) and AlMPK (T-I-Y motif) of fungi. This suggests that AkMPKs and AlMPKs which contains the T-T-Y and T-I-Y motifs can also be plant-specific as well, which is yet to be elucidated. The other MAPKs having T-N-Y, T-H-Y, T-S-Y, K-G-Y, T-Q-Y and S-E-Y activation loop motifs are those that fall into unique groups, which are specific to fungi only. The activation loop T-E-Y motif is very common and present in all three domains (plant, animal and fungi) of life. Therefore, one MAPK from fungi that contained the T-E-Y (MbMPK) motif was included in this study to better understand their grouping system and phylogenetic relationship. Owing to the presence of the T-E-Y motif, MbMPK is placed in between fungi and plant. Because the T-E-Y motif is common to all the three domains of life and others are unique to fungi, hence MbMPK is present in between the plant and fungi domains. ScMPK in the figure represents SLT2 of *Sachharomyces cerevisiae*.

During the study, we found several fungal MAPKs that contain the novel activation loop motifs. Therefore, it was very important to understand their grouping system and phylogenetic relationship with other common activation loop motifs (T-E-Y and T-D-Y) as well to understand their evolution and subsequent divergence. Therefore, we constructed a phylogenetic tree by including fungal MAPKs with novel activation loop motifs (K-G-Y, T-Q-Y, S-D-Y, T-S-Y, T-T-Y, T-I-Y, S-E-Y, T-H-Y, and T-N-Y), and plant MAPK with common activation loop motifs T-E-Y and T-D-Y. Plant MAPKs were taken from *A*. *thaliana* (AtMPK4, AtMPK16) and *O*. *sativa* (OsMPK6, OsMPK14). The phylogenetic tree resulted in four distinct groups (Fig 4, Table 2). We named them as groups A (red), B (lime), C (magenta), and D (blue). The activation loop motifs T-E-Y, T-G-Y, T-N-Y, K-G-Y, T-H-Y, and T-S-Y, were found to be present in group A; T-E-Y, T-G-Y, T-Q-Y, and T-H-Y in group B; T-E-Y, T-G-Y, T-N-Y, and K-G-Y in group C; and T-E-Y,T-G-Y,T-I-Y, T-T-Y, S-E-Y, T-D-Y and S-D-Y in group D (Table 2). The fungi and plants have close evolutionary relationship. During evolution, fungi have helped the photosynthetic plant lineage to move to terrestrial ecosystem to green the earth in the early Palaeozoic era and played a significant role in assisting colonization of terrestrial environments [39]. Besides, the mutualistic relationships of fungi are much closer to the plants [39]. Therefore, we used the MAPK sequences of plant lineage to construct the phylogenetic tree to understand their evolutionary linkage. Later, we constructed another phylogenetic tree by taking all the representative MAPKs of *A*. *thaliana* and *O*. *sativa* to understand the evolutionary relationship of fungal MAPKs that contain the novel activation loop motifs. The phylogenetic tree resulted into four groups (Fig 5). From the resulting four groups, two groups were shared by fungal MAPKs (magenta and blue), and the other two groups were shared by plant MAPKs (red and lime). Phylogenetic result shows, fungal MAPKs are evolutionarily older than plant MAPKs and are derived from their common ancestors during the process of evolution.

### Statistical analysis

Tajima’s relative rate test explains the test of the molecular evolutionary hypothesis (i.e. a constant rate of molecular evolution) between two samples using an out-group. It applied to nucleotide and protein sequences. Therefore, we conducted Tajima’s relative rate test of fungal MAPKs that contained novel activation loop motif combining with AtMAPKs and OsMAPKs that contained common activation loop motifs. During the analysis, sequences A (OsMPK6) and B (ScMPK-A), with sequence C (CjMPK) were used as random selection by the MEGA program. Results of the analysis show a *p*-value of 0.01004 (*p* = 0.01004), and *X*^*2*^ value of 6.63 (*X*^*2*^ = 6.63) (Table 3). Tajima’s relative rate test with fungal MAPKs that contained a representative of a novel activation loop motif combined with the representative AtMAPKs and OsMAPKs, resulted in a *p* value of 0.0522 (*p* = 0.0522), and *X*^*2*^ test result of 3.77 (*X*^*2*^ = 3.77) (Table 3). The sequences A (DeMPK) and B (AtMPK1), with sequence C (CjMPK) were used during this analysis. When all the MAPKs that contained the novel activation loop motifs were analysed for Tajima’s relative rate test, the resulting *p-* value was 0.0000 *(p* = 0.0000), and *X*^*2*^ value was 97.92 (*X*^*2*^ = 97.92) (Table 3). The sequences A (JGI sequence ID: 687684) and B (JGI sequence ID: 91669), with sequence C (JGI sequence ID: 5240) were used during this analysis as random selections by MEGA software. The *p* value less than 0.05 is often used to reject the null hypothesis of equal rates between the lineages (*p* ≤ 0.01: very strong presumption against the null hypothesis, 0.01< p ≤ 0.05: strong presumption against the null hypothesis, 0.05 < *p* ≤ 0.1 low presumption against the null hypothesis).

**Table 3:** Tajima’s relative rate test. The equality of evolutionary rate analysis between sequences **A** (*OsMPK6*) and **B** (*ScMPK-A*), with sequence **C** (*CjMPK*) were used for the analysis of fungal MAPKs with a few AtMAPKs and OsMAPKs as the representative of T-E-Y and T-D-Y motifs. Sequences **A** (*DeMPK*) and **B** (*AtMPK1*), with sequence **C** (*CjMPK*) were used for fungal MAPKs with all the representative AtMAPKs and OsMAPKs as per default selection on the MEGA program. In the case of all fungal MAPKs that contain the novel activation loop motif, the equality of the evolutionary rate was calculated between sequences A (687684) and B (91669), with sequence C (5240). A *P*-value less than 0.05 is often used to reject the null hypothesis of equal rates between lineages. The analysis involved 3 amino acid sequences. All positions containing gaps and missing datas were eliminated. Evolutionary analyses were conducted in MEGA5.

Configuration	Fungal MAPKs having novel activation loop motifs with few AtMAPKs and OsMAPKs	Fungal MAPKs having novel activation loop motifs with all AtMAPKs and OsMAPKs	All Fungal MAPKs of that contain novel activation loop motif
Identical sites in all three sequences	130	117	193
Divergent sites in all three sequences	83	93	4
Unique differences in Sequence A	64	52	137
Unique differences in Sequence B	38	34	15
Unique differences in Sequence C	20	38	1
*P*-value	0.01004	0.0522	0.0000
*X*^*2*^ test	6.63	3.77	97.92
Degree of freedom	1	1	1

Tajima’s neutrality test distinguishes between the DNA sequences that evolve randomly (neutrally), and one evolving under a non-random process like a balancing selection. This explains the evolution of a particular gene, or group of genes or a genome, and explains whether they evolved neutrally, or by directional selection, or by balancing selection. Therefore, we conducted Tajima’s neutrality test of fungal MAPKs. When fungal MAPKs that contained novel activation loop motifs were analysed in combination with a few AtMAPKs and OsMAPKs (two AtMPKs and two OsMAPKs) as the representative of T-E-Y and T-D-Y motif, the Tajima’s D test result was 5.189926 (Table 4). When fungal MAPKs that contained novel activation loop motifs were analysed in combination with all AtMAPKs and OsMAPKs of *A*. *thaliana* and *O*. *sativa*, the Tajima’s D result was 5.233833 (Table 4). When all the fungal MAPKs of 173 species were analysed for Tajima’s neutrality test, the resulting D value was -3.500934. When fungal MAPKs that contained only novel activation loop motifs were analysed, the resulting D value was 4.7514 (D = 4.751476). As per thumb rule presumption, D value greater than +2 (plus two) or less than -2 (minus two) is considered as highly significant. Therefore, all the results of Tajima’s neutrality test were significant.

**Table 4:** Tajima’s test for neutrality. Statistical analysis was carried out using MEGA5. In the statistical analysis, all the positions with site coverage 95% site coverage were eliminated. That is, fewer than 5% alignment gaps, missing data, and ambiguous bases were allowed at any position. All ambiguous positions were removed for each sequence pair. Abbreviations: m = number of sequences; S = number of segregating sites; P_s_ = S/n; *Θ* = *p*_s_/a_1;_ *π* = nucleotide diversity; and *D* is the Tajima test statistic.

Category	*m*	*S*	*p*_s_	*Θ*	*π*	*D*
All fungal MAPKs	1226	130	0.992366	0.129075	0.290760	3.500934
Only novel activation loop motif fungal MAPKs	24	265	0.880399	0.235761	0.514011	4.751476
Novel motif with two MAPKs of rice and Arabidopsis	30	280	0.906149	0.228730	0.534338	5.189926
Novel activation loop motif fungal MAPKs with all AtMAPKs and OsMAPKs	63	290	0.929487	0.197243	0.489426	5.233833

### Gene duplication analysis

**Fig 6:**
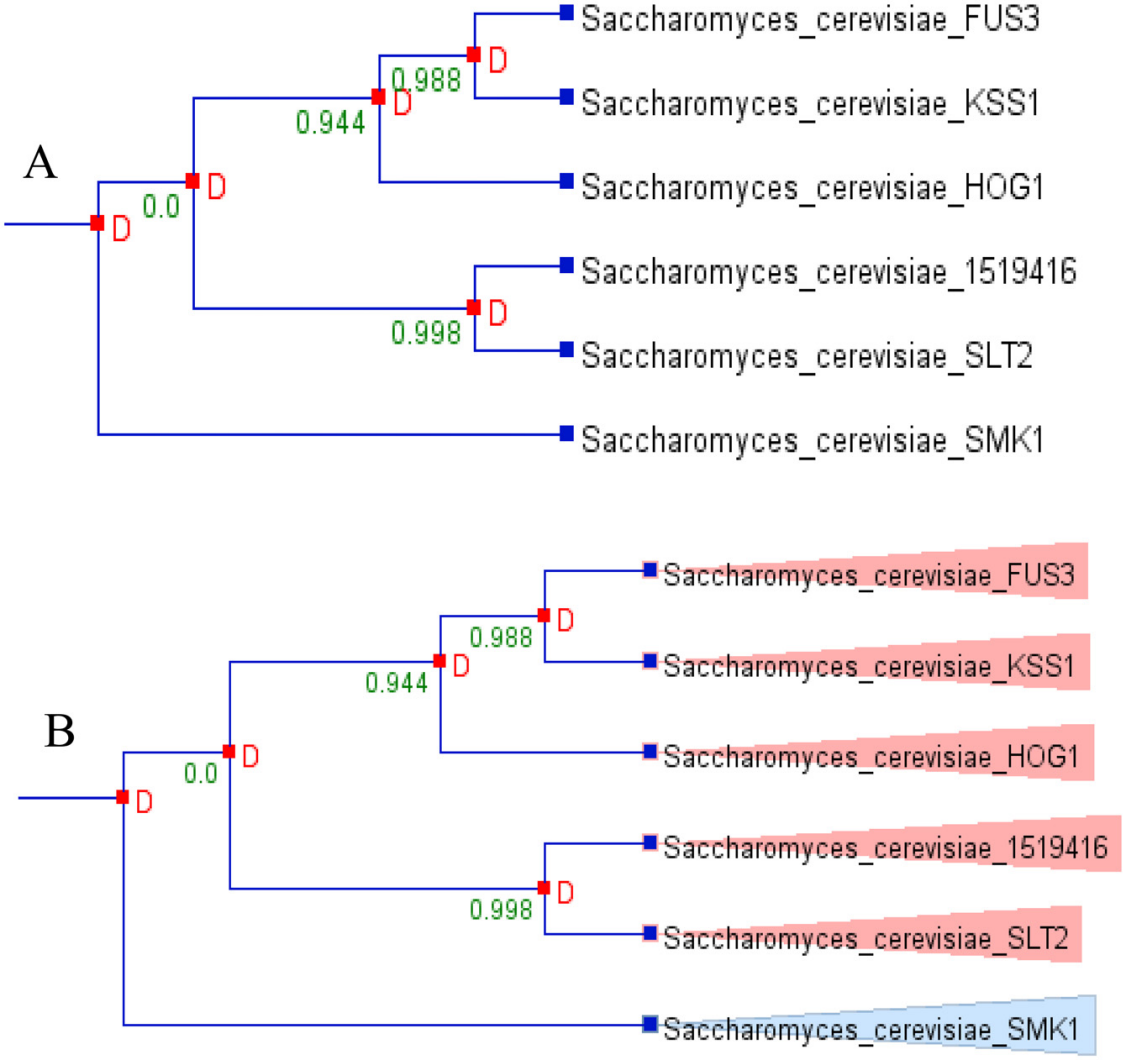
Duplication and orthologous/paralogous analysis of S. *cerevisiae* MAPKs. (A) Duplication analysis shows, *S*. *cerevisiae* MAPKs are highly duplicated. The duplicated MAPKs are Fus3, Kss1, Hog1, 1519416 (uncharacterized) and Slt2 and non duplicated MAPK is Smk1. (B) Orthologous/paralogous gene analysis shows, except Smk1, all other MAPKs are orthologs. The analysis was done using Notung 2.6 software.

Gene duplication is a major mechanism which helps in generating new genetic material during molecular evolution, and this creates the genetic novelty. To understand this phenomena, we analysed the duplication event of the model fungi *S*. *cerevisiae*. From the six MAPKs of *S*. *cerevisiae*, five were found to be duplicated and no MAPK was found to be lost during the evolution (Fig 6). Due to certain limitation and lack of species tree of all the studied (173) fungal species, it was difficult to study the duplication event of all the fungal MAPKs, and hence we restricted out study to *S*. *cerevisiae* only. Still, it was very peculiar to understand the duplication event of the MAPKs that contained the novel activation loop motif. Therefore, we studied the duplication event of MAPKs that contained the novel activation loop by using Pinda server. We found that the MAPKs that contain the most common activation loop motifs T-E-Y, T-G-Y, K-G-Y, T-Q-Y, S-D-Y, and T-S-Y are highly duplicated and contain a z-score of more than four (Table 5). This shows that the most common form of activation loop motif MAPKs is highly duplicated. This result is due to presence of large numbers of evolutionarily conserved orthologous genes (Fig 6). From, six MAPKs of *S*. *cerevisiae*, five were found to be orthologous gene and only one (Smk1) was found to be paralogous (Fig 6). The MAPKs that contain the activation loop motifs T-T-Y, T-I-Y, S-E-Y, T-H-Y and T-N-Y show a z-score of less than four and are found to be non-duplicated.

**Table 5:** Duplication analysis of novel activation loop motif of fungal MAPKs. The most common activation loop motifs T-E-Y, T-G-Y, and K-G-Y of fungal MAPKs have Z-score of 10.86 with 100% confidence level, and are highly duplicated MAPKs. The MAPKs that contain the activation loop motifs T-T-Y, T-I-Y, S-E-Y, T-H-Y and T-N-Y, have a Z-score below four. Genes with Z-score of four or less were not considered as significant enough to be duplicated. Hence, MAPKs that contain Z-score below four are non duplicated MPAKs.

MAPK Genes with Activation loop Motif	Z-score	Level of confidence(%)
T-E-Y	10.86	100
T-G-Y	10.86	100
K-G-Y	10.86	100
T-Q-Y	8.64	100
S-D-Y	7.40	100
T-S-Y	5.99	100
T-T-Y	2.03	95.7
T-I-Y	1.99	95.3
S-E-Y	1.67	90.6
T-H-Y	1.49	86.4
T-N-Y	1.15	75

## Discussion

**Fig 7:**
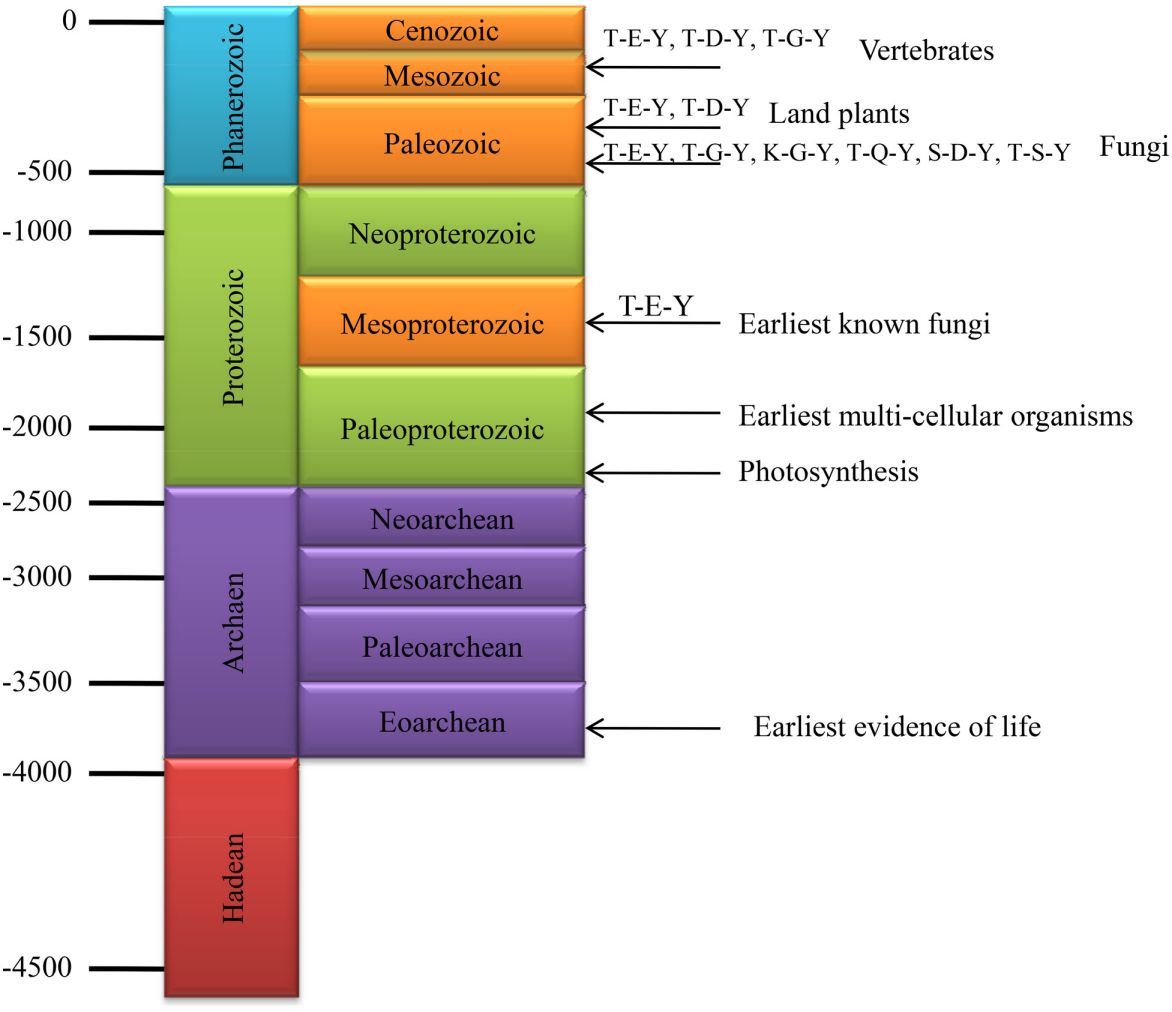
The evolution of mitogen-activated protein kinases (MAPKs). Evolutionary history suggests that fungi have existed since the past 1500 million years. The MAPK that contains T-E-Y motif in activation loop motif is distributed in the plant, animal and fungi kingdoms. So, it can be speculated that the T-E-Y motif containing MAPKs is the most ancient form MAPK, and existed since the past 1500 million years. Except for the T-E-Y motif, T-G-Y, K-G-Y, T-Q-Y, S-D-Y and T-S-Y motifs containing MAPKs have evolved around 4.5 million years ago. The T-D-Y motif containing MAPK is absent in fungi and only present in plants and animals. This suggests that the T-D-Y motif containing MAPKs evolved around four million years ago. All the time scales are based on millions of years.

The origin and evolution of fungi dates back to 1500 million years, when fungi were diverged from other forms of life (Fig 7) [40,41]. Various groups of fungi colonized the land more than 500 million years ago during the Cambrian period [42]. We found that, the presence of T-E-Y motif in MAPK is very common to almost all fungi and presence of the fungal kingdom before 1500 million years directly reflects that, T-E-Y motif of MAPK is the ancient most motif compared to others and might present since 1500 million years ago.

The number of MAPK sequences identified from different fungal species ranges from 2 to 20 (Table 1). The presence of diverse numbers of MAPK sequences and the presence of diverse activation loop motifs in fungal species reflects wide diversity of MAPKs in fungi. The presence of the activation loop T-E-Y motif is common to the plants, animals, and fungi, whereas the presence of the T-G-Y motif is common to the animals and fungi only [2]. Similarly, the activation loop T-D-Y motif is only present in plants and animals. No fungal MAPKs were found to contain any T-D-Y motif in their activation loop region, which is common to plants and animals. The presence of the activation loop T-P-Y motif is unique to animals and fungi [13]. In addition to T-E-Y and T-D-Y motifs in plants, a recent study shows presence of M-E-Y, T-E-M, T-Q-M, T-R-M, T-V-Y, T-S-Y, T-E-C and T-Q-Y motifs in plant MAPKs [16]. The newly identified activation loop motifs of fungal MAPKs are T-T-Y, T-I-Y, T-N-Y, T-H-Y, T-S-Y, K-G-Y, T-Q-Y, S-E-Y and S-D-Y (Figs 1 and 2). The activation loop T-Q-Y motif is present in fungi and animals. The T-S-Y and T-Q-Y motifs are present in plants and fungi. Excluding the T-S-Y and T-Q-Y motifs, all the other seven new activation loop motifs are unique to fungi only. These diverse activation loop MAPK motifs are may be responsible for the diverse kind of functionalities and phosphorylation related signaling events in fungi. The T-I-Y, S-E-Y and T-T-Y motifs found in *A*. *limacinum* and *A*. *kerguelense*, respectively, are specific to the phylum Labyrinthulomycota. The T-H-Y motif is found in both Ascomycota and Zygomycota. The T-N-Y, T-S-Y, and K-G-Y motifs are specific to Ascomycota only. The S-D-Y motif is found in *M*. *bicolour*, which is specific to Basidiomycota, and the T-Q-Y motif found in *U*. *ramanniana* is specific to Zygomycota only. As fungi have also been in existence since the past 1500 million years, it can be possible that fungal MAPKs have also been in existence since the past 1500 million years. The absence of T-D-Y motifs in fungi reflects that, this motif might have evolved recently, and is present only in the plant and animal kingdoms.

**Fig 8:**
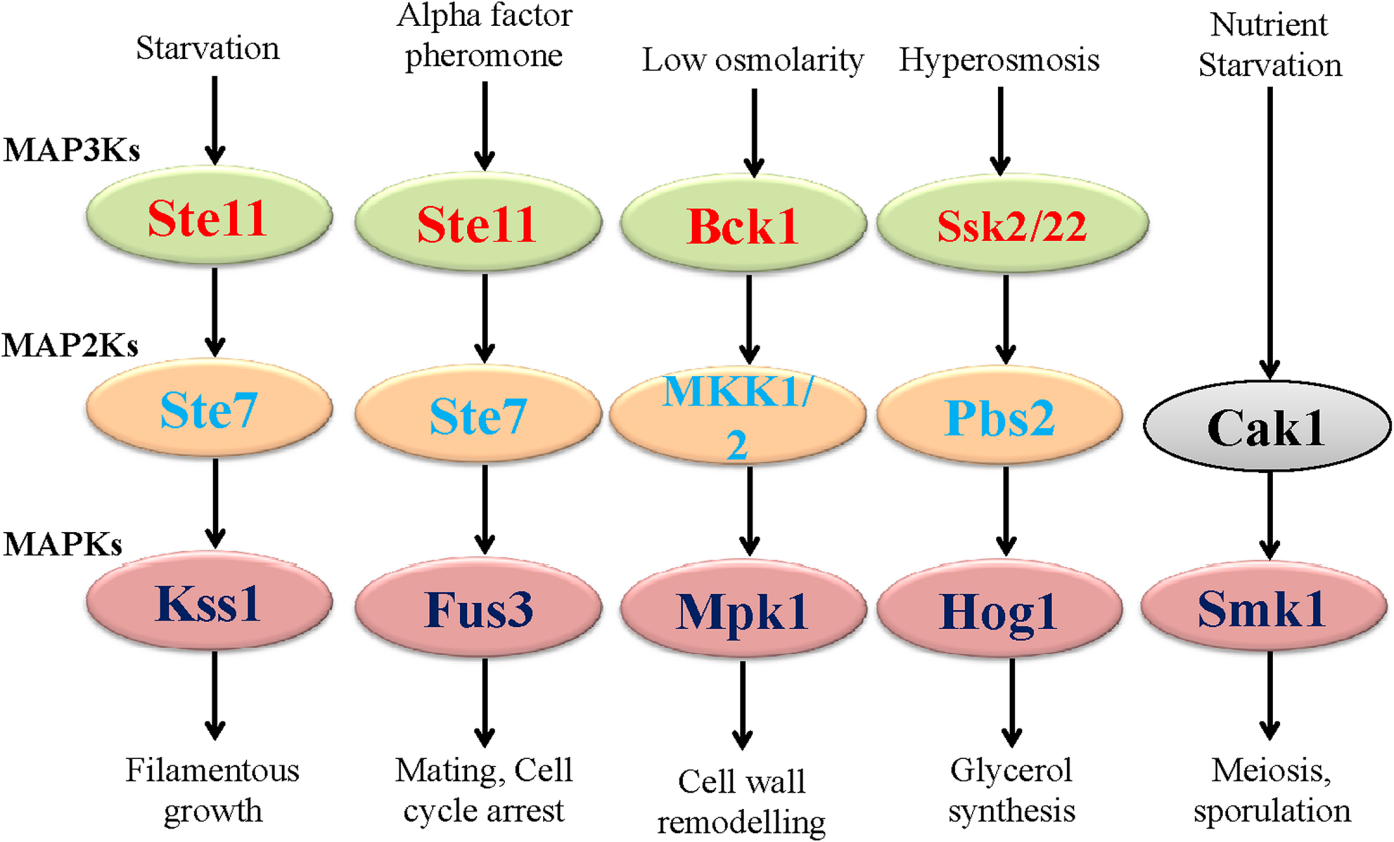
The MAPK cascades in fungi. The Kss1 MAPK that regulates Kss1 pathway is responsible for filamentous growth contains T-E-Y motif in the activation loop domain. The Fus3 MAPK which responsible for mating and cell cycle arrest contains T-E-Y motif in its activation loop domain while Mpk1 which is responsible for cell wall remodeling, contains T-E-Y motif in the activation loop domain. The Hog1 which is responsible for hyperosmosis mediated glycerol synthesis contains T-G-Y motif. The Smk1 which regulates meiosis and sporulation pathway contains T-N-Y motif in the activation loop domain. This suggests that, different signaling pathways mediated by different fungal MAPKs are may be due to presence of different activation loop motifs. The activation loop motif play crucial role in mediating different signaling pathways in fungi.

In fungi, MAPK signaling cascades are regulated through cell integrity (CWI pathway) [43], the high osmolarity glycerol (HOG) pathway, Kss/Fus3 cascade, Mpk1 and SmK1 (sporulation and meiosis) pathways (Fig 8) [18,44–50]. The HOG pathway is crucial for adaptation to osmotic stress and is regulated by accumulation of glycerol in intracellular spaces [51]. During starvation, fungi activate the Kss1 pathway [52–54]. In the Kss1 pathway, the upstream MAPKKK Ste11 activates Ste7 that followed by activation of MAPK Kss1 [55,56]. The Fus3 pathway is involved in mating and cell cycle arrest [48,52,57]. The mating is induced by the presence of specialized pheromones which is sensed by the Ste2 and Ste3 G-protein coupled receptor which, in turn, leads to the activation of MAPK signaling module. The Fus3 and Kss1 MAPK contain T-E-Y motif in the activation loop region. Therefore, the presence of T-E-Y motif in the activation loop region of MAPK that regulates the Fus3 and Kss1 pathways is supposed to be responsible for mating and filamentous growth, respectively. The HOG1 MAPK, which is responsible for hyperosmosis-mediated glycerol synthesis contains T-G-Y motif. This indicates that the presence of the T-G-Y motif in the activation loop motif might mediate critical role for HOG pathway. The Mpk1 pathway is responsible for cell wall remodeling in fungi and it contains T-E-Y motif. The Smk1 protein that mediates Smk1 pathway contains T-N-Y motif. Hence, the presence of the T-N-Y motif in the activation loop might be responsible for regulating Smk1 pathway. The activation loop motif K-G-Y was found in MAPK of *S*. *cerevisiae* and *X*. *parietina*, which are not reported before. Therefore, the presence of mitogen activated protein kinase that contain K-G-Y motif is new to *S*. *cerevisiae*. The presence of K-G-Y motif corroborates to the K-D-X motif of KDX1 protein in *Saccharomyces* genome database. This KDX1 protein is considered as catalytically dead and the presence of the K-G-Y motif in this MAPK is of particular interest. As the tyrosine residue is a potential phosphorylation site of upstream MAP2Ks, the presence of K-G-Y motif in ScMPK-A might be functionally active. Motif specific MAPKs in fungi regulate a different pathway in *S*. *cerevisiae* and presence of K-G-Y motif might regulate some important pathway as well, which is yet to be elucidated. In this context, the presence of several new activation loop motifs in fungal MAPKs seems very interesting, and they might be regulating some novel pathways in fungi. A detailed experimental study with fungal MAPKs that contain a different activation loop motifs may elucidate a new MAPK pathway in fungi.

The mutualistic relationships of fungi are closer to plants, and fungi has helped the plants to colonize the terrestrial environment due to their ecological dominance [39,58]. Beside this, during the evolutionary process of life, the evolutions of plants were followed by evolution of fungi and evolutionary relationships of fungi are much closer to the plants. Therefore, to better understand the evolutionary event of fungal MAPKs, it was highly necessary to conduct the comparative analysis of fungal MAPKs with plant MAPKs. Therefore, MAPKs of *A*. *thaliana* and *O*. *sativa* were included in this study to construct the phylogenetic tree. The resulting phylogenetic tree shows the presence of four distinct groups (Fig 5). The two upper groups belong to MAPKs of *O*. *sativa* and *A*. *thaliana* (except fungal AkMPK and AlMPK that contain the T-T-Y and T-I-Y motifs), and the two lower groups belong to the MAPKs of fungi. Two fungal MAPKs (AkMPK and AlMPK) that contain T-T-Y and T-I-Y motifs, respectively, were found to be grouped with the MAPKs of *A*. *thaliana* and *O*. *sativa* (Fig 5). This shows that the T-T-Y and T-I-Y motifs might be plant-specific as well, which are yet to be reported. The two lower clades of the phylogenetic tree are distinctly separated from the MAPKs of higher plants. This suggests that fungal MAPKs were evolved independently and polyphyletically from their common ancestor and diverged during the evolution, which led to the presence of the diverse activation loop motifs. Earlier it was widely reported that, motif specificity plays important role in grouping of MAPKs [2]. The group D MAPKs of plants and animals always contains T-D-Y motif. But here, we can see that the plant MAPKs of *A*. *thaliana* and *O*. *sativa* with T-D-Y motif don’t grouped together (Fig 5). This clearly explains, T-D-Y motif has no role in grouping of MAPKs and might be applicable to plants and animals only. When MAPKs sequences from all the fungal species were subjected to construct the phylogenetic tree, it resulted in six distinct groups, similar to the MAPK of plant systems (Fig 2) [16]. This explains, although MAPKs of plants and fungal systems were evolved independently from their polyphyletic common ancestors, their basic sequence and group architecture remain conserved. This indicates the conserved and lineage-specific evolution of MAPKs. When the phylogenetic tree was constructed by taking the sequence of MAPKs with the novel activation loop motif group, it resulted in four groups (groups A, B, C and D) (Fig 4, Table 2). The activation loop motifs T-E-Y and T-G-Y were found to be distributed in all four groups while activation loop motif T-N-Y distributed in groups A and C (Table 2). Similarly MAPKs having the activation loop motif T-H-Y were distributed in groups A and B only. The activation loop motifs S-D-Y, S-E-Y, T-T-Y, and T-I-Y are unique to group D; the T-S-Y and T-Q-Y motifs are unique to groups A and B, respectively (Table 2). The K-G-Y activation loop motif is unique to groups A and C.

In the statistical analysis of Tajima’s relative rate test, the *p* value of all the three groups lies between 0.0000 and 0.05, which are considered as significant [30]. The *X*^*2*^ values for all the three groups were also very significant (Table 3). This indicates a high level of significance to Tajima’s test for neutrality (D test). The D values of all the fungal MAPKs studied were found to be -3.500934. In the case of fungal MAPKs that contained only novel activation loop motifs combined with selected AtMAPKs and OsMAPK, the resulting D value was found to be 5.189926 [30,59,60]. Similarly, the D value of fungal MAPKs that contained only novel activation loop motifs was found to be 4.751476 (Table 4). When fungal MAPKs that contained novel activation loop motifs were combined with all representative MAPKs of *A*. *thaliana* and *O*. *sativa*, the resulting D value was 5.233833 (Table 4). A negative Tajima’s D value represents very low frequencies of genetic polymorphism relative to expectation [30,59,60]. This represents an expansion in the population size after the selective and purifying process of selection. A positive Tajima’s D value represents a high level of frequencies of polymorphism [30]. This indicates a decrease in population size by balancing selection. The D value of all fungal MAPKs was found to be positive. This confirms that, overall, fungal MAPKs underwent very high frequencies of genetic polymorphism. The novel activation loop motifs of MAPKs were recently evolved and had undergone a significant genetic polymorphism. When Tajima’s D = 0, theta-Pi is equivalent to theta-k (observed = expected). This implies that the average heterozygosity is equal to the number of segregating sites, or otherwise, it can be explained as an observed variation being similar to expected variation [30,59,60]. This signifies that the population is evolving as per mutation-drift equilibrium and there is no evidence of selection. When Tajima’s D < 0, it indicates a lower average heterozygosity than the number of segregating sites, and rare alleles are present at low frequencies [30,59,60]. This signifies a recent selective sweep and population expansion after a recent bottleneck and linkage to a swept gene. When Tajima’s D > 0, it indicates a more average heterozygosity than the number of segregating sites, and can be present in multiple alleles, some at low and others at high frequencies. This signifies a balancing in selection and sudden contraction of the population [30,59,60]. In the cases of novel activation loop motif fungal MAPKs, and a comparative study with selected *A*. *thaliana* and *O*. *sativa* MAPKs, the D value is greater than zero (D > 0) (Table 4). This confirms that, novel activation loop motif fungal MAPKs and plant MAPKs have multiple alleles present at high frequencies that were responsible for the sudden decrease in population by balancing selection. The D value of all fungal MAPKs together was found to be -3.500934, which is less than zero (D < 0). This explains why rare MAPK alleles are present at very low frequencies and the population expansion after a recent bottleneck. As per thumb rule, the D values greater than +2 or less than -2 are supposed to be significant [30]. In all the cases, the D value was found to be more than +2, and hence; the analyses were highly significant. In the cases of fungal MAPKs that contain the novel activation loop motifs analysed with all representatives of AtMAPKs and OsMAPK, the resulting D value was found to be of 5.233833 (D = 5.233833). This denotes that the novel activation loop containing fungal MAPKs and plant MAPKs are highly polymorphic and evolutionarily conserved, and that they have evolved only recently. In the gene duplication study, the MAPKs that contain the new activation loop motifs T-T-Y, T-I-Y, S-E-Y, T-H-Y and T-N-Y are those that contain a Z-score below four (Table 5). The presence of Z-score of a gene above four is considered as highly significant to be duplicated [33]. Thus, fungal MAPKs that contain these novel activation loop motifs are highly nonduplicated, and most probably evolved only recently, and are yet to undergo any duplication events. The abundance and numbers of MAPK sequences that contain T-T-Y, T-I-Y, S-E-Y, T-H-Y and T-N-Y motifs are very low. This signifies the nonduplication aspects of these MAPKs.

## Conclusion

Genome-wide identification of the MAPK gene family in fungi revealed the presence of several novel activation loop motifs. Evolutionary study shows that fungal MAPKs that contain the T-E-Y motif in the activation loop region are older than other activation loop motifs and MAPKs that contains the T-D-Y motifs are supposed to be evolved recently. The Mpk1, Fus3 and Kss1 pathway is mediated by MAPKs that contains T-E-Y motifs. The HOG1 pathway mediated by MAPK that contains T-G-Y motif and the Smk1 pathway is mediated by MAPK that contains T-N-Y motif. This reflects that, activation loop motif in fungal MAPK decides the fate of different pathways. From this point, we can speculate that, presence of novel activation loop motif MAPKs of fungi might regulate some other novel pathways in different species, which are yet to be elucidated.

## Supporting Information

S1 Appendix Details of fungal MAPKs and JGI accession number used during this study. (ZIP)

S1 Fig Multiple sequence alignments of fungal MAPKs. Results show the presence of the novel activation loop motifs in fungal MAPKs.
(PDF)
