# Supplementary material for: Genome-Wide Identification of Mitogen-Activated Protein Kinase Gene Family across Fungal Lineage Shows Presence of Novel and Diverse Activation Loop Motifs
Source: PLoS One. 2016 Feb 26;11(2):e0149861. doi: 10.1371/journal.pone.0149861 (PMC4769017; doi:10.1371/journal.pone.0149861)
Supplement: S1 Appendix — (ZIP) [file pone.0149861.s001.zip › No. of Identified MAPKs/References.docx]

References

1. Nordberg H, Cantor M, Dusheyko S, Hua S, Poliakov A, et al. (2014) The genome portal of the Department of Energy Joint Genome Institute: 2014 updates. Nucleic Acids Res 42: D26–D31.

2. Grigoriev I V, Nikitin R, Haridas S, Kuo A, Ohm R, et al. (2014) MycoCosm portal: gearing up for 1000 fungal genomes. Nucleic Acids Res 42: D699–D704.
