# Supplementary material for: Genome-Wide Identification of Mitogen-Activated Protein Kinase Gene Family across Fungal Lineage Shows Presence of Novel and Diverse Activation Loop Motifs
Source: PLoS One. 2016 Feb 26;11(2):e0149861. doi: 10.1371/journal.pone.0149861 (PMC4769017; doi:10.1371/journal.pone.0149861)
Supplement: S1 Fig — Results show the presence of the novel activation loop motifs in fungal MAPKs. (PDF) [file pone.0149861.s002.pdf]

```
 757539 ----ADLHAI -------IRS GQPLTDAH-- -------FQS FLYQ------ ---------- ---------- ---------- -----TLCG- ---LKYIHSA NVLHRDLKPG NLLVNADCEL KICDFGLARG YT-------- ----------
 184184 ----ADLHAI -------IRS GQPLTDAH-- -------FQS FLYQ------ ---------- ---------- ---------- -----TLCG- ---LKYIHSA SVLHRDLKPG NLLVNADCEL KICDFGLARG FT-------- ----------
 917307 ----ADLH-A I------IRS GQPLTDAH-- -------FQS FLYQ------ ---------- ---------- ---------- -----TLCG- ---LKYIHSA NVLHRDLKPG NLLVNADCEL KICDFGLARG YT-------- ----------
6355211 ----ADLH-A I------IRS GQPLTDAH-- -------FQS FLYQ------ ---------- ---------- ---------- -----TLCG- ---LKYIHSA NVLHRDLKPG NLLVNADCEL KICDFGLARG YT-------- ----------
 249241 ----ADLH-A I------IRS GQPLTDAH-- -------FQS FIYQ------ ---------- ---------- ---------- -----TLCG- ---LKYIHSA NVLHRDLKPG NLLVNADCEL KICDFGLARG YT-------- ----------
 216822 ----ADLH-A I------IRS GQPLTDAH-- -------FQS FIYQ------ ---------- ---------- ---------- -----TLCG- ---LKYIHSA NVLHRDLKPG NLLVNADCEL KICDFGLARG YT-------- ----------
 805839 ----ADLH-A I------IRS GQPLTDAH-- -------FQS FLYQ------ ---------- ---------- ---------- -----TLCG- ---LKYIHSA NVLHRDLKPG NLLVNADCEL KICDFGLARG FT-------- ----------
 355172 ----ADLH-A I------IRS GQPLTDAH-- -------FQS FLYQ------ ---------- ---------- ---------- -----TLCG- ---LKYIHSA NVLHRDLKPG NLLVNADCEL KICDFGLARG YT-------- ----------
 302825 ----ADLH-A I------IRS GQPLTDAH-- -------FQS FLYQ------ ---------- ---------- ---------- -----TLCG- ---LKYIHSA NVLHRDLKPG NLLVNADCEL KICDFGLARG YT-------- ----------
 239059 ----ADLH-A I------IRS GQPLTDAH-- -------FQS FIYQ------ ---------- ---------- ---------- -----TLCG- ---LKYIHSA NVLHRDLKPG NLLVNADCEL KICDFGLARG YT-------- ----------
 251409 ----ADLH-A I------IRS QQPLSDAH-- -------FQS FIYQ------ ---------- ---------- ---------- -----TLCG- ---LKYIHSA NVLHRDLKPG NLLVNADCEL KICDFGLARG YS-------- ----------
 251443 ----ASQSPA Q------IRS QQPLSDAH-- -------FQS FIYQ------ ---------- ---------- ---------- -----TLCG- ---LKYIHSA NVLHRDLKPG NLLVNADCEL KICDFGLARG YS-------- ----------
  23484 ----ADLHQI -------IRS GQPLTDAH-- -------FQY FVYQ------ ---------- ---------- ---------- -----ICRG- ---LKYIHSA NVLHRDLKPG NLLVNADCEL KICDFGLARG YS-------- ----------
 306334 ----ADLHQI -------IRS EQPLTDAH-- -------FQY FVYQ------ ---------- ---------- ---------- -----ILRG- ---LKYIHSA NVLHRDLKPG NLLVNADCEL KICDFGLARG FS-------- ----------
 306351 ----ADLHQI -------IRS EQPLTDAH-- -------FQY FVYQ------ ---------- ---------- ---------- -----ILRG- ---LKYIHSA NVLHRDLKPG NLLVNADCEL KICDFGLARG FS-------- ----------
 148766 ----ADLHQI -------IRS QQPLTDAH-- -------YQY FIYQ------ ---------- ---------- ---------- -----ICRG- ---LKYIHSA NVIHRDLKPG NLLVNSDCEL KICDFGLARG FS-------- ----------
  84676 ----ADLHQI -------IKS QQPLTDAH-- -------YQY FIYQ------ ---------- ---------- ---------- -----ICRG- ---LKYIHSA NVIHRDLKPG NLLVNSDCEL KICDFGLARG FS-------- ----------
  74082 ----CDLAAI -------IRS GQPLTDAH-- -------FQS FIYQ------ ---------- ---------- ---------- -----ILCG- ---LKYIHSA NVLHRDLKPG NLLVNADCEL KICDFGLARG FS-------- ----------
  97664 ----CDLAAI -------IRS GQPLTDAH-- -------FQS FIYQ------ ---------- ---------- ---------- -----ILCG- ---LKYIHSA NVLHRDLKPG NLLVNADCEL KICDFGLARG FS-------- ----------
  97676 ----CDLAAI -------IRS GQPLTDAH-- -------FQS FIYQ------ ---------- ---------- ---------- -----ILCG- ---LKYIHSA NVLHRDLKPG NLLVNADCEL KICDFGLARG FS-------- ----------
 297954 ----CDLAAI -------IRS GQPLTDAH-- -------FQS FIYQ------ ---------- ---------- ---------- -----ILCG- ---LKYIHSA NVLHRDLKPG NLLVNADCEL KICDFGLARG FS-------- ----------
 323749 ----CDLAAI -------IRS GQPLTDAH-- -------FQS FIYQ------ ---------- ---------- ---------- -----ILCG- ---LKYIHSA NVLHRDLKPG NLLVNADCEL KICDFGLARG FS-------- ----------
1926181 ----CDLAAI -------IRS GQPLTDAH-- -------FQS FIYQ------ ---------- ---------- ---------- -----ILCG- ---LKYIHSA NVLHRDLKPG NLLVNADCEL KICDFGLARG FS-------- ----------
1457364 ----WTFPDP -------ITS TRHIFTRS-- -------SWN AIWQLSSDP- ---------- ---------- ---------- -----ILCG- ---LKYIHSA NVLHRDLKPG NLLVNADCEL KICDFGLARG FS-------- ----------
  78435 ----CDLAAI -------IRS GQPLTDAH-- -------FQS FIYQ------ ---------- ---------- ---------- -----ILCG- ---LKYIHSA NVLHRDLKPG NLLVNADCEL KICDFGLARG FS-------- ----------
1457361         MR -------FGS YHPIRPTA-- -------DRR ---------- ---------- ---------- ---------- --------P- ---LPVLHLP DPLRSQVHP- -LLVNADCEL KICDFGLARG FS-------- ----------
1328540 ----ADLHAI -------IRS GQPLSDAH-- -------FQS FIYQ------ ---------- ---------- ---------- -----TLCG- ---LKYIHSA SVLHRDLKPG NLLVNADCEL KICDFGLARG FE-------- ----------
1364342 ----ADLHAI -------SFI YQTLCGLK-- -------VGY PFYH------ ---------- ---------- ---------- -----RSLGA HEHFQYIHSA SVLHRDLKPG NLLVNADCEL KICDFGLARG FE-------- ----------
 350087 ----ADLHAI -------IRS NQPLSDAH-- -------FQS FLYQ------ ---------- ---------- ---------- -----TLCG- ---LKYIHSA NVLHRDLKPG NLLVNADCEL KICDFGLARG FQ-------- ----------
 288508 ----ADLHAI -------IRS GQPLSDAH-- -------FQS FIYQ------ ---------- ---------- ---------- -----TLCG- ---LRYIHSA SVLHRDLKPG NLLVNADCEL KICDFGLARG FE-------- ----------
 283473 ----ADLHAI -------IRS QQPLSDAH-- -------FQS FIYQ------ ---------- ---------- ---------- -----TLCG- ---LKYIHSA NVLHRDLKPG NLLVNADCEL KICDFGLARG YN-------- ----------
 734869 ----ADLHAI -------IRS GQPLSDAH-- -------YQS FIYQ------ ---------- ---------- ---------- -----TLCG- ---LKYIHSA GVLHRDLKPG NLLVNADCLL KICDFGLARS NT-------- ----------
   9394 ----YDLAKV -------IHS PVQFSEFH-- -------IQN FLYQ------ ---------- ---------- ---------- -----ILCG- ---LKYIHSA DVIHRDLKPG NILITIQGEL KICDFGLARG IN-------- ----------
 167564 ----YDLSKV -------IHS STQFSEFH-- -------IQS FLYQ------ ---------- ---------- ---------- -----ILCG- ---LKYIHSA DVVHRDLKPG NILVTMQGTL KICDFGLARG IS-------- ----------
 113741 ----YDLARV -------IHS AVQFSEFH-- -------VQS FLYQ------ ---------- ---------- ---------- -----ILCG- ---LKYIHSA DVIHRDLKPG NILVTTQGSI KICDFGLARG VN-------- ----------
   6575 ----YDLARV -------IHS AVQFSEFH-- -------VQS FLYQ------ ---------- ---------- ---------- -----ILCG- ---LKYIHSA DVIHRDLKPG NILVTTQGSI KICDFGLARG VN-------- ----------
  30703 ----YDLAKV -------IHS SVQFSEFH-- -------IQC FLYQ------ ---------- ---------- ---------- -----ILCG- ---LKYIHSA DVIHRDLKPG NILVTSQGVL KICDFGLARG IS-------- ----------
  65523 ----YDLAKV -------IHS SVQFSEFH-- -------IQC FLYQ------ ---------- ---------- ---------- -----ILCG- ---LKYIHSA DVIHRDLKPG NILVTSQGVL KICDFGLARG IS-------- ----------
   5078 ----YDLAKV -------IHS SVQFSEYH-- -------IQC FLYQ------ ---------- ---------- ---------- -----ILCG- ---LKYIHSA DVIHRDLKPG NILVTSQGIL KICDFGLARG IN-------- ----------
  54997 ----YDLAKV -------IHS SVQFSEYH-- -------IQC FSYQ------ ---------- ---------- ---------- -----ILCG- ---LKYIHSA DVIHRDLKPG NILVTSQGIL KICDFGLARG IN-------- ----------
 114746 ----YDLARV -------IHS SVQLSEFH-- -------IQS FFYQ------ ---------- ---------- ---------- -----IMCG- ---LKYIHSA DVIHRDLKPG NILCSIQGTL KICDFGLARG MN-------- ----------
 238776 ----YDLSKV -------IYS SSQFSEFH-- -------IKN FMYQ------ ---------- ---------- ---------- -----MLCG- ---LKYIHSA DVIHRDLKPG NILVTVHGVL KICDFGLARG IN-------- ----------
  71410 ----ADLHQI -------IKS GQVLTNEH-- -------VQY FIYQ------ ---------- ---------- ---------- -----ILRG- --RMKYIHSA SVIHRDLKPG NLLVNANCEL KICDFGLSRG F--------- ----------
 676425 ----ADLHQI -------IKS GQSLSNEH-- -------VQY FLYQ------ ---------- ---------- ---------- -----VLRG- ---MKYIHSA SVVHRDLKPG NLLVNADCEL KICDFGLSRG F--------- ----------
 676527 ----ADLHQI -------IKS GQSLSNEH-- -------VQY FLYQ------ ---------- ---------- ---------- -----VLRG- ---MKYIHSA SVVHRDLKPG NLLVNADCEL KICDFGLSRG F--------- ----------
 152706 ----ADLHQI -------IKS GQTLTNEH-- -------VQY FLYQ------ ---------- ---------- ---------- -----ILRG- ---MKYIHSA SVIHRDLKPG NILVNSDCEL KICDFGLSRG F--------- ----------
  88536 ----ADLHQI -------IKS GQTLTNEH-- -------VQY FLYQ------ ---------- ---------- ---------- -----ILRG- ---MKYIHSA SVIHRDLKPG NILVNSDCEL KICDFGLSRG F--------- ----------
 122893 ----ADLHQI -------IKS GQSLTNEH-- -------VQY FLYQ------ ---------- ---------- ---------- -----ILRG- ---MKYVHSA SVVHRDLKPG NLLVNADCEL KICDFGLARG F--------- ----------
 151264 ----ADLHQI -------IKS GQSLTIEH-- -------VQY FVYQ------ ---------- ---------- ---------- -----ILRG- ---LKYIHSA SVVHRDLKPG NLLVNADCEL KICDFGLSRG F--------- ----------
  67588 ----ADLHQI -------IRS GQVLTNEH-- -------VQY FTYQ------ ---------- ---------- ---------- -----ILRG- ---MKFIHSA GVIHRDLKPG NLLVNADCEL KICDFGLSRG Y--------- ----------
 153824 ----ADLYQI -------IRS GQQITNAH-- -------VQY FIYQ------ ---------- ---------- ---------- -----VLRG- ---MKYIHTA HVVHRDLKPG NLLVNSDCEL KICDLGLSRG Y--------- ----------
 103584 ----TDLYQI -------IRS GQQITNAH-- -------VQY FIYQ------ ---------- ---------- ---------- -----VLRG- ---MKYIHTA HVVHRDLKPG NLLVNSDCEL KICDLGLSRG Y--------- ----------
 118819 ----ADLHQI -------IKS GQVLTNEH-- -------VQY FIYQ------ ---------- ---------- ---------- -----ILRG- ---MKYIHSA SVIHRDLKPG NLLVNANCEL KICDFGLSRG F--------- ----------
7227248 ----SDLHQI -------IRS GQTLTNEH-- -------VQY FTYQ------ ---------- ---------- ---------- -----ILRG- ---MKFIHSA GVIHRDLKPG NLLVNADCEL KICDFGLSRG F--------- ----------
 240796 ----ADLHQI -------IKS GQSLTNEH-- -------VQY FTYQ------ ---------- ---------- ---------- -----ILRG- ---MKYIHSA GVIHRDLKPG NLLVNADCEL KICDFGLSRG Y--------- ----------
 366893 ----ADLHQI -------IKS GQVLTNEH-- -------VQY FTYQ------ ---------- ---------- ---------- -----ILRG- ---MKFIHSA GVIHRDLKPG NLLVNADCEL KICDFGLSRG Y--------- ----------
  79368 ----ADLHQI -------IRS GQTLTNEH-- -------VQY FTYQ------ ---------- ---------- ---------- -----ILRG- ---MKYIHSA GVIHRDLKPG NLLVNADCEL KICDFGLSRG Y--------- ----------
 119618 ----ADLHQI -------IKS GQTLTNEH-- -------VQY FLYQ------ ---------- ---------- ---------- -----ILRG- ---MKYVHSA SVIHRDLKPG NLLVNSDCEL KICDFGLSRG F--------- ----------
 500495 ----ADLHQI -------IKS GQHLTNEH-- -------VQY FLYQ------ ---------- ---------- ---------- -----ILRG- ---MKYVHSA SVIHRDLKPG NLLVNADCEL KICDFGLSRG F--------- ----------
 449465 ----ADLHQI -------IKS GQHLTNEH-- -------VQY FLYQ------ ---------- ---------- ---------- -----ILRG- ---MKYVHSA SVIHRDLKPG NLLVNADCEL KICDFGLSRG F--------- ----------
 509502 ----ADLHQI -------IKS GQQLTNEH-- -------VQY FLYQ------ ---------- ---------- ---------- -----VLRG- ---MKYIHSA DVIHRDLKPG NLLVNADCEL KICDFGLSRG F--------- ----------
 964561 ----ADLHQI -------IKS GQTLSNEH-- -------VQY FLYQ------ ---------- ---------- ---------- -----ILRG- ---MKYIHSA SVIHRDLKPG NLLVNSDCEL KICDFGLSRG F--------- ----------
 261448 ----ADLHQI -------IKS GQTLTNEH-- -------VQY FLYQ------ ---------- ---------- ---------- -----ILRG- ---MKYIHSA SVIHRDLKPG NLLVNSDCEL KICDFGLSRG F--------- ----------
1150169 EHGTADLHQI -------IKS GQQLTNEH-- -------VQY FLYQ------ ---------- ---------- ---------- -----ILRG- ---MKYVHSA CVIHRDLKPG NLLVNSDCEL KICDFGLSRG F--------- ----------
 152363 --MEADLHQI -------IKS GQVLTNEH-- -------VQY FLYQ------ ---------- ---------- ---------- -----ILRG- ---MKFIHSA GVIHRDLKPG NLLVNADCEL KICDFGLSRG F--------- ----------
 379740 TVLQADLHQI -------IKS GQVLTNEH-- -------VQY FLYQ------ ---------- ---------- ---------- -----ILRG- ---MKFIHSA GVIHRDLKPG NLLVNADCEL KICDFGLSRG F--------- ----------
 337664 ---------- ---------S GQVLTNEH-- -------VQY FLYQ------ ---------- ---------- ---------- -----ILRG- ---MKFIHSA GVIHRDLKPG NLLVNADCEL KICDFGLSRG F--------- ----------
 434295 --MEADLHQI -------IKS GQVLTNEH-- -------VQY FLYQ------ ---------- ---------- ---------- -----ILRG- ---MKFIHSA GVIHRDLKPG NLLVNADCEL KICDFGLSRG F--------- ----------
 517048 ----ADLHQI -------IKS GQSLSNEH-- -------VQY FLYQ------ ---------- ---------- ---------- -----VLRG- ---MKYIHSA SVVHRDLKPG NLLVNADCEL KICDFGLSRG F--------- ----------
 731201 ----ADLHQI -------IKS GQSLSNEH-- -------VQY FLYQ------ ---------- ---------- ---------- -----VLRG- ---MKYIHSA SVVHRDLKPG NLLVNADCEL KICDFGLSRG F--------- ----------
 768698 ----ADLHQI -------IKS GQSLSNEH-- -------VQY FLYQ------ ---------- ---------- ---------- -----VLRG- ---MKYIHSA SVVHRDLKPG NLLVNADCEL KICDFGLSRG F--------- ----------
 517043 ----ADLHQI -------IKS GQSLSNEH-- -------VQY FLYQ------ ---------- ---------- ---------- -----VLRG- ---MKYIHSA SVVHRDLKPG NLLVNADCEL KICDFGLSRG F--------- ----------
 740533 ----ADLHQI -------IKS GQSLSNEH-- -------VQY FLYQ------ ---------- ---------- ---------- -----VLRG- ---MKYIHSA SVVHRDLKPG NLLVNADCEL KICDFGLSRG F--------- ----------
 178741 ----ADLHQI -------IKS GQALTNEH-- -------VQY FMYQ------ ---------- ---------- ---------- -----VLRG- ---MKYVHSA SVVHRDLKPG NLLVNADCEL KICDFGLARG F--------- ----------
  54225 ----ADLHQI -------IKS GQALTNEH-- -------VQY FMYQ------ ---------- ---------- ---------- -----VLRG- ---MKYVHSA SVVHRDLKPG NLLVNADCEL KICDFGLARG F--------- ----------
 163441 ----ADLHQI -------IKS GQSLTNEH-- -------VQY FMYQ------ ---------- ---------- ---------- -----VLRG- ---MKYIHSA SVVHRDLKPG NLLVNADCEL KICDFGLSRG F--------- ----------
 327966 ----ADLHQI -------IKS GQALTNEH-- -------VQY FVYQ------ ---------- ---------- ---------- -----ILRG- ---MKYIHSA SVVHRDLKPG NLLVNADCEL KICDFGLSRG F--------- ----------
 722194 ----ADLHQI -------IKS GQALSNEH-- -------VQY FVYQ------ ---------- ---------- ---------- -----ILRG- ---MKYVHSA SVVHRDLKPG NLLVNADCEL KICDFGLSRG F--------- ----------
 128404 ----ADLHQI -------IKS GQSLTNEH-- -------VQY FLYQ------ ---------- ---------- ---------- -----ILRG- ---MKYIHSA SVVHRDLKPG NLLVNADCEL KICDFGLARG F--------- ----------
 167758 ----ADLHQI -------IKS GQSLTIEH-- -------VQY FVYQ------ ---------- ---------- ---------- -----ILRG- ---LKYIHSA SVVHRDLKPG NLLVNADCEL KICDFGLSRG F--------- ----------
  26908 ----ADLHQI -------IKS GQSLTIEH-- -------VQY FVYQ------ ---------- ---------- ---------- -----ILRG- ---LKYIHSA SVVHRDLKPG NLLVNADCEL KICDFGLSRG F--------- ----------
 987811 ----ADLHQI -------IKS GQSLTNEH-- -------VQY FMYQ------ ---------- ---------- ---------- -----ILRG- ---MKYVHSA SVIHRDLKPG NLLVNSDCEL KICDFGLSRG F--------- ----------
 913595 ----ADLHQI -------IKS GQSLTNEH-- -------VQY FMYQ------ ---------- ---------- ---------- -----ILRG- ---MKYVHSA SVIHRDLKPG NLLVNSDCEL KICDFGLSRG F--------- ----------
 922124 ----ADLHQI -------IRS GQSLTNEH-- -------VQY FMYQ------ ---------- ---------- ---------- -----ILRG- ---MKYVHSA SVIHRDLKPG NLLVNSDCEL KICDFGLSRG F--------- ----------
 319286 ----ADLHQI -------IRS GQRLSNSH-- -------IQY FLYQ------ ---------- ---------- ---------- -----ILRG- ---MKYIHSA NVIHRDLKPG NLLVNADCEL KICDFGLARG F--------- ----------
 332677 ----ADLHQI -------VRS GQPLSNPH-- -------IQY FIYQ------ ---------- ---------- ---------- -----LLRG- ---MKYIHSA NVIHRDLKPG NLLVNADCEL KICDFGLARG Y--------- ----------
  75030 ----ADLHMI -------IRS GQQLTNEH-- -------CSY FVYQ------ ---------- ---------- ---------- -----ILRG- ---MKYVHSA NIIHRDLKPG NLLVNSDCEL KLADFGLARS F--------- ----------
 740342 ----ADLHMI -------IRS GQQLTNEH-- -------CSY FVYQ------ ---------- ---------- ---------- -----ILRG- ---MKYVHSA NIIHRDLKPG NLLVNSDCEL KLADFGLARS F--------- ----------
 769030 ----ADLHMI -------IRS GQQLTNEH-- -------CSY FVYQ------ ---------- ---------- ---------- -----ILRG- ---MKYVHSA NIIHRDLKPG NLLVNSDCEL KLADFGLARS F--------- ----------
 493654 ----ADLHQI -------IRS GQKLTNAH-- -------VQY FLYQ------ ---------- ---------- ---------- -----ILRG- ---VKYIHSA NVVHRDIKPG NLLVNADCEL KICDFGLSRG F--------- ----------
  12113 ----ADLHQI -------IRS GQKLTNAH-- -------VQY FLYQ------ ---------- ---------- ---------- -----ILRG- ---VKYIHSA NVVHRDIKPG NLLVNADCEL KICDFGLSRG F--------- ----------
 115011 ----ADLHQI -------IRS GQKLTNAH-- -------VQY FLYQ------ ---------- ---------- ---------- -----ILRG- ---VKYIHSA NVVHRDIKPG NLLVNADCEL KICDFGLSRG F--------- ----------
 192400 ----ADLHQI -------IRS GQKLTNAH-- -------VQY FLYQ------ ---------- ---------- ---------- -----ILRG- ---VKYIHSA NVVHRDIKPG NLLVNADCEL KICDFGLSRG F--------- ----------
 543665 ----ADLHQI -------IRS GQKLTNAH-- -------VQY FLYQ------ ---------- ---------- ---------- -----ILRG- ---VKYIHSA NVVHRDIKPG NLLVNADCEL KICDFGLSRG F--------- ----------
  91520 ----TDLHQI -------IKS GQHLSNEH-- -------VQY FLYQ------ ---------- ---------- ---------- -----VLRG- ---VKYIHSA NVIHRDLKPG NLLVNSDCEL KICDFGLSRG F--------- ----------
 127212 ----ADLHQI -------IKS GQHLSNEH-- -------VQY FLYQ------ ---------- ---------- ---------- -----VLRG- ---VKYIHSA NVIHRDLKPG NLLVNSDCEL KICDFGLSRG F--------- ----------
 438879 ----ADLHQI -------IKS GQTLTNEH-- -------VQY FLYQ------ ---------- ---------- ---------- -----VLRG- ---MKYIHSA CVIHRDLKPG NLLVNADCEL KLADFGLSRG F--------- ----------
 174304 ----ADLHQI -------IKS GQTLTNEH-- -------VQY FLYQ------ ---------- ---------- ---------- -----VLRG- ---MKYIHSA CVIHRDLKPG NLLVNADCEL KLADFGLSRG F--------- ----------
 174289 ----ADLHQI -------IKS GQTLTNEH-- -------VQY FLYQ------ ---------- ---------- ---------- -----VLRG- ---MKYIHSA CVIHRDLKPG NLLVNADCEL KLADFGLSRG F--------- ----------
 244084 ----ADLHQI -------IKS GQQLTNEH-- -------VQY FLYQ------ ---------- ---------- ---------- -----ILRG- ---MKYVHSA CVIHRDLKPG NLLVNSDCEL KICDFGLSRG F--------- ----------
 123741 ----SDLHQI -------IKS GQSLTNEH-- -------VQY FLYQ------ ---------- ---------- ---------- -----ILRG- ---MKYVHSA SVVHRDLKPG NLLVNADCEL KICDFGLSRG F--------- ----------
 172164 ----ADLHQI -------IKS GQSLTNEH-- -------VQY FLYQ------ ---------- ---------- ---------- -----ILRG- ---MKYVHSA SVVHRDLKPG NLLVNADCEL KICDFGLARG F--------- ----------
  40528 VPCTADLHQI -------IKS GQSLTNEH-- -------VQY FLYQ------ ---------- ---------- ---------- -----ILRG- ---MKYIHSA SVVHRDLKPG NLLVNADCEL KICDFGLARG F--------- ----------
  39781 VPCTADLHQI -------IRS GQSLTNEH-- -------VQY FLYQ------ ---------- ---------- ---------- -----ILRG- ---MKYIHSA SVVHRDLKPG NLLVNADCEL KICDFGLARG F--------- ----------
1155690 ---AADLHMI -------IRS GQQLTNEH-- -------CSY FVYQ------ ---------- ---------- ---------- -----ILRG- ---MKYLHSG NIIHRDLKPG NLLVNSDCEL KIADFGLARS F--------- ----------
 247448 ---A-DLHQI -------VRS GQPLSNPH-- -------IQY FIYQ------ ---------- ---------- ---------- -----LLRG- ---MKYIHSA NVIHRDLKPG NLLVNADCEL KICDFGLARG Y--------- ----------
 269975 ----ADLSQI -------IRS GQSLSDAH-- -------LQY FVAQ------ ---------- ---------- ---------- -----ILRG- ---IRYMHSA NIIHRDLKPG NLLVNADCQL KVCDFGLARA YA-------- ----------
 246067 ----ADLSQI -------IRS GQSLSDAH-- -------LQY FVAQ------ ---------- ---------- ---------- -----ILRG- ---IRYMHSA NIIHRDLKPG NLLVNADCQL KVCDFGLARA YAVRAKDGEK VYKSSDSARG
  44396 ---------- ---------- ---------- ---------- ---------- ---------- ---------- ---------- -----ILRG- ---IRYMHSA NIIHRDLKPG NLLVNADCQL KVCDFGLARA YAVRAKDGEK VYKSSDSARG
 111248 ----TDMHRV -------IRT -QDLSDDH-- -------CQY FIYQ------ ---------- ---------- ---------- -----TLRA- ---LKAMHSA NVLHRDLKPS NLLLNANCDL KVCDFGLARS AA-------- ----------
  54538 ----TDMHRV -------IRT -QDLSDDH-- -------AQY FIYQ------ ---------- ---------- ---------- -----TLRA- ---LKALHSA DVIHRDLKPS NLLLNANCDL KVCDFGLARS VK-------- ----------
1341438 ----TDMHRV -------IRT -QELSDDH-- -------CQY FIYQ------ ---------- ---------- ---------- -----TLRA- ---VKALHSA NVLHRDLKPS NLLLNANCDL KVCDFGLARS AT-------- ----------
1267921 ----TDMHRV -------IRT -QELSDDH-- -------CQY FIYQ------ ---------- ---------- ---------- -----TLRA- ---VKALHSA NVLHRDLKPS NLLLNANCDL KVCDFGLARS AT-------- ----------
 692594 ----TDLHRL -------LTS -RPLEKQF-- -------IQY FLYQ------ ---------- ---------- ---------- -----ILRG- ---LKYVHSA GVVHRDLKPS NILVNENCDL KICDFGLAR- ---------- ----------
 384872 ----CDLAAI -------IRS GQPLTDAH-- -------FQS FIYQ------ ---------- ---------- ---------- -----ILCG- ---LKYIHSA NVLHRDLKPG NLLVNADCEL KICDFGLARG F--------- ----------
 419622 ----CDLAAI -------IRS GQPLTDAH-- -------FQS FIYQ------ ---------- ---------- ---------- -----ILCG- ---LKYIHSA NVLHRDLKPG NLLVNADCEL KICDFGLARG F--------- ----------
 328081 ----ADLHAI ---------- ---------- ---------- ---------- ---------- ---------- ---------- ---------- -----YIHSA NVLHRDLKPG NLLVNADCEL KICDFGLARG FQ-------- ----------
  61803 ----TDLYRL -------IYS KQKFGREH-- -------IQY FIYQ------ ---------- ---------- ---------- -----ILRA- ---LKYIHSA NVIHRDLKPS NILLNSNCDL KVCDFGLARG L--------- ----------
  40664 ----TDLYRL -------IYS KQKFGREH-- -------IQY FIYQ------ ---------- ---------- ---------- -----ILRA- ---LKYIHSA NVIHRDLKPS NILLNSNCDL KVCDFGLARG L--------- ----------
 105104 ----TDLNRI -------IYS QQKLSNDH-- -------IQY FIYQ------ ---------- ---------- ---------- -----TLRA- ---LKYIHSA GVLHRDLKPS NLLLNADCDL KVCDFGLSRG V--------- ----------
 147180 ----TDLNRV -------IYS EQKLSDQH-- -------IQF LMFQ------ ---------- ---------- ---------- -----LLSA- ---VHYLATA NVIHRDLKPS NILVNSACEL RVCDFGLARG M--------- ----------
 148245 ----CDLDRI -------VSS NQPLSDQH-- -------AQY FLYQ------ ---------- ---------- ---------- -----ILRG- ---IKYIHSA NVLHRDLKPS NLLVNSNCDL AICDFGLARG V--------- ----------
 146852 ----TDLHRI -------IYS KQKLSDDH-- -------VQY FVYQ------ ---------- ---------- ---------- -----MLRS- ---LKYMHSA GVLHRDLKPS NLLLNSNCDL KVCDFGLARG V--------- ----------
  33818 ----TDLHRI -------IYS KQKLSDDH-- -------VQY FVYQ------ ---------- ---------- ---------- -----MLRS- ---LKYMHSA GVLHRDLKPS NLLLNSNCDL KVCDFGLARG V--------- ----------
 116469 ----TDLHRI -------IYS KQKLSDDH-- -------VQY FVYQ------ ---------- ---------- ---------- -----MLRS- ---LKYMHSA GVLHRDLKPS NLLLNSNCDL KVCDFGLARG V--------- ----------
 503885 ----TDLHAI -------IRS GQALSDQH-- -------YQY FVYQ------ ---------- ---------- ---------- -----LVRG- ---LKWVHSA GIIHRDLKPG NLLVNSDCEL KVCDFGLARQ V--------- ----------
 503914 ----TDLHAI -------IRS GQALSDQH-- -------YQY FVYQ------ ---------- ---------- ---------- -----LVRG- ---LKWVHSA GIIHRDLKPG NLLVNSDCEL KVCDFGLARQ V--------- ----------
6817382 ----ADLHQI -------IRS GQTLTNEH-- -------VQY FTYQ------ ---------- ---------- ---------- -----ILRG- ---MKFIHSA GVIHRDLKPG NLLVNADCEL KICDFGLSRG F--------- ----------
 145980 ----CDLAAI -------IRS GQPLTDAH-- -------FQS FIYQ------ ---------- ---------- ---------- -----ILCG- ---LKYIHSA NVLHRDLKPG NLLVNADCEL KIADFGLAR- ---------- ----------
 328170 ----CDLAAI -------IRS GQPLTDAH-- -------FQS FIYQ------ ---------- ---------- ---------- -----ILCG- ---LKYIHSA NVLHRDLKPG NLLVNADCEL KICDFGLAR- ---------- ----------
 691008 ----CDLAAI -------IRS GQPLTDAH-- -------FQS FIYQ------ ---------- ---------- ---------- -----ILCG- ---LKYIHSA NVLHRDLKPG NLLVNADCEL KICDFGLAR- ---------- ----------
  73316 ----CDLAAI -------IRS GQPLTDAH-- -------FQS FIYQ------ ---------- ---------- ---------- -----ILCG- ---LKYIHSA NVLHRDLKPG NLLVNADCEL KICDFGLAR- ---------- ----------
1219281 ----TFLFFY -------F-S GQPLTDAH-- -------FQS FIYQ------ ---------- ---------- ---------- -----ILCG- ---LKYIHSA NVLHRDLKPG NLLVNADCEL KICDFGLAR- ---------- ----------
```

```
 422525 ----ADLHQI -------IRS EQPLTDAH-- -------YQY FIYQ------ ---------- ---------- ---------- -----ICRG- ---LKYIHSA NVLHRDLKPG NLLVNADCEL KICDFGLAR- ---------- ----------
 212435 ----ADLHQI -------IRS GQPLTDAH-- -------FQY FVYQ------ ---------- ---------- ---------- -----ICRG- ---LKYIHSA NVLHRDLKPG NLLVNADCEL KICDFGLAR- ---------- ----------
 187307 ----PLAH-- ---------- RQPLTDAH-- -------FQS FLYQ------ ---------- ---------- ---------- -----TLCG- ---LKYIHSA NVLHRDLKPG NLLVNADCEL KICDFGLAR- ---------- ----------
 146240 ----ADLHAI -------IRS QQPLSDAH-- -------FQS FIYQ------ ---------- ---------- ---------- -----TLCG- ---LKYIHSA NVLHRDLKPG NLLVNADCEL KICDFGLAR- ---------- ----------
 156221 ----ADLHAI -------IRS GQSLTDSH-- -------YQS FIYQ------ ---------- ---------- ---------- -----TLCG- ---LKYIHSA NVLHRDLKPG NLLLNADCEL KICDFGLAR- ---------- ----------
  48865 ----ADLHAI -------IRS GQPLSDAH-- -------FQS FLYQ------ ---------- ---------- ---------- -----TLCG- ---LKYIHSA NVLHRDLKPG NLLVNADCEL KICDFGLAR- ---------- ----------
 226510 ----ADLHAI -------IRS QQPLSDAH-- -------FQS FIYQ------ ---------- ---------- ---------- -----TLCG- ---LKYIHSA NVLHRDLKPG NLLVNADCEL KICDFGLAR- ---------- ----------
 821431 ----ADLHAI -------IRS GQPLTDAH-- -------FQS FIYQ------ ---------- ---------- ---------- -----TLCG- ---LKYIHSA NVLHRDLKPG NLLVNADCEL KICDFGLAR- ---------- ----------
  12784 ----PDPPG- ---------- TWNFNEVY-- -------LYE ELME------ ---------- ---------- ---------- -----ADLH- ---AIYIHSA NVLHRDLKPG NLLVNADCEL KICDFGLAR- ---------- ----------
 157692 ----TSLASI -------IRS GQPLTDAH-- -------FQS FIYQ------ ---------- ---------- ---------- -----ILCG- ---LKYIHSA NVLHRDLKPG NLLVNADCEL KIADFGLAR- ---------- ----------
  47352 ----TDLNRI -------IYS QQKLSNDH-- -------IQY FIYQ------ ---------- ---------- ---------- -----TLRA- ---LKYIHSA GVLHRDLKPS NLLLNADCDL KVCDFGLSR- ---------- ----------
 136580 ----TDMHRV -------IRT -QELSDDH-- -------CQY FIYQ------ ---------- ---------- ---------- -----TLRA- ---LKAMHSA NVLHRDLKPS NLLLNANCDL KVCDFGLAR- ---------- ----------
 422908 ----TDMHRV -------IRT -QELSDDH-- -------CQY FIYQ------ ---------- ---------- ---------- -----TLRA- ---LKAMHSA NVLHRDLKPS NLLLNANCDL KVCDFGLAR- ---------- ----------
  71188 ----TDMHRV -------IRT -QELSDDH-- -------CQY FIYQ------ ---------- ---------- ---------- -----TLRA- ---LKAMHSA NVLHRDLKPS NLLLNANCDL KVCDFGLAR- ---------- ----------
2049832 ----TDMHRV -------IRT -QDLSDDH-- -------CQY FIYQ------ ---------- ---------- ---------- -----TLRA- ---LKAMHSA NVLHRDLKPS NLLLNANCDL KVCDFGLAR- ---------- ----------
2023270 ----TDMHRV -------IRT -QDLSDDH-- -------CQY FIYQ------ ---------- ---------- ---------- -----TLRA- ---LKAMHSA NVLHRDLKPS NLLLNANCDL KVCDFGLAR- ---------- ----------
  62157 ----TDMHRV -------IRT -QELSDDH-- -------CQY FIYQ------ ---------- ---------- ---------- -----TLRA- ---LKAMHSA NVLHRDLKPS NLLLNANCDL KVCDFGLAR- ---------- ----------
 117327 ----TDMHRV -------IRT -QELSDDH-- -------CQY FIYQ------ ---------- ---------- ---------- -----TLRA- ---LKAMHSA NVLHRDLKPS NLLLNANCDL KVCDFGLAR- ---------- ----------
1420954 ----TDMHRV -------IRT -QDLSDDH-- -------CQY FIYQ------ ---------- ---------- ---------- -----TLRA- ---LKAMHSA NVLHRDLKPS NLLLNANCDL KVCDFGLAR- ---------- ----------
 146600 ----TDMHRV -------IRT -QDLSDDH-- -------CQY FIYQ------ ---------- ---------- ---------- -----TLRA- ---LKAMHSA NVLHRDLKPS NLLLNANCDL KVCDFGLAR- ---------- ----------
 427542 ----TDMHRV -------IRT -QDLSDDH-- -------CQY FIYQ------ ---------- ---------- ---------- -----TLRA- ---LKAMHSA NVLHRDLKPS NLLLNANCDL KVCDFGLAR- ---------- ----------
 524886 ----TDMHRV -------IRT -QDLSDDH-- -------CQY FIYQ------ ---------- ---------- ---------- -----TLRA- ---LKAMHSA NVLHRDLKPS NLLLNANCDL KVCDFGLAR- ---------- ----------
 304061 ----TDMHRV -------IRT -QELSDDH-- -------CQY FIYQ------ ---------- ---------- ---------- -----TLRA- ---LKAMHSA NVLHRDLKPS NLLLNANCDL KVCDFGLAR- ---------- ----------
 262011 ----TDMHRV -------IRT -QDLSDDH-- -------CQY FIYQ------ ---------- ---------- ---------- -----TLRA- ---LKAMHSA NVLHRDLKPS NLLLNANCDL KVCDFGLAR- ---------- ----------
 385460 ----TDMHRV -------IRT -QDLSDDH-- -------CQY FIYQ------ ---------- ---------- ---------- -----TLRA- ---LKAMHSA NVLHRDLKPS NLLLNANCDL KVCDFGLAR- ---------- ----------
 322662 ----TDMHRV -------IRT -QDLSDDH-- -------CQY FIYQ------ ---------- ---------- ---------- -----TLRA- ---LKAMHSA NVLHRDLKPS NLLLNANCDL KVCDFGLAR- ---------- ----------
1531698 ----TDMHRV -------IRT -QDLSDDH-- -------CQY FIYQ------ ---------- ---------- ---------- -----TLRA- ---LKAMHSA NVLHRDLKPS NLLLNANCDL KVCDFGLAR- ---------- ----------
1273298 ----TDMHRV -------IRT -QDLSDDH-- -------CQY FIYQ------ ---------- ---------- ---------- -----TLRA- ---LKAMHSA NVLHRDLKPS NLLLNANCDL KVCDFGLAR- ---------- ----------
 211888 ----TDMHRV -------IRT -QDLSDDH-- -------CQY FIYQ------ ---------- ---------- ---------- -----TLRA- ---LKAMHSA NVLHRDLKPS NLLLNANCDL KVCDFGLAR- ---------- ----------
1140823 ----TDMHRV -------IRT -QDLSDDH-- -------CQY FIYQ------ ---------- ---------- ---------- -----TLRA- ---LKAMHSA NVLHRDLKPS NLLLNANCDL KVCDFGLAR- ---------- ----------
  84032 ----TDMHRV -------IRT -QELSDDH-- -------CQY FIYQ------ ---------- ---------- ---------- -----TLRA- ---LKAMHSA NVLHRDLKPS NLLLNANCDL KVCDFGLAR- ---------- ----------
 337569 ----TDMHRV -------IRT -QDLSDDH-- -------CQY FIYQ------ ---------- ---------- ---------- -----TLRA- ---LKAMHSA NVLHRDLKPS NLLLNANCDL KVCDFGLAR- ---------- ----------
 675946 ----TDMHRV -------IRT -QELSDDH-- -------CQY FIYQ------ ---------- ---------- ---------- -----TLRA- ---LKAMHSA NVLHRDLKPS NLLLNANCDL KVCDFGLAR- ---------- ----------
 188798 ----TDMHRV -------IRT -QELSDDH-- -------CQY FIYQ------ ---------- ---------- ---------- -----TLRA- ---LKAMHSA NVLHRDLKPS NLLLNANCDL KVCDFGLAR- ---------- ----------
 364357 ----TDMHRV -------IRT -QELSDDH-- -------CQY FIYQ------ ---------- ---------- ---------- -----TLRA- ---LKAMHSA NVLHRDLKPS NLLLNANCDL KVCDFGLAR- ---------- ----------
 417259 ----TDMHRV -------IRT -QELSDDH-- -------CQY FIYQ------ ---------- ---------- ---------- -----TLRA- ---LKAMHSA NVLHRDLKPS NLLLNANCDL KVCDFGLAR- ---------- ----------
 391850 ----TDMHRV -------IRT -QELSDDH-- -------CQY FIYQ------ ---------- ---------- ---------- -----TLRA- ---LKAMHSA NVLHRDLKPS NLLLNANCDL KVCDFGLAR- ---------- ----------
 420539 ----TDMHRV -------IRT -QELSDDH-- -------CQY FIYQ------ ---------- ---------- ---------- -----TLRA- ---LKALHSA NVLHRDLKPS NLLLNANCDL KVCDFGLAR- ---------- ----------
1649896 ----TDMHRV -------IRT -QDLSDDH-- -------CQY FIYQ------ ---------- ---------- ---------- -----TLRA- ---LKAMHSA NVLHRDLKPS NLLLNANCDL KVCDFGLAR- ---------- ----------
 280694 ----TDMHRV -------IRT -QDLSDDH-- -------CQY FIYQ------ ---------- ---------- ---------- -----TLRA- ---LKAMHSA NVLHRDLKPS NLLLNANCDL KVCDFGLAR- ---------- ----------
 381396 ----TDMHRV -------IRT -QDLSDDH-- -------CQY FIYQ------ ---------- ---------- ---------- -----TLRA- ---LKAMHSA NVLHRDLKPS NLLLNANCDL KVCDFGLAR- ---------- ----------
 685453 ----TDMHRV -------IRT -QDLSDDH-- -------CQY FIYQ------ ---------- ---------- ---------- -----TLRA- ---LKAMHSA NVLHRDLKPS NLLLNANCDL KVCDFGLAR- ---------- ----------
 284413 ----TDMHRV -------IRT -QDLSDDH-- -------CQY FTYQ------ ---------- ---------- ---------- -----TLRA- ---LKAMHSA NVLHRDLKPS NLLLNANCDL KICDLGLAR- ---------- ----------
 384436 ----TDMHRV -------IRT -QDLSDDH-- -------CQY FIYQ------ ---------- ---------- ---------- -----TLRA- ---LKAMHSA NVLHRDLKPS NLLLNANCDL KVCDFGLAR- ---------- ----------
 907966 ----TDMHRV -------IRT -QDLSDDH-- -------CQY FIYQ------ ---------- ---------- ---------- -----TLRA- ---LKAMHSA NVLHRDLKPS NLLLNANCDL KVCDFGLAR- ---------- ----------
 205730 ----TDMHRV -------IRT -QELSDDH-- -------CQY FVYQ------ ---------- ---------- ---------- -----TLRA- ---LKCLHSA KILHRDLKPS NLLLNSNCDL KVCDFGLAR- ---------- ----------
  86646 ----TDLHRV -------IKT -QDLSDDH-- -------CQY FIYQ------ ---------- ---------- ---------- -----TLRA- ---LKYMHSA NILHRDLKPS NLLLNANCDL KVCDFGLAR- ---------- ----------
   9721 ----TDLHRV -------IRT -QELSDDH-- -------CQY FIYQ------ ---------- ---------- ---------- -----TLRA- ---LKYMHSA NVLHRDLKPS NLLLNANCDL KVCDFGLAR- ---------- ----------
   2612 ----TDLHRV -------IRS -QTLTDDH-- -------CQY FVYQ------ ---------- ---------- ---------- -----TLRA- ---LKALHSA GVLHRDLKPS NLLLNANCDL KVCDFGLAR- ---------- ----------
 102501 ----TDLNRV -------IKT -QKLSDDH-- -------CQY FIYQ------ ---------- ---------- ---------- -----TLRA- ---LKSLHSA NVLHRDLKPS NLLLNANCDL KLCDFGLAR- ---------- ----------
  98652 ----TDLHRV -------IKT -QKLSDDH-- -------CQY FIYQ------ ---------- ---------- ---------- -----TLRA- ---LKALHSA NVLHRDLKPS NLLLNANCDL KICDFGLAR- ---------- ----------
  78159 ----TDLHRV -------IHT -QKLSDDH-- -------CQY FIYQ------ ---------- ---------- ---------- -----TLRA- ---LKALHSA NVLHRDLKPS NLLLNANCDL KICDFGLAR- ---------- ----------
 240194 ----TDLHRV -------IRT -QKLSDDH-- -------IQY FIYQ------ ---------- ---------- ---------- -----TLRA- ---LKSMHSA NVLHRDLKPS NLLLNSNCDL KICDFGLAR- ---------- ----------
 193495 ----TDLHRV -------IKS -QTLSDDH-- -------CQY FIYQ------ ---------- ---------- ---------- -----TLRA- ---LKAMHSA NVLHRDLKPS NLLLNSNCDL KVCDFGLAR- ---------- ----------
 171744 ----TDLHRV -------IKS -QTLSDDH-- -------CQY FIYQ------ ---------- ---------- ---------- -----TLRA- ---LKAMHSA NVLHRDLKPS NLLLNSNCDL KVCDFGLAR- ---------- ----------
   4564 ----TDLSRV -------IRT -QRLSDNH-- -------CQY FLYQ------ ---------- ---------- ---------- -----TLRA- ---LKALHSA NVLHRDLKPS NLLVNANCDL KICDFGLAR- ---------- ----------
   6035 ----TDLHRV -------IRT -QTLSDNH-- -------CQY FIYQ------ ---------- ---------- ---------- -----TLRA- ---LKALHSA NVLHRDLKPS NLLLNSNCDL KICDFGLAR- ---------- ----------
 256278 ----TDMHRV -------IRT -QDLSDDH-- -------CQY FIYQ------ ---------- ---------- ---------- -----TLRA- ---LKAMHSA NVLHRDLKPS NLLLNANCDL KVCDFGLAR- ---------- ----------
 120199 ----TDMHRV -------IRT -QDLSDDH-- -------AQY FIYQ------ ---------- ---------- ---------- -----TLRA- ---LKALHSA DVIHRDLKPS NLLLNANCDL KVCDFGLAR- ---------- ----------
1003994 ----TDMHRV -------IRT -QDLSDDH-- -------AQY FVYQ------ ---------- ---------- ---------- -----TLRA- ---LKALHSA DVIHRDLKPS NLLLNANCDL KVCDFGLAR- ---------- ----------
 145010 ----TDMHRV -------IRT -QDLSDDH-- -------AQY FIYQ------ ---------- ---------- ---------- -----TLRA- ---LKALHSA DVIHRDLKPS NLLLNANCDL KVCDFGLAR- ---------- ----------
 183924 ----TDMHRV -------IRT -QDLSDDH-- -------AQY FIYQ------ ---------- ---------- ---------- -----TLRA- ---LKALHSA DVIHRDLKPS NLLLNANCDL KVCDFGLAR- ---------- ----------
 725911 ----TDMHRV -------IRT -QDLSDDH-- -------AQY FIYQ------ ---------- ---------- ---------- -----TLRA- ---LKALHSA DVIHRDLKPS NLLLNANCDL KVCDFGLAR- ---------- ----------
1166877 ----TDMHRV -------IRT -QDLSDDH-- -------AQY FIYQ------ ---------- ---------- ---------- -----TLRA- ---LKALHSA DVIHRDLKPS NLLLNANCDL KVCDFGLAR- ---------- ----------
 574353 ----TDMHRV -------IRT -QDLSDDH-- -------AQY FIYQ------ ---------- ---------- ---------- -----TLRA- ---LKALHSA DVIHRDLKPS NLLLNANCDL KVCDFGLAR- ---------- ----------
 277341 ----TDMHRV -------IRT -QDLSDDH-- -------AQY FIYQ------ ---------- ---------- ---------- -----TLRA- ---LKALHSA DVIHRDLKPS NLLLNANCDL KVCDFGLAR- ---------- ----------
 965410 ----TDMHRV -------IRT -QDLSDDH-- -------AQY FIYQ------ ---------- ---------- ---------- -----TLRA- ---LKALHSA DVIHRDLKPS NLLLNANCDL KVCDFGLAR- ---------- ----------
1040134 ----TDMHRV -------IRT -QDLSDDH-- -------AQY FIYQ------ ---------- ---------- ---------- -----TLRA- ---LKALHSA DVIHRDLKPS NLLLNANCDL KVCDFGLAR- ---------- ----------
 801239 ----TDMHRV -------IRT -QDLSDDH-- -------AQY FIYQ------ ---------- ---------- ---------- -----TLRA- ---LKALHSA DVIHRDLKPS NLLLNANCDL KVCDFGLAR- ---------- ----------
 457049 ----TDMHRV -------IRT -QDLSDDH-- -------AQY FIYQ------ ---------- ---------- ---------- -----TLRA- ---LKALHSA DVIHRDLKPS NLLLNANCDL KVCDFGLAR- ---------- ----------
 777636 ----TDMHRV -------IRT -QDLSDDH-- -------AQY FVYQ------ ---------- ---------- ---------- -----TLRA- ---LKALHSA DVIHRDLKPS NLLLNANCDL KVCDFGLAR- ---------- ----------
 167531 ----TDMHRV -------IRT -QDLSDDH-- -------AQY FVYQ------ ---------- ---------- ---------- -----TLRA- ---LKALHSA DVIHRDLKPS NLLLNANCDL KVCDFGLAR- ---------- ----------
 179443 ----TDMHRV -------IRT -QDLSDDH-- -------AQY FIYQ------ ---------- ---------- ---------- -----TLRA- ---LKALHSA DVIHRDLKPS NLLLNANCDL KVCDFGLAR- ---------- ----------
 388203 ----TDMHRV -------IRT -QDLSDDH-- -------AQY FVYQ------ ---------- ---------- ---------- -----TLRA- ---LKALHSA DVIHRDLKPS NLLLNANCDL KVCDFGLAR- ---------- ----------
 761034 ----TDMHRV -------IRT -QDLSDDH-- -------AQY FIYQ------ ---------- ---------- ---------- -----TLRA- ---LKALHSA DVIHRDLKPS NLLLNANCDL KVCDFGLAR- ---------- ----------
 117742 ----TDMHRV -------IRT -QDLSDDH-- -------AQY FVYQ------ ---------- ---------- ---------- -----TLRA- ---LKALHSA DVIHRDLKPS NLLLNANCDL KVCDFGLAR- ---------- ----------
 506090 ----TDMHRV -------IRT -QDLSDDH-- -------AQY FIYQ------ ---------- ---------- ---------- -----TLRA- ---LKALHSA DVIHRDLKPS NLLLNSNCDL KVCDFGLAR- ---------- ----------
  21911 ----TDMHRV -------IRT -QDLSDDH-- -------AQY FIYQ------ ---------- ---------- ---------- -----TLRA- ---LKALHSA DVIHRDLKPS NLLLNSNCDL KVCDFGLAR- ---------- ----------
7342346 ----TDMHRV -------IRT -QDLSDDH-- -------AQY FIYQ------ ---------- ---------- ---------- -----TLRA- ---LKALHSA DVIHRDLKPS NLLLNANCDL KVCDFGLAR- ---------- ----------
 435735 ----TDMHRV -------IRT -QDLSDDH-- -------AQY FVYQ------ ---------- ---------- ---------- -----TLRA- ---LKALHSA DVIHRDLKPS NLLLNANCDL KVCDFGLAR- ---------- ----------
 267232 ----TDMHRV -------IRT -QDLSDDH-- -------AQY FIYQ------ ---------- ---------- ---------- -----TLRA- ---LKALHSA DVIHRDLKPS NLLLNANCDL KVCDFGLAR- ---------- ----------
 679497 ----TDMHRV -------IRT -QDLSDDH-- -------AQY FIYQ------ ---------- ---------- ---------- -----TLRA- ---LKALHSA DVIHRDLKPS NLLLNANCDL KVCDFGLAR- ---------- ----------
 457636 ----TDMHRV -------IRT -QDLSDDH-- -------AQY FIYQ------ ---------- ---------- ---------- -----TLRA- ---LKALHSA DVIHRDLKPS NLLLNANCDL KVCDFGLAR- ---------- ----------
1340042 ----TDMHRV -------IRT -QDLSDDH-- -------CQY FIYQ------ ---------- ---------- ---------- -----TLRA- ---MKALHSA DVIHRDLKPS NLLLNANCDL KVCDFGLAR- ---------- ----------
 225932 ----TDMHRV -------IRT -QDLSDDH-- -------CQY FIYQ------ ---------- ---------- ---------- -----TLRA- ---LKALHSA DVIHRDLKPS NLLLNANCDL KVCDFGLAR- ---------- ----------
 346924 ----TDLHRV -------IRT -QDLSDDH-- -------CQY FLYQ------ ---------- ---------- ---------- -----TCRA- ---LKALHSA DIIHRDLKPS NLLLNANCDL KVCDFGLAR- ---------- ----------
 760343 ----TDMHRV -------IRT -QDLSDDH-- -------CQY FIYQ------ ---------- ---------- ---------- -----TLRA- ---MKALHSA DVIHRDLKPS NLLLNANCDL KVCDFGLAR- ---------- ----------
  15147 ----TDLHRV -------IRT -QDLSDDH-- -------SQY FIYQ------ ---------- ---------- ---------- -----TCRA- ---LKALHGA EIVHRDLKPS NLLLNANCDL KVCDFGLAR- ---------- ----------
 108680 ----TDMHRV -------IRT -QDLSDDH-- -------SQY FIYQ------ ---------- ---------- ---------- -----TLRG- ---LKALHSA AVLHRDLKPS NLLLNSNCDL KICDFGLAR- ---------- ----------
 724148 ----TDMHRV -------IRT -QDLSDDH-- -------SQY FIYQ------ ---------- ---------- ---------- -----TLRG- ---LKALHSA AVLHRDLKPS NLLLNSNCDL KICDFGLAR- ---------- ----------
 276168 ----TDMHRV -------IRT -QELSDDH-- -------CQY FIYQ------ ---------- ---------- ---------- -----TLRG- ---LKALHSA QVLHRDLKPS NLLLNANCDL KICDFGLAR- ---------- ----------
 194959 ----TDLHRV -------IRT -QSLTDDH-- -------CQY FVYQ------ ---------- ---------- ---------- -----TLRA- ---LKSIHTA DIVHRDLKPA NLLLNANCDL KVCDFGLAR- ---------- ----------
 345309 ----TDLHRV -------IRT -QHLTDDH-- -------CQY FVYQ------ ---------- ---------- ---------- -----TLRA- ---LKSIHSA DIVHRDLKPA NLLLNANCDL KVCDFGLAR- ---------- ----------
7189608 ----TDLHRV -------IRT -QHLTDDH-- -------CQY FVYQ------ ---------- ---------- ---------- -----TLRA- ---LKSIHSA DIVHRDLKPA NLLLNANCDL KVCDFGLAR- ---------- ----------
 439783 ----TDLHRV -------IRT -QHLTDDH-- -------CQY FVYQ------ ---------- ---------- ---------- -----TLRA- ---LKSIHSA DIVHRDLKPA NLLLNANCDL KVCDFGLAR- ---------- ----------
  73775 ----TDLHRV -------IRT -QHLTDDH-- -------CQY FVYQ------ ---------- ---------- ---------- -----TLRA- ---LKSIHSA DIVHRDLKPA NLLLNANCDL KVCDFGLAR- ---------- ----------
 681486 ----TDLHRV -------IRT -QHLTDDH-- -------CQY FVYQ------ ---------- ---------- ---------- -----TLRA- ---LKSIHSA DIVHRDLKPA NLLLNANCDL KVCDFGLAR- ---------- ----------
 671673 ----TDLHRV -------IRT -QHLTDDH-- -------CQY FVYQ------ ---------- ---------- ---------- -----TLRA- ---LKSIHSA DIVHRDLKPA NLLLNANCDL KVCDFGLAR- ---------- ----------
 829815 ----TDLHRV -------IRT -QSLTDDH-- -------CQY FIYQ------ ---------- ---------- ---------- -----TLRA- ---LKSIHSA DIIHRDLKPA NLLLNANCDL KVCDFGLAR- ---------- ----------
 413627 ----TDLHRV -------IRT -QKLTDDH-- -------CQY FIYQ------ ---------- ---------- ---------- -----TLRA- ---LRSIHSA DIVHRDLKPA NLLVNANCDL KVCDFGLAR- ---------- ----------
 109090 ----TDLHRV -------IRT -QQLTDDH-- -------CQY FIYQ------ ---------- ---------- ---------- -----TLRA- ---LKSIHSA DIVHRDLKPA NLLLNSNCDL KVCDFGLAR- ---------- ----------
  43366 ----TDLHRV -------IRT -QTLSDDH-- -------CQY FIYQ------ ---------- ---------- ---------- -----TLRA- ---LKSIHSA DIVHRDLKPA NLLLNANCDL KVCDFGLAR- ---------- ----------
 706326 ----TDLHRV -------IRT -QQLTDDH-- -------CQY FVYQ------ ---------- ---------- ---------- -----TLRA- ---LKTMHSA DIVHRDLKPA NLLLNANCDL KVCDFGLAR- ---------- ----------
1157202 ----TDLHRV -------IRT -QQLTDDH-- -------CQY FVYQ------ ---------- ---------- ---------- -----TLRA- ---LKTMHSA DIVHRDLKPA NLLLNANCDL KVCDFGLAR- ---------- ----------
 743174 ----TDLHRV -------IRT -QHLTDDH-- -------CQY FIYQ------ ---------- ---------- ---------- -----TLRA- ---LKTMHSA DIVHRDLKPA NLLLNANCDL KVCDFGLAR- ---------- ----------
   5331 ----TDLHRV -------IRT -QQLTDDH-- -------CQY FVYQ------ ---------- ---------- ---------- -----TLRA- ---LKTMHSA DIVHRDLKPA NLLLNANCDL KVCDFGLAR- ---------- ----------
 713967 ----TDLHRV -------IRT -QHLTDDH-- -------CQA SPFR------ ---------- ---------- -CLIASTHRE LMGSSTLRA- ---LKTMHSA DIVHRDLKPA NLLLNANCDL KVCDFGLAR- ---------- ----------
 151043 ----TDLHRV -------IRT -QQLTDDH-- -------CQY FVYQ------ ---------- ---------- ---------- -----TLRA- ---LKTMHSA DIVHRDLKPA NLLLNANCDL KVCDFGLAR- ---------- ----------
 481570 ----TDLHRV -------IRT -QQLTDDH-- -------CQY FVYQ------ ---------- ---------- ---------- -----TLRA- ---LKTMHSA DIVHRDLKPA NLLLNANCDL KVCDFGLAR- ---------- ----------
 104738 ----TDLHRV -------IRT -QQLTDDH-- -------CQY FVYQ------ ---------- ---------- ---------- -----TLRA- ---LKTMHSA DIVHRDLKPA NLLLNANCDL KVCDFGLAR- ---------- ----------
 689474 ----TDLHRV -------IRT -QSLSDDH-- -------CQY FIYQ------ ---------- ---------- ---------- -----VLRA- ---LKSIHSA DIVHRDLKPA NLLLNANCDL KVCDFGLAR- ---------- ----------
 179470 ----TDLHRV -------IRT -QSLSDDH-- -------CQY FIYQ------ ---------- ---------- ---------- -----VLRA- ---LKSIHSA DIVHRDLKPA NLLLNANCDL KVCDFGLAR- ---------- ----------
  31001 ----TDLHRV -------IRT -QELTDDH-- -------CQY FVYQ------ ---------- ---------- ---------- -----ILRA- ---LKSIHSA DIVHRDLKPA NLLLNANCDL KVCDFGLAR- ---------- ----------
 155293 ----ADLHQI -------IRT -QHLTDDH-- -------CEY FIYQ------ ---------- ---------- ---------- -----ILRA- ---IKSMHSA DIVHRDLKPA NILVNQNCDL KVCDFGLAR- ---------- ----------
  83606 ----ADLHQI -------IRT -QHLTDDH-- -------CEY FIYQ------ ---------- ---------- ---------- -----ILRA- ---IKSMHSA DIVHRDLKPA NILVNQNCDL KVCDFGLAR- ---------- ----------
  79026 ----TDLHRV -------IRT -QELSDDH-- -------CQY FIYQ------ ---------- ---------- ---------- -----TLRA- ---LKTLHSA DVLHRDLKPS NLLLNANCDL KICDFGLAR- ---------- ----------
  79053 ----TDLHRV -------IRT -QELSDDH-- -------CQY FIYQ------ ---------- ---------- ---------- -----TLRA- ---LKTLHSA DVLHRDLKPS NLLLNANCDL KICDFGLAR- ---------- ----------
 188294 ----TDLHRV -------IRT -QELSDDH-- -------CQY FIYQ------ ---------- ---------- ---------- -----TLRA- ---LKALHSA DVLHRDLKPS NLLLNANCDL KICDFGLAR- ---------- ----------
 422236 ----TDLHRV -------IRT -QELSDDH-- -------CQY FIYQ------ ---------- ---------- ---------- -----TLRA- ---LKALHSA DVLHRDLKPS NLLLNANCDL KLCDFGLAR- ---------- ----------
  59295 ----TDLHRV -------IRT -QELSDDH-- -------CQY FIYQ------ ---------- ---------- ---------- -----TLRA- ---LKALHSA DVLHRDLKPS NLLLNANCDL KLCDFGLAR- ---------- ----------
  91727 ----TDLHRV -------IRT -QQLSDDH-- -------CQY FIYQ------ ---------- ---------- ---------- -----TLRA- ---LKALHSA DVLHRDLKPS NLLLNANCDL KLCDFGLAR- ---------- ----------
  92869 ----TDLHRV -------IRT -QQLSDDH-- -------CQY FIYQ------ ---------- ---------- ---------- -----TLRA- ---LKALHSA DVLHRDLKPS NLLLNANCDL KLCDFGLAR- ---------- ----------
 513929 ----TDLHRV -------IRT -QQLSDDH-- -------CQY FIYQ------ ---------- ---------- ---------- -----TLRA- ---LKALHSA DVLHRDLKPS NLLLNANCDL KLCDFGLAR- ---------- ----------
 166192 ----TDLHRV -------IRT -QQLSDDH-- -------CQY FIYQ------ ---------- ---------- ---------- -----TLRA- ---LKALHSA DVLHRDLKPS NLLLNANCDL KLCDFGLAR- ---------- ----------
 210022 ----TDLHRV -------IRT -QELSDDH-- -------CQY FIYQ------ ---------- ---------- ---------- -----TLRA- ---LKALHSA DVLHRDLKPS NLLLNANCDL KICDFGLAR- ---------- ----------
 847083 ----TDLHRV -------IRT -QELSDDH-- -------CQY FIYQ------ ---------- ---------- ---------- -----TLRA- ---LKALHSA DVLHRDLKPS NLLLNANCDL KVCDFGLAR- ---------- ----------
 819022 ----TDLHRV -------IRT -QELSDDH-- -------CQY FIYQ------ ---------- ---------- ---------- -----TLRA- ---LKALHSA DVLHRDLKPS NLLLNANCDL KLCDFGLAR- ---------- ----------
  50473 ----TDLHRV -------IRT -QELSDDH-- -------CQY FIYQ------ ---------- ---------- ---------- -----TLRA- ---LKALHSA DVLHRDLKPS NLLLNANCDL KLCDFGLAR- ---------- ----------
 850567 ----TDLHRV -------IKT -QRLSDDH-- -------CQY FIYQ------ ---------- ---------- ---------- -----TLRA- ---LKALHSA DVLHRDLKPS NLLLNANCDL KLCDFGLAR- ---------- ----------
 183076 ----TDLHRV -------IKT -QRLSDDH-- -------CQY FIYQ------ ---------- ---------- ---------- -----TLRA- ---LKALHSA DVLHRDLKPS NLLLNANCDL KLCDFGLAR- ---------- ----------
1114014 ----TDLHRV -------IRT -QQLSDDH-- -------CQY FIYQ------ ---------- ---------- ---------- -----TLRA- ---LKALHSA DILHRDLKPS NLLLNANCDL KLCDFGLAR- ---------- ----------
  47661 ----TDLHRV -------IRT -QELSDDH-- -------CQY FIYQ------ ---------- ---------- ---------- -----TLRA- ---LKALHSA DVLHRDLKPS NLLLNANCDL KLCDFGLAR- ---------- ----------
6536531 ----TDLHRV -------IHT -QELSDDH-- -------CQY FIYQ------ ---------- ---------- ---------- -----TLRA- ---LKTLHSA DVLHRDLKPS NLLLNSNCDL KICDFGLAR- ---------- ----------
```

```
 277624  ----TDLHRV -------IRT -QELSDDH-- -------IQY FTYQ------ ---------- ---------- ---------- -----TLRA- ---LKALHSA DVLHRDLKPS NLLLNSNCDL KICDFGLAR- ---------- ----------
  77457  ----TDLHRV -------IRT -QELSDDH-- -------CQY FIYQ------ ---------- ---------- ---------- -----TLRA- ---LKALHSA DVLHRDLKPS NLLLNSNCDL KICDFGLAR- ---------- ----------
  89431  ----TDLHRV -------IRT -QELSDDH-- -------CQY FIYQ------ ---------- ---------- ---------- -----TLRA- ---LKALHSA DVLHRDLKPS NLLLNSNCDL KICDFGLAR- ---------- ----------
 484632  ----TDLHRV -------IRT -QELSDDH-- -------CQY FIYQ------ ---------- ---------- ---------- -----TLRA- ---LKALHSA DVLHRDLKPS NLLLNSNCDL KICDFGLAR- ---------- ----------
 161998  ----TDMHRV -------IRT -QELSDDH-- -------CQY FIYQVSNITS DFITSIRAIF SLFTASLRAH CDAACPAGAG LDANLTLRA- ---LKALHSA DVLHRDLKPS NLLLNANCDL KLCDFGLAR- ---------- ----------
 324877  ----TDLHRV -------IRT -QTLSDDH-- -------CQY FIYQVRPLPP SL--SIRSGR SLST------ ---------- -----TLRA- ---LKALHSA DVLHRDLKPS NLLLNANCDL KLCDFGLAR- ---------- ----------
 117759  ----TDLHRV -------IRS -QELSDDH-- -------CQY FIYQ------ ---------- ---------- ---------- -----TLRA- ---LKALHSA NVLHRDLKPS NLLLNANCDL KLCDFGLAR- ---------- ----------
 489827  ----TDMHRV -------IRT -QELSDDH-- -------CQY FIYQ------ ---------- ---------- ---------- -----TLRG- ---LKALHSA NVLHRDLKPS NLLLNANCDL KICDFGLAR- ---------- ----------
 115935  ----TDMHRV -------IRT -QELSDDH-- -------CQY FIYQ------ ---------- ---------- ---------- -----TLRG- ---LKALHSA NVLHRDLKPS NLLLNANCDL KICDFGLAR- ---------- ----------
 482781  ----TDMHRV -------IRT -QELSDDH-- -------CQY FIYQ------ ---------- ---------- ---------- -----TLRG- ---LKALHSA NVLHRDLKPS NLLLNANCDL KICDFGLAR- ---------- ----------
 545919  ----TDMHRV -------IRT -QELSDDH-- -------CQY FIYQ------ ---------- ---------- ---------- -----TLRG- ---LKALHSA NVLHRDLKPS NLLLNANCDL KICDFGLAR- ---------- ----------
6536562  ----TDLHRV -------IHT -QELSDDH-- -------CQY FIYQ------ ---------- ---------- ---------- -----TLRA- ---LKTLHSA DVLHRDLKPS NLLLNSNCDL KICDFGLAR- ---------- ----------
 309997  ----TDLHRV -------IRT -QELSDDH-- -------CQY FIYQ------ ---------- ---------- ---------- -----TVRG- ---LKALHTA DVLHRDLKPS NLLLNANCDL KICDFGLAR- ---------- ----------
 316873  ----TDLHRV -------IRT -QELSDDH-- -------CQY FIYQ------ ---------- ---------- ---------- -----TVRG- ---LKALHTA DVLHRDLKPS NLLLNANCDL KICDFGLAR- ---------- ----------
 429267  ----TDLHRV -------IRT -QTLSDDH-- -------CQY FIYQ------ ---------- ---------- ---------- -----TLRA- ---LKALHSA DVLHRDLKPS NLLLNANCDL KLCDFGLAR- ---------- ----------
 103465  ----TDMHQA -------IQT -QTLTDDH-- -------IQY FIYQ------ ---------- ---------- ---------- -----SLRG- ---LKYIHSA GVIHRDLKPS NLLLNSNCDL KICDFGLAR- ---------- ----------
 167039  ----TDLHKA -------IVS -QNLSDDH-- -------VQY FIYQ------ ---------- ---------- ---------- -----TLRG- ---LKALHSA NVIHRDLKPS NLLVNANCDL KICDFGLAR- ---------- ----------
   8637  ----ADLHTV -------IQT -QELSDDH-- -------IKY FLYQ------ ---------- ---------- ---------- -----ILKG- ---VKYLHSC GVIHRDLKPA NLLVNENCDL KICDFGLAR- ---------- ----------
  65963  ----TDLHHV -------IRS -QTLSDDH-- -------VQY LMYQ------ ---------- ---------- ---------- -----ALKG- ---VKCLHSC GVIHRDLKPA NLLVNSNCDL KICDFGLAR- ---------- ----------
 246409  ----TDLHRV -------IRT -QELSDDH-- -------CQY FIYQ------ ---------- ---------- ---------- -----TLRA- ---LKALHSA DVLHRDLKPS NLLLNANCDL KLCDFGLAR- ---------- ----------
 246352  ----TDLHRV -------IRT -QELSDDH-- -------CQY FIYQ------ ---------- ---------- ---------- -----TLRA- ---LKALHSA DVLHRDLKPS NLLLNANCDL KLCDFGLAR- ---------- ----------
 668220  ----TDMHRV -------IRT -QDLSDDH-- -------AQY FIYQ------ ---------- ---------- ---------- -----TLRA- ---LKALHSA DVIHRDLKPS NLLLNANCDL KVCDFGLAR- ---------- ----------
 619500  ----TDMHRV -------IRT -QDLSDDH-- -------AQY FIYQ------ ---------- ---------- ---------- -----TLRA- ---LKALHSA DVIHRDLKPS NLLLNANCDL KVCDFGLAR- ---------- ----------
  64492  ----TDMHRV -------IRT -QDLSDDH-- -------AQY FIYQ------ ---------- ---------- ---------- -----TLRA- ---LKALHSA DVIHRDLKPS NLLLNANCDL KVCDFGLAR- ---------- ----------
  80250  ----TDMHRV -------IRT -QDLSDDH-- -------SQY FIYQ------ ---------- ---------- ---------- -----TLRG- ---LKALHSA AVLHRDLKPS NLLLNSNCDL KICDFGLAR- ---------- ----------
  21917  ----TDMHRV -------IRT -QELSDDH-- -------CQY FIYQ------ ---------- ---------- ---------- -----TLRG- ---LKALHSA NVLHRDLKPS NLLLNANCDL KICDFGLAR- ---------- ----------
 600175  ----TDLHRV -------IRT -QELSDDH-- -------CQY FIYQ------ ---------- ---------- ---------- -----TLRA- ---LKALHSA DVLHRDLKPS NLLLNANCDL KLCDFGLAR- ---------- ----------
 721307  ----TDLHRV -------IRT -QELSDDH-- -------CQY FIYQ------ ---------- ---------- ---------- -----TLRA- ---LKALHSA DVLHRDLKPS NLLLNANCDL KLCDFGLAR- ---------- ----------
 847084  ----TDLHRV -------IRT -QELSDDH-- -------CQY FIYQ------ ---------- ---------- ---------- -----TLRA- ---LKALHSA DVLHRDLKPS NLLLNANCDL KVCDFGLAR- ---------- ----------
 280696  ----TDMHRV -------IRT -QDLSDDH-- -------CQY FIYQ------ ---------- ---------- ---------- -----TLRA- ---LKAMHSA NVLHRDLKPS NLLLNANCDL KVCDFGLAR- ---------- ----------
 908144  ----TDMHRV -------IRT -QDLSDDH-- -------CQY FIYQ------ ---------- ---------- ---------- -----TLRA- ---LKAMHSA NVLHRDLKPS NLLLNANCDL KVCDFGLAR- ---------- ----------
 148879  ----TDMHRV -------IRT -QELSDDH-- -------CQY FIYQ------ ---------- ---------- ---------- -----TLRA- ---LKAMHSA NVLHRDLKPS NLLLNANCDL KVCDFGLAR- ---------- ----------
 102030  ----IDLHRI -------IKT -QPLSEEH-- -------VQY FLYQ------ ---------- ---------- ---------- -----ILRA- ---LKYIHSC NVLHRDLKPA NILLNANCDL KVCDFGLAR- ---------- ----------
  92334  ----IDLHRI -------IKT -QPLSEEH-- -------VQY FLYQ------ ---------- ---------- ---------- -----ILRA- ---LKYIHSC NVLHRDLKPA NILLNANCDL KVCDFGLAR- ---------- ----------
 146634  ----TDLHRV -------IYS KQKLTNDH-- -------IQY FVYQ------ ---------- ---------- ---------- -----TLRA- ---LKYIHSA NVIHRDLKPS NLLLNSNCDL KICDFGLAR- ---------- ----------
  72485  ----TDLHAI -------IRS GQALSDQH-- -------YQY FVYQ------ ---------- ---------- ---------- -----LVRG- ---LKWVHSA GIIHRDLKPG NLLVNSDCEL KVCDFGLAR- ---------- ----------
 124061  ----DHLHLG -------YHQ AAYLGVVHGD L------LQY FVYQ------ ---------- ---------- ---------- -----TLRA- ---LKSIHSA DIVHRDLKPA NLLLNANCDL KVCDFGLAR- ---------- ----------
 109792  ----TDMHRV -------IRT -QDLSDDH-- -------CQY FIYQ------ ---------- ---------- ---------- -----TLRA- ---LKALHSA DVIHRDLKPS NLLLNANCDL KVCDFGLAR- ---------- ----------
 408982  ----TDMHRV -------IRT -QDLSDDH-- -------AQY FVYQ------ ---------- ---------- ---------- -----TLRA- ---LKALHSA DVIHRDLKPS NLLLNANCDL KVCDFGLAR- ---------- ----------
 149279  ----TDMHRV -------IRT -QDLSDDH-- -------AQY FIYQ------ ---------- ---------- ---------- -----TLRA- ---LKALHSA DVIHRDLKPS NLLLNANCDL KVCDFGLAR- ---------- ----------
  13526  ----TDLHRV -------IRT -QELSDDH-- -------CQY FIYQ------ ---------- ---------- ---------- -----TLRA- ---LKALHSA DVLHRDLKPS NLLLNSNCDL KICDFGLAR- ---------- ----------
 465497  ----TDLHRV -------IRT -QELSDDH-- -------CQY FIYQ------ ---------- ---------- ---------- -----TLRA- ---LKALHSA DVLHRDLKPS NLLLNSNCDL KICDFGLAR- ---------- ----------
 216389  ----TDLHRV -------IRT -QELSDDH-- -------CQY FIYQ------ ---------- ---------- ---------- -----TLRA- ---LKALHSA DVLHRDLKPS NLLLNSNCDL KVCDFGLAR- ---------- ----------
  17191  ----TDLHRV -------VRS -QKLSEDH-- -------AQY FVYQ------ ---------- ---------- ---------- -----ILRA- ---LKVMHSA DVIHRDLKPS NLLLNANCDL KVCDFGLAR- ---------- ----------
 530963  ----TDLHRL -------LTS -RPLEKQF-- -------IQY FLYQ------ ---------- ---------- ---------- -----ILRG- ---LKYVHSA GVVHRDLKPS NILVNENCDL KICDFGLAR- ---------- ----------
  84099  ----TDLHRL -------LTS -RPLEKQF-- -------IQY FLYQ------ ---------- ---------- ---------- -----ILRG- ---LKYVHSA GVVHRDLKPS NILVNENCDL KICDFGLAR- ---------- ----------
 928715  ----SLLNAL -------IRT -QGLSDDH-- -------CQY FIYQ------ ---------- ---------- ---------- -----ALRG- ---LKALHSA GILHRDLKPS NLLLNANCDL KICDFGLAR- ---------- ----------
 480104  ----TDLHRV -------IRT -QSLTDDH-- -------CQY FVYQ------ ---------- ---------- ---------- -----TLRA- ---LKSIHTA DIVHRDLKPA NLLLNANCDL KVCDFGLAR- ---------- ----------
  37144  ----TDLHRV -------IRT -QSLSDDH-- -------CQY FIYQ------ ---------- ---------- ---------- -----LLRA- ---LKSIHGA GIVHRDLKPA NLLLNADCDL KVCDFGLAR- ---------- ----------
 993637  ----TDLHRV -------IRT -QSLSDDH-- -------CQY FIYQ------ ---------- ---------- ---------- -----LLRA- ---LKSIHGA GIVHRDLKPA NLLLNADCDL KVCDFGLAR- ---------- ----------
 706327  ----TDLHRV -------IRT -QQLTDDH-- -------CQY FVYQ------ ---------- ---------- ---------- -----TLRA- ---LKTMHSA DIVHRDLKPA NLLLNANCDL KVCDFGLAR- ---------- ----------
   5366  ----TDLHRV -------IRT -QQLTDDH-- -------CQY FVYQ------ ---------- ---------- ---------- -----TLRA- ---LKTMHSA DIVHRDLKPA NLLLNANCDL KVCDFGLAR- ---------- ----------
 144998  ----TDMHRV -------IRT -QDLSDDH-- -------AQY FIYQ------ ---------- ---------- ---------- -----TLRA- ---LKALHSA DVIHRDLKPS NLLLNANCDL KVCDFGLAR- ---------- ----------
 232199  ----TDMHRV -------IRT -QDLSDDH-- -------AQY FIYQ------ ---------- ---------- ---------- -----TLRA- ---LKALHSA DVIHRDLKPS NLLLNANCDL KVCDFGLAR- ---------- ----------
  60405  ----TDMHRV -------IRT -QDLSDDH-- -------AQY FIYQ------ ---------- ---------- ---------- -----TLRA- ---LKALHSA DVIHRDLKPS NLLLNANCDL KVCDFGLAR- ---------- ----------
 572635  ----TDMHRV -------IRT -QDLSDDH-- -------AQY FIYQ------ ---------- ---------- ---------- -----TLRA- ---LKALHSA DVIHRDLKPS NLLLNANCDL KVCDFGLAR- ---------- ----------
  37912  ----TDMHRV -------IRT -QDLSDDH-- -------AQY FVYQ------ ---------- ---------- ---------- -----TLRA- ---LKALHSA DVIHRDLKPS NLLLNANCDL KVCDFGLAR- ---------- ----------
 819552  ----TDMHRV -------IRT -QDLSDDH-- -------AQY FIYQ------ ---------- ---------- ---------- -----TLRA- ---LKALHSA DVIHRDLKPS NLLLNANCDL KVCDFGLAR- ---------- ----------
1026351  ----TDLHRV -------IRT -QELSDDH-- -------CQY FIYQ------ ---------- ---------- ---------- -----TLRA- ---LKALHSA DVLHRDLKPS NLLLNANCDL KICDFGLAR- ---------- ----------
 352542  ----TDLHRV -------IRT -QELSDDH-- -------CQY FIYQ------ ---------- ---------- ---------- -----TLRA- ---LKALHSA DVLHRDLKPS NLLLNANCDL KLCDFGLAR- ---------- ----------
  42114  ----TDLHRV -------IRT -QELSDDH-- -------CQY FIYQ------ ---------- ---------- ---------- -----TLRA- ---LKALHSA DVLHRDLKPS NLLLNSNCDL KVCDFGLAR- ---------- ----------
 204011  ----TDMHRV -------IRT -QELSDDH-- -------CQY FIYQ------ ---------- ---------- ---------- -----TLRG- ---LKALHSA NVLHRDLKPS NLLLNANCDL KICDFGLAR- ---------- ----------
  46962  ----TDMHRV -------IRT -QDLSDDH-- -------CQY FIYQ------ ---------- ---------- ---------- -----TLRA- ---LKAMHSA NVLHRDLKPS NLLLNANCDL KVCDFGLAR- ---------- ----------
   3452  ----TDMHRV -------IRT -QELSDDH-- -------CQY FIYQ------ ---------- ---------- ---------- -----TLRA- ---LKAMHSA NVLHRDLKPS NLLLNANCDL KVCDFGLAR- ---------- ----------
1531701  ----TDMHRV -------IRT -QDLSDDH-- -------CQY FIYQ------ ---------- ---------- ---------- -----TLRA- ---LKAMHSA NVLHRDLKPS NLLLNANCDL KVCDFGLAR- ---------- ----------
 375250        MHRV -------IRT -QELSDDH-- -------CQY FIYQ------ ---------- ---------- ---------- -----TLRA- ---LKAMHSA NVLHRDLKPS NLLLNANCDL KVCDFGLAR- ---------- ----------
 391393  ----TDMHRV -------IRT -QELSDDH-- -------CQY FIYQ------ ---------- ---------- ---------- -----TLRA- ---LKALHSA NVLHRDLKPS NLLLNANCDL KVCDFGLAR- ---------- ----------
 518679  ----TDMHRV -------IRT -QDLSDDH-- -------CQY FIYQ------ ---------- ---------- ---------- -----TLRA- ---LKAMHSA NVLHRDLKPS NLLLNANCDL KVCDFGLAR- ---------- ----------
1272884  ----TDMHRV -------IRT -QDLSDDH-- -------CQY FIYQ------ ---------- ---------- ---------- -----TLRA- ---LKAMHSA NVLHRDLKPS NLLLNANCDL KVCDFGLAR- ---------- ----------
 108249  ----TDLHRV -------IKT -QDLSDDH-- -------CQY FIYQ------ ---------- ---------- ---------- -----TLRA- ---LKYMHSA NILHRDLKPS NLLLNANCDL KVCDFGLAR- ---------- ----------
 111885  ----TDLSKV -------ISN SRDSFLQTGV RTVTLEHIQY FIYQ------ ---------- ---------- ---------- -----ILRG- ---LKAIHSA KVIHRDLKPS NVLLNGNCDL KICDFGLSR- ---------- ----------
 228039  ----SDLHQI -------IHT Q---YLQD-- -----DHLQY FIYQ------ ---------- ---------- ---------- -----ILRG- ---LKYIHSA NVIHRDLKPS NLLINENCDL KICDFGLAR- ---------- ----------
 117728  ----TDLHAV -------IRA -GILEHVH-- -------KQY ITYQ------ ---------- ---------- ---------- -----LLRA- ---IKYMHTG DLLHRDLKPS NVLLNADCHL KVCDFGLCR- ---------- ----------
 267276  ----TDMHRV -------IRT -QDLSDDH-- -------AQY FIYQ------ ---------- ---------- ---------- -----TLRA- ---LKALHSA DVIHRDLKPS NLLLNANCDL KVCDFGLAR- ---------- ----------
 295270  ----TDMHRV -------IRT -QDLSDDH-- -------AQY FIYQ------ ---------- ---------- ---------- -----TLRA- ---LKALHSA DVIHRDLKPS NLLLNANCDL KVCDFGLAR- ---------- ----------
 174214  ----TDLHRL -------LTS -RPLEKQF-- -------IQY FLYQ------ ---------- ---------- ---------- -----ILRG- ---LKYVHSA GVVHRDLKPS NILVNENCDL KICDFGLAR- ---------- ----------
 111562  ----TDLHRL -------LTS -RPLEKQF-- -------IQY FLYQ------ ---------- ---------- ---------- -----ILRG- ---LKYVHSA GVVHRDLKPS NILVNENCDL KICDFGLAR- ---------- ----------
 385026  ----TDLHRL -------LTS -RPLEKQF-- -------IQY FLYQ------ ---------- ---------- ---------- -----ILRG- ---LKYVHSA GVVHRDLKPS NILVNENCDL KICDFGLAR- ---------- ----------
  47937  ----TDLHRL -------LTS -RPLEKQF-- -------IQY FLYQ------ ---------- ---------- ---------- -----ILRG- ---LKYVHSA GVVHRDLKPS NILVNENCDL KICDFGLAR- ---------- ----------
 191658  ----TDLHRL -------LTS -RPLEKQF-- -------IQY FLYQ------ ---------- ---------- ---------- -----ILRG- ---LKYVHSA GVVHRDLKPS NILVNENCDL KICDFGLAR- ---------- ----------
 361397  ----TDLHRL -------LTS -RPLEKQF-- -------IQY FLYQ------ ---------- ---------- ---------- -----ILRG- ---LKYVHSA GVVHRDLKPS NILVNENCDL KICDFGLAR- ---------- ----------
 368356  ----TDLHRL -------LTS -RPLEKQF-- -------IQY FLYQ------ ---------- ---------- ---------- -----ILRG- ---LKYVHSA GVVHRDLKPS NILVNENCDL KICDFGLAR- ---------- ----------
 422287  ----TDLHRL -------LTS -RPLEKQF-- -------IQY FLYQ------ ---------- ---------- ---------- -----ILRG- ---LKYVHSA GVVHRDLKPS NILVNENCDL KICDFGLAR- ---------- ----------
1051981  ----TDLHRL -------LTS -RPLEKQF-- -------IQY FLYQ------ ---------- ---------- ---------- -----IMRG- ---LKYVHSA GVVHRDLKPS NILVNENCDL KICDFGLAR- ---------- ----------
 512786  ----TDLHRL -------LTS -RPLEKQF-- -------IQY FLYQ------ ---------- ---------- ---------- -----IMRG- ---LKYVHSA GVVHRDLKPS NILVNENCDL KICDFGLAR- ---------- ----------
 700253  ----TDLHRL -------LTS -RPLEKQF-- -------IQY FLYQ------ ---------- ---------- ---------- -----IMRG- ---LKYVHSA GVVHRDLKPS NILVNENCDL KICDFGLAR- ---------- ----------
 171641  ----TDLHRL -------LTS -RPLEKQF-- -------IQY FLYQ------ ---------- ---------- ---------- -----IMRG- ---LKYVHSA GVVHRDLKPS NILVNENCDL KICDFGLAR- ---------- ----------
 447383  ----TDLHRL -------LTS -RPLEKQF-- -------IQY FLYQ------ ---------- ---------- ---------- -----IMRG- ---LKYVHSA GVVHRDLKPS NILVNENCDL KICDFGLAR- ---------- ----------
 134451  ----TDLHRL -------LTS -RPLEKQF-- -------IQY FLYQ------ ---------- ---------- ---------- -----ILRG- ---LKYVHSA GVVHRDLKPS NILVNENCDL KICDFGLAR- ---------- ----------
 525556  ----TDLHRL -------LTS -RPLEKQF-- -------IQY FLYQ------ ---------- ---------- ---------- -----ILRG- ---LKYVHSA GVVHRDLKPS NILINENCDL KICDFGLAR- ---------- ----------
1776212  ----TDLHRL -------LTS -RPLEKQF-- -------IQY FLYQ------ ---------- ---------- ---------- -----ILRG- ---LKYVHSA GVVHRDLKPS NILVNENCDL KICDFGLAR- ---------- ----------
 443108  ----TDLHRL -------LTS -RPLEKQF-- -------IQY FLYQ------ ---------- ---------- ---------- -----ILRG- ---LKYVHSA GVVHRDLKPS NILVNENCDL KICDFGLAR- ---------- ----------
 129540  ----TDLHRL -------LTS -RPLEKQF-- -------IQY FLYQ------ ---------- ---------- ---------- -----ILRG- ---LKYVHSA GVVHRDLKPS NILINENCDL KICDFGLAR- ---------- ----------
 341724  ----TDLHRL -------LTS -RPLEKQF-- -------IQY FLYQ------ ---------- ---------- ---------- -----ILRG- ---LKYVHSA GVVHRDLKPS NILINENCDL KICDFGLAR- ---------- ----------
 138417  ----TDLHRL -------LTS -RPLEKQF-- -------IQY FLYQ------ ---------- ---------- ---------- -----ILRG- ---LKYVHSA GVVHRDLKPS NILINENCDL KICDFGLAR- ---------- ----------
 158191  ----TDLHRL -------LTS -RPLEKQF-- -------IQY FLYQ------ ---------- ---------- ---------- -----ILRG- ---LKYVHSA GVVHRDLKPS NILINENCDL KICDFGLAR- ---------- ----------
 376076  ----TDLHRL -------LTS -RPLEKQF-- -------IQY FLYQ------ ---------- ---------- ---------- -----IMRG- ---LKYVHSA GVVHRDLKPS NILVNENCDL KICDFGLAR- ---------- ----------
 368607  ----TDLHRL -------LTS -RPLEKQF-- -------IQY FLYQ------ ---------- ---------- ---------- -----IFRG- ---LKYVHSA GVVHRDLKPS NILVNENCDL KICDFGLAR- ---------- ----------
1312592  ----TDLHRL -------LTS -RPLEKQF-- -------IQY FLYQ------ ---------- ---------- ---------- -----ILRG- ---LKYVHSA GVVHRDLKPS NILVNENCDL KICDFGLAR- ---------- ----------
  46955  ----TDLHRL -------LTS -RPLEKQF-- -------IQY FLYQ------ ---------- ---------- ---------- -----ILRG- ---LKYVHSA GVVHRDLKPS NILINENCDL KICDFGLAR- ---------- ----------
1164008  ----TDLHRL -------LTS -RPLEKQF-- -------IQY FLYQ------ ---------- ---------- ---------- -----ILRG- ---LKYVHSA GVVHRDLKPS NILINENCDL KICDFGLAR- ---------- ----------
 600771  ----TDLHRL -------LTS -RPLEKQF-- -------IQY FLYQ------ ---------- ---------- ---------- -----ILRG- ---LKYVHSA GVVHRDLKPS NILINENCDL KICDFGLAR- ---------- ----------
 161654  ----TDLHRL -------LTS -RPLEKQF-- -------IQY FLYQ------ ---------- ---------- ---------- -----ILRG- ---LKYVHSA GVVHRDLKPS NILVNENCDL RICDFGLAR- ---------- ----------
 149140  ----TDLHRL -------LTS -RPLEKQF-- -------IQY FLYQ------ ---------- ---------- ---------- -----ILRG- ---LKYVHSA GVVHRDLKPS NILVNENCDL RICDFGLAR- ---------- ----------
 474692  ----TDLHRL -------LTS -RPLEKQF-- -------IQY FLYQ------ ---------- ---------- ---------- -----ILRG- ---LKYVHSA GVVHRDLKPS NILVNENCDL KICDFGLAR- ---------- ----------
 134758  ----TDLHRL -------LTS -RPLEKQF-- -------IQY FLYQ------ ---------- ---------- ---------- -----ILRG- ---LKYVHSA GVVHRDLKPS NILVNENCDL KICDFGLAR- ---------- ----------
 177791  ----TDLHRL -------LTS -RPLEKQF-- -------IQY FLYQ------ ---------- ---------- ---------- -----ILRG- ---LKYVHSA GVVHRDLKPS NILVNENCDL KICDFGLAR- ---------- ----------
 132015  ----TDLHRL -------LTS -RPLEKQF-- -------IQY FLYQ------ ---------- ---------- ---------- -----ILRG- ---LKYVHSA GVVHRDLKPS NILVNENCDL KICDFGLAR- ---------- ----------
 917530  ----TDLHRL -------LTS -RPLEKQF-- -------IQY FLYQ------ ---------- ---------- ---------- -----ILRG- ---LKYVHSA GVVHRDLKPS NILVNENCDL KICDFGLAR- ---------- ----------
 428496  ----TDLHRL -------LTS -RPLEKQF-- -------IQY FLYQ------ ---------- ---------- ---------- -----ILRG- ---LKYVHSA GVVHRDLKPS NILINENCDL KICDFGLAR- ---------- ----------
1276586  ----TDLHRL -------ITS -RPLEKQF-- -------IQY FLYQ------ ---------- ---------- ---------- -----ILRG- ---LKYVHSA GVVHRDLKPS NILVNENCDL RICDFGLAR- ---------- ----------
 115601  ----TDLHRL -------LTS -RPLEKQF-- -------IQY FLYQ------ ---------- ---------- ---------- -----ILRG- ---LKYVHSA GVVHRDLKPS NILVNENCDL KICDFGLAR- ---------- ----------
 171809  ----TDLHRL -------LTS -RPLEKQF-- -------IQY FLYQ------ ---------- ---------- ---------- -----ILRG- ---LKYVHSA GVVHRDLKPS NILVNENCDL KICDFGLAR- ---------- ----------
 571775  ----TDLHRL -------LTS -RPLEKQF-- -------IQY FLYQ------ ---------- ---------- ---------- -----ILRG- ---LKYVHSA GVVHRDLKPS NILVNENCDL KICDFGLAR- ---------- ----------
 722302  ----TDLHRL -------LTS -RPLEKQF-- -------IQY FLYQ------ ---------- ---------- ---------- -----ILRG- ---LKYVHSA GVVHRDLKPS NILVNENCDL KICDFGLAR- ---------- ----------
 289694  ----TDLHRL -------LTS -RPLEKQF-- -------IQY FLYQ------ ---------- ---------- ---------- -----ILRG- ---LKYVHSA GVVHRDLKPS NILVNENCDL KICDFGLAR- ---------- ----------
 137978  ----TDLHRL -------LQS -RPLEKQF-- -------IQY FLYQ------ ---------- ---------- ---------- -----ILRG- ---LKYVHSA GVIHRDLKPS NILINENCDL KICDFGLAR- ---------- ----------
  19116  ----TDLHRL -------LTS -RPLEKQF-- -------IQY FLYQ------ ---------- ---------- ---------- -----ILRG- ---LKYVHSA GVVHRDLKPS NILINENCDL KICDFGLAR- ---------- ----------
 129801  ----TDLHRL -------LTS -RPLEKQF-- -------IQY FLYQ------ ---------- ---------- ---------- -----ILRG- ---LKYVHSA GVIHRDLKPS NILINENCDL KICDFGLAR- ---------- ----------
  80382  ----TDLHRL -------LAS -RPLEKQF-- -------IQY FLYQ------ ---------- ---------- ---------- -----ILRG- ---LKYVHSA GVIHRDLKPS NILINENCDL KICDFGLAR- ---------- ----------
 141916  ----TDLHRL -------LAS -RPLEKQF-- -------IQY FLYQ------ ---------- ---------- ---------- -----ILRG- ---LKYVHSA GVIHRDLKPS NILINENCDL KICDFGLAR- ---------- ----------
 187748  ----TDLHRL -------LTS -RPLEKQF-- -------IQY FTYQ------ ---------- ---------- ---------- -----ILRG- ---LKYVHSA GVIHRDLKPS NILINENCDL KICDFGLAR- ---------- ----------
 168984  ----TDLHRL -------LTS -RPLEKQF-- -------IQY FLYQ------ ---------- ---------- ---------- -----IIRG- ---LKYVHSA GVIHRDLKPS NILINENCDL KICDFGLAR- ---------- ----------
  71808  ----TDLHRL -------RQS -RPLEKQF-- -------IQY FLYQ------ ---------- ---------- ---------- -----ILRG- ---LKYVHSA GVIHRDLKPS NIVINENCDL KICDFGLAR- ---------- ----------
    432  ----TDLHRL -------LTS -RPLEREF-- -------ILY FLYQ------ ---------- ---------- ---------- -----ILRG- ---LKYVHSA GVVHRDLKPS NILINENCDL KICDFGLAR- ---------- ----------
  59395  ----TDLHRL -------LTT -RPLEKQF-- -------IQY FLYQ------ ---------- ---------- ---------- -----ILRG- ---LKYIHSA GVVHRDLKPS NILVNENCDL KICDFGLAR- ---------- ----------
   2865  ----TDLHRL -------LTS -RPLEKQF-- -------IQY FLYQ------ ---------- ---------- ---------- -----ILRG- ---LKYVHSA GVVHRDLKPS NILVNENCDL KICDFGLAR- ---------- ----------
 189766  ----TDLHRL -------LTS -RPLEKQF-- -------IQY FLYQ------ ---------- ---------- ---------- -----ILRG- ---LKYVHSA GVVHRDLKPS NILVNENCDL KICDFGLAR- ---------- ----------
  88183  ----TDLHRL -------LTS -RPLEKQF-- -------IQY FLYQ------ ---------- ---------- ---------- -----ILRG- ---LKYVHSA GVVHRDLKPS NILVNENCDL KICDFGLAR- ---------- ----------
 216492  ----TDLHRL -------LTS -RPLEKQF-- -------IQY FLYQ------ ---------- ---------- ---------- -----IIRG- ---LKYVHSA GVVHRDLKPS NILVNENCDL KICDFGLAR- ---------- ----------
 188879  ----TDLHRL -------LTS -RPLEKQF-- -------IQY FLYQ------ ---------- ---------- ---------- -----IIRG- ---LKYVHSA GVVHRDLKPS NILVNENCDL KICDFGLAR- ---------- ----------
 182850  ----TDLHRL -------LTS -RPLEKQF-- -------IQY FLYQ------ ---------- ---------- ---------- -----IIRG- ---LKYVHSA GVVHRDLKPS NILVNENCDL KICDFGLAR- ---------- ----------
 174697  ----TDLHRL -------LTS -RPLEKQF-- -------IQY FLYQ------ ---------- ---------- ---------- -----IIRG- ---LKYVHSA GVVHRDLKPS NILVNENCDL KICDFGLAR- ---------- ----------
```

```
 934899  ----TDLHRL -------LTS -RPLEKQF-- -------IQY FLYQ------ ---------- ---------- ---------- -----ILRG- ---LKYVHSA GVVHRDLKPS NILVNENCDL KICDFGLAR- ---------- ----------
 362397  ----TDLHRL -------LTS -RPLEKQF-- -------IQY FLYQ------ ---------- ---------- ---------- -----ILRG- ---LKYVHSA GVVHRDLKPS NILVNENCDL KICDFGLAR- ---------- ----------
 416973  ----TDLHRL -------LTS -RPLEKQF-- -------IQY FLYQ------ ---------- ---------- ---------- -----ILRG- ---LKYVHSA GVVHRDLKPS NILVNENCDL KRCDFGLAR- ---------- ----------
 551386  ----TDLHRL -------LTS -RPLEKQF-- -------IQY FLYQ------ ---------- ---------- ---------- -----ILRG- ---LKYVHSA GVVHRDLKPS NILVNENCDL KICDFGLAR- ---------- ----------
  15239  ----TDLHRL -------LTS -RPLEKQF-- -------IQY FLYQ------ ---------- ---------- ---------- -----I---- ---------- ------LKPS NILVNENCDL KICDFGLAR- ---------- ----------
 497504  ----TDLHRL -------LTS -RPLEKQF-- -------IQY FLYQ------ ---------- ---------- ---------- -----ILRG- ---LKYVHSA GVVHRDLKPS NILVNENCDL KICDFGLAR- ---------- ----------
1117682  ----TDLHRL -------LTS -RRLEKQF-- -------IQY FLYQ------ ---------- ---------- ---------- -----ILRA- ---LKYVHSA GVVHRDLKPS NILINENCDL KICDFGLAR- ---------- ----------
 252496  ----TDLHRL -------LTS -RPLEKQF-- -------IQY FLYQ------ ---------- ---------- ---------- -----ILRG- ---LKYVHSA GVVHRDLKPS NILVNENCDL KICDFGLAR- ---------- ----------
 148867  ----TDLHRL -------LIS -RPLDTQF-- -------IQY FLYQ------ ---------- ---------- ---------- -----ILRG- ---LKYVHSA GVVHRDLKPS NILVNENCDL KICDFGLAR- ---------- ----------
 423027  ----TDLNRL -------LTS -KPLDGKF-- -------VQY FTYQ------ ---------- ---------- ---------- -----LLRG- ---LKYIHSA GVIHRDLKPS NILINENCDL KICDFGLAR- ---------- ----------
1104996  ----TDLNRL -------LTS -KPLDGKF-- -------VQY FTYQ------ ---------- ---------- ---------- -----LLRG- ---LKYIHSA GVIHRDLKPS NILINENCDL KICDFGLAR- ---------- ----------
  29266  ----TDLHRL -------LTS -RPLEKQF-- -------IQY FLYQ------ ---------- ---------- ---------- -----IMRG- ---LKYVHSA GVVHRDLKPS NILVNENCDL KICDFGLAR- ---------- ----------
 171653  ----TDLHRL -------LTS -RPLEKQF-- -------IQY FLYQ------ ---------- ---------- ---------- -----IMRG- ---LKYVHSA GVVHRDLKPS NILVNENCDL KICDFGLAR- ---------- ----------
 117063  ----TDLHRL -------LTS -RPLEKQF-- -------IQY FLYQ------ ---------- ---------- ---------- -----ILRG- ---LKYVHSA GVVHRDLKPS NILVNENCDL KICDFGLAR- ---------- ----------
 158104  ----TDLHRL -------LTS -RPLEKQF-- -------IQY FLYQ------ ---------- ---------- ---------- -----ILRG- ---LKYVHSA GVVHRDLKPS NILINENCDL KICDFGLAR- ---------- ----------
 160940  ----TDLHRL -------LTS -RPLEKQF-- -------IQY FLYQ------ ---------- ---------- ---------- -----ILRG- ---LKYVHSA GVVHRDLKPS NILVNENCDL KICDFGLAR- ---------- ----------
   2813  ----TDLHRL -------LTS -RPLEKQF-- -------IQY FLYQ------ ---------- ---------- ---------- -----ILRG- ---LKYVHSA GVVHRDLKPS NILVNENCDL KICDFGLAR- ---------- ----------
 129541  ----TDLHRL -------LTS -RPLEKQF-- -------IQY FLYQ------ ---------- ---------- ---------- -----ILRG- ---LKYVHSA GVVHRDLKPS NILINENCDL KICDFGLAR- ---------- ----------
 257371  ----TDLHRL -------LTS -RPLEKQF-- -------IQY FLYQ------ ---------- ---------- ---------- -----IFRG- ---LKYVHSA GVVHRDLKPS NILVNENCDL KICDFGLAR- ---------- ----------
 389086  ----TDLYQV -------IRT -TTLDGQF-- -------VQF FTYQ------ ---------- ---------- ---------- -----ILRG- ---LRYIHSA GVIHRDLKPQ NLLVNNNCDL KICDFGLAR- ---------- ----------
  73401  ----TDLNRL -------MRS -KPLEPQF-- -------VQY FTYQ------ ---------- ---------- ---------- -----ILRG- ---LKYIHSA GVIHRDLKPS NILVNENCDL KICDFGLAR- ---------- ----------
1139913  ----TDLNRL -------MRS -KPLEPQF-- -------VQY FTYQ------ ---------- ---------- ---------- -----ILRG- ---LKYIHSA GVIHRDLKPS NILVNENCDL KICDFGLAR- ---------- ----------
1168371                    MRS -KPLEPQF-- -------VQY FTYQ------ ---------- ---------- ---------- -----ILRG- ---LKYIHSA GVIHRDLKPS NILVNENCDL KICDFGLAR- ---------- ----------
 698519  ----TDLNRL -------MRS -KPLEPQF-- -------VQY FTYQ------ ---------- ---------- ---------- -----ILRG- ---LKYIHSA GVIHRDLKPS NILVNENCDL KICDFGLAR- ---------- ----------
 332332  ---------L -------LTS -RPLEKQF-- -------IQY FLYQ------ ---------- ---------- ---------- -----ILRG- ---LKYVHSA GVVHRDLKPS NILINENCDL KICDFGLAR- ---------- ----------
 243292  ----TDLHRL -------LTS -RPLEKQF-- -------IQY FLYQ------ ---------- ---------- ---------- -----ILRG- ---LKYVHSA GVVHRDLKPS NILVNENCDL KICDFGLAR- ---------- ----------
 243775  ----TDLHRL -------LTS -RPLEKQF-- -------IQY FLYQ------ ---------- ---------- ---------- -----ILRG- ---LKYVHSA GVVHRDLKPS NILVNENCDL KICDFGLAR- ---------- ----------
  24163  ----TDLHRL -------LTS -RPLEKQF-- -------IQY FLYQ------ ---------- ---------- ---------- -----ILRG- ---LKYVHSA GVVHRDLKPS NILVNENCDL KICDFGLAR- ---------- ----------
1431451  ----TDLHRL -------LTS -RPLEKQF-- -------IQY FLYQ------ ---------- ---------- ---------- -----ILRG- ---LKYVHSA GVVHRDLKPS NILVNENCDL KICDFGLAR- ---------- ----------
  99398                                                                                        MQRG- ---LKYVHSA GVVHRDLKPS NILINENCDL KICDFGLAR- ---------- ----------
 171665                                                                                         MRG- ---LKYVHSA GVVHRDLKPS NILVNENCDL KICDFGLAR- ---------- ----------
  15876  ----TDLYQV -------IRT -TTLDGQF-- -------VQF FTYQ------ ---------- ---------- ---------- -----ILRG- ---LRYIHSA GVIHRDLKPQ NLLVNNNCDL KICDFGLAR- ---------- ----------
  43014  ----TDMHRV -------IRT -QELSDDH-- -------CQY FIYQ------ ---------- ---------- ---------- -----TLRG- ---LKALHSA QVLHRDLKPS NLLLNANCDL KICDFGLAR- ---------- ----------
  43498  ----TDMHRV -------IRT -QELSDDH-- -------CQY FIYQ------ ---------- ---------- ---------- -----TLRG- ---LKALHSA QVLHRDLKPS NLLLNANCDL KICDFGLAR- ---------- ----------
1148336                                                                                                   MHSA NVLHRDLKPS NLLLNANCDL KVCDFGLAR- ---------- ----------
 220391                                                                     M CEREHYIHPR YHQAAHARV- ---LHRDLLA DIVHRDLKPA NLLLNANCDL KVCDFGLAR- ---------- ----------
 774567                                                                          MITVRQ YFIYQTLRA- ---LKTMHSA DIVHRDLKPA NLLLNANCDL KVCDFGLAR- ---------- ----------
 138915                                                                              MQ YFIYQTLRA- ---LKTMHSA DIVHRDLKPA NLLLNANCDL KVCDFGLAR- ---------- ----------
 757677
 313819
 290180
  12232
 865310
 312015
 500478
 500460
 243933
  64065
   2938
 196733
 145976
 501440
  71512
 543038
  99402
1431403
 342880
 417966
Consensus  tD$hr.        irs .q.L.d.h          .Qy F.YQ                                                .lrg     $Ky.HSA .!.HRDLKP. NlLvNa#C#L K!CDFGLaR. ..

         901                                                                                                                                                   1050
   6423  ---------- ---------- ---------- ---VDPE--- ----E-NAGY MTEYVATRWY RAPEIMLSFQ GY-------- ---T------ --------KA IDVWSVGCIL AELLGS--KP FFKGRDY--- ---------- -------VDQ
 160078  ---------- ---------- ---------- ---VDPE--- ----E-NAGY MTEYVATRWY RAPEIMLSFQ SY-------- ---T------ --------KA IDVWSVGCIL AELLGS--KP FFKGRDY--- ---------- -------VDQ
 116650  ---------- ---------- ---------- ---IDPE--- ----E-NAGY MTEYVATRWY RAPEIMLSFQ SY-------- ---T------ --------KA IDVWSVGCIL AELLGS--KP FFKGRDY--- ---------- -------VDQ
 377992  ---------- ---------- ---------- ---VDPD--- ----E-NAGY MTEYVATRWY RAPEIMLSFQ SY-------- ---T------ --------KA IDVWSVGCIL AELLGS--KP FFKGRDY--- ---------- -------VDQ
 289793  ---------- ---------- ---------- ---MDPE--- ----E-NAGY MTEYVATRWY RAPEIMLSFQ SY-------- ---T------ --------KA IDVWSVGCIL AELLGG--KP FFKGRDY--- ---------- -------VDQ
 670269  ---------- ---------- ---------- ---MDPE--- ----E-NAGY MTEYVATRWY RAPEIMLSFQ SY-------- ---T------ --------KA IDVWSVGCIL AELLGG--KP FFKGRDY--- ---------- -------VDQ
  32808  ---------- ---------- ---------- ---MDPE--- ----E-NAGY MTEYVATRWY RAPEIMLSFQ SY-------- ---T------ --------KA IDVWSVGCIL AELLGG--KP FFKGRDY--- ---------- -------VDQ
 156295  ---------- ---------- ---------- ---MDPE--- ----E-NAGY MTEYVATRWY RAPEIMLSFQ SY-------- ---T------ --------KA IDVWSVGCIL AELLGG--KP FFKGRDY--- ---------- -------VDQ
  22314  ---------- ---------- ---------- ---MDPE--- ----E-NAGY MTEYVATRWY RAPEIMLSFQ SY-------- ---T------ --------KA ------RCIL AELLGG--KP FFKGRDY--- ---------- -------VDQ
 417978  ---------- ---------- ---------- ---MDPE--- ----E-NAGY MTEYVATRWY RAPEIMLSFQ SY-------- ---T------ --------KA IDVWSVGCIL AELLGG--KP FFKGRDY--- ---------- -------VDQ
 414783  ---------- ---------- ---------- ---IDPE--- ----E-NAGY MTEYVATRWY RAPEIMLSFQ SY-------- ---T------ --------KA IDVWSVGCIL AELLGG--KP FFKGRDY--- ---------- -------VDQ
1074946  ---------- ---------- ---------- ---VDPE--- ----E-NAGY MTEYVATRWY RAPEIMLSFQ SY-------- ---T------ --------KA IDVWSVGCIL AELLGG--RP FFKGRDY--- ---------- -------VDQ
 865350  ---------- ---------- ---------- ---VDPE--- ----E-NAGY MTEYVATRWY RAPEIMLSFQ SY-------- ---T------ --------KA IDVWSVGCIL AELLGG--RP FFKGRDY--- ---------- -------VDQ
1656563  ---------- ---------- ---------- ---VDPE--- ----E-NAGY MTEYVATRWY RAPEIMLSFQ SY-------- ---T------ --------KA IDVWSVGCIL AELLGG--RP FFKGRDY--- ---------- -------VDQ
 333276  ---------- ---------- ---------- ---VDPE--- ----E-NAGY MTEYVATRWY RAPEIMLSFQ SY-------- ---T------ --------KA IDVWSVGCIL AELLGG--RP FFKGRDY--- ---------- -------VDQ
 422586  ---------- ---------- ---------- ---VDPE--- ----E-NAGY MTEYVATRWY RAPEIMLSFQ SY-------- ---T------ --------KA IDVWSVGCIL AELLGG--RP FFKGRDY--- ---------- -------VDQ
 342887  ---------- ---------- ---------- ---VDPE--- ----E-NAGY MTEYVATRWY RAPEIMLSFQ SY-------- ---T------ --------KA IDVWSVGCIL AELLGG--RP FFKGRDY--- ---------- -------VDQ
 386771  ---------- ---------- ---------- ---VDPE--- ----E-NAGY MTEYVATRWY RAPEIMLSFQ SY-------- ---T------ --------KA IDVWSVGCIL AELLGG--RP FFKGRDY--- ---------- -------VDQ
  14976  ---------- ---------- ---------- ---VDPE--- ----E-NAGY MTEYVATRWY RAPEIMLSFQ SY-------- ---T------ --------KA IDVWSVGCIL AELLGG--RP FFKGRDY--- ---------- -------VDQ
 130981  ---------- ---------- ---------- ---VDPE--- ----E-NAGY MTEYVATRWY RAPEIMLSFQ SY-------- ---T------ --------KA IDVWSVGCIL AELLGG--RP FFKGRDY--- ---------- -------VDQ
 435286  ---------- ---------- ---------- ---VDPE--- ----E-NAGY MTEYVATRWY RAPEIMLSFQ SY-------- ---T------ --------KA IDVWSVGCIL AELLGG--RP FFKGRDY--- ---------- -------VDQ
1457372  ---------- ---------- ---------- ---VDPE--- ----E-NAGY MTEYVATRWY RAPEIMLSFQ SY-------- ---T------ --------KA IDVWSVGCIL AELLGG--RP FFKGRDY--- ---------- -------VDQ
 249807  ---------- ---------- ---------- ---IDPE--- ----E-NAGY MTEYVATRWY RAPEIMLSFQ SY-------- ---T------ --------KA IDVWSVGCIL AELLGG--RP FFKGRDY--- ---------- -------VDQ
1142566  ---------- ---------- ---------- ---IDPE--- ----E-NAGY MTEYVATRWY RAPEIMLSFQ SY-------- ---T------ --------KA IDVWSVGCIL AELLGG--RP FFKGRDY--- ---------- -------VDQ
1008492  ---------- ---------- ---------- ---IDPE--- ----E-NAGY MTEYVATRWY RAPEIMLSFQ SY-------- ---T------ --------KA IDVWSVGCIL AELLGG--RP FFKGRDY--- ---------- -------VDQ
 152510  ---------- ---------- ---------- ---VDPD--- ----E-NAGY MTEYVATRWY RAPEIMLSFP SY-------- ---T------ --------KA IDVWSVGCIL AELLGG--RP FFKGRDY--- ---------- -------VDQ
 336937  ---------- ---------- ---------- ---VDPD--- ----E-NAGY MTEYVATRWY RAPEIMLSFP SY-------- ---T------ --------KA IDVWSVGCIL AELLGG--RP FFKGRDY--- ---------- -------VDQ
1649893  ---------- ---------- ---------- ---VDPE--- ----E-NAGY MTEYVATRWY RAPEIMLSFQ SY-------- ---T------ --------KA IDVWSVGCIL AELLGG--RP FFKGRDY--- ---------- -------VDQ
 371270  ---------- ---------- ---------- ---IDPE--- ----E-NAGY LTEYVATRWY RAPEIMLSFQ SY-------- ---T------ --------KA IDVWSVGCIL AELLGG--RP FFKGRDY--- ---------- -------VDQ
  82103  ---------- ---------- ---------- ---MDPE--- ----E-NAGY MTEYVATRWY RAPEIMLSFQ SY-------- ---T------ --------KA IDVWSVGCIL AELLGG--KP FFKGRDY--- ---------- -------VDQ
  42859  ---------- ---------- ---------- ---NEPD--- ----NVAAGF QTEYVATRWY RAPEIMLSFA EY-------- ---T------ --------KA IDVWSVGCIL AELLGG--KP FLKGKDY--- ---------- -------VDQ
 212507  ---------- ---------- ---------- ---VDPE--- ----E-NAGY MTEYVATRWY RAPEIMLSFQ SY-------- ---T------ --------KA IDVWSVGCIL AELLGG--RP FFKGRDY--- ---------- -------VDQ
 212502  ---------- ---------- ---------- ---VDPE--- ----E-NAGY MTEYVATRWY RAPEIMLSFQ SY-------- ---T------ --------KA IDVWSVGCIL AELLGG--RP FFKGRDY--- ---------- -------VDQ
 461664  ---------- ---------- ---------- ---VDPE--- ----E-NAGY MTEYVATRWY RAPEIMLSFQ SY-------- ---T------ --------KA IDVWSVGCIL AELLGG--RP FFKGRDY--- ---------- -------VDQ
  39613  ---------- ---------- ---------- ---EDPE--- ----R-NAGF MTEYVATRWY RAPEIMSSVQ SY-------- ---S------ --------KA IDVWSVGCIL AESLGG--RP SFRGRDY--- ---------- -------VDQ
  91135  ---------- ---------- ---------- ---EDPS--- ----K-NRGF LTEYVATRWY RAPEIMLSFQ SY-------- ---S------ --------KA IDLWSVGCIL AELLGG--KP IFKGKDY--- ---------- -------VDQ
 103403  ---------- ---------- ---------- ---SDPA--- ----T-NNGF LTEYVATRWY RAPEIMLSFQ SY-------- ---T------ --------KA IDIWSVGCIL AELLGG--KP IFRGKDY--- ---------- -------VDQ
 112360  ---------- ---------- ---------- ---SIPE--- ----K-NTGF LTEYVATRWY RAPEIMLSFQ SY-------- ---T------ --------KA IDVWSIGCIL AELLGG--KP IFKGKDY--- ---------- -------VDQ
   4266  ---------- ---------- ---------- ---SDPT--- ----K-NNGF MTEYVATRWY RAPEIILSFQ GY-------- ---T------ --------KA IDMWSAGCIL AELLGR--RT IFKGKDY--- ---------- -------VDQ
 168486  ---------- ---------- ---------- ---ENIE--- ----Q-NSGF MTEYVATRWY RAPEIMLSFT NY-------- ---T------ --------KA IDIWSVGCIL AELLGG--KP LFRGKDY--- ---------- -------VDQ
  35227  ---------- ---------- ---------- ---NDPE--- ----K-NQGF LTEYVATRWY RAPEIILSFQ GY-------- ---T------ --------KA IDMWSVGCIL AELLGG--ST IFKGKDY--- ---------- -------VDQ
  67581  ---------- ---------- ---------- ---NDPE--- ----K-NQGF LTEYVATRWY RAPEIILSFQ GY-------- ---T------ --------KA IDMWSVGCIL AELLGG--ST IFKGKDY--- ---------- -------VDQ
  59367  ---------- ---------- ---------- ---ENPV--- ----K-NNQF LTEYVATRWY RAPEIMLSYQ GY-------- ---T------ --------KS IDVWSTGCIL AELLGG--KP IFKGKDY--- ---------- -------VDQ
 336175  ---------- ---------- ---------- ---SSPE--- ----GNV-GF MTEYVATRWY RAPEIMLSFQ QY-------- ---T------ --------KA IDMWSVGCIF AEMLGG--RP LFKGRDY--- ---------- -------VDQ
 177826  ---------- ---------- ---------- ---ENPE--- ----SNAAGF MTEYVATRWY RAPEIMLSFQ NY-------- ---T------ --------KA IDMWSVGCIF AEMLGG--KP LFKGRDY--- ---------- -------VDQ
 212434  ---------- ---------- ---------- ---ESPE--- ----SNAAGF MTEYVATRWY RAPEIMLSFQ NY-------- ---T------ --------KA IDMWSVGCIF AEMLGG--KP LFKGRDY--- ---------- -------VDQ
 164796  ---------- ---------- ---------- ---ESPE--- ----SNAAGF MTEYVATRWY RAPEIMLSFQ NY-------- ---T------ --------KA IDMWSVGCIF AEMLGG--KP LFKGRDY--- ---------- -------VDQ
 100890  ---------- ---------- ---------- ---ETPE--- ----GNI-GF MTEYVATRWY RAPEIMLSFL SY-------- ---T------ --------KA IDIWSVGCIF AELLGG--KP LFKGRDY--- ---------- -------VDQ
 288633  ---------- ---------- ---------- ---ESKG--- ----EGDAGF MTEYVATRWY RAPEIMLSFR NY-------- ---T------ --------KA IDMWSLGCIL AELLGA--KP LFKGTNN--- ---------- -------VDQ
  69432  ---------- ---------- ---------- ---PGGGTS- -RA-AGNQGF MTEYVATRWY RAPEIMLSFA NY-------- ---T------ --------TA IDVWSIGCIL AELLGG--KP IYKGRDY--- ---------- -------VDQ
  60279  ---------- ---------- ---------- ---PGGGTS- -RV-AGNSGF MTEYVATRWY RAPEIMLSFA NY-------- ---S------ --------TA IDVWSIGCIL AELLGG--KP IYKGRDY--- ---------- -------VDQ
 166223  ---------- ---------- ---------- ---PGGGAS- -AS-AGNQGF MTEYVATRWY RAPEIMLSFA NY-------- ---T------ --------TA IDVWSIGCIL AELLGG--KP IFKGRDY--- ---------- -------VDQ
1111368  ---------- ---------- ---------- ---PGGGAS- -KS-AGNQGF MTEYVATRWY RAPEIMLSFA NY-------- ---T------ --------TA IDVWSVGCIL AELLGG--KP IYKGRDY--- ---------- -------VDQ
7180514  ---------- ---------- ---------- ---PGGGTS- -RA-AGNQGF MTEYVATRWY RAPEIMLSFA NY-------- ---S------ --------TA IDVWSVGCIL AELLGG--KP IYKGRDY--- ---------- -------VDQ
 671172  ---------- ---------- ---------- ---PGGGTS- -KA-AGNQGF MTEYVATRWY RAPEIMLSFA NY-------- ---S------ --------TA IDVWSVGCIL AELLGG--KP IYKGRDY--- ---------- -------VDQ
  69595  ---------- ---------- ---------- ---PGGGTS- -KA-AGNQGF MTEYVATRWY RAPEIMLSFA NY-------- ---S------ --------TA IDVWSVGCIL AELLGG--KP IYKGRDY--- ---------- -------VDQ
 188428  ---------- ---------- ---------- ---PGGGTS- -KA-AGNQGF MTEYVATRWY RAPEIMLSFA NY-------- ---S------ --------TA IDVWSVGCIL AELLGG--KP IYKGRDY--- ---------- -------VDQ
 799680  ---------- ---------- ---------- ---PGGGAS- -KS-AGNQGF MTEYVATRWY RAPEIMLSFA NY-------- ---T------ --------TA IDVWSVGCIL AELLGG--KP IYKGRDY--- ---------- -------VDQ
 178052  ---------- ---------- ---------- ---PGGGAS- -KS-AGNQGF MTEYVATRWY RAPEIMLSFA NY-------- ---T------ --------TA IDVWSVGCIL AELLGG--KP IFKGRDY--- ---------- -------VDQ
 129301  ---------- ---------- ---------- ---PGGGAS- -RA-AGNQGF MTEYVATRWY RAPEIMLSFA NY-------- ---T------ --------TA IDVWSIGCIL AELLGG--KP IFKGRDY--- ---------- -------VDQ
 208817  ---------- ---------- ---------- ---PGGGAS- -RA-AGNQGF MTEYVATRWY RAPEIMLSFA NY-------- ---T------ --------TA IDVWSVGCIL AELLGG--KP IYKGRDY--- ---------- -------VDQ
 424444  ---------- ---------- ---------- ---PGGGAS- -KA-AGNQGF MTEYVATRWY RAPEIMLSFA NY-------- ---T------ --------SS IDVWSVGCIL AELLGG--KP IFKGRDY--- ---------- -------VDQ
 830405  ---------- ---------- ---------- ---PGSGTS- -RA-AGNQGF MTEYVATRWY RAPEIMLSFA NY-------- ---T------ --------TA IDVWSVGCIL AELLGG--KP IFKGRDY--- ---------- -------VDQ
 892596  ---------- ---------- ---------- ---PGGGAS- -KS-AGNQGF MTEYVATRWY RAPEIMLSFA NY-------- ---T------ --------TA IDVWSIGCIL AELLGG--KP IFKGRDY--- ---------- -------VDQ
 186189  ---------- ---------- ---------- ---PGGGSNQ TRA-AANQGF MTEYVATRWY RAPEIMLSFA NY-------- ---G------ --------PA IDVWSVGCIL AELLAG--KP IFKGRDY--- ---------- -------VDQ
 164780  ---------- ---------- ---------- ---PGGGANA VRG-APNQGF MTEYVATRWY RAPEIMLSFA NY-------- ---T------ --------TA IDVWSVGCIL AELLGG--KP IFKGRDY--- ---------- -------VDQ
 167312  ---------- ---------- ---------- ---PGGGNSG TRG-AANQGF MTEYVATRWY RAPEIMLSFA NY-------- ---G------ --------PA IDVWSVGCIL AELLAG--KP IFKGRDY--- ---------- -------VDQ
 119588  ---------- ---------- ---------- ---PGGGNNN ARG-AANQGF MTEYVATRWY RAPEIMLSFA NY-------- ---G------ --------PA IDVWSVGCIL AELLAG--KP IFKGRDY--- ---------- -------VDQ
 423592  ---------- ---------- ---------- ---------- --Q-AQRQGF MTEYVATRWY RAPEIMLSFA NY-------- ---A------ --------SA IDVWSVGCIL AELLGG--KP IFKGRDY--- ---------- -------VDQ
 474667  ---------- ---------- ---------- ---PGSGTS- -RA-AGNQGF MTEYVATRWY RAPEIMLSFA NY-------- ---S------ --------TA IDVWSVGCIL AELLGG--RP IFKGRDY--- ---------- -------VDQ
 459613  ---------- ---------- ---------- ---PGSGTS- -RA-AGNQGF MTEYVATRWY RAPEIMLSFA NY-------- ---S------ --------TA IDVWSVGCIL AELLGG--RP IFKGRDY--- ---------- -------VDQ
 156206  ---------- ---------- ---------- ---PGGGGTS -KA-AGNQGF MTEYVATRWY RAPEIMLSFA NY-------- ---S------ --------TA IDVWSVGCIL AELLGG--KP IYRGKDY--- ---------- -------VDQ
```

```
 791811  ---------- ---------- ---------- ---PSGKNAA -KS-AANQGF MTEYVATRWY RAPEIMLSFA NY-------- ---G------ --------PA IDVWSVGCIL AELLAG--KP IFKGRDY--- ---------- -------VDQ
 146905  ---------- ---------- ---------- ---PDGTAGD PKA-AGVQGF MTEYVATRWY RAPEIMLSFA NY-------- ---T------ --------TA IDVWSIGCIL AELLGG--KP IFKGRDY--- ---------- -------VDQ
 715329  ---------- ---------- ---------- ---PGSGAPA SKG-AANQGF MTEYVATRWY RAPEIMLSFA NY-------- ---G------ --------PA IDVWSVGCIL AELLAG--KP IFKGRDY--- ---------- -------VDQ
 511921  ---------- ---------- ---------- ---PGPGAQQ TQSNKGQQGF MTEYVATRWY RAPEIMLSFA NY-------- ---G------ --------PA IDIWSVGCIF AELLGG--KP IFKGRDY--- ---------- -------VDQ
 737951  ---------- ---------- ---------- ---PAGSGQG TAKNSANQGF MTEYVATRWY RAPEIMLSFA NY-------- ---G------ --------PA IDVWSVGCIL AELLAG--KP IFKGRDY--- ---------- -------VDQ
 545584  ---------- ---------- ---------- ---PGPGTAQ QTSNKGQQGF MTEYVATRWY RAPEIMLSFA NY-------- ---G------ --------PA IDIWSVGCIF AELLGG--KP IFKGRDY--- ---------- -------VDQ
 102765  ---------- ---------- ---------- ---PGPGT-Q QHSNKGQQGF MTEYVATRWY RAPEIMLSFA NY-------- ---G------ --------PA IDIWSVGCIF AELLGG--KP IFKGRDY--- ---------- -------VDQ
 203555  ---------- ---------- ---------- ---PGGGAS- -----GNHGF MTEYVATRWY RAPEIMLSFA NY-------- ---S------ --------TA IDIWSVGCIL AELLGG--QP IFKGKDY--- ---------- -------IDQ
 302829  ---------- ---------- ---------- ---PGGGTS- --KAAGNQGF MTEYVATRWY RAPEIMLSFA NY-------- ---S------ --------TA IDVWSVGCIL AELLGG--KP IYKGRDY--- ---------- -------VDQ
 701651  ---------- ---------- ---------- ---PSGKNA- -AKSAANQGF MTEYVATRWY RAPEIMLSFA NY-----SQ- ---G------ --------PA IDVWSVGCIL AELLAG--KP IFKGRDY--- ---------- -------VDQ
 972433  ---------- ---------- ---------- ---PGG-GT- -SKSAGNQGF MTEYVATRWY RAPEIMLSFA NY-------- ---T------ --------TA IDVWSVGCIL AELLGG--KP IYKGRDY--- ---------- -------VDQ
 931864  ---------- ---------- ---------- ---PGG-GT- -SKSAGNQGF MTEYVATRWY RAPEIMLSFA NY-----VR- ---T------ --------TA IDVWSVGCIL AELLGG--KP IYKGRDY--- ---------- -------VDQ
 813292  ---------- ---------- ---------- ---PGG-GA- -SKSAGNQGF MTEYVATRWY RAPEIMLSFA NY-------- ---T------ --------TA IDVWSVGCIL AELLGG--KP IFKGRES--- ---------- -------CTI
  78187  ---------- ---------- ---------- ---PGG-GT- -SKAAGNQGF MTEYVATRWY RAPEIMLSFA NY-------- ---S------ --------TA IDVWSVGCIL AELLGG--KP IYKGRDY--- ---------- -------VDQ
 406308  ---------- ---------- ---------- ---------- -IDPEENAGY MTEYVATRWY RAPEIMLSFQ SY-----TKA MILT------ --------PI VDVWSVGCIL AELLGG--RP FFKGRDY--- ---------- -------VDQ
 118382  ---------- ---------- ---------- ---PGGGTS- --KAAGNQGF MTEYVATRWY RAPEIMLSFA NY-----VST ---S------ --------TA IDVWSVGCIL AELLGG--KP IYKGRDY--- ---------- -------VDQ
 100328  ---------- ---------- ---------- ---PGGGSNQ TRA-AANQGF MTEYVATRWY RAPEIMLSFA NY-------- ---G------ --------PA IDVWSVGCIL AELLAG--KP IFKGRDY--- ---------- -------VDQ
1411022  ---------- ---------- ---------- ---SSNGS-- --------GF MTEYVATRWY RAPEIMLSYA NY-------- ---T------ --------TS IDVWSIGCIL AELLGG--KP IFKGRDY--- ---------- -------VDQ
 741167  ---------- ---------- ---------- ---SSNGA-- --------GF MTEYVATRWY RAPEIMLSYA NY-------- ---T------ --------TS IDVWSIGCIL AELLGG--KP IFKGRDY--- ---------- -------VDQ
1338511  ---------- ---------- ---------- ---MDKGA-- ------ENSL MTEYVATRWY RAPEVMLSFG NY-------- ---S------ --------TA IDIWSVGCIL AELHGR--KP IFKGRDY--- ---------- -------VDQ
 756718  ---------- ---------- ---------- ---SDQGA-- ------ENSL MTEYVATRWY RAPEVMLSFG NY-------- ---S------ --------TA IDIWSVGCIL AELHGR--KP IFKGRDY--- ---------- -------VDQ
 302653  ---------- ---------- ---------- ---EGGSAE- ----TGQAGF MTEYVATRWY RAPEIMLSFA NY-------- ---N------ --------SS IDMWSVGCIL AELLGG--RP IFKGRDY--- ---------- -------VDQ
1311110  ---------- ---------- ---------- ---TDPELAQ -AANA---GF MTEYVATRWY RAPEIMLSFQ NY-------- ---T------ --------TA IDLWSVGCIL AELFGG--RP IYKGRDY--- ---------- -------VDQ
 722894  ---------- ---------- ---------- ---TDPTLAQ -ACNQ---GF MTEYVATRWY RAPEIMLSFQ NY-------- ---T------ --------TA IDMWSVGCIL AELLGG--RP IFKGRDY--- ---------- -------VDQ
 288507  ---------- ---------- ---------- ---TDAELAA -KGGAPVGGF MTEYVATRWY RAPEIMLSFQ NY-------- ---T------ --------TA IDIWSVGCIL AELLGG--KP IFKGRDY--- ---------- -------VDQ
 757539  ---------- ---------- ---------- ---PGGGTS- --RVAGNSGF MTEYVATRWY RAPEIMLSFA NY-------- ---S------ --------TA IDVWSIGCIL AELLGG--KP IYKGRDY--- ---------- -------VDQ
 184184  ---------- ---------- ---------- ---PGGGAS- --KSAGNQGF MTEYVATRWY RAPEIMLSFA NY-------- ---T------ --------TA IDVWSVGCIL AELLGG--KP IFKGRDY--- ---------- -------VDQ
 917307  ---------- ---------- ---------- ---PGG---G TSKSAGNQGF MTEYVATRWY RAPEIMLSFA NY-------- ---T------ --------TA IDVWSVGCIL AELLGG--KP IYKGRDY--- ---------- -------VDQ
6355211  ---------- ---------- ---------- ---PGG---G TSRAAGNQGF MTEYVATRWY RAPEIMLSFA NY-------- ---S------ --------TA IDVWSVGCIL AELLGG--KP IFKGRDY--- ---------- -------VDQ
 249241  ---------- ---------- ---------- ---PGG---G TSKAAGNQGF MTEYVATRWY RAPEIMLSFA NY-------- ---S------ --------TA IDVWSVGCIL AELLGG--KP IYKGRDY--- ---------- -------VDQ
 216822  ---------- ---------- ---------- ---PGG---G TSKAAGNQGF MTEYVATRWY RAPEIMLSFA NY-------- ---S------ --------TA IDVWSVGCIL AELLGG--KP IYKGRDY--- ---------- -------VDQ
 805839  ---------- ---------- ---------- ---PGG---G ASKSAGNQGF MTEYVATRWY RAPEIMLSFA NY-------- ---T------ --------TA IDVWSVGCIL AELLGG--KP IFKGRDY--- ---------- -------VDQ
 355172  ---------- ---------- ---------- ---PGG---G TSRAAGNQGF MTEYVATRWY RAPEIMLSFA NY-------- ---S------ --------TA IDVWSVGCIL AELLGG--KP IYKGRDY--- ---------- -------VDQ
 302825  ---------- ---------- ---------- ---PGG---G TSKAAGNQGF MTEYVATRWY RAPEIMLSFA NY-------- ---S------ --------TA IDVWSVGCIL AELLGG--KP IYKGRDY--- ---------- -------VDQ
 239059  ---------- ---------- ---------- ---PGS---G TSRAAGNQGF MTEYVATRWY RAPEIMLSFA NY-------- ---S------ --------TA IDVWSVGCIL AELLGG--RP IFKGRDY--- ---------- -------VDQ
 251409  ---------- ---------- ---------- ---PAGSGQG TAKNSANQGF MTEYVATRWY RAPEIMLSFA NY-------- ---G------ --------PA IDVWSVGCIL AELLAG--KP IFKGRDY--- ---------- -------VDQ
 251443  ---------- ---------- ---------- ---PAGSGQG TAKNSANQGF MTEYVATRWY RAPEIMLSFA NY-------- ---G------ --------PA IDVWSVGCIL AELLAG--KP IFKGRDY--- ---------- -------VDQ
  23484  ---------- ---------- ---------- ---SSP---- ----EGNVGF MTEYVATRWY RAPEIMLSFQ QY-------- ---T------ --------KA IDMWSVGCIF AEMLGG--RP LFKGRDY--- ---------- -------VDQ
 306334  ---------- ---------- ---------- ---KDP---- ----DTG-GF MTEYVATRWY RAPEIMLSFQ CY-------- ---T------ --------KA IDMWSVGCIF GELLGG--KP MFKGRDY--- ---------- -------VDQ
 306351  ---------- ---------- ---------- ---KDP---- ----DTG-GF MTEYVATRWY RAPEIMLSFQ CY-------- ---T------ --------KA IDMWSVGCIF GELLGG--KP MFKGRDY--- ---------- -------VDQ
 148766  ---------- ---------- ---------- ---MGP---- ----ETG-GV MTEYVATRWY RAPEIMLSYQ CY-------- ---S------ --------QA IDMWSVGCIF AELMGG--RP LFKGRDY--- ---------- -------VDQ
  84676  ---------- ---------- ---------- ---MGP---- ----ETG-GV MTEYVATRWY RAPEIMLSYQ CY-------- ---S------ --------QA IDMWSVGCIF AELMGG--RP LFKGRDY--- ---------- -------VDQ
  74082  ---------- ---------- ---------- ---MDP---- ----EENAGY MTEYVATRWY RAPEIMLSFQ SY-------- ---T------ --------KA IDVWSVGCIL AELLGG--KP FFKGRDY--- ---------- -------VDQ
  97664  ---------- ---------- ---------- ---MDP---- ----EENAGY MTEYVATRWY RAPEIMLSFQ SY-------- ---T------ --------KA IDVWSVGCIL AELLGG--KP FFKGRDY--- ---------- -------VDQ
  97676  ---------- ---------- ---------- ---MDP---- ----EENAGY MTEYVATRWY RAPEIMLSFQ SY-------- ---T------ --------KA IDVWSVGCIL AELLGG--KP FFKGRDY--- ---------- -------VDQ
 297954  ---------- ---------- ---------- ---IDP---- ----EENAGY LTEYVATRWY RAPEIMLSFQ SY-------- ---T------ --------KA IDVWSVGCIL AELLGG--RP FFKGRDY--- ---------- -------VDQ
 323749  ---------- ---------- ---------- ---VDP---- ----EENAGY MTEYVATRWY RAPEIMLSFQ SY-------- ---T------ --------KA IDVWSVGCIL AELLGG--RP FFKGRDY--- ---------- -------VDQ
1926181  ---------- ---------- ---------- ---VDP---- ----EENAGY MTEYVATRWY RAPEIMLSFQ SY-------- ---T------ --------KA IDVWSVGCIL AELLGG--RP FFKGRDY--- ---------- -------VDQ
1457364  ---------- ---------- ---------- ---VDP---- ----EENAGY MTEYVATRWY RAPEIMLSFQ SY-------- ---T------ --------KA IDVWSVGCIL AELLGG--RP FFKGRDY--- ---------- -------VDQ
  78435  ---------- ---------- ---------- ---VDP---- ----EENAGY MTEYVATRWY RAPEIMLSFQ SY-------- ---T------ --------KA IDVWSVGCIL AELLGG--RP FFKGRDY--- ---------- -------VDQ
1457361  ---------- ---------- ---------- ---VDP---- ----EENAGY MTEYVATRWY RAPEIMLSFQ SY-------- ---T------ --------KA IDVWSVGCIL AELLGG--RP FFKGRDY--- ---------- -------VDQ
1328540  ---------- ---------- ---------- ---TDPELAQ -AANA---GF MTEYVATRWY RAPEIMLSFQ NY-------- ---T------ --------TA IDLWSVGCIL AELFGG--RP IYKGRDY--- ---------- -------VDQ
1364342  ---------- ---------- ---------- ---TDPELAQ -AANA---GF MTEYVATRWY RAPEIMLSFQ NY-------- ---T------ --------TA ----TVGCIL AELFGG--RP IYKGRDY--- ---------- -------VDQ
 350087  ---------- ---------- ---------- ---PGAVQTD -QGQA---GF MTEYVATRWY RAPEIMLSFA NY-------- ---T------ --------SS IDTWSVGCIL AELLGG--RP IFKGEDY--- ---------- -------VDQ
 288508  ---------- ---------- ---------- ---TDAELAA -KGGAPVGGF MTEYVATRWY RAPEIMLSFQ NY-------- ---T------ --------TA IDIWSVGCIL AELLGG--KP IFKGRDY--- ---------- -------VDQ
 283473  ---------- ---------- ---------- ---PGSGAPA -SKGAANQGF MTEYVATRWY RAPEIMLSFA NY-------- ---G------ --------PA IDVWSVGCIL AELLAG--KP IFKGRDY--- ---------- -------VDQ
 734869  ---------- ---------- ---------- ---SDQGAEN -S-------L MTEYVATRWY RAPEVMLSFG NY-------- ---S------ --------TA IDIWSVGCIL AELHGR--KP IFKGRDY--- ---------- -------VDQ
   9394  ---------- ---------- ---------- ---P----NF -FKNKSQQT- ITNYVATRWY RAPELILSHR NY-------- ---S------ --------KS IDMWAVGCIL GEFYGR--RP LFVGNDQ--- ---------- -------LHQ
 167564  ---------- ---------- ---------- ---P----KF -MGSKSQ-A- ITNYVATRWY RAPELLLSRR NY-------- ---S------ --------KL VDLWSVGCIL GELYGR--KP LFMGNDQ--- ---------- -------IHQ
 113741  ---------- ---------- ---------- ---P----KF -FRT--RSAN ITNYVATRWY RAPELILSNK NY-------- ---T------ --------KS IDMWAVGCIF AELYGR--KP LFVGNDQ--- ---------- -------IHQ
   6575  ---------- ---------- ---------- ---P----KF -FRT--RSAN ITNYVATRWY RAPELILSNK NY-------- ---T------ --------KS IDMWAVGCIF AELYGR--RP LFVGNDQ--- ---------- -------IHQ
  30703  ---------- ---------- ---------- ---E----QY -MNKNHRSSN ITNYVATRWY RAPELILTRK QY-------- ---G------ --------KE IDMWAVGCIF GELYGR--KP LFIGDDQ--- ---------- -------MHQ
  65523  ---------- ---------- ---------- ---E----QY -MNKNHRSSN ITNYVATRWY RAPELILTRK QY-------- ---G------ --------KE IDMWAVGCIF GELYGR--KP LFIGDDQ--- ---------- -------MHQ
   5078  ---------- ---------- ---------- ---E----KF -MQNNRGSHN ITHYVATRWY RAPELILSRR TY-------- ---G------ --------KP VDMWAVGCIL GELYGR--KP MFIGNSQ--- ---------- -------IHQ
  54997  ---------- ---------- ---------- ---E----KF -MQNNRGSHN ITHYVATRWY RAPELILSRR TY-------- ---G------ --------KP VDMWAVGCIL GELYGR--KP MFIGNSQ--- ---------- -------IHQ
 114746  ---------- ---------- ---------- ---EDCFQKI -KNETGKQSH ITSYVATRWY RAPELMLCRK KY-------- ---T------ --------KA IDIWACGCIL AEFYGR--KP IFIGNDQ--- ---------- -------MHQ
 238776  ---------- ---------- ---------- ---I----NY -YQKKKFPQP ITNYVATRWY RAPELMLSSK NY-------- ---G------ --------KA VDLWAVGCIL CEFYGR--RP LFVGKDQ--- ---------- -------MHQ
  71410  ---------- ---------- ---------- ---DSAPDEN TP-------H LTEYVATRWY RAPEIMLAFR RYNVAI---- ---------- ---------- -DVWSIGCIL AELLMQ--KP LFKGKDYV-- ---------- --------DQ
 676425  ---------- ---------- ---------- ---NSKPDEE PT-------N MTEYVATRWY RAPEIMLAFK RYNTAI---- ---------- ---------- -DVWSIGCIF AELMLG--RP LFKGKDYV-- ---------- --------DQ
 676527  ---------- ---------- ---------- ---NSKPDEE PT-------N MTEYVATRWY RAPEIMLAFK RYNTAI---- ---------- ---------- -DVWSIGCIF AELMLG--RP LFKGKDAATV SSLF------ --------DQ
 152706  ---------- ---------- ---------- ---DAVPDEH TSG------N LTEYVATRWY RAPEIMLAFR GYSTAI---- ---------- ---------- -DVWSIGCIL AELLVG--RP LFKGKDYV-- ---------- --------DQ
  88536  ---------- ---------- ---------- ---DAVPDEH TSG------N LTEYVATRWY RAPEIMLAFR GYSTAI---- ---------- ---------- -DVWSIGCIF AELLVG--RP LFKGKDYV-- ---------- --------DQ
 122893  ---------- ---------- ---------- ---DQSPDEN ST-------Q MTEYVATRWY RAPEIMLAFR SRLLTVLP-- ---------- ---------- VDVWSIGCIF AELMLG--RP LFKGKDYV-- ---------- --------DQ
 151264  ---------- ---------- ---------- ---NAAPDEN AT-------M MTEYVATRWY RAPEIMLAYR AYNTAV---- ---------- ---------- -DIWAIGCIV AELLLG--RP LFKGKDYV-- ---------- --------DQ
  67588  ---------- ---------- ---------- ---DTAPDEN AS-------H LTEYVATRWY RAPEIMLAYR LVFISASL-- ---------- ---------- VDVWSIGCIL AELLIG--AP LFKGKEYELL S--------- --------SS
 153824  ---------- ---------- ---------- ---DARADEE NAT------H MTEYVATRWY RAPEVMLSFR SYNTAI---- ---------- ---------- -DVWSIGCIL GELLGC--RP MFKGKDYV-- ---------- --------DQ
 103584  ---------- ---------- ---------- ---DARADEE NAT------H MTEYVATRWY RAPEVMLSFR SYNTAI---- ---------- ---------- -DVWSIGCIL GELLGC--KP MFKGKDYV-- ---------- --------DQ
 118819  ---------- ---------- ---------- ---DSAPDEN TP-------H LTEYVATRWY RAPEIMLAFR RYN-VA---- ---------- ---------- IDVWSIGCIL AELLMQ--KP LFKGKDLTLL NSDLVQS--- -------VDQ
7227248  ---------- ---------- ---------- ---EATPDEN AS-------H LTEYVATRWY RAPEIMLAYR KYN-TA---- ---------- ---------- IDVWSIGCIL AELLIG--KP LFKGKDY--- ---------- -------VDQ
 240796  ---------- ---------- ---------- ---DAIPDEN AS-------H LTEYVATRWY RAPEIMLAYR KYD-TA---- ---------- ---------- IDVWSIGCIL AELLLG--AP LFKGKEY--- ---------- -------VDQ
 366893  ---------- ---------- ---------- ---DTAPDEN AS-------H LTEYVATRWY RAPEIMLAYR KYD-TA---- ---------- ---------- IDVWSIGCIL AELLIG--AP LFKGKDY--- ---------- -------VDQ
  79368  ---------- ---------- ---------- ---DAVPDEN AS-------H LTEYVATRWY RAPEIMLAYR KYD-TA---- ---------- ---------- IDMWSIGCIL AELLIG--GP LFKGRDY--- ---------- -------VDQ
 119618  ---------- ---------- ---------- ---GATP-EY TT-------H LTEYVATRWY RAPEIMLAFR GYN-TA---- ---------- ---------- IDVWSIGCIL AELLLG--KP LFKGKDY--- ---------- -------VDQ
 500495  ---------- ---------- ---------- ---DARPDEY AS-------Q LTEYVATRWY RAPEIMLAFR RYN-TA---- ---------- ---------- IDVWSIGCIL AELLLG--KP LFKGKDY--- ---------- -------VDQ
 449465  ---------- ---------- ---------- ---DARPDEY AS-------H LTEYVATRWY RAPEIMLAFR RYN-TA---- ---------- ---------- IDVWSIGCIL AELLLG--KP LFKGKEL--I DLQFLST--- -------VDQ
 509502  ---------- ---------- ---------- ---NSKPDEN AT-------H LTEYVATRWY RAPEIMLAFR RYN-TA---- ---------- ---------- IDVWSIGCIF AELLTG--KP LFKGKDY--- ---------- -------VDQ
 964561  ---------- ---------- ---------- ---DAIPDEN TSG------Y LTEYVATRWY RAPEIMLAFR GYT-TA---- ---------- ---------- IDVWSIGCIF AELLLG--RP LFKGKDY--- ---------- -------VDQ
 261448  ---------- ---------- ---------- ---DAVPDEH TSG------H LTEYVATRWY RAPEIMLAFR GYT-TA---- ---------- ---------- IDVWSIGCIF AELLLG--RP LFKGKDY--- ---------- -------VDQ
1150169  ---------- ---------- ---------- ---DASPDER V-G------H LTEYVATRWY RAPEIMLAFR LIPYLT---- ---------- ---------- VDVWSIGCIF AELLQG--RP MFKGRDY--- ---------- -------VDQ
 152363  ---------- ---------- ---------- ---DAVPDEN AT-------R LTEYVATRWY RAPEIMLAFP R-YNTA---- ---------- ---------- IDVWSIGCIL GELLLG--KP LFKGKDY--- ---------- -------VDQ
 379740  ---------- ---------- ---------- ---DAVPDEN AT-------R LTEYVATRWY RAPEIMLAFP R-YNTA---- ---------- ---------- IDVWSIGCIL GELLLG--KP LFKGKDY--- ---------- -------VDQ
 337664  ---------- ---------- ---------- ---DAVPDEN AT-------R LTEYVATRWY RAPEIMLAFP KSYQLALNFL CLLG------ --------IL VDVWSIGCIL GELLLG--KP LFKGKDY--- ---------- -------VDQ
 434295  ---------- ---------- ---------- ---DAVPDEN AT-------R LTEYVATRWY RAPEIMLAFP R-YNTA---- ---------- ---------- IDVWSIGCIL GELLLG--KP LFKGKDY--- ---------- -------VDQ
 517048  ---------- ---------- ---------- ---NSKPDEE PT-------N MTEYVATRWY RAPEIMLAFK RYNTA----- ---------- ---------- IDVWSIGCIF AELMLG--RP LFKGKDY--- ---------- -------VDQ
 731201  ---------- ---------- ---------- ---NSKPDEE PT-------N MTEYVATRWY RAPEIMLAFK RYNTA----- ---------- ---------- IDVWSIGCIL AELMLG--RP LFKGKDY--- ---------- -------VDQ
 768698  ---------- ---------- ---------- ---NSKPDEE PT-------N MTEYVATRWY RAPEIMLAFK RYNTA----- ---------- ---------- NHFSRGRSEH GSSILAYRRL TIVERSY--- ---------- -------VDQ
 517043  ---------- ---------- ---------- ---NSKPDEE PT-------N MTEYVATRWY RAPEIMLAFK RYNTA----- ---------- ---------- IDVWSIGCIF AELMLG--RP LFKGKDY--- ---------- -------VDQ
 740533  ---------- ---------- ---------- ---NSKPDEE PT-------N MTEYVATRWY RAPEIMLAFK ---------- ---------- ---------- -----GRSEH GSSILAYRRL TIVERSY--- ---------- -------VDQ
 178741  ---------- ---------- ---------- ---NSAPDEN PT-------L MTEYVATRWY RAPEIMLVYK AYNTA----- ---------- ---------- IDVWSIGCIM AELMTG--KP LFKGKDYGW- ---------- -------FDQ
  54225  ---------- ---------- ---------- ---NSAPDEN PT-------L MTEYVATRWY RAPEIMLVYK AYNTA----- ---------- ---------- IDVWSIGCIM AELMTG--KP LFKGKD---- ---------- --------DY
 163441  ---------- ---------- ---------- ---NSAPDEN TI-------L MTEYVATRWY RAPEIMLVYK AYDTA----- ---------- ---------- IDVWSIGCIM AELLMG--KP LFKGKDY--- ---------- -------VDQ
 327966  ---------- ---------- ---------- ---NSAPDEN PT-------L MTEYVATRWY RAPEIMLVYK AYNTA----- ---------- ---------- IDVWSIGCIM AELIMG--KP LFKGKDY--- ---------- -------VDQ
 722194  ---------- ---------- ---------- ---NSKSDDN PT-------M MTEYVATRWY RAPEIMLAYK EYNTA----- ---------- ---------- IDVWSIGCIL AELMLG--KP LFKGKDY--- ---------- -------VDQ
 128404  ---------- ---------- ---------- ---EHTPDGN AT-------Q LTEYVATRWY RAPELMLAFR QYNTA----- ---------- ---------- IDVWSIGCIF GELLLG--KP LFKGKDYG-- ---------- -------QNI
 167758  ---------- ---------- ---------- ---NAAPDEN AT-------M MTEYVATRWY RAPEIMLAYR AYNTA----- ---------- ---------- VDIWAIG--- ---------- ---------- ---------- -------LDQ
  26908  ---------- ---------- ---------- ---NAAPDEN AT-------M MTEYVATRWY RAPEIMLAYR AYNTA----- ---------- ---------- VD-------- ---------- ---------- ---------- ---------Q
 987811  ---------- ---------- ---------- ---ESVNGEN VT-------H LTEYVATRWY RAPEIMLAFN RYSTA----- ---------- ---------- IDVWSIGCVL AELLLG--KP LFKGKELAPT EIDDIHA--- -------VDQ
 913595  ---------- ---------- ---------- ---ESVNGEN VT-------H LTEYVATRWY RAPEIMLAFN RYSTA----- ---------- ---------- IDVWSIGCVL AELLLG--KP LFKGKELAPT EIDDIHA--- -------VDQ
 922124  ---------- ---------- ---------- ---ESVNGEN VT-------H LTEYVATRWY RAPEIMLAFN RYSTA----- ---------- ---------- IDVWSIGCVL AELLLG--KP LFKGKELAPT EIDDIHA--- -------VDQ
 319286  ---------- ---------- ---------- ---RPDPSEW DPNSQGPGGP MTEYVATRWY RAPEIMLSYK RYSTG----- ---------- ---------- IDVWSIGCIL AELLGG--KP IFKGKDY--- ---------- -------IEQ
 332677  ---------- ---------- ---------- ---RPSQNGM EEG----GLR LTEYVATRWY RAPEVMLSNS SYTTA----- ---------- ---------- MDVWSIGCIL AEVLGG--KP LFKGNDY--- ---------- -------VHQ
  75030  ---------- ---------- ---------- ---AAEEDGA IVT------Q LTEYVATRWY RAPEIMLSFK SYTTA----- ---------- ---------- IDVWSIGCIL AELLMG--KP IFKGKDY--- ---------- -------VDQ
 740342  ---------- ---------- ---------- ---AAEEDGA IVT------Q MTEYVATRWY RAPEIMLSFK SYTTA----- ---------- ---------- IDVWSIGCIL AELLMG--KP IFKGKDY--- ---------- -------VDQ
 769030  ---------- ---------- ---------- ---AAEEDGA IVT------Q MTEYVATRWY RAPEIMLSFK SYTTA----- ---------- ---------- IDVWSIGCIL AELLMG--KP IFKGKDY--- ---------- -------VDQ
 493654  ---------- ---------- ---------- ---NARTDEN TT-------Q MTEYVATRWY RAPEVMLSFK RYSFG----- ---------- ---------- IDVWSIGCVL GELVMG--KP VFKGKDY--- ---------- -------VDQ
  12113  ---------- ---------- ---------- ---NARVDDN TT-------Q MTEYVATRWY RAPEVMLSFK RYSFG----- ---------- ---------- IDVWSIGCVL GELVMG--KP VFKGKDY--- ---------- -------VDQ
 115011  ---------- ---------- ---------- ---NARADEN TT-------Q MTEYVATRWY RAPEVMLSFK RYSFG----- ---------- ---------- IDLWSIGCVL GELVMG--KP VFKGKDY--- ---------- -------VDQ
 192400  ---------- ---------- ---------- ---NARTDEN TT-------Q MTEYVATRWY RAPEVMLSFK RYSFG----- ---------- ---------- IDVWSIGCVL GELVMG--KP VFKGKDY--- ---------- -------VDQ
 543665  ---------- ---------- ---------- ---NARVDDN TT-------Q MTEYVATRWY RAPEVMLSFK RYSFG----- ---------- ---------- IDVWSIGCVL GELVMG--KP VFKGKDY--- ---------- -------VDQ
  91520  ---------- ---------- ---------- ---EANADEQ VG-------N LTEYVATRWY RAPEIMLAFK NYTTATYVLC ILTV------ --------YT VDVWSIGCIF AELLLG--RP LFKGKD---- ------Y--- -------VDQ
 127212  ---------- ---------- ---------- ---EANADEQ VG-------N LTEYVATRWY RAPEIMLAFK NYTTA----- ---------- ---------- IDVWSIGCIF AELLLG--RP LFKGKETTD- -----NY--- -------FDQ
 438879  ---------- ---------- ---------- ---DARPDHQ T--------H LTEYVATRWY RAPEIMLAFK RYN------- ---------- --------TA IDVWSIGCIL AELLMG--KP LFKGKDY--- ---------- -------VDQ
 174304  ---------- ---------- ---------- ---DARPDHQ T--------H LTEYVATRWY RAPEIMLAFK RYN------- ---------- --------TA IDVWSIGCIL AELLMG--KP LFKGKDY--- ---------- -------VDQ
 174289  ---------- ---------- ---------- ---DARPDHQ T--------H LTEYVATRWY RAPEIMLAFK RYN------- ---------- --------TA IDVWSIGCIL AELLMG--KP LFKGKDY--- ---------- -------VDQ
 244084  ---------- ---------- ---------- ---DASPDER VG-------H LTEYVATRWY RAPEIMLAFR GYD------- ---------- --------TA IDVWSIGCIF AELLQG--RP MFKGRDY--- ---------- -------VDQ
 123741  ---------- ---------- ---------- ---DQSPDEN ST-------Q MTEYVATRWY RAPELMLAFR SVQFHFNPPE LLTV------ --------LP VDVWSIGCIF AELMLG--KP LFKGKDY--- ---------- -------VDQ
 172164  ---------- ---------- ---------- ---DQSPDEN ST-------Q MTEYVATRWY RAPELMLAFR RYD------- ---------- --------TA IDVWSIGCIF AELMLG--KP LFKGKDY--- ---------- -------VDQ
  40528  ---------- ---------- ---------- ---EHTPDGN AT-------Q LTEYVATRWY RAPELMLAFR QYNT------ ---------- ---------A IDVWSIGCIF GELLLG--KP LFKGKESVSL L-----A--- -------VDQ
  39781  ---------- ---------- ---------- ---EHTPDGN AT-------Q LTEYVATRWY RAPELMLAFR SSLVCGDTHG --QI------ --------YA IDVWSIGCIF GELLLG--KP LFKGKESVSL L-----A--- -------VDQ
1155690  ---------- ---------- ---------- ---ATECEG- -ET----VTH MTEYVATRWY RAPEIMLSFK SYTTA----- ---------- ---------- IDVWSIGCIL AELLMG--KP IFKGKDY--- ---------- -------VDQ
 247448  ---------- ---------- ---------- ---RPSQNGM EEG----GLR LTEYVATRWY RAPEVMLSNS SYTTA----- ---------- ---------- MDVWSIGCIL AEVLGG--KP LFKGNDY--- ---------- -------VHQ
 269975  ---------- ---------- ---------- -----PARLQ TTTLDFPGGP LTEYVATRWY RAPEIMLCFR KGYGQQ---- ---------- ---------- IDMWSVGCIV AELLGG--KP IFAGKDY--- ---------- -------VDQ
 246067  GGEESSPPSA ERSFPESPRT NTPTSPKGTL PDGEEPARLQ TTTLDFPGGP LTEYVATRWY RAPEIMLCFR KGYGQQ---- ---------- ---------- IDMWSVGCIV AELLGG--KP IFAGKDY--- ---------- -------VDQ
```

```
  44396  GGEESSPPSA ERSFPESPRT NTPTSPKGTL PDGEEPARLQ TTTLDFPGGP LTEYVATRWY RAPEIMLCFR KGYGQQ---- ---------- ---------- IDMWSVGCIV AELLGG--KP IFAGKDY--- ---------- -------VDQ
 111248  ---------- ---------- ---------- S-QEDN---S G--------F MTEYVATRWY RAPEIMLTFK EYTKA----- ---------- ---------- IDVWSVGCIL AEMLSG--KP LFPGKDY--- ---------- -------HHQ
  54538  ---------- ---------- ---------- T-AEPSGTET G--------F MTEYVATRWY RAPEIMLTFK QYTKA----- ---------- ---------- IDIWSVGCIL AEMLSG--KP LFPGRDY--- ---------- -------HHQ
1341438  ---------- ---------- ---------- P-PPGLANDT ST-------F MTEYVATRWY RAPEVMLTFK EYTRA----- ---------- ---------- IDMWSVGCVL AEMLSG--KP LFPGRDY--- ---------- -------HHQ
1267921  ---------- ---------- ---------- P-PPGLANDT ST-------F MTEYVATRWY RAPEVMLTFK EYTRA----- ---------- ---------- IDMWSVGCVL AEMLSG--KP LFPGRDY--- ---------- -------HHQ
 692594  ---------- ---------- ---------- ---IQDPQ-- ---------- MTGYVSTRYY RAPEIMLTWQ KYDVE----- ---------- ---------- VDIWSAGCIF AEMLEG--KP LFPGKDH--- ---------- -------VNQ
 384872  ---------- ---------- ---------- ---SMDPEEN AG-------Y MTEYVATRWY RAPEIMLSFQ SYTKA----- ---------- ---------- IDVWSVGCIL AELLGG--KP FFKGRDY--- ---------- -------VDQ
 419622  ---------- ---------- ---------- ---SMDPEEN AG-------Y MTEYVATRWY RAPEIMLSFQ SYTKA----- ---------- ---------- IDVWSVGCIL AELLGG--KP FFKGRDY--- ---------- -------VDQ
 328081  ---------- ---------- ---------- PGAVQTDQGQ AG-------F MTEYVATRWY RAPEIMLSFA NYTSS----- ---------- ---------- IDTWSVGCIL AELLGG--KP IFKGEDY--- ---------- -------VDQ
  61803  ---------- ---------- ---------- ------EDSD LE-------- LTEYVVTRWY RAPELLLACR AYTKA----- ---------- ---------- IDIWSVGCIF AELLGR--RP LFPGEDY--- ---------- -------VHQ
  40664  ---------- ---------- ---------- ------EDSD LE-------- LTEYVVTRWY RAPELLLACR AYTKA----- ---------- ---------- IDIWSVGCIF AELLGR--RP LFPGEDY--- ---------- -------VHQ
 105104  ---------- ---------- ---------- ------DDDQ AA-------- LTEYVVTRWY RAPEIMLSHT MYTYA----- ---------- ---------- IDVWSVGCIL GELLGR--RP MFPGDDY--- ---------- -------IHQ
 147180  ---------- ---------- ---------- ------HAET ED-------- LTIYVVTRWY RAPELILACD HYSSA----- ---------- ---------- IDIWSVGCIF AELMTR--RP LFPGEDF--- ---------- -------LHQ
 148245  ---------- ---------- ---------- ------NTEY ED-------T LTEYVVTRWY RAPELLTDSR YYGSG----- ---------- ---------- VDVWSIGCIF GEILSR--RP IFQGKDY--- ---------- -------MHQ
 146852  ---------- ---------- ---------- ------NDEK LD-------- LTEYVVTRWY RAPEIMLSCK QYSAA----- ---------- ---------- IDVWSVGCIL GELLGR--KP MFPGEDY--- ---------- -------IHQ
  33818  ---------- ---------- ---------- ------NDEK LD-------- LTEYVVTRWY RAPEIMLSCK QYSAA----- ---------- ---------- IDVWSVGCIL GELLGR--KP MFPGEDY--- ---------- -------IHQ
 116469  ---------- ---------- ---------- ------NDEK LD-------- LTEYVVTRWY RAPEIMLSCK QYSAA----- ---------- ---------- IDVWSVGCIL GELLGR--KP MFPGEDY--- ---------- -------IHQ
 503885  ---------- ---------- ---------- ------RNMD LG-------F MTEYVATRWY RAPEVMMTSG EYTQA----- ---------- ---------- MDMWSVGVIL AELLGG--KT FFKGKNY--- ---------- -------LDT
 503914  ---------- ---------- ---------- ------RNMD LG-------F MTEYVATRWY RAPEVMMTSG EYTQA----- ---------- ---------- MDMWSVGVIL AELLGG--KT FFKGKNY--- ---------- -------LDT
6817382  ---------- ---------- ---------- ---EATPDE- ------NASH LTEYVATRWY RAPEIMLAYR KY-------- ---N------ --------TA IDVWSIGCIL AELLIG--AP LFK------- ---------- ----------
 145980  ---------- ---------- ---------- -GFSVDP--- ---EE-NAGY MTEYVATRWY RAPEIMLSFQ GYTKA----- ---------- ---------- IDVWSVGCIL AELL-G-SKP FFKGRD---- ---------- ------YVDQ
 328170  ---------- ---------- ---------- -GFSMDP--- ---EE-NAGY MTEYVATRWY RAPEIMLSFQ SYTKA----- ---------- ---------- IDVWSVGCIL AELL-G-GKP FFKGRD---- ---------- ------YVDQ
 691008  ---------- ---------- ---------- -GFSMDP--- ---EE-NAGY MTEYVATRWY RAPEIMLSFQ SYTKA----- ---------- ---------- IDVWSVGCIL AELL-G-GKP FFKGRD---- ---------- ------YVDQ
  73316  ---------- ---------- ---------- -GFSNEP--- ---DNVAAGF QTEYVATRWY RAPEIMLSFA EYTKA----- ---------- ---------- IDVWSVGCIL AELL-G-GKP FLKGKD---- ---------- ------YVDQ
1219281  ---------- ---------- ---------- -GFSVDP--- ---EE-NAGY MTEYVATRWY RAPEIMLSFQ SYTKASELPV SNGEIHGLEF RVLMSQLLCL VDVWSVGCIL AELL-G-GRP FFKGRD---- ---------- ------YVDQ
 422525  ---------- ---------- ---------- -GFSDHP--- ---EANA-GF MTEYVATRWY RAPEIMLSFR SYTKA----- ---------- ---------- IDMWSVGCIF AELL-G-GKP LFKGRD---- ---------- ------YVDQ
 212435  ---------- ---------- ---------- -GYSESP--- ---ESNAAGF MTEYVATRWY RAPEIMLSFQ NYTKA----- ---------- ---------- IDMWSVGCIF AEML-G-GKP LFKGRD---- ---------- ------YVDQ
 187307  ---------- ---------- ---------- -GYTPGGGTS --RVAGNSGF MTEYVATRWY RAPEIMLSFA NYSTA----- ---------- ---------- IDVWSIGCIL AELL-G-GKP IYKGRD---- ---------- ------YVDQ
 146240  ---------- ---------- ---------- -GYSPSGGNN NARGTANQGF MTEYVATRWY RAPEIMLSFA NYGPA----- ---------- ---------- IDVWSVGCIL AELL-A-GKP IFKGRD---- ---------- ------YVDQ
 156221  ---------- ---------- ---------- -GYTPGGGGT S-KAAGNQGF MTEYVATRWY RAPEIMLSFA NYSTA----- ---------- ---------- IDVWSVGCIL AELL-G-GKP IYRGKD---- ---------- ------YVDQ
  48865  ---------- ---------- ---------- -GYTPGGGA- S-RAAGNQGF MTE------- -APEIMLSFA NYTTA----- ---------- ---------- IDVWSVGCIL AELL-G-GKP IYKGRD---- ---------- ------YVDQ
 226510  ---------- ---------- ---------- -GYSPSGKNA A-KSAANQGF MTEYVATRWY RAPEIMLSFA NYGPA----- ---------- ---------- IDVWSVGCIL AELL-A-GKP IFKGRD---- ---------- ------YVDQ
 821431  ---------- ---------- ---------- -GYTPGSGTS --RAAGNQGF MTEYVATRWY RAPEIMLSFA NYTTA----- ---------- ---------- IDVWSVGCIL AELL-G-GKP IFKGRD---- ---------- ------YVDQ
  12784  ---------- ---------- ---------- -GFQEGGSA- ---ETGQAGF MTEYVATRWY RAPEIMLSFA NYNSS----- ---------- ---------- IDMWSVGCIL AELL-G-GRP IFKGRD---- ---------- ------YVDQ
 157692  ---------- ---------- ---------- -GFSVDP--- ----DENAGY MTEYVATRWY RAPEIMLSFQ SYTKA----- ---------- ---------- IDVWSVGCIL AELL-G-SKP FFKGRD---- ---------- ------YVDQ
  47352  ---------- ---------- ---------- -GVDDD---- -------QAA LTEYVVTRWY RAPEIMLSHT MYTYA----- ---------- ---------- IDVWSVGCIL GELL-G-RRP MFPGDD---- ---------- ------YIHQ
 136580  ---------- ---------- ---------- -S-AASTD-- --D--NQ-GF MTEYVATRWY RAPEIMLTFK EYTK------ ---------- ---------A IDVWSVGCIL AEML-S-GKP LFPGKD---- ---------- ------YHHQ
 422908  ---------- ---------- ---------- -S-AASTE-- --D--NS-GF MTEYVATRWY RAPEIMLTFK EYTK------ ---------- ---------A IDVWSVGCIL AEML-S-GKP LFPGKD---- ---------- ------YHHQ
  71188  ---------- ---------- ---------- -S-AASSE-- --D--NQ-GF MTEYVATRWY RAPEIMLTFK EYTK------ ---------- ---------A IDVWSVGCIL AEML-S-GKP LFPGKD---- ---------- ------YHHQ
2049832  ---------- ---------- ---------- -S-AASQE-- --D--NS-GF MTEYVATRWY RAPEIMLTFK EYTK------ ---------- ---------A IDVWSVGCIL AEML-S-GKP LFPGKD---- ---------- ------YHHQ
2023270  ---------- ---------- ---------- -S-AASQE-- --D--NS-GF MTEYVATRWY RAPEIMLTFK EYTK------ ---------- ---------A IDVWSVGCIL AEML-S-GKP LFPGKDCK-- ------QHLV FPSYSAHHHQ
  62157  ---------- ---------- ---------- -S-AASTE-- --D--NQ-GF MTEYVATRWY RAPEIMLTFK EYTK------ ---------- ---------A IDVWSVGCIL AEML-S-GKP LFPGKD---- ---------- ------YHHQ
 117327  ---------- ---------- ---------- -S-AASTE-- --D--NQ-GF MTEYVATRWY RAPEIMLTFK EYTK------ ---------- ---------A IDVWSVGCIL AEML-S-GKP LFPGKD---- ---------- ------YHHQ
1420954  ---------- ---------- ---------- -S-AASQE-- --D--NS-GF MTEYVATRWY RAPEIMLTFK EYTK------ ---------- ---------A IDVWSVGCIL AEML-S-GKP LFPGKDCKLD CYSAPSNDTK LTTHMTDHHQ
 146600  ---------- ---------- ---------- -S-AASQE-- --D--NS-GF MTEYVATRWY RAPEIMLTFK EYTK------ ---------- ---------A IDVWSVGCIL AEML-S-GKP LFPGKD---- ---------- ------YHHQ
 427542  ---------- ---------- ---------- -S-AASQE-- --D--NS-GF MTEYVATRWY RAPEIMLTFK EYTK------ ---------- ---------A IDVWSVGCIL AEML-S-GKP LFPGKD---- ---------- ------YHHQ
 524886  ---------- ---------- ---------- -S-AASQE-- --D--NS-GF MTEYVATRWY RAPEIMLTFK EYTK------ ---------- ---------A IDVWSVGCIL AEML-S-GKP LFPGKD---- ---------- ------YHHQ
 304061  ---------- ---------- ---------- -S-AASTE-- --D--NS-GF MTEYVATRWY RAPEIMLTFK EYTK------ ---------- ---------A IDVWSVGCIL AEML-S-GKP LFPGKD---- ---------- ------YHHQ
 262011  ---------- ---------- ---------- -S-AASQE-- --D--NS-GF MTEYVATRWY RAPEIMLTFK EYTK------ ---------- ---------A IDVWSVGCIL AEML-S-GKP LFPGKD---- ---------- ------YHHQ
 385460  ---------- ---------- ---------- -S-AASQE-- --D--NS-GF MTEYVATRWY RAPEIMLTFK EYTK------ ---------- ---------A IDVWSVGCIL AEML-S-GKP LFPGKD---- ---------- ------YHHQ
 322662  ---------- ---------- ---------- -S-AASQE-- --D--NS-GF MTEYVATRWY RAPEIMLTFK EYTK------ ---------- ---------A IDVWSVGCIL AEML-S-GKP LFPGKD---- ---------- ------YHHQ
1531698  ---------- ---------- ---------- -S-AASQE-- --D--NS-GF MTEYVATRWY RAPEIMLTFK EYTK------ ---------- ---------A IDVWSVGCIL AEML-S-GKP LFPGKD---- ---------- ------YHHQ
1273298  ---------- ---------- ---------- -S-AASQE-- --D--NS-GF MTEYVATRWY RAPEIMLTFK EYTK------ ---------- ---------A IDVWSVGCIL AEML-S-GKP LFPGKD---- ---------- ------YHHQ
 211888  ---------- ---------- ---------- -S-AASTD-- --D--NS-GF MTEYVATRWY RAPEIMLTFK EYTK------ ---------- ---------A IDVWSVGCIL AEML-S-GKP LFPGKD---- ---------- ------YHHQ
1140823  ---------- ---------- ---------- -S-AASTD-- --D--NS-GF MTEYVATRWY RAPEIMLTFK EYTK------ ---------- ---------A IDVWSVGCIL AEML-S-GKP LFPGKD---- ---------- ------YHHQ
  84032  ---------- ---------- ---------- -S-AASTD-- --D--NS-GF MTEYVATRWY RAPEIMLTFK EYTK------ ---------- ---------A IDVWSVGCIL AEML-S-GKP LFPGKD---- ---------- ------YHHQ
 337569  ---------- ---------- ---------- -S-AASTD-- --D--NS-GF MTEYVATRWY RAPEIMLTFK EYTK------ ---------- ---------A IDVWSVGCIL AEML-S-GKP LFPGKD---- ---------- ------YHHQ
 675946  ---------- ---------- ---------- -S-AASTE-- --D--NS-GF MTEYVATRWY RAPEIMLTFK EYTK------ ---------- ---------A IDVWSVGCIL AEML-S-GKP LFPGKD---- ---------- ------YHHQ
 188798  ---------- ---------- ---------- -S-AASTE-- --D--NS-GF MTEYVATRWY RAPEIMLTFK EYTK------ ---------- ---------A IDVWSVGCIL AEML-S-GKP LFPGKD---- ---------- ------YHHQ
 364357  ---------- ---------- ---------- -S-AASTE-- --D--NS-GF MTEYVATRWY RAPEIMLTFK EYTK------ ---------- ---------A IDVWSVGCIL AEML-S-GKP LFPGKD---- ---------- ------YHHQ
 417259  ---------- ---------- ---------- -S-AASTE-- --D--NS-GF MTEYVATRWY RAPEIMLTFK EYTK------ ---------- ---------A IDVWSVGCIL AEML-S-GKP LFPGKD---- ---------- ------YHHQ
 391850  ---------- ---------- ---------- -S-AASTE-- --D--NS-GF MTEYVATRWY RAPEIMLTFK EYTK------ ---------- ---------A IDVWSVGCIL AEML-S-GKP LFPGKD---- ---------- ------YHHQ
 420539  ---------- ---------- ---------- -S-AASTE-- --D--NQ-GF MTEYVATRWY RAPEIMLTFK EYTK------ ---------- ---------A IDVWSVGCIL AEML-S-GKP LFPGKD---- ---------- ------YHHQ
1649896  ---------- ---------- ---------- -S-AASTE-- --D--NS-GF MTEYVATRWY RAPEIMLTFK EYTK------ ---------- ---------A IDVWSVGCIL AEML-S-GKP LFPGKD---- ---------- ------YHHQ
 280694  ---------- ---------- ---------- -S-AASQE-- --D--NS-GF MTEYVATRWY RAPEIMLTFK EYTK------ ---------- ---------A IDVWSVGCIL AEML-S-GKP LFPGKD---- ---------- ------YHHQ
 381396  ---------- ---------- ---------- -S-AASTD-- --D--NS-GF MTEYVATRWY RAPEIMLTFK EYTK------ ---------- ---------A IDVWSVGCIL AEML-S-GKP LFPGKD---- ---------- ------YHHQ
 685453  ---------- ---------- ---------- -S-AASQE-- --D--NS-GF MTEYVATRWY RAPEIMLTFK EYTK------ ---------- ---------A IDVWSVGCIL AEML-S-GKP LFPGKD---- ---------- ------YHHQ
 284413  ---------- ---------- ---------- -S-ANSAD-- --E--NS-GF MTEYVATRWY RAPEIMLTFK EYTK------ ---------- ---------A IDVWSVGCIL AEML-S-GKP LFPGRD---- ---------- ------YHHQ
 384436  ---------- ---------- ---------- -S-AASTD-- --D--NS-GF MTEYVATRWY RAPEIMLTFK EYTK------ ---------- ---------A IDVWSVGCIL AEML-S-GKP LFPGKD---- ---------- ------YHHQ
 907966  ---------- ---------- ---------- -S-AASTE-- --D--NS-GF MTEYVATRWY RAPEIMLTFK EYTK------ ---------- ---------A IDVWSVGCIL AEML-S-GKP LFPGKD---- ---------- ------YHHQ
 205730  ---------- ---------- ---------- -I-MNIDH-- --ETNNP-GF MTEYVATRWY RAPEIMLTFK EYTQ------ ---------- ---------A IDVWSVGCIL AEML-S-GKP LFPGRD---- ---------- ------YHHQ
  86646  ---------- ---------- ---------- -S-TNLSE-- --E--HA-SF MTEYVATRWY RAPEIMLTFK EYTK------ ---------- ---------A IDVWSVGCIL AEML-S-AKP LFPGRD---- ---------- ------YHHQ
   9721  ---------- ---------- ---------- -S-TNLSE-- --E--HA-SF MTEYVATRWY RAPEIMLTFK EYTK------ ---------- ---------A IDVWSVGCIL AEML-S-GRP LFPGRD---- ---------- ------YHHQ
   2612  ---------- ---------- ---------- -L-VQNVSVN GFQGQQH-GY MTEYVATRWY RAPEIMLTFK EYTK------ ---------- ---------A IDMWSVGCIL AEML-T-GKA LFPGRD---- ---------- ------YNDQ
 102501  ---------- ---------- ---------- -S-IASSE-- ----DNY-GF MTEYVATRWY RAPEIMLTFQ EYTT------ ---------- ---------A IDVWSVGCIL AEMI-R-SKP LFPGRD---- ---------- ------YHNQ
  98652  ---------- ---------- ---------- -S-VASSE-- ----DNY-GY MTEYVATRWY RAPEIMLTFQ EYTT------ ---------- ---------A IDVWSVGCIL AEMI-S-SKP LFPGRD---- ---------- ------YHNQ
  78159  ---------- ---------- ---------- -S-VASQE-- ----DNF-GF MTEYVATRWY RAPEIMLTFQ EYTT------ ---------- ---------A IDVWSVGCIL AEML-S-GKP LFPGRD---- ---------- ------YHNQ
 240194  ---------- ---------- ---------- -S-IASSE-- ----DNF-GF MTEYVATRWY RAPEIMLTFQ EYTT------ ---------- ---------A IDVWSVGCIL AEML-T-GRP LFPGRD---- ---------- ------YHNQ
 193495  ---------- ---------- ---------- -S-VASSE-- ----NNF-GF MTEYVATRWY RAPEIMLTFQ EYTT------ ---------- ---------A IDVWSVGCIL AEML-S-GKP LFPGRD---- ---------- ------YHNQ
 171744  ---------- ---------- ---------- -S-VASSE-- ----NNF-GF MTEYVATRWY RAPEIMLTFQ EYTT------ ---------- ---------A IDVWSVGCIL AEML-S-GKP LFPGRD---- ---------- ------YHNQ
   4564  ---------- ---------- ---------- -S-IASKD-- ----DNY-GF MTEYVATRWY RAPEIMLTFQ QYTT------ ---------- ---------A IDVWSVGCIL AEML-S-GRP LFPGTD---- ---------- ------YHNQ
   6035  ---------- ---------- ---------- -S-VASQE-- ----DNF-GF MTEYVATRWY RAPEIMLTFQ EYTT------ ---------- ---------A IDVWSVGCIL AEML-S-GRP LFPGTD---- ---------- ------YHNQ
 256278  ---------- ---------- ---------- -S-AASTD-- ----DNS-GF MTEYVATRWY RAPEIMLTFK EYTK------ ---------- ---------A IDVWSVGCIL AEML-S-GKP LFPGKD---- ---------- ------YHHQ
 120199  ---------- ---------- ---------- -S-VKTA-EP --SGTET-GF MTEYVATRWY RAPEIMLTFK QYTK------ ---------- ---------A IDVWSVGCIL AEML-S-GKP LFPGRD---- ---------- ------YHHQ
1003994  ---------- ---------- ---------- -S-VRTA-EP --SGTET-GF MTEYVATRWY RAPEIMLTFK QYTK------ ---------- ---------A IDVWSVGCIL AEML-S-GKP LFPGRD---- ---------- ------YHHQ
 145010  ---------- ---------- ---------- -S-VKTA-EP --SGTET-GF MTEYVATRWY RAPEIMLTFK QYTK------ ---------- ---------A IDIWSVGCIL AEML-S-GKP LFPGRD---- ---------- ------YHHQ
 183924  ---------- ---------- ---------- -S-VRTA-EP --SGTET-GF MTEYVATRWY RAPEIMLTFK QYTK------ ---------- ---------A IDIWSVGCIL AEML-S-GKP LFPGRD---- ---------- ------YHHQ
 725911  ---------- ---------- ---------- -S-VKTA-EP --SGTET-GF MTEYVATRWY RAPEIMLTFK QYTK------ ---------- ---------A IDIWSVGCIL AEML-S-GKP LFPGRD---- ---------- ------YHHQ
1166877  ---------- ---------- ---------- -S-VKTA-EP --SGTET-GF MTEYVATRWY RAPEIMLTFK QYTK------ ---------- ---------A IDIWSVGCIL AEML-S-GKP LFPGRD---- ---------- ------YHHQ
 574353  ---------- ---------- ---------- -S-VKTA-EP --SGTET-GF MTEYVATRWY RAPEIMLTFK QYTK------ ---------- ---------A IDIWSVGCIL AEML-S-GKP LFPGRD---- ---------- ------YHHQ
 277341  ---------- ---------- ---------- -S-VKTA-EP --SGTET-GF MTEYVATRWY RAPEIMLTFK QYTK------ ---------- ---------A IDVWSVGCIL AEML-S-GKP LFPGRD---- ---------- ------YHHQ
 965410  ---------- ---------- ---------- -S-VKTA-EP --SGTET-GF MTEYVATRWY RAPEIMLTFK QYTK------ ---------- ---------A IDVWSVGCIL AEML-S-GKP LFPGRD---- ---------- ------YHHQ
1040134  ---------- ---------- ---------- -S-VKTA-EP --SGTET-GF MTEYVATRWY RAPEIMLTFK QYTK------ ---------- ---------A IDVWSVGCIL AEML-S-GKP LFPGRD---- ---------- ------YHHQ
 801239  ---------- ---------- ---------- -S-VKTA-EP --SGTET-GF MTEYVATRWY RAPEIMLTFK QYTK------ ---------- ---------A IDVWSVGCIL AEML-S-GKP LFPGRD---- ---------- ------YHHQ
 457049  ---------- ---------- ---------- -S-VKTA-EP --SGTET-GF MTEYVATRWY RAPEIMLTFK QYTK------ ---------- ---------A IDVWSVGCIL AEML-S-GKP LFPGRD---- ---------- ------YHHQ
 777636  ---------- ---------- ---------- -S-VKTA-EP --SGTET-GF MTEYVATRWY RAPEIMLTFK QYTK------ ---------- ---------A IDVWSVGCIL AEML-S-GKP LFPGRD---- ---------- ------YHHQ
 167531  ---------- ---------- ---------- -S-VKTA-EP --SGTET-GF MTEYVATRWY RAPEIMLTFK QYTK------ ---------- ---------A IDVWSVGCIL AEML-S-GKP LFPGRD---- ---------- ------YHHQ
 179443  ---------- ---------- ---------- -S-VKTA-EP --SGTET-GF MTEYVATRWY RAPEIMLTFK QYTK------ ---------- ---------A IDIWSVGCIL AEML-S-GKP LFPGRD---- ---------- ------YHHQ
 388203  ---------- ---------- ---------- -S-VKTA-EP --SGTET-GF MTEYVATRWY RAPEIMLTFK QYTK------ ---------- ---------A IDVWSVGCIL AEML-S-GKP LFPGRD---- ---------- ------YHHQ
 761034  ---------- ---------- ---------- -S-VKTA-EP --SGTET-GF MTEYVATRWY RAPEIMLTFK QYTK------ ---------- ---------A IDIWSVGCIL AEML-S-GKP LFPGRD---- ---------- ------YHHQ
 117742  ---------- ---------- ---------- -S-VKTA-EP --SGTET-GF MTEYVATRWY RAPEIMLTFK QYTK------ ---------- ---------A IDVWSVGCIL AEML-S-GKP LFPGRD---- ---------- ------YHHQ
 506090  ---------- ---------- ---------- -S-VRTA-EP --SGTET-GF MTEYVATRWY RAPEIMLTFK QYTK------ ---------- ---------A IDVWSVGCIL AEML-S-GKP LFPGRD---- ---------- ------YHHQ
  21911  ---------- ---------- ---------- -S-VRTA-EP --SGTET-GF MTEYVATRWY RAPEIMLTFK QYTK------ ---------- ---------A IDIWSVGCIL AEML-S-GKP LFPGRD---- ---------- ------YHHQ
7342346  ---------- ---------- ---------- -S-VKTA-EP --SGTET-GF MTEYVATRWY RAPEIMLTFK QYTK------ ---------- ---------A IDVWSVGCIL AEML-S-GKP LFPGRD---- ---------- ------YHHQ
 435735  ---------- ---------- ---------- -S-VKTA-EP --SGTET-GF MTEYVATRWY RAPEIMLTFK QYTK------ ---------- ---------A IDVWSVGCIL AEML-S-GKP LFPGRD---- ---------- ------YHHQ
 267232  ---------- ---------- ---------- -S-VKTA-EP --SGTET-GF MTEYVATRWY RAPEIMLTFK QYTK------ ---------- ---------A IDVWSVGCIL AEML-S-SKP LFPGRD---- ---------- ------YHHQ
 679497  ---------- ---------- ---------- -S-VKTA-EP --SGTET-GF MTEYVATRWY RAPEIMLTFK QYTK------ ---------- ---------A IDVWSVGCIL AEML-S-GKP LFPGRD---- ---------- ------YHHQ
 457636  ---------- ---------- ---------- -S-VKTA-EP --SGTET-GF MTEYVATRWY RAPEIMLTFK QYTK------ -VRDALVLQF STDLITKYKA IDVWSVGCIL AEML-S-GKP LFPGRD---- ---------- ------YHHQ
1340042  ---------- ---------- ---------- -S-VRTA-EP --NGGET-GF MTEYVATRWY RAPEIMLTFK QYTK------ ---------- ---------A IDIWSVGCIL AEML-S-GRP LFPGRD---- ---------- ------YHHQ
 225932  ---------- ---------- ---------- -S-VRTA-EQ -----ET-GF MTEYVATRWY RAPEIMLTFK QYTK------ ---------- ---------A IDVWSVGCIL AEML-S-GRP LFPGRD---- ---------- ------YHHQ
 346924  ---------- ---------- ---------- -S-CQTL-VP --AEGGQ-GF MTEYVATRWY RAPEVMLSFR MYNK------ ---------- ---------A LDVWSVGCIL AEML-S-GRP LFPGKD---- ---------- ------YHDQ
 760343  ---------- ---------- ---------- -S-VRTA-EP --NGGET-GF MTEYVATRWY RAPEIMLTFK QYTK------ ---------- ---------A IDIWSVGCIL AEML-S-GRP LFPGRD---- ---------- ------YHHQ
  15147  ---------- ---------- ---------- -S-TQTALHQ --EGAAN-GI MTEYVATRWY RAPEVMLSFK QYTK------ ---------- ---------A IDVWSIGCIL AEML-N-GKP LFPGKD---- ---------- ------YHDQ
 108680  ---------- ---------- ---------- -S-AF---MG --EQEAT-GF MTEYVATRWY RAPEIMLTFK EYTK------ ---------- ---------A IDIWSVGCIL AEML-N-GKP LFPGRD---- ---------- ------YHHQ
 724148  ---------- ---------- ---------- -S-AF---MG --EQEAT-GF MTEYVATRWY RAPEIMLTFK EYTK------ ---------- ---------A IDIWSVGCIL AEML-N-GKP LFPGRD---- ---------- ------YHHQ
 276168  ---------- ---------- ---------- -S-AN---QP --EAEGT-GF MTEYVATRWY RAPEIMLTFK EYTK------ ---------- ---------A IDVWSVGCIL AEML-S-GKP LFPGRD---- ---------- ------YHHQ
 194959  ---------- ---------- ---------- -S-VK-TSIP --PGKEV-GL MTEYVATRWY RAPEIMLSFK MYTK------ ---------- ---------A IDIWAVGCIL AELL-T-GRP LFPGRD---- ---------- ------YSHQ
 345309  ---------- ---------- ---------- -S-VK-TSVP --AGKEV-GL MTEYVATRWY RAPEIMLSFK MYTK------ ---------- ---------A IDIWAVGCIL AELL-T-GRP LFPGRD---- ---------- ------YSHQ
7189608  ---------- ---------- ---------- -S-VK-TSVP --GGKEV-GL MTEYVATRWY RAPEIMLSFK MYTK------ ---------- ---------A IDIWAIGCIL AELL-T-GRP LFPGRD---- ---------- ------YSHQ
 439783  ---------- ---------- ---------- -S-VK-TSVP --GGKEV-GL MTEYVATRWY RAPEIMLSFK MYTK------ ---------- ---------A IDIWAVGCIL AELL-T-GRP LFPGRD---- ---------- ------YSHQ
  73775  ---------- ---------- ---------- -S-VK-TSVP --GGKEV-GL MTEYVATRWY RAPEIMLSFK MYTK------ ---------- ---------A IDIWAVGCVL AELL-T-GRP LFPGRD---- ---------- ------YSHQ
 681486  ---------- ---------- ---------- -S-VK-TSVV --GGKEV-GI MTEYVATRWY RAPEIMLSFK MYTK------ ---------- ---------A IDIWAVGCIL AELL-T-GRP LFPGRD---- ---------- ------YSHQ
 671673  ---------- ---------- ---------- -S-VK-TSVV --GGKEV-GI MTEYVATRWY RAPEIMLSFK MYTK------ ---------- ---------A IDIWAVGCIL AELL-T-GRP LFPGRD---- ---------- ------YSHQ
 829815  ---------- ---------- ---------- -S-VK-TTVP --IGKEK-GL MTEYVATRWY RAPEIMLSFK MYTK------ ---------- ---------A VDIWAVGCIL AELL-S-GRP LFPGRD---- ---------- ------YSHQ
 413627  ---------- ---------- ---------- -S-VKQSSTQ --GGKDI-GV MTEYVATRWY RAPEIMLSFK MYTK------ ---------- ---------A IDVWAVGCIL AEML-S-GRP LFPGRD---- ---------- ------YGHQ
 109090  ---------- ---------- ---------- -S-VN-SASP --DGKDA-GL LTEYVATRWY RAPEIMLSFK RYTK------ ---------- ---------A IDMWSVGCIL AELL-T-GKP LFPGRD---- ---------- ------YSHQ
  43366  ---------- ---------- ---------- -S-VKQSSTE ---GKET-GL MTEYVATRWY RAPEIMLSFK MYTK------ ---------- ---------A IDVWAVGCIL AELL-T-GRP LFPGRD---- ---------- ------YHSQ
 706326  ---------- ---------- ---------- -S-TR-STNP --GGKEI-GL MTEYVATRWY RAPEIMLSFK MYTK------ ---------- ---------A IDVWAVGCIL AELI-N-GRP LFPGRD---- ---------- ------YGHQ
1157202  ---------- ---------- ---------- -S-TR-STNP --GGKEV-GL MTEYVATRWY RAPEIMLSFK MYTK------ ---------- ---------A IDIWAVGCIL AELI-N-GRP LFPGRD---- ---------- ------YGHQ
 743174  ---------- ---------- ---------- -S-MR-STHP --DGKEA-GL MTEYVATRWY RAPEIMLSFK MYTK------ ---------- ---------A IDIWAVGCIL AELL-T-GRP LFPGRD---- ---------- ------YGHQ
   5331  ---------- ---------- ---------- -S-TR-STNP --GGKEV-GL MTEYVATRWY RAPEIMLSFK MYTK------ ---------- ---------A IDIWAVGCIL AELI-N-GRP LFPGRD---- ---------- ------YGHQ
 713967  ---------- ---------- ---------- -S-MR-STHP --DGKEA-GL MTEYVATRWY RAPEIMLSFK MYTK------ ---------- ---------A IDIWAVGCIL AELL-T-GRP LFPGRD---- ---------- ------YGHQ
 151043  ---------- ---------- ---------- -S-TR-STSP --GGKEA-GL MTEYVATRWY RAPEIMLSFK MYTK------ ---------- ---------A IDVWAVGCIL AELL-N-GRP LFPGRD---- ---------- ------YGHQ
```

```
 481570 ---------- ---------- ---------- -S-TR-STNP --GGKEA-GL MTEYVATRWY RAPEIMLSFK MYTK------ ---------- ---------A IDIWAVGCIL AELL-N-GRP LFPGRD---- ---------- ------YGHQ
 104738 ---------- ---------- ---------- -S-TR-STNP --GGKEA-GL MTEYVATRWY RAPEIMLSFK MYTK------ ---------- ---------A IDIWAVGCIL AELL-N-GRP LFPGRD---- ---------- ------YGHQ
 689474 ---------- ---------- ---------- -S-IN-STAP --GGKEM-GL MTEYVATRWY RAPEIMLSFK MYTK------ ---------- ---------A VDIWAVGCVL AELL-T-GKP LFPGRD---- ---------- ------YGHQ
 179470 ---------- ---------- ---------- -S-IN-STAP --GGKEM-GL MTEYVATRWY RAPEIMLSFK MYTK------ ---------- ---------A IDIWAVGCIL AELL-N-GRP LFPGRD---- ---------- ------YGHQ
  31001 ---------- ---------- ---------- -S-IK-STSP --GGKEM-GL MTEYVATRWY RAPEIMLSFK MYTK------ ---------- ---------A VDVWAVGCIL AELL-T-GKA LFPGRD---- ---------- ------YGHQ
 155293 ---------- ---------- ---------- -N-AH-TGTP --AG-ES-GL MTEYVATRWY RAPEIMLSFK MYTK------ ---------- ---------A IDLWAVGCIL AEMM-M-GKP LFPGRD---- ---------- ------YGHQ
  83606 ---------- ---------- ---------- -N-AH-TGTP --AG-ES-GL MTEYVATRWY RAPEIMLSFK MYTK------ ---------- ---------A IDLWAVGCIL AEMM-M-GKP LFPGRD---- ---------- ------YGHQ
  79026 ---------- ---------- ---------- -S-ARPPPN- -LANDSN-TF MTEYVATRWY RAPEVMLTFK EYTR------ ---------- ---------A IDMWSVGCVL AEML-S-GKP LFPGRD---- ---------- ------YHHQ
  79053 ---------- ---------- ---------- -S-ARPPPN- -LANDSN-TF MTEYVATRWY RAPEVMLTFK EYTR------ ---------- ---------A IDMWSVGCVL AEML-S-GKP LFPGRD---- ---------- ------YHHQ
 188294 ---------- ---------- ---------- -S-ARPPPN- -V-DDSS-TF MTEYVATRWY RAPEVMLTFK EYTR------ ---------- ---------A IDMWSVGCVL AEML-S-GKP LFPGRD---- ---------- ------YHHQ
 422236 ---------- ---------- ---------- -S-ARPPPN- -V-DDSS-TF MTEYVATRWY RAPEVMLTFK EYTR------ ---------- ---------A IDVWSVGCVL AEML-S-GKP LFPGRD---- ---------- ------YHHQ
  59295 ---------- ---------- ---------- -S-ATPPPN- -FANDSS-TF MTEYVATRWY RAPEVMLTFK EYTR------ ---------- ---------A IDIWSVGCVL AEML-S-GKP LFPGRD---- ---------- ------YHHQ
  91727 ---------- ---------- ---------- -S-ARPPPN- -VANDSS-TF MTEYVATRWY RAPEVMLTFK EYTR------ ---------- ---------A IDIWSVGCVL AEML-S-GKP LFPGRD---- ---------- ------YHHQ
  92869 ---------- ---------- ---------- -S-ARPPPN- -VANDSS-TF MTEYVATRWY RAPEVMLTFK EYTR------ ---------- ---------A IDIWSVGCVL AEML-S-GKP LFPGRD---- ---------- ------YHHQ
 513929 ---------- ---------- ---------- -S-ARPPPN- -VANDSS-TF MTEYVATRWY RAPEVMLTFK EYTR------ ---------- ---------A IDIWSVGCVL AEML-S-GKP LFPGRD---- ---------- ------YHHQ
 166192 ---------- ---------- ---------- -S-ARPPPN- -VANDSS-TF MTEYVATRWY RAPEVMLTFK EYTR------ ---------- ---------A IDIWSVGCVL AEML-S-GKP LFPGRD---- ---------- ------YHHQ
 210022 ---------- ---------- ---------- -S-ARPPPN- -F-DESS-TF MTEYVATRWY RAPEVMLTFK DYTR------ ---------- ---------A IDMWSVGCVL AEML-S-GRP LFPGRD---- ---------- ------YHHQ
 847083 ---------- ---------- ---------- -S-ARPPPN- -VANDSS-TF MTEYVATRWY RAPEVMLTFK EYTR------ ---------- ---------A IDMWSVGCVL AEML-S-GKP LFPGRD---- ---------- ------YHDQ
 819022 ---------- ---------- ---------- -S-ARPSPD- -VANDSS-TF MTEYVATRWY RAPEVMLTFK EYTR------ ---------- ---------A IDMWSVGCVL AEML-S-GKP LFPGRD---- ---------- ------YHHQ
  50473 ---------- ---------- ---------- -S-ARPSPD- -VANDSS-TF MTEYVATRWY RAPEVMLTFK EYTR------ ---------- ---------A IDMWSVGCVL AEML-S-GKP LFPGRD---- ---------- ------YHHQ
 850567 ---------- ---------- ---------- -S-ARPPPN- -VANDSS-TF MTEYVATRWY RAPEVMLTFK EYTR------ ---------- ---------A IDMWSVGCVL AEML-S-GRP LFPGRD---- ---------- ------YHDQ
 183076 ---------- ---------- ---------- -S-ARPPPN- -IANDSS-TF MTEYVATRWY RAPEVMLTFK EYTR------ ---------- ---------A IDMWSVGCVL AEML-S-GKP LFPGRD---- ---------- ------YHDQ
1114014 ---------- ---------- ---------- -S-ARPPPN- -VANDSS-TF MTEYVATRWY RAPEVMLTFK EYTR------ ---------- ---------A IDMWSVGCVL AEML-S-GKP LFPGRD---- ---------- ------YHDQ
  47661 ---------- ---------- ---------- -S-ARPPPN- -V-DDTS-TF MTEYVATRWY RAPEVMLTFK EYTR------ ---------- ---------A IDIWSVGCVL AEML-S-GKP LFPGRD---- ---------- ------YHHQ
6536531 ---------- ---------- ---------- -S-ARPHPD- -A-DDTS-TF MTEYVATRWY RAPEVMLTFK EYTR------ ---------- ---------A IDVWSVGCVL AEML-S-GRP LFPGRD---- ---------- ------YHHQ
 277624 ---------- ---------- ---------- -S-ARPPPD- -A-DDTS-TF MTEYVATRWY RAPEVMLTFK EYTR------ ---------- ---------A IDVWSVGCVL AEML-S-GRP LFPGRD---- ---------- ------YHHQ
  77457 ---------- ---------- ---------- -S-ARPPPD- -A-DDTS-TF MTEYVATRWY RAPEVMLTFK EYTR------ ---------- ---------A IDVWSVGCVL AEML-S-GRP LFPGRD---- ---------- ------YHHQ
  89431 ---------- ---------- ---------- -S-ARPPPD- -V-DDTS-TF MTEYVATRWY RAPEVMLTFK EYTR------ ---------- ---------A IDMWSVGCVL AEML-S-GRP LFPGRD---- ---------- ------YHHQ
 484632 ---------- ---------- ---------- -S-ARPPPD- -I-DDTS-TF MTEYVATRWY RAPEVMLTFK EYTR------ ---------- ---------A IDMWSVGCVL AEML-S-GRP LFPGRD---- ---------- ------YHHQ
 161998 ---------- ---------- ---------- -S-ARPPPN- -V-DDTS-TF MTEYVATRWY RAPEVMLTFK EYTR------ ---------- ---------A IDIWSVGCIL AEML-S-GKP LFPGRD---- ---------- ------YHHQ
 324877 ---------- ---------- ---------- -S-ARPPPD- -V-DDSS-NF LTEYVATRWY RAPEVMLTFK EYTR------ ---------- ---------A IDIWSVGCIL AEML-S-GKP LFPGRD---- ---------- ------YHHQ
 117759 ---------- ---------- ---------- -S-SLPPPNS SAANDAS-TF MTEYVATRWY RAPEVMLTFK EYTR------ ---------- ---------A IDIWSVGCVL AEML-S-GKP LFPGRD---- ---------- ------YHHQ
 489827 ---------- ---------- ---------- -S-ASPPPG- -VQLDAA-TF MTEYVATRWY RAPEVMLTFK EYTR------ ---------- ---------A IDVWSVGCVL AEML-S-GKP LFPGRD---- ---------- ------YHHQ
 115935 ---------- ---------- ---------- -S-ASPPPG- -VQLDAA-TF MTEYVATRWY RAPEVMLTFK EYTR------ ---------- ---------A IDVWSVGCVL AEML-S-GKP LFPGRD---- ---------- ------YHHQ
 482781 ---------- ---------- ---------- -S-ASPPPG- -VQLDAA-TF MTEYVATRWY RAPEVMLTFK EYTR------ ---------- ---------A IDVWSVGCVL AEML-S-GKP LFPGRD---- ---------- ------YHHQ
 545919 ---------- ---------- ---------- -S-ASPPPG- -VQLDAA-TF MTEYVATRWY RAPEVMLTFK EYTR------ ---------- ---------A IDVWSVGCVL AEML-S-GKP LFPGRD---- ---------- ------YHHQ
6536562 ---------- ---------- ---------- -S-ARPHPD- --ADDTS-TF MTEYVATRWY RAPEVMLTFK EYTR------ ---------- ---------A IDVWSVGCVL AEML-S-GRP LFPGRD---- ---------- ------YHHQ
 309997 ---------- ---------- ---------- -SSAHPPPD- -TQGGGN-AY MTEYVATRWY RAPEVMLSFK EYTK------ ---------- ---------A IDMWSVGCIL AEMI-N-GRP LFPGRD---- ---------- ------YHHQ
 316873 ---------- ---------- ---------- -SSAHPPPD- -TQGGGN-AY MTEYVATRWY RAPEVMLSFK EYTK------ ---------- ---------A IDMWSVGCIL AEMI-N-GRP LFPGRD---- ---------- ------YHHQ
 429267 ---------- ---------- ---------- -S-ARPPPD- --VDDSS-NF LTEYVATRWY RAPEVMLTFK EYTR------ ---------- ---------A IDIWSVGCVL AEML-S-GKP LFPGRD---- ---------- ------YHHQ
 103465 ---------- ---------- ---------- -FEQSSHQQ- --DPDERINM MTEYVATRWY RAPEIMLSSS QYTT------ ---------- ---------A IDIWSMGCIL AELF-M-AAP LFPGKD---- ---------- ------YRHQ
 167039 ---------- ---------- ---------- -IDDRKEGQT TSSPEAKISM LTEYVATRWY RAPEIMLSAS KYTT------ ---------- ---------A IDIWSVGCIL AELL-N-RRP LFPGKD---- ---------- ------YRHQ
   8637 ---------- ---------- ---------- -I--DANST- --KGEDKISF LTEYVATRWY RAPEIMLSSA QYSK------ ---------- ---------A IDIWSIGCIV AEMI-L-REP LFPGTD---- ---------- ------YRNQ
  65963 ---------- ---------- ---------- -VGRDNSSS- --VSESKDSF LTEYVATRWY RAPEIMLTSS QYSK------ ---------- ---------A IDIWSLACIL AEML-L-REP FLPGKD---- ---------- ------YRHQ
 246409 ---------- ---------- ---------- -S-ARPPPN- --VANDSSTF MTEYVATRWY RAPEVMLTFK EYTR------ ---------- ---------A IDIWSVGCVL AEML-S-GKP LFPGRD---- ---------- ------YHHQ
 246352 ---------- ---------- ---------- -S-ARPPPN- --VANDSSTF MTEYVATRWY RAPEVMLTFK EYTR------ ---------- ---------A IDIWSVGCVL AEML-S-GKP LFPGRD---- ---------- ------YHHQ
 668220 ---------- ---------- ---------- -SVKTAEP-- --SGTE-TGF MTEYVATRWY RAPEIMLTFK QYTK------ ---------- ---------A IDIWSVGCIL AEML-S-GKP LFPGRD---- ---------- ------YHHQ
 619500 ---------- ---------- ---------- -SVKTAEP-- --SGTE-TGF MTEYVATRWY RAPEIMLTFK QYTK------ ---------- ---------A IDVWSVGCIL AEML-S-GKP LFPGRD---- ---------- ------YHHQ
  64492 ---------- ---------- ---------- -SVKTAEP-- --SGTE-TGF MTEYVATRWY RAPEIMLTFK QYTK------ ---------- ---------A IDIWSVGCIL AEML-S-GKP LFPGRD---- ---------- ------YHHQ
  80250 ---------- ---------- ---------- -SAFMG---- ---EQEATGF MTEYVATRWY RAPEIMLTFK EYTK------ ---------- ---------A IDIWSVGCIL AEML-N-GKP LFPGRD---- ---------- ------YHHQ
  21917 ---------- ---------- ---------- -SASPPPG-- --VQLDAATF MTEYVATRWY RAPEVMLTFK EYTR------ ---------- ---------A IDVWSVGCVL AEML-S-GKP LFPGRD---- ---------- ------YHHQ
 600175 ---------- ---------- ---------- -SARPPPN-- --FANDSSTF MTEYVATRWY RAPEVMLTFK EYTR------ ---------- ---------A IDIWSVGCVL AEML-S-GKP LFPGRD---- ---------- ------YHHQ
 721307 ---------- ---------- ---------- -SARPSPD-- --VANDSSTF MTEYVATRWY RAPEVMLTFK EYTR------ ---------- ---------A IDMWSVGCVL AEML-S-GKP LFPGRD---- ---------- ------YHHQ
 847084 ---------- ---------- ---------- -SARPPPN-- --VANDSSTF MTEYVATRWY RAPEVMLTFK EYTR------ ---------- ---------A IDMWSVGCVL AEML-S-GKP LFPGRD---- ---------- ------YHDQ
 280696 ---------- ---------- ---------- -SAASQE--- -----DNSGF MTEYVATRWY RAPEIMLTFK EYTK------ ---------- ---------A IDVWSVGCIL AEML-S-GKP LFPGKD---- ---------- ------YHHQ
 908144 ---------- ---------- ---------- -SAASTE--- -----DNSGF MTEYVATRWY RAPEIMLTFK EYTK------ ---------- ---------A IDVWSVGCIL AEML-S-GKP LFPGKD---- ---------- ------YHHQ
 148879 ---------- ---------- ---------- -SAASSE--- -----DNQGF MTEYVATRWY RAPEIMLTFK EYTK------ ---------- ---------A IDVWSVGCIL AEML-S-GKP LFPGKD---- ---------- ------YHHQ
 102030 ---------- ---------- ---------- -GSKD----- --ENDNKESF LTEYVATRWY RAPEIMLSFK EYTK------ ---------- ---------A IDIWSVGCIM AEML-S-KMP LFPGHD---- ---------- ------YHHQ
  92334 ---------- ---------- ---------- -GSKD----- --ENDNKESF LTEYVATRWY RAPEIMLSFK EYTK------ ---------- ---------A IDIWSVGCIM AEML-S-KMP LFPGHD---- ---------- ------YHHQ
 146634 ---------- ---------- ---------- -GLEDS---- ------NLE- LTEYVVTRWY RAPEIMLACR EYTK------ ---------- ---------S IDVWAVGCIF AELL-V-RKP LVPGEDY--- ---------- -------IHQ
  72485 ---------- ---------- ---------- -QVRNM---- ------DLGF MTEYVATRWY RAPEVMMTSG EYTQ------ ---------- ---------A MDMWSVGVIL AELL-G-GKT FFKGKNY--- ---------- -------LDT
 124061 ---------- ---------- ---------- -SVKTSVV-- ---GGKEVGI MTEYVATRWY RAPEIMLSFK MYTK------ ---------- ---------A IDIWAVGCIL AELL-T-GRP LFPGRDY--- ---------- -------SHQ
 109792 ---------- ---------- ---------- -SVRTAEQ-- ------ETGF MTEYVATRWY RAPEIMLTFK QYTK------ ---------- ---------A IDVWSVGCIL AEML-S-GRP LFPGRDY--- ---------- -------HHQ
 408982 ---------- ---------- ---------- -SVKTAEP-- ---SGTETGF MTEYVATRWY RAPEIMLTFK QYTK------ ---------- ---------A IDVWSVGCIL AEML-S-GKP LFPGRDY--- ---------- -------HHQ
 149279 ---------- ---------- ---------- -SVKTAEP-- ---SGTETGF MTEYVATRWY RAPEIMLTFK QYTK------ ---------- ---------A IDVWSVGCIL AEML-S-GKP LFPGRDY--- ---------- -------HHQ
  13526 ---------- ---------- ---------- -SARPPPD-- ---ADDTSTF MTEYVATRWY RAPEVMLTFK EYTR------ ---------- ---------A IDVWSVGCVL AEML-S-GRP LFPGRDY--- ---------- ---------
 465497 ---------- ---------- ---------- -SARPPPD-- ---IDDTSTF MTEYVATRWY RAPEVMLTFK EYTR------ ---------- ---------A IDMWSVGYHH QLSL-I-LET LGTPSID--- ---------- ----------
 216389 ---------- ---------- ---------- -SARPPPD-- ---ADDTSTF MTEYVATRWY RAPEVMLTFK EYTR------ ---------- ---------A IDVWSVGCIM AEML-S-GRP LFPGRDY--- ---------- -------HHQ
  17191 ---------- ---------- ---------- -SVLTAEP-- ---GG-ENGF MTEXXXXXXX XXXXXXXTFK QYTK------ ---------- ---------A IDIWSVGCIL AEMI-S-GRP LFPGRDY--- ---------- -------HHQ
 530963 ---------- ---------- ---------- -IQDPQ---- ---------- MTGYVSTRYY RAPEIMLTWQ KYDV------ ---------- ---------A VDIWSTGCIF AEML-E-GKP LFPGKDRECF PDLPMFPPEL DID----VNQ
  84099 ---------- ---------- ---------- -IQDPQ---- ---------- MTGYVSTRYY RAPEIMLTWQ KYDV------ ---------- ---------E VDIWSAGCIF AEML-E-GKP LFPGKDH--- ---------- -------VNQ
 928715 ---------- ---------- --------RD VSEERKKNGG KEEANDQIGF MTEYVATRWY RAPEIMLTFK EYTK------ ---------- ---------A IDMWSVGCVL AEMI-G-SRP LFPGRD---- ---------- ------YHHQ
 480104 ---------- ---------- ---------- -SVKTSIP-- ---PGKEVGL MTEYVATRWY RAPEIMLSFK MYTK------ ---------- ---------A IDIWAVGCIL AELL-T-GRP LFPGRD---- ---------- ------YSHQ
  37144 ---------- ---------- ---------- -CINSTAP-- ---GAKEAGL MTEYVATRWY RAPEIMLSFK MYTK------ ---------- ---------A VDLWAVGCIL AELL-N-QKP LFPGRD---- ---------- ------YGHQ
 993637 ---------- ---------- ---------- -CINSTAP-- ---GAKEAGL MTEYVATRWY RAPEIMLSFK MYTK------ ---------- ---------A VDLWAVGCIL AELL-N-QKP LFPGRD---- ---------- ------YGHQ
 706327 ---------- ---------- ---------- -STRSTNP-- ---GGKEIGL MTEYVATRWY RAPEIMLSFK MYTK------ ---------- ---------A IDVWAVGCIL AELI-N-GRP LFPGRD---- ---------- ------YGHQ
   5366 ---------- ---------- ---------- -STRSTNP-- ---GGKEVGL MTEYVATRWY RAPEIMLSFK MYTK------ ---------- ---------A IDIWAVGCIL AELI-N-GRP LFPGRD---- ---------- ------YGHQ
 144998 ---------- ---------- ---------- -SVKTAEP-- ---SGTETGF MTEYVATRWY RAPEIMLTFK QYTK------ ---------- ---------A IDIWSVGCIL AEML-S-GKP LFPGRD---- ---------- ------YHHQ
 232199 ---------- ---------- ---------- -SVKTAEP-- ---SGTETGF MTEYVATRWY RAPEIMLTFK QYTK------ ---------- ---------A IDIWSVGCIL AEML-S-GRP LFPGRD---- ---------- ------YHHQ
  60405 ---------- ---------- ---------- -SVKTAEP-- ---SGTETGF MTEYVATRWY RAPEIMLTFK QYTK------ ---------- ---------A IDVWSVGCIL AEML-S-GKP LFPGRD---- ---------- ------YHHQ
 572635 ---------- ---------- ---------- -SVKTAEP-- ---SGTETGF MTEYVATRWY RAPEIMLTFK QYTK------ ---------- ---------A IDVWSVGCIL AEML-S-GKP LFPGRD---- ---------- ------YHHQ
  37912 ---------- ---------- ---------- -SVKTAEP-- ---SGTETGF MTEYVATRWY RAPEIMLTFK QYTK------ ---------- ---------A IDVWSVGCIL AEML-S-GKP LFPGRD---- ---------- ------YHHQ
 819552 ---------- ---------- ---------- -SVKTAEP-- ---SGTETGF MTEYVATRWY RAPEIMLTFK QYTK------ ---------- ---------A IDVWSVGCIL AEML-S-GKP LFPGRD---- ---------- ------YHHQ
1026351 ---------- ---------- ---------- -SARPPPNVD -----DSSTF MTEYVATRWY RAPEVMLTFK EYTR------ ---------- ---------A IDMWSVGCVL AEML-S-GKP LFPGRD---- ---------- ------YHHQ
 352542 ---------- ---------- ---------- -SARPPPNVD -----DSSTF MTEYVATRWY RAPEVMLTFK EYTR------ ---------- ---------A IDVWSVGCVL AEML-S-GKP LFPGRD---- ---------- ------YHHQ
  42114 ---------- ---------- ---------- -SARPPPDAD -----DTSTF MTEYVATRWY RAPEVMLTFK EYTR------ ---------- ---------A IDVWSVGCIM AEML-S-GRP LFPGRD---- ---------- ------YHHQ
 204011 ---------- ---------- ---------- -SASPPPGVQ L----DAATF MTEYVATRWY RAPEVMLTFK EYTR------ ---------- ---------A IDVWSVGCVL AEML-S-GKP LFPGRD---- ---------- ------YHHQ
  46962 ---------- ---------- ---------- -SAASQE--- -----DNSGF MTEYVATRWY RAPEIMLTFK EYTK------ ---------- ---------A IDVWSVGCIL AEML-S-GKP LFPGKD---- ---------- ------YHHQ
   3452 ---------- ---------- ---------- -SAASTE--- -----DNSGF MTEYVATRWY RAPEIMLTFK EYTK------ ---------- ---------A IDVWSVGCIL AEML-S-GKP LFPGKD---- ---------- ------YHHQ
1531701 ---------- ---------- ---------- -SAASQE--- -----DNSGF MTEYVATRWY RAPEIMLTFK EYTK------ ---------- ---------A IDVWSVGCIL AEML-S-GKP LFPGKD---- ---------- ------YHHQ
 375250 ---------- ---------- ---------- -SAASTE--- -----DNSGF MTEYVATRWY RAPEIMLTFK EYTK------ ---------- ---------A IDVWSVGCIL AEML-S-GKP LFPGKD---- ---------- ------YHHQ
 391393 ---------- ---------- ---------- -SAASTE--- -----DNQGF MTEYVATRWY RAPEIMLTFK EYTK------ ---------- ---------A IDVWSVGCIL AEML-S-GKP LFPGKD---- ---------- ------YHHQ
 518679 ---------- ---------- ---------- -SAASTD--- -----DNSGF MTEYVATRWY RAPEIMLTFK EYTK------ ---------- ---------A IDVWSVGCIL AEML-S-GKP LFPGKD---- ---------- ------YHHQ
1272884 ---------- ---------- ---------- -SAASQE--- -----DNSGF MTEYVATRWY RAPEIMLTFK EYTK------ ---------- ---------A IDVWSVGCIL AEML-S-GKP LFPGKD---- ---------- ------YHHQ
 108249 ---------- ---------- ---------- -STNLSE--- -----EHASF MTEYVATRWY RAPEIMLTFK EYTK------ ---------- ---------A IDVWSVGCIL AEML-S-AKP LFPGRD---- ---------- ------YHHQ
 111885 ---------- ---------- --------C- -LLSTGST-- ---NKDMVGF MTEYVATRWY RAPEIMLSFQ EYST------ ---------- ---------A IDIWSCGCIL GELL-NDGKA VFPGRD---- ---------- ------YHHQ
 228039 ---------- ---------- --------VD TLKKTNDL-- ---YNKDPGI LTEYVATRWY RAPEIMLTPS QYST------ ---------- ---------A VDIWSVGCIL AELL-IC-TP LFPGRD---- ---------- ------YTSQ
 117728 ---------- ---------- ---------- -SITEDP--- -----ANKAA YSEYTATRWY RSPEILMGST KYST------ ---------- ---------G VDIWALGCIL GEML-L-GKP LFPGTS---- ---------- ------TMNQ
 267276 ---------- ---------- ---------- -SVKTAEP-- ---SGTETGF MTEYVATRWY RAPEIMLTFK QYTK------ ---------- ---------A IDVWSVGCIL AEML-S-SKP LFPGRD---- ---------- ------YHHQ
 295270 ---------- ---------- ---------- -SVKTAEP-- ---SGTETGF MTEYVATRWY RAPEIMLTFK QYTK------ ---------- ---------A IDIWSVGCIL AEML-S-GKP LFPGRD---- ---------- ------YHHQ
 174214 ---------- ---------- ---------- ---------- -----IQDPQ MTGYVSTRYY RAPEIMLTWQ KYDV------ ---------- ---------E VDIWSAGCIF AEML-E-GKP LFPGKD---- ---------- ------HVNQ
 111562 ---------- ---------- ---------- ---------- -----IQDPQ MTGYVSTRYY RAPEIMLTWQ KYDV------ ---------- ---------E VDIWSAGCIF AEML-E-GKP LFPGKD---- ---------- ------HVNQ
 385026 ---------- ---------- ---------- ---------- -----IQDPQ MTGYVSTRYY RAPEIMLTWQ KYDV------ ---------- ---------E VDIWSAGCIF AEML-E-GKP LFPGKD---- ---------- ------HVNQ
  47937 ---------- ---------- ---------- ---------- -----IQDPQ MTGYVSTRYY RAPEIMLTWQ KYDV------ ---------- ---------E VDIWSVGCIF AEML-E-GKP LFPGKD---- ---------- ------HVNQ
 191658 ---------- ---------- ---------- ---------- -----IQDPQ MTGYVSTRYY RAPEIMLTWQ KYDV------ ---------- ---------E VDIWSAGCIF AEML-E-GKP LFPGKD---- ---------- ------HVNQ
 361397 ---------- ---------- ---------- ---------- -----IQDPQ MTGYVSTRYY RAPEIMLTWQ KYDI------ ---------- ---------E VDIWSAGCIF AEML-E-GKP LFPGKD---- ---------- ------HVNQ
 368356 ---------- ---------- ---------- ---------- -----IQDPQ MTGYVSTRYY RAPEIMLTWQ KYDV------ ---------- ---------E VDIWSAGCIF AEML-E-GKP LFPGKD---- ---------- ------HVNQ
 422287 ---------- ---------- ---------- ---------- -----IQDPQ MTGYVSTRYY RAPEIMLTWQ KYDV------ ---------- ---------E VDIWSAGCIF AEML-E-GKP LFPGKD---- ---------- ------HVNQ
1051981 ---------- ---------- ---------- ---------- -----IQDPQ MTGYVSTRYY RAPEIMLTWQ KYDV------ ---------- ---------E VDIWSAGCIF AEML-D-GKP LFPGKD---- ---------- ------HVNQ
 512786 ---------- ---------- ---------- ---------- -----IQDPQ MTGYVSTRYY RAPEIMLTWQ KYDI------ ---------- ---------E VDIWSAGCIF AEML-E-GKP LFPGKD---- ---------- ------HVNQ
 700253 ---------- ---------- ---------- ---------- -----IQDPQ MTGYVSTRYY RAPEIMLTWQ KYDV------ ---------- ---------E VDIWSAGCIF AEML-E-GKP LFPGKD---- ---------- ------HVNQ
 171641 ---------- ---------- ---------- ---------- -----IQDPQ MTGYVSTRYY RAPEIMLTWQ KYDV------ ---------- ---------E VDIWSAGCIF AEML-E-GKP LFPGKD---- ---------- ------HVNQ
 447383 ---------- ---------- ---------- ---------- -----IQDPQ MTGYVSTRYY RAPEIMLTWQ KYDI------ ---------- ---------E VDIWSAGCIF AEML-D-GKP LFPGKD---- ---------- ------HVNQ
 134451 ---------- ---------- ---------- ---------- -----IQDPQ MTGYVSTRYY RAPEIMLTWQ KYDV------ ---------- ---------E VDIWSAGCIF AEML-E-GKP LFPGKD---- ---------- ------HVNQ
 525556 ---------- ---------- ---------- ---------- -----IQDPQ MTGYVSTRYY RAPEIMLTWQ KYDV------ ---------- ---------E VDIWSAGCIF AEML-D-GKP LFPGKD---- ---------- ------HVNQ
1776212 ---------- ---------- ---------- ---------- -----IQDPQ MTGYVSTRYY RAPEIMLTWQ KYDV------ ---------- ---------E VDIWSAGCIF AEML-E-GKP LFPGKD---- ---------- ------HVNQ
 443108 ---------- ---------- ---------- ---------- -----IQDPQ MTGYVSTRYY RAPEIMLTWQ KYDI------ ---------- ---------E VDIWSAGCIF AEML-E-GKP LFPGKD---- ---------- ------HVNQ
 129540 ---------- ---------- ---------- ---------- -----VQDPQ MTGYVSTRYY RAPEIMLTWQ KYDV------ ---------- ---------E VDVWSAGCIF AEML-S-GKP LFPGKD---- ---------- ------HVNQ
 341724 ---------- ---------- ---------- ---------- -----VQDPQ MTGYVSTRYY RAPEIMLTWQ KYDV------ ---------- ---------E VDVWSAGCIF AEML-S-GKP LFPGKD---- ---------- ------HVNQ
 138417 ---------- ---------- ---------- ---------- -----VQDPQ MTGYVSTRYY RAPEIMLTWQ KYDV------ ---------- ---------E VDVWSAGCIF AEML-S-GKP LFPGKD---- ---------- ------HVNQ
 158191 ---------- ---------- ---------- ---------- -----IQDPQ MTGYVSTRYY RAPEIMLTWQ KYDV------ ---------- ---------E VDIWSAGCIF AEML-E-GKP LFPGKD---- ---------- ------HVNQ
 376076 ---------- ---------- ---------- ---------- -----VQDPQ MTGYVSTRYY RAPEIMLTWQ KYDV------ ---------- ---------E VDIWSAGCIF AEML-E-GKP LFPGKD---- ---------- ------HVNQ
 368607 ---------- ---------- ---------- ---------- -----IQDPQ MTGYVSTRYY RAPEIMLTWQ KYDV------ ---------- ---------E VDIWSAGCIF AEML-E-GKP LFPGKD---- ---------- ------HVNQ
1312592 ---------- ---------- ---------- ---------- -----LQDPQ MTGYVSTRYY RAPEIMLTWQ KYDV------ ---------- ---------A VDIWSAGCIF AEML-E-GKP LFPGKD---- ---------- ------HVNQ
  46955 ---------- ---------- ---------- ---------- -----IQDPQ MTGYVSTRYY RAPEIMLTWQ KYDV------ ---------- ---------A VDIWSAGCIF AEML-E-GKP LFPGKD---- ---------- ------HVHQ
1164008 ---------- ---------- ---------- ---------- -----IQDPQ MTGYVSTRYY RAPEIMLTWQ KYDV------ ---------- ---------E VDIWSAGCIF AEML-E-GKP LFPGKD---- ---------- ------HVHQ
 600771 ---------- ---------- ---------- ---------- -----IQDPQ MTGYVSTRYY RAPEIMLTWQ KYDV------ ---------- ---------A VDIWSAGCIF AEML-E-GKP LFPGKD---- ---------- ------HVHQ
 161654 ---------- ---------- ---------- ---------- -----IQDPQ MTGYVSTRYY RAPEIMLTWQ KYDV------ ---------- ---------E VDIWSAGCIF AEML-E-GKP LFPGKD---- ---------- ------HVNQ
 149140 ---------- ---------- ---------- ---------- -----IQDPQ MTGYVSTRYY RAPEIMLTWQ KYDV------ ---------- ---------A VDIWSAGCIF AEML-E-GKP LFPGKD---- ---------- ------HVNQ
 474692 ---------- ---------- ---------- ---------- -----IQDPQ MTGYVSTRYY RAPEIMLTWQ KYDV------ ---------- ---------E VDIWSTGCIF AEML-E-GKP LFPGKD---- ---------- ------HVNQ
 134758 ---------- ---------- ---------- ---------- -----IQDPQ MTGYVSTRYY RAPEIMLTWQ KYDV------ ---------- ---------A VDIWSAGCIF AEML-E-GKP LFPGKD---- ---------- ------HVNQ
 177791 ---------- ---------- ---------- ---------- -----IQDPQ MTGYVSTRYY RAPEIMLTWQ KYDV------ ---------- ---------E VDIWSTGCIF AEML-E-GKP LFPGKD---- ---------- ------HVNQ
 132015 ---------- ---------- ---------- ---------- -----IQDPQ MTGYVSTRYY RAPEIMLTWQ KYDV------ ---------- ---------A VDIWSTGCIF AEML-E-GKP LFPGKD---- ---------- ------HVNQ
```

```
   917530  ---------- ---------- ---------- ---------- -----IQDPQ MTGYVSTRYY RAPEIMLTWQ KYDV------ ---------- ---------A VDVWSTGCIF AEML-E-GKP LFPGKD---- ---------- ------HVNQ
   428496  ---------- ---------- ---------- ---------- -----IQDPQ MTGYVSTRYY RAPEIMLTWQ KYDV------ ---------- ---------A VDIWSTGCIF AEML-E-GKP LFPGKD---- ---------- ------HVNQ
  1276586  ---------- ---------- ---------- ---------- -----IQDPQ MTGYVSTRYY RAPEIMLTWQ KYDV------ ---------- ---------A VDIWSAGCIF AEML-E-GKP LFPGKD---- ---------- ------HVHQ
   115601  ---------- ---------- ---------- ---------- -----IQDPQ MTGYVSTRYY RAPEIMLTWQ KYDV------ ---------- ---------A VDIWSTGCIF AEML-E-GKP LFPGKD---- ---------- ------HVNQ
   171809  ---------- ---------- ---------- ---------- -----IQDPQ MTGYVSTRYY RAPEIMLTWQ KYDV------ ---------- ---------A VDIWSTGCIF AEML-E-GKP LFPGKD---- ---------- ------HVNQ
   571775  ---------- ---------- ---------- ---------- -----IQDPQ MTGYVSTRYY RAPEIMLTWQ KYDV------ ---------- ---------A VDIWSTGCIF AEML-E-GKP LFPGKD---- ---------- ------HVNQ
   722302  ---------- ---------- ---------- ---------- -----LQDPQ MTGYVSTRYY RAPEIMLTWQ KYDV------ ---------- ---------A VDIWSAGCIF AEML-E-GKP LFPGKD---- ---------- ------HVNQ
   289694  ---------- ---------- ---------- ---------- -----IQDPQ MTGYVSTRYY RAPEIMLTWQ KYDV------ ---------- ---------A VDVWSAGCIF AEML-E-GRP LFPGKD---- ---------- ------HVNQ
   137978  ---------- ---------- ---------- ---------- -----IQDPQ MTGYVSTRYY RAPEIMLTWQ KYDV------ ---------- ---------A VDIWSTGCIF AEML-E-SKP LFPGKD---- ---------- ------HVHQ
    19116  ---------- ---------- ---------- ---------- -----IQDPQ MTGYVSTRYY RAPEIMLTWQ KYDV------ ---------- ---------A VDIWSAGCIF AEML-E-GKP LFPGKD---- ---------- ------HVHQ
   129801  ---------- ---------- ---------- ---------- -----IQDPQ MTGYVSTRYY RAPEIMLTWQ KYDI------ ---------- ---------E VDMWSAGCIF AEMI-L-GKP LFPGKD---- ---------- ------HVHQ
    80382  ---------- ---------- ---------- ---------- -----IQDPQ MTGYVSTRYY RAPEIMLTWQ KYDT------ ---------- ---------E VDIWSAGCIF AEMI-E-GKP LFPGKD---- ---------- ------HVDQ
   141916  ---------- ---------- ---------- ---------- -----IQDPQ MTGYVSTRYY RAPEIMLTWQ KYDT------ ---------- ---------E VDIWSAGCIF AEMI-E-GKP LFPGKD---- ---------- ------HVDQ
   187748  ---------- ---------- ---------- ---------- -----IQDPQ MTGYVSTRYY RAPEIMLTWQ KYDT------ ---------- ---------E VDLWSVGCIL SEMI-E-GKP LFPGKD---- ---------- ------HVHQ
   168984  ---------- ---------- ---------- ---------- -----VQDPQ MTGYVSTRYY RAPEIMLTWQ KYDT------ ---------- ---------E VDIWSAGCIF AEMI-E-GKP LFPGKD---- ---------- ------HVHQ
    71808  ---------- ---------- ---------- ---------- -----VQDPQ MTGYVSTRYY RAPEIMLTWQ KYDV------ ---------- ---------A VDIWSTGCIF AEMF-E-GTP LFPGKD---- ---------- ------HVDQ
      432  ---------- ---------- ---------- ---------- -----IQDAQ MTGYVSTRYY RAPEIMLTWQ TYDV------ ---------- ---------E VDIWSTGCIF AEMI-Q-GKP LFPGKD---- ---------- ------HINQ
    59395  ---------- ---------- ---------- ---------- -----VTESQ MTGYVSTRYY RAPEIMLTWQ KYDE------ ---------- ---------A VDIWSAGCIL AEMI-E-GKP LFPGKE---- ---------- ------HVHQ
     2865  ---------- ---------- ---------- ---------- -----IQDPQ MTGYVSTRYY RAPEIMLTWQ KYDI------ ---------- ---------E VDIWSAGCIF AEML-E-GKP LFPGKD---- ---------- ------HVNQ
   189766  ---------- ---------- ---------- ---------- -----IQDPQ MTGYVSTRYY RAPEIMLTWQ KYDV------ ---------- ---------E VDIWSAGCIF AEML-E-GKP LFPGKD---- ---------- ------HVNQ
    88183  ---------- ---------- ---------- ---------- -----IQDPQ MTGYVSTRYY RAPEIMLTWQ KYDV------ ---------- ---------A VDIWSTGCIF AEML-E-GKP LFPGKD---- ---------- ------HVNQ
   216492  ---------- ---------- ---------- ---------- -----IQDPQ MTGYVSTRYY RAPEIMLTWQ KYDV------ ---------- ---------A VDIWSTGCIF AEML-E-GKP LFPGKD---- ---------- ------HVNQ
   188879  ---------- ---------- ---------- ---------- -----IQDPQ MTGYVSTRYY RAPEIMLTWQ KYDV------ ---------- ---------A VDIWSTGCIF AEML-E-GKP LFPGKD---- ---------- ------HVNQ
   182850  ---------- ---------- ---------- ---------- -----IQDPQ MTGYVSTRYY RAPEIMLTWQ KYDV------ ---------- ---------A VDIWSTGCIF AEML-E-GKP LFPGKD---- ---------- ------HVNQ
   174697  ---------- ---------- ---------- ---------- -----IQDPQ MTGYVSTRYY RAPEIMLTWQ KYDV------ ---------- ---------A VDIWSTGCIF AEML-E-GKP LFPGKD---- ---------- ------HVNQ
   934899  ---------- ---------- ---------- ---------- -----IQDPQ MTGYVSTRYY RAPEIMLTWQ KYDV------ ---------- ---------A VDIWSTGCIF AEML-E-GKP LFPGKD---- ---------- ------HVNQ
   362397  ---------- ---------- ---------- ---------- -----IQDPQ MTGYVSTRYY RAPEIMLTWQ KYDV------ ---------- ---------A VDIWSTGCIF AEML-E-GKP LFPGKD---- ---------- ------HVNQ
   416973  ---------- ---------- ---------- ---------- -----IQDPQ MTGYVSTRYY RAPEIMLTWQ KYDV------ ---------- ---------A VDIWSTGCIF AEML-E-GKP LFPGKD---- ---------- ------HVNQ
   551386  ---------- ---------- ---------- ---------- -----IQDPQ MTGYVSTRYY RAPEIMLTWQ KYDV------ ---------- ---------A VDIWSTGCIF AEML-E-GKP LFPGKD---- ---------- ------HVNQ
    15239  ---------- ---------- ---------- ---------- -----IQDPQ MTGYVSTRYY RAPEIMLTWQ KYDV------ ---------- ---------A VDIWSTGCIF AEML-E-GKP LFPGKD---- ---------- ------HVNQ
   497504  ---------- ---------- ---------- ---------- -----IQDPQ MTGYVSTRYY RAPEIMLTWQ KYDV------ ---------- ---------E VDIWSAGCIF AEML-E-GKP LFPGKD---- ---------- ------HVNQ
  1117682  ---------- ---------- ---------- ---------- -----IKDNQ MTGYVSTRYY RAPEIMLTWQ QYDV------ ---------- ---------A VDMWSAGCIF AEML-E-GRP LFPGRD---- ---------- ------HVHQ
   252496  ---------- ---------- ---------- ---------- -----IQDPQ MTGYVSTRYY RAPEIMLTWQ KYDV------ ---------- ---------A VDIWSTGCIF AEML-E-GKP LFPGKD---- ---------- ------HVNQ
   148867  ---------- ---------- ---------- ---------- -----TNDPQ MTGYVSTRYY RAPEIMLTWQ KYDR------ ---------- ---------A VDLWSVGCIF AELL-T-GKP LFPGKD---- ---------- ------HVNQ
   423027  ---------- ---------- ---------- ---------- -----LQEPQ MTGYVSTRYY RAPEIMLTWQ KYGM------ ---------- ---------Q VDIWSAGCIV AEML-R-GKP LFPGKD---- ---------- ------HIHQ
  1104996  ---------- ---------- ---------- ---------- -----LQEPQ MTGYVSTRYY RAPEIMLTWQ KYGM------ ---------- ---------Q VDIWSAGCIV AEML-R-GKP LFPGKD---- ---------- ------HINQ
    29266  ---------- ---------- ---------- ---------- -----IQDPQ MTGYVSTRYY RAPEIMLTWQ KYDI------ ---------- ---------E VDIWSAGCIF AEML-D-GKP LFPGKD---- ---------- ------HVNQ
   171653  ---------- ---------- ---------- ---------- -----IQDPQ MTGYVSTRYY RAPEIMLTWQ KYDV------ ---------- ---------E VDIWSAGCIF AEML-E-GKP LFPGKD---- ---------- ------HVNQ
   117063  ---------- ---------- ---------- ---------- -----IQDPQ MTGYVSTRYY RAPEIMLTWQ KYDV------ ---------- ---------E VDIWSAGCIF AEML-E-GKP LFPGKD---- ---------- ------HVNQ
   158104  ---------- ---------- ---------- ---------- -----IQDPQ MTGYVSTRYY RAPEIMLTWQ KYDV------ ---------- ---------E VDIWSAGCIF AEML-E-GKP LFPGKD---- ---------- ------HVNQ
   160940  ---------- ---------- ---------- ---------- -----IQDPQ MTGYVSTRYY RAPEIMLTWQ KYDV------ ---------- ---------E VDIWSAGCIF AEML-E-GKP LFPGKD---- ---------- ------HVNQ
     2813  ---------- ---------- ---------- ---------- -----IQDPQ MTGYVSTRYY RAPEIMLTWQ KYDI------ ---------- ---------E VDIWSAGCIF AEML-E-GKP LFPGKD---- ---------- ------HVNQ
   129541  ---------- ---------- ---------- ---------- -----VQDPQ MTGYVSTRYY RAPEIMLTWQ KYDV------ ---------- ---------E VDVWSAGCIF AEML-S-GKP LFPGKD---- ---------- ------HVNQ
   257371  ---------- ---------- ---------- ---------- -----IQDPQ MTGYVSTRYY RAPEIMLTWQ KYDV------ ---------- ---------E VDIWSAGCIF AEML-E-GKP LFPGKD---- ---------- ------HVNQ
   389086  ---------- ---------- ---------- ---------- -----EQDHQ MTGYVVTRYY RAPEVMLTWQ QYNY------ ---------- ---------A VDIWSVGCIL AEML-R-GKP LFPGKN---- ---------- ------HIDQ
    73401  ---------- ---------- ---------- ---------- -----VKEHQ MTGYVSTRYY RAPEIMLTWQ RYGV------ ---------- ---------E VDLWSTGCIL AEML-S-GKP LFPGKD---- ---------- ------HINQ
  1139913  ---------- ---------- ---------- ---------- -----VKEQQ MTGYVSTRYY RAPEIMLTWQ RYGV------ ---------- ---------E VDLWSTGCIL AEML-T-GKP LFPGKD---- ---------- ------HINQ
  1168371  ---------- ---------- ---------- ---------- -----VKEQQ MTGYVSTRYY RAPEIMLTWQ RYGV------ ---------- ---------E VDLWSTGCIL AEML-T-GKP LFPGKD---- ---------- ------HINQ
   698519  ---------- ---------- ---------- ---------- -----VKEHQ MTGYVSTRYY RAPEIMLTWQ RYGV------ ---------- ---------E VDLWSTGCIL AEML-S-GKP LFPGKD---- ---------- ------HINQ
   332332  ---------- ---------- ---------- ---------- -----VQDPQ MTGYVSTRYY RAPEIMLTWQ KYDV------ ---------- ---------E VDVWSAGCIF AEML-S-GKP LFPGKD---- ---------- ------HVNQ
   243292  ---------- ---------- ---------- ---------- -----IQDPQ MTGYVSTRYY RAPEIMLTWQ KYDV------ ---------- ---------E VDIWSAGCIF AEML-E-GKP LFPGKD---- ---------- ------HVNQ
   243775  ---------- ---------- ---------- ---------- -----IQDPQ MTGYVSTRYY RAPEIMLTWQ KYDV------ ---------- ---------E VDIWSAGCIF AEML-E-GKP LFPGKD---- ---------- ------HVNQ
    24163  ---------- ---------- ---------- ---------- -----IQDPQ MTGYVSTRYY RAPEIMLTWQ KYDV------ ---------- ---------E VDIWSAGCIF AEML-E-GKP LFPGKD---- ---------- ------HVNQ
  1431451  ---------- ---------- ---------- ---------- -----IQDPQ MTGYVSTRYY RAPEIMLTWQ KYDV------ ---------- ---------E VDIWSAGCIF AEML-E-GKP LFPGKD---- ---------- ------HVNQ
    99398  ---------- ---------- ---------- ---------- -----VQDPQ MTGYVSTRYY RAPEIMLTWQ KYDV------ ---------- ---------E VDVWSAGCIF AEML-S-GKP LFPGKD---- ---------- ------HVNQ
   171665  ---------- ---------- ---------- ---------- -----IQDPQ MTGYVSTRYY RAPEIMLTWQ KYDV------ ---------- ---------E VDIWSAGCIF AEML-E-GKP LFPGKD---- ---------- ------HVNQ
    15876  ---------- ---------- ---------- ---------- -----EQDHQ MTGYVVTRYY RAPEVMLTWQ QYNY------ ---------- ---------A VDIWSVGCIL AEML-R-GKP LFPGKN---- ---------- ------HIDQ
    43014  ---------- ---------- ---------- -SANQPEA-- -----EGTGF MTEYVATRWY RAPEIMLTFK EYTK------ ---------- ---------A IDVWSVGCIL AEML-S-GKP LFPGRD---- ---------- ------YHHQ
    43498  ---------- ---------- ---------- -SANQPEA-- -----EGTGF MTEYVATRWY RAPEIMLTFK EYTK------ ---------- ---------A IDVWSVGCIL AEML-S-GKP LFPGRD---- ---------- ------YHHQ
  1148336  ---------- ---------- ---------- -SAASTDD-- ---NS---GF MTEYVATRWY RAPEIMLTFK EYTK------ ---------- ---------A IDVWSVGCIL AEML-S-GKP LFPGKD---- ---------- ------YHHQ
   220391  ---------- ---------- ---------- -SVKTSVP-- ---GGKEVGL MTEYVATRWY RAPEIMLSFK MYTK------ ---------- ---------A IDIWAVGCIL AELL-T-GRP LFPGRD---- ---------- ------YSHQ
   774567  ---------- ---------- ---------- -SMRSTHP-- ---DGKEAGL MTEYVATRWY RAPEIMLSFK MYTK------ ---------- ---------A IDIWAVGCIL AELL-T-GRP LFPGRD---- ---------- ------YGHQ
   138915  ---------- ---------- ---------- -SMRSTHP-- ---DGKEAGL MTEYVATRWY RAPEIMLSFK MYTK------ ---------- ---------A IDIWAVGCIL AELL-T-GRP LFPGRD---- ---------- ------YGHQ
   757677
   313819
   290180
    12232
   865310
   312015
   500478
   500460
   243933
    64065
     2938
   196733
   145976
   501440
    71512
   543038
    99402
  1431403
   342880
   417966
Consensus                                    ......... ........g. $TeYVaTRwY RAPE!ML.f. .Y...                .                  .a !DvWsvGC!l aE$l.. gkP l%pGrD.                   ...Q

           1051                                                                                                                                                           1200
     6423  LNQILHYLG- ----TPSEST LSRIGSPRA- ---------- -------QEY VRN-LPYMSK I-PFQQL--F P--------- -------RAN PDA------- ---LDLLDRM L--------- ---------- --AFDPSQRI GVEEALEHRY
   160078  LNQILHYLG- ----TPSEST LSRIGSPRA- ---------- -------QEY VRN-LPYMSK I-PFQQL--F P--------- -------RAN PDA------- ---LDLLDRM L--------- ---------- --AFDPSQRI GVEEALEHRY
   116650  LNQILHYLG- ----TPSEST LSRIGSPRA- ---------- -------QEY VRN-LPYMSK V-PFQQL--F P--------- -------RAN PDA------- ---LDLLDRM L--------- ---------- --AFDPSQRI SVEDALEHRY
   377992  LNQILHFLG- ----TPSEAT LARIGSPRA- ---------- -------QEY VRN-LPYMPK I-SFAQL--F P--------- -------RAN PDA------- ---LDLLDRL L--------- ---------- --AFDPSQRI GVEESLEHRY
   289793  LNQILHYLG- ----TPNEDT LSRIGSPRA- ---------- -------QEY VRH-LPRMPK V-PFASL--F R--------- -------NAN PDA------- ---LDLLDRM L--------- ---------- --AFDPSSRI SVEEALEHRY
   670269  LNQILHYLG- ----TPNEET LSRIGSPRA- ---------- -------QEY VRN-LPYMQK V-SFQSL--F R--------- -------NAN PDA------- ---LDLLDRM L--------- ---------- --AFDPSSRI SVEDALEHRY
    32808  LNQILHYLG- ----TPNEET LSRIGSPRA- ---------- -------QDY VRN-LPYMQK M-SFQSL--F K--------- -------NAN PDA------- ---LDLLDRM L--------- ---------- --AFDPSSRI SVEEALEHRY
   156295  LNQILHYLG- ----TPNEET LSRIGSPRA- ---------- -------QDY VRN-LPYMQK M-SFQSL--F K--------- -------NAN PDA------- ---LDLLDRM L--------- ---------- --AFDPSSRI SVEEALEHRY
    22314  LNQILHYLG- ----TPNEET LSRIGSPRA- ---------- -------QDY VRN-LPYMQK M-SFQSL--F K--------- -------NAN PDA------- ---LDLLDRM L--------- ---------- --AFDPSSRI SVEEALEHRY
   417978  LNQILHYLG- ----TPNEET LSRIGSPRA- ---------- -------QEY VRN-LPRMQK M-TFQSL--F R--------- -------NAN PDA------- ---LDLLDRL L--------- ---------- --AFDPSSRI SVEEALEHPY
   414783  LNQILHYLG- ----TPNEET LSRIGSPRA- ---------- -------QEY VRN-LPYMQK V-SFAAL--F R--------- -------NAN PDA------- ---LDLLDKM L--------- ---------- --AFDPSSRI SVETALEHRY
  1074946  LNQILHILG- ----TPNEET LSRIGSPRA- ---------- -------QEY VRN-LPFMAK K-PFPQL--F P--------- -------NAN PDA------- ---LDLLDRM L--------- ---------- --AFDPASRI TVEQALEHPY
   865350  LNQILHILG- ----TPNEET LSRIGSPRA- ---------- -------QEY VRN-LPFMPK K-PFPSL--F P--------- -------QAN PDA------- ---LDLLDRM L--------- ---------- --AFDPSSRI SVEQALEHPY
  1656563  LNQILHILG- ----TPNEET LSRIGSPRA- ---------- -------QEY VRN-LPFMPK K-TFPSL--F P--------- -------QAN PDA------- ---LDLLDRM L--------- ---------- --AFDPSSRI SVEQALEHPY
   333276  LNQILHILG- ----TPNEET LSRIGSPRA- ---------- -------QEY VRN-LPFMPK K-SFQSL--F P--------- -------NAN PDA------- ---LDLLDRM L--------- ---------- --AFDPTSRI SVEQALEHPY
   422586  LNQILHILG- ----TPNEET LSRIGSSRA- ---------- -------QEY VRN-LPYMAK K-PFPTL--F P--------- -------NAN PDA------- ---LDLLDHM L--------- ---------- --AFDPSSRI SVERALEHPY
   342887  LNQILHILG- ----TPNEET LARIGSSRA- ---------- -------QEY VRN-LPYMAK K-PFATL--F P--------- -------NAN PDA------- ---LDLLDRM L--------- ---------- --AFDPSSRI SVEEALQHPY
   386771  LNQILHILG- ----TPNEET LLRIGSSRA- ---------- -------QEY VRN-LPYMAK K-TFPTL--F P--------- -------NAN PDA------- ---LDLLDRM L--------- ---------- --AFDPSSRI SVEQALEHPY
    14976  LNQILHILG- ----TPNEET LSRIGSSRA- ---------- -------QEY VRN-LPFMAK K-PFPTL--F P--------- -------NAN PDA------- ---LDLLDRM L--------- ---------- --AFDPSSRI SVEQALEHPY
   130981  LNQILHILG- ----TPNEET LSRIGSPRA- ---------- -------QEY VRN-LPFMAK R-PFPTL--F P--------- -------TAN PDA------- ---LDLLNQM L--------- ---------- --AFDPSSRI SVESALEHPY
   435286  LNQILHILG- ----TPNEET LSRIGSPRA- ---------- -------QEY VRN-LPFMAK R-PFPSL--F P--------- -------NAN PDA------- ---LDLLNHM L--------- ---------- --AFDPSSRI SVEEALEHPY
  1457372  LNQILHILG- ----TPNEET LSRIGSPRA- ---------- -------QEY VRN-LPFMAK K-TFPSL--F P--------- -------QAN PDA------- ---LDLLDRM L--------- ---------- --AFDPSSRI SVEQALEHPY
   249807  LNQILHYLG- ----TPNEET LSRIGSPRA- ---------- -------QEY VRN-LPFMPK I-PFQRL--F P--------- -------NAN PDA------- ---LDLLDRM L--------- ---------- --AFDPTSRI SVEEALEHPY
  1142566  LNQILHYLG- ----TPNEET LSRIGSPRA- ---------- -------QEY VRN-LPFMPK I-PFQRL--F P--------- -------NAN PDA------- ---LDLLDRM L--------- ---------- --AFDPTSRI SVEEALEHPY
  1008492  LNQILHYLG- ----TPNEET LSRIGSPRA- ---------- -------QEY VRN-LPFMPK I-PFQRL--F P--------- -------NAN PDA------- ---LDLLDRM L--------- ---------- --AFDPTSRI SVEEALEHPY
   152510  LNQILHYLG- ----TPNEET LCRIGSPRA- ---------- -------QEY VRN-LPYMHK I-PFQKL--F P--------- -------NAN PDA------- ---LDLLDRM L--------- ---------- --AFDPSSRI SVEQALEHPY
   336937  LNQILHYLG- ----TPNEET LCRIGSPRA- ---------- -------QEY VRN-LPYMHK I-PFQKL--F P--------- -------NAN PDA------- ---LDLLDRM L--------- ---------- --AFDPSSRI SVEQALEHPY
  1649893  LNQILHHLG- ----TPNEET LNRIGSPRA- ---------- -------QEY VRN-LPFMPK V-SFARL--F P--------- -------NAN PDA------- ---LDLLNEM L--------- ---------- --AFDPTSRV SVETALEHRY
   371270  LTQILKLLG- ----TPNEET LSRIGSPRA- ---------- -------QDY VRN-LGYMEK I-PFRQI--F P--------- -------HAN PDA------- ---LDLLDKM L--------- ---------- --AFDPTSRI TVEEALEHPY
    82103  LNQILHYLG- ----TPNEET LSRIGSPRA- ---------- -------QDY VRN-LPYMQK I-SFQSL--F R--------- -------NAN PDA------- ---LDLLDRM L--------- ---------- --AFDPSSRI SVEEALEHRY
    42859  LNQILHVLG- ----TPDEST LQLIGSPRA- ---------- -------REY VRG-LPYMPR I-PYQQF--F P--------- -------RAN PDA------- ---LDLLEKM L--------- ---------- --AFVPAKRI TTEEALEHPY
   212507  LNQILHILG- ----TPNEET LSRIGSSRA- ---------- -------QEY VRN-LPYMAK K-PFPTL--F P--------- -------NAN PDA------- ---LDLLDHM L--------- ---------- --AFDPSSRI SVERALEHPY
   212502  LNQILHILG- ----TPNEET LSRIGSSRA- ---------- -------QEY VRN-LPYMAK K-PFPTL--F P--------- -------NAN PDA------- ---LDLLDHM L--------- ---------- --AFDPSSRI SVERALEHPY
   461664  LNQILHILG- ----TPNEET LSRIGSPRA- ---------- -------QEY VRN-LPFMAK R-PFPSL--F P--------- -------NAN PDA------- ---LDLLNHM L--------- ---------- --AFDPSSRI SVEEALEHPY
    39613  SNQILSVLG- ----TPDEDT LSRIGSQRA- ---------- -------QDY VRS-SPAQPK L-PFSKS--F P--------- -------EAN PSA------- ---SDSLDKL L--------- ---------- --AFDPDRRI TVQEALEHEY
    91135  LNQILRIIG- ----TPNEET LVRIGSRRA- ---------- -------QDY VRS-LPYMPK V-EYLKL--F Q--------G LSGNPNLSIN PLA------- ---LDLLEKM L--------- ---------- --TLDPTQRI DVNQALHHAY
   103403  LNQILYIVG- ----TPDETT LQRIGSKRA- ---------- -------QEY VRS-LPFMPK V-PFSQL--Y P--------- -------NAN PDG------- ---LDLLEKM L--------- ---------- --TLDPSQRI TAEEALEHPY
   112360  LNQILYIVG- ----TPAEET LTRIGSQRA- ---------- -------QDY VRS-LPFMPK V-PFSQL--Y P--------- -------DAN PDA------- ---LDLLDRM L--------- ---------- --CLDPAKRI TVEEALTHPY
     4266  LNQILRVLG- ----TPDEKT IENIKSPRA- ---------- -------RAY VRS-LPYMPR I-PFETL--Y P--------- -------DAS PLA------- ---LDLLSKL L--------- ---------- --TLDPKTRI TAEQALEHPY
   168486  LNQILLILG- ----TPKEST LTKIGSIRA- ---------- -------QNY VRS-LPLMRK I-DYSEI--F P--------- -------NAN PLA------- ---LDLLEKM L--------- ---------- --TLDPYQRI NVEDALNHPY
    35227  LNEILKVLG- ----TPNEHT MQSIKSSRA- ---------- -------QAY VRS-LPYMPK V-PFENF--F P--------- -------HAN PLA------- ---IDLLNRM L--------- ---------- --TLNPEDRI TVEDALQHPY
    67581  LNEILKVLG- ----TPNEHT MQSIKSSRA- ---------- -------QAY VRS-LPYMPK V-PFENF--F P--------- -------HAN PLA------- ---IDLLNRM L--------- ---------- --TLNPEDRI TVEDALQHPY
    59367  LNRILQVLG- ----TPAEHT LERIGSKNV- ---------- -------QDY IHQ-LGFIPK V-PVEL---Y P--------- -------NAN PDA------- ---LDLMEKM L--------- ---------- --AFDPIQRI TVDEALAHPY
   336175  LNQILGILG- ----TPDEET LCRVGSERA- ---------- -------QVY IRS-LTRMPK I-PFQNL--Y P--------- -------RAS PLA------- ---IDLLTKL L--------- ---------- --AFDPAKRI TVEEALAHPY
   177826  LNQILGILG- ----TPDEET LRRVGSERA- ---------- -------QVY IRS-LPHMPK I-LFQNL--Y P--------- -------RAN PLA------- ---IDLLNKL L--------- ---------- --EFDPAKRI TVEEALAHPY
   212434  LNQILGILG- ----TPDEET LRRVGSERA- ---------- -------QVY IRS-LPRMPK I-PFQNL--Y P--------- -------RAN PLA------- ---IDLLNKL L--------- ---------- --EFDPAKRI TVEEALAHPY
   164796  LNQILGILG- ----TPDEET LRRVGSERA- ---------- -------QVY IRS-LPRMPK I-PFQNL--Y P--------- -------RAN PLA------- ---IDLLNKL L--------- ---------- --EFDPAKRI TVEEALAHPY
```

```
  100890  LNQILQILG- ----TPDDAT LSRIGSERA- ---------- -------QMY IRS-LPYMPK V-SWERI--F P--------- -------NAT PLA------- ---LDLLEKM L--------- ---------- --DFDPSTRI TVEEVLAHPY
  288633  LNQILYVLG- ----TPDDHT IGRVGSERA- ---------- -------QLY IKS-LDKFEK I-PLCRL--Y P--------- -------NAS PLA------- ---LDLLEKL L--------- ---------- --TFDPTSRI TIEDALEHPY
   69432  LNQILHYLG- ----TPPEET LRRVGSPRA- ---------- -------QDY IRS-LPIKPR V-PFSTL--Y P--------- -------RAN PLA------- ---IDLLGQM L--------- ---------- --HFDPIKRI GCEQALNHPY
   60279  LNQILHYLG- ----TPSEDT LRRVGSPRA- ---------- -------QDY IRS-LPIKPR I-SFANI--Y P--------- -------KAN PLA------- ---IDLLSQM L--------- ---------- --CFDPARRI SCEQALNHPY
  166223  LNQILHYLG- ----TPSEDT LRRVGSPRA- ---------- -------QDY IRS-LPIKPR V-PFSTL--Y P--------- -------NAN PLA------- ---IDLLGQM L--------- ---------- --CFDPARRI SCEQALNHPY
 1111368  LNQILHYLG- ----TPSEDT LRRVGSPRA- ---------- -------QDY IRS-LPIKPK V-PFSTL--Y P--------- -------HAN PLA------- ---IDLLSNL L--------- ---------- --CFDPAKRI SCEQALNHPY
 7180514  LNQILHYLG- ----TPSEDT LRRVGSPRA- ---------- -------QDY IRS-LPIKPR V-PFSTL--F P--------- -------HAN PLA------- ---IDLLSQM L--------- ---------- --CFDPAKRI SCEQALNHPY
  671172  LNQILHYLG- ----TPSEDT LRRVGSPRA- ---------- -------QDY IRS-LPIKPR V-PFSTL--F P--------- -------HAN PLA------- ---IDLLSQM L--------- ---------- --CFDPAKRI SCEQALNHPY
   69595  LNQILHYLG- ----TPSEDT LRRVGSPRA- ---------- -------QDY IRS-LPIKPR V-PFSTL--F P--------- -------QAN PLA------- ---IDLLSQM L--------- ---------- --CFDPAKRM SCEQALNHPY
  188428  LNQILHYLG- ----TPSEDT LRRVGSPRA- ---------- -------QDY IRS-LPIKPR V-PFSTL--F P--------- -------QAN PLA------- ---IDLLSQM L--------- ---------- --CFDPAKRM SCEQALNHPY
  799680  LNQILHYLG- ----TPSEDT LRRVGSPRA- ---------- -------QDY IRS-LPIKPR V-PFATL--Y P--------- -------HAN PLA------- ---IDLLSQM L--------- ---------- --CFDPAKRI SCEQALNHPY
  178052  LNQILHYLG- ----TPSEDT LRRVGSPRA- ---------- -------QDY IRS-LPIKPR V-PFSTL--Y P--------- -------HAN PLA------- ---IDLLSQM L--------- ---------- --CFDPAKRI SCEQALNHPY
  129301  LNQILHYLG- ----TPSEDT LRRVGSPRA- ---------- -------QDY IRS-LPIKPR I-SFATL--Y P--------- -------RAN PLA------- ---IDLLSKM L--------- ---------- --CFDPAKRI SCEDALAHPY
  208817  LNQILHYLG- ----TPSEDT LRRVGSPRA- ---------- -------QDY IRS-LPIKPR I-SFATL--Y P--------- -------RAN PLA------- ---IDLLSKM L--------- ---------- --CFDPAKRI SCEEALDHPY
  424444  LNQILHYLG- ----TPSEDT LRRVGSPRA- ---------- -------QDY IRS-LPIKPR V-LFSTL--F P--------- -------HAN PLA------- ---IDLLSGM L--------- ---------- --CFDPAKRI SCEQALNHPY
  830405  LNQILHYLG- ----TPSEDT LRRVGSPRA- ---------- -------QDY IRS-LPIKPR V-PFSTL--F P--------- -------QAN PLA------- ---IDLLSQM L--------- ---------- --CFDPAKRM SCEQALNHPY
  892596  LNQILHYLG- ----TPSEDT LRRVGSPRA- ---------- -------QDY IRS-LPIKPR V-PFATL--Y P--------- -------HGN PLA------- ---IDLLSQM L--------- ---------- --CFDPAKRI SCEQALNHPY
  186189  LNQILHYLG- ----TPSEDT LRRVGSPRA- ---------- -------QDY IRS-LPIKPR V-PFSSL--F P--------- -------RAN PMA------- ---IDLLQQL L--------- ---------- --NFDPAKRI TCEQALKHPY
  164780  LNQILHYLG- ----TPSEDT LRRVGSPRA- ---------- -------QDY IRS-LPIKPR I-PFTTL--Y P--------- -------HAN PLA------- ---LDLLSKM L--------- ---------- --TFDPAKRI SCEQALAHPY
  167312  LNQILHYLG- ----TPSEDT LRRVGSPRA- ---------- -------QDY IRS-LPIKPR V-PFATL--F P--------- -------RAN PLA------- ---IDLLSQL L--------- ---------- --HFDPAKRI TCEQALNHPY
  119588  LNQILHYLG- ----TPSEDT LRRVGSPRA- ---------- -------QDY IRS-LPIKPR V-PFQTL--F P--------- -------RAN PLA------- ---IDLLAQL L--------- ---------- --HFDPAKRI TCEQALNHPY
  423592  LNQILHYLG- ----TPSEDT LRRVGSPRA- ---------- -------QDY IRS-LPIKPR V-PFQQL--F P--------- -------HAN PLA------- ---IDLLSKM L--------- ---------- --NFDPAKRI TCEQALQHPY
  474667  LNQILHYLG- ----TPSEDT LRRVGSPRA- ---------- -------QDY IRS-LPIRPR I-SFSTL--F P--------- -------QAN PLA------- ---IDLLSRM L--------- ---------- --CFDPARRI SCEEALNHPY
  459613  LNQILHYLG- ----TPSEDT LRRVGSPRA- ---------- -------QDY IRS-LPIRPR I-SFSTL--F P--------- -------QAN PLA------- ---IDLLSRM L--------- ---------- --CFDPARRI SCEEALNHPY
  156206  LNQILHYLG- ----TPSEET LRRVGSPRA- ---------- -------QDY IRS-LPKKPR V-PFSTL--F P--------- -------QAN PLA------- ---SDLLSQL L--------- ---------- --CFDPAKRM SCEQALGHPY
  791811  LNQILHYLG- ----TPSEET LRRVGSPRA- ---------- -------QDY IRS-LPIKPR V-PFQTL--F P--------- -------RAN PLA------- ---IDLLSQL L--------- ---------- --QFDPAKRI TCEQALKHPY
  146905  LNQILHYLG- ----TPTEDT LRRVGSPRA- ---------- -------QDY IRS-LPIKAR I-PFATL--Y P--------- -------HAN PLA------- ---VDLLTQM L--------- ---------- --TFDPAKRI SCEQALAHPY
  715329  LNQILHYLG- ----TPLEDT LRRVGSPRA- ---------- -------QDY IRS-LPIKPR V-PFQTL--F P--------- -------RAN PLA------- ---IDLLGQL L--------- ---------- --HFDPAKRI TCEQALNHPY
  511921  LNQILHYLG- ----TPSEDT LRRVGSPRA- ---------- -------QDY IRS-LPIKPR I-PFQSL--Y P--------- -------HAN PLA------- ---IDLLAQM L--------- ---------- --CFDPAKRI SCEKALEHPY
  737951  LNQILHYLG- ----TPSEDT LRRVGSPRA- ---------- -------QDY IRS-LPIKPR V-PFQTL--F P--------- -------RAN PLA------- ---IDLLSQL L--------- ---------- --HFDPAKRI TCEQALNHPY
  545584  LNQILHYLG- ----TPSEDT LRRVGSPRA- ---------- -------QDY IRS-LPIKPR I-PFQSL--Y P--------- -------HAN PLA------- ---IDLLAQM L--------- ---------- --CFDPAKRI SCEKALEHPY
  102765  LNQILHYLG- ----TPSEDT LRRVGSPRA- ---------- -------QDY IRS-LPIKPR I-PFQSL--Y P--------- -------HAN PLA------- ---IDLLSQM L--------- ---------- --CFDPAKRI SCEKALEHPY
  203555  LNQILHYLG- ----SPSEEA LERVGSLRA- ---------- -------QDY IRS-LPFKPH V-SFPKM--Y P--------- -------QAN PLA------- ---LDALSKM L--------- ---------- --CFDPTERM SCEEALEHPY
  302829  LNQILHYLG- ----TPSEDT LRRVGSPRA- ---------- -------QDY IRS-LPIKPR V-PFSTL--F P--------- -------HAN PLA------- ---IDLLSQM L--------- ---------- --CFDPAKRI SCEQALNHPY
  701651  LNQILHYLG- ----TPSEET LRRVGSPRA- ---------- -------QDY IRS-LPIKPR V-PFQTL--F P--------- -------RAN PLA------- ---IDLLSQL L--------- ---------- --QFDPAKRI TCEQALKHPY
  972433  LNQILHYLG- ----TPSEDT LRRVGSPRA- ---------- -------QDY IRS-LPIKPR V-PFATL--Y P--------- -------HAN PLA------- ---IDLLSQL L--------- ---------- --CFDPAKRI SCEQALNHPY
  931864  LNQILHYLG- ----TPSEDT LRRVGSPRA- ---------- -------QDY IRS-LPIKPR V-PFATL--Y P--------- -------HAN PLA------- ---IDLLSQL L--------- ---------- --CFDPAKRI SCEQALNHPY
  813292  WVLLLRTLY- ----AELAHL ------EFA- ---------- -------QDY IRS-LPIKPR V-PFATL--Y P--------- -------HAN PLA------- ---IDLLSQM L--------- ---------- --CFDPAKRI SCEQALNHPY
   78187  LNQILHYLG- ----TPSEDT LRRVGSPRA- ---------- -------QDY IRS-LPIKPR V-PFSTL--F P--------- -------QAN PLA------- ---IDLLSQM L--------- ---------- --CFDPAKRM SCEQALNHPY
  406308  LNQILHYLG- ----TPNEET LGRIGSPRA- ---------- -------QEY VRN-LPFMPK I-PFDRL--F P--------- -------NAN PDA------- ---LDLLDRM L--------- ---------- --AFDPTSRI SVEQALEHPY
  118382  LNQILHYLG- ----TPSEDT LRRVGSPRA- ---------- -------QDY IRS-LPIKPR V-PFSTL--F P--------- -------HAN PLA------- ---IDLLSQM L--------- ---------- --CFDPAKRI SCEQALNHPY
  100328  LNQILHYLG- ----TPSEDT LRRVGSPRA- ---------- -------QDY IRS-LPIKPR V-PFSSL--F P--------- -------RAN PMA------- ---IDLLQQL L--------- ---------- --NFDPAKRI TCEQALKHPY
 1411022  LNQILHLLG- ----TPTEDT LRRVGSPRA- ---------- -------QDY IRS-LPIKPR I-PFATL--Y P--------- -------HAN PLA------- ---IDLLGQM L--------- ---------- --NFDPAKRI SCDQALTHPY
  741167  LNQILHLLG- ----TPTEDT LRRVGSPRA- ---------- -------QDY IRS-LPIKPR I-PFATL--Y P--------- -------HAN PLA------- ---IDLLGQM L--------- ---------- --NFDPAKRI SCEAALNHPY
 1338511  LNQILQCLG- ----TPTEDA LRRIGSPRA- ---------- -------QEY IRS-LPIRAR V-PFSTL--Y R--------- -------NAN HEA------- ---LDLLGRM L--------- ---------- --AFDPARRL TCEQALAHPY
  756718  LNQILQCLG- ----TPTEDA LRRIGSPRA- ---------- -------QEY IRS-LPIRAR V-PFSTM--Y R--------- -------NAN AEA------- ---LDLLGRM L--------- ---------- --AFDPARRL TCEQALAHPY
  302653  LNHILNVLG- ----TPTEDT LRRVGSPRA- ---------- -------QDY IRS-LPIKPR Q-PWHQL--Y P--------- -------KAN PLA------- ---LDLLSKL L--------- ---------- --CFDPAKRI SCEEALNHPY
 1311110  LNQILHYHG- ----TPDDAT LRRVGSPRA- ---------- -------QEY IRS-LPFKPG V-PFNQL--Y P--------- -------NAN PVG------- ---LDLLAKL L--------- ---------- --AFDPGQRI TCDKALEHPY
  722894  LNQILHYLG- ----TPSEQT LRRVGSPRA- ---------- -------QDY IRS-LPHKAG V-PFTQL--Y P--------- -------NAN PLA------- ---LDLLSKL L--------- ---------- --SFDPSERI SCNEALEHPY
  288507  LNQILHYLG- ----TPNEQA LRRVGSPRA- ---------- -------QDY IRS-LPFKPK I-PFQQL--Y P--------- -------SAN PLA------- ---LDLLEKM L--------- ---------- --EFDPALRI TCDQALQHPY
  757539  LNQILHYLG- ----TPSEDT LRRVGSPRA- ---------- -------QDY IRS-LPIKPR I-SFANI--Y P--------- -------KAN PLA------- ---IDLLSQM L--------- ---------- --CFDPARRI SCEQALNHPY
  184184  LNQILHYLG- ----TPSEDT LRRVGSPRA- ---------- -------QDY IRS-LPIKPR V-PFSTL--Y P--------- -------HAN PLA------- ---IDLLSQM L--------- ---------- --CFDPAKRI SCEQALNHPY
  917307  LNQILHYLG- ----TPSEDT LRRVGSPRA- ---------- -------QDY IRS-LPIKPR V-PFATL--Y P--------- -------HAN PLA------- ---IDLLSQL L--------- ---------- --CFDPAKRI SCEQALNHPY
 6355211  LNQILHYLG- ----TPSEDT LRRVGSPRA- ---------- -------QDY IRS-LPIKPR V-PFSTL--F P--------- -------HAN PLA------- ---IDLLSQM L--------- ---------- --CFDPAKRI SCEQALNHPY
  249241  LNQILHYLG- ----TPSEDT LRRVGSPRA- ---------- -------QDY IRS-LPIKPR V-PFSTL--F P--------- -------QAN PLA------- ---IDLLSQM L--------- ---------- --CFDPAKRM SCEQALNHPY
  216822  LNQILHYLG- ----TPSEDT LRRVGSPRA- ---------- -------QDY IRS-LPIKPR V-PFSTL--F P--------- -------QAN PLA------- ---IDLLSQM L--------- ---------- --CFDPAKRM SCEQALNHPY
  805839  LNQILHYLG- ----TPSEDT LRRVGSPRA- ---------- -------QDY IRS-LPIKPR V-PFATL--Y P--------- -------HAN PLA------- ---IDLLSQM L--------- ---------- --CFDPAKRI SCEQALNHPY
  355172  LNQILHYLG- ----TPSEDT LRRVGSPRA- ---------- -------QDY IRS-LPIKPR V-PFSTL--F P--------- -------HAN PLA------- ---IDLLSQM L--------- ---------- --CFDPAKRI SCEQALNHPY
  302825  LNQILHYLG- ----TPSEDT LRRVGSPRA- ---------- -------QDY IRS-LPIKPR V-PFSTL--F P--------- -------HAN PLA------- ---IDLLSQM L--------- ---------- --CFDPAKRI SCEQALNHPY
  239059  LNQILHYLG- ----TPSEDT LRRVGSPRA- ---------- -------QDY IRS-LPIRPR I-SFSTL--F P--------- -------QAN PLA------- ---IDLLSRM L--------- ---------- --CFDPARRI SCEEALNHPY
  251409  LNQILHYLG- ----TPSEDT LRRVGSPRA- ---------- -------QDY IRS-LPIKPR V-PFQTL--F P--------- -------RAN PLA------- ---IDLLSQL L--------- ---------- --HFDPAKRI TCEQALNHPY
  251443  LNQILHYLG- ----TPSEDT LRRVGSPRA- ---------- -------QDY IRS-LPIKPR V-PFQTL--F P--------- -------RAN PLA------- ---IDLLSQL L--------- ---------- --HFDPAKRI TCEQALNHPY
   23484  LNQILGILG- ----TPDEET LCRVGSERA- ---------- -------QVY IRS-LTRMPK I-PFQNL--Y P--------- -------RAS PLA------- ---IDLLTKL L--------- ---------- --AFDPAKRI TVEEALAHPY
  306334  LNQILAVLG- ----TPDDAT LKRVGSQRA- ---------- -------QVY IRS-LPRMPR V-PFTTM--F P--------- -------KAT PLA------- ---LDLLDKL L--------- ---------- --QFDPAQRI TVEQALAHPY
  306351  LNQILAVLG- ----TPDDAT LKRVGSQRA- ---------- -------QVY IRS-LPRMPR V-PFTTM--F P--------- -------KAT PLA------- ---LDLLDKL L--------- ---------- --QFDPAQRI TVEQALAHPY
  148766  LKQILGTLG- ----TPDDQT LQRMGSDRA- ---------- -------QEY IKS-LPRMQK V-PFQQI--Y P--------- -------KAT PAA------- ---IDLLERL L--------- ---------- --DLDHTRRI TAQEALAHPY
   84676  LKQILGTLG- ----TPDDQT LQRMGSDRA- ---------- -------QEY IKS-LPRMQK V-PFQQI--Y P--------- -------KAT PAA------- ---IDLLERL L--------- ---------- --DLDHTRRI TAQEALAHPY
   74082  LNQILHYLG- ----TPNEDT LSRIGSPRA- ---------- -------QEY VRH-LPRMPK V-PFASL--F R--------- -------NAN PDA------- ---LDLLDRM L--------- ---------- --AFDPSSRI SVEEALEHRY
   97664  LNQILHYLG- ----TPSEET LARIGSPRA- ---------- -------QDY VRN-LPYMQK I-SFQSL--F R--------- -------NAN PDA------- ---LDLLDRM L--------- ---------- --AFDPSQRI SVEEALEHRY
   97676  LNQILHYLG- ----TPSEET LARIGSPRA- ---------- -------QDY VRN-LPYMQK I-SFQSL--F R--------- -------NAN PDA------- ---LDLLDRM L--------- ---------- --AFDPSQRI SVEEALEHRY
  297954  LTQILKLLG- ----TPNEET LSRIGSPRA- ---------- -------QDY VRN-LGYMEK I-PFRQI--F P--------- -------HAN PDA------- ---LDLLDKM L--------- ---------- --AFDPTSRI TVEEALEHPY
  323749  LNQILHILG- ----TPNEET LSRIGSPRA- ---------- -------QEY VRN-LPFMPK K-SFQSL--F P--------- -------NAN PDA------- ---LDLLDRM L--------- ---------- --AFDPTSRI SVEQALEHPY
 1926181  LNQILHILG- ----TPNEET LSRIGSPRA- ---------- -------QEY VRN-LPFMPK K-PFPSL--F P--------- -------QAN PDA------- ---LDLLDRM L--------- ---------- --AFDPSSRI SVEQALEHPY
 1457364  LNQILHILG- ----TPNEET LSRIGSPRA- ---------- -------QEY VRN-LPFMPK K-TFPSL--F P--------- -------QAN PDA------- ---LDLLDRM L--------- ---------- --AFDPSSRI SVEQALEHPY
   78435  LNQILHILG- ----TPNEET LLRIGSSRA- ---------- -------QEY VRN-LPYMAK K-TFPTL--F P--------- -------NAN PDA------- ---LDLLDRM L--------- ---------- --AFDPSSRI SVEQALEHPY
 1457361  LNQILHILG- ----TPNEET LSRIGSPRA- ---------- -------QEY VRN-LPFMPK K-TFPSL--F P--------- -------QAN PDA------- ---LDLLDRM L--------- ---------- --AFDPSSRI SVEQALEHPY
 1328540  LNQILHYHG- ----TPDDAT LRRVGSPRA- ---------- -------QEY IRS-LPFKPG V-PFNQL--Y P--------- -------NAN PVG------- ---LDLLAKL L--------- ---------- --AFDPGQRI TCDKALEHPY
 1364342  LNQILHYHG- ----TPDDAT LRRVGSPRA- ---------- -------QEY IRS-LPFKPG V-PFNQL--Y P--------- -------NAN PVG------- ---LDLLAKL L--------- ---------- --AFDPGQRI TCDKALEHPY
  350087  LNKILNYLG- ----TPTEET LRRVGSPRA- ---------- -------QDY IRS-LPIKPR I-HFKTL--Y P--------- -------TAS PHA------- ---LDLLQRL L--------- ---------- --AFDPARRI NCEEALAHPY
  288508  LNQILHYLG- ----TPNEQA LRRVGSPRA- ---------- -------QDY IRS-LPFKPK I-PFQQL--Y P--------- -------SAN PLA------- ---LDLLEKM L--------- ---------- --EFDPALRI TCDQALQHPY
  283473  LNQILHYLG- ----TPLEDT LRRVGSPRA- ---------- -------QDY IRS-LPIKPR V-PFQTL--F P--------- -------RAN PLA------- ---IDLLGQL L--------- ---------- --HFDPAKRI TCEQALNHPY
  734869  LNQILQCLG- ----TPTEDA LRRIGSPRA- ---------- -------QEY IRS-LPIRAR V-PFSTM--Y R--------- -------NAN AEA------- ---LDLLGRM L--------- ---------- --AFDPARRL TCEQALAHPY
    9394  ITEIVKVLG- ----TPSIDI IKRYGSNIA- ---------- -------FEF FTSPKPQYQA V-LWSNV--Y P--------- -------YAS KVA------- ---VDLIGRL I--------- ---------- --CWDSSRRL NVEKALEHAF
  167564  STEILKVLG- ----TPSREI ILRYGSSAA- ---------- -------WNL FSPPSPQFQP M-AWSDV--Y P--------- -------FAS ENA------- ---HDLMSKL I--------- ---------- --CWDSNERY NVHELISHRF
  113741  INEILKVLG- ----TPPRDV FTRYGSHKA- ---------- -------CDF FSPPKPQYRA I-PWSHV--Y P--------- -------YAS ELA------- ---HDLLENL L--------- ---------- --DWDPELRL EIQQCINHPY
    6575  INEILKVLG- ----TPPRDV FTRYGSHKA- ---------- -------CDF FSPPKPQYRA I-PWSHV--Y P--------- -------YAS ELA------- ---HDLLENL L--------- ---------- --DWDPELRL EIQQCINHPY
   30703  ISEICKVLG- ----TPSRNV ILGYNSTVA- ---------- -------WDY FASSKPQFQK Q-EWSNV--Y P--------- -------HAS VLA------- ---HDLLSNL L--------- ---------- --CWKVEKRY TVEQCLDHPL
   65523  ISEICKVLG- ----TPSRNV ILGYNSTVA- ---------- -------WDY FASSKPQFQK Q-EWSNV--Y P--------- -------HAS VLA------- ---HDLLSNL L--------- ---------- --CWKVEKRY TVEQCLDHPL
    5078  ITEICKVLG- ----TPSRKV ILSYESAAA- ---------- -------WDF FSTTRPSFQK K-DLNAL--F P--------- -------NAN TDA------- ---ITILSGL L--------- ---------- --CWDQKSRL TVGAAIESPF
   54997  ITEICKVLG- ----TPSRKV ILSYESAAA- ---------- -------WDF FSTTRPSFQK K-DLNAL--F P--------- -------NAN TDA------- ---ITILSGS L--------- ---------- --CWDQKSRL TVGAAIESPF
  114746  VVEILKVLG- ----TPDKDT ILNYGSTVA- ---------- -------MEI FASPKPQYAK M-AWKKI--Y P--------- -------FAS DEG------- ---LDLIERL L--------- ---------- --KWDPEKRL TCNQVLEHPF
  238776  INEILKVLG- ----TPNREI INKYGWR--- ---------- ---------- YLNTRTRYEK I-EFDKI--Y P--------- -------FAS GYS------- ---IHMIDNL L--------- ---------- --CWDPYERL DVNEALNHPF
   71410  LNKIFDVLG- ----SPEEYI MQKIGSERA- ---------- -------QAY VRS-LPFKKT V-PFRKL--M PDADPQ---- ---------- --A------- ---LDLLNKI L--------- ---------- --TFDPSERI SVLQALEHPW
  676425  LNKILEVLG- ----TPENEI IERMASPKA- ---------- -------RAY IRS-LPWKKG T-PLSKV--L PGADPQ---- ---------- --A------- ---LDLLSKM L--------- ---------- --TFDPAARI TVSQALEHPW
  676527  LNKILEVLG- ----TPENEI IERMASPKA- ---------- -------RAY IRS-LPWKKG T-PLSKV--L PGADPQ---- ---------- --A------- ---LDLLSKM L--------- ---------- --TFDPAARI TVSQALEHPW
  152706  LNKILDVLG- ----TPEESA INRIGSEKA- ---------- -------QAY VRS-LPFKKP V-PLKKL--L PAADLQ---- ---------- --A------- ---LDLLEKM I--------- ---------- --SFDPSCRI SVPDALAHPW
   88536  LNKILDVLG- ----TPEESA INRIGSEKA- ---------- -------QAY VRS-LPFKKP V-PLKKL--L PAADLQ---- ---------- --A------- ---LDLLEKM I--------- ---------- --SFDPSCRI SVPDALAHPW
  122893  LNKILEVLG- ----SPDDKV VTRIANERA- ---------- -------QNY VRS-LPFKKA I-PFRKL--I PMADPQ---- ---------- --A------- ---LDLLSRM L--------- ---------- --AFDPAQRI RVPEALEHPW
  151264  LNKILEVLG- ----SPDEQV MERIGSEKA- ---------- -------RAY VRS-LPFKKS Q-PMKRV--V PTADAE---- ---------- --A------- ---QDFLQKL T--------- ---------- --SFDPEGRP SAAGALAHPW
   67588  LLIIHKTPG- ----TPDANV IRKIGSPKA- ---------- -------QAY VRS-LPIKRV V-PFRKV--I PKGDAQ---- ---------- --A------- ---LDLLAHM L--------- ---------- --CFDPSKRI SVPGALEHAW
  153824  LNQILNILG- ----TPEERS IQRIGSEKA- ---------- -------QAY IRS-LPLKKQ I-PFVKL--Y PHADPL---- ---------- --A------- ---LDLLAKM V--------- ---------- --AFDPSDRI SVIEALEHPY
  103584  LNQILNILG- ----TPEERS IQRIGSEKA- ---------- -------QAY IRS-LPLKKQ I-PFVKL--Y PHADPL---- ---------- --A------- ---LDLLAKM V--------- ---------- --AFDPSDRI SVIEALEHPY
  118819  LNKIFDVLG- ----SPEEYI MQKIGSERA- ---------- -------QAY VRS-LPFKKT V-PFRKL--M PDADPQGRKP SSSPLYNTQP FPA------- ---LDLLNKI L--------- ---------- --TFDPSERI SVLQALEHPW
 7227248  LNKIFAILG- ----TPDVNV IRKIGSPKA- ---------- -------QAY VRS-LPVKRV V-PFRKV--V PKGDPQ---- ---------- --A------- ---LELLAKM L--------- ---------- --SFDPSERI TVPDALAHSW
  240796  LNKIFAVLG- ----SPNVNV IRKIGSPKA- ---------- -------QAY VRS-LPIRKV V-PFRKV--I PKADPQ---- ---------- --A------- ---LDLLGKM L--------- ---------- --CFDPTERI TVPGALEHPW
  366893  LNKIFAVLG- ----TPDANV IRKIGSPKA- ---------- -------QAY VRS-LPIKRV V-PFRKV--I PKGDAQ---- ---------- --A------- ---LDLLAHM L--------- ---------- --CFDPSKRI SVPGALEHAW
   79368  LNKIFAVLGE ACRCTPDADV IRKIGSPKAR MHAAISPSVR LIALPQAQAY VRS-LPTKKT V-PFKKI--I PKGDPQ---- ---------- --A------- ---LELLGKM L--------- ---------- --CFDPTERV RVPGALEHPW
  119618  LNKILDVLG- ----SPDERV MKKIGSQKA- ---------- -------QAY VRS-LPLKKK V-HFRHI--I PTADVQ---- ---------- --A------- ---LDLLEKM L--------- ---------- --AFDPEARI TVPEALEHPW
  500495  LNKVLDVLG- ----TPDESV IRKVGSDKA- ---------- -------QAY VRS-LPFKKA V-PLRKI--L PTADPQ---- ---------- --A------- ---LDLLSKM L--------- ---------- --AFDPSDRI TVPEALEHPW
  449465  LNKVLDVLG- ----TPDESV IRKIGSDKA- ---------- -------QAY VRS-LPSKKS V-PLRKI--L PTADPQ---- ---------- --A------- ---LDLLSKM L--------- ---------- --AFDPSDRI TVPEALEHPW
  509502  LNKILNVLG- ----TPDDAV IRKIGSEKA- ---------- -------QAY VRS-LPIKKS V-PFKKI--I PHADHQ---- ---------- --A------- ---LDLLKKM L--------- ---------- --TFDPDERI TVPEALEHPW
  964561  LNKILDVLG- ----TPDDNV IHRIGSEKA- ---------- -------QTY IRS-LPFKPA I-PLSKL--L PVADIQ---- ---------- --A------- ---LDLLKKM I--------- ---------- --AFDPSCRI TVRDALSHPW
  261448  VNKILDVLG- ----TPGESV IHRIGSDKA- ---------- -------QAY IRS-LPVKEG V-PLRKL--L PTADLQ---- ---------- --A------- ---LDLLEKM I--------- ---------- --TFDPTSRI AVLDALSHPW
 1150169  LNKILEVLG- ----TPEESV IHRIGSDKA- ---------- -------QAY VRS-LPYKKA V-PLRRL--L PAADFQ---- ---------- --A------- ---LDLLSKM L--------- ---------- --SFDPVTRI TISEALAHPW
  152363  LNKILDILG- ----SPDETI IQKIGSDKA- ---------- -------QAY VRS-LPFRRT V-PFRRV--L PGSDPL---- ---------- --A------- ---LDLMAKM L--------- ---------- --SFDPSTRV TVLESLEHPW
  379740  LNKILDILG- ----SPDETI IQKIGSDKA- ---------- -------QAY VRS-LPFRRT V-PFRRV--L PGSDPL---- ---------- --A------- ---LDLMAKM L--------- ---------- --SFDPSTRV TVLESLEHPW
  337664  LNKILDILG- ----SPDETI IQKIGSDKA- ---------- -------QAY VRS-LPFRRT V-PFRRV--L PGSDPL---- ---------- --A------- ---LDLMAKM L--------- ---------- --SFDPSTRV TVLESLEHPW
  434295  LNKILDILG- ----SPDETI IQKIGSDKA- ---------- -------QAY VRS-LPFRRT V-PFRRV--L PGSDPL---- ---------- --A------- ---LDLMAKM L--------- ---------- --SFDPSTRV TVLESLEHPW
  517048  LNKILEVLG- ----SPENEI IERMASPKA- ---------- -------RAY IRS-LPWKKG T-PLSKV--L PGADPQ---- ---------- --A------- ---LDLLSKM L--------- ---------- --TFDPAARI TVSQALEHPW
  731201  LNKILEVLG- ----SPENEI IERMASPKA- ---------- -------RAY IRS-LPWKKG T-PLSKV--L PGADPQ---- ---------- --A------- ---LDLLSKM L--------- ---------- --TFDPAARI TVSQALEHPW
  768698  LNKILEVLG- ----SPENEI IERMASPKA- ---------- -------RAY IRS-LPWKKG T-PLSKV--L PGADPQ---- ---------- --A------- ---LDLLSKM L--------- ---------- --TFDPAARI TVSQALEHPW
  517043  LNKILEVLG- ----SPENEI IERMASPKA- ---------- -------RAY IRS-LPWKKG T-PLSKV--L PGADPQ---- ---------- --A------- ---LDLLSKM L--------- ---------- --TFDPAARI TVSQALEHPW
  740533  LNKILEVLG- ----SPENEI IERMASPKV- ----------            ---------- --DMSPL--L -SCPSD---- ---------- --A------- ---LDLLSKM L--------- ---------- --TFDPAARI TVSQALEHPW
  178741  LNKILDIVG- ----TPEDDV ISRIASPKA- ---------- -------AAY LRS-LPYKKR V-PFERL--I PTADPQ---- ---------- --A------- ---LDLLTKL L--------- ---------- --AFDPAARI TVREALEHPW
   54225  ARAHRNRIG- ----TPEDDV ISRIASPKA- ---------- -------AAY LRS-LPYKKR V-PFERL--I PTADPQ---- ---------- --A------- ---LDLLTKL L--------- ---------- --AFDPAARI TVREALEHPW
  163441  LNKILDVLG- ----TPDNEV ISRIASSKA- ---------- -------AAY LRS-LPYKKT V-PFEKL--I PTADPQ---- ---------- --A------- ---LDLLSKL L--------- ---------- --TFDPAARI TVREALEHPW
  327966  LNKILDVVG- ----TPDNEV VTRIASPKA- ---------- -------QAY LRS-LPYKKR V-PFEKL--I PTADPQ---- ---------- --A------- ---VDLLTKL L--------- ---------- --VFDPASRI TVQQALEHPW
  722194  LNKILDVLG- ----TPDDEV IRRVASQKA- ---------- -------AAY VRS-LPIRKA V-SFSKV--L PVADLQ---- ---------- --A------- ---TDLLTKI L--------- ---------- --AFDPASRF SVVEALEHPW
  128404  IFQILDVLG- ----TPDDEV IKRIASDRA- ---------- -------QAY LRS-LPYKKM V-PYLKI--M PNADHQ---- ---------- --A------- ---INLLAKM L--------- ---------- --TFDPAARI TVADALEHPW
  167758  LNKILEVLG- ----SPDEQV MERIGSEKA- ---------- -------RAY VRS-LPFKKS Q-PMKRV--V PTADAE---- ---------- --A------- ---QDFLQKL T--------- ---------- --SFDPEGRP SAAGALAHPW
   26908  LNKILEVLG- ----SPDEQV MERIGSEKA- ---------- -------RAY VRS-LPFKKS Q-PMKRV--V PTADAE---- ---------- --A------- ---QDFLQKL T--------- ---------- --SFDPEGRP SAAGALAHPW
  987811  LNKILDVLG- ----TPAESV IQRIGSEKA- ---------- -------QTY IRS-LPIKKV Q-PFKKL--V PAADSN---- ---------- --A------- ---IDLLTKM L--------- ---------- --YFDPSMRI SVEDALAHPW
  913595  LNKILDVLG- ----TPAESV IQRIGSEKA- ---------- -------QTY IRS-LPIKKV Q-PFKKL--V PAADSN---- ---------- --A------- ---IDLLTKM L--------- ---------- --YFDPSMRI SVEDALAHPW
```

```
 922124  LNKILDVLG- ----TPAESV IQRIGSEKA- ---------- -------QTY IRS-LPIKKV Q-PFKKL--V PAADSN---- ---------- --A------- ---IDLLTKM L--------- ---------- --YFDPSMRI SVEDALAHPW
 319286  LNLILQTLG- ----MPDDST LTRIGSEKA- ---------- -------CAY IRS-VPTYEK V-PFEKL--Y PDADTD---- ---------- --A------- ---LDLLEKL L--------- ---------- --RFDPAERI SVAEAIRHPY
 332677  INLILAALG- ----SPDEDT LTKVGSEKA- ---------- -------CAY MRT-LPHYDP V-DFSTI--L PDSDPD---- ---------- --A------- ---LDLLAQL L--------- ---------- --TFDPSSRL TVPQSLTHPY
  75030  VNKILDVLG- ----TPSERV LQSISSERA- ---------- -------QAY VRS-LPIKKP V-PLVQL--M PKANPD---- ---------- --A------- ---LDLLKKM I--------- ---------- --SLDPSDRP SVAEALQHPW
 740342  VNKILDVLG- ----TPSERV LQSISSERA- ---------- -------QAY VRS-LPIKKP V-PLVQL--M PKANPD---- ---------- --A------- ---LDLLKKM I--------- ---------- --SLDPSDRP SVAEALQHPW
 769030  VNKILDVLG- ----TPSERV LQSISSERA- ---------- -------QAY VRS-LPIKKP V-PLVQL--M PKANPD---- ---------- --A------- ---LDLLKKM I--------- ---------- --SLDPSDRP SVAEALQHPW
 493654  LNLIINTLG- ----TPSDET IRKIGSDKA- ---------- -------REY VRT-LPFKLP Q-SLPKL--F PKTDNMG--- ---------- ---------- ---RDLIHKF L--------- ---------- --RFDPDERI TVNEALAHPF
  12113  LNLIINTLG- ----TPSDDT IRKIGSDKA- ---------- -------REY VRN-LPFKLP Q-SLPKL--F PKTDNMG--- ---------- ---------- ---RDLIHKF L--------- ---------- --RFDPDERI TVNEALAHPF
 115011  LNLIINTLG- ----TPPDDT IKKIGSDKA- ---------- -------REY VRN-LPFKLP Q-SLPKL--F PRTDNMG--- ---------- ---------- ---RDLIHRF L--------- ---------- --RFDPDERI SVNEALAHPF
 192400  LNLIINTLG- ----TPSDET IRKIGSDKA- ---------- -------REY VRT-LPFKLP Q-SLPKL--F PKTDNMGESP DGAIGADTNC CIG------- ---RDLIHKF L--------- ---------- --RFDPDERI TVNEALAHPF
 543665  LNLIINTLG- ----TPSDDT IRKIGSDKA- ---------- -------REY VRN-LPFKLP Q-SLPKL--F PKTDNMG--- ---------- ---------- ---RDLIHKF L--------- ---------- --RFDPDERI TVNEALAHPF
  91520  LNKILEVLG- ----TPDESI YAQVKSEKA- ---------- -------VAY VRS-LPVKRK V-PLRKI--I PSADAQ---- ---------- --A------- ---LDLLTKM L--------- ---------- --SFDPATRI SVTEALQHPW
 127212  LNKILEVLG- ----TPDESI YAQVKSEKA- ---------- -------VAY VRS-LPVKRK V-PLRKI--I PSADAQ---- ---------- --A------- ---LDLLTKM L--------- ---------- --SFDPATRI SVTEALQHPW
 438879  LNKILDVLG- ----TPRDEV IKKIGSEKA- ---------- -------QAY VRS-LPIKKT V-PLKKI--I PHADPL---- ---------- --A------- ---LDLLNKM L--------- ---------- --SFDPTDRI TVVGALEHPW
 174304  LNKILDVLG- ----TPRDEV IKKIGSEKA- ---------- -------QAY VRS-LPIKKT V-PLKKI--I PHADPL---- ---------- --A------- ---LDLLNKM L--------- ---------- --SFDPTDRI TVVGALEHPW
 174289  LNKILDVLG- ----TPRDEV IKKIGSEKA- ---------- -------QAY VRS-LPIKKT V-PLKKI--I PHADPL---- ---------- --A------- ---LDLLNKM L--------- ---------- --SFDPTDRI TVVGALEHPW
 244084  LNKILEVLG- ----TPEESV IHRIGSDKA- ---------- -------QAY VRS-LPYKKA V-PLRRL--L PAADFQ---- ---------- --A------- ---LDLLSKM L--------- ---------- --SFDPVTRI TISEALAHPW
 123741  LNKILEVLG- ----SPDDKV VTRIANERA- ---------- -------QNY VRS-LPFKKA I-PFRKL--I PMADPQ---- ---------- --A------- ---LDLLSRM L--------- ---------- --AFDPAQRI RVPEALEHPW
 172164  LNKILEVLG- ----SPDDKV VTRIANERA- ---------- -------QNY VRS-LPFKKA I-PFRKL--I PMADPQ---- ---------- --A------- ---LDLLSRM L--------- ---------- --AFDPAQRI RVPEALEHPW
  40528  LNKILDVLG- ----TPDDEV IKRIASDRA- ---------- -------QAY LRS-LPYKKM V-PYLKI--M PNADHQ---- ---------- --A------- ---INLLAKM L--------- ---------- --TFDPAARI TVADALEHPW
  39781  LNKILDVLG- ----TPDDEV IKRIASDRA- ---------- -------QAY LRS-LPYKKM V-PYLKI--M PNADHQ---- ---------- --A------- ---INLLAKM L--------- ---------- --TFDPAARI TVADALEHPW
1155690  VNKILEILG- ----TPSEKV LQAISSERA- ---------- -------QAY VRS-LPIKKP V-HLATL--M GRADFD---- ---------- --A------- ---LDLLRKM L--------- ---------- --SLDPNDRP TVMQALEHRW
 247448  INLILAALG- ----SPDEDT LTKVGSEKA- ---------- -------CAY MRT-LPHYDP V-DFSTI--L PDSDPD---- ---------- --A------- ---LDLLAQL L--------- ---------- --TFDPSSRL TVPQSLTHPY
 269975  IARINNVLG- ----SPDAKT IAKVGSERA- ---------- -------RTY VES-LPKMPA V-PFAKL--Y PSASPL---- ---------- --A------- ---VDLLSKL L--------- ---------- --CWDPEDRI SAEEALQHPW
 246067  IARINNVLG- ----SPDAKT IAKVGSERA- ---------- -------RTY VES-LPKMPA V-PFAKL--Y PSASPL---- ---------- --A------- ---VDLLSKL L--------- ---------- --CWDPEDRI SAEEALQHPW
  44396  IARINNVLG- ----SPDAKT IAKVGSERA- ---------- -------RTY VES-LPKMPA V-PFAKL--Y PSASPL---- ---------- --A------- ---VDLLSKL L--------- ---------- --CWDPEDRI SAEEALQHPW
 111248  LTLILDVLG- ----TPTMED YYGIKSRRA- ---------- -------REY IRS-LPFKKK V-PFRTL--F PKTSDL---- ---------- --A------- ---LDLLEKL L--------- ---------- --AFNPVKRI TVEEALKHPY
  54538  LTLILDVLG- ----TPTLDE FYAITTRRS- ---------- -------RDY IRA-LPFRKR R-PFATL--F PKANPL---- ---------- --A------- ---VDFLTKS L--------- ---------- --TFDPKKRI TVEDALAHPY
1341438  LSIILDVLG- ----TPSLDD FYAISSSRS- ---------- -------REY IRA-LPFRKK K-PLGQL--F PGANPQ---- ---------- --A------- ---IDLMEKC L--------- ---------- --TFSPKRRI DVEAALAHPY
1267921  LSIILDVLG- ----TPSLDD FYAISSSRS- ---------- -------REY IRA-LPFRKK K-PLGQL--F PGANPQ---- ---------- --A------- ---IDLMEKC L--------- ---------- --TFSPKRRI DVEAALAHPY
 692594  FSIITELLG- ----TPPDDV IQTICSENT- ---------- -------LRF VQS-LPRRER Q-PLSNK--F NNADPL---- ---------- --A------- ---IDLLESM L--------- ---------- --VFDPKKRV RAESALAHPY
 384872  LNQILHYLG- ----TPNEET LSRIGSPRA- ---------- -------QDY VRN-LPYMQK I-SFQSL--F RNANPD---- ---------- --A------- ---LDLLDRM L--------- ---------- --AFDPSSRI SVEEALEHRY
 419622  LNQILHYLG- ----TPSEET LARIGSPRA- ---------- -------QDY VRN-LPYMQK I-SFQSL--F RNANPD---- ---------- --A------- ---LDLLDRM L--------- ---------- --AFDPSQRI SVEEALEHRY
 328081  LNKILNYLG- ----TPTEET LRRVGSPRA- ---------- -------QDY IRS-LPIKPR I-HFKTL--Y PTASPH---- ---------- --A------- ---LDLLQRL L--------- ---------- --AFDPARRI NCEEALAHPY
  61803  LKLITQKIG- ----TPGNDD I-QFVTSTK- ---------- ------AKSF MLS-QRYQKR C-DFAQL--Y PSASSD---- ---------- --A------- ---IDLLNRM L--------- ---------- --VFNPGKRI GVNDALAHPY
  40664  LKLITQKIG- ----TPGNDD I-QFVTSTK- ---------- ------AKSF MLS-QRYQKR C-DFAQL--Y PSASSD---- ---------- --A------- ---IDLLNRM L--------- ---------- --VFNPGKRI GVNDALAHPY
 105104  IRIITDKIG- ----TPSDED LLSFVKSTK- ---------- ------AKRF MKD-QGFKPP Q-DFKRT--Y PNADPA---- ---------- --A------- ---LDLLQRM L--------- ---------- --TFNPAKRI TVQDALKHPY
 147180  LRLICQVLG- ----SPSNEE M-AFMSEGS- ---------- ------ANRY LRE-LEFRPK K-PWNTVKGL ENAPPE---- ---------- --A------- ---LDLLSKM L--------- ---------- --TFSPKDRI TALEALKHPY
 148245  LQVIIEILG- ----TPSASE I-DFISNPS- ---------- ------ARRA IQS-FGRHRK R-SLIKMSQF HGANPL---- ---------- --A------- ---IDLLQRM L--------- ---------- --VFDPRGRI TIDEALEHPY
 146852  LTLISDLLG- ----KPTQED L-AFVTSEK- ---------- ------ARRF MER-QPEKAR V-PFENL--Y PRANPN---- ---------- --A------- ---VNLLERM L--------- ---------- --VFDPSRRI TVVEALAHPY
  33818  LTLISDLLG- ----KPTQED L-AFVTSEK- ---------- ------ARRF MER-QPEKAR V-PFENL--Y PRANPN---- ---------- --A------- ---VNLLERM L--------- ---------- --VFDPSRRI TVVEALAHPY
 116469  LTLISDLLG- ----KPTQED L-AFVTSEK- ---------- ------ARRF MER-QPEKAR V-PFENL--Y PRANPN---- ---------- --A------- ---VNLLERM L--------- ---------- --VFDPSRRI TVVEALAHPY
 503885  LTQIMSVLG- ----TPSDEV LDRIIKPTP- ---------- ------HDPY LKS-IKKMPK I-SLADR--F PGANPK---- ---------- --A------- ---IDLLEKL L--------- ---------- --VWCPSDRL TAEQVLAHPY
 503914  LTQIMSVLG- ----TPSDEQ TPSRIRK--- ---------- ---------Y LKS-IKKMPK I-SLADR--F PGANPK---- ---------- --A------- ---IDLLEKL L--------- ---------- --VWCPSDRL TAEQVLAHPY
6817382  ---------- ------DVNV IRKIGSPKA- ---------- -------QAY VRS-LPVKRV V-PFRKV--V P--------- --------KGD PQA------- ---LELLAKM L--------- ---------- --SFDPSERI TVPDALAHSW
 145980  LNQILHYLG- ----TPS-ES TLSR------ ---I------ --GSPRAQEY V-RNLPYMSK I-PFQQL--- ----F----- ------PRAN PDA------- ---LDLLDRM L--------- ---------- --AFDPSQRI GVEEALEHRY
 328170  LNQILHYLG- ----TPN-ED TLSR------ ---I------ --GSPRAQEY V-RHLPRMPK V-PFASL--- ----F----- ------RNAN PDA------- ---LDLLDRM L--------- ---------- --AFDPSSRI SVEEALEHRY
 691008  LNQILHYLG- ----TPN-EE TLSR------ ---I------ --GSPRAQEY V-RNLPYMQK V-SFQSL--- ----F----- ------RNAN PDA------- ---LDLLDRM L--------- ---------- --AFDPSSRI SVEDALEHRY
  73316  LNQILHVLG- ----TPD-ES TLQL------ ---I------ --GSPRAREY V-RGLPYMPR I-PYQQF--- ----F----- ------PRAN PDA------- ---LDLLEKM L--------- ---------- --AFVPAKRI TTEEALEHPY
1219281  LNQILHHLG- ----TPN-EE TLNR------ ---I------ --GSPRAQEY V-RNLPFMPK V-SFARL--- ----F----- ------PNAN PDA------- ---LDLLNEM L--------- ---------- --AFDPTSRV SVETALEHRY
 422525  LNQILHILG- ----TPD-DE TLRR------ ---I------ --GSDRALLY I-RSLERMPK I-PFSHL--- ----F----- ------PRAD PQA------- ---LDLLEKL L--------- ---------- --KFDPAARI TVEDALSHPY
 212435  LNQILGILG- ----TPD-EE TLRR------ ---V------ --GSERAQVY I-RSLPRMPK I-PFQNL--- ----Y----- ------PRAN PLA------- ---IDLLNKL L--------- ---------- --EFDPAKRI TVEEALKHPY
 187307  LNQILHYLG- ----TPS-ED TLRR------ ---V------ --GSPRAQDY I-RSLPIKPR I-SFANI--- ----Y----- ------PKAN PLA------- ---IDLLSQM L--------- ---------- --CFDPARRI SCEQALNHPY
 146240  LNQILHYLG- ----TPS-ED TLRR------ ---------- ------AQDY I-RSLPIKPR V-PFATL--- ----F----- ------PRAN PLA------- ---IDLLSQL L--------- ---------- --HFDPAKRI TCEQALNHPY
 156221  LNQILHYLG- ----TPS-EE TLRR------ ---V------ --GSPRAQDY I-RSLPKKPR V-PFSTL--- ----F----- ------PQAN PLA------- ---SDLLSQL L--------- ---------- --CFDPAKRM SCEQALGHPY
  48865  LNQILHYLG- ----TPS-ED TLRR------ ---V------ --GSPRAQDY I-RSLPIKPR I-SFATL--- ----Y----- ------PRAN PLA------- ---IDLLSKM L--------- ---------- --CFDPAKRI SCEEALDHPY
 226510  LNQILHYLG- ----TPS-EE TLRR------ ---V------ --GSPRAQDY I-RSLPIKPR V-PFQTL--- ----F----- ------PRAN PLA------- ---IDLLSQL L--------- ---------- --QFDPAKRI TCEQALKHPY
 821431  LNQILHYLG- ----TPS-ED TLRR------ ---V------ --GSPRVVYF S-PRHRLKTI FGPSPSN--- ----L----- ------ESPS RPS------- ---IDLLSQM L--------- ---------- --CFDPAKRM SCEQALNHPY
  12784  LNHILNVLG- ----TPT-ED TLRR------ ---V------ --GSPRAQE- ---SLPIKPR Q-PWHQL--- ----Y----- ------PKAN PLA------- ---LDLLSKL L--------- ---------- --CFDPAKRI SCEEALNHPY
 157692  LNQILHFLG- ----TPS-EA TLAR------ ---I------ --GSPRAQEY V-RNLPYMPK I-SFAQL--- ----F----- ------PDSR DAS------- ---NDPRRGR T--------- ---------- --IPPIRSCA TVSSAGTRPG
  47352  IRIITDKIG- ----TPSDED LLSF------ ---V------ --KSTKARKF M-KDQGFKPP Q-DFKRT--- ----Y----- ------PNAD PAA------- ---IDLLQRM L--------- ---------- --TFNPAKRI TVQDALKHPY
 136580  LTLILDVLG- ----TPTMED YYGI------ ---------- --KSRRAREY I-RSLPFKKK I-PWKVM--- ----F----- ------PKTS DLA------- ---LDLLDKL L--------- ---------- --AFNPVKRI TVEEALRHPY
 422908  LTLILDVLG- ----TPTMED YYGI------ ---------- --KSRRAREY I-RSLPFKKK I-PWKVM--- ----F----- ------PKTS DLA------- ---LDLLEKL L--------- ---------- --AFNPVKRI TVEEALKHPY
  71188  LTLILDVLG- ----TPTMED YYGI------ ---------- --KSRRAREY I-RSLPFKKK I-PWKVM--- ----F----- ------PKTS DLA------- ---LDLLERL L--------- ---------- --AFNPVKRI TVEEALKHPY
2049832  LTLILDVLG- ----TPTMED YYGI------ ---------- --KSRRAREY I-RSLPFKKK V-PFRTL--- ----F----- ------PKTS DLA------- ---LDLLEKL L--------- ---------- --AFNPVKRI TVEEALKHPY
2023270  LTLILDVLG- ----TPTMED YYGI------ ---------- --KSRRAREY I-RSLPFKKK V-PFRTL--- ----F----- ------PKTS DLA------- ---LDLLEKL L--------- ---------- --AFNPVKRI TVEEALKHPY
  62157  LTLILDVLG- ----TPTMED YYGI------ ---------- --KSRRAREY I-RSLPFKKK I-PWKAM--- ----F----- ------PKTS DLA------- ---LDLLDKL L--------- ---------- --AFNPAKRI TVEEALRHPY
 117327  LTLILDVLG- ----TPTMED YYGI------ ---------- --KSRRAREY I-RSLPFKKK I-PWKAM--- ----F----- ------PKTS DLA------- ---LDLLEKL L--------- ---------- --AFNPAKRI TVEEALKHPY
1420954  LTLILDVLG- ----TPTMED YYGI------ ---------- --KSRRAREY I-RSLPFKKK V-PFRTL--- ----F----- ------PKTS DLA------- ---LDLLEKL L--------- ---------- --AFNPVKRI TVEDALKHPY
 146600  LTLILDVLG- ----TPTMED YYGI------ ---------- --KSRRAREY I-RSLPFKKK V-PFKGM--- ----F----- ------PKTS DLA------- ---LDLLEKL L--------- ---------- --AFNPVKRI TVEEALQHPY
 427542  LTLILDVLG- ----TPTMED YYGI------ ---------- --KSRRAREY I-RSLPFKKK I-PFKQM--- ----F----- ------PKTS DLA------- ---LDLLEKL L--------- ---------- --AFNPVKRI TVDEALQHPY
 524886  LTLILDVLG- ----TPTMED YYGI------ ---------- --KSRRAREY I-RSLPFKKK V-PFRTL--- ----F----- ------PKTS DLA------- ---LDLLEKL L--------- ---------- --AFNPVKRI TVEEALKHPY
 304061  LTLILDVLG- ----TPTMED YYGI------ ---------- --KSRRAREY I-RSLPFKKK I-PWKAM--- ----F----- ------PKTN DLA------- ---LDLLERL L--------- ---------- --AFNPVKRI TVEEALKHPY
 262011  LTLILDVLG- ----TPTMED YYGI------ ---------- --KSRRAREY I-RSLPFKKK V-PFKTL--- ----F----- ------PKTS ELA------- ---LDLLEKL L--------- ---------- --AFNPVKRI TVEEALKHPY
 385460  LTLILDVLG- ----TPTMED YYGI------ ---------- --KSRRAREY I-RSLPFKKK V-PFRTL--- ----F----- ------PKTS DLA------- ---LDLLEKL L--------- ---------- --AFNPVKRI TVEEALKHPY
 322662  LTLILDVLG- ----TPTMED YYGI------ ---------- --KSRRAREY I-RSLPFKKK V-PFRTL--- ----F----- ------PKTS DLA------- ---LDLLEKL L--------- ---------- --AFNPVKRI TVEEALKHPY
1531698  LTLILDVLG- ----TPTMED YYGI------ ---------- --KSRRAREY I-RSLPFKKK V-PFRTL--- ----F----- ------PKTS DLA------- ---LDLLEKL L--------- ---------- --AFNPVKRI TVEDALKHPY
1273298  LTLILDVLG- ----TPTMED YYGI------ ---------- --KSRRAREY I-RSLPFKKK V-PFRTL--- ----F----- ------PKTS DLA------- ---LDLLEKL L--------- ---------- --AFNPVKRI TVEDALKHPY
 211888  LTLILDVLG- ----TPTMED YYGI------ ---------- --KSRRAREY I-RSLPFKKK I-PFRAM--- ----F----- ------PKSN ELA------- ---LDLLEKL L--------- ---------- --AFNPAKRI TVEEALRHPY
1140823  LTLILDVLG- ----TPTMED YYGI------ ---------- --KSRRAREY I-RSLPFKKK I-PFRAM--- ----F----- ------PKSN ELA------- ---LDLLEKL L--------- ---------- --AFNPAKRI TVEEALRHPY
  84032  LTLILDVLG- ----TPTMED YYGI------ ---------- --KSRRAREY I-RSLPFKKK I-PLKVL--- ----F----- ------PKTS DLA------- ---LDLLEKL L--------- ---------- --SFNPAKRI TVEEALRHPY
 337569  LTLILDVLG- ----TPTMED YYGI------ ---------- --KSRRAREY I-RSLPFKKK I-PLKVL--- ----F----- ------PKTS DLA------- ---LDLLEKL L--------- ---------- --AFNPAKRI TVEDALRHPY
 675946  LTLILDVLG- ----TPTMED YYGI------ ---------- --KSRRAREY I-RSLPFKKK I-PWKAM--- ----F----- ------PKTS DLA------- ---LDLLERL L--------- ---------- --AFNPVKRI TVEDALKHPY
 188798  LTLILDVLG- ----TPTMED YYGI------ ---------- --KSRRAREY I-RSLPFKKK I-PWKAM--- ----F----- ------PKTN DLA------- ---LDLLERL L--------- ---------- --AFNPVKRI SVEDALKHPY
 364357  LTLILDVLG- ----TPTMED YYGI------ ---------- --KSRRAREY I-RSLPFKKK I-PWKAM--- ----F----- ------PKTN DLA------- ---LDLLERL L--------- ---------- --AFNPVKRI TVEDALKHPY
 417259  LTLILDVLG- ----TPTMED YYGI------ ---------- --KSRRAREY I-RSLPFKKK I-PWKAM--- ----F----- ------PKTN DLA------- ---LDLLERL L--------- ---------- --AFNPVKRI TVEEALKHPY
 391850  LTLILDVLG- ----TPTMED YYGI------ ---------- --KSRRAREY I-RSLPFKKK I-PWKAM--- ----F----- ------PKTN DLA------- ---LDLLERL L--------- ---------- --AFNPVKRI TVEEALKHPY
 420539  LTLILDVLG- ----TPTMED YYGI------ ---------- --KSRRAREY I-RSLPFKKK I-PWKVM--- ----F----- ------PKTS DLA------- ---LDLLERL L--------- ---------- --AFNPVKRI SVEDALKHPY
1649896  LTLILDVLG- ----TPTMED YYGI------ ---------- --KSRRAREY I-RSLPFKKR I-PFSAM--- ----F----- ------PKTS ASA------- ---LDLLEKL L--------- ---------- --AFNPAKRI TVEDALKHPY
 280694  LTLILEMLG- ----TPTMED YYGI------ ---------- --KSRRAREY I-RSLPFKKR V-PFSTL--- ----F----- ------PDTS PQA------- ---LDLLEKL L--------- ---------- --AFNPAKRI TVEEALKHPY
 381396  LTLILDVLG- ----TPTMED YYGI------ ---------- --KSRRAREY I-RSLPFKKK I-PFKAL--- ----F----- ------PKSN DQA------- ---LDLLEKL L--------- ---------- --AFNPAKRI SVEEALQHPY
 685453  LTLILDVLG- ----TPTMED YYGI------ ---------- --KSRRAREY I-RSLPFKKK V-PFRTL--- ----F----- ------PKTS DLA------- ---LDLLEKL L--------- ---------- --AFNPVKRI TVEEALKHPY
 284413  LTLILDVLG- ----TPTMDD FYGI------ ---------- --KSRRARDY I-RSLPFKKR I-PFARL--- ----F----- ------PNAN PMA------- ---VDLLEKL L--------- ---------- --TFNPVRRI TVEDALKHPY
 384436  LTLILDVLG- ----TPTMED YYGI------ ---------- --KSRRAREY I-RSLPFKKK I-PFRAM--- ----F----- ------PKSN ELA------- ---LDLLEKL L--------- ---------- --AFNPAKRI TVEEALRHPY
 907966  LTLILDVLG- ----TPTMED YYGI------ ---------- --KSRRAREY I-RSLPFKKR I-PFSAM--- ----F----- ------PKTS ASA------- ---LDLLEKL L--------- ---------- --AFNPAKRI TVEDALKHPY
 205730  LSLILDMLG- ----TPSLED FNAI------ ---------- --KSRRARDY I-RSLPFKKK R-SFAQE--- ----F----- ------PRAN PNA------- ---VDLLEKM L--------- ---------- --TFNPTKRI TVDEALSHPY
  86646  LSLILDVLG- ----TPSMDD FYAI------ ---------- --GSKRARDY I-RGLPFKRK I-PFNTL--- ----F----- ------PNAS PLA------- ---IDMLEKL L--------- ---------- --AFNPRKRI TCEEALKHPY
   9721  LTLILDVLG- ----TPSIDD FTAI------ ---------- --KSRRARDY I-RGLPIKNK I-PFTTL--- ----F----- ------PNAT PLA------- ---IDMLEKL L--------- ---------- --AFNPRKRI TCEEALKHPY
   2612  LALIFDVLG- ----TPTMED YYSV------ ---------- --KSRRAREY I-RGLPFKKR V-PFNVL--- ----F----- ------QGHN PLA------- ---LDLLEKL L--------- ---------- --SFNPSKRI TVEQALQHPY
 102501  LFLIIEILG- ----TPSRED IAQI------ ---------- --NSRRAREY I-NSLPFKSR I-PFQQQ--- ----F----- ------PNTN PLA------- ---IDLLERL L--------- ---------- --TFNPLKRI TVDEALTHPY
  98652  LYLIMDVLG- ----TPSMED IRDI------ ---------- --KSRRAREY I-RSLPLKRK V-ALNEK--- ----F----- ------PSNN PLA------- ---LDLLEKL L--------- ---------- --TFNPVKRI KVEEALTHEY
  78159  LWLIMDVLG- ----TPTSED YYNI------ ---------- --KSKRAREY I-RSLPFKKR A-ALDKL--- ----F----- -----VEGTN PLA------- ---IDLLEKL L--------- ---------- --SFNPQKRI TVSEALNHPY
 240194  LWLIMEVLG- ----TPNMED YYNI------ ---------- --KSKRAREY I-RSLPFCKK I-PFQTL--- ----FNDMMS NSNTNYDPIN PLA------- ---IDLLEKL L--------- ---------- --TFNPTKRI TVDNALNHPY
 193495  LWLIMEVLG- ----TPTMDD YYNI------ ---------- --KSKRAREY I-RTLPFRKK V-PLHTI--- ----F----- -----PQGTN PLA------- ---LDLLEKL L--------- ---------- --TFNPQKRI SVVQALDHPY
 171744  LWLIMEVLG- ----TPTMDD YYNI------ ---------- --KSKRAREY I-RTLPFRKK V-PLHTI--- ----F----- -----PQGTN PLA------- ---LDLLEKL L--------- ---------- --TFNPQKRI SVVQALDHPY
   4564  LQLIIQTLG- ----TPTADD FNVI------ ---------- --KSWRAKEY I-RMLPLCNK K-PFSEL--- ----F----- ------PDAN PLA------- ---VDLLEKL L--------- ---------- --SFNPKKRI TVTQALEHPY
   6035  LWLIIDVLG- ----TPLMED YSSI------ ---------- --KSRRAKEY I-RTLPFRKK K-NFRDL--- ----F----- ------PDAN PDA------- ---IDLLEKL L--------- ---------- --TFNPKKRI TVEEALNHPY
 256278  LTLILDVLG- ----TPTMED YYGI------ ---------- --KSRRAREY I-RSLPFKKK I-PFKAL--- ----F----- ------PKSN DQA------- ---LDLLEKL L--------- ---------- --AFNPAKRI SVEEALQHPY
 120199  LTLILDVLG- ----TPTLDE FYAI------ ---------- --TTRRSRDY I-RALPFRKK K-SFATI--- ----F----- ------PNAN PLA------- ---IDFLTKT L--------- ---------- --TFDPKKRI TVEDALAHPY
1003994  LTLILDVLG- ----TPTLDE FYAI------ ---------- --TTRRSRDY I-RALPFRKR R-PFAQL--- ----F----- ------PNAS SLA------- ---VDFLTKT L--------- ---------- --SFDPKKRL TVEQALAHPY
 145010  LTLILDVLG- ----TPTLDE FYAI------ ---------- --TTRRSRDY I-RALPFRKR K-PFAQL--- ----F----- ------PNAS PLA------- ---VDFLTRS L--------- ---------- --TFDPKKRI TVEEALAHPY
 183924  LTLILDVLG- ----TPTLDE FYAI------ ---------- --TTRRSRDY I-RALPFRKR K-PFAQL--- ----F----- ------PNAN PLA------- ---IDFLTRT L--------- ---------- --TFDPKKRI TVEDALAHPY
 725911  LTLILDVLG- ----TPTLDE FYAI------ ---------- --TTRRSRDY I-RALPFRKR K-PFAQL--- ----F----- ------PNAN PLA------- ---VDFLTKS L--------- ---------- --TFDPKKRI TVEEALAHPY
1166877  LTLILDVLG- ----TPTLDE FYAI------ ---------- --TTRRSRDY I-RALPFRKR K-PFAQL--- ----F----- ------PNAS PLA------- ---VDFLTKS L--------- ---------- --TFDPKKRI TVEEALAHPY
 574353  LTLILDVLG- ----TPTLDE FYAI------ ---------- --TTRRSRDY I-RALPFRKR K-PFAQL--- ----F----- ------PNAN PLA------- ---IDFLTRT L--------- ---------- --TFDPKKRI TVEDALAHPY
 277341  LTLILDVLG- ----TPTLDE FYAI------ ---------- --TTRRSRDY I-RALPFRKR R-PFATL--- ----F----- ------PNAN PLA------- ---VDFLTKT L--------- ---------- --TFDPKKRI TVEDALAHPY
 965410  LTLILDVLG- ----TPTLDE FYAI------ ---------- --TTRRSRDY I-RALPFRKR R-PFAQL--- ----F----- ------PNAN PSA------- ---VDFLTKT L--------- ---------- --TFDPKKRI TVEEALAHPY
1040134  LTLILDVLG- ----TPTLDE FYAI------ ---------- --TTRRSRDY I-RALPFRKR R-PFAQL--- ----F----- ------PNAT PMA------- ---VDFLSKT L--------- ---------- --TFDPKKRI SVEDALAHPY
 801239  LTLILDVLG- ----TPTLDE FYAI------ ---------- --TTRRSRDY I-RALPFRKR R-PFAQL--- ----F----- ------PNAN PLA------- ---VDFLTKT L--------- ---------- --TFDPKKRI TVEEALAHPY
 457049  LTLILDVLG- ----TPTLDE FYAI------ ---------- --TTRRSRDY I-RALPFRKR K-PFSQL--- ----F----- ------PNAN PLA------- ---VDFLTRT L--------- ---------- --TFDPKKRI TVEDALAHPY
 777636  LTLILDVLG- ----TPTLDE FYAI------ ---------- --TTRRSRDY I-RALPFRKR R-PFSQL--- ----F----- ------PNAN PLA------- ---IDFLTKT L--------- ---------- --TFDPKKRI TVEDALAHPY
 167531  LTLILDVLG- ----TPTLDE FYAI------ ---------- --TTRRSRDY I-RALPFRKR K-PFAQI--- ----F----- ------PSNN PLA------- ---IDFLTKA L--------- ---------- --TFDPKKRM TVEEALAHPY
 179443  LTLILDVLG- ----TPTLDE FYAI------ ---------- --TTRRSRDY I-RALPFRKK K-PFATL--- ----F----- ------PNAN PLA------- ---IDFLTRS L--------- ---------- --TFDPKKRI TVEEALAHPY
 388203  LTLILDVLG- ----TPTLDE FYAI------ ---------- --TTRRSRDY I-RALPFRKR K-PFTQL--- ----F----- ------PNAN PNA------- ---IDFLTKT L--------- ---------- --TFDPKKRI TVEQALAHPY
 761034  LTLILDVLG- ----TPTLDE FYAI------ ---------- --TTRRSRDY I-RALPFRKK K-PFAQL--- ----F----- ------PNAS PLA------- ---VDFLTRS LVSIGNAPVA CAGALISPFS LQTFDPKKRI TVEEALAHPY
 117742  LTLILDVLG- ----TPTLDE FYAI------ ---------- --TTRRSRDY I-RALPFRKK R-PFSQL--- ----F----- ------PNAT PLA------- ---IDFLTKT L--------- ---------- --TFDPKKRI TVEMALCHPY
 506090  LTLILDVLG- ----TPTIEE FYNI------ ---------- --STRRSRDY L-RALPFRKK K-SFASL--- ----F----- ------PNAS PLA------- ---VDFLTRT L--------- ---------- --TFDPKKRM TVEAALAHPY
  21911  LSLILDVLG- ----TPTIEE FYNI------ ---------- --STRRSRDY L-RALPFRKK K-SFASL--- ----F----- ------PNAS PLA------- ---VDFLTRT L--------- ---------- --TFDPKKRM TVEAALAHPY
7342346  LTLILDVLG- ----TPTLDE FYAI------ ---------- --STRRSRDY I-RALPFRKR R-SFATI--- ----F----- ------PNAS ALA------- ---VDFLTKT L--------- ---------- --TFDPKKRI TAEDALAHPY
 435735  LTLILDVLG- ----TPTLDE FYAI------ ---------- --TTRRSRDY I-RALPFRKR R-PFSTL--- ----F----- ------PNAN PLA------- ---IDFLTKT L--------- ---------- --TFDPKKRI TCEEALAHPY
 267232  LTLILDVLG- ----TPTLDE FYAI------ ---------- --TTRRSRDY I-RALPFRKR R-PFATL--- ----F----- ------PNAN PLA------- ---VDFLTKT L--------- ---------- --TFDPKKRI TVEDALAHPY
 679497  LTLILDVLG- ----TPTLDE FYAI------ ---------- --TTRRSRDY I-RALPFRKR K-PFSQL--- ----F----- ------PNAN PLA------- ---VDFLAKT L--------- ---------- --TFDPKKRI TVEDALAHPY
 457636  LTLILDVLG- ----TPTLDE FYAI------ ---------- --TTRRSRDY I-RALPFRKR K-PFAQL--- ----F----- ------PNAN PLA------- ---VDFLAKT L--------- ---------- --TFDPKKRI TVENALAHPY
```

```
1340042  LTLILDVLG- ----TPTLDE FYAI------ ---------- --NSRRSRDY L-RALPFRKK R-PFSQL--- ----Y----- ------PNAS ALA------- ---VDFLTKC L--------- ---------- --TFDPKKRL TVEQALEHPY
 225932  LTLILDVLG- ----TPTLDE FYAI------ ---------- --NSRRSRDY I-RALPFRKK R-PFNVL--- ----Y----- ------PNAS ALA------- ---IDFLNKT L--------- ---------- --TFDPKKRL TVEEALQHPY
 346924  LSRILDVLG- ----TPTIEE FYAI------ ---------- --TSRRSKEY V-RNMPFRKK K-DFATL--- ----Y----- ------PNAS PAA------- ---IDFLTKT L--------- ---------- --TFDPKKRF TVEECLSHPY
 760343  LTLILDVLG- ----TPTLDE FYAI------ ---------- --NSRRSRDY L-RALPFRKK R-PFSQL--- ----Y----- ------PNAS ALA------- ---VDFLTKC L--------- ---------- --TFDPKKRL TVEQALEHPY
  15147  LRLILDVLG- ----TPPMEE FYDV------ ---------- --TSRRSRDY L-RNLPVRTK K-SFESL--- ----Y----- ------PKAS PAA------- ---IDFLKRT L--------- ---------- --TFSSKKRM TVEECLQHPY
 108680  LTLILDILG- ----TPSLDD FYAI------ ---------- --SSHRSRDY I-RALPFKKK K-PFSQL--- ----Y----- ------PNAS ALA------- ---IDLLERC L--------- ---------- --TFNPKKRI TCEEALRHPY
 724148  LTLILDILG- ----TPSLDD FYAI------ ---------- --SSHRSRDY I-RALPFKKK K-PFSQL--- ----Y----- ------PNAS ALA------- ---IDLLERC L--------- ---------- --TFNPKKRI TCEEALRHPY
 276168  LSLTLEILG- ----TPSLDD FYAI------ ---------- --SSTRSRDY I-RALPFRKR R-NFPQM--- ----F----- ------PNAN PLA------- ---VDLMERC L--------- ---------- --TFSPRKRI TAEDALAHPY
 194959  LDLILDVIG- ----TPSLDE FYAI------ ---------- --TSRRSRDY I-RALPIRKK R-PFTSL--- ----F----- ------PQAS KEA------- ---IDFLTKT L--------- ---------- --TFDPKKRM TVDEALEHPY
 345309  LDLILDVIG- ----TPSLDE FYAI------ ---------- --TSRRSRDY I-RALPIRKK R-PFPTL--- ----F----- ------PHAS QEA------- ---IDFLTKT L--------- ---------- --TFDPKKRM TVDEALEHPY
7189608  LDLILDVIG- ----TPSLDE FYAI------ ---------- --TSRRSRDY I-RALPIRKK R-PFPTL--- ----F----- ------PQAS TDA------- ---IDFLAKT L--------- ---------- --TFDPKKRM AVDEALEHPY
 439783  LDLILDVIG- ----TPSLDE FYAI------ ---------- --TSRRSRDY I-RALPIRKK R-PFPTL--- ----F----- ------PQAS AEA------- ---IDFLSKT L--------- ---------- --TFDPKKRM AVDEALEHPY
  73775  LDLILDVIG- ----TPSLDE FYAI------ ---------- --TSRRSRDY I-RALPIRKK R-PFPTL--- ----F----- ------PHAS AEA------- ---IDFLSKT L--------- ---------- --TFDPKKRM TTDEALEHPY
 681486  LDLILDVIG- ----TPSLEE FYAI------ ---------- --TSRRSRDY I-RALPIRKR R-SWETL--- ----F----- ------PHAS KEA------- ---IDFLAKT L--------- ---------- --TFDPKKRM AVDEALEHPY
 671673  LDLILDVIG- ----TPSLDE FYAI------ ---------- --TSRRSRDY I-RALPIRKR R-SWESL--- ----F----- ------PHAS KEA------- ---IDFLAKT L--------- ---------- --TFDPKKRM TVDEALEHPY
 829815  LDLILDIIG- ----TPSLDE FYAI------ ---------- --TSRRSRDY I-RALPIRKR R-PFPTL--- ----F----- ------PQAS QEA------- ---IDFLSRT L--------- ---------- --TFDPKKRM NVDEALCHPY
 413627  LDLVLDVIG- ----TPTLEE FYGI------ ---------- --TSRRSRDY I-RALPIRKR R-PFTAL--- ----F----- ------PSAS ADA------- ---IDFLNQT L--------- ---------- --TFDPKKRM TVDAALEHPY
 109090  LDLILDVIG- ----TPTLDE YYSI------ ---------- --TSRRSREY I-RALPIRKR R-PFTAL--- ----F----- ------PKAS EDA------- ---IDFLQRT L--------- ---------- --TFDPKKRI TVEQALEHPY
  43366  LELILDVIG- ----TPSLEE FYAI------ ---------- --TSRRSRDY I-RALPIRKR R-PFAAL--- ----F----- ------PKAS PEA------- ---IDFLSKT L--------- ---------- --TFDPKKRI TADQALEHPY
 706326  LDLILDVIG- ----TPTLEE FYGI------ ---------- --TSRRSRDY I-RALPIKKR R-SFGAL--- ----F----- ------PKAS EDA------- ---LDFLAKT L--------- ---------- --TFDPKKRL TVDQALEHPY
1157202  LDLILDVIG- ----TPTLEE FYGI------ ---------- --TSRRSRDY I-RALPIKKR K-SFSSV--- ----F----- ------PKAT PEA------- ---LDFLSKT L--------- ---------- --TFDPKKRL TVDQALEHPY
 743174  LDLILDVIG- ----TPTLEE FYSI------ ---------- --TSRRSRDY I-RALPIKKR K-SFSAL--- ----F----- ------PKAS PEA------- ---LDFLRKT L--------- ---------- --TFDPKKRL TVDQALEHPY
   5331  LDLILDVIG- ----TPTLEE FYGI------ ---------- --TSRRSRDY I-RALPIKKR K-SFSTI--- ----F----- ------PKAT PEA------- ---LDFLTKT L--------- ---------- --TFDPKKRL TVDQALEHPY
 713967  LDLILDVIG- ----TPTLEE FYSI------ ---------- --TSRRSRDY I-RALPIKKR K-SFSAL--- ----F----- ------PKAS PEA------- ---LDFLRKT L--------- ---------- --TFDPKKRL TVDQALEHPY
 151043  LDLILDVIG- ----TPTLEE FYGI------ ---------- --TSRRSRDY I-RALPIRKR R-PFTAL--- ----F----- ------PKAS PEA------- ---IDFLQKT L--------- ---------- --TFDPKKRL TVDQALDHPY
 481570  LDLILDVIG- ----TPTLEE FYGI------ ---------- --TSRRSRDY I-RALPIRKR R-PFTAL--- ----F----- ------PKAS PEA------- ---IDFLQKT L--------- ---------- --TFDPKKRL TVDQALDHPY
 104738  LDLILDVIG- ----TPTLDE FYGI------ ---------- --TSRRSRDY I-RALPIRKR R-PFTAL--- ----F----- ------PKAS PDA------- ---IDFLQKT L--------- ---------- --TFDPKKRL TVDQALEHPY
 689474  LDLILDVIG- ----TPTLDE FYSI------ ---------- --TSRRSRDY I-RALPIRKR K-QFTQL--- ----F----- ------PKAS ADA------- ---IDFLHKT L--------- ---------- --TFDPKKRL TVDEALDHPY
 179470  LDLILDVIG- ----TPTLDE FYSI------ ---------- --TSRRSRDY I-RALPIRKR K-SFTQL--- ----F----- ------PKAS ADS------- ---IDFLQRT L--------- ---------- --TFDQKKRL TVDEALEHPY
  31001  LDLILDVIG- ----TPTLAE FYSI------ ---------- --TSRRSRDY I-RALPIRKR K-NFSSL--- ----F----- ------PKAS PDA------- ---IDFLQKT L--------- ---------- --TFDQKRRL TVDEALQHPY
 155293  LDLILDVIG- ----TPTLEE YAGI------ ---------- --TSRRSRDY I-RALPIRRR K-PLISL--- ----I----- ------PSAT PST------- ---IDFLSNT L--------- ---------- --TFDPKKRM TIEEALRHPY
  83606  LDLILDVIG- ----TPTLEE YAGI------ ---------- --TSRRSRDY I-RALPIRRR K-PLISL--- ----I----- ------PSAT PST------- ---IDFLSNT L--------- ---------- --TFDPKKRM TIEEALRHPY
  79026  LSLILDTLG- ----TPSIDD FYAI------ ---------- --SSARSREY L-RALPFRKR V-NFHHV--- ----F----- ------SKAN PLA------- ---VDLMEKC L--------- ---------- --TFSPKRRI TVEEALQHPY
  79053  LSLILDTLG- ----TPSIDD FYAI------ ---------- --SSARSREY L-RALPFRKR V-NFHHV--- ----F----- ------SKAN PLA------- ---VDLMEKC L--------- ---------- --TFSPKRRI TVEEALQHPY
 188294  LSSILDILG- ----TPSIDD FYAI------ ---------- --TSPRSREY I-RALPFRKK K-NFAQL--- ----F----- ------PKAN PLA------- ---IDLLEKC L--------- ---------- --TFSPKRRI EVSEALKHPY
 422236  LSIILDILG- ----TPSIDD FYAI------ ---------- --SSARSREY I-RALPFRKK K-NFATL--- ----F----- ------PKAN PLA------- ---IDLLEKL L--------- ---------- --TFSPKRRI EVEEALRHPY
  59295  LSIILDVLG- ----TPSLDD FYAI------ ---------- --TSQRSREY I-RALPFRKK R-QFSSL--- ----F----- ------PGAN PLA------- ---LDLMEKC L--------- ---------- --TFSPKRRI DVDEALQHPY
  91727  LSIILDILG- ----TPSLDD FYAI------ ---------- --TSQRSREY I-RALPFRKK K-PFSQL--- ----F----- ------PTAN PLA------- ---IDLMEKC L--------- ---------- --TFSPKRRI EVEDALKHPY
  92869  LSIILDILG- ----TPSLDD FYAI------ ---------- --TSQRSREY I-RALPFRKK K-PLSQL--- ----F----- ------PTAN PLA------- ---IDLMEKC L--------- ---------- --TFSPKRRI EVEDALKHPY
 513929  LSIILDILG- ----TPSLDD FYAI------ ---------- --TSQRSREY I-RALPFRKK K-PFSQL--- ----F----- ------PGAN PQA------- ---IDLMEKC L--------- ---------- --TFSPKRRI EVEEALKHPY
 166192  LSIILDILG- ----TPSLDD FYAI------ ---------- --TSQRSREY I-RALPFRKK K-PLSQL--- ----F----- ------PTAN PLA------- ---IDLMEKC L--------- ---------- --TFSPKRRI EVEDALKHPY
 210022  LSIILDVLG- ----TPTLDD FYAI------ ---------- --TSPRSREY I-RALPFRKK K-PFSVL--- ----F----- ------PGAN PLA------- ---VELLERC L--------- ---------- --TFSPKRRI QVEEALRHPY
 847083  LSIILDILG- ----TPSIDD FYAI------ ---------- --TSSRSREY I-RALPFRKK R-PFAQL--- ----F----- ------PNAS PLA------- ---IDFLEHC L--------- ---------- --TFSPKRRI EVGEALKHPY
 819022  LSIILDILG- ----TPSIDD FYAI------ ---------- --TSQRSREY I-RALPFRKK K-SFHQL--- ----F----- ------PKAN PLA------- ---IDLMEKC L--------- ---------- --TFSPKRRI EVGEALKHPY
  50473  LSIILDILG- ----TPSIDD FYAI------ ---------- --TSQRSREY I-RALPFRKK K-SFHQL--- ----F----- ------PKAN PLA------- ---IDLMEKC L--------- ---------- --TFSPKRRI EVGEALKHPY
 850567  LSLILDVLG- ----TPSIDD FYAI------ ---------- --SSPRSREY I-RALPFRRG K-DFKIM--- ----F----- ------QGAN ELA------- ---IDFLEKC L--------- ---------- --TFSPQRRI TVVEALQHPY
 183076  LSLILDVLG- ----TPSIDD FYAI------ ---------- --SSPRSREY I-RALPFRKG K-ELSGM--- ----F----- ------SGAN ELA------- ---IDFLEKC L--------- ---------- --TFSPRRRI TVVEALQHPY
1114014  LSIILDILG- ----TPSIDD FYAI------ ---------- --SSSRSREY I-RALPFRKN R-PFAQL--- ----F----- ------PQAN PLA------- ---IDFLEKC L--------- ---------- --TFSPKRRI TVGEALQHPY
  47661  LSLILDILG- ----TPSLDD FYAI------ ---------- --TSPRSREY I-RALPFRKK K-PFSTL--- ----F----- ------PNAN PLA------- ---IDFLEQC L--------- ---------- --TFSPKRRI QVEEALKHPY
6536531  LSLILETLG- ----TPSIDD FYAI------ ---------- --NSQRSREY I-RALPFRRK K-NFSQM--- ----F----- ------PNAN PLA------- ---IDLMEKC L--------- ---------- --AFSPKRRL DVAEALRHPY
 277624  LSLILETLG- ----TPSIDD FYAI------ ---------- --NSQRSREY I-RALPFRRK R-NFTQM--- ----F----- ------PNAN PLA------- ---IDLMEKC L--------- ---------- --AFSPKKRL DVAEALRHPY
  77457  LSLILETLG- ----TPSIDD FYAI------ ---------- --NSQRSREY I-RALPFRRK K-NFAQM--- ----F----- ------PNAN PLA------- ---IDLMEKC L--------- ---------- --AFSPKKRL DVSEALRHPY
  89431  LSLILETLG- ----TPSIDD FYAI------ ---------- --NSSRSREY I-RALPFKKK K-NFAQM--- ----F----- ------PKAN PLA------- ---IDLMEKC L--------- ---------- --TFSPKRRL PVSEALQHPY
 484632  LSLILETLG- ----TPSIDD FYAI------ ---------- --NSSRSREY I-RALPFKKK K-SFAQM--- ----F----- ------PNAN PLA------- ---IDLMEKC L--------- ---------- --TFSPKRRL QVSEALQHPY
 161998  LSLILDILG- ----TPSIDD FYAI------ ---------- --SSPRSREY I-RALPFRKK R-SLMTL--- ----F----- ------PNAN PLA------- ---VDLMEKC L--------- ---------- --TFSPKKRI QVDDALKHPY
 324877  LSIILDILG- ----TPSIDD FYAI------ ---------- --TSQRSREY L-RALPFRKQ K-PFSQL--- ----F----- ------PEAN PLA------- ---IDLMEKC L--------- ---------- --TFSPKKRI DVSEALKHPY
 117759  LSIILDILG- ----TPSLDD FYAI------ ---------- --NSTRSREY I-RALPFRKK K-SFASL--- ----F----- ------PNAN PLA------- ---VDLMEKC L--------- ---------- --TFNPKRRI EVGDALAHPY
 489827  LSIILDVLG- ----TPSLDD FYAI------ ---------- --TSLRSREY I-RALPFRKK K-PFGQL--- ----F----- ------PNAN PLA------- ---VDLMERC L--------- ---------- --TFNPKMRI TVEEALGHPY
 115935  LSIILDVLG- ----TPSLDD FYAI------ ---------- --TSLRSREY I-RALPFRKK K-PFTQL--- ----F----- ------PNAN PLA------- ---VDLMERC L--------- ---------- --TFNPKMRV TVEEALGHPY
 482781  LSIILDVLG- ----TPSLDD FYAI------ ---------- --TSLRSREY I-RALPFRKK K-PFSQL--- ----F----- ------PNAN PLA------- ---VDLMERC L--------- ---------- --TFNPKMRI TVEEALGHPY
 545919  LSIILDVLG- ----TPSLDD FYAI------ ---------- --TSLRSREY I-RALPFRKK K-PFSQL--- ----F----- ------PNAN PLA------- ---VDLMERC L--------- ---------- --TFNPKMRI TVEEALGHPY
6536562  LSLILETLG- ----TPSIDD FYAI------ ---------- --NSQRSREY I-RALPFRRK K-NFSQM--- ----F----- ------PNAN PLA------- ---IDLMEKC L--------- ---------- --AFSPKRRL DVAEALRHPY
 309997  LSLILDVVG- ----TPNMDD FDEI------ ---------- --TSARSKDY L-RQLPFRMR R-DFAEI--- ----C----- ------PKAT PRA------- ---VDFIKRC L--------- ---------- --TFSPTKRM TVQQALEHPY
 316873  LSLILDVVG- ----TPNMDD FDEI------ ---------- --TSARSKDY L-RQLPFRMR R-DFAEI--- ----C----- ------PKAT PRA------- ---VDFIKRC L--------- ---------- --TFSPTKRM TVQQALEHPY
 429267  LSIILDILG- ----TPSIDD FYAI------ ---------- --TSQRSREY L-RALPFRKQ K-PFSQL--- ----F----- ------PEAN PLA------- ---IDLMEKC L--------- ---------- --TFSPKKRI DVSEALKHPY
 103465  LLLIFELLG- ----TPSSDD MLSV------ ---------- --RSKRAREY I-RTLPQFPR V-NLRAK--- ----F----- ------SFAN PLG------- ---LDLLEKL L--------- ---------- --IFDPTKRI KVTEALEHPY
 167039  LMLIFQVLG- ----SPIGDD YKSI------ ---------- --RSRRAREY I-RSLTFYKK Q-PFSQL--- ----F----- ------PSAN PKA------- ---IDLLDKM L--------- ---------- --TFDPDKRI TVYEALEHPY
   8637  LALIFEVLG- ----TPTAEE MKCI------ ---------- --KLKKARDF V-ASQRVRRK I-PMKKI--- ----F----- ------RNAN PLA------- ---IDFIEKT V--------- ---------- --CFDPEKRM TIEQTLEHPY
  65963  LLLIFEILG- ----TPTGAD FQSI------ ---------- --KSKRAREY I-RSQRFFKK I-PLRKI--- ----F----- ------PHAN PSA------- ---VDLMDRM L--------- ---------- --KFDPTQRL TVEEALEHPY
 246409  LSIILDVLG- ----TPSLDD FYAI------ ---------- --TSPRSREY I-RALPFRKK R-PFSAL--- ----F----- ------PNAN PLA------- ---LDLMEKC L--------- ---------- --TFSPKRRI EVEDTSLRRN
 246352  LSIILDVLG- ----TPSLDD FYAI------ ---------- --TSPRSREY I-RALPFRKK R-PFSAL--- ----F----- ------PNAN PLA------- ---LDLMEKC L--------- ---------- --TFSPKRRI EVEDTSLRRN
 668220  LTLILDVLG- ----TPTLDE FYAI------ ---------- --TTRRSRDY I-RALPFRKR K-PFAQL--- ----F----- ------PNAS PLA------- ---VDFLTRS L--------- ---------- --TFDPKKRI TVEEALAHPY
 619500  LTLILDVLG- ----TPTLDE FYAI------ ---------- --TTRRSRDY I-RALPFRKR K-PFSQL--- ----F----- ------PNAN PLA------- ---VDFLAKT L--------- ---------- --TFDPKKRI TVEDALAHPY
  64492  LTLILDVLG- ----TPTLDE FYAI------ ---------- --TTRRSRDY I-RALPFRKR K-PFAQL--- ----F----- ------PNAN PLA------- ---IDFLTRT L--------- ---------- --TFDPKKRI TVEDALAHPY
  80250  LTLILDILG- ----TPSLDD FYAI------ ---------- --SSHRSRDY I-RALPFKKK K-PFSQL--- ----Y----- ------PNAS ALA------- ---IDLLERC L--------- ---------- --TFNPKKRI TCEEALRHPY
  21917  LSIILDVLG- ----TPSLDD FYAI------ ---------- --TSLRSREY I-RALPFRKK K-PFTQL--- ----F----- ------PNAN PLA------- ---VDLMERC L--------- ---------- --TFNPKMRV TVEEALGHPY
 600175  LSIILDVLG- ----TPSLDD FYAI------ ---------- --TSPRSREY I-RALPFRKK K-SFSML--- ----F----- ------PNAN PLA------- ---LDLMEKC L--------- ---------- --TFSPKRRI EVDEALKHPY
 721307  LSIILDILG- ----TPSIDD FYAI------ ---------- --TSQRSREY I-RALPFRKK K-SFHQL--- ----F----- ------PKAN PLA------- ---IDLMEKC L--------- ---------- --TFSPKRRI EVGEALKHPY
 847084  LSIILDILG- ----TPSIDD FYAI------ ---------- --TSSRSREY I-RALPFRKK R-PFAQL--- ----F----- ------PNAS PLA------- ---IDFLEHC L--------- ---------- --TFSPKRRI EVGEALKHPY
 280696  LTLILEMLG- ----TPTMED YYGI------ ---------- --KSRRAREY I-RSLPFKKR V-PFSTL--- ----F----- ------PDTS PQA------- ---LDLLEKL L--------- ---------- --AFNPAKRI TVEEALKHPY
 908144  LTLILDVLG- ----TPTMED YYGI------ ---------- --KSRRAREY I-RSLPFKKR I-PFSAM--- ----F----- ------PKTS ASA------- ---LDLLEKL L--------- ---------- --AFNPAKRI TVEDALKHPY
 148879  LTLILDVLG- ----TPTMED YYGI------ ---------- --KSRRAREY I-RSLPFKKK I-PWKVM--- ----F----- ------PKTS DLA------- ---LDLLERL L--------- ---------- --AFNPVKRI TVEEALKHPY
 102030  LKLIFDVLG- ----TPSSDA YLGI------ ---------- --KSQRARDY I-RSLPLKAK A-NFNKM--- ----F----- ------PYAS PLA------- ---IDLMDKM L--------- ---------- --NFNPRKRI TVEEALAHPY
  92334  LKLIFDVLG- ----TPSSDA YLGI------ ---------- --KSQRARDY I-RSLPLKAK A-NFNKM--- ----F----- ------PYAS PLA------- ---IDLMDKM L--------- ---------- --NFNPRKRI TVEEALAHPY
 146634  LQLITDVLG- ----TPRESD MHFI------ ---------- --RSDKAKRF M-RAQPLRPK R-NYAEM--- ----F----- ------PNAS SEG------- ---LDLLDKM L--------- ---------- --QFNPDKRI SVEDALAHPY
  72485  LTQIMSVLG- ----TPSDEV LDRI------ ---IKPTPHD PQTPSRIRKY L-KSIKKMPK I-SLADR--- ----F----- ------PGAN PKA------- ---IDLLEKL L--------- ---------- --VWCPSDRL TAEQVLAHPY
 124061  LDLILDVIG- ----TPSLEE FYAI------ ---------- --TSRRSRDY I-RALPIRKR R-SWETL--- ----F----- ------PHAS KEA------- ---IDFLAKT L--------- ---------- --TFDPKKRM AVDEALEHPY
 109792  LTLILDVLG- ----TPTLDE FYAI------ ---------- --NSRRSRDY I-RALPFRKK R-PFNVL--- ----Y----- ------PNAS ALA------- ---IDFLNKT L--------- ---------- --TFDPKKRL TVEEALQHPY
 408982  LTLILDVLG- ----TPTLDE FYAI------ ---------- --TTRRSRDY I-RALPFRKK R-PFSTL--- ----F----- ------PNAN PLA------- ---IDFLTKT L--------- ---------- --TFDPKKRI TCEEALAHPY
 149279  LTLILDVLG- ----TPTLDE FYAI------ ---------- --TTRRSRDY I-RALPFRKR R-PFATL--- ----F----- ------PNAS PLA------- ---IDFLTKT L--------- ---------- --TFDPKKRI TVEEALAHPY
  13526  ---------- ---------D FYAI------ ---------- --NSQRSREY I-RALPFRRK K-NFAQM--- ----F----- ------PNAN PLA------- ---IDLMEKC L--------- ---------- --AFSPKKRL DVSEALRHPY
 465497  ---------- ---------D FYAI------ ---------- --NSSRSREY I-RALPFKKK K-SFAQM--- ----F----- ------PNAN PLA------- ---IDLMEKC L--------- ---------- --TFSPKRRL QVSEALQHPY
 216389  LSLILETLG- ----TPSIDD FYAI------ ---------- --NSQRSREY I-RALPFRRK K-SFAQM--- ----F----- ------PAAN PLA------- ---IDLMEKC L--------- ---------- --AFSPKKRL DVSEALRHPY
  17191  ISLILEVLG- ----TPSIDD FYAI------ ---------- --GSRRSRDY I-RQMPLRKK K-DFNKL--- ----F----- ------PTAS ADA------- ---IDFLTKT L--------- ---------- --AFNPKKRP TVEECLRHPY
 530963  FSIITELLG- ----TPPDDV IATI------ ---------- --CSENTLRF V-QSLPKRER V-PFGQK--- ----F----- ------RTQD PVGKYMPMSH VGPLDLLERM L--------- ---------- --VFDPRKRI TAAESLAHEY
  84099  FSIITELLG- ----TPPDDV ISTI------ ---------- --CSENTLRF V-QSLPKRER Q-PLKNK--- ----F----- ------KNAD PQGRSLTSSA I---ELLERM L--------- ---------- --VFDPRKRV KAGEALADPY
 928715  LTLILDVLG- ----TPQIDD FHQI------ ---------- --SSQRSRDY L-RALPFKKR R-PFANL--- ----Y----- ------PDAN QQA------- ---VDLLEKL L--------- ---------- --AFNPKKRI NVTDALAHPY
 480104  LDLILDVIG- ----TPSLDE FYAI------ ---------- --TSRRSRDY I-RALPIRKK R-PFTSL--- ----F----- ------PQAS KEA------- ---IDFLTKT L--------- ---------- --TFDPKKRM TVDEALEHPY
  37144  LDLILDVMG- ----TPTLDE FYSI------ ---------- --TSRRSRDY I-RALPIRRR K-SFTQM--- ----F----- ------PKAS ADA------- ---IDFLQKT L--------- ---------- --TFDPKKRM TVDEALEHPY
 993637  LDLILDVMG- ----TPTLDE FYSI------ ---------- --TSRRSRDY I-RALPIRRR K-SFTQM--- ----F----- ------PKAS ADA------- ---IDFLQKT L--------- ---------- --TFDPKKRM TVDEALEHPY
 706327  LDLILDVIG- ----TPTLEE FYGI------ ---------- --TSRRSRDY I-RALPIKKR R-SFGAL--- ----F----- ------PKAS EDA------- ---LDFLAKT L--------- ---------- --TFDPKKRL TVDQALEHPY
   5366  LDLILDVIG- ----TPTLEE FYGI------ ---------- --TSRRSRDY I-RALPIKKR K-SFSTI--- ----F----- ------PKAT PEA------- ---LDFLTKT L--------- ---------- --TFDPKKRL TVDQALEHPY
 144998  LTLILDVLG- ----TPTLDE FYAI------ ---------- --TTRRSRDY I-RALPFRKR K-PFAQL--- ----F----- ------PNAS PLA------- ---VDFLTRS L--------- ---------- --TFDPKKRI TVEEALAHPY
 232199  LTLILDVLG- ----TPTLDE FYAI------ ---------- --TTRRSRDY I-RALPFRKR K-PFAQL--- ----F----- ------PNAN PLA------- ---VDFLTKS L--------- ---------- --TFDPKKRI TVEEALAHPY
  60405  LTLILDVLG- ----TPTLDE FYAI------ ---------- --TTRRSRDY I-RALPFRKR R-PFAQL--- ----F----- ------PNAN PSA------- ---VDFLTKT L--------- ---------- --TFDPKKRI TVEEALAHPY
 572635  LTLILDVLG- ----TPTLDE FYAI------ ---------- --TTRRSRDY I-RALPFRKR R-PFAQL--- ----F----- ------PNAN PLA------- ---VDFLTKT L--------- ---------- --TFDPKKRI TVEEALAHPY
  37912  LTLILDVLG- ----TPTLDE FYAI------ ---------- --TTRRSRDY I-RALPFRKK R-PFSQL--- ----F----- ------PNAN PLA------- ---IDFLTKT L--------- ---------- --TFDPKKRI TVEEALAHPY
 819552  LTLILDVLG- ----TPTLDE FYAI------ ---------- --TTRRSRDY I-RALPFRKR R-PFAQL--- ----F----- ------PNAT PMA------- ---VDFLSKT L--------- ---------- --TFDPKKRI SVEDALAHPY
1026351  LSSILDILG- ----TPSIDD FYAI------ ---------- --TSPRSREY I-RALPFRKK K-NFAQL--- ----F----- ------PKAN PLA------- ---IDLLEKC L--------- ---------- --TFSPKRRI EVSEALKHPY
 352542  LSIILDILG- ----TPSIDD FYAI------ ---------- --SSARSREY I-RALPFRKK K-NFATL--- ----F----- ------PKAN PLA------- ---IDLLEKL L--------- ---------- --TFSPKRRI EVEEALRHPY
  42114  LSLILETLG- ----TPSIDD FYAI------ ---------- --NSQRSREY I-RALPFRRK K-SFAQM--- ----F----- ------PAAN PLA------- ---IDLMEKC L--------- ---------- --AFSPKKRL DVSEALRHPY
 204011  LSIILDVLG- ----TPSLDD FYAI------ ---------- --TSLRSREY I-RALPFRKK K-PFGQL--- ----F----- ------PNAN PLA------- ---VDLMERC L--------- ---------- --TFNPKMRI TVEEALGHPY
  46962  LTLILDVLG- ----TPTMED YYGI------ ---------- --KSRRAREY I-RSLPFKKK I-PFKQM--- ----F----- ------PKTS DLA------- ---LDLLEKL L--------- ---------- --AFNPVKRI TVDEALQHPY
   3452  LTLILDVLG- ----TPTMED YYGI------ ---------- --KSRRAREY I-RSLPFKKK I-PWKAM--- ----F----- ------PKTN DLA------- ---LDLLERL L--------- ---------- --AFNPVKRI TVEEALKHPY
1531701  LTLILDVLG- ----TPTMED YYGI------ ---------- --KSRRAREY I-RSLPFKKK V-PFRTL--- ----F----- ------PKTS DLA------- ---LDLLEKL L--------- ---------- --AFNPVKRI TVEDALKHPY
 375250  LTLILDVLG- ----TPTMED YYGI------ ---------- --KSRRAREY I-RSLPFKKK I-PWKAM--- ----F----- ------PKTN DLA------- ---LDLLERL L--------- ---------- --AFNPVKRI TVEDALKHPY
 391393  LTLILDVLG- ----TPTMED YYGI------ ---------- --KSRRAREY I-RSLPFKKK I-PWKVM--- ----F----- ------PKTS DLA------- ---LDLLERL L--------- ---------- --AFNPVKRI SVEDALKHPY
 518679  LTLILDVLG- ----TPTMED YYGI------ ---------- --KSRRAREY I-RSLPFKKK I-PFKAL--- ----F----- ------PKSN DQA------- ---LDLLEKL L--------- ---------- --AFNPAKRI SVEEALQHPY
1272884  LTLILDVLG- ----TPTMED YYGI------ ---------- --KSRRAREY I-RSLPFKKK V-PFRTL--- ----F----- ------PKTS DLA------- ---LDLLEKL L--------- ---------- --AFNPVKRI TVEDALKHPY
 108249  LSLILDVLG- ----TPSMDD FYAI------ ---------- --GSKRARDY I-RGLPFKRK I-PFNTL--- ----F----- ------PNAS PLA------- ---IDMLEKL L--------- ---------- --AFNPRKRI TCEEALKHPY
 111885  LLLILEKLG- ----TPNEED FNII------ ---------- --KSDRARAY V-QSLPTFEK I-D------- ---------- -------WSK FLPFGTDF-- -LAIDLLDKL L--------- ---------- --TFNPHKRI SAEEALKHPF
 228039  LLLIFQLLG- ----TPKDED FNCI------ ---------- --KSKRAREY I-STLEFFYP V-DFDVF--- ----FN---- -----KHYNR FKKYGNERIN PLAIDLLKKL L--------- ---------- --VFDQNKRI TAEKALQHPY
 117728  LERILELTG- ----VPASED LQSI------ ---------- --QSPFAANM L-ESLAKPSI K-PLEEM--- ----I----- ------PNAP ADG------- ---LDFIRRC M--------- ---------- --HFNPDKRA SVEALLDHSY
 267276  LTLILDVLG- ----TPTLDE FYAI------ ---------- --TTRRSRDY I-RALPFRKR R-PFATL--- ----F----- ------PNAN PLA------- ---VDFLTKT L--------- ---------- --TFDPKKRI TVEDALAHPY
 295270  LTLILDVLG- ----TPTLDE FYAI------ ---------- --TTRRSRDY I-RALPFRKR K-PFAQL--- ----F----- ------PNAS PLA------- ---VDFLTRS L--------- ---------- --TFDPKKRI TVEEALAHPY
 174214  FSIITELLG- ----TPPDDV ISTI------ ---------- --CSENTLRF V-QSLPKRER Q-PLKNK--- ----FKN--- --------AD PQA------- ---IELLERM L--------- ---------- --VFDPRKRV KAGEALADPY
 111562  FSIITELLG- ----TPPDDV ISTI------ ---------- --CSENTLRF V-QSLPKRER Q-PLKNK--- ----FKN--- --------AD PQA------- ---IELLERM L--------- ---------- --VFDPRKRV KAGEALADPY
 385026  FSIITELLG- ----TPPDDV ISTI------ ---------- --CSENTLRF V-QSLPKRER L-PLKNK--- ----FKN--- --------AD PQA------- ---IELLERM L--------- ---------- --VFDPRKRV KAGEALADPY
  47937  FSIITELLG- ----TPPDDV IQTI------ ---------- --CSENTLRF V-QSLPKRER Q-PLTKK--- ----FKN--- --------AD PQA------- ---IELLERM L--------- ---------- --VFDPRTRI RAGDALADPY
 191658  FSIITELLG- ----TPPDDV IQTI------ ---------- --CSENTLRF V-QSLPKRER Q-PLANK--- ----FKN--- --------AE PDA------- ---VDLLENM L--------- ---------- --VFDPRKRV RAEQALAHAY
 361397  FSIITELLG- ----TPPDDV IQTI------ ---------- --CSENTLRF V-QSLPKRER I-PLANK--- ----FKN--- --------AE PAA------- ---VDLLENM L--------- ---------- --VFDPRKRV RAEEALAHSY
 368356  FSIITELLG- ----TPPDDV IQGI------ ---------- --CSENTLRF V-QSLPKRER I-PLSNK--- ----FKN--- --------AE PQA------- ---VDLLENM L--------- ---------- --VFDPKKRV RAEEALAHPY
 422287  FSIITELLG- ----TPPDDV IQTI------ ---------- --CSENTLRF V-QSLPKRER Q-PLANK--- ----FRN--- --------AE AEA------- ---IDLLESM L--------- ---------- --VFDPRKRV KATEALAHPY
```

```
  1051981  FSIITELLG- ----TPPDDV INTI------ ---------- --ASENTLRF V-KSLPKRER Q-PLKNK--- ----FRN--- --------AD PAA------- ---IDVLERM L--------- ---------- --VFDPKSRI TATEALAHEY
   512786  FSIITELLG- ----TPPDDV INTI------ ---------- --ASENTLRF V-KSLPKRER Q-PLKNK--- ----FKN--- --------AD PAA------- ---IDLLERM L--------- ---------- --VFDPKKRI TATEALAHEY
   700253  FSIITELLG- ----TPPDDV INTI------ ---------- --ASENTLRF V-KSLPKRER Q-PLKNK--- ----FKN--- --------AD PSA------- ---IDLLERM L--------- ---------- --VFDPKKRV TATEALSHDY
   171641  FSIITELLG- ----TPPDDV INTI------ ---------- --ASENTLRF V-KSLPKRER Q-PLKNK--- ----FKN--- --------AE PAA------- ---IDLLERM L--------- ---------- --VFDPKKRI TAADALAHEY
   447383  FSIITELLG- ----TPPDDV INTI------ ---------- --ASENTLRF V-KSLPKRER Q-PLTNK--- ----FKN--- --------AD EPA------- ---IDLLERM L--------- ---------- --VFDPKKRI TAAEALAHEY
   134451  FSIITELLG- ----TPPDDV IHTI------ ---------- --ASENTLRF V-QSLPKRER Q-PLKDK--- ----FKN--- --------AD PLA------- ---IDLLEEM L--------- ---------- --VFDPRKRV KAADALAHEY
   525556  FSIITELLG- ----TPPDDV IETI------ ---------- --CSENTLRF V-KSLPKRER Q-PLTNR--- ----FKN--- --------AD PEA------- ---VDLLERM L--------- ---------- --VFDPKKRI RAGEALAHEY
  1776212  FSIITELLG- ----TPPDDV IHTI------ ---------- --CSENTLRF V-QSLPKRER Q-PLSEK--- ----FKN--- --------AD PLA------- ---IDLLEHM L--------- ---------- --VFDPKKRI TAEAALAHEY
   443108  FSIITELLG- ----TPPDDV IQTI------ ---------- --CSENTLRF V-QSLPKRER Q-PLTNR--- ----FKN--- --------AD PQA------- ---IDMLERM L--------- ---------- --VFDPRKRI RAGEALAHPY
   129540  FSIITELLG- ----SPPEEV IKTI------ ---------- --CSANTLQF V-QSLPKRER Q-PLSQK--- ----FKD--- --------AD PLA------- ---VDLLEKM L--------- ---------- --VFDPKQRI TAANGLAHEY
   341724  FSIITELLG- ----SPPEEV IKTI------ ---------- --CSANTLQF V-QSLPKRER Q-PLSQK--- ----FKD--- --------AD PLA------- ---VDLLEKM L--------- ---------- --VFDPKQRI SAADGLAHEY
   138417  FSIITELLG- ----SPPEEV IKTI------ ---------- --CSANTLQF V-QSLPKRER Q-PLSQK--- ----FKD--- --------AD PLA------- ---VDLLEKM L--------- ---------- --VFDPKQRI TAANGLAHEY
   158191  FSIITELLG- ----TPPDDV IQTI------ ---------- --CSENTLRF V-QSLPKRDR Q-PLAQK--- ----FKN--- --------AD PLA------- ---IDLLENM L--------- ---------- --VFDPKKRV RAGDALAHPY
   376076  FSIITELLG- ----TPPDDV INTI------ ---------- --ASENTLRF V-KSLPKRER Q-PLKNK--- ----FKN--- --------AD PAA------- ---VDLLERM L--------- ---------- --VFDPKKRI TAADALAHEY
   368607  FSIITELLG- ----TPPDDV IQTI------ ---------- --CSENTLRF V-QSLPKRER I-PLSQK--- ----FKN--- --------AE PLA------- ---IDLLEKM L--------- ---------- --VFDPRKRI TAADALAHPY
  1312592  FSIITELLG- ----TPPDDV VQTI------ ---------- --ASENTLRF V-QSLPKRER V-PFTQK--- ----FKN--- --------AD PMA------- ---LDLLERM L--------- ---------- --VFDPKKRI TATESLGHEY
    46955  FSIITELLG- ----TPPEDV IQTI------ ---------- --ASENTLRF V-QSLPKRER V-PFSEK--- ----LHC--- --------KD PDA------- ---IDLLEKM L--------- ---------- --VFDPRTRI NATESLEHDY
  1164008  FSIITELLG- ----TPPEDV IQTI------ ---------- --ASENTLRF V-QSLPKRER V-PFSEK--- ----LSC--- --------ND PVA------- ---IDLLEKM L--------- ---------- --VFDPRKRI TATESLAHEY
   600771  FSIITELLG- ----TPPEDV IQTI------ ---------- --ASENTLRF V-QSLPKRER V-PFSEK--- ----LTC--- --------KD PVA------- ---IDLLEKM L--------- ---------- --VFDPRKRI NATESLAHEY
   161654  FSIITELLG- ----TPPDDV IATI------ ---------- --ASENTLRF V-QSLPKRER V-PFKQK--- ----LKC--- --------QD ESA------- ---LDLLEKM L--------- ---------- --VFDPRRRI NATESLAHEY
   149140  FSIITELLG- ----TPPDDV IATI------ ---------- --ASENTLRF V-QSLPKRER V-PFKQK--- ----LKC--- --------QD ESA------- ---LDLLEKM L--------- ---------- --VFDPRRRI NATESLAHEY
   474692  FSIITELLG- ----TPPDDV IETI------ ---------- --CSENTLRF V-QSLPKRQR V-PFSQK--- ----LRT--- --------TD PDA------- ---LDLLERM L--------- ---------- --VFDPRKRI NASESLAHAY
   134758  FSIITELLG- ----TPPDDV IATI------ ---------- --ASENTLRF V-QSLPKRER Q-PFSNK--- ----LHC--- --------KD PDA------- ---LDLLEKM L--------- ---------- --VFDPRKRI TATEALAHEY
   177791  FSIITELLG- ----TPPDDV IETI------ ---------- --ASENTLRF V-QSLPKRER I-PFSQK--- ----LRT--- --------SD PDA------- ---IDLLEKM L--------- ---------- --VFDPRKRI DASQSLAHEY
   132015  FSIITELLG- ----TPPDDV IETI------ ---------- --CSENTLRF V-QSLPKRER I-PFSQK--- ----LRT--- --------AD PDA------- ---IDLLEKM L--------- ---------- --VFDPRKRI DATQSLGHEY
   917530  FSIITELLG- ----TPPDDV IESI------ ---------- --ASENTLRF V-QSLPKRER V-PFSQK--- ----LRT--- --------TD PDA------- ---LALLEKM L--------- ---------- --VFDPRKRI NATECLEDEY
   428496  FSIITELLG- ----TPPDDV IQTI------ ---------- --ASENTLRF V-QSLPKRER V-PFSSK--- ----LRT--- --------AD ADA------- ---IDLLEKM L--------- ---------- --VFDPRKRI TATESLGHEY
  1276586  FSIITELLG- ----TPPDDV IQTI------ ---------- --CSENTLRF V-QSLPKRDK I-PFGEK--- ----LRN--- --------AD SVS------- ---IDLLERM L--------- ---------- --VFDPRKRI TAVNALDHPY
   115601  FSIITELLG- ----TPPDDV IATI------ ---------- --CSENTLRF V-QSLPKRER V-PFGQK--- ----FRT--- --------QD PVA------- ---LDLLERM L--------- ---------- --VFDPRKRI TAAESLAHEY
   171809  FSIITELLG- ----TPDDDV IKTI------ ---------- --CSENTLRF V-QSLPKRDR V-PFADK--- ----FRN--- --------TD GPA------- ---LDLLEKM L--------- ---------- --VFDPRKRI NATEALAHEY
   571775  FSIITELLG- ----TPPDDV IETI------ ---------- --CSENTLRF V-QSLPKRER I-PFSQK--- ----LRT--- --------AD PDA------- ---IDLLEKM L--------- ---------- --VFDPRKRI DATQSLGHEY
   722302  FSIITELLG- ----TPPDDV IATI------ ---------- --CSENTLRF V-QSLPKRER I-PFTEK--- ----FKN--- --------AD PLA------- ---LDLLERM L--------- ---------- --IFDPRKRI NAPQALEHEY
   289694  FSIITELLG- ----TPPDEV IQTI------ ---------- --CSENTLRF V-QSLPKRER V-PFSQK--- ----FRN--- --------QD PQA------- ---LDLLEKM L--------- ---------- --VFDPRTRI TAAEALSHPY
   137978  FSIITELLG- ----TPNDDM VSTI------ ---------- --CSENTLRF V-KNLPKRDP I-PFSER--- ----FVG--- --------QH PDA------- ---IDLLEKM L--------- ---------- --TFDPRKRI TAEEALAHPY
    19116  FSIITELLG- ----TPSDDV IDTI------ ---------- --CSENTLRF V-KSLPVMTK V-PFSTR--- ----FAG--- --------AD PDA------- ---VDLLEKM L--------- ---------- --VFDSRKRI TAAEALSHPY
   129801  FSIITELLG- ----SPPADV IDTI------ ---------- --CSENTLKF V-QSLPHKEP V-PFAEY--- ----FAS-NE --------ME PEA------- ---IDLLAKM L--------- ---------- --VFDPKKRI SAEEALSHPY
    80382  FSIITKLLG- ----SPPPDV IDTI------ ---------- --CSENTLRF V-KSLPHRDP V-SFSER--- ----FKG--- --------VD PDA------- ---IDLLSKM L--------- ---------- --VFDPKKRI TAAEALEHKY
   141916  FSIITKLLG- ----SPPPDV IDTI------ ---------- --CSENTLRF V-KSLPHRDP V-SFSER--- ----FKG--- --------VD PDA------- ---IDLLSKM L--------- ---------- --VFDPKKRI TAAEALEHKY
   187748  FSIITELLG- ----SPPPDV IDTI------ ---------- --CSENTLRF V-QSLPHRDP I-PFSER--- ----FAQCTH --------VE PEA------- ---IDLLAKM L--------- ---------- --IFDPKKRI SAADALQHPY
   168984  FSIITELLG- ----TPPADV IDTI------ ---------- --ASENTLRF V-KSLPHRDP V-PLTER--- ----FKG--- --------VE PEA------- ---IDLLEKM L--------- ---------- --VFDPKKRI TAAEILEHPY
    71808  FSIITELLG- ----TPPDDV IQTI------ ---------- --GSENTLRF V-QMLPHREP V-SFRAR--- ----FPH--- --------ID PFA------- ---VDLLERM L--------- ---------- --VFDPRKRI TAAEALAHPY
      432  FNIITELLG- ----TPPPDV IYTI------ ---------- --CSKNTLRY I-QSIPERKP V-PLKTR--- ----FTD--- --------ID DDS------- ---VDLLEKM L--------- ---------- --VFNPKARI SAADALAHPL
    59395  FSIITELLG- ----TPPADV IQTI------ ---------- --GSENTLRF V-QSLPRRTK I-PFPQR--- ----FAH--- --------AD PLA------- ---VDLLERM L--------- ---------- --VFDPRKRI KAAEALAHPF
     2865  FSIITELLG- ----TPPDDV IQTI------ ---------- --CSENTLRF V-QSLPKRER Q-PLTNR--- ----FKN--- --------AD PQA------- ---IDMLERM L--------- ---------- --VFDPRKRI RAGEALAHPY
   189766  FSIITELLG- ----TPPDDV IQTI------ ---------- --CSENTLRF V-QSLPKRER Q-PLANK--- ----FRN--- --------AE AEA------- ---IDLLESM L--------- ---------- --VFDPRKRV KATEALAHPY
    88183  FSIITELLG- ----TPPDDV IQTI------ ---------- --ASENTLRF V-QSLPKRER V-PFNKK--- ----FTN--- --------AD PLA------- ---LDLLEKM L--------- ---------- --VFDPRARI NATDALANEY
   216492  FSIITELLG- ----TPPDDV IQTI------ ---------- --CSENTLRF V-RSLPKRDR V-PFSQK--- ----LRT--- --------AD PDA------- ---LDLLERM L--------- ---------- --VFDPRKRI DATQSLAHSY
   188879  FSIITELLG- ----TPPDDV IQTI------ ---------- --CSENTLRF V-RSLPKRDR V-PFSQK--- ----LRT--- --------AD PDA------- ---LDLLERM L--------- ---------- --VFDPRKRI DATQSLAHSY
   182850  FSIITELLG- ----TPPDDV IQTI------ ---------- --CSENTLRF V-RSLPKRDR V-PFSQK--- ----LRT--- --------AD PDA------- ---LDLLERM L--------- ---------- --VFDPRKRI DATQSLAHSY
   174697  FSIITELLG- ----TPPDDV IQTI------ ---------- --CSENTLRF V-RSLPKRDR V-PFSQK--- ----LRT--- --------AD PDA------- ---LDLLERM L--------- ---------- --VFDPRKRI DATQSLAHSY
   934899  FSIITELLG- ----TPPDDV IETI------ ---------- --ASENTLRF V-QSLPKRER V-PFSQK--- ----LRT--- --------ND KDA------- ---LDLLEKM L--------- ---------- --VFDPRKRI DAATSLMHEY
   362397  FSIITELLG- ----TPPDDV IQTI------ ---------- --ASENTLRF V-QSLPKREK V-PFTTK--- ----FPN--- --------AD PLA------- ---LDLLEKM L--------- ---------- --VFDPRTRI SATEALAHEY
   416973  FSIITELLG- ----TPPDDV IESI------ ---------- --ASENTLRF V-QSLPKRER I-PFNQK--- ----LRT--- --------SD PDA------- ---LDLLEKM L--------- ---------- --VFDPRKRI DATQSLAHEY
   551386  FSIITELLG- ----TPPDDV IATI------ ---------- --CSENTLRF V-QSLPKRER V-PFGQK--- ----FRT--- --------QD PVA------- ---LDLLERM L--------- ---------- --VFDPRKRI TAAESLAHEY
    15239  FSIITELLG- ----TPPDDV IATI------ ---------- --CSENTLRF V-QSLPKRER V-PF------ ---------- ---------- --A------- ---LDLLERM L--------- ---------- --VFDPRKRI TAAESLAHEY
   497504  FSIITELLG- ----TPPDDV IHTI------ ---------- --ASENTLRF V-QSLPKRER Q-PLKDK--- ----FRN--- --------AD PAA------- ---IDLLEDM L--------- ---------- --VFDPKKRV KAADALAHEY
  1117682  FTLITELLG- ----TPPDDV IRTI------ ---------- --SSANTLRF V-KNMPHRER W-PFSQR--- ----FET--- --------KD PVA------- ---LDLLEKM L--------- ---------- --VFNPKKRI AAADALSHEY
   252496  FSIITELLG- ----TPPDDV IETI------ ---------- --CSENTLRF V-QSLPKRQR V-PFSQK--- ----LRT--- --------TD PDA------- ---LDLLERM L--------- ---------- --VFDPRKRI NASESLAHAY
   148867  FSIITELLG- ----SPPESM VSTI------ ---------- --CSENTLKF V-KSLPPREP K-PFAEA--- ----FPG--- --------ID PLA------- ---LDLLSKL L--------- ---------- --VFDSRERY TASEALAHPY
   423027  FYLITDALG- ----NPPDEV IERI------ ---------- --CTKAALNI V-KSLPKRQP A-PWATL--- ----FPD--- --------SD EDA------- ---IDLLAKM L--------- ---------- --IFDPDKRI SAAKALEHPY
  1104996  FFLITDALG- ----NPPDEV IERI------ ---------- --CTKTTLDL V-KSLPKRQP A-PWATL--- ----FPD--- --------SD ENA------- ---IDLLGEM L--------- ---------- --IFDPDKRI SAAKALEHPY
    29266  FSIITELLG- ----TPPDDV INTI------ ---------- --ASENTLRF V-KSLPKRER Q-PLTNK--- ----FKN--- --------AD EPA------- ---IDLLERM L--------- ---------- --VFDPKKRI TAAEALAHEY
   171653  FSIITELLG- ----TPPDDV INTI------ ---------- --ASENTLRF V-KSLPKRER Q-PLKNK--- ----FKN--- --------AE PAA------- ---IDLLERM L--------- ---------- --VFDPKKRI TAADALAHEY
   117063  FSIITELLG- ----TPPDDV IHTI------ ---------- --ASENTLRF V-QSLPKRER Q-PLKDK--- ----FRN--- --------AD PAA------- ---IDLLEDM L--------- ---------- --VFDPKKRV KAADALAHEY
   158104  FSIITELLG- ----TPPDDV IQTI------ ---------- --CSENTLRF V-QSLPKRDR Q-PLAQK--- ----FKN--- --------AD PLA------- ---IDLLENM L--------- ---------- --VFDPKKRV RAGDALAHPY
   160940  FSIITELLG- ----TPPDDV IQTI------ ---------- --CSENTLRF V-QSLPRRER Q-PLSNK--- ----FNN--- --------AD PLA------- ---IDLLESM L--------- ---------- --VFDPKKRV RAESALAHPY
     2813  FSIITELLG- ----TPPDDV IQTI------ ---------- --CSENTLRF V-QSLPKRER Q-PLTNR--- ----FKN--- --------AD PQA------- ---IDMLERM L--------- ---------- --VFDPRKRI RAGEALAHPY
   129541  FSIITELLG- ----SPPEEV IKTI------ ---------- --CSANTLQF V-QSLPKRER Q-PLSQK--- ----FKD--- --------AD PLA------- ---VDLLEKM L--------- ---------- --VFDPKQRI TAANGLAHEY
   257371  FSIITELLG- ----TPPDDV IQTI------ ---------- --CSENTLRF V-QSLPKRER I-PLSQK--- ----FKN--- --------AE PLA------- ---IDLLEKM L--------- ---------- --VFDPRKRI TAADALAHPY
   389086  FTIITQVLG- ----NPPQEV VERV------ ---------- --YSRNTLKF L-ESLPPREP H-PLSSF--- ----FTG--- --------AE EEV------- ---VDLIEKM L--------- ---------- --QLDPYKRI TATDALSHPY
    73401  FYLIIDLLG- ----NPPDEV IDRI------ ---------- --CSKNTVQI V-KSLGDRQP R-CLRTM--- ----FSS--- --------VD EAA------- ---VDLLDKF L--------- ---------- --VFDPHARI SASNALEHRY
  1139913  FYLIIDLLG- ----NPPDGV IDRI------ ---------- --CSKNTVQI V-KSLGKRQS R-CLKTM--- ----FSG--- --------VD EAA------- ---VDLLEKF L--------- ---------- --IFDLHARI SASNALEHRY
  1168371  FYLIIDLLG- ----NPPDGV IDRI------ ---------- --CSKNTVQI V-KSLGKRQS R-CLKTM--- ----FSG--- --------VD EAA------- ---VDLLEKF L--------- ---------- --IFDLHARI SASNALEHRY
   698519  FYLIIDLLG- ----NPPDEV IDRI------ ---------- --CSKNTVQI V-KSLGDRQP R-CLRTM--- ----FSS--- --------VD EAA------- ---VDLLDKF L--------- ---------- --VFDPHARI SASNALEHRY
   332332  FSIITELLG- ----SPPEEV IKTI------ ---------- --CSANDLAD M-LLV----- ---------- ---------- --A------- ---VDLLEKM L--------- ---------- --VFDPKQRI SAADGLAHEY
   243292  FSIITELLG- ----TPPDDV ISTI------ ---------- --CSENTLRF V-QSLPKRER Q-PLKNK--- ----FKN--- --------AD PQA------- ---IELLERM L--------- ---------- --VFDPRKRV KAGEALADPY
   243775  FSIITELLG- ----TPPDDV ISTI------ ---------- --CSENTLRF V-QSLPKRER Q-PLKNK--- ----FKN--- --------AD PQA------- ---IELLERM L--------- ---------- --VFDPRKRV KAGEALADPY
    24163  FSIITELLG- ----TPPDDV ISTI------ ---------- --CSENTLRF V-QSLPKRER Q-PLKNK--- ----FKN--- --------AD PQA------- ---IELLERM L--------- ---------- --VFDPRKRV KAGEALADPY
  1431451  FSIITELLG- ----TPPDDV IHTI------ ---------- --CSENTLRF V-QSLPKRER Q-PLSEK--- ----FKN--- --------AD PLA------- ---IDLLEHM L--------- ---------- --VFDPKKRI TAEAALAHEY
    99398  FSIITELLG- ----SPPEEV IKTI------ ---------- --CSANTLQF V-QSLPKRER Q-PLSQK--- ----FKD--- --------AD PLA------- ---VDLLEKM L--------- ---------- --VFDPKQRI SAADGLAHEY
   171665  FSIITELLG- ----TPPDDV INTI------ ---------- --ASENTLRF V-KSLPKRER Q-PLKNK--- ----FKN--- --------AE PAA------- ---IDLLERM L--------- ---------- --VFDPKKRI TAADALAHEY
    15876  FTIITQVLG- ----NPPQEV VERV------ ---------- --YSRNTLKF L-ESLPPREP H-PLSSF--- ----FTG--- --------AE EEV------- ---VDLIEKM L--------- ---------- --QLDPYKRI TATDALSHPY
    43014  LSLTLEILG- ----TPSLEK RRNF------ ---PQMFPN- --ANPLAVDL MERCLTFSPR K-RITAE--- ----DALAHP YLEPYHDPED EPT------- ---ASPLDPS FFS------- ---------- --FDYTKEQL SRSQLKQLIY
    43498  LSLTLEILG- ----TPSLD- ---------- ----DFYAI- --SSTRSRDY I-RALPFRKR R-NFPQM--- ----FPNANP LAVDLMPTET YHC------- ---RRCFGSS LFG------- ---------- --TLS
  1148336  LTLILDVLG- ----TPTMED YYGI------ ---------- --KSRRAREY I-RSLPFKKK I-PFRAM--- ----F----- ------PKSN ELA------- ---LDLLEKL L--------- ---------- --AFNPAKRI TVEEALRHPY
   220391  LDLILDVIG- ----TPSLDE FYAI------ ---------- --TSRRSRDY I-RALPIRKK R-PFPTL--- ----F----- ------PQAS AEA------- ---IDFLSKT L--------- ---------- --TFDPKKRM AVDEALEHPY
   774567  LDLILDVIG- ----TPTLEE FYSI------ ---------- --TSRRSRDY I-RALPIKKR K-SFSAL--- ----F----- ------PKAS PEA------- ---LDFLRKT L--------- ---------- --TFDPKKRL TVDQALEHPY
   138915  LDLILDVIG- ----TPTLEE FYSI------ ---------- --TSRRSRDY I-RALPIKKR K-SFSAL--- ----F----- ------PKAS PEA------- ---LDFLRKT L--------- ---------- --TFDPKKRL TVDQALEHPY
   757677
   313819
   290180
    12232
   865310
   312015
   500478
   500460
   243933
    64065
     2938
   196733
   145976
   501440
    71512
   543038
    99402
  1431403
   342880
   417966
Consensus  l..Il..lG      tP.... ...i.....                 .......% i...lp.... . pf..l   . .......        ..a. p.a           .Dl$.k. L                                .fdP.kRi tve.aL.Hpy

           1201                                                                                                                                                    1350
     6423  LAIW------ ----HDAS-D EPACPT-PFD F-GFEVVDDV --GQMRQMIL EE-------- ---VRNFRNS VRAPQQMQYS ----QGMQQ- ---------- ---------- ---------- ---G--GQQV PIPQGYDA-- ----------
   160078  LSIW------ ----HDPS-D EPSCPQ-TFD F-AFEVVEDV --PEMRQMIL EE-------- ---VRQFRHA VRTPQHLQYQ ----QGIQQ- ---------- ---------- ---------- ---A--GQPV PIPQSYDP-- ----------
   116650  LAIW------ ----HDAS-D EPKCPT-TFD F-GFEVVDDV --QDMRQMIL EE-------- ---VRRFRHS VRQPQQYAHA ----QQQGQ- ---------- ---------- ---------- ---Q---QQV PIPDNYDP-- ----------
   377992  LSIW------ ----HDAS-D EPTCPT-PFD F-GFEAAEEI --PEMRQMIL AE-------- ---VAQFRQS VRAPQSAPLG ----QGQGA- ---------- ---------- ---------- ---PAPGGQV PIPDQFPQ-- ----------
   289793  LHIW------ ----HDAS-D EPSCPT-TFD F-HFEVVEEI --PEMKRMIL QE-------- ---VQKFRQI VRVQPQVG-- ----APGQA- ---------- ---------- ---------- -----TQPQV PIPDNYDP-- ----------
   670269  LHIW------ ----HDAS-D EPNCPT-TFD F-HFEVVEDI --AEMKKMIL AE-------- ---VNRFRSM VRVQPQQGQI ----ANPMQ- ---------- ---------- ---------- -----QAGNV PIPEHYDP-- ----------
    32808  LQIW------ ----HDAS-D EPSCPT-TFD F-QFEVVEEI --PEMKKMIL DE-------- ---VSRFRQM VRVQPGAG-- ----AGTAQ- ---------- ---------- ---------- ------APQV PIPNNYDR-- ----------
   156295  LQIW------ ----HDAS-D EPSCPT-TFD F-QFEVVEEI --PEMKKMIL DE-------- ---VSRFRQM VRVQPGAG-- ----AGTAQ- ---------- ---------- ---------- ------APQV PIPNNYDR-- ----------
    22314  LQIW------ ----HDAS-D EPSCPT-TFD F-QFEVVEEI --PEMKKMIL DE-------- ---VSRFRQM VRVQPGAG-- ----AGTAQ- ---------- ---------- ---------- ------APQV PIPNNYDR-- ----------
   417978  LQIW------ ----HDPS-D EPSCPT-TFD F-QFEVVDEI --PDMKRMIL DE-------- ---VNRFRQM VRVQPSAG-- ----AGQPQ- ---------- ---------- ---------- -----PGQQV PIPNNYDR-- ----------
   414783  LQIW------ ----HDAS-D EPVCPQ-TFD F-HFEVVEDI --QQMKKMIL EE-------- ---VRLFRQS VRVGSGGGMG ----GPDMSV Q--------- ---------- ---------- ---QGQQQSV PIPENYDR-- ----------
  1074946  LHIW------ ----HDAS-D EPECKT-TFD F-DFEVVDDV --GEMRKMIL DE-------- ---VYRFRQF VRTVPGSAQA ----GSQAA- Q--------- ---------- ---------- ---QAAAGQV PIPSG-HG-- ----------
   865350  LHIW------ ----HDAS-D EPDCPT-TFN F-DFEVVEDV --GEMRKMIL EE-------- ---VYRFRQL VRTVPGATQA ----GSQAA- Q--------- ---------- ---------- ---QAAAGQV PIPSG-QA-- ----------
  1656563  LHIW------ ----HDAS-D EPDCPT-TFN F-DFEVVEDV --GDMRKMIL DE-------- ---VYRFRQL VRTVPGATQA ----GTQAA- G--------- ---------- ---------- ---QQGGGQV PIPTGGNQ-- ----------
   333276  LQIW------ ----HDAS-D EPDCPT-HFN F-DFEVVEDV --GEMRKMIL EE-------- ---VFRFRQT VRTVPGG--A ----GNQHG- A--------- ---------- ---------- ---QAAAGQV PMPSGGQQ-- ----------
   422586  LQIW------ ----HDAS-D EPDCPT-TFN F-DFEVVDEV --GDMRKMIM EE-------- ---VFRFRQH VRTDPSTA-S ----QIPGG- Q--------- ---------- ---------- ---PTQAGQV PMPEAGK--- ----------
   342887  LKIW------ ----HDET-D EPDCPT-TFN F-DFEVVDDV --GDMRKMIL DE-------- ---VYRFRQQ VRTAPAGG-A ----PGVAG- Q--------- ---------- ---------- ---QPGAGQV PMPSGQG--- ----------
   386771  LQIW------ ----HDAS-D EPDCPT-TFN F-DFEVIDDV --GHMREMIK EE-------- ---VFRFRQL VRTAPGSI-G ----Q-TGG- P--------- ---------- ---------- ---QAAAGQV PMPSGGNS-- ----------
    14976  LHIW------ ----HDAS-D EPDCPT-TFN F-DFEVIDDV --GHMREMIK DE-------- ---VLRFRQQ VRTAPGSG-G ----Q-TTG- P--------- ---------- ---------- ---QAAAGQV PMPSGGNS-- ----------
   130981  LHIW------ ----HDAS-D EPGCPT-TFN F-DFEVVEDV --GEMRKMIL EE-------- ---VHRFRQH VRIQPGQ--- --------Q- A--------- ---------- ---------- ---PSQGPAV PIPQGGG--- ----------
   435286  LQIW------ ----HDAS-D EPGCPT-TFN F-DFEVVEDV --GEMRKMIL DE-------- ---VARFRQH VRIQPGQ--- --------Q- G--------- ---------- ---------- ---QNQGPTV PIPQAGG--- ----------
  1457372  LHIW------ ----HDAS-D EPDCPT-TFN F-DFEVVEDV --GDMRKMIL DE-------- ---VYRFRQL VRTVPGATQA ----GTQAA- G--------- ---------- ---------- ---QQGGGQV PIPTGGNQ-- ----------
```

```
 249807  LHIW------ ----HDAS-D EPTCPT-TFD F-HFEVVEDV --QEMRHMIY DE-------- ---VVRFRAL VRQQSQAQAA -----AQQQ- Q--------- ---------- ---------- ---IAQQTNV PIPDNQQG-- ----------
1142566  LHIW------ ----HDAS-D EPTCPT-TFD F-HFEVVEDV --QEMRHMIY DE-------- ---VVRFRAL VRQQSQAQAA -----AQQQ- Q--------- ---------- ---------- ---IAQQTNV PIPDNQQG-- ----------
1008492  LHIW------ ----HDAS-D EPTCPT-TFD F-HFEVVEDV --QEMRHMIY DE-------- ---VVRFRAL VRQQSQAQAA -----AQQQ- Q--------- ---------- ---------- ---IAQQTNV PIPDNQQG-- ----------
 152510  LSIW------ ----HDAS-D EPSCPT-PFD F-AFEVVDEV --PEMRKMIL GE-------- ---VLQFRQH VRETA--RHA ----MAGGQ- Q--------- ---------- ---------- ---GQAQTNV PIPDGHHG-- ----------
 336937  LSIW------ ----HDAS-D EPSCPT-PFD F-SFEVVEEV --SDMRKMIL DE-------- ---VVRFRAH VRQPSHVQAA ----MAAMQ- Q--------- ---------- ---------- ---GQAQTNV PIPDGHHG-- ----------
1649893  LQIW------ ----HDAQ-D EPNCPT-PFD F-SFEVVDEI --SDMRKMIL NE-------- ---VQNFRNA VRNKPVHVID ----IAALG- ---------- ---------- ---------- -----RKDSV GIPDHQWV-- ----------
 371270  LSIW------ ----HDVS-D EPECPA-TFD F-AFEVVEEV --PEMRQMIL DE-------- ---CQQFRNL VRRQPNAPTS ----LQLQQL Q--------- ---------- ---------- ---QEQQRKD SIGIPAGV-- ----------
  82103  LHIW------ ----HDAS-D EPSCPT-TFD F-QFEVVEEI --NDMKKMIL AE-------- ---VNRFRQM VRVQPGAGAG ----PAQ--- ---------- ---------- ---------- ------APQV PIPNNYDR-- ----------
  42859  LAIW------ ----HDPS-D EPACPT-KFD F-RFEVVKEI --PELRTIIF DE-------- ---VRRFRSE VRAPLHQQQQ ----QQQQQH YGDY------ ---------- ---------- ---QQQAGQV PMPDQWNP-- ----------
 212507  LQIW------ ----HDAS-D EPDCPT-TFN F-DFEVVDEV --GDMRKMIM EE-------- ---VFRFRQH VRTDPSTASQ ----IPGGQ- ---------- ---------- ---------- ---PTQAGQV PMPEAGKQ-- ----------
 212502  LQIW------ ----HDAS-D EPDCPT-TFN F-DFEVVDEV --GDMRKMIM EE-------- ---VFRFRQH VRTDPSTASQ ----IPGGQ- ---------- ---------- ---------- ---PTQAGQV PMPEAGKQ-- ----------
 461664  LQIW------ ----HDAS-D EPGCPT-TFN F-DFEVVEDV --GEMRKMIL DE-------- ---VARFRQH VRIQPGQQGQ ----NQG--- ---------- ---------- ---------- ---PT----V PIPQAGGS-- ----------
  39613  SKLW------ ----HDPV-D EMECPI-PFD F-SFETINDM --DKSREIIS NE-------- ---VIDMRNE VSGARRPSQL ----DFTAT- ---------- ---------- ---------- ----TAVSVT GTAASTEY-- ----------
  91135  LKVW------ ----HDPN-E EISCET-KFD F-GFEEINDL --DEMRSLIV EE-------- ---VRSFRES VRIPQTPVNS ----DPKSQI QPQS------ ---------- ---------- ---QPQSQDE IPDITHQQ-- ----------
 103403  LNVW------ ----HDPS-D EPVCTS-KFD F-SFESVEDS --AVMRQMII DE-------- ---VQTFRQS VR---APIEE ----Q----- ---------- ---------- ---------- ---------- ----TNLQ-- ----------
 112360  LKVW------ ----HDPA-D EPICAS-KFD F-TFEEVDDL --DVMRTMIL EE-------- ---VESFREF VR---KPIDE ----Q----- --QL------ ---------- ---------- ---ELSNPEA MGHGQHQQ-- ----------
   4266  LEVW------ ----HDPN-D EPVCPR-KFD F-SFESVDDI --HDMKKMIV DE-------- ---VAGFREY VR---KPLEE ----QERIY- --QE------ ---------- ---------- ---EQQREEQ VNQTSLQQ-- ----------
 168486  LAVW------ ----HDPQ-D EPECQM-KFD FKSFETVDDI --DSMKTLII DE-------- ---VKSFREF VR---KPIHE ----QQQIQL QQQL------ ---------- ---------- ---QQQHHQQ QHQHQQQQ-- ----------
  35227  LEVW------ ----HDPN-D EPVCAA-KFD F-SFEETDDI --NEMKQMII EE-------- ---TRSFREY VR---RPLEE ----QTIV-- --SE------ ---------- ---------- ---QYGATGA YEMDQPQQ-- ----------
  67581  LEVW------ ----HDPN-D EPVCAA-KFD F-SFEETDDI --NEMKQMII EE-------- ---TRSFREY VR---RPLEE ----QTIV-- --SE------ ---------- ---------- ---QYGATGA YEMDQPQQ-- ----------
  59367  LSIW------ ----HDPN-D EPVCAS-TFD F-GFEVVNDL --EDLKLMVV EE-------- ---VQDFRNY VR--QPLLDQ ----QHEEEE EAQQ------ ---------- ---------- ---QQQQSMQ NLNSNNSN-- ----------
 336175  LSAY------ ----HDED-D EPSHPQ-VFD F-SFEVLESM --EDMRKMIA QE-------- ---VMQFKAQ K-QGLPPLQL ----NTATN- ---------- ---------- ---------- -----LRRKD SLSAGDRE-- ----------
 177826  LSAY------ ----HDVD-D EPVHTE-TFD F-SFEVVDSI --EDMRKMIA QE-------- ---VMSFKAN K-QGL--LQP ----QPSVS- ---------- ---------- ---------- -----LRRKE SLSTQDRE-- ----------
 212434  LSAY------ ----HDED-D EPSHTE-TFD F-SFEVVDSI --EDMRKMIA QE-------- ---VMSFKAN K-QGL--MPP ----QAGAN- ---------- ---------- ---------- -----LRRKE SLSAQDRE-- ----------
 164796  LSAY------ ----HDED-D EPSHTE-TFD F-SFEVVDSI --EDMRKMIA QE-------- ---VMSFKAN K-QGL--MPP ----QAGAN- ---------- ---------- ---------- -----LRRKE R
 100890  LKAH------ ----HNFD-D EISCPV-SCD M-SFETVTSM --PDVKRMII EE-------- ---VIDFKRQ AVQETHEAAN ----IDISA- ---------- ---------- ---------- -----YLEQQ NVSSGGLD-- ----------
 288633  LQLF------ ----HDPE-D EPSHYA-TVD F-SFESLQTT --EEMRETII EE-------- ---VKMFKAR KHSLKIDMKG ----LRRQN- ---------- ---------- ---------- -----SITTP TSKRAKYE-- ----------
  69432  FAVW------ ----HDPA-D EPVCPS-TFD F-SFEEEDTI --EGMKKLIV EE-------- ---VRSFRTE VRNQAKASSQ --IRRQDS-- ---------- ---------- ---------- ---LPIPSRE EI-INSPV-- ----------
  60279  FQVW------ ----HDPS-D EPICDS-KFD F-GFEEEDSI --EGMKKLIV NE-------- ---VNGFRAE VRAQARAANQ --MRRQDS-- ---------- ---------- ---------- ---LPIPSRD EI-ISSPV-- ----------
 166223  LQVW------ ----HDPA-D EPVCEQ-KFD F-SFEEEDSI --DGMKKLIV EE-------- ---VRDFRAE VRHQARGGSQ --MRRQDS-- ---------- ---------- ---------- ---LPIPTRD DI-LASPV-- ----------
1111368  LQVW------ ----HDPA-D EPICEA-KFD F-SFEEEDSI --EGMKKLIV DE-------- ---VNSFRAE VRQPARAAGQ --IRRQDS-- ---------- ---------- ---------- ---LPIPSRD EI-ISSPV-- ----------
7180514  LQVW------ ----HDPA-D EPVCDT-KFD F-GFEEEDSI --DGMKKLII AE-------- ---VNTFRAE VRAQARAAGQ --IRRQDS-- ---------- ---------- ---------- ---LPIPSRD EI-LSSPV-- ----------
 671172  LQVW------ ----HDPA-D EPVCDT-KFD F-GFEEEDSI --DGMKKLII DE-------- ---VNSFRAE VRAQARAAGQ --IRRQDS-- ---------- ---------- ---------- ---LPIPSRD EI-LSSPV-- ----------
  69595  LQVW------ ----HDPA-D EPTCDS-KFD F-GFEEEDSI --DGMKRLII AE-------- ---VNSFRAE VRAQARAAGQ --IRRQDRFV M-SL------ ---------- ---------- ---LPIPSRD EI-LSSPV-- ----------
 188428  LQVW------ ----HDPA-D EPTCDS-KFD F-GFEEEDSI --DGMKRLII DE-------- ---VNSFRAE VRAQARAAGQ --IRRQDS-- ---------- ---------- ---------- ---LPIPSRD EI-LSSPV-- ----------
 799680  LQVW------ ----HDPA-D EPVCET-KFD F-SFEEEDSI --EGMKKLIV EE-------- ---VNSFRAE VRAQARAAGQ --IRRQDS-- ---------- ---------- ---------- ---LPIPSRD EI-LSSPV-- ----------
 178052  LQVW------ ----HDPA-D EPVCEY-KFD F-SFEEEDSI --EGMKKLIV DE-------- ---VNSFRAE VRAQARAAGQ --IRRQDS-- ---------- ---------- ---------- ---LPIPSRD DI-INSPV-- ----------
 129301  LQVW------ ----HDPA-D EPVCEK-KFD F-SFEEEDNI --EGMKKLIV EE-------- ---VNLFRAE VRAQARAAGQ --IRRQDS-- ---------- ---------- ---------- ---LPIPSRD DI-INSPV-- ----------
 208817  LQVW------ ----HDPA-D EPICET-KFD F-NFEEEDNI --EGMKKLII EE-------- ---VNSFRAE VRAQARASGQ --IRRQDS-- ---------- ---------- ---------- ---LPIPSRD DI-INSPM-- ----------
 424444  LQVW------ ----HDPA-D EPVCDT-KFD F-GFEEEDGI --DGMKKLIV DE-------- ---VNAFRAE VRAQARAAGQ --VRRQDS-- ---------- ---------- ---------- ---LPIPSRD DL-LNSPM-- ----------
 830405  LQVW------ ----HDPA-D EPVCES-KFD F-GFEEEDSI --EGMKKLIV EE-------- ---VNQFRAE VRAQARASNQ --IRRQES-- ---------- ---------- ---------- ---LPIPSRE EI-LNSPM-- ----------
 892596  LQVW------ ----HDPA-D EPLCET-KFD F-AFEEEDTI --DGMKKMIV EE-------- ---VNSFRAE VRQQARSASN --MRRQDS-- ---------- ---------- ---------- ---LPIPSRD EI-IASPV-- ----------
 186189  LQVW------ ----HDPA-D EPVCPT-PFD F-SFEDEDAI --EGMKRLIV EE-------- ---VQSFRAE VRHQARAGGQ --IRRNDS-- ---------- ---------- ---------- ---LPIPSRE EI-QNSPT-- ----------
 164780  LAVW------ ----HDPT-D EPVCEK-KFD F-SFEEEDSI --EGMKRLIV EE-------- ---VNTFRAL VRSQARAAGQ --MRRQES-- ---------- ---------- ---------- ---LPIPTRE EI-MNSPV-- ----------
 167312  LAVW------ ----HDPA-D EPVCAS-SFD F-GFEEEDSI --EGMKRLIV EE-------- ---VHAFRAE VRAQARAAGQ --IRRQES-- ---------- ---------- ---------- ---LPMPTRD EI-LNSPA-- ----------
 119588  LAVW------ ----HDPA-D EPVCAS-SFD F-GFEEEDSI --EGMKRLIV EE-------- ---VHAFRAE VRAQARAAGQ --IRRQES-- ---------- ---------- ---------- ---LPMPTRD EI-LNSPV-- ----------
 423592  LAVW------ ----HDPS-D EPVCPT-QFD F-GFEDEDSI --EGMKKLIV EE-------- ---VNHFRAE VRAHARASGQ --IRRQDS-- ---------- ---------- ---------- ---LPIPSRD DI-IASPI-- ----------
 474667  LAVW------ ----HDPA-D EPNCDS-IFD F-SFEEEDSI --EGMKRLIV EE-------- ---VNNFRAE VRAQARGSAQ --NHRSTKDS D-N------- ---------- ---------- ---LGIPSRD EI-ISSPV-- ----------
 459613  LAVW------ ----HDPA-D EPNCDS-IFD F-SFEEEDSI --EGMKRLIV EE-------- ---VNNFRAE VRAQARGSAQ --NHRSTKDS D-N------- ---------- ---------- ---LGIPSRD EI-ISSPV-- ----------
 156206  FQVW------ ----CDPA-D EPVCHS-TFD F-GFENEDSM --DGMKRLIV DE-------- ---VNSFRAE IRSQVRVEGQ LEIHRQDG-- --SL------ ---------- ---------- ---PPIPSRD EI-LSSPV-- ----------
 791811  LAVW------ ----HDPA-D EPVCPT-PFD F-GFEEEDSI --EGMKRLIV EE-------- ---VHAFRAE VRAQARAAGQ --IRRQDS-- ---------- ---------- ---------- ---LPMPTRE EL-LNSPE-- ----------
 146905  LAVW------ ----HDPS-D EPVCEK-KFD F-GFEEEDSV --EGMKRLIV NE-------- ---VNVFRSL VRTQARAAGQ --GRRQDS-- ---------- ---------- ---------- ---LPIPSRD DI-IASPT-- ----------
 715329  LAVW------ ----HDPA-D EPICNE-TFD F-GFEEEDTI --DGMKQLIV EE-------- ---VREFRAE VRQQARASGQ --IRRQDS-- ---------- ---------- ---------- ---LPIPTRE EI-LASPV-- ----------
 511921  LAVW------ ----HDAA-D EPVCSQ-PFD F-GFEDVDDM --PGMKALIV QE-------- ---VNTFRAL VRSQARQQQQ --ARKQDS-- ---------- ---------- ---------- ---LPIPSRD DI-LASPV-- ----------
 737951  LAVW------ ----HDPA-D EPICPT-PFD F-SFEEEDSI --DGMKRLIV SE-------- ---VQTFRAE VRAQARAAGQ --IRRQDS-- ---------- ---------- ---------- ---LPIPTRE EI-LSSPV-- ----------
 545584  LAVW------ ----HDTA-D EPVCSQ-PFD F-GFEDVDDM --PGMKALIV QE-------- ---VNTFRAL VRSQARQQQQ --ARKQDS-- ---------- ---------- ---------- ---LPIPSRD DI-LASPV-- ----------
 102765  LAVW------ ----HDTA-D EPLCSQ-TFD F-GFEEVDDM --PGMKQLII EE-------- ---VNSFRAL VRSQARQQLQ --ARKQDS-- ---------- ---------- ---------- ---LPIPSRD DI-LASPV-- ----------
 203555  FRVW------ ----HLPP-D EPICNR-ACD F-SFEDENEV --EGMKALIQ HE-------- ---VNMFQKE VRAREQARQV AQLRPVDS-- ---------- ---------- ---------- ---LPVPSRD DI-LRSPV-- ----------
 302829  LQVW------ ----HDPA-D EPVCDT-KFD F-GFEEEDSI --DGMKKLII DE-------- ---VNSFRAE VRAQARAAGQ --IRRQDS-- ---------- ---------- ---------- ---LPIPSRD EI-LSSPV-- ----------
 701651  LAVW------ ----HDPA-D EPVCPT-PFD F-GFEEEDSI --EGMKRLIV EE-------- ---VHAFRAE VRAQARAAGQ --IRRQDS-- ---------- ---------- ---------- ---LPMPTRE EL-LNSPE-- ----------
 972433  LQVW------ ----HDPA-D EPICEA-KFD F-SFEEEDSI --EGMKKLIV EE-------- ---VTSFRKE VRGQARAPGQ --MRRQDS-- ---------- ---------- ---------- ---LPIPSRD EI-IKSPV-- ----------
 931864  LQVW------ ----HDPA-D EPICEA-KFD F-SFEEEDSI --EGMKKLIV EE-------- ---VTSFRKE VRGQARAPGQ --MRRQDRRV VIPF------ ---------- ---------- ---LPIPSRD EI-IKSPV-- ----------
 813292  LQVW------ ----HDPA-D EPVCET-KFD F-SFEEEDSI --EGMKKLIV EE-------- ---VNSFRAE VRAQARAAGQ --IRRQDS-- ---------- ---------- ---------- ---LPIPSRD EI-LSSPV-- ----------
  78187  LQVW------ ----HDPA-D EPTCDS-KFD F-GFEEEDSI --DGMKRLII DE-------- ---VNSFRAE VRAQARAAGQ --IRRQDS-- ---------- ---------- ---------- ---LPIPSRD EI-LSSPV-- ----------
 406308  LHIW------ ----HDAS-D EPACPT-TFD F-HFEVVEDV --AQMRKMIY EE-------- ---VVNFRAA VRQQSQAHAA --A------- ---------- ---------- ---------- ----VAAQQQ QL-GNVPI-- ----------
 118382  LQVW------ ----HDPA-D EPVCDT-KFD F-GFEEEDSI --DGMKKLII DE-------- ---VNSFRAE VRAQARAAGQ --IRRQDR-- ---------- ---------- ---------- ---NTVLTTV QL-PLIPR-- ----------
 100328  LQVW------ ----HDPA-D EPVCPT-PFD F-SFEDEDAI --EGMKRLIV EE-------- ---VQSFRAE VRHQARAGGQ --IRRND
1411022  LAVW------ ----HDPN-D EPVCPR-KFD F-GFEEEDSM --EGMKKLIV EE-------- ---VNSFRAE VRRGRRRAES S-ARRQDS-- ---------- ---------- ---------- ---LPIPTRE EI-MASPV-- ----------
 741167  LAVW------ ----HDPN-D EPVCPT-KFD F-GFEEEDSM --EGMKRLII EE-------- ---VNSFRAQ VRSGRR-GSN A-ARRQES-- ---------- ---------- ---------- ---LPIPSRE EI-IASPV-- ----------
1338511  LAQW------ ----HDEF-Y EPTCES-IFD F-SFEDEDSM --EGMKKLIV EE-------- ---VMAFRRL VRQQATPASK --KRTQDDS- ---------- ---------- ---------- ---LAVPSRD DI-IASPI-- ----------
 756718  LAQW------ ----HDEF-Y EPNCDS-IFD F-SFEAEDAM --EGMKKLIV DE-------- ---VMAFRRL VRQQSQPAGK --KRGVDDS- ---------- ---------- ---------- ---LAVPSRD DI-IASPV-- ----------
 302653  LAVW------ ----HDPA-D EPVCPA-TFD F-GFEAEESS --EGMKAMII TE-------- ---VAAFRQQ VRNQIRAA-- ---------- ---------- ---------- ---------- ------PVRR ET-L--PV-- ----------
1311110  LSVW------ ----HDPA-D EPMCPS-KFD F-SFESEETI --PGMKKMII DE-------- ---VENFRRL VR----TPPE DQDGAEA--- ---------- ---------- ---------- ---VPKTADS -VPI--PS-- ----------
 722894  LAVW------ ----HDPI-D EIDCKD-KFD F-GFEAEETT --EGMKKLIV EE-------- ---VTNFRRM VRPNAPTRQN STSGREA--- ---------- ---------- ---------- ---LPIPSRE -IVMGSPT-- ----------
 288507  LSVW------ ----HDPQ-D EPICPT-KFD F-GFENVDDI --EGMKHLIL RE-------- ---VASFREE VRGR--ARMG QAKRQDS--- ---------- ---------- ---------- ---LPIPSRE AIQAASPS-- ----------
 757539  FQVW------ ----HDPS-D EPICDS-KFD F-GFEEEDSI --EGMKKLIV NE-------- ---VNGFRAE VRAQARAANQ --MRRQDR-- ---------- ---------- ---------- ---SVVIITL DIDLNTSC-- ----------
 184184  LQVW------ ----HDPA-D EPVCEY---- ---VQIRLLV --RGG-GFY
 917307  LQVW------ ----HDPA-D EPICEA-KFD F-SFEEEDSI --EGMKKLIV EE-------- ---VTSFRKE VRGQARAPGQ --MRRQDRRV VIP------- ---------- ---------- ---LPIPSRD EI-IKSPV-- ----------
6355211  LQVW------ ----HDPA-D EPVCDT-KFD F-GFEEEDSI --DGMKKLII AE-------- ---VNTFRAE VRAQARAAGQ --IRRQDS-- ---------- ---------- ---------- ---LPIPSRD EI-LSSPV-- ----------
 249241  LQVW------ ----HDPA-D EPTCDS-KFD F-GFEEEDSI --DGMKRLII AE-------- ---VNSFRAE VRAQARAAGQ --IRRQDS-- ---------- ---------- ---------- ---LPIPSRD EI-LSSPV-- ----------
 216822  LQVW------ ----HDPA-D EPTCDS-KFD F-GFEEEDSI --DGMKRLII DE-------- ---VNSFRAE VRAQARAAGQ --IRRQDS-- ---------- ---------- ---------- ---LPIPSRD EI-LSSPV-- ----------
 805839  LQVW------ ----HDPA-D EPVCET-KFD F-SFEEEDSI --EGMKKLIV EE-------- ---VNSFRAE VRAQARAAGQ --IRRQDS-- ---------- ---------- ---------- ---LPIPSRD EI-LSSPV-- ----------
 355172  LQVW------ ----HDPA-D EPVCDT-KFD F-GFEEEDSI --DGMKKLII AE-------- ---VNTFRAE VRAQARAAGQ --IRRQDS-- ---------- ---------- ---------- ---LPIPSRD EI-LSSPV-- ----------
 302825  LQVW------ ----HDPA-D EPVCDT-KFD F-GFEEEDSI --DGMKKLII DE-------- ---VNSFRAE VRAQARAAGQ --IRRQDS-- ---------- ---------- ---------- ---LPIPSRD EI-LSSPV-- ----------
 239059  LAVW------ ----HDPA-D EPNCDS-IFD F-SFEEEDSI --EGMKRLIV EE-------- ---VNNFRAE VRAQARGSAQ --NHRSTKDS D-N------- ---------- ---------- ---LGIPSRD EI-ISSPV-- ----------
 251409  LAVW------ ----HDPA-D EPICPT-PFD F-SFEEEDSI --DGMKRLIV SE-------- ---VQTFRAE VRAQARAAGQ --IRRQDS-- ---------- ---------- ---------- ---LPIPTRE EI-LSSPV-- ----------
 251443  LAVW------ ----HDPA-D EPICPT-PFD F-SFEEEDSI --DGMKRLIV SE-------- ---VQTFRAE VRAQARAAGQ --IRRQDS-- ---------- ---------- ---------- ---LPIPTRE EI-LSSPV-- ----------
  23484  LSAY------ ----HDED-D EPSHPQ-VFD F-SFEVLESM --EDMRKMIA QE-------- ---VMQFKAQ KQGLPPLQLN -----TATNL RRKD------ ---------- ---------- ---SLSAGDR ETLERSKE-- ----------
 306334  LATY------ ----HDPD-E EPSHAQ-MFD F-AFEKAASI --DEIRALIT RE-------- ---IVSWKAE RHGGPALPTV -----PVP-- ---------- ---------- ---------- -----VPAPQ PVQDIPPQ-- ----------
 306351  LATY------ ----HDPD-E EPSHAQ-MFD F-AFEKAASI --DEIRALIT RE-------- ---IVSWKAE RHGGPALPTV -----PVP-- ---------- ---------- ---------- -----VPAPQ PVQDIPPQ-- ----------
 148766  LASH------ ----HQPD-D EPTHDQ-IFD F-SFESVESM --DEMRNMIV QE-------- ---VIDFKKI LRA
  84676  LASH------ ----HQPD-D EPTHDQ-IFD F-SFESVESM --DEMRNMIV QE-------- ---VIDFKKI LRA
  74082  LHIW------ ----HDAS-D EPSCPT-TFD F-HFEVVEEI --PEMKRMIL QE-------- ---VQKFRQI VRVQPQVG-A PGQATQP--- ---------- ---------- ---------- -------QVP IPDN---Y-- ----------
  97664  LQIW------ ----HDAS-D EPSCPT-TFD F-QFEVVEEI --PDMKKMIL AE-------- ---VHRFRQM VRVQPGAG-A GGQGQQQN-- ---------- ---------- ---------- ------QQVP IPNN---Q-- ----------
  97676  LQIW------ ----HDAS-D EPSCPT-TFD F-QFEVVEEI --PDMKKMIL AE-------- ---VHRFRQM VRVQPGAG-A GGQGQQQN-- ---------- ---------- ---------- ------QQVP IPNN---Q-- ----------
 297954  LSIW------ ----HDVS-D EPECPA-TFD F-AFEVVEEV --PEMRQMIL DE-------- ---CQQFRNL VRRQPNAP-T SLQLQQLQ-- ---------- ---------- ---------- ------QEQQ RKDSIGIP-- ----------
 323749  LQIW------ ----HDAS-D EPDCPT-HFN F-DFEVVEDV --GEMRKMIL EE-------- ---VFRFRQT VRTVPGG--A GNQHGAQA-- ---------- ---------- ---------- ------AAGQ VPMPSGGQ-- ----------
1926181  LHIW------ ----HDAS-D EPDCPT-TFN F-DFEVVEDV --GEMRKMIL EE-------- ---VYRFRQL VRTVPGATQA GSQAAQQA-- ---------- ---------- ---------- ------AAGQ VPIPSG-Q-- ----------
1457364  LHIW------ ----HDAS-D EPDCPT-TFN F-DFEVVEDV --GDMRKMIL DE-------- ---VYRFRQL VRTVPGATQA GTQAAGQQ-- ---------- ---------- ---------- ------GGGQ VPIPTGGN-- ----------
  78435  LQIW------ ----HDAS-D EPDCPT-TFN F-DFEVIDDV --GHMREMIK EE-------- ---VFRFRQL VRTAPGS--I GQTGGPQA-- ---------- ---------- ---------- ------AAGQ PAGHSQFA
1457361  LHIW------ ----HDAS-D EPDCPT-TFN F-DFEVVEDV --GDMRKMIL DE-------- ---VYRFRQL VRTVPGATQA GTQAAGQQ-- ---------- ---------- ---------- ------GGGQ VPIPTGGN-- ----------
1328540  LSVW------ ----HDPA-D EPMCPS-KFD F-SFESEETI --PGMKKMII DE-------- ---VENFRRL VRTPPEDQDG ---------- ---------- ---------- ---------- -----AEAVP KTADSVPI-- ----------
1364342  LSVW------ ----HDPA-D EPMCPS-KFD F-SFESEETI --PGMKKMII DE-------- ---VENFRRL VRTPPEDQDG ---------- ---------- ---------- ---------- -----AEAVP KTADSVPI-- ----------
 350087  LQVW------ ----HDPA-D EPLCEV-PFD F-SFEQEDST --NGMRNLII QE-------- ---VRSFRQL VRRQAAPAPP ---------- ---------- ---------- ---------- -----RAHEL PPAPQPPQ-- ----------
 288508  LSVW------ ----HDPQ-D EPICPT-KFD F-GFENVDDI --EGMKHLIL RE-------- ---VASFREE VRGRARMGQA ---------- ---------- ---------- ---------- -----KRQDS LPIPSREA-- ----------
 283473  LAVW------ ----HDPA-D EPICNE-TFD F-GFEEEDTI --DGMKQLIV EEPSDPNARG DPGVARARAG VCGTGAGACG ---------- ---------- ---------- ---------- -----LAGDG RPECGAGA-- ----------
 734869  LAQW------ ----HDEF-Y EPNCDS-IFD F-SFEAEDAM --EGMKKLIV DE-------- ---VMAFRRL VRQQSQPAGK ---------- ---------- ---------- ---------- -----KRGV- --DDSLAV-- ----------
   9394  LKEV------ ----RNGD-D EPVLEK-PFD FSFENTATTM --TDLKVLIH EE-------- ---VEAFKKN PGCTIIPLVS ---------- ---------- ---------- ---------- ------IRRR EIELRQDV-- ----------
 167564  LRMV------ ----RDPS-D EPITPQ-VFD FQFENTAKTF --NDYKILIH EE-------- ---VELFKQA RLGSLS
 113741  LNAV------ ----RDYN-D EPDCTK-PFD FSFEGYATSI --TDLKLLIK DE-------- ---VEEFKIE RRTQGHVKGS ---------- ---------- ---------- ---------- ------P
   6575  LNAV------ ----RDYN-D EPDCTK-PFD FSFEGYATSI --TDLKLLIK DE-------- ---VEEFKIE RRTQGHVKGS ---------- ---------- ---------- ---------- ------P
  30703  LAEV------ ----RNLE-D EPEVAQ-PFD FSFEWKCRTV --NDMKILLH EE-------- ---VELFRKE RKATLK
  65523  LAEV------ ----RNLE-D EPEVAQ-PFD FSFEWKCRTV --NDMKILLH EE-------- ---VELFRKE RKATLK
   5078  FSTV------ ----RNID-E EPNTET-IFD FNFEHQASSI --EEMKIMLQ DE-------- ---VDQFRAQ VRKTSIKI
  54997  FSTV------ ----RNID-E EPNTET-IFD FNFEHQASSI --EEMKIMLQ DE-------- ---VDQFRAQ VRKTSIKI
 114746  FIKV------ ----KNAN-D EATCPYGPFD FQYEHNITSI --NDLTKLLF KE-------- ---VEEFKKA KIKNNTSI
 238776  LKQV------ ----KSQF-Q ESVCSK-PFD FNFENEAITQ --SDLKKVLQ QE-------- ---VDSFKLE RFRK
  71410  LSTY------ ----HDPD-D EPTCPTNFDK WKEIERLETI --EEFREALW NEIEDFRKEV R
 676425  LASY------ ----HDVT-D EPDCPETFER WRTLEALETL --EQYKEMLY NEIQDYRREV R
 676527  LASY------ ----HDVT-D EPDCPETFER WRTLEALETL --EQYKEMLY NEIQDYRREV RGIP
 152706  LASY------ ----HEET-D EPECPTKFEK WRDIEKLETI --AEFKEALW KEIEDYRMEV R
  88536  LASY------ ----HEET-D EPECPTKFEK WRDIEKLETI --AEFKEALW KEIEDYRMEV R
 122893  LASY------ ----HDID-D EPDCPVKFEK WRWIEELETI --DEFREALW DEIQDYRKEV R
 151264  LATY------ ----HDVE-D EPVCGKDFNR WREIEELETL --DDFRDALV NEVLECRKEV R
  67588  LAAY------ ----HDPI-D EPDCPYKFEK WREIEELETM --EDFREALW NEIEAYRREV R
 153824  LASY------ ----HEVG-D EPSCPEPFEK WREIEEH
 103584  LASY------ ----HEVG-D EPSCPEPFEK WREIEELDTI --EQFRVAIA REVAEFRREV RTIDVEEDVE I
 118819  LSTY------ ----HDPD-D EPTCPTNFDK WKEIERLETI --EEFREALW NEIEDFRKEV RGMNINMADM TNRTIDAAIN ASVQAYRARE PTGPADSLPS APITAPSYAT LPQNSHSQAS LEPGTHQTLS GSTTAVAD-- ----------
7227248  LSAY------ ----HDPL-D EPDCPEKFEK WREIEELKTM --EEFREALW KEIENYRREV RGID---LEL ----VLPPLP SSPA-VSPLS PS-------- ---------- -SILGTFEGN VS--KPLPCL SETNVVCD-- ----------
 240796  LAAY------ ----HDPV-D EPDCPVKFEK WREIEELETL --EEFREALW NEIEDYRREV RGLD---LEL S---ALPPLS SSPPPMSPPQ PT-------- ---------- LSSSVKFEEG IF--QNAK-N QEETGIIV-- ----------
 366893  LAAY------ ----HDPI-D EPDCPYKFEK WREIEELETM --EDFREALW NEIEAYRREV RGLDH--LEL S---ALPPLS VSPSSMSPLQ PEVVPQPPFP PPSHPPPAAP ITPAIKFEED VF--RRSP-S QSYAKPTE-- ----------
  79368  LSSY------ ----HDPA-D EPDCPAKFEK WREIEELETM --EDFRQALW DEIEDYRNQV RGLDLDIEEE D---EELPLS ASPPPVSEPP P--------- -PPPPPPVIE LQPAVVFEEG IF--NMSPKT QDTATEAP-- ----------
 119618  LASY------ ----HDIS-D EPECHQVFDR WQEIESLETI --DDFRDALW CEIEDYRREV RGLKLVSPKR T--LSTSSVR ELETTMPILR EE-------- ---------- -------REQ ERSVFEHDTI REEDSAEL-- ----------
```

```
 500495  LTSY------ ----HDIS-D EPDCPQKFER WREIERLNTL --EEFREALW NEIEDYRKEV RGIDTHSPDQ L--FRIPSIG GTRGGESPPQ PS-------- ---------- -------QAT LPAVPPDVLM PTASVDEN-- ----------
 449465  LTSY------ ----HDIS-D EPDCPQKFER WREIEKLNTL --EEFREALW NEIEDYRKEV RAVDIHSPSQ L--FRIPSLG GTTGTESPPQ PS-------- ---------- -------QAM LPAVPPDVLM PTASVEED-- ----------
 509502  LSAY------ ----HDDA-D EPECPEKFGK WRFIEELQTI --DEFREALW NEIQDYRKEV RGVNDLSGMP I--GRV-SLG SEEGSRRPSA PE-------- ---------- -------QTH VPE-EELGPV PEAVMTQI-- ----------
 964561  LAAY------ ----HEES-D EPDCPTKFER WRDIEKLQTL --DEFREALW KEIEDYRMEV RGMKTETTPF PSVAEKDRVQ TSPSLSPVQS ---------- ---------- ---------- ---------V RSPTTERD-- ----------
 261448  LASY------ ----HEES-D EPYCPTKFER WRDIEKLETL --EEFREALW KEIEDYRMDV RGLKPEAASL PRVVEESQIL LSPSRSPGKS ---------- ---------- ---------- ---------I KSPMTEAG-- ----------
1150169  LASY------ ----HDEA-D EPVCTTKFEK WKEIEKLETL --EEFREALW NEIEDYRMEV RGIRRVSGG- -----EKRVE ASPIQSPTPS ---------- ---------- ---------- ---------V KSPISRES-- ----------
 152363  LAAY------ ----HDES-D EPECSERFER WREIEKLETL --DDFRKALW DEIEDYRREV RGLLPVVDPL ELKERDADKE RDATKEEEFS ET-------- ------KAPE GDVQNGKDTP SADVSVSTVP SEPAVETP-- ----------
 379740  LAAY------ ----HDES-D EPECSERFER WREIEKLETL --DDFRKALW DEIEDYRREV RGLLPVVDPL ELKERDADKE RDATKEEEFS ET-------- ------KAPE GDVQNGKDTP SADVSVSTVP SEPAVETP-- ----------
 337664  LAAY------ ----HDES-D EPECSERFER WREIEKLETL --DDFRKALW DEIEDYRREV RGLLPVVDPL ELKERDADKE RDATKEEEFS ET-------- ------KAPE GDVQNGKDTP SADVSVSTVP SEPAVETP-- ----------
 434295  LAAY------ ----HDES-D EPECSERFER WREIEKLETL --DDFRKALW DEIEDYRREV RGLLPVVDPL ELKERDADKE RDATKEEEFS ET-------- ------KAPE GDVQNGKDTP SADVSVSTVP SEPAVETP-- ----------
 517048  LASY------ ----HDVT-D EPDCPETFER WRTLEALETL --EQYKEMLY NEIQDYRREV RGIPMEEPE- PEQEPALEPE P-ELESPRQL SA-------- -----RQSPI REMQRK-LEE IREMAENLQP PGADDTVF-- ----------
 731201  LASY------ ----HDVT-D EPDCPETFER WRTLEALETL --EQYKEMLY NEIQDYRREV RGIPMEEPE- PEQEPALEPE P-ELESPRQL SA-------- -----RQSPI REMQRK-LEE IREMAENLQP PGADDTVF-- ----------
 768698  LASY------ ----HDVT-D EPDCPETFER WRTLEALETL --EQYKEMLY NEIQDYRREV RGIPMEEPE- PEQEPALEPE P-ELESPRQL SA-------- -----RQSPI REMQRK-LEE IREMAENLQP PGADDTVF-- ----------
 517043  LASY------ ----HDVT-D EPDCPETFER WRTLEALETL --EQYKEMLY NEIQDYRREV RGIPMEEPE- PEQEPALEPE P-ELESPRQL SA-------- -----RQSPI REMQRK-LEE IREMAENLQP PGADDTVF-- ----------
 740533  LASY------ ----HDVT-D EPDCPETFER WRTLEALETL --EQYKEMLY NEIQDYRREV RGIPMEEPE- PEQEPALEPE P-ELESPRQL SA-------- -----RQSPI REMQRK-LEE IREMAENLQP PGADDTVF-- ----------
 178741  LASY------ ----HDVN-D EPECFEVFGR WREIEKLETL --PQFREAIY NEIDDYRKEV RSIYYDASES LDRSPSLRRQ LSEVTSPPAS EE-------- -----LQSPF ELKPAAEPGE AQWVQSPETA LQIRDVDP-- ----------
  54225  LASY------ ----HDVN-D EPECFEVFGR WREIEKLETL --PQFREAIY NEIDDYRKEV RSIYYDASES LDRSPSLRRQ LSEVTSPPAS EE-------- -----LQSPF ELKPAAEPGE AQWVQSPETA LQIRDVDP-- ----------
 163441  LASY------ ----HDVN-D EPECPEVFER WRDIEKLETL --PQFREAIF NEIEDYRNEV RSVFYDAPES IDRSPSLRRH PRAATSPPVS EE-------- -----LQSPF EYKAA---GE AARMQSPEST LDTREL---- ----------
 327966  LAAY------ ----HDVS-D EPSCPEVFER WRDIEKLETL --PEFKEAIW NEIQDYRREV RSIYYDAQES PDGSPIPDHQ PPETSLAPAA EE-------- -----LMSPF ELKPSA---E VPRVQSPEST EPLDVV---- ----------
 722194  LASY------ ----HDVN-D EPECPEPFKR WHTLEALETL --EQYKETLW DEIQDYRREV RSV---AATE GEHSASGRGS PTS-EIRRGL EE-------- -----IREMA ENMPR--PSD SVFLASPDMG AQVPE----- ----------
 128404  LSSY------ ----HDID-D EPVCAVKFNR WREVEELETI --EQFRDAIW REIQDYRREV RTFSFEIPED YERTRSRDRK ESSL------ ---------- ---------- ---------- ---------P GGPSTTQF-- ----------
 167758  LATY------ ----HDVE-D EPVCGKDFNR WREIEELETL --DDFRDALV NEVLECRKEV RNMAALDPEP EPPMVNVTEE PDSTEDHDEH DT-------- ---------- ---------- -----EEPAP LGGHKASR-- ----------
  26908  LATY------ ----HDVE-D EPVCGKDFNR WREIEELETL --DDFRDALV NEVLECRKEV RNMAALDPEP EPPMVNVTEE PDSTEDHDEH DT-------- ---------- ---------- -----EEPAP LGGHKASR-- ----------
 987811  LANY------ ----HDID-D EPVCPVPFEK WRAIEKLETI --EDFQKALW TEIEDYRREV RGIPVEIDDV ARPKSAGEVR AIEQKEA--- ---------- ---------- ---------- --------LV AMGNADGD-- ----------
 913595  LANY------ ----HDID-D EPVCPVPFEK WRAIEKLETI --EDFQKALW TEIEDYRREV RGIPVEIDDV ARPKSAGEVR AIEQKEA--- ---------- ---------- ---------- --------LV AMGNADGD-- ----------
 922124  LANY------ ----HDID-D EPVCPVPFEK WRAIEKLETI --EDFQKALW TEIEDYRREV RGIPVEIDDV ARPKSAGEVR AIEQKEA--- ---------- ---------- ---------- --------LV AMGNADGD-- ----------
 319286  LAPY------ ----HDET-D EPDCPSVFDK WEEVEGTHSL --DDFKRIIR KEVEEFRKEV RTVEEWEVQP ESENSKVGGA EQVEDGDEGE SE-------- -----QSEEW EHPPGLAAGS AVSTPDRPQD ASLQVTPR-- ----------
 332677  VATY------ ----HDES-D EPSCHAIFDK WEQVESLETI --DELRTAIS REIEEFRAEV RS-ETIEEED EEAEWVYEYA IEPEGGDGGV TG-------- -----SPAML E-PGERAHRL HYSGVDGPSP RSGSVTLP-- ----------
  75030  LATY------ ----HDMA-D EPDAPHSFHK WHEIERLETL --DQYRQAIW DEIHDYRREV RSMGAAAAAA ARQRDPDEIP SSRPSESP-- ---------- ---------- ---------- ---------P PVPDSPPP-- ----------
 740342  LATY------ ----HDMA-D EPDAPHSFHK WHEIERLETL --DQYRQAIW DEIHDYRREV RSMGAAAAAA ARQRDPDEIP SSRPSESP-- ---------- ---------- ---------- ---------P PVPDSPPP-- ----------
 769030  LATY------ ----HDMA-D EPDAPHSFHK WHEIERLETL --DQYRQAIW DEIHDYRREV RSMGAAAAAA ARQRDPDEIP SSRPSESP-- ---------- ---------- ---------- ---------P PVPDSPPP-- ----------
 493654  LETY------ ----HDEA-D EPTCPHVFEK WERIETLETL --DEFRAEIY KEVEEFRKEV RMVFEEPVVE EPSVIVNVES PEEVQKA--- ---------- ---------- ---------- ---------L PTVDGLPE-- ----------
  12113  LETY------ ----HDEA-D EPTCPHVFEK WQRIETLETL --DEFRAEIY KEVEEFRKEV RMVYEEPAVE EPSVIVNVES PEEVQKG--- ---------- ---------- ---------- ---------L PTVDGLPE-- ----------
 115011  LETY------ ----HDEA-D EPTCPHIFNK WERIETLESM --DDFRQEIY KEVEEFRKEV RMVYDEPAPV E-LLVAAPES PEHEQEE--- ---------- ---------- ---------- ---------L PAVDGLPE-- ----------
 192400  LETY------ ----HDEA-D EPTCPHVFTK WERIETLETL --DEFRAEIY KEVEEFRKEV RMVFEEPVVE EPSVIVNVES PEEVQKA--- ---------- ---------- ---------- ---------L PTVDGLPE-- ----------
 543665  LETY------ ----HDEA-D EPTCPHVFEK WQRIETLETL --DEFRAEIY KEVEEFRKE- RELFHPRATP DHLGLGRVTP PSAAQAA--- ---------- ---------- ---------- ---------L DNLPRHV--- ----------
  91520  LASY------ ----HDEG-D EPDCHAVFDK WRQIEQLETL --QEFREALW NEIEDYRAEV RGLKVEAVET TEVDDTVTPK PSILPSTEDE Q--------- ---------- ---------- ------PEKM KVGSSPEQ-- ----------
 127212  LASY------ ----HDEG-D EPDCHAVFDK WRQIEQLETL --QEFREALW NEIEDYRAEV RGLKVEAVET TEVDDTVTPK PSILPSTEDE Q--------- ---------- ---------- ------PEKM KVGSSPEQ-- ----------
 438879  LASY------ ----HDVS-D EPECPRIFDR WQFIEELQTM --DEYRDALW NEIEDYRREV RGVN-DLSGT PIRRFSEGSG SRPRFLKPNT KE-------- ---------- ---------- --------PE PEVVVDSPVD DKARMSAV-- ----------
 174304  LASY------ ----HDVS-D EPECPRIFDR WQFIEELQTM --DEYRDALW NEIEDYRREV RGVN-DLSGT PIRRFSEGSG SRPRFLKPNT KE-------- ---------- ---------- --------PE PEVVVDSPVD DKARMSAV-- ----------
 174289  LASY------ ----HDVS-D EPECPRIFDR WQFIEELQTM --DEYRDALW NEIEDYRREV RGVN-DLSGT PIRRFSEGSG SRPRFLKPNT KE-------- ---------- ---------- --------PE PEVVVDSPVD DKARMSAV-- ----------
 244084  LASY------ ----HDEA-D EPVCTTKFEK WKEIEKLETL --EEFREALW NEIEDYRMEV RGIR-RVSG- ---------- GEKRVEASPI QS-------- ---------- ---------- --------PT PSV--KSPIS RESNITGI-- ----------
 123741  LASY------ ----HDID-D EPDCPVKFEK WRWIEELETI --DEFREALW DEIQDYRREV RSMGYDLSGQ PIR------- -------STS RG-------- ---------- ---------- --------AS SSLGRQQQAE PVAPFSTS-- ----------
 172164  LASY------ ----HDID-D EPDCPVKFEK WRWIEELETI --DEFREALW DEIQDYRREV RSMGYDLSGQ PIR------- -------STS RG-------- ---------- ---------- --------AS SSLGRQQQAE PVAPFSTS-- ----------
  40528  LSSY------ ----HDID-D EPVCAVKFNR WREVEELETI --EQFRDAIW REIQDYRREV R
  39781  LSSY------ ----HDID-D EPVCAVKFNR WREVEELETI --EQFRDAIW REIQDYRREV RT
1155690  LGTY------ ----HDVA-D EPEAPKSFQK WHDIEKLETL --DQYRQAIW DEVQDYRREV R
 247448  VATY------ ----HDES-D EPSCHAIFDK WEQVESLETI --DELRTAIS REIEEFRAEV RSETIEEEDE EAE
 269975  LKAY------ ----HRSN-T NWIPPYPFDR FEEVEMLKTI --PDFQMGLE READEMQAEL
 246067  LKAY------ ----HRSN-T NWIPPYPFDR FEEVEMLKTI --PDFQMGLE REA
  44396  LKAY------ ----HRSN-T NWIPPYPFDR FEEVEMLKTI --PDFQMGLE READEMQAEL EALEAEEREA GFHQSSEGEG ESQNDSPEGT GG-------- ---------- ---------- ---------- --DVKDIKSS SSRNARGS-- ----------
 111248  LEPY------ ----HDPE-D EPTAP-PIP- -EEFFDFDKH --KDNLSKEQ LKQLIYQEIM RTNVNTPRPT LKKAVKEIRK SEQARTRAEG SLADNYSDGS PSNNPYLRTG TRPKSGNSGD SRQIKQEFAK TQANEP---- ----------
  54538  LEAY------ ----HDPE-D EPVAP-PLD- -PEFFEFDLH --KDDISREQ LKELLYEEII HSG------- --SFFVDLRK AAGGHYVDIC KSACTGYAGL WGAQPFI-DG VWVQLSSPRI CNYMPREFVT LQLGQC---- ----------
1341438  LEPY------ ----HDPA-D EPTAP-PLD- -PSFFDFDNG --KEQ-KKEH LKVLIYQEVM RPAAGAAHC- --SEYLDSML AAATPEEVRA DLATLAECAA IGRWLGGESH YERQAANESE RRALLR-FAG LAESEL---- ----------
1267921  LEPY------ ----HDPA-D EPTAP-PLD- -PSFFDFDNG --KEQ-KKEH LKVLIYQEVM RPAAGAAHC- --SEYLDSML AAATPEEVRA DLATLAECAA IGRWLGGESH YERQAANESE RRALLR-FAG LAESEL---- ----------
 692594  LAPY------ ----HDPT-D EPVAEEKFD- -WSFNDADLP --VDTWKIKI GP--EVETWL GVEEGSDPAI --ENEWPLLP FMALSDGAHT LENPLLYALS LMYPNARKEE NVLTQAGASS TEDF------ ---------- ----------
 384872  LHIW------ ----HDAS-D EPSCPTTFD- -FQFEVVEEI --NDMKKMIL AEVNRFRQMV RVQPGAGAG- --PAQAPQVP IPNNYDRAGY EDPRPQEAFN QGGWQGSDLE RDLQGLDGSP THPYISKTIH TMTAADIETN KRKRKHKSKK
 419622  LQIW------ ----HDAS-D EPSCPTTFD- -FQFEVVEEI --PDMKKMIL AEVHRFRQMV RVQPGAGAGG QGQQQNQQVP IPNNQDRGGY EDPRPQEAFN QGGWQGSDLE RDLQG----- ---------- ---------- ----------
 328081  LQVW------ ----HDPA-D EPLCEVPFD- -FSFEQEDST --NGMRNLII QEVRSFRQLV RRQAAPAPPR --AHELPPAP QPPQQPHAGV GPAFGDEDVN TGA-DALEHP GNSSGAGMHR EGGIGERKVG NRAEAVWRGV VGRKWH----
  61803  LASL------ ----HQPD-D EPEHHEFFQF D-ENVANSDL RKDDIQRQIL QEVFSFHPEE Q-KVAEATRR KPPLRRLLTR QNVFSRQ
  40664  LASL------ ----HQPD-D EPEHHEFFQF D-ENVANSDL RKDDIQRQIL QEVFSFHPEE Q-KVAEATRR KPPLRRLLTR QNVFSRQ
 105104  LASL------ ----SNEA-D EPICEKRFEF E-YE--KETL SKEMLQELMW QEMAKYHPHS M----EELRQ KQREGRLATP RLIIPV
 147180  LKTY------ ----HR---- QPIRSAPRPF DLESIEQMDL TRKQLRQAMS QEIAHYRGLG A-YSSGSNFP PQQGQPQVQP QQQSQLPQST MR-------- ---------S ETSANSATPP IPEQHSVRP
 148245  LADL------ ----HDEN-E VPVASSMFDL SFEHGYRAEM PKDVMQRLIF DDMITFHPEE TFYTAQNSAR SDISSSSRSS SSSLPVPTGP AH-------- ---------Q YHQSFPRVSS KGRQRTAGAA AHAHSHK
 146852  MASL------ ----ANPD-D EPNCSEMFNF EHETV--EDF SKPMLQELIW KEMVHYHPEA QAALDLRKSQ GILAIDALET KM
  33818  MASL------ ----ANPD-D EPNCSEMFNF EHETV--EDF SKPMLQELIW KEMVHYHPEA QAALDLRKSQ GILAIDALET KM
 116469  MASL------ ----ANPD-D EPNCSEMFNF EHETV--EDF SKPMLQELIW KEMRAGLDHM HLDQDIRDGN
 503885  LAEY------ ----HAEE-D EPNCPSQISI PFDSV----L QTKTHFSSEE QDEIRKHITE AVNTFKMAQM KLQAQKLQST KSPASA
 503914  LAEY------ ----HAEE-D EPNCPSQISI PFDSV----L QTKTHFSSEE QDEIRKHITE AVNTFKMAQM KLQAQKLQST KSPASA
6817382  LSAY------ ----HDPL-D EPDCPEKFEN AGYYSFN
 145980  LAIW------ ----HDAS-D EPASKGRTKS R--KRQ---Q LATTEPRY-- ---------- ---------- ---------- ------S--- ---------- ---------- ---------- P
 328170  LHIW------ ----HDAS-D EPSCPTTFDF H--FEV---W L--FSTRI-- ---------- ---------- ---------- ------F--- ---------- ---------- ---------- PPL
 691008  LHIW------ ----HDAS-D EPNCPTTPGY Q--ALL---T MKWLPTVI-- ---------- ---------- ---------- ------Y--- ---------- ---------- ---------- DELCRTGERK THQCTI
  73316  LAIW------ ----HDPS-D EPACPTKFDF R--FEN---S ERPLQTNT-- ---------- ---------- ---------- ------I--- ---------- ---------- ---------- MKGSN
1219281  LQIW------ ----HDAQ-D EPNCPTPFDF S--FEVVD-E ISDMRKMI-- ---------- ---------- ---------- ------L--- ---------- ---------- ---------- NEVQNFRNAV RNKPVHVI-- ----------
 422525  LSAY------ ----HDPE-D EPVHNRSFDF S--FESVN-D MLQMKGSH-- ---------- ---------- ---------- ------P--- ---------- ---------- ---------- PSFHLMEDWK LIKIPGTR-- ----------
 212435  LSAY------ ----HDED-D ERLSNQLW-L S--NKNIY-T LCSSAVLL-- ---------- ---------- ---------- ------T--- ---------- ---------- ---------- GPILMPRSKI ITK
 187307  FQVW------ ----HDPS-D EPICDSEEE- ----EEEE-G EEQLRILL-- ---------- ---------- ---------- ------L--- ---------- ---------- ---------- QRCSRLLHCC SRGTR----- ----------
 146240  LAVW------ ----HDPA-D EPVCASPFDF G--FEEED-S IEQMKRLI-- ---------- ---------- ---------- ------V--- ---------- ---------- ---------- EEVHAFRAER MPPL
 156221  FQVW------ ----CDPA-D EPVCHSTFDF G--FENED-S MDGMKRLI-- ---------- ---------- ---------- ------V--- ---------- ---------- ---------- DEVNSFRAEI RSQV------ ----------
  48865  LQVW------ ----HDPA-D EPICETKFDF N--FEEED-N IEGMKKLI-- ---------- ---------- ---------- ------I--- ---------- ---------- ---------- EEVNSFRAES PHPIA----- ----------
 226510  LAVW------ ----HDPA-D EPVCPTVSAC D--IRFT
 821431  LQVW------ ----HDPA-D EPVCESHVEF A--GKERR-Q RVDLL
  12784  LAVW------ ----HDPA-D EPVCPATFDF G--FEAEE-S SEGMKAMI-- ---------- ---------- ---------- ------I--- ---------- ---------- ---------- TEVAAFRQQV RNQIRAAP-- ----------
 157692  CSSA------ ----RRSS-T YSRPVPTGSI R--GPSAT-G DNFIREWS-- ---------- ---------- ---------- ------R--- ---------- ---------- ---------- LRERAARRIG RHAMNNAA-- ----------
  47352  LASL------ ----SNEA-D EPICEKRFEF E--YEKETLS KEMLQELM-- ---------- ---------- ---------- ------W--- ---------- ---------- ---------- QEMAKYHPHS MEELR----- ----------
 136580  LEPY------ ----HDPE-D EPTAEPIPEE F--F-DFD-- --KNKDNL-- ---------- ---------- ---------- ------S--- ---------- ---------- ---------- KEDLRRKLKP SHNADANF-- ----------
 422908  LEPY------ ----HDPE-D EPTADPIPEA F--F-DFD-- --KNKDTL-- ---------- ---------- ---------- ------T--- ---------- ---------- ---------- KEQLKGRISL PVLRMPTD-- ----------
  71188  LEPY------ ----HDPD-D EPTAEPIPEE F--F-SFD-- --LQKDTL-- ---------- ---------- ---------- ------S--- ---------- ---------- ---------- KEQLR-QLIY EEIMR
2049832  LEPY------ ----HDPD-D EPTAPPIPEE F--F-DFD-- --KHKDSL-- ---------- ---------- ---------- ------S--- ---------- ---------- ---------- KEHGQ-GSVS QNCLRKRF-- ----------
2023270  LEPY------ ----HDPD-D EPTAPPIPEE F--F-DFD-- --KHKDSL-- ---------- ---------- ---------- ------S--- ---------- ---------- ---------- KEHGQ-GSVS QNCLRKRF-- ----------
  62157  LEPY------ ----HDPE-D EPTADAIPEE F--F-DFD-- --KNKDNL-- ---------- ---------- ---------- ------T--- ---------- ---------- ---------- KEQLK-QLIF EEIMR----- ----------
 117327  LEPY------ ----HDPE-D EPTADPIPEE F--F-DFD-- --KNKDNL-- ---------- ---------- ---------- ------T--- ---------- ---------- ---------- KEQLK-RLIY EEIMR----- ----------
1420954  LEPY------ ----HDPD-D EPTAPPIPEE F--F-DFD-- --KNKDNL-- ---------- ---------- ---------- ------S--- ---------- ---------- ---------- KEQLK-QLIY QEIMR----- ----------
 146600  LEPY------ ----HDPE-D EPTAAPIPEE F--F-DFD-- --KNKDNL-- ---------- ---------- ---------- ------T--- ---------- ---------- ---------- KEQLK-QLIY NEIMR
 427542  LEPY------ ----HDPD-D EPTASPIPEE F--F-DFD-- --KNKDNL-- ---------- ---------- ---------- ------S--- ---------- ---------- ---------- KEQLK-ELIY QEIMR
 524886  LEPY------ ----HDPE-D EPTAPPIPEE F--F-DFD-- --KHKDNL-- ---------- ---------- ---------- ------S--- ---------- ---------- ---------- KEQLK-QLIY QEIMRGGG-- ----------
 304061  LEPY------ ----HDPE-D EPTADPIPEE F--F-DFD-- --KNKDNL-- ---------- ---------- ---------- ------T--- ---------- ---------- ---------- KEQLK-MLIY QEIMRRID-- ----------
 262011  LEPY------ ----HDPD-D EPTAPPIPEE F--F-DFD-- --KNKDNL-- ---------- ---------- ---------- ------S--- ---------- ---------- ---------- KEQLK-QLIY QEIMR
 385460  LEPY------ ----HDPE-D EPTAPPIPEE F--F-DFD-- --KHKDNL-- ---------- ---------- ---------- ------S--- ---------- ---------- ---------- KEQLK-QLIY QEIMR
 322662  LEPY------ ----HDPE-D EPTAPPIPEE F--F-DFD-- --KHKDNL-- ---------- ---------- ---------- ------S--- ---------- ---------- ---------- KEQLK-QLIY QEIMR
1531698  LEPY------ ----HDPD-D EPTAPPIPEE F--F-DFD-- --KHKDNL-- ---------- ---------- ---------- ------S--- ---------- ---------- ---------- KEQLK-QLIY QEIMRKTR-- ----------
1273298  LEPY------ ----HDPD-D EPTAPPIPEE F--F-DFD-- --KHKDNL-- ---------- ---------- ---------- ------S--- ---------- ---------- ---------- KEQLK-QLIY QEIMR
 211888  LEPY------ ----HDPD-D EPTAPPIPEG F--F-DFD-- --KNKDAL-- ---------- ---------- ---------- ------S--- ---------- ---------- ---------- KEQLK-LLIY EEIMRHGT-- ----------
1140823  LEPY------ ----HDPD-D EPTAPPIPEG F--F-DFD-- --KNKDAL-- ---------- ---------- ---------- ------S--- ---------- ---------- ---------- KEQLK-LLIY EEIMR
  84032  LEPY------ ----HDPE-D EPTAEPIPEE F--F-DFD-- --KAKDSL-- ---------- ---------- ---------- ------S--- ---------- ---------- ---------- KEQLK-VLIY EEIMR
 337569  LEPY------ ----HDPD-D EPTAEPIPEE F--F-DFD-- --KAKDSL-- ---------- ---------- ---------- ------S--- ---------- ---------- ---------- KEQLK-VLIY EEIMR
 675946  LEPY------ ----HDPD-D EPTADPIPEE F--F-DFD-- --KNKDNL-- ---------- ---------- ---------- ------T--- ---------- ---------- ---------- KEQLK-LLIY QEIMR
 188798  LEPY------ ----HDPD-D EPGADPIPEE F--F-DFD-- --KNKDNL-- ---------- ---------- ---------- ------T--- ---------- ---------- ---------- KEQLK-LLIY QEIMR
 364357  LEPY------ ----HDPD-D EPTADPIPEE F--F-DFD-- --KNKDNL-- ---------- ---------- ---------- ------T--- ---------- ---------- ---------- KEQLK-LLIY QEIMR
 417259  LEPY------ ----HDPD-D EPTADPIPEE F--F-DFD-- --KNKDNL-- ---------- ---------- ---------- ------T--- ---------- ---------- ---------- KEQLK-LLIY QEIMR
 391850  LEPY------ ----HDPD-D EPTADPIPEE F--F-DFD-- --KNKDNL-- ---------- ---------- ---------- ------T--- ---------- ---------- ---------- KEQLK-R-DY AVIASPTI-- ----------
 420539  LEPY------ ----HDPE-D EPTADPIPEE F--F-DFD-- --KKKDNL-- ---------- ---------- ---------- ------S--- ---------- ---------- ---------- KEQLK-SEFR VDCPDISV-- ----------
1649896  LEPY------ ----HDPE-D EPTAPTIPDE F--F-DFD-- --RRKDEL-- ---------- ---------- ---------- ------S--- ---------- ---------- ---------- KEQLK-MLIY QEIMR
 280694  LEPY------ ----HDPE-D EPTAPPIPDA F--F-DFD-- --RRKDEL-- ---------- ---------- ---------- ------S--- ---------- ---------- ---------- KEELKSKSHN ALNCE
 381396  LEPY------ ----HDPD-D EPTAPPIPDG F--F-DFD-- --KNKDAL-- ---------- ---------- ---------- ------S--- ---------- ---------- ---------- KEQLKCMFHL HPNEHYLQ-- ----------
 685453  LEPY------ ----HDPE-D EPTAPPIPEE F--F-DFD-- --KHKDNL-- ---------- ---------- ---------- ------S--- ---------- ---------- ---------- KEQLK-QLIY QEIMR
 284413  LEPY------ ----HDPD-D EPDAPPIPES F--F-DFD-- --RYKDQL-- ---------- ---------- ---------- ------T--- ---------- ---------- ---------- KEQLK-QMLY EEIAH
 384436  LEPY------ ----HDPD-D EPTAPPIPEG F--F-DFD-- --KNKDAL-- ---------- ---------- ---------- ------S--- ---------- ---------- ---------- KEQLK-LLIY EEIMR
 907966  LEPY------ ----HDPE-D EPTAPTIPDE F--F-DFD-- --RRKDEL-- ---------- ---------- ---------- ------S--- ---------- ---------- ---------- KEQLK-MLIY QEIMR
 205730  LEPY------ ----HDPE-D EPIAPPIPES F--F-EFD-- --RYKDTL-- ---------- ---------- ---------- ------T--- ---------- ---------- ---------- KDQLKVLLYN EIMTPPSF-- ----------
  86646  LEQY------ ----HDPE-D EPIAPPVPES F--F-DFD-- --RYKEQL-- ---------- ---------- ---------- ------S--- ---------- ---------- ---------- KEQLKVMIYD EITFNGGQ-- ----------
   9721  LEQY------ ----HDPE-D EPMAQPMPES F--F-DFD-- --RYKEQL-- ---------- ---------- ---------- ------S--- ---------- ---------- ---------- KEQLKVMIYE EITNNGVT-- ----------
   2612  LELY------ ----HDPN-D EPNAPPIPPG F--F-DFD-- --SHKDSL-- ---------- ---------- ---------- ------T--- ---------- ---------- ---------- KLQLK-ELIY AEACDIPR-- ----------
 102501  VKLY------ ----HDPI-D EPIANPIPEN F--F-DFD-- --KNKDKL-- ---------- ---------- ---------- ------T--- ---------- ---------- ---------- LADLK-QLLY NEIMEPLP-- ----------
  98652  LKLY------ ----HDPN-D EPVANPIPRN F--F-DFD-- --KYKDKL-- ---------- ---------- ---------- ------S--- ---------- ---------- ---------- IEQLK-KLLY GEIMAPLP-- ----------
  78159  LALY------ ----HDAE-D EPDAEPIPND F--F-DFD-- --KHKDDL-- ---------- ---------- ---------- ------S--- ---------- ---------- ---------- VEELK-RMLF EEVMKPLG-- ----------
 240194  LKLY------ ----HDPD-D EPISDKIPED F--F-DFD-- --KRKDEL-- ---------- ---------- ---------- ------S--- ---------- ---------- ---------- IEELK-KLLY DEIMKPL
 193495  LQLY------ ----HDPD-D EPNAAPLDEK F--WGDFN-- --ENPEQL-- ---------- ---------- ---------- ------D--- ---------- ---------- ---------- MEELK-TLLF EEVMKPLR-- ----------
 171744  LQLY------ ----HDPD-D EPNAAPLDEK F--WGDFN-- --ENPEQL-- ---------- ---------- ---------- ------D--- ---------- ---------- ---------- MEELK-TLLF EEVMKPLR-- ----------
   4564  LSFY------ ----HEPD-D EPDAEKIPDD F--F-DFD-- --RRKDEL-- ---------- ---------- ---------- ------T--- ---------- ---------- ---------- MPELK-KMLY EEIMKPLR
   6035  VSFY------ ----HEPN-D EPVAEKIPDD F--F-DFD-- --RRKDEL-- ---------- ---------- ---------- ------S--- ---------- ---------- ---------- LLELK-KMLY DEIMEPLN-- ----------
 256278  LEPY------ ----HDPD-D EPTAPPIPDG F--F-DFD-- --KNKDAL-- ---------- ---------- ---------- ------S--- ---------- ---------- ---------- KEQLK-YLIY EEIMR
```

```
 120199 LEAY------ ----HDPD-D EPVAPPLDPE F--F-DFD-- --LHKDDI-- ---------- ---------- ---------- ------S--- ---------- ---------- ---------- REQLK-ELLY EEIMSFQP-- ----------
1003994 LESY------ ----HDPD-D EPVAPPLDPS F--F-EFD-- --LHKDDI-- ---------- ---------- ---------- ------S--- ---------- ---------- ---------- REQLK-ELLY DEIMSFKA-- ----------
 145010 LEAY------ ----HDPE-D EPVAPPLDPE F--F-EFD-- --LHKDDI-- ---------- ---------- ---------- ------S--- ---------- ---------- ---------- REQLK-ELLY EEIMSFRP-- ----------
 183924 LEAY------ ----HDPE-D EPVAPPLDPE F--F-EFD-- --LHKDDI-- ---------- ---------- ---------- ------S--- ---------- ---------- ---------- REQLK-ELLY EEIMSFRP-- ----------
 725911 LEAY------ ----HDPE-D EPVAPPLDPE F--F-EFD-- --LHKDDI-- ---------- ---------- ---------- ------S--- ---------- ---------- ---------- REQLK-ELLY EEIMSFRA-- ----------
1166877 LEAY------ ----HDPE-D EPVAPPLDPE F--F-EFD-- --LHKDDI-- ---------- ---------- ---------- ------S--- ---------- ---------- ---------- REQLK-ELLY EEIMSFRA-- ----------
 574353 LEAY------ ----HDPD-D EPCAPPLDPE F--F-EFD-- --LHKDDI-- ---------- ---------- ---------- ------S--- ---------- ---------- ---------- REQLK-ELLY EEIMSFRP-- ----------
 277341 LEAY------ ----HDPE-D EPVAPPLDPE F--F-EFD-- --LHKDDI-- ---------- ---------- ---------- ------S--- ---------- ---------- ---------- REQLK-ELLY EEIMSFQP-- ----------
 965410 LEAY------ ----HDPD-D EPVAPPLDPE F--F-EFD-- --LHKDDI-- ---------- ---------- ---------- ------S--- ---------- ---------- ---------- REQLK-ELLY DEIMSFRP-- ----------
1040134 LEAY------ ----HDPD-D EPVAPPLDPE F--F-EFD-- --LHKDDI-- ---------- ---------- ---------- ------S--- ---------- ---------- ---------- REQLK-ELLY EEIMSFRP-- ----------
 801239 LEAY------ ----HDPD-D EPIAPPLDPE F--F-EFD-- --LHKDDI-- ---------- ---------- ---------- ------S--- ---------- ---------- ---------- REQLK-ELLY EEIMSFRP-- ----------
 457049 LEAY------ ----HDPD-D EPVAPPLDPE F--F-EFD-- --LHKDDI-- ---------- ---------- ---------- ------S--- ---------- ---------- ---------- REQLK-ELLY EEIMSFKP-- ----------
 777636 LEAY------ ----HDPD-D EPVAPPLDPE F--F-EFD-- --LHKDDI-- ---------- ---------- ---------- ------S--- ---------- ---------- ---------- REQLK-ELLY EEIMTFRP-- ----------
 167531 LEAY------ ----HDPD-D EPVAPPLDPE F--F-EFD-- --LHKDDI-- ---------- ---------- ---------- ------S--- ---------- ---------- ---------- REQLK-ELLY EEIMTFRP-- ----------
 179443 LEAY------ ----HDPE-D EPCAPPLDPE F--F-EFD-- --LHKDDI-- ---------- ---------- ---------- ------S--- ---------- ---------- ---------- REQLK-ELLY EEIMSFRP-- ----------
 388203 LEAY------ ----HDPD-D EPVAPPLDPE F--F-EFD-- --LHKDDI-- ---------- ---------- ---------- ------S--- ---------- ---------- ---------- REQLK-ELLY EEIMSFNP-- ----------
 761034 LEAY------ ----HDPE-D EPVAPPLDPE F--F-EFD-- --LHKDDI-- ---------- ---------- ---------- ------S--- ---------- ---------- ---------- REQLK-ELLY EEIMSFHP-- ----------
 117742 LEAYASTFNP FMRCHDPD-D EPVAPPLDPD F--F-EFD-- --LHKDDI-- ---------- ---------- ---------- ------S--- ---------- ---------- ---------- RDQLK-ELLY EEIMSFTP-- ----------
 506090 LEAY------ ----HDPD-D EPVAPPLEPE F--F-EFD-- --LHKDDI-- ---------- ---------- ---------- ------S--- ---------- ---------- ---------- REELK-KMLY DEIISFNP-- ----------
  21911 LEAY------ ----HDPD-D EPVAPPLEPE F--F-EFD-- --LHKDDI-- ---------- ---------- ---------- ------S--- ---------- ---------- ---------- REELK-KMLY DEILSFNP-- ----------
7342346 LEAY------ ----HDPD-D EPVAPPLEPE F--F-EFD-- --LHKDDI-- ---------- ---------- ---------- ------S--- ---------- ---------- ---------- REQLK-ELLY EEIMTFQP-- ----------
 435735 LEAY------ ----HDPD-D EPVAPPLDPE F--F-EFD-- --LHKDDI-- ---------- ---------- ---------- ------S--- ---------- ---------- ---------- REQLK-ELLY EEIMSFRP-- ----------
 267232 LEAY------ ----HDPE-D EPVAPPLDPE F--F-EFD-- --LHKDDI-- ---------- ---------- ---------- ------S--- ---------- ---------- ---------- REQLK-ELLY EEIMSFQP-- ----------
 679497 LESY------ ----HDPE-D EPVAPPLDPE F--F-EFD-- --LHKDDI-- ---------- ---------- ---------- ------S--- ---------- ---------- ---------- REQLK-ELLY DEIMSFQP-- ----------
 457636 LESY------ ----HDPE-D EPVAPPLDPE F--F-EFD-- --LHKDDI-- ---------- ---------- ---------- ------S--- ---------- ---------- ---------- REQLK-ELLY DEIMSFQP-- ----------
1340042 LEAY------ ----HDES-D EPVAPPLEPE F--F-DFD-- --LQKDNI-- ---------- ---------- ---------- ------S--- ---------- ---------- ---------- REDLKSGSIH AHPKLSLI-- ----------
 225932 LEAY------ ----HDPE-D EPVAPPLDED F--F-AFD-- --RQKDDI-- ---------- ---------- ---------- ------S--- ---------- ---------- ---------- REELKRLLFE EIESEFQA-- ----------
 346924 LDAY------ ----HDPE-D EPVAKPLDPD F--F-DFD-- --LQKNAI-- ---------- ---------- ---------- ------G--- ---------- ---------- ---------- REELK-QLLY DEIHSFHA-- ----------
 760343 LEAY------ ----HDES-D EPVAPPLEPE F--F-DFD-- --LQKDNI-- ---------- ---------- ---------- ------S--- ---------- ---------- ---------- REDLK-KLLY NEIMTFKP-- ----------
  15147 LDAY------ ----HDPE-D EPGATPLDPN F--F-EFD-- --LQKDNI-- ---------- ---------- ---------- ------T--- ---------- ---------- ---------- REELK-KLLY DEIVTFRP-- ----------
 108680 LASY------ ----HDPD-D EPDSAPLEPS F--F-HFD-- --NGKEQL-- ---------- ---------- ---------- ------T--- ---------- ---------- ---------- REQLK-QLIY NEIMT
 724148 LASY------ ----HDPD-D EPDSAPLEPS F--F-HFD-- --NGKEQL-- ---------- ---------- ---------- ------T--- ---------- ---------- ---------- REQLK-QLIY NEIMT
 276168 LEPY------ ----HDPE-D EPTASPLDPS F--F-SFD-- --YTKEQL-- ---------- ---------- ---------- ------S--- ---------- ---------- ---------- RSQLK-QLIY QEIMQ
 194959 VSAY------ ----HDPE-D EPAATPLEPS Y--F-DFD-- --MHKDDL-- ---------- ---------- ---------- ------S--- ---------- ---------- ---------- KDQLK-ELLY EEVLSFVP-- ----------
 345309 LAAY------ ----HDPE-D EPVVTPLSPD Y--F-EFD-- --MHKDDL-- ---------- ---------- ---------- ------S--- ---------- ---------- ---------- KDQLKVELLY QEVLSFTP-- ----------
7189608 LSAY------ ----HDPE-D EPVVTPLSPS Y--F-EFD-- --LHKDDL-- ---------- ---------- ---------- ------S--- ---------- ---------- ---------- KDQLK-ELLY EEVLSFNP-- ----------
 439783 LAAY------ ----HDPE-D EPVVTPLSPS Y--F-EFD-- --LHKDDL-- ---------- ---------- ---------- ------S--- ---------- ---------- ---------- KDQLK-ELLY EEVLSFVP-- ----------
  73775 LSAY------ ----HDPE-D EPTVTPLAPE Y--F-EFD-- --LHKDDL-- ---------- ---------- ---------- ------S--- ---------- ---------- ---------- KDQLK-ELLY AEVMSFVP-- ----------
 681486 LTAY------ ----HDPE-D EPTVQPLSPS Y--F-EFD-- --LHKDDL-- ---------- ---------- ---------- ------S--- ---------- ---------- ---------- KDQLK-ELLY EEVLSFIP-- ----------
 671673 LTAY------ ----HDPE-D EPTVQPLSPS Y--F-EFD-- --LHKDDL-- ---------- ---------- ---------- ------S--- ---------- ---------- ---------- KDQLK-ELLY EEVLSFVP-- ----------
 829815 LASY------ ----HDPE-D EPVVAPLSAS Y--F-EFD-- --LHKDQL-- ---------- ---------- ---------- ------T--- ---------- ---------- ---------- KDQLK-QLLY EEVISFAP-- ----------
 413627 LSAY------ ----HDPD-D EPVATSLDPD Y--F-DFD-- --DHKEAL-- ---------- ---------- ---------- ------S--- ---------- ---------- ---------- KTQLK-ELLY EEVVAFSP-- ----------
 109090 LAAY------ ----HDPE-D EPTCAILDPD Y--F-AFD-- --TYKDAL-- ---------- ---------- ---------- ------S--- ---------- ---------- ---------- KEELK-TLLY EEIISFNS-- ----------
  43366 LANY------ ----HDPE-D EPTTEELDPD Y--F-EFD-- --SRKEDM-- ---------- ---------- ---------- ------T--- ---------- ---------- ---------- KADLK-ELLY QEILSFVP-- ----------
 706326 LAAY------ ----HDPE-D EPAVSSLDSE Y--F-EFD-- ----YLDL-- ---------- ---------- ---------- ------S--- ---------- ---------- ---------- KDELK-ELLY AEVKSFVP-- ----------
1157202 LAAY------ ----HDPE-D EPAVSSLDSE Y--F-EFD-- ----YLDL-- ---------- ---------- ---------- ------N--- ---------- ---------- ---------- KDELK-GLLY AEVMSFVP-- ----------
 743174 LTAY------ ----HDPD-D EPAVSSLDAD Y--F-ECD-- ----YLDL-- ---------- ---------- ---------- ------S--- ---------- ---------- ---------- KQELK-ELLY EEVMSFTP-- ----------
   5331 LAAY------ ----HDPD-D EPAVSSLDSE Y--F-EFD-- ----YLDL-- ---------- ---------- ---------- ------N--- ---------- ---------- ---------- KDELK-DLLY AEVMSFVP-- ----------
 713967 LTAY------ ----HDPD-D EPAVSSLDAD Y--F-ECD-- ----YLDL-- ---------- ---------- ---------- ------S--- ---------- ---------- ---------- KQELK-GICL TRFRCGDL
 151043 LSAY------ ----HDPE-D EPAVPSLDPE Y--F-EFD-- ----YQEL-- ---------- ---------- ---------- ------N--- ---------- ---------- ---------- KDELK-KLLY DEVMSFQS-- ----------
 481570 LSAY------ ----HDPE-D EPAVPSLDPE Y--F-EFD-- ----YKEL-- ---------- ---------- ---------- ------N--- ---------- ---------- ---------- KDELK-QLLY DEVMSFQS-- ----------
 104738 LAAY------ ----HDPE-D EPAVSSLDPE Y--F-EFD-- ----YQEL-- ---------- ---------- ---------- ------N--- ---------- ---------- ---------- KEDLK-KLLF DEVMSFQS-- ----------
 689474 LAAY------ ----HDPQ-D EPVVASLDPD Y--F-DFDRE P-STLHKL-- ---------- ---------- ---------- ------P--- ---------- ---------- ---------- LGFPY-SRSL HRVQRRPH-- ----------
 179470 LASY------ ----HDPE-D EPVVASLDPD Y--F-DFD-- --QYKDDL-- ---------- ---------- ---------- ------T--- ---------- ---------- ---------- KAELK-ELLY EEIQSFVP-- ----------
  31001 LSAY------ ----HDPE-D EPIVASLDPD Y--F-DFDLK V-EYKDDL-- ---------- ---------- ---------- ------S--- ---------- ---------- ---------- KSELK-ELLY EEILSFVP-- ----------
 155293 VAAY------ ----YDLN-D EPECFPMDPE Y--F-HFD-- --TQKEML-- ---------- ---------- ---------- ------G--- ---------- ---------- ---------- KAQLK-ELLY EEIQSFTP-- ----------
  83606 VAAY------ ----YDLN-D EPECFPMDPE Y--F-HFD-- --SKFSSL-- ---------- ---------- ---------- ------A--- ---------- ---------- ---------- CRSQT-NGMA VI
  79026 LEP------- ---YHDPH-D EPDGAPLPPS F--F-DFD-- ---YVEPL-- ---------- ---------- ---------- ------G--- ---------- ---------- ---------- KEELK----- ---------- ----------
  79053 LEP------- ---YHDPH-D EPDGAPLPPS F--F-DFD-- ---YVEPL-- ---------- ---------- ---------- ------G--- ---------- ---------- ---------- KEELK----- ---------- ----------
 188294 LQP------- ---YHDPL-D EPTAEPLDPS F--F-DFD-- ---YGDPI-- ---------- ---------- ---------- ------N--- ---------- ---------- ---------- KEELK----- ---------- ----------
 422236 LAP------- ---YHDPG-D EPTAEPLDPS F--F-DFD-- ---NGEQM-- ---------- ---------- ---------- ------R--- ---------- ---------- ---------- KEELK----- ---------- ----------
  59295 LQS------- ---YHDAQ-D EPTSEPLDPS F--F-DFD-- ---YGTPL-- ---------- ---------- ---------- ------G--- ---------- ---------- ---------- KEELK----- ---------- ----------
  91727 LEA------- ---YHDPS-D EPTAEPLDPS F--F-DFD-- ---NGDPL-- ---------- ---------- ---------- ------G--- ---------- ---------- ---------- KEQLKGECY- ---------- ----------
  92869 LEA------- ---YHDPS-D EPTAEPLDPS F--F-DFD-- ---NGDPL-- ---------- ---------- ---------- ------G--- ---------- ---------- ---------- KEQLK----- ---------- ----------
 513929 LEA-NPDSVR VQAYHDPS-D EPTAEPLDPS F--F-DFD-- ---NGDPL-- ---------- ---------- ---------- ------G--- ---------- ---------- ---------- KEDLK----- ---------- ----------
 166192 LEA------- ---YHDPS-D EPTAEPLDPS F--F-DFD-- ---NGDPL-- ---------- ---------- ---------- ------G--- ---------- ---------- ---------- KEQLK----- ---------- ----------
 210022 LEP------- ---YHDPQ-D EPTAAPLDPT F--F-DFD-- ---NGDAL-- ---------- ---------- ---------- ------T--- ---------- ---------- ---------- KEELK----- ---------- ----------
 847083 LAP------- ---YHDPL-D EPKAPPINPS F--F-DFD-- ---NGGET-- ---------- ---------- ---------- ------S--- ---------- ---------- ---------- KEELK----- ---------- ----------
 819022 LAP------- ---YHDPQ-D EPTAEPIDPQ F--F-HFD-- ---NGDPM-- ---------- ---------- ---------- ------K--- ---------- ---------- ---------- KEDLK----- ---------- ----------
  50473 LAP------- ---YHDPQ-D EPTAEPIDPQ F--F-HFD-- ---NGDPM-- ---------- ---------- ---------- ------K--- ---------- ---------- ---------- KEDLK----- ---------- ----------
 850567 FDP------- ---YHDVD-D EPVADPIDPS F--F-DFD-- ---NGAPL-- ---------- ---------- ---------- ------S--- ---------- ---------- ---------- KEQLKGRNLH DPHLFYAS-- ----------
 183076 FEP------- ---YHDPD-D EPVADPIDPS F--F-DFD-- ---NGEPL-- ---------- ---------- ---------- ------E--- ---------- ---------- ---------- KERLK----- ---------- ----------
1114014 FEP------- ---YHDAE-D EPVADPIDPS F--F-DFD-- ---NGDPL-- ---------- ---------- ---------- ------E--- ---------- ---------- ---------- KEELK----- ---------- ----------
  47661 LEP------- ---YHDPQ-D EPTAEPLDPS F--F-DFD-- ---NGIPL-- ---------- ---------- ---------- ------E--- ---------- ---------- ---------- KEELKG---- ---------- ----------
6536531 LQP------- ---YHDAE-D EPTAEPLDPT F--F-DFD-- ---IGDPL-- ---------- ---------- ---------- ------S--- ---------- ---------- ---------- KEELKVLIYK EI--IKPD-- ----------
 277624 LLP------- ---YHDIE-D EPTAEPLDPS F--F-DFDM- ---VAEPL-- ---------- ---------- ---------- ------S--- ---------- ---------- ---------- KEQLKVLIYE EI--TSPD-- ----------
  77457 LSP------- ---YHDLD-D EPTAAPLDPS F--F-DFD-- ---IGEPL-- ---------- ---------- ---------- ------T--- ---------- ---------- ---------- KEELKGITFL RFFCVACL-- ----------
  89431 LQP------- ---YHDPE-D EPTAQPLDSA F--F-DFD-- ---FGDPL-- ---------- ---------- ---------- ------P--- ---------- ---------- ---------- KEQLKGLLPS SLL-APTN-- ----------
 484632 LQP------- ---YHDPE-D EPTAQPLDPA F--F-DFD-- ---FGDPL-- ---------- ---------- ---------- ------P--- ---------- ---------- ---------- KEQLK----- ---------- ----------
 161998 LEP------- ---YHDPM-D EPTAPPLDPS F--F-HFD-- ---NGVQL-- ---------- ---------- ---------- ------S--- ---------- ---------- ---------- KEDLK----- ---------- ----------
 324877 LES------- ---YHDPD-D EPIADPLDPS F--F-DFD-- ---SAEPL-- ---------- ---------- ---------- ------G--- ---------- ---------- ---------- KEELK----- ---------- ----------
 117759 LEP------- ---YHDPL-D EPTAAPLDPS F--F-DFD-- ---NGNEL-- ---------- ---------- ---------- ------S--- ---------- ---------- ---------- KEQLKGISQT FF
 489827 LEP------- ---YHDPT-D EPDAEPLDPS F--F-DFDY- ---TKEPL-- ---------- ---------- ---------- ------N--- ---------- ---------- ---------- KEQLK----- ---------- ----------
 115935 LEP------- ---YHDPT-D EPDAEPLDPS F--F-DFDY- ---TKEPL-- ---------- ---------- ---------- ------N--- ---------- ---------- ---------- KEQLK----- ---------- ----------
 482781 LEP------- ---YHDPT-D EPDAEPLDPS F--F-DFDY- ---TKEPL-- ---------- ---------- ---------- ------N--- ---------- ---------- ---------- KEQLK----- ---------- ----------
 545919 LEP------- ---YHDPT-D EPDAEPLDPS F--F-DFDY- ---TKEPL-- ---------- ---------- ---------- ------N--- ---------- ---------- ---------- KEQLK----- ---------- ----------
6536562 LQP------- ---YHDAE-D EPTAEPLDPT F--F-DFD-- ---IGDPL-- ---------- ---------- ---------- ------S--- ---------- ---------- ---------- KEELK----- ---------- ----------
 309997 LEP------- ---YHDDQ-D EPAADPLPPR F--F-DFDE- ---NPEMQ-- ---------- ---------- ---------- ------S--- ---------- ---------- ---------- RTVLK----- ---------- ----------
 316873 LEP------- ---YHDDQ-D EPAADPLPPR F--F-DFDE- ---NPEMQ-- ---------- ---------- ---------- ------S--- ---------- ---------- ---------- RTVLK----- ---------- ----------
 429267 LES------- ---YHDPD-D EPIADPLDPS F--F-DFD-- ---SAEPL-- ---------- ---------- ---------- ------G--- ---------- ---------- ---------- KEELKAIDPT VL
 103465 LAAY------ ----HDSN-D EPTCEVADQA Q--F-SFD-- --NHKDQL-- ---------- ---------- ---------- ------T--- ---------- ---------- ---------- CYELK-QMLY KEVLSYRF-- ----------
 167039 LSAY------ ----HDPA-D EPVGKPIPPE F--F-AFD-- --KVKEEL-- ---------- ---------- ---------- ------T--- ---------- ---------- ---------- IPELK-TILY NEIMSS
   8637 VQDF------ ----RNPK-E ELSAERIPSD L--F-FFD-- --DYKDQL-- ---------- ---------- ---------- ------K--- ---------- ---------- ---------- MIDLK-RMLY AEIVAG
  65963 FSAY------ ----RVIQ-E ETTAVPIPKD F--F-FFD-- --DCKEQL-- ---------- ---------- ---------- ------E--- ---------- ---------- ---------- MIDLK-RMLY AEILAT
 246409 R
 246352 R
 668220 LEAY------ ----HDPE-D EPVAPPLDPE F--F-EFD-- --LHKDDI-- ---------- ---------- ---------- ------S--- ---------- ---------- ---------- REQLK-ELLY EEIMSFRP-- ----------
 619500 LESY------ ----HDPE-D EPVAPPLDPE F--F-EFD-- --LHKDDI-- ---------- ---------- ---------- ------S--- ---------- ---------- ---------- REQLK-ELLY DEIMSFQP-- ----------
  64492 LEAY------ ----HDPD-D EPCAPPLDPE F--F-EFD-- --LHKDDI-- ---------- ---------- ---------- ------S--- ---------- ---------- ---------- REQLK-ELLY EEIMSFRP-- ----------
  80250 LASY------ ----HDPD-D EPDSAPLEPS F--F-HFD-- --NGKEQL-- ---------- ---------- ---------- ------T--- ---------- ---------- ---------- REQLK-QLIY NEIMT
  21917 LEPY------ ----HDPT-D EPDAEPLDPS F--F-DFD-- --YTKEPL-- ---------- ---------- ---------- ------N--- ---------- ---------- ---------- KEQLK-VLIY EEIMRDSM
 600175 LEAY------ ----HDPQ-D EPTAEPLDPS F--F-DFD-- --YG-ETL-- ---------- ---------- ---------- ------G--- ---------- ---------- ---------- KEELK-VLIY EEVTRPRP-- ----------
 721307 LAPY------ ----HDPQ-D EPTAEPIDPQ F--F-HFD-- --NG-DPM-- ---------- ---------- ---------- ------K--- ---------- ---------- ---------- KEDLK-VLIY QEITKPDP-- ----------
 847084 LAPY------ ----HDPL-D EPKAPPINPS F--F-DFD-- --NG-GET-- ---------- ---------- ---------- ------S--- ---------- ---------- ---------- KEELK-ELIY EEITRPYV-- ----------
 280696 LEPY------ ----HDPE-D EPTAPPIPDA F--F-DFD-- --RKKDEL-- ---------- ---------- ---------- ------S--- ---------- ---------- ---------- KEELK-SKSH NALNCE
 908144 LEPY------ ----HDPE-D EPTAPTIPDE F--F-DFD-- --RRKDEL-- ---------- ---------- ---------- ------S--- ---------- ---------- ---------- KEQLK-SKLY FLSTL
 148879 LEPY------ ----HDPD-D EPTAEPIPEE F--F-SFD-- --LQKDTL-- ---------- ---------- ---------- ------S--- ---------- ---------- ---------- KEQLR-QLIY EEIMRISQ-- ----------
 102030 LAQY------ ----HDEA-D EPTCPPLPEN F--F-DFD-- --NFREKL-- ---------- ---------- ---------- ------S--- ---------- ---------- ---------- VEQLKDLLWN EIHSNFDT-- ----------
  92334 LAQY------ ----HDEA-D EPTCPPLPEN F--F-DFD-- --NFREKL-- ---------- ---------- ---------- ------S--- ---------- ---------- ---------- VEQLKDLLSF ENFVRPCS-- ----------
 146634 LGTL------ ----YQPN-D EPTCPSPFL- ---F-DDN-- --KPDDEL-- ---------- ---------- ---------- ------C--- ---------- ---------- ---------- KRDIQDQILQ EVLLFHPE-- ----------
  72485 LAEY------ ----HAEE-D EPNCPSQISI P--F-DSV-- --LQTKTH-- ---------- ---------- ---------- ------F--- ---------- ---------- ---------- SSEEQDEIRK HITEAVNT-- ----------
 124061 LTAY------ ----HDPE-D EPTVQPLSPS Y--F-EFD-- --LHKDDL-- ---------- ---------- ---------- ------S--- ---------- ---------- ---------- KDQLKELLYE EVLSFIPS-- ----------
 109792 LEAY------ ----HDPE-D EPVAPPLDED F--F-AFD-- --RQKDDI-- ---------- ---------- ---------- ------S--- ---------- ---------- ---------- REELKRLLFE EI-----E-- ----------
 408982 LEAY------ ----HDPD-D EPVAPPLDPE F--F-EFD-- --LHKDDI-- ---------- ---------- ---------- ------S--- ---------- ---------- ---------- REQLKELLYE EI-----M-- ----------
 149279 LEAY------ ----HDPD-D EPEAPPLDPE F--F-EFD-- --LHKDDI-- ---------- ---------- ---------- ------S--- ---------- ---------- ---------- REQLKQLLYE EI-----M-- ----------
  13526 LSPY------ ----HDLD-D EPTAAPLDPS F--F-DFD-- --IG-EPL-- ---------- ---------- ---------- ------T--- ---------- ---------- ---------- KEELKVRIYE EITRPEND-- ----------
 465497 LQPY------ ----HDPE-D EPTAQPLDPA F--F-DFD-- --FG-DPL-- ---------- ---------- ---------- ------P--- ---------- ---------- ---------- KEQLKVLIYE EITRSD-D-- ----------
 216389 LMPY------ ----HDVD-D EPVAEPLDPS F--F-DFD-- --VG-EPL-- ---------- ---------- ---------- ------S--- ---------- ---------- ---------- KEELKVRIYE EITRDD-L-- ----------
  17191 VACY------ ----SDEENE EPSLESLPKE Y--F-AYD-- --MAPRP--- ---------- ---------- ---------- ------S--- ---------- ---------- ---------- IEEIKKAMWE EINTLDRL-- ----------
 530963 VAPY------ ----HDPT-D EPVAA----E Q--F-DWS-- --FNDADL-- ---------- ---------- ---------- ------P--- ---------- ---------- ---------- VDTWKVMMYS EILDFHQL-- ----------
  84099 LAPY------ ----HDPT-D EPEAE----E K--F-DWS-- --FNDADL-- ---------- ---------- ---------- ------P--- ---------- ---------- ---------- VDTWKIMMYS EILDYHNV-- ----------
 928715 LQPY------ ----HDLD-D EPESEPIPAG F--F-DFDK- --PGDQAL-- ---------- ---------- ---------- ------S--- ---------- ---------- ---------- RETLKNLIAQ ELITVREP-- ----------
 480104 VSAY------ ----HDPE-D EPAATPLEPS Y--F-DFD-- --MHKDDL-- ---------- ---------- ---------- ------S--- ---------- ---------- ---------- KDQLK-ELLY EEVLSFVP-- ----------
  37144 VSAY------ ----HDPD-D TPSVASLDSD Y--F-DFD-- --QYKDDL-- ---------- ---------- ---------- ------T--- ---------- ---------- ---------- KAELK-ELLF EEIQSFVP-- ----------
 993637 VSAY------ ----HDPD-D TPSVASLDSD Y--F-DFD-- --QYKDDL-- ---------- ---------- ---------- ------T--- ---------- ---------- ---------- KAELK-ELLF EEIQSFVP-- ----------
 706327 LAAY------ ----HDPE-D EPAVSSLDSE Y--F-EFD-- --YL--DL-- ---------- ---------- ---------- ------S--- ---------- ---------- ---------- KDELK-ELLY AEVKSFVP-- ----------
   5366 LAAY------ ----HDPD-D EPAVSSLDSE Y--F-EFD-- --YL--DL-- ---------- ---------- ---------- ------N--- ---------- ---------- ---------- KDELK-DLLY AEVMSFVP-- ----------
 144998 LEAY------ ----HDPE-D EPVAPPLDPE F--F-EFD-- --LHKDDI-- ---------- ---------- ---------- ------S--- ---------- ---------- ---------- REQLK-ELLY EEIMSFRP-- ----------
 232199 LEAY------ ----HDPE-D EPVAPPLDPE F--F-EFD-- --LHKDDI-- ---------- ---------- ---------- ------S--- ---------- ---------- ---------- REQLK-ELLY EEIMSFRA-- ----------
  60405 LEAY------ ----HDPD-D EPVAPPLDPE F--F-EFD-- --LHKDDI-- ---------- ---------- ---------- ------S--- ---------- ---------- ---------- REQLK-ELLY DEIMSFRP-- ----------
 572635 LEAY------ ----HDPD-D EPIAPPLDPE F--F-EFD-- --LHKDDI-- ---------- ---------- ---------- ------S--- ---------- ---------- ---------- REQLK-ELLY EEIMSFRP-- ----------
  37912 LEAY------ ----HDPD-D EPVAPPLDPE F--F-EFD-- --LHKDDI-- ---------- ---------- ---------- ------S--- ---------- ---------- ---------- REQLK-ELLY EEIMTFRP-- ----------
 819552 LEAY------ ----HDPD-D EPVAPPLDPE F--F-EFD-- --LHKDDI-- ---------- ---------- ---------- ------S--- ---------- ---------- ---------- REQLK-ELLY EEIMSFRP-- ----------
```

```
1026351 LQPY------ ----HDPL-D EPTAEPLDPS F--F-DFD-- --YG-DPI-- ---------- ---------- ---------- ------N--- ---------- ---------- ---------- KEELK-GPCC RLG
 352542 LAPY------ ----HDPG-D EPTAEPLDPS F--F-DFD-- --NG-EQM-- ---------- ---------- ---------- ------R--- ---------- ---------- ---------- KEELK-VLIY HEITRPVE-- ----------
  42114 LMPY------ ----HDVD-D EPVAEPLDPS F--F-DFD-- --VG-EPL-- ---------- ---------- ---------- ------S--- ---------- ---------- ---------- KEELK-VRIY EEITRDDL-- ----------
 204011 LEPY------ ----HDPT-D EPDAEPLDPS F--F-DFD-- --YTKEPL-- ---------- ---------- ---------- ------N--- ---------- ---------- ---------- KEQLK-VLIY EEIMRDPL
  46962 LEPY------ ----HDPD-D EPTASPIPEE F--F-DFD-- --KNKDNL-- ---------- ---------- ---------- ------S--- ---------- ---------- ---------- KEQLK-ELIY HFLIA
   3452 LEPY------ ----HDPD-D EPTADPIPEE F--F-DFD-- --KNKDNL-- ---------- ---------- ---------- ------T--- ---------- ---------- ---------- KEQLK-LLIY QEIMR
1531701 LEPY------ ----HDPD-D EPTAPPIPEE F--F-DFD-- --KHKDNL-- ---------- ---------- ---------- ------S--- ---------- ---------- ---------- KEQLK-QLIY QEIMR
 375250 LEPY------ ----HDPD-D EPTADPIPEE F--F-DFD-- --KNKDNL-- ---------- ---------- ---------- ------T--- ---------- ---------- ---------- KEQLK-QITS PIFPPSPP-- ----------
 391393 LEPY------ ----HDPE-D EPTADPIPEE F--F-DFD-- --KKKDNL-- ---------- ---------- ---------- ------S--- ---------- ---------- ---------- KEQLK-RLQQ QSENQGRR-- ----------
 518679 LEPY------ ----HDPD-D EPTAPPIPDG F--F-DFD-- --KNKDAL-- ---------- ---------- ---------- ------S--- ---------- ---------- ---------- KEQLK-YFKE SEAICPIH
1272884 LEPY------ ----HDPD-D EPTAPPIPEE F--F-DFD-- --K----L-- ---------- ---------- ---------- ------C--- ---------- ---------- ---------- GEQEH-KMDE RAVVGTCW-- ----------
 108249 LEQY------ ----HDPE-D EPIAPPVPES F--F-DFD-- --RYKEQL-- ---------- ---------- ---------- ------S--- ---------- ---------- ---------- KEQLK-GN
 111885 LATY------ ----HDED-D EPCYKEIDVV G--DKFWRLD S-DVNSQS-- ---------- ---------- ---------- ------W--- ---------- ---------- ---------- EQSEENEELM ESLKRMLY-- ----------
 228039 LAMY------ ----HDPS-D EPTITPIEL- ----EEFLIS K-NENPNL-- ---------- ---------- ---------- ------N--- ---------- ---------- ---------- DLKIELFEQV MNLK
 117728 VAQF------ --RKIDEELS APRKFKIGSN D--NIKYTVD DYRNHKQS-- ---------- ---------- ---------- ------T--- ---------- ---------- ---------- TKQLQSEVLW QNLLYKRT-- ----------
 267276 LEAY------ ---------V RPSLFFFLQC Q--ILIY--- ---FSSCI-- ---------- ---------- ---------- ------S--- ---------- ---------- ---------- TTQKTSPSHH PSTPSSSN-- ----------
 295270 LEAY------ ---------C TKTISAANSS R--NCSM--- ---RKSCP-- ---------- ---------- ---------- ------S--- ---------- ---------- ---------- IRRPWCRRAH FTRTSTS
 174214 LAPY------ ----HDPT-D EPEAEEKFDW S--FNDADLP VDTWKIMM-- ---------- ---------- ---------- ------Y--- ---------- ---------- ---------- SEILD--YHN VD----TA-- ----------
 111562 LAPY------ ----HDPT-D EPEAEEKFDW S--FNDADLP VDTWKIMM-- ---------- ---------- ---------- ------Y--- ---------- ---------- ---------- SEILD--YHN VDQGNEAT-- ----------
 385026 LSPY------ ----HDPT-D EPEADDKFDW S--FNDADLP VDTWKIMM-- ---------- ---------- ---------- ------Y--- ---------- ---------- ---------- SEILD--YHN VD-----S-- ----------
  47937 LAPY------ ----HDPT-D EPEAEEKFDW S--FNDADLP VDTWKIMM-- ---------- ---------- ---------- ------Y--- ---------- ---------- ---------- SEILD--YHN VDAQNGEE-- ----------
 191658 LAPY------ ----HDPT-D EPVADEKFDW S--FNDADLP VDTWKIMM-- ---------- ---------- ---------- ------Y--- ---------- ---------- ---------- SEILD--YHN VD-------- ----------
 361397 LAPY------ ----HDPT-D EPVAEEKFDW S--FNDADLP VDTWKIMM-- ---------- ---------- ---------- ------Y--- ---------- ---------- ---------- SEILD--YHN VD-------- ----------
 368356 LAPY------ ----HDPT-D EPGAEEKFDW S--FNDADLP VDTWKIMM-- ---------- ---------- ---------- ------Y--- ---------- ---------- ---------- SEILD--YHN VD-------- ----------
 422287 LAPY------ ----HDPT-D EPSADEKFDW S--FNDADLP VDTWKIMM-- ---------- ---------- ---------- ------Y--- ---------- ---------- ---------- SEILD--YHN VD-------- ----------
1051981 LAPY------ ----HDPT-D EPVAEEKFDW S--FNDADLP VDTWKIMM-- ---------- ---------- ---------- ------Y--- ---------- ---------- ---------- SEILD--YHN VDTNMGQL-- ----------
 512786 LAPY------ ----HDPS-D EPVAEEKFDW S--FNDADLP VDTWKIMM-- ---------- ---------- ---------- ------Y--- ---------- ---------- ---------- SEILD--YHN VESNNPPA-- ----------
 700253 LAPY------ ----HDPT-D EPVAEEKFDW S--FNDADLP VDTWKIMM-- ---------- ---------- ---------- ------Y--- ---------- ---------- ---------- SEILD--YHN VDAATQQM-- ----------
 171641 LAPY------ ----HDPS-D EPVAEEKFDW S--FNDADLP VDTWKIMM-- ---------- ---------- ---------- ------Y--- ---------- ---------- ---------- SEILD--YHN MESSAGVM-- ----------
 447383 LAPY------ ----HDPT-D EPVAEEKFDW S--FNDADLP VDTWKIMM-- ---------- ---------- ---------- ------Y--- ---------- ---------- ---------- SEILD--YHN VENNLPSA-- ----------
 134451 LAPY------ ----HDPT-D EPVAEEKFDW S--FNDADLP VDTWKIMM-- ---------- ---------- ---------- ------Y--- ---------- ---------- ---------- SEILD--YHN VESDSNQP-- ----------
 525556 LAPY------ ----HDPT-D EPEAQEKFDW S--FNDADLP VDTWKIMM-- ---------- ---------- ---------- ------Y--- ---------- ---------- ---------- SEILD--FHN IDQSEDAG-- ----------
1776212 LAPY------ ----HDPT-D EPVAGEKFDW S--FNDADLP VDTWKIMM-- ---------- ---------- ---------- ------Y--- ---------- ---------- ---------- SEILD--YHN VDGSTQS
 443108 LTPY------ ----HDPS-D EPVAEEKFDW S--FNDADLP VDTWKIMM-- ---------- ---------- ---------- ------Y--- ---------- ---------- ---------- EELID--FQQ TSPE
 129540 LAPY------ ----HDPT-D EPVAKEKFDW S--FNDADLP VDTWKIMM-- ---------- ---------- ---------- ------Y--- ---------- ---------- ---------- SEILD--YHN VDQVAPEG-- ----------
 341724 LAPY------ ----HDPT-D EPVANEKFDW S--FNDADLP VDTWKIMM-- ---------- ---------- ---------- ------Y--- ---------- ---------- ---------- SEILD--YHN VDQAAPES-- ----------
 138417 LAPY------ ----HDPT-D EPVAKEKFDW S--FNDADLP VDTWKIMM-- ---------- ---------- ---------- ------YAIS SRLPVLSVLQ PGIIRLYQDY TLTIGLSNRY SEILD--YHN VDQVAPEG-- ----------
 158191 LSPY------ ----HDPS-D EPVAEEKFDW S--FNDADLP VDTWKIMM-- ---------- ---------- ---------- ------YDGP AAQLMGA--- ---------- ---------- LLITN--VAG TPRSSTTT-- ----------
 376076 LAPY------ ----HDPT-D EPVAEEKFDW S--FNDADLP VDTWKIMM-- ---------- ---------- ---------- ---------- -RQAQST--- ---------- ---------- ATILR--FKG NLRTTLQT-- ----------
 368607 LAPY------ ----HDPS-D EPVAAEKFDW S--FNNADLP TDTWKIMI-- ---------- ---------- ---------- ------G--- ---------- ---------- ---------- NEINE--FAW GGAIAPDE-- ----------
1312592 LGPY------ ----HDPT-D EPEANEKFDW S--FNDADLP VDTWKVMM-- ---------- ---------- ---------- ------Y--- ---------- ---------- ---------- SEILD--FHN VDSTAMVN-- ----------
  46955 VSPY------ ----HDST-D EPEAAEKFDW S--FNDADLP VDTWKVMM-- ---------- ---------- ---------- ------Y--- ---------- ---------- ---------- SEILD--FHQ VGDARLAA-- ----------
1164008 VAPY------ ----HDPT-D EPEAAEKFDW S--FNDADLP VDTWKVMM-- ---------- ---------- ---------- ------Y--- ---------- ---------- ---------- SEILD--FHQ VGDSRLEN-- ----------
 600771 VAPY------ ----HDPT-D EPEAAEKFDW S--FNDAELP VDTWKVMM-- ---------- ---------- ---------- ------Y--- ---------- ---------- ---------- SEILD--FHQ VGDSRLND-- ----------
 161654 VAPY------ ----HDPT-D EPEASEKFDW S--FNDADLP VDTWKVMM-- ---------- ---------- ---------- ------Y--- ---------- ---------- ---------- SEILD--FHQ VGESTLLD-- ----------
 149140 VAPY------ ----HDPT-D EPEASEKFDW S--FNDADLP VDTWKVMM-- ---------- ---------- ---------- ------Y--- ---------- ---------- ---------- SEILD--FHQ VGESTLLD-- ----------
 474692 VAPY------ ----HDPT-D EPEAEEKFDW S--FNDADLP IDTWKVMM-- ---------- ---------- ---------- ------Y--- ---------- ---------- ---------- SEILD--FHQ VDQG--QG-- ----------
 134758 VAPY------ ----HDPT-D EPEAADKFDW S--FNDADLP VDTWKVMM-- ---------- ---------- ---------- ------Y--- ---------- ---------- ---------- SEILD--FHQ VGEATTNA-- ----------
 177791 VALY------ ----HDPT-D EPVAGEKFDW S--FNDADLP VDTWKVMM-- ---------- ---------- ---------- ------Y--- ---------- ---------- ---------- SEILD--FHQ VADGGAVS-- ----------
 132015 VSPY------ ----HDPT-D EPVAAEKFDW S--FNDADLP VDTWKVMM-- ---------- ---------- ---------- ------Y--- ---------- ---------- ---------- SEILD--FHQ VGEASITA-- ----------
 917530 VAPY------ ----HDPT-D EPVAGEKFDW S--FNDADLP VDTWKVMM-- ---------- ---------- ---------- ------Y--- ---------- ---------- ---------- SEILD--FHQ VGDPSTTS-- ----------
 428496 VAPY------ ----HDPS-D EPEAEEKFDW S--FNDADLP TDTWKVMM-- ---------- ---------- ---------- ------Y--- ---------- ---------- ---------- SEILE--FHQ VDGDGQAS-- ----------
1276586 VSPY------ ----HDPS-D EPAAAEPFDW S--FNDAELP VDTWKVMM-- ---------- ---------- ---------- ------Y--- ---------- ---------- ---------- SEILD--FHN IGNTGYED-- ----------
 115601 VAPY------ ----HDPT-D EPVAAEQFDW S--FNDADLP VDTWKVMM-- ---------- ---------- ---------- ------Y--- ---------- ---------- ---------- SEILD--FHQ LGDADGLD-- ----------
 171809 VSPY------ ----HDPT-D EPVASDPFDW S--FNDADLP VDTWKVMM-- ---------- ---------- ---------- ------Y--- ---------- ---------- ---------- SEILGGCHTL PSTSYFSF-- ----------
 571775 VSPY------ ----HDPT-D EPVAAEKFDW S--FNDADLP VDTWKVMM-- ---------- ---------- ---------- ------Y--- ---------- ---------- ---------- SEILGKSFCV IAVEP-SF-- ----------
 722302 LSPY------ ----HDPT-D EPIASEPFDW S--FNDADLP VDTWRVMM-- ---------- ---------- ---------- ------Y--- ---------- ---------- ---------- SEILD--FHD VKEPGSVT-- ----------
 289694 LAPY------ ----HDPT-D EPEADEPFDW S--FNDADLP VDTWKVMM-- ---------- ---------- ---------- ------Y--- ---------- ---------- ---------- SEILD--FHN IDDAQGQS-- ----------
 137978 LAPY------ ----HDPT-D EPVAPEKFDW S--FNEADLP IDTWKVMM-- ---------- ---------- ---------- ------Y--- ---------- ---------- ---------- SEILD--FHN VDDIDGSQ-- ----------
  19116 IECY------ ----HDES-D EPVATEKFDW S--FSDSDLP VDTWKVMM-- ---------- ---------- ---------- ------Y--- ---------- ---------- ---------- SEIMD--FHS AGGNNTQQ-- ----------
 129801 LQPY------ ----HDPS-D EPVAGEKFDW S--FNDADLP VDTWRVMM-- ---------- ---------- ---------- ------Y--- ---------- ---------- ---------- SEILD--FHH VDSG------ ----------
  80382 LAPY------ ----HDPT-D EPIAEERFDW S--FNDADLP VETWKIMM-- ---------- ---------- ---------- ------Y--- ---------- ---------- ---------- SEILD--FHE IEGAEAMH-- ----------
 141916 LAPY------ ----HDPT-D EPIAEERFDW S--FNDADLP VETWKIMM-- ---------- ---------- ---------- ------Y--- ---------- ---------- ---------- SEILD--FHE IEGAEAMH-- ----------
 187748 MEPY------ ----HDPT-D EPVCEQKFDW S--FNDVDLP VDTWRVMM-- ---------- ---------- ---------- ------Y--- ---------- ---------- ---------- SEILD--FHH LVGG------ ----------
 168984 LAPY------ ----HDPS-D EPVAEEKFDW S--FNDAELP IDTWRVMM-- ---------- ---------- ---------- ------Y--- ---------- ---------- ---------- SEILD--FHQ VDNMQQ
  71808 LETY------ ----HDEA-D EPMAESKFDW S--FNDADLP VEEWKVMM-- ---------- ---------- ---------- ------Y--- ---------- ---------- ---------- NEILD--FLN VSEGPTAI-- ----------
    432 LANY------ ----HVPS-D EPVAEEKFDW S--FNEAELP IETWKAMI-- ---------- ---------- ---------- ------Y--- ---------- ---------- ---------- AEILD--FHR LDDSDLDL-- ----------
  59395 LAPY------ ----HDPD-D EPTAPVAFDW S--VTSQDLT AEQWKVLM-- ---------- ---------- ---------- ------F--- ---------- ---------- ---------- NGVLS--FHQ RENQQVYP-- ----------
   2865 LTPY------ ----HDPS-D EPVAEEKFDW S--FNDADLP VDTWKIMM-- ---------- ---------- ---------- ------Y--- ---------- ---------- ---------- SEILD--YHN VDQTQEGG-- ----------
 189766 LAPY------ ----HDPT-D EPSADEKFDW S--FNDADLP VDTWKIMM-- ---------- ---------- ---------- ------Y--- ---------- ---------- ---------- SEILD--YHN VDAAAQEE-- ----------
  88183 LAPY------ ----HDPT-D EPAAAEKFDW S--FNDADLP VDTWKVMI-- ---------- ---------- ---------- ------YSEI LGMFS
 216492 VAPY------ ----HDPS-D EPVAAEKFDW S--FNDADLP IDTWKVMM-- ---------- ---------- ---------- ------Y--- ---------- ---------- ---------- GEILG--LV
 188879 VAPY------ ----HDPS-D EPVAAEKFDW S--FNDADLP IDTWKVMM-- ---------- ---------- ---------- ------Y--- ---------- ---------- ---------- GEILD--FHQ TGDVIPPE-- ----------
 182850 VAPY------ ----HDPS-D EPVAAEKFDW S--FNDADLP IDTWKVMM-- ---------- ---------- ---------- ------Y--- ---------- ---------- ---------- GEILD--FHQ TGDVIPPE-- ----------
 174697 VAPY------ ----HDPS-D EPVAAEKFDW S--FNDADLP IDTWKVMM-- ---------- ---------- ---------- ------Y--- ---------- ---------- ---------- GEILD--FHQ TGDVIPPE-- ----------
 934899 VAPY------ ----HDPS-D EPVAGEQFDW S--FNDADLP VDTWKVMM-- ---------- ---------- ---------- ------Y--- ---------- ---------- ---------- SEILD--FHQ VGEPATSP-- ----------
 362397 LAPY------ ----HDPS-D EPSAAEVFDW S--FNDADLP VDTWKVMM-- ---------- ---------- ---------- ------Y--- ---------- ---------- ---------- SEILD--FHN LGDISQAD-- ----------
 416973 VAPY------ ----HDPS-D EPVAGEKFDW S--FNDADLP VDTWKVMM-- ---------- ---------- ---------- ------Y--- ---------- ---------- ---------- GEILD--FHQ VADGTGIA-- ----------
 551386 VAPY------ ----HDPT-D EPVAAEQFDW S--FNDADLP VDTWKVMM-- ---------- ---------- ---------- ------Y--- ---------- ---------- ---------- SEILD--FHQ LGDTDGLD-- ----------
  15239 VAPY------ ----HDPT-D EPVAAEQFDW S--FNDADLP VDTWKVMM-- ---------- ---------- ---------- ------Y--- ---------- ---------- ---------- SEILD--FHQ LGDADGLD-- ----------
 497504 LSPY------ ----HDPT-D EPVAEEKFDW S--FNDADLP VDTWKIMM-- ---------- ---------- ---------- ------YEQL YEYQDETSQG
1117682 VSPY------ ----HEPA-D EPVAASPFDW S--FNEADLP METWKVMM-- ---------- ---------- ---------- ------YSEI V
 252496 VAPY------ ----HDPT-D EPEAEEKFDW S--FNDADLP IDTWKVMM-- ---------- ---------- ---------- ------Y--- ---------- ---------- ---------- SEILGKSFCF TRRCFCVN-- ----------
 148867 LSMY------ ----HDPE-D EPEATNEFDW S--FTDAELS LEQWQEMT-- ---------- ---------- ---------- ------L--- ---------- ---------- ---------- RDIEDFRKQQ VAEEAAAT-- ----------
 423027 LGVY------ ----HDPT-D EPVAEQKFDW S--FSEVAHS IDEWKIMI-- ---------- ---------- ---------- ------Y--- ---------- ---------- ---------- TEVVDFHDLG PTGEPAIT-- ----------
1104996 LSVY------ ----HDPT-D EPVAEQMFDW S--FSEVAHS IDEWKIMI-- ---------- ---------- ---------- ------Y--- ---------- ---------- ---------- TEVVDFHDIG
  29266 LAPY------ ----HDPT-D EPVAEEKFDW S--FNDADLP VDTWKIMM-- ---------- ---------- ---------- ------Y--- ---------- ---------- ---------- SEILD--YHN VENNLP---- ----------
 171653 LAPY------ ----HDPS-D EPVAEEKFDW S--FNDADLP VDTWKIMM-- ---------- ---------- ---------- ------Y--- ---------- ---------- ---------- SEILD--YHN MESSAG---- ----------
 117063 LSPY------ ----HDPT-D EPVAEEKFDW S--FNDADLP VDTWKIMM-- ---------- ---------- ---------- ------Y--- ---------- ---------- ---------- SEILD--YHN VETGDDNH-- ----------
 158104 LSPY------ ----HDPS-D EPVAEEKFDW S--FNDADLP VDTWKIMM-- ---------- ---------- ---------- ------Y--- ---------- ---------- ---------- SEILD--YHN VDAQSAD--- ----------
 160940 LAPY------ ----HDPT-D EPVAEEKFDW S--FNDADLP VDTWKIKM-- ---------- ---------- ---------- ------Y--- ---------- ---------- ---------- SEILD--YHN VDAQGSG--- ----------
   2813 LTPY------ ----HDPS-D EPVAEEKFDW S--FNDADLP VDTWKIMM-- ---------- ---------- ---------- ------Y--- ---------- ---------- ---------- SEILD--YHN VDQTQEG--- ----------
 129541 LAPY------ ----HDPT-D EPVAKEKFDW S--FNDADLP VDTWKIMM-- ---------- ---------- ---------- ------Y--- ---------- ---------- ---------- SEILD--YHN VDQVAPEG-- ----------
 257371 LAPY------ ----HDPS-D EPVAAEKFDW S--FNNADLP TDTWKIMM
 389086 LVNF------ ----HDPE-D EPEASEEINM S--YDEVELS PEEWKKIM-- ---------- ---------- ---------- ------Y--- ---------- ---------- ---------- REVLD--FHE GSAPSKPG-- ----------
  73401 LQIY------ ----DDPT-D EPSVERPFDW S--FDGARLS KETLKLMM-- ---------- ---------- ---------- ------HVSP VSLIYAACR- ---------- ---------Y AEVLD--YHQ SDELAHPV-- ----------
1139913 LQIY------ ----HDPT-D EPSVERPFDW S--FDGAQLS KETLKLMM-- ---------- ---------- ---------- ------HVTP V--------- ---------- ---------Y SEVLD--YHQ ADELAHPV-- ----------
1168371 LQIY------ ----HDPT-D EPSVERPFDW S--FDGAQLS KETLKLMM-- ---------- ---------- ---------- ------HVTP V--------- ---------- ---------Y SEVLD--YHQ ADELAHPV-- ----------
 698519 LQIY------ ----DDPT-D EPSVERPFDW S--FDGARLS KETLKLMM-- ---------- ---------- ---------- ------HVSP VSLIYAYHL- ---------- ---------L TQSMQ--ICR SAGLS-PI-- ----------
 332332 LAPY------ ----HDPT-D EPVANEKFDW S--FNDADLP VDTWKIMM-- ---------- ---------- ---------- ------Y--- ---------- ---------- ---------- SEILD--YHN VDQAAPES-- ----------
 243292 LAPY------ ----HDPT-D EPEAEEKFDW S--FNDADLP VDTWKIMM-- ---------- ---------- ---------- ------Y--- ---------- ---------- ---------- SEILD--YHN VDTAAAEP-- ----------
 243775 LAPY------ ----HDPT-D EPEAEEKFDW S--FNDADLP VDTWKIMM-- ---------- ---------- ---------- ------Y--- ---------- ---------- ---------- SEILD--YHN VDTAAAEP-- ----------
  24163 LAPY------ ----HDPT-D EPEAEEKFDW S--FNDADLP VDTWKIMM-- ---------- ---------- ---------- ------Y--- ---------- ---------- ---------- SEILD--FHN VDSAQAEQ-- ----------
1431451 LAPY------ ----HDPT-D EPVAGEKFDW S--FNDADLP VDTWKIMM-- ---------- ---------- ---------- ------Y--- ---------- ---------- ---------- SEILD--YHN VDGSTQS
  99398 LAPY------ ----HDPT-D EPVANEKFDW S--FNDADLP VDTWKIMM-- ---------- ---------- ---------- ------YE-- ---------- ---------- ---------- SPALD--CCY SISGSSVH-- ----------
 171665 LAPY------ ----HDPS-D EPVAEEKFDW S--FNDADLP VDTWKIMM-- ---------- ---------- ---------- ------Y--- ---------- ---------- ---------- SEILD--YHN MESSAGVM-- ----------
  15876 LVNF------ ----HDPE-D EPEASEEINM S--YDEVELS PEEWKKIM-- ---------- ---------- ---------- ------Y--- ---------- ---------- ---------- REVLD--FHE GSAPSKPG-- ----------
  43014 QEIMQ
  43498
1148336 LEPY------ ----HDPD-D EPTAPPIPEG F--FDFDKNK DALSKEQL-- ---------- ---------- ---------- ------KLLI YEEIMR
 220391 LAAY------ ----HDPE-D EPVVTPLSPS Y--FEFDLHK DDLSKDQL-- ---------- ---------- ---------- ------KELL YEEVLSFVPS I
 774567 LTAY------ ----HDPD-D EPAVSSLDAD Y--FECDSDL D-LSKQEL-- ---------- ---------- ---------- ------KELL YEEVMSFTPA I
 138915 LTAY------ ----HDPD-D EPAVSSLDAD Y--FECDY-L D-LSKQEL-- ---------- ---------- ---------- ------KELL YEEVMSFTPA I
 757677
 313819
 290180
  12232
 865310
 312015
 500478
 500460
 243933
  64065
   2938
 196733
 145976
 501440
  71512
 543038
  99402
1431403
 342880
 417966
```

```
 Consensus  l..y          h#p. D EP........ f..f...d.. .......... .......... .......... .......... .......... .                                    ...l...... ........

            1351                                                                                                                                                    1500
      6423  --------QQ YGREDPRPQE MAG------- -QV---PNQD LERELQGGLD ARRK
    160078  --------RA Y--ENPRPQE VPD------- -RT---AN-D LERELAMQ
    116650  --------RA Y--EDPRPQE LAQ------- -AP-GGAQHD LERELQ-GLD AMRQ
    377992  --------GR Y--EDPRPQE TTS------- -FA---NGQD LERELQGGLD AMR
    289793  --------RA Y--EDPRPHE A--------- -NA-AMRDAG LEQELQAGMD RR
    670269  --------RA Y--EDPRPQE AQQ------- -YM-G---HG LESDLQGLDG RNMAHHSMR
     32808  --------TG Y--EEPRPQE AFN------- -QG-GWNGSD LERDLQGLDG RMR
    156295  --------TG Y--EEPRPQE AFN------- -QV-AVDAEP DVKSVRASDM VRAYTRVL
     22314  --------TG Y--EEPRPQE AFN------- -QG-GWNGSD LERDLQGLDG RMR
    417978  --------AG Y--EDPRPQE AFQ------- -QQ-G-QWND LERDLQGLDG RM
    414783  --------SG F--ENPRPQE AAM------- -GG-GM--EG LEGELAGGMD MKMRG
   1074946  --------Q- WTADEPRPQE YVG------- ------QGNG LEADLAGGLD GRR
    865350  --------G- WTSSDPRPQE YLG------- -HG---QGNG LEADLAGGLD GRR
   1656563  --------G- WSSSDPRPQE YGG------- -QG---DKAI ASSSWIGTLR ENAPE
    333276  --------Q- WRTEDPRPEE YLG------- -HS---QG-G LEADLAMDDL LSPGEADLWS RTCQWLFDED LENGILTTGS SS------LD IDRHLGEEDE ---------- -DDTAENQKK RNCGELTGRS PVV--EDLSP DGQRHYWARE
    422586  --------Q- WRAEDPRPQE YMN------- ------QVGG LEGELQGGLD ASRR
    342887  --------Q- WTHEDPRPQE YGG------- ------QAGN LEAELQGGLD RR
    386771  --------Q- WSAEDPRPQE YTN------- ------QMG- LEGELQGGLD AGVRR
     14976  --------Q- WRAEDPRPQE YVD------- ------QMG- LEGELQGGLD AGVRR
    130981  --------S- WGAEDPRPQE AYG------- -Q----GATG LEQDLQGGLD AMHA
    435286  --------S- WGAEDPRPQE AYG------- -Q----SSQG LEQDLERGLD AMRG
   1457372  --------G- WSSSDPRPQE YGG------- ------QGGG LEADLAGGLD HRR
    249807  --------G- WKTEEPKPQE ALA------- -AG-GGHHND LESSLQRGMD VQ
   1142566  --------G- WKTEEPKPQE ALA------- -AG-GGHHND LESSLQRGMD VQ
   1008492  --------G- WKTEEPKPQE ALA------- -AG-GGHHND LESSLQRGMD VQ
    152510  --------AS WRAEDPRPQE AAG------- -AGNAGQMSD LEASLQRGMD AMH
    336937  --------AS WREEDPRPQE A-G------- -AGVAGQMSD LEASLQRGMD AMH
   1649893  --------QQ QRNDAPRPHD APR------- PHDQQFNEAN LEQELAGGLD VMHQ
    371270  --------GG WLGCHDVLEG LF-------- -------CAL LAPPLSLLV
     82103  --------AG Y--EDPRPQE AFN------- QGG--WQGSD LERDLQGLDG RLR
     42859  --------R- -QAEDPRPQP LGE------- -----STNEG LERELEYGLD AQYGQQQMR
    212507  ---------- WRAEDPRPQE YMN------- -----QVG-G LEGELQGGLD ASRR
    212502  ---------- WRAEDPRPQE YMN------- -----QVG-G LEGELQGGLD ASRR
    461664  ---------- WGAEDPRPQE AYG------- -----QSSQG LEQDLERGLD AMRG
     39613  --------ES AYDEVPKPQE NSG------- ----NTDTAD LEKELEYGLD ALH
     91135  --------QL EQYQQQQQQQ QQQ------- QQYQAQAQAQ AQAQAQAQAQ AQAQAQAQAH AHAQAQIEAQ MQKQYQYQQY QQQYQAQIAQ AQAVQAAQGQ ---------- -NQAQNQGLI AQVGQLDFRT PAVNEEDLPP KPQENIYIQQ
    103403  --------N- LQDHYAQQMA EHH------- QIQQGHLEAA KELQSQADQF Y-------AN PNYQMLSTPT MLQNIPNDSF LDD-----LP PRPQENVFSD ---------- -PSDILGLEQ ELEFGLDRNL GFI
    112360  --------E- DQYS--EQQY QQD------- QIHESVPAVP ASEQQQQQQF QFGTP-IQQS FFPQAGESPV GQIGIPQGQF TSD-----LP PKPEE---VY ---------- -NDSFIDLER ELGYGLDGIR
      4266  --------Q- QQHSDFERTQ QNF------- QIQSPQETIQ ESPGIMSPRA TFASPEATPQ VDLHEHPSMQ GSLRTPTGAS SNDPD---IP PRPEE---AT ---------- -DDGEMDLEH DLVFGAERYN
    168486  --------Q- QQQQQQQQQQ QQQ------- QQQQQQQQQQ QQQQQQQQQQ YDLDYPTSVN TNPQLNENTQ YYQSIPKPQE LDDFNFHGLN DNVDR---VD ---------- -NSNLFKLEE ELGFGLDGAS MFNNL
     35227  --------QP QLKTPFEGQV ADY------- ELPPKPQEML YDENIDLERE LAYGLDGYNF QYQ
     67581  --------QP QLKTPFEGQV ADY------- ELPPKPQEML YDENIDLERE LAYGLDGYNF QYQ
     59367  --------NN NSSSSNNNNS SNI------- GISADNLPAH KSLSNENNNS EKFHQDYNNV ISEFLDLEKE LEFGLDGNNF EQ
    336175  --------TL ERSKEAAAKS NEA------- AMHGDYNPNR NSSSIAAGAV PTQNDDIRQM DVDALERELG GACAQISIP
    177826  --------TL ERSQQVAAQS RTV------- DGAAQDTHAT ASGSVAGSTV A--NPPVNHM EVDELERELS GHM
    212434  --------AL ERSKQAAAQQ PR-------- -GGEQEANVA AGGSVAASAV G--NHAVHHM EVDELERELS GRA
    164796
    100890  --------AQ NNFSGNDTFA MAR------- EYRSVDDSLE PGVAAQHQSY MDDKSMLNPS DAATLERELS GEVPMH
    288633  --------NM EHDTGYADQL VAS------- PVGVDPKLER DLGGF
     69432  --------QE HAPPHGATSG FTMSS-MG-- QRPASPVYDD PSADLEREL- ASTHLGKR
     60279  --------QE YGHVSGATSG FTHSV----- QRPASPIIDD PSAELEREL- ASTHLG
    166223  --------QE YGPHHGATSG YTQQN-PNGH ARAASPVMDD PSKELEREL- AGTHLGRR
   1111368  --------QE YGPLHGATSG FTAPS-H--- -RPASPVMDD PSDALEREL- AGTHLSKR
   7180514  --------QE YGPHHGATSS FTAVQ-NN-- ARPPSPIMDD PSEELEREL- AGTHLGAGKR
    671172  --------QE HGPHHGATSS YTAQS-HGG- ARPPSPIMDD PSEELEREL- ASTHLGITRR
     69595  --------QE YGPHHGATSS FTAAH-TA-- ERPPSPIMDD PSEELEREL- ASTHLGKRG
    188428  --------QE YGPHHGATSS FTAAA-TT-- ERPPSPIMDD PSEELEREL- ASTHLAGRR
    799680  --------HE YGPVTGATSG FTAPS-S--- RKPPSPVMED PSDALEKEL- AGTHIGR
    178052  --------QE HGPVPGATSG FTAPS-Q--- R-PPSPIMDD PSDALEKEL- AGTHIGKR
    129301  --------QD HAPPNGITSG YTAPN----- ARPPTPVMDD PSAELEREL- AGTHIGRT
    208817  --------QE DAPSNGATSG YTAPG----- QRVPSPVMDD PSADLEREL- AGTHLGNK
    424444  --------QG Q---EGTTSN FTQHP-AN-- NRPPTPTVDD PSAELEKEL- AGTHISR
    830405  --------QG Q---EGTTSN FTQPV-QA-- NRPPSPTMDD PSESLEREL- ASTHLGGGRR K
    892596  --------QE HGPVSGATSG YTGST-G--- RPQGSPVMDD PSAELDREL- AKKARK
    186189  --------T- HAPPNGATSG YT-VS-DQ-- QRPVSPVTED PSAELEKEL- ASTHLGSRR
    164780  --------RE YAPPNGATSN FTGAA-VG-- GRPSSPIMDD PGADLEREL- AGAGINGKKY
    167312  --------TE QATTNGATSN YTGAK-SE-- ARPGSPILDD PSAELEREL- S--HIRR
    119588  --------TD QAPPNGATSH YTGAA-GE-- ARPGSPIIDD PSAELEREL- ASTHIARR
    423592  --------QE DAPTNGATSG YYSGN-PVS- ERAPSPVMDD PSAELEREL- AETHIGRS
    474667  --------KE FGPHPGHTSN YTTNA-P--- -RAPSPIMDD PSAELEREL- ASTHISKK
    459613  --------KE FGPHPGHTSN YTTNA-P--- -RAPSPIMDD PSAELEREL- ASTHISKK
    156206  --------QE FQYVDRHASA G--------- KRPRSPVLDD PSEEFEREL- SSTHL
    791811  --------LE NAPSNGSTSG FTESL-P--- -RAESPVMDD PSAELEREL- AGTHLGRA
    146905  --------NE YAPD------ -PSER-M--- -NTASPVLDD PSAELEREL- ADTHIGQGGA GGDELNTESA SNANT-RVNS LPSQQTQADK -EGVRAPRKS ---------- MSSSTRDASE RAATPPRRAE SPSVQGQAQG NRGMDDVHGD
    715329  --------LE QAYA------ -EPGQ-A--- -RADSPVMDD PSAELEREL- AGTHLGRVWN KSIQSLVERY WPASMCPLSG RWMRSTRSDK AEGVRTSRTS APREDGAEHD LNTNSPELRT NRGLPCCALR LSVVLRDSGL GPGVRICQNQ
    511921  --------RE N--D------ -ATSG-Y--- -TFPHPNAPA PSGDMVEEP- GQELDRELGA VRFFFQPKEQ KPFLGRRKDG RLHGDSRDFE RLDIGA---- ---------- -GDPVPELAE EPPAGELLLA AEWVLAEAEA EEPAERKQQG
    737951  --------RE NAPPNGATSG FTEAQ-P--- -RADSPVLDD PSAELEREL- AGTHLGKRAS QPTGSWIEAA A-VSATSQSA FVQWSGSSSA TQRAKCNR-- ---------- -RAPRGPLQT CRGTWTRCFI SRLTVGIVGV NNGKRYSLRE
    545584  --------RE ND-------- -ATSG-Y--- -TFPHPNAPA PEGDMVEEP- GQELDRELGA YGQR
    102765  --------VE ND-------- -GTSG-Y--- -TFPHPSAPG PSGGMVEEP- GAELDKELGA FQIWEKRAAD EVGLIFYEKE RSKTEQE
    203555  --------NE GAPQHGATSS LTAAH-IG-- EGPYSPVIDE PSAELEREL- AVADNDG
    302829  --------QE HGPHHGATSS YTAQS-HGG- ARPPSPIMDD PSEELEREL- ASTHLGITRR
    701651  --------LE NAPSNGSTSG FT--E-SL-- PRAESPVMDD PSAELEREL- AGTHLGRA
    972433  --------RE YGDVPGATSG YTV-S-A--- HRPPSPVMDN PSDELDREL- ARTHAGKR
    931864  --------RE YGDVPGATSG YTV-S-A--- HRPPSPVMDN PSDELDREL- ARTHAGKR
    813292  --------HE YGPVTGATSG FTAPS-S--- RKPPSPVMED PSDALEKEL- AGTHIGR
     78187  --------QE YGPHHGATSS FTAAA-TT-- ERPPSPIMDD PSEELEREL- ASTHLAGRR
    406308  --------PE -----GQNGG WKHEE-PK-- PQ-EAAGGGG PASDLEASL- ARGMDAHHR
    118382  --------RV TVERGHPARL WMIPA-KNWS ESLPLLILGL RGDEMPIIC- LDTLNGNLTSC CVLWTKYLTY TVAGIFAWGN YSLAIPGSFI FTMCFLPLYA ---------- VVAPAIGFST EYSGIVHRLW TSSVFYFVLV FIPIFCLVRD
    100328
   1411022  --------AE HAPANGFTSA YTAKP-GAH- GLHDSPILED PSQDLEREL- ANAQARAQAQ VA
    741167  --------AE HAPANGITSG YTAKP-SER- GLTGSPMLDD PSHELDREL- ADAQLA
   1338511  --------HE QAPANGATSS YTVPAEPQLD PDTNTPVVDN PTEDLEREL- DRRLRVSDNL GQDRKSTIAN EDKVGRRCVE EELAKQGAF
    756718  --------RD DAPRDGATSN YTLPAQSQVD PDTDTPIVDN PTEDLEREL- DQRLHVSQK
    302653  --------EV PEPTQPAA-- ---------- -RAPTPFEED PSSELEKEL- AGTRI
   1311110  --------ES DLKATGNSFV GVGGLGGK-- VDEEPEDEGS LENDLERELA GRA
    722894  --------TQ DAPENGGTSS STVALNDR-- NKNSSPINEN PAEDLDAELV ARDKAGN
    288507  --------NN SASQSMLGAS SGLVQGGT-- ATETASGQLS DAQMMEDPSE AFEREILAK
    757539  --------SL
    184184
    917307  --------RE YGDVPGATSG YTV---SA-- HRPPSPVMDN PSDELDREL- ARTHAGKR
   6355211  --------QE YGPHHGATSS FTA-VQNN-- ARPPSPIMDD PSEELEREL- AGTHLGAGKR DAL
    249241  --------QE YGPHHGATSS FTA-AHTA-- ERPPSPIMDD PSEELEREL- ASTHLGKRG
    216822  --------QE YGPHHGATSS FTA-AATT-- ERPPSPIMDD PSEELEREL- ASTHLAGRR
    805839  --------HE YGPVTGATSG FTA-P-SS-- RKPPSPVMED PSDALEKEL- AGTHIGR
    355172  --------QE YGPHHGATSS FTA-VQNN-- ARPPSPIMDD PSEELEREL- AGTHLGAGKR
    302825  --------QE HGPHHGATSS YTAQSHGG-- ARPPSPIMDD PSEELEREL- ASTHLGITRR
    239059  --------KE FGPHPGHTSN YTT---NA-- PRAPSPIMDD PSAELEREL- ASTHISKK
    251409  --------RE NAPPNGATSG FTE---AQ-- PRADSPVLDD PSAELEREL- AGTHLGKRA
    251443  --------RE NAPPNGATSG FTE---AQ-- PRADSPVLDD PSAELEREL- AGTHLGKRA
     23484  --------AA AKSNEAAMHG DYN------- ------PNRN SSSIAAGAVP TQNDDIRQMD VDA-LERELG GGI
    306334  --------TP GTRGTAHLRG AAA------- ------PDIS QAKQVPKAVE EPTAPQPRPG VDPELERELA GGVF
    306351  --------TP GTRGTAHLRG AAA------- ------PDIS QAKQVPKAVE EPTAPQPRPG VDPELERELA GGVF
    148766
     84676
     74082  --------DP RAYEDPRPHE A--------- --NAAMRDAG LEQELQAGMD RR
     97664  --------DR GGYEDPRPQE AFN------- --QGGWQGSD LERDLQ-GLD GRLR
     97676  --------DR GGYEDPRPQE AFN------- --QGGWQGSD LERDLQ-GLD GRLR
    297954  --------EE RSYQQDQPRP HEA------- --TTHEQGQ- LEQELAGGLD AMMS
    323749  --------QQ WRTEDPRPEE Y--------- --LGHSQG-G LEADLAMGAD GRR
   1926181  --------AG WTSSDPRPQE Y--------- --LGHGQGNG LEADLAGGLD GRR
   1457364  --------QG WSSSDPRPQE Y--------- --GG--QGGG LEADLAGGLD HRR
     78435
   1457361  --------QG WSSSDPRPQE Y--------- ----GGQGGG LEADLAGGLD HRR
   1328540  --------PS ESDLKATGNS FVGVGGLGGK VDEEPEDEGS LENDLERELA GRA
   1364342  --------PS ESDLKATGNS FVGVGGLGGK VDEEPEDEGS LENDLERELA GRA
    350087  --------QP HAGVGPAFGD EDVNTGADA- ---LEHPGSA LERQLDQGRF
    288508  --------IQ AASPSNNSAS QSMLGASSG- ---LVQGGTA TETASGQLSD AQMMEDPSEA FEREILAK
    283473  --------RA GGDAPREGVS ARGCIASACV RELLILGAGA GVGSVRKLFD LVLGWPHGGL FESMYIVISV GRLLLCIE
    734869  --------PS RDDIIASPVR DDAPRDGATS NYTLPAQSQV DPDTDTPIVD NPTEDLEREL DQRLHVSQK
      9394  --------IY LMGTIGISKD ELNYID---- DKWIKTVRRF YNDDKDGGSK LKYVLVDHNE VEKNSENRNI EKKQVIGVID HHAIKSEILK EYRERVERK- ---------- -EMNVLIIEE NGSCSSLIMN YWYEIIFNSK PGERKEELMK
```

```
 167564
 113741
   6575
  30703
  65523
   5078
  54997
 114746
 238776
  71410
 676425
 676527
 152706
  88536
 122893
 151264
  67588
 153824
 103584
 118819  --------NL PLVEADMVPT VEQSKELPND EDKIVMEQEN DARRIRRPSS DIY-RQSMTT PTDPLMNYA- -RRSSVLQTS RQGSTYNSPI PS-SQNLSHL IS-------- SP---ES--I SEFGGSSITF P--S--QNYV VPVRSRTGS-
7227248  --------ES STETAAQKLE TE-------- --KAKEADDV LRDIIPSINS D-HHRRSITT PTDPLVTYA- -RRSSILQPS GQGSTYNSPL AP-THVPAFA DT-------- AV---NA--E KLAQGSVV-F P--T--QGFI IPARSRTGS-
 240796  --------D- SKDSSDWVEN NESE------ --KLKEAEDV LPDNMPSISP ETYHRRSVTT PTDPVVTYA- -RRSSILQPS RQGSTYNSPL PA-SFVPSFA EG-------- AN---NA--D KLGQG-TVAF P--T--QGYV VPARSRTGS-
 366893  --------EP TANISEGMEE SATE------ --KRKEAEDV LPEFMPSISP ETY-RRSVTT PTDPVVTYA- -RRSSIMQPS RQGSTYNSPL PG-SYVPAFV DG-------- ASGLTDS--N KLGQSSTIAF P--T--QGYV VPARSRTGS-
  79368  --------EP SEPTSTTVFD AEVAAEIRDK LTRLKEAEDT LPDTMPSISP DAHHRRSLMT PTDPVVTYA- -RRSSILQPS RQSSTYNSPM PS-SHVPAFV EG-------- AS----T--G DKALGGMVPF P--T--QGFV VPARSRTGS-
 119618  --------EA FSNTIEEAIE LP-------- --KVTHEEVE RTLPPQRKLA DTDSIA--FC AADPVVMYA- -RRSSILQPS RQNSTYNSPV PPPQNLPSYL EN-------- THATQPA--- ---EHGGVVF P--T--QGYV VPARSRTGS-
 500495  --------KT F--------- ---------- ---------- ---------- ---------- ---------- --------PS RRGSTYNSPV PSSQHLPSFV EG-------- YVSAEPSKLE PALAPGSVVF P--S--QGYV VPARSRTGS-
 449465  --------KT FISSPEVMLE RE-------- --GLSRRLEA AKAEIERRPS MSPDLPRHLP TSDPVTSYV- -RRSSILQPS RRGSTYNSPV PSSQQLPSFV EG-------- YVSAEPT--- --LAPGSVVF P--T--QGFV VPARSRTGS-
 509502  --------VT EEPSPVEVVE EK-------- --TALPPVTE EPRTETLTTP LDRRRSQTVS SNDPVVTYS- -RRSSIMQPA RT-SSYTTPF PS-QHTISYS ES-------- AAGQMRS--- ------SVAF P--SRGEGYV VPARSRTQS-
 964561  --------NR FLDASLSRT- ---------- ---------- ---------I TAPETFHATE PTDPVVTYA- -RRSSTIPPG RQST-----S PIASTVQHPP PA-------- TVDGLPT--A ---C-NGIEF P--S-GETYV VPARSRAAS-
 261448  --------ST PLDVSLPRVS SP-------- ----K--QEG DAIDTLVPPT TTSELLRSTT PTDPVVTYA- -RRSSILPPA RQSSTSTYNS PLTSTIQHPL PT-------- MTDGLPT--T ---SGHGVAF P--S--ETYV VPARSRAAS-
1150169  --------NI TGISAPPRVE SP-------- ----EGLTEQ NGVTDTLMPS TAQEFLRPTT PTDPVVTYA- -RRSSILQPT RQTSTSAYNS PLGS-ISHQL SQ-------- SIEGLPT--S NNGSAGGIAF P--T--QSYV VPARSRAPS-
 152363  --------PT EPTAPVAVVD TSL------- --KSTAASII SANKVMVDSP ESQHVQTSKG GTDPVVTYA- -RRSSIMQPS RQGSTYNSPV PSTQYLPTFI EGQSLNLGGD AASILGASGI GAGAGNSIAF PS-TQPPGYV VPARSRTGS-
 379740  --------PT EPTAPVAVVD TSL------- --KSTAASII SANKVMVDSP ESQHVQTSKG GTDPVVTYA- -RRSSIMQPS RQGSTYNSPV PSTQYLPTFI EGQSLNLGGD AASILGASGI GAGAGNSIAF PS-TQPPGYV VPARSRTGS-
 337664  --------PT EPTAPVAVVD TSL------- --KSTAASII SANKVMVDSP ESQHVQTSKG GTDPVVTYA- -RRSSIMQPS RQGSTYNSPV PSTQYLPTFI EGQSLNLGGD AASILGASGI GAGAGNSIAF PS-TQPPGYV VPARSRTGS-
 434295  --------PT EPTAPVAVVD TSL------- --KSTAASII SANKVMVDSP ESQHVQTSKG GTDPVVTYA- -RRSSIMQPS RQGSTYNSPV PSTQYLPTFI EGQSLNLGGD AASILGASGI GAGAGNSIAF PS-TQPPGYV VPARSRTGS-
 517048  --------LA TPEIPQSVLE GL-------- --EPPTKPDE RLAEEGTPST TRPIPRTRQL -VDPVVAYS- -RRSSTFAPS RANSTYGGSA STSYR----P TP-------- SRRTSTSGGE GQGAASTVAF P---IQEDYV VPARSRTAS-
 731201  --------LA TPEIPQSVLE GL-------- --EPPTKPDE RLAEEGTPST TRPIPRTRQL -VDPVVAYS- -RRSSTFAPS RANSTYGGSA STSYR----P TP-------- SRRTSTSGGE GQGAASTVAF P---IQEDYV VPARSRTAS-
 768698  --------LA TPEIPQSVLE GL-------- --EPPTKPDE RLAEEGTPST TRPIPRTRQL -VDPVVAYS- -RRSSTFAPS RANSTYGGSA STSYR----P TP-------- SRRTSTSGGE GQGAASTVAF P---IQEDYV VPARSRTAS-
 517043  --------LA TPEIPQSVLE GL-------- --EPPTKPDE RLAEEGTPST TRPIPRTRQL -VDPVVAYS- -RRSSTFAPS RANSTYGGSA STSYR----P TP-------- SRRTSTSGGE GQGAASTVAF P---IQEDYV VPARSRTAS-
 740533  --------LA TPEIPQSVLE GL-------- --EPPTKPDE RLAEEGTPST TRPIPRTRQL -VDPVVAYS- -RRSSTFAPS RANSTYGGSA STSYR----P TP-------- SRRTSTSGGE GQGAASTVAF P---IQEDYV VPARSRTAS-
 178741  --------SE DPSTSTATTV EP-------- --SETAVPPI AAPSFSAPL- ERSSERPGQS -ADPVVTYH- -RRSSFMHPS RQSSIYSTYA GRTRHSAAYP TE-------- SRNSLERGPA ESGEESSIAF P----TQEYV VPARSRTAS-
  54225  --------SE DPSTSTATTV EP-------- --SETAVPPI AAPSFSAPL- ERSSERPGQS -ADPVVTYH- -RRSSFMHPS RQSSIYSTYA GRTRHSAAYP TE-------- SRNSLERGPA ESGEESSIAF P----TQEYV VPARSRTAS-
 163441  ---------V SPEASSTAAP EP-------- --LGTAMPPS ATPSASAPLP DRPLERERSG QADPIATYV- -RRSSIMQPS RQSSLYSASA GRTRHSIAYP P--------- SRSSLERALD GAGE-SSIAF PG-GTTQEYV VPARSRTGS-
 327966  ---------- SPSSIGLATV ---------- --TETEAPST EFPSTTRP-- DPSSER---- -GDSVAPYP- -RRPSFMRPS RTNSMYSTYA ARNRHSIAYQ PE-------- SRNSLERGPD G---ESSVAF P----TQEYV VPARSRTAS-
 722194  ---------- RPLSFDERLA EQ-------- --SQEETVPA SAADEST-LA DLPAGRI--- -TDPVVRYS- -RRASTFIPS RTHSLFAGHT GTNIWQQRTH TP-------- SSNEQP---- ---ESSSVAF P----TQDYV IPGRSRTSS-
 128404  --------PS ISEDPEVSVQ EA-------- ---------- ----EDSTVS EATIPPGAVT PTDPVLAYS- -RRSSFFGPR RRDSYASSPY VGRQPLPPSR EEPA------ VTQESDTMAS GTTGSNTVAF P----SENYV VPARSRTISQ
 167758  --------SR SPEDRFARRP TA-------- ----SRKASI EPGTLPPSIP EGDAFPFPGS THDPVVAYS- -RRSIFGQPS RTNSMYSIHR ASPAS----- ---------- ---------D APQGSNTVAF PS-TGTGEYV VPARHRTASM
  26908  --------SR SPEDRFARRP TA-------- ----SRKASI EPGTLPPSIP EGDAFPFPGS THDPVVAYS- -RRSIFGQPS RTNSMYSIHR ASPAS----- ---------- ---------D APQGSNTVAF PS-TGTGEYV VPARHRTASM
 987811  --------LA HPPPPASILQ PE-------- ---KEDAKEP VARPRHAPSP SSIGPDHATT PVDPLVSYA- -RRSMIMGHS RQSSAFPSPV MHAHPGPGVP PSSY------ MDGAMQMHAE PGASTSVIAF PTRARTDSYV IPARSRTAS-
 913595  --------LA HPPPPASILQ PE-------- ---KEDAKEP VARPRHAPSP SSIGPDHATT PVDPLVSYA- -RRSMIMGHS RQSSAFPSPV MHAHPGPGVP PSSY------ MDGAMQMHAE PGASTSVIAF PTRARTDSYV IPARSRTAS-
 922124  --------LA HPPPPASILQ PE-------- ---KEDAKEP VARPRHAPSP SSIGPDHATT PVDPLVSYA- -RRSMIMGHS RQSSAFPSPV MHAHPGPGVP PSSY------ MDGAMQMHAE PGASTSVIAF PTRARTDSYV IPARSRTAS-
 319286  --------AL VPNSDSSPSP ARR------- --ALGRTVRH PPAMAISPRR DTNEPNHARG YSQPPSSTG- -TRTGRRRSS VGLTDPFTRR PVSMFGLGLG MSGMTALDGS IGIPAAGGPA GEGSGISASL PRRHGTHASI SGEIEHRGSK
 332677  --------HV LARERSPGTP LSV------- --FSDDSSRP STAGPGPPTS GTISGRSSRR TST--HSTN- -HSAHRRPNS FLFSSGLGAA MTPLSSIGPQ AMSTSTSASS AAAGQGVGGG MHSRAASMSG PGGGGEHPQR PGR-RSRAPS
  75030  --------EY DEPEPISAIP LH-------- ---------- TASTTFSEKD FAPALSSTDS VMDPIANYA- -RRSSLMQPP AQQFAFPTAA GTEP------ ---------- ---------- ---------- ------EVFV VPSRSRMGS-
 740342  --------EY DEPEPISAIP LH-------- ---------- TASTTFSEKD FAPALSSTDS VMDPIANYA- -RRSSLMQPP AQQFAFPTAA GTEP------ ---------- ---------- ---------- ------EVFV VPSRSRMGS-
 769030  --------EY DEPEPISAIP LH-------- ---------- TASTTFSEKD FAPALSSTDS VMDPIANYA- -RRSSLMQPP AQQFAFPTAA GTEP------ ---------- ---------- ---------- ------EVFV VPSRSRMGS-
 493654  --------EG GVPFPTTQ-G ---------- ----LPPEPW PTFYRERARA VSDLPPNSAK AVDPYTVYA- -RRSSIIGFS NLSPDATRSP PGQARPVTRS P--------- STQTVPEGAE GAEEAERSA- -------SYF IPARHRTIS-
  12113  --------EG GVPFPSTQ-G GA-------- ---GIPPEPS PTFYRERARA VSDLPPNSAK AVDPYTVYA- -RRSSIIGFS NLSPDSTL-P PG-ARPVTRS P--------- STQTVPEAAE PAEEGDKSA- -------SYF IPARHRTIS-
 115011  --------EG -VPFPTQT-D ---------- ----FPPEPS PVAYRERLRA VSDLPPRSAK AVDPYTVYA- -RRSSIIGFS NLSPDAARSP TG-GRPPLRS A--------- SSALVEDIAE EVEGGEKSA- -------SYF IPARHRSIS-
 192400  --------EG GVPFPTTQ-G ---------- ----LPPEPW PTFYRERARA VSDLPPNSAK AVDPYTVYA- -RRSSIIGFS NLSPDATRSP PGQARPVTRS P--------- STQTVPEGAE GAEEAERSA- -------SYF IPARHRTIS-
 543665  --------AD DFKQKAGHVT QA-------- ---DAPPSEM PLEFKMRTRS GRGAGGLLGP G
  91520  --------KA TEPFPSREEM TH-------- ------ARER TISPETTTFS CFGERPVSVA PTDPVVTYS- -RRTSIILPS RQNSMSSFQS PFTNHRP--- ---------- ---LSSVDGL PPPSTNAIMF P--TQTEGYV VPARSRASS-
 127212  --------KA TEPFPSREEM TH-------- ------ARER TISPETTTFS CFGERPVSVA PTDPVVTYS- -RRTSIILPS RQNSMSSFQS PFTNHRP--- ---------- ---LSSVDGL PPPSTNAIMF P--TQTEGYV VPARSRASS-
 438879  --------EE EPPSDDAVLE KE-------- ------KDSV KETEALVPRG AHHRGPSATT PTDPLVSYAG ARRSSILQ-- -QQQNSA--- HNSPVVP-AH PLSK-----S FTEGPLTQEP SSLPQGGIAF P----SQGYV FPARSRTGS-
 174304  --------EE EPPSDDAVLE KE-------- ------KDSV KETEALVPRG AHHRGPSATT PTDPLVSYAG ARRSSILQ-- -QQQNSA--- HNSPVVP-AH PLSK-----S FTEGPLTQEP SSLPQGGIAF P----SQGYV FPARSRTGS-
 174289  --------EE EPPSDDAVLE KE-------- ------KDSV KETEALVPRG AHHRGPSATT PTDPLVSYAG ARRSSILQ-- -QQQNSA--- HNSPVVP-AH PLSK-----S FTEGPLTQEP SSLPQGGIAF P----SQGYV FPARSRTGS-
 244084  --------SA PPRVESPEGL TE-------- ------QNGV --TDTLMPST AQEF-LRPTT PTDPVVTYA- -RRSSILQPT RQTSTSA--- YNSPLGSISH QLSQ-----S IEGLPTSNNG SA---GGIAF P----TQSYV VPARSRAPS-
 123741  --------QI SPTVPQPNTR EY-------- ------FPQV --TEGSVSPE IGRDGPAATT -TDPVVTYS- -RRSSLLQPR RNSSFASPYV RNQPLPSFAE DSTK------ -TAEPGTMPP GSVGTNTVAF P----SESYI VPARSRTLS-
 172164  --------QI SPTVPQPNTR EY-------- ------FPQV --TEGSVSPE IGRDGPAATT -TDPVVTYS- -RRSSLLQPR RNSSFASPYV RNQPLPSFAE DSTK------ -TAEPGTMPP GSVGTNTVAF P----SESYI VPARSRTLS-
  40528
  39781
1155690
 247448
 269975
 246067
  44396  --------DT RLRTSNSSAG AS-------- ---PVPTPSS LATSANETSP TYETDLTSAS APHSVTGSNS AKRNGIRAMD GLGLLEQDQH HNKENSSHRH LP-------- -LGMTEEDLI DEKDKRSNAI ------QERR ALDETLANHG
 111248  --------QE RSRTPLQETS TKAKQSVDAS DSYIEVLDDD GSSVLSSLFD EQGMSSETSI STPESLNAVE FLLGGLLYDE ILQYLWPQLF LRTSSVEQAS QLITRFLLRY SLDLQTLAQN FGSARTPDKF ADLKLRAGQF VEKRRR----
  54538  --------GN QAHEKGTEWS LILKVG---S AFWQRLCTEH GISEDGILKD ASIDGGDRKS AFFYQADDER YIPRAILVDL EPRVINNVLS SAYANLYNPE NIYKWKDGGG AGNNWAQGYS YGGEIYEEVM DMVDRETEGC DSLEAF----
1341438  --------RR RGREAPLEWA AGG------G TWTPRAPLDA FGGASPASSD SRHHDGERPQ AVDVSRPARS PAPESDAEGE DKENIAPDCE L-VDGPRNAE DEDGAGHLQH ADDERVAAPS RGVKRKREEC TVPYPKARRE YTLAFP----
1267921  --------RR RGREAPLEWA AGG------G TWTPRAPLDA FGGASPASSD SRHHDGERPQ AVDVSRPARS PAPESDAEGE DKENIAPDCE L-VDGPRNAE DEDGAGHLQH ADDERVAAPS RGVKRKREEC TVPYPKARRE YTLAFP----
 692594  ---------- -SYFTLRHEV TPDRPATSLF GISCTRQMDA NELINRPADV TRSTVQKAVV VISDSPHHFG QLREKLLVVT TAWFAQR-DF TDLDILQKFQ ESLAK-SFRD QDDDRDQYFG LSLRELIHEF KYQTLVLFKC CLLQPK----
 384872  GEEATAPTAH TNGAATAEKS RKKSKKAHTP EPEVEDLVAE ASADGAESEV EEDEEQLNRE LNEDEESEEE QASGANGEAL GTS--DLISG TSMPTMENPT LFSDL-KLSD RTMEAIKTMG FDTMTEIQQK TIPILLSGKD VLGAAKTGSG
 419622  ---------- -NGTAAAEKP RKKAKKEHTP EPEVEAAEDD VESADEEVEE QVDDEEEVEE VEEDEEQLNT ELKQIVAKAT GANSEELPSG TSIPTVEDPQ LFSEL-NLSD RTMEAIKAMG FESMTEIQRK TIPPLLSGKD VLGAAKTGSG
 328081  --------SE DGGLGILRGV ADHPHLPSLT ALPATELRES TYNMSIASDN ASADFKDYPH HTSGPRSRLA AYKLYDTLNP IVSAGFDSAY TSLGKDASTS LRCTLAELKN PNMGGLNQET IASICLGTAE TSQDIIAALQ RSNELAYDSE
  61803
  40664
 105104
 147180
 148245
 146852
  33818
 116469
 503885
 503914
6817382
 145980
 328170
 691008
  73316
1219281  --------DI AALGRKDSVG IPDHQWVQQQ RNDAPRPHDA PRPHDQQFNE ANLEQELAGG LDVMHQ
 422525  --------NR QL
 212435
 187307  ---------G
 146240
 156221  ---------R V--------- --EGQL-EIH RQEFVIVFLS Q--AFNPSNV FSFQW
  48865  ---------R R--------- --HHQLSDAR RRSLQWCYLW V--HCSRSEG A
 226510
 821431
  12784  --------VR R--------- --ETLPVEVP EPTQPAARAP T--PFEEDPS SELEKELAGT RI
 157692  --------MS IHMCR
  47352  ---------- ---------- ----QKQREG RLATPRLIIP V
 136580  --------AD FLKGLIYEEI MR
 422908  --------SI KLSSMKRS
  71188
2049832  --------SA ERYLCDVGFE QDWASVVGT- AINDQSNMNS FSKRQSHCDA LAWCQRYTCL --FALIMLYG IPLGPIAVSS KNFSWLVVII FSIQPENDRE TGRRRKKDGV RITAFSGNGF PFS
2023270  --------SA ERYLCDVGFE QDWASVVGT- AINDQSNMNS FSKRQSHCDA LAWCQRYTCL --FALIMLYG IPLGPIAVSS KNFSWLVVII FSIQPENDRE TGRRRKKDGV RITAFSGNGF PFS
  62157  --------DC TLDKPKYHPP KPNATAMDY- YPSADSSPLL AANAADLIET PTCSSNSTPR --PSVHTPRT PVIDPSAEPG -SWTRNLPIM KTLFDSNKVT DGFELEKKQH QSAASNADSS PTRRTSDEKT PWYQTSQRWL L
 117327  --------NI RLRLCSITGT NPVPHSDRL- VPSPQKYRRD RCDSTSIPPT PALTSDPHSS --SSVHRNLS STSSSHATSS -HSRLFPRLL PSVYPGNNGN IPIGVDNRLC RRGRCSTI
1420954  ---------- YLWPPGVDNV TD-TSLKEH- EENTETHCKI WNRYTTVRQT WILGGKARQG --ASFFNEHG
 146600
 427542
 524886  --------DD VDRTTEVYQM IEVEQVFELR AQLAFV
 304061  --------YT LQIVDSVKLR FTGGRIAGFG TVTAIVHFCV PAKHGNELDA
 262011
 385460
 322662
1531698  --------ST AGA
1273298
 211888  --------KA EPDMMDGVGL AHTHDA
1140823
  84032
 337569
 675946
```

```
 188798
 364357
 417259
 391850  --------GY E
 420539  --------RG HC
1649896
 280694
 381396  --------PS
 685453
 284413
 384436
 907966
 205730  --------YQ DQIVQQQQIY VQQAQQPAQA APAQQGYPAQ PVPPAGYPPA AAYAGEQPNQ --A
  86646  --------PQ VQNPALPFNG QGQI
   9721  --------LP RPPPVVINEH HHNNQMMA
   2612  --------TG
 102501  --------YN
  98652  --------Y
  78159  --------H
 240194
 193495  --------S
 171744  --------S
   4564
   6035  --------VN GA
 256278
 120199  --------AP IV
1003994  --------AP IT
 145010  --------PP MA
 183924  --------PP IS
 725911  --------PP IV
1166877  --------PP LV
 574353  --------PP IS
 277341  --------AP IT
 965410  --------AP IT
1040134  --------AP IT
 801239  --------AP IT
 457049  --------AP IT
 777636  --------AP IV
 167531  --------AP MT
 179443  --------PP MV
 388203  --------IA MLPGSEPLAA LQLVFFLSLR PSLGPNNHIS FCSYIHMYGK ISKINLFAPS HLYFLCRSNA FPSGCLGDPS CSLTSLCVFL PAVDPMEKEG M
 761034  --------PP MVVSSLSHGS LDVYNTITSH FGLF-SLHLA GARVAHLPYR -RRVDLPASS ---LLCCASV
 117742  --------TI
 506090  --------TI GTI
  21911  --------TI GTL
7342346  --------AP IT
 435735  --------LP IT
 267232  --------AP IT
 679497  --------AP IT
 457636  --------AP IT
1340042  --------DW LA
 225932  --------PC LTTLRATH
 346924  --------AQ
 760343  --------LA
  15147  --------PH STSQAMEPME M
 108680
 724148
 276168
 194959  --------SI
 345309  --------SI
7189608  --------AI
 439783  --------SI
  73775  --------SI
 681486  --------SI
 671673  --------SI
 829815  --------TI
 413627  --------SI
 109090  --------GL
  43366  --------AI
 706326  --------CI
1157202  --------CI
 743174  --------AI
   5331  --------CI
 713967
 151043  --------LI
 481570  --------LI
 104738  --------LI
 689474  --------QS RTERWAATAS PKYADLTVSS RAPI
 179470  --------AI
  31001  --------AI
 155293  --------TL GQ
  83606
  79026  ---------- --VLIYEEVT KPPLQSSPYL APAYS
  79053  ---------- --VLIYEEVT KPPLQSSPYL APAYS
 188294  ---------- --VLIYEEVT KPYPQDPLLT SNEYS
 422236  ---------- --VLIYHEIT RPVENELGSS A
  59295  ---------- --ALIYEEIT RPRGE
  91727  ---------- -IVLIYEEVT RPRPQPVTQ
  92869  ---------- --VLIYEEVT KPRLQPAQST Q
 513929  ---------- --VLIYEEVT KPRPPPGTQ
 166192  ---------- --VLIYEEVT KPRLQPAQST Q
 210022  ---------- --VLIYHEVT SPH
 847083  ---------- --ELIYEEIT RPYVQPS
 819022  ---------- --VLIYQEIT KPDPSQATSY LLSGQGVGAA GMNGS
  50473  ---------- --VLIYQEIT KPDPSQATSY LLSGQGVGAA GMNGS
 850567  --------PR STELIYQEVT S-RPPPASSF LTIGDGSP
 183076  ---------- --ELIYNEVT STRTQPPSLF LSGADAAP
1114014  ---------- --VLIYKEIT TPRQSPFLLS STTGGG
  47661  ---------- --LLACHSGS ACTDFRLSTS AHI
6536531  --------NN RAPLLLARYT
 277624  --------DD RAPLLLAKYT
  77457  --------SS HHTFLQCVYM KRLHGQRMII EHHYFFRNTH NVIPHVFLLV VI
  89431  --------CP TVVLIYEEIT RSDDGRPPLA LAEYR
 484632  ---------- --VLIYEEIT RSDDGRPPLP LAEYR
 161998  ---------- --ELIYEEVM RPM
 324877  ---------- --VLIYKEIT HS
 117759
 489827  ---------- --VLIYEEIM RDPL
 115935  ---------- --VLIYEEIM RDSM
 482781  ---------- --GMQYLPPQ VNAHGSNPAI SSHIRGDHA
 545919  ---------- --VLIYEEIM RDPL
6536562  ---------- -------EII KPDNNRAPLL LARYT
 309997  ---------- --ELIYKEIM GAPNVNGR
 316873  ---------- --ARGICLVY GSGVYGIAL
 429267
 103465  --------H
 167039
   8637
  65963
 246409
 246352
 668220  --------PP M
 619500  --------AP IT
  64492  --------PP IS
  80250
  21917
 600175  --------AR
 721307  --------SQ ATSYLLSGQG VGAAGMNGS
 847084  --------QP S
 280696
 908144
 148879  --------ES IYPDVL
 102030  --------YP QSDSVEDSEV DDEDM
```

```
  92334  --------LS QHLGAG
 146634  --------DA PRFIAEQKAA NPDAAFPLQA PA
  72485  --------FK MAQMKLQAQK LQSTKSPASA RITPKEGPIE RDDHDEDINA PESLDAELRR AGIAVGDQ
 124061  --------I
 109792  --------SF KPLA
 408982  --------SF RPLPIT
 149279  --------SF RPQPIT
  13526  --------NR APLLLSKYS
 465497  --------GR PPLPLAEYR
 216389  --------GC IPLLLAGYS
  17191  --------I
 530963  --------GD -TDGLDLGAM TDGLAVQT
  84099  --------DQ GNEATAAGGL TENGAA
 928715  --------TW PIPSA
 480104  --------SI
  37144  --------SI
 993637  --------SI
 706327  --------CI
   5366  --------CI
 144998  --------PP MA
 232199  --------PP IV
  60405  --------AP IT
 572635  --------AP IT
  37912  --------AP IV
 819552  --------AP IT
1026351
 352542  --------NE LGSSA
  42114  --------GC IPLLLAGYS
 204011
  46962
   3452
1531701
 375250  --------PL LPSPTIKRLL SQLQIDCIMR LTHPRPKLAP
 391393  --------WA TGQS
 518679
1272884  --------WR HR
 108249
 111885  --------DE TTGPF
 228039
 117728  --------HL QFLKIHQLIR CQLPKLSLQL FAIKHKFSTL PFPFIQNQDK AG
 267276  --------SI CIKMTSAANS
 295270
 174214  --------AA EPAAENGTAI P
 111562  --------AA GGLTENGAA
 385026  --------AN GDAVENGGVG QI
  47937  --------VG VPQ
 191658  --------AA VPEQENNGS
 361397  --------AA AQEQENGS
 368356  --------AA TQEQENNGS
 422287  --------AA AQEEQNDS
1051981  --------DE PMNGQGEAQ
 512786  --------VD PEQYNGQ
 700253  --------EQ EQQFNGQ
 171641  --------DD --QFNGQ
 447383  --------ES QLNGH
 134451  --------ME EIT-NGQ
 525556  --------QV LVEGVGDSQQ AFAA
1776212
 443108
 129540  --------AL PLESQALP
 341724  --------AL SLDNTLGTVP R
 138417  --------AL PLESQALP
 158191  --------TW TPRAPMSTPT VPASLNAF
 376076  --------RI VSWRFETVTG LGYALNSQLR GGRWYKKHRN T
 368607  --------LP EDGASGF
1312592  --------T- --PEGAEAAA RAVGAAPAEV STTA
  46955  --------G- -VPEGALAGP EDPSHAIAAG VVQAE
1164008  --------G- NVPEGALAAP EGDVVQAPAV GAEA
 600771  --------G- -VPEGALAAP EDTATASTAV AATAE
 161654  --------GD NIPEGSLAGP DKDGEIVGSG PSSVEEAAVP TIQPSAQAGA
 149140  --------GD NIPEGSLAGP DKDGEIVGSG PSSVEEAAVP TIQPSAQAGA
 474692  --------LG -IPEGALAGP EDGGYSNGHA
 134758  --------SG -IPEGPISGP EDGAGGPGQP VAAAAS
 177791  --------TE -VPEGALSAP ENHL--SNGI PLVTN
 132015  --------SG -IPGGALAGP ENGSLQTNGI VLAA
 917530  --------SG TVPEGALAGP DAQGVPVE
 428496  ---------- ---EGALAGP DTTA
1276586  --------VD PQTFGVPASA VAAT
 115601  --------LG AMTDGLSVTA
 171809  --------PR AISGTTRSDS KLDRTFDHLS PPVT
 571775  --------IL SITEDFHQVG EASITASGIP GGALAGPDNG TLQANGIVLT A
 722302  --------EG PLAAPVV
 289694  --------AG PVPPVSS
 137978  --------FL GTPLTGPTLE N
  19116  --------PI TLFCVN
 129801  ---------- ---QIQAQSE QLQYQQMQQP AQ
  80382  --------ND NSVQGQYESD IIQQQHLQQR LEQQQQQQQQ QQQQGQERVG EKSS
 141916  --------ND NSVQGQYESD IIQQQHLQQR LEQQQQQQQQ QQQQGQERVG EKSS
 187748  --------ND LTSNADGSTN PQQQEQLAQI QQEGIQAPIN KHEQQI
 168984
  71808  --------PH MSAATNDTSV SESTSKAVAD DNVAAQPL-N
    432  --------IP EENENFDVQE AQFR
  59395  --------MN GGSDTNGASV DLLPS
   2865  --------SG TENNGN
 189766  --------QN DS
  88183
 216492
 188879  --------DS YNQGLIQGQE ILQGA
 182850  --------DS YNQGLIQGQE ILQGA
 174697  --------DS YNQGLIQGQE ILQGA
 934899  --------GG VPEGALAGPE VPNATAASA
 362397  --------A- --EGPITG-E LPPAPAS
 416973  --------TG IPEGALAGPE NGQLSNGAPV TI
 551386  --------LG AMTDGLAVQT
  15239  --------LG AMTDGLSVTA
 497504
1117682
 252496  --------ID
 148867  --------TE NGGSD
 423027  --------EP FLPPDPSLSP EVLDPLGQIQ TQSMVTKSAD TLDQDILQY- LQI
1104996
  29266  --------SA ESQLNGH
 171653  --------VM DDQFNGQ
 117063  --------VM QEFTNGQ
 158104  --------VD ANGAS
 160940  --------DA ENGS
   2813  --------GS GTENNGN
 129541  --------AL PLESQALP
 257371
 389086  --------A
  73401  --------TL AEEPMSGADL LPNFSTFPVP GRETDTNPDS TFSLLEPWST WDIGG-LDLS ---LPESDYV DPAGPSLPTT IG
1139913  --------TL AEEPRSGADL LPNFSTIPVL GRETDTNPDS TFSLLEPWST WDIGD-LDLS ---IPATDYV DPAGPSLSTT IGLKATFWFV YCIGVVHISQ LSKKRTSSRL RVQTDSDHTW P-RRRDVYVL NWEQGALTCI PPRTDIAPGP
1168371  --------TL AEEPRSGADL LPNFSTIPVL GRETDTNPDS TFSLLEPWST WDIGD-LDLS ---IPATDYV DPAGPSLSTT IGLKATFWFV YCIGVVHISQ LSKKRTSSRL RVQTDSDHTW P-RRRDVYVL NWEQGALTCI PPRTDIAPGP
 698519  --------RR ISPPSYPC
 332332  --------AL SLDNTLGTVP RVFDNALGWG NSTVRKMSVG IWELLDFCSM LGPGVMLNSN ---IMVVGYC DKGKA
 243292  --------AA ENGTAIP
 243775  --------AA ENGTAIP
  24163  --------AA EQNASVP
1431451
  99398  --------TR IIR
 171665  --------DD QFNGQ
  15876  --------A
  43014
```

```
    43498
  1148336
   220391
   774567
   138915
   757677                                                                                                                                MPRHTFT ALNS-TFVVD SEYEFVKELG QGAYGCVVA-
   313819                                                                                                                           M TLYSKRHSFN ALNS-TFVVD AEYEFVKELG QGAYGCVVA-
   290180                                                                                                                            MQLQRHSFT ALNS-TFVVD SEYQFVKELG QGAYGCVVA-
    12232                                                                                                                                                   M SRSAGSWYES WVRGRTALW-
   865310                                                                                                                           M GDLQGRKVFK VFNQ-DFIVD ERYTVTKELG QGAYGIVCA-
   312015                                                                                                                                                                MDVSS-
   500478                                                                                                                                                     MEINTG NGFWGIRSRY
   500460                                                 MSRPGSSRR SSRGHRRRES CADGKGEEPK SSSPVPSPGS --------WN VDRGRSRVRR PSNPFSEDNL RRRGYHSLLS SFGK-VFHVE RRWKLIREMG SGAYGVVIS-
   243933                                                                MREHL SQTSSSSDEE SSSPVASPVS --------AT VDRGRSRIRR PSNQLAEHNL RKRGYHSFMS SFGK-MFHVE KRWKLVREMG SGAYGVVVS-
    64065                                                                                                                        MISVHNF TPLNTVFIFK KEREVTFNVS DYYEIKDIIG EGAYGIVCS-
     2938
   196733
   145976                                                                                                                                                   M ENYQKLEKIG EGTYGVVYKA
   501440                                      MPAPRQLP PPSRPRPPPP ANGGMPRQTS SSSTLVSDSS QLQIAAVPDD PKRLSTRLQG VPAQLSTPDS NQPSINQPSN SHYRPKYSSY LTCNTRFDLP PRYRLIHPLG SGSFGLVCAA
    71512                                      MPAPRQLP PPSRPRPPPP ANGGMPRQTS SSSTLVSDSS QLQIAAI--- ---------- ---------- ---------- ---------- ---------- ---------- ----------
   543038
    99402
  1431403
   342880
   417966
Consensus          .. .......... .......... .......... .......... .......... .........   ......... .......... ......   .             ......... .......... .     .... .........

           1501                                                                                                                                                            1650
     6423
   160078
   116650
   377992
   289793
   670269
    32808
   156295
    22314
   417978
   414783
  1074946
   865350
  1656563
   333276  QMWLREAVRR LDMDGQ
   422586
   342887
   386771
    14976
   130981
   435286
  1457372
   249807
  1142566
  1008492
   152510
   336937
  1649893
   371270
    82103
    42859
   212507
   212502
   461664
    39613
    91135  NYQNNNHNNS NTNANNNNAG NAGYNNDNNT NNNHNVNSFD NLEKELEFGL DRYC
   103403
   112360
     4266
   168486
    35227
    67581
    59367
   336175
   177826
   212434
   164796
   100890
   288633
    69432
    60279
   166223
  1111368
  7180514
   671172
    69595
   188428
   799680
   178052
   129301
   208817
   424444
   830405
   892596
   186189
   164780
   167312
   119588
   423592
   474667
   459613
   156206
   791811
   146905  ---RQARKSW IF--GCFTLS SWPPPWFDVD GPRFRIWKEA SIRWLGRR-T LIESS----- -----TSHLR AFSVFVRVRA QKLCDPRVWA IMKFARLRRA RR---YHAAG WPRVWPRMDK RVNRHVRELF VDA
   715329  ---VCQVRPY DIADGSLHTG NVIAYVFFIL APSLSLPSPA EFRFMTTRGS VVRFI----- -----DPPLT PSAVKWRTNS RFAYDMPPTR SSKSVGDQRD HSRLLIRTMG SPLTAAGTAS AALTRSRKET RDAKDAFMLW DAMIITNASL
   511921  ---PILRRP- ------TRSG SWGRPM--PP RPLPASVAEA HHAAHALEQL LAEQR----- -----LDLFQ HAGVQDIRYP AVAKPPKNEH QLLQIALSRC KW---IQASD SLGVCAGMWD SAPNRVLKLI SSEAVARSIK DMKVVEQACS
   737951  ---AKTIKV- ------RVSG QEGPSVRNTK RESRSRVGGK
   545584
   102765
   203555
   302829
   701651
   972433
   931864
   813292
    78187
   406308
   118382  FVWKSFVPLW RLLAQNVHAA IVSHCTGTPK DQRSRLPAEA RTAIKKVRAT QRMRR----- -----NCGFA FSPTESAGKQ DQAKIIRAYD TSKSNARPSG Y
   100328
  1411022
   741167
  1338511
   756718
   302653
  1311110
   722894
   288507
   757539
   184184
   917307
  6355211
   249241
   216822
   805839
   355172
```

```
     53464
    105414
    291876
     50744
    863837
     81355
    152896
   1196002
   1195972
     49917
    116823                                          ML TP---PATS- ------SPSF SAPISTACS- --------HL ALSRNSSS-- STSSIRFW-Y VAKRGPADQS ANNSKRG--- ---------- ------LKYV HSAGVVHRDL
    271167                                                   MSE- ------AFRT SWWFTDNAC- --------SY FVTELLGT-- DLHRLLTS-R PLEKQFIQYF LYQILRG--- ---------- ------LKYV HSAGVVHRDL
    151286 QLTGQ----- PVAVKKIMKP FSTPVLSKRT YREL-KLLKH IR---HENI- ------ISLN DIFISPLED- --------IY FVTELLGT-- DLHRLLTM-R PLEKQFIQYF LYQILRG--- ---------- ------LKY- ----------
     97820                   MKP FSTPVLAKRT YREL-KLLKH LR---HENI- ------ISLS DIFISPLED- --------IY FVTELLGT-- DLHRLLTS-R PLEKQFIQYF LYQIMRG--- ---------- ------LKYV HSAGVVHRDL
    167647                   MKP FSTPVLAKRT YREL-KLLKH LK---HENV- ------ISLS DIFISPLED- --------IY FVTELLGT-- DLHRLLTS-R PLEKQFIQYF LYQIMRG--- ---------- ------LKYV HSAGVVHRDL
    430394                   MKP FSTPVLAKRT YREL-KLLKH LK---HENV- ------ISLS DIFISPLED- --------IY FVTELLGT-- DLHRLLTS-R PLEKQFIQYF LYQIMRG--- ---------- ------LKYV HSAGVVHRDL
    266211                   MKP FSTPVLAKRT YREL-KLLKH LK---HENV- ------ISLS DIFISPLED- --------IY FVTELLGT-- DLHRLLTS-R PLEKQFIQYF LYQIMRG--- ---------- ------LKYV HSAGVVHRDL
    266223 QLTNQ----- NVAIKKIMKP FSTPVLAKRT YREL-KLLKH LK---HENV- ------ISLS DIFISPLED- --------IY FVTELLGT-- DLHRLLTS-R PLEKQFIQYF LYQIMRG--- ---------- ------LKYV HSAGVVHRDL
    266189                   MKP FSTPVLAKRT YREL-KLLKH LK---HENV- ------ISLS DIFISPLED- --------IY FVTELLGT-- DLHRLLTS-R PLEKQFIQYF LYQIMRG--- ---------- ------LKYV HSAGVVHRDL
    151328                                                                                                          M-R PLEKQFIQYF LYQILRG--- ---------- ------LKYV HSAGVVHRDL
    800193 EISGE----- TVAIKLVTRV FEKVQLAKRA LREI-TLLRH F---NHENIT -----GLIDV DAISPDFNE- -IY-----IF ME--PMEA-- DLHQIIKSGQ VLTNEHVQYF VYQILRG--- ---------- ------MKYI HSASVIHRDL
     22041 EISGE----- TVAIKLVTRV FEKVQLAKRA LREI-TLLRH F---NHENIT -----GLIDV DAISPDFNE- -IY-----IF ME--PMEA-- DLHQIIKSGQ VLTNEHVQYF VYQILRG--- ---------- ------MKYI HSASVIHRDL
     22014                              MSM PFRL-TLTRC Q---WH---- ------LVVI SVRYADILF- -SY-----IF ME--PMEA-- DLHQIIKSGQ VLTNEHVQYF VYQILRG--- ---------- ------MKYI HSASVIHRDL
     22020                                                                                                                                  MGTQG--- ---------- ------MKYI HSASVIHRDL
    448385 DISGE----- TVAIKLVTRV FEKVQLAKRA LREI-TLLRH FA--NHENIT -----GLIDV DAVSPDFNE- -IY-----IF ME--PMEA-- DLHQIIKSGQ TLTNEHVQYF LYQILRG--- ---------- ------MKYV HSASVIHRDL
    122008 EISGE----- TVAIKQVNRV FDKIQLAKRA LREV-TLLRH FA--NHENIT -----GLIDL DAISPDFNE- -IY-----IF ME--PMEA-- DLHQIIKSGQ SLTNEHVQYF LYQVLRG--- ---------- ------MKYV HSASVVHRDL
    578295 EISGE----- TVAIKLVTRV FEKVSLAKRA LREI-TLLRH FA--NHENIT -----GLIDV DAISKDFNE- -IY-----LF ME--PMEA-- DLHQIIKSGQ SLTNEHVQYF VYQILRG--- ---------- ------MKYV HSASVVHRDL
    139503 DISGE----- TVAIKMVTRI FEKTQLAKRA LREL-ALLRH FN--NHENIT -----GLIDV DAIAPNFNE- -MY-----LF ME--PMEA-- DLHQIIKSGQ MLSNEHVQYF TYQILRG--- ---------- ------MKYV HSASVVHRDL
   2468658 EISGE----- TVAIKMVTRV FEKTQLAKRA LREI-TLLRH FA--GHENIT -----GLIDV DAITPDFNE- -LY-----V- ----PMEA-- DLHQIIKSGQ TLTNEHVQYF LYQVLRG--- ---------- ------MKYI HSASVIHRDL
   2215386 EISGE----- TVAIKMVTRV FEKTQLAKRA LREI-TLLRH FA--GHENIT -----GLIDV DAITPDFNE- -LY-----IF ME--PMEA-- DLHQIIKSGQ TLTNEHVQYF LYQVLRG--- ---------- ------MKYI HSASVIHRDL
     88816 EISGE----- TVAIKQVNRV FEKASFSKRC LREI-TLLRH FA--NHENIT -----GLIDV DAISKDFNE- -VY-----LF LE--PMEA-- DLQQIIKSGQ PLTNEHVQYF LYQILRG--- ---------- ------MKYV HSASVVHRDL
     88949 EISGE----- TVAIKQVNRV FEKASFSKRC LREI-TLLRH FA--NHENIT -----GLIDV DAISKDFNE- -VY-----LF LE--PMEA-- DLQQIIKSGQ PLTNEHVQYF LYQILRG--- ---------- ------MKYV HSASVVHRDL
     88917                                                                                             MEA-- DLQQIIKSGQ PLTNEHVQYF LYQILRG--- ---------- ------MKYV HSASVVHRDL
    206606 SISGE----- NVAIKQVTRV FEKKELAKRA LREI-VLLRH FN--HHENIT -----GLIDM DLLSPDFNE- -IY-----LF ME--PMEA-- DLHQIIRSGQ ALTNAHIQYF LYQILRG--- ---------- ------CKYI HSANVVHRDL
    123647 EISGE----- PVAIKLVTRV TQKIQLAKRA LREL-TLLRH FS---HENIT -----GLIDV D-ISTNSDE- -II-----V- -------A-- DLHQIIKSGQ TLTVEHVQYF VYQILRG--- ---------- ------IKYI HSASVVHRDL
    143931 EISGE----- PVAIKLVTRV TQKIQLAKRA LREL-TLLRH FS---HENIT -----GLIDV D-ISTNSDE- -IY-----IF ME--PMEA-- DLHQIIKSGQ TLTVEHVQYF VYQILRG--- ---------- ------IKYI HSASVVHRDL
    165009 EVSGE----- PVAIKMVTRA TEKLQLAKRV LREI-TLLRH FN---HENVT -----GLIDV D-CTSDLSE- -IY-----IF ME--PMEA-- DLHQIIKSGQ ALSNEHVQYF VYQILRG--- ---------- ------MKYV HSASVVHRDL
    580315 EISGE----- PVAIKMVTRA LEKVALAKRV LREI-TLLRH FA---HENIT -----GLIDV D-CTPDMSE- -IY-----IF ME--PMEA-- DLHQIIKSGQ ALTNEHVQYF VYQIFRG--- ---------- ------MKYV HSASVVHRDL
    492840 EISGE----- PVAIKMVTRA LEKVALAKRV LREI-TLLRH FA---HENIT -----GLIDV D-CTPDMSE- -MH-----L- -----SPA-- DLHQIIKSGQ ALTNEHVQYF VYQILRG--- ---------- ------MKYV HSASVVHRDL
    594182 EISGE----- PVAIKMVTRA LEKVALAKRV LREI-TLLRH FA---HENIT -----GLIDV D-CTPDLSE- -IY-----IF ME--PMEA-- DLHQIIKSGQ PLTNEHVQYF VYQVFRG--- ---------- ------MKYI HSASVVHRDL
    168238 DISGE----- PVAIKMVTRA LEKVALAKRV LREI-TLLRH FA---HENIT -----GLIDV D-CSPDLSEM HVY-----IF ME--PMEA-- DLHQIIKSGQ ALTNEHVQYF VYQVLRG--- ---------- ------MKYI HSASVVHRDL
    107302 DISGE----- PVAIKMVTRA LEKVALAKRV LREI-TLLRH FA---HENIT -----GLIDV D-CSPDLSEM HVY-----IF MEVRPSLP-- DLHQIIKSGQ ALTNEHVQYF VYQVLRG--- ---------- ------MKYI HSASVVHRDL
     64718 DISGE----- TVAIKQVSRV FDKVQLAKRA LREI-TLLRH FS--NHENIT -----GMIDL DVISPDADE- -IY-----IF ME--PMEA-- DLHQIIKSGQ ILTNEHVQYF LYQILRG--- ---------- ------MKYI HSASVVHRDL
     64830 DISGE----- TVAIKQVSRV FDKVQLAKRA LREI-TLLRH FS--NHENIT -----GMIDL DVISPDADE- -IY-----IF ME--PMEA-- DLHQIIKSGQ ILTNEHVQYF LYQILRG--- ---------- ------MKYI HSASVVHRDL
   1154823 DISGE----- TVAIKQVSRV FDKVQLAKRA LREI-TLLRH FS--NHENIT -----GMIDL DVISPDADE- -I-------- SW--ECLA-- DLHQIIKSGQ ILTNEHVQYF LYQILRG--- ---------- ------MKYI HSASVVHRDL
    433002 EISGE----- TVAIKLVTRV FEKVQLAKRA LREI-TLLRH FA--NHENIT -----GLIDV DAISPDFQE- -IY-----IF ME--PMEA-- DLHQIIKSGQ TLTNEHVQYF LYQILRG--- ---------- ------MKYI HSASVIHRDL
    822334 EISGE----- TVAIKLVTRV FEKVQLAKRA LREI-TLLRH FA--NHENIT -----GLIDV DAISPDFQE- -IY-----IF ME--PMEA-- DLHQIIKSGQ TLTNEHVQYF LYQILRG--- ---------- ------MKYI HSASVIHRDL
    511572 EISGE----- TVAIKLVTRI FEKVQLAKRA LREV-TLLRH FS--SHENIT -----GLIDV DAISPDFQE- -IY-----IF ME--PMEA-- DLHQIIKSGQ HLSNEHVQYF LYQILRG--- ---------- ------MKYV HSAAVIHRDL
    688421 QISGE----- TVAIKLVTRV FEKLQLAKRA LREI-TLLRH FS--NHENIT -----GLIDM DAIYPNFQE- -IY-----IF ME--PMEA-- DLHQIIKSGQ TLTNEHVQYF LYQVLRG--- ---------- ------MKYI HSASVIHRDL
     17778 QISGE----- TVAIKLVTRV FEKLQLAKRA LREI-TLLRH FS--NHENIT -----GLIDM DAIYPNFQE- -IY-----IF ME--PMEA-- DLHQIIKSGQ TLTNEHVQYF LYQVLRG--- ---------- ------MKYI HSASVIHRDL
   1336823 KISGE----- TVAIKMVTPV LDKLQLAKRA LREI-TLLRH FSTRGHENIT -----GLIDL DAIHPEFTE- -IY-----IF ME--PMEA-- DLHQILRSEQ CLTNEHIQYF LYQILRG--- ---------- ------MKYI HTASVIHRDL
    605623 EISGE----- TVAIKMVTRV FDKVSLAKRA LREL-TLLRH FN--NHENIT -----GLIDV DAIAPGFNEM YVQLSAPSIE RALTSLLA-- DLHQIIKSGQ ALTNEHTQYF VYQILRG--- ---------- ------MKYI HSASVVHRDL
    511468                                                                                        ME--PMEA-- DLHQIIKSGQ HLSNEHVQYF LYQILRG--- ---------- ------MKYV HSAAVIHRDL
     60300 EISGE----- TVAIKLVTRV FDKVQLAKRA LREI-TLLRH FA--GHENIT -----GLIDV DAISPDFNEI YIF------M EVSIPLST-- DLHQIVKSGQ TLTNEHVQYF LYQVLRG--- ---------- ------MKYV HSASVIHRDL
   2435698 EISGE----- TVAIKMVTRV FEKTQLAKRA LREI-TLLRH FA--GHENIT -----GLIDV DAITPDFNEL IL-------- ------AA-- DLHQIIKSGQ TLTNEHVQYF LYQVLRG--- ---------- ------MKYI HSASVIHRDL
   1222436 QISGQ----- TVAIKLVTRI FEKTQLAKRA LREI-TLLRH FT--NHENIT -----GLIDM DAISPDFQEI YIF------M E---PMEA-- DLHQIIKSGQ TLTNEHVQYF LYQILRG--- ---------- ------MKYI HSASVIHRDL
   1172380 EISGQ----- TVAIKLVTRV FEKIQLAKRA LREI-TLLRH FA--NHENIT -----GLIDV DAITPDFHEV YIF------M E---PMEA-- DLHQIIKSGQ HLTNEHVQYF LYQILRG--- ---------- ------MKYI HTASVIHRDL
   1151825 EISGQ----- TVAIKLVTRV FEKIQLAKRA LREI-TLLRH FA--NHENIT -----GLIDV DAITPDFHEV QLI------Y H-----SA-- DLHQIIKSGQ HLTNEHVQYF LYQILRG--- ---------- ------MKYI HTASVIHRDL
    410058 EISGE----- HVAIKQVSRI FTKVELAKRA LREI-TLLRH FA--YHENIT -----GLIGQ PTVALYSNML NYS------Q ----PMEA-- DLHQIIRSGQ VLTSAHVQYF LYQVLRG--- ---------- ------VKFI HSAGVIHRDL
     57461 DISGE----- TVAIKMVTRI FEKTQLAKRA LREL-ALLRH FN--NHENIT -----GLIDV DAIAPNFNEM YLF------M E----VRS-- DLHQIIKSGQ MLSNEHVQYF TYQILRG--- ---------- ------MKYV HSASVVHRDL
    311323 NQTQE----- GVAIKKITNV FSKKILAKRA LREI-KLLQH FR--GHRNIT -----CLYDM DIPRPDN--- FNE-----TY LYEELMEC-- DLAAIIRSGQ PLTDAHFQSF IYQILCG--- ---------- ------LKYI HSANVLHRDL
    143102 TSNDE----- QVAIKKVCRI FEKNILAKRA LREV-KLLKH FN--GHENIT -----SIIDM DIVNLQD--- FNE-----LY LVQELMEA-- DLHQIIRSGQ PLTDAHFQYF VYQICRG--- ---------- ------LKYI HSANVLHRDL
   2750129 RRTGE----- GCAIKKITNI NTKRILTKRC LREI-RLLHH FR--GHKNIT -----CLYDM DIVFQPN-GN FDE-----VY LYEELMEA-- DLHAIIRSGQ PLTDAHFQSF LYQTLCG--- ---------- ------LKYI HSANVLHRDL
   2702932 RRTGE----- GCAIKKITNI NTKRILTKRC LREI-RLLHH FR--GHKNIT -----CLYDM DIVFQPN-GN FDE-----VY LYEELMEA-- DLHAIIRSGQ PLTDAHFQSF LYQTLCG--- ---------- ------LKYI HSANVLHRDL
    108010 VPTQR----- KVAIKRITP- FDHSMFCLRT LREI-KLLRH FH---HEN-- -----IIAIL DILRPPSLAD FTE-----VY LVQELMET-- DLHRVIRT-Q ELSDDHCQYF IYQTLRA--- ---------- ------LKAL HSADVLHRDL
   1754091 VPSQR----- KVAIKRITP- FDHTMFCLRT LREI-KLLRH FH---HEN-- -----IISIL DILRPSSLSE FKE-----VY LVQELMET-- DLHRVIRT-Q QLSDDHCQYF IYQTLRA--- ---------- ------LKAL HSADVLHRDL
    564944 RPSGR----- KVAIKKIAP- FDHSMFCLRT LREL-KLLKF LS---EAGVS ENPAQIISIL DIIKPPSIEQ FKE-----VY LIQELMET-- DMHRVIRT-Q DLSDDHAQYF IYQTLRA--- ---------- ------LKAL HSADVIHRDL
    AtMPK18 THTGE----- KVAIKKINDV FEHISDALRI LREV-KLLRL LR---HPDIV ------EIKS IMLPPSKREF KD------IY VVFELMES-- DLHQVIKAND DLTREHHQFF LYQMLRA--- ---------- ------LKFM HTANVYHRDL
    AtMPK19 TQTGE----- KVAIKKINDV FEHVSDALRI LREV-KLLRL LR---HPDIV ------EIKS IMLPPSKREF KD------IY VVFELMES-- DLHQVIKAND DLTREHHQFF LYQMLRA--- ---------- ------LKYM HTANVYHRDL
    AtMPK20 TLTGE----- KVAIKKIHDI FEHISDAARI LREI-KLLRL LR---HPDIV ------EIKH IMLPPSRREF KD------IY VVFELMES-- DLHQVIKAND DLTREHYQFF LYQLLRA--- ---------- ------LKYI HTANVYHRDL
  OsMPK20-1 VHTGE----- KVAIKKIHDI FEHISDAARI LREI-KLLRL LR---HPDIV ------EIKH IMLPPSRRDF KD------IY VVFELMES-- DLHQVIKAND DLTKEHYQFF LYQLLRA--- ---------- ------LKYI HTANVYHRDL
  OsMPK20-4 QHTGD----- KVAIKKIHNI FEHLSDAARI LREI-KLLRL LR---HPDIV ------EIKH IMLPPSRRDF KD------IY VVFELMDT-- DLHQVIKAND DLTKEHHQFF LYQMLRA--- ---------- ------LKYI HTANVYHRDL
  OsMPK20-5 QHTGD----- KVAIKKIHNI FEHLSDAARI LREI-KLLRL LR---HPDIV ------EIKH IMLPPSRRDF KD------IY VVFELMDT-- DLHQVIKAND DLTKEHHQFF LYQMLRA--- ---------- ------LKYI HTANVYHRDL
  OsMPK20-3 IHTGE----- KVAIKKIHNI FEHISDAARI LREI-KLLRL LR---HPDIV ------EIKH IMLPPSKMDF RD------IY VVFELMES-- DLHQVIKAND DLTREHYQFF LYQMLRA--- ---------- ------LKYI HTANVYHRDL
    AtMPK16 THTGE----- KVAIKKINDI FEHVSDATRI LREI-KLLRL LR---HPDIV ------EIKH ILLPPSRREF RD------IY VVFELMES-- DLHQVIKAND DLTPEHYQFF LYQLLRG--- ---------- ------LKYI HTANVFHRDL
  OsMPK16-1 THTGD----- KVAIKKINDI FEHVSDATRI LREI-KLLRL LR---HPDIV ------EIKH ILLPPSRREF KD------IY VVFELMES-- DLHQVIKAND DLTPEHYQFF LYQLLRG--- ---------- ------LKYI HTANVFHRDL
    AtMPK17 PHTGG----- KVAIKKMTNV FEHVSDAIRI LREI-KLLRL LR---HPDIV ------EIKH IMLPPCRKEF KD------IY VVFELMES-- DLHHVLKVND DLTPQHHQFF LYQLLRG--- ---------- ------LKFM HSAHVFHRDL
     AtMPK8 SHTGE----- RVAIKKINDV FEHVSDATRI LREI-KLLRL LR---HPDVV ------EIKH IMLPPSRREF RD------IY VVFELMES-- DLHQVIKAND DLTPEHYQFF LYQLLRG--- ---------- ------LKYV HAANVFHRDL
    AtMPK15 THTGE----- RVAIKKINDV FDHISDATRI LREI-KLLRL LL---HPDVV ------EIKH IMLPPSRREF RD------VY VVFELMES-- DLHQVIKAND DLTPEHHQFF LYQLLRG--- ---------- ------LKYV HAANVFHRDL
  OsMPK17-1 TRTGE----- RVAIKKINDV FEHVSDATRI LREI-KLLRL LR---HPDIV ------EIKH IMLPPSRREF QD------IY VVFELMES-- DLHQVIRAND DLTPEHYQFF LYQLLRA--- ---------- ------LKYI HAANVFHRDL
     AtMPK9 THSGE----- KVAIKKINDV FEHVSDATRI LREI-KLLRL LR---HPDIV ------EIKH VMLPPSRREF RD------IY VVFELMES-- DLHQVIKAND DLTPEHYQFF LYQLLRG--- ---------- ------LKFI HTANVFHRDL
  OsMPK21-1 TQTGE----- RVAIKKINDV FDHVSDATRI LREI-KLLRL LR---HPDIV ------EIKH IMLPPSRREF RD------IY VIFELMES-- DLHQVIKAND DLTPEHHQFF LYQLLRG--- ---------- ------MKYI HAASVFHRDL
  OsMPK21-2 THTGG----- RVAIKKINDV FEHISDATRI LREI-KLLRL LR---HPDIV ------EIKH IMLPPSRREF RD------IY IIFELMES-- DLHQVIKAND DLTPEHHQFF LYQLLRG--- ---------- ------MKYI HAASVFHRDL
  OsMPK17-2 THTGE----- RVAIKKINDV FEHVSDAIRI LREI-KVLRL LR---HPDIV ------VIKH IMLPPTRREF RD------IY VVFELMES-- DLHQVIEANH DLSPEHHRFF LYQLLCA--- ---------- ------LKYI HSANVFHRDL
     30260 EISGE----- TVAIKLVTRV FEKVQLAKRA LREI-TLLRH FA--NHENIT -----GLIDV DAISPDFQEM WV-------- ----LFAA-- DLHQIIKSGQ TLTNEHVQYF LYQILRG--- ---------- ------MKYI HSASVIHRDL
    130090 FVHGG----- TFAIKKLGQF LRHPKVALLA LREI-KLMSE IG--THPCLM ------GFHE LQRPLDYEHF ED------LY IIQPLMET-- DLCRIIHSKE SLSDDQVQYF LYQMLCG--- ---------- ------IHYL HSADVLHRDL
    599215                 MVTRA LEKVALAKRV LREI-TLLRH FA---HENIT -----GLIDV D--------- ---------- -------S-- DLHQIIKSGQ ALTNEHVQYF VYQILRG--- ---------- ------MKYV HSASVVHRDL
    219362 TKSQDPV--- KLAVKKICKI LQREILLKRA IREL-KLMRF FR--GHRNIV -----SLVDV YFITLSPYEG --------LY CFQEHMDY-- DLARVLYSTV QFSEFHIRNF LYQVVCG--- ---------- ------IKYI HSANVVHRDL
     56051 AKSLNSGHEH KIAIKKVTKI FNKDILLIRA IREL-KFMKF FR--GHKNIS -----TLIDL DVVYVKPYDG --------LY CFQELADL-- DLARVLYSNV QFSEFHIQNF MYQIMCG--- ---------- ------IKYI HSADVIHRDL
    139809 AKSLNSGHEH KIAIKKVTKI FNKDILLIRA IREL-KFMKF FR--GHKNIS -----TLIDL DVVYVKPYDG --------LY CFQELADL-- DLARVLYSNV QFSEFHIQNF MYQIMCG--- ---------- ------IKYI HSADVIHRDL
      1060 LKSREN-V-- PLAIKKVSDI FTKEVLLKRA LREL-KLMRY FK--GHKNII -----NLLDL DIIYIKPYDG --------LY CFQELIDY-- DLARVIHSSV QFSEFHIQNF FYQILCG--- ---------- ------LKYI HSADVIHRDL
    184799 LMEREN-V-- PLAIKKVSDI FTKEVLLKRA LREL-KLMRY FK--GHKNII -----NLLDL DIIYIKPYDG --------LY CFQELIDY-- DLARVIHSSV QFSEFHIQNF FYQILCG--- ---------- ------LKYI HSADVIHRDL
     68008 ISNKDKPV-- SMAIKKVSNI FNKEILLKRA VREL-KLMRF FK--GHKNIV -----NLYDL DIVYCTPYDG --------LY CFQELADY-- DLARVIHSSV QFSEHHIQSF LYQILCG--- ---------- ------LKYM HSADVIHRDL
     99901 ISNKDKPV-- SMAIKKVSNI FNKEILLKRA VREL-KLMRF FK--GHKNIV -----NLYDL DIVYCTPYDG --------LY CFQELADY-- DLARVIHSSV QFSEHHIQSF LYQILCG--- ---------- ------LKYM HSADVIHRDL
    104502                            MFDYT YFPP-FTLDT TN--NPEKIV -----NLYDL DIVYCTPYDG --------LY CFQELADY-- DLARVIHSSV QFSEHHIQSF LYQILCG--- ---------- ------LKYM HSADVIHRDL
    645859 FKGRSPAA-- RIAVKKISNI FNKEILLKRA IREL-KFMNF FK--GHKNIV -----NLIDL EIVTSSPYDG --------LY CYQELIDY-- DLAKVIHSSV QLSEFHIKYF LYQILCG--- ---------- ------LKYI HSADVIHRDL
    599559 FKGRSPAA-- RIAVKKISNI FNKEILLKRA IREL-KFMNF FK--GHKNIV -----NLIDL EIVTSSPYDG --------LY CYQELIDY-- DLAKVIHSSV QLSEFHIKYF LYQILCG--- ---------- ------LKYI HSADVIHRDL
 36518:4-24                                                            MATSIV -----NLIDL DIVFTKPYDG --------LY CYQELADL-- DLARVLYSNI QFSEFHIQSF IYQILTG--- ---------- ------LKYI HSADVIHRDL
    178963 NQTSE----- GVAIKKVTNV FSKKILAKRA LREI-KLLQH FR--GHRNIT -----CLVDG VRLGSHRP-- ---------- ----IRAA-- PDRRPLPVLH LPDLVRPQVH PLGQRAA--- ---------- ------SRPQ AGQPARQRRL
    196817                                                                                             MEC-- DLAAIIRSGQ PLTDAHFQSF IYQILCG--- ---------- ------LKYI HSANVLHRDL
    738557                                                                                        MDIPRPEN-- FNECYLYEGQ PLTDAHFQSF IYQILCG--- ---------- ------LKYI HSANVLHRDL
   1159263 RRSGE----- GCAIKKITNI NNKAILTKRC LREI-KLESL LS-----FIT -----CLYDM DIVFGPDGDF DE------VY LYEELMEA-- DLHAIIRSGQ PLSDAHFQSF LYQTLCG--- ---------- ------LKYI HSANVLHRDL
    176895                                                                            M DIVFQANMNF DE------VY LYEELMEA-- DLHAIIRSGQ PLTDAHFQSF LYQTLCG--- ---------- ------LKYI HSANVLHRDL
    664529                                                                            M DIVFQSSGNF DE------VY LYEELMEA-- DLHAIIRSGQ PLTDAHFQSF LYQTLCG--- ---------- ------LKYI HSANVLHRDL
   1194920                                                      M -----SHVSV TVINSRSQRL HT------YP RQEELMEA-- DLHAIIRSGQ PLSDAHFQSF LYQTLCG--- ---------- ------LKYI HSANVLHRDL
    432994 EISGE----- TVAIKLVTRV FEKVQLAKRA LREI-TLLRH FA--NHENIT -----GLIDV DAISPDFQE- --------IY IFMEPMEA-- DLHQIIKSGQ TLTNEHVQYF LYQILRGIFI QSTTSASFTI TCPHVGMKYI HSASVIHRDL
     98579 EISGE----- TVAIKLVTRV FDKVQLAKRA LREI-TLLRH FA--GHENIT -----GLIDV DAISPDFNE- --------IY IFMEV--T-- DLHQIVKSGQ TLTNEHVQYF LYQVLRG--- ---------- ------MKYV HSASVIHRDL
     88826 EISGE----- TVAIKQVNRV FEKASFSKRC LREI-TLLRH FA--NHENIT -----GLIDV DAISKDFNE- --------VY LFLEPMEA-- DLQQIIKSGQ PLTNEHVQYF LYQILRG--- ---------- ------MKYV HSASVVHRDL
     54157                                                                                                     MHRVIRT-Q DLSDDHCQYF IYQTLRA--- ---------- ------LKAL HSADVIHRDL
    914084                                                                                             MET-- DMHRVIRT-Q DLSDDHAQYF IYQTLRA--- ---------- ------LKAL HSADVIHRDL
    503422                                                                                             MET-- DMHRVIRT-Q DLSDDHAQYF IYQTLRA--- ---------- ------LKAL HSADVIHRDL
     95205                                                                                             MQT-- DLHRVIRT-Q QLTDDHCQYF VYQTLRA--- ---------- ------LKTM HSADIVHRDL
    229298                                                                            M KILSQYWTSS NPLHSMTSRR FI-ELMQT-- DLHRVIRT-Q PLSDDHCQYF IYQTLRA--- ---------- ------LKSI HSADIVHRDL
    455126                                                                                          MDSFKEIY FVQELMET-- DLHRVIKS-Q HLSDDHCQYF IYQTLRA--- ---------- ------LRSV HSADIVHRDL
    256867                                                                                             MQT-- DMHHVIRT-Q HLSPDHCEYF TYQTLLA--- ---------- ------IKSM HDADIVHRDL
    419576                                                                                    MRT SSPSSISF-- DMHRVIRT-Q ELSDDHCQYF LYQTLRG--- ---------- ------LKAL HSAAVLHRDL
     74409                                                                                             MET-- DMHRVIRT-Q DLSDDHCQYF IYQTLRA--- ---------- ------LKAM HSANVLHRDL
    521809                                                                                                     MHRVIRT-Q ELSDDHCQYF IYQTLRA--- ---------- ------LKAM HSANVLHRDL
     54281                                                                                             MQT-- DLHKVIST-Q ALTDDHCQYF IYQILRA--- ---------- ------LKML HSANVIHRDL
     48349                                                                                                                                                                ML HSANVIHRDL
    594110                                                                                          MEPMEA-- DLHQIIKSGQ PLTNEHVQYF VYQVFRG--- ---------- ------MKYI HSASVVHRDL
     27538 QPSQR----- KVAIKRI-TP FDHSMFCLRT LREI-KLLRH FH---HENI- ------ISIL DILRPPSFEM FKE-----VY LVQELMET-- DLHRVIR-TQ NLSDDHCQYF IYQ------- ---------- ---------- ----------
     96623 VPSQR----- KVAIKRI-TP FDHTMFCLRT LREI-KLLRH FH---HENI- ------ISIL DILRPSSLSE FKE-----VY LVQELMET-- DLHRVIR-TQ QLSDDHCQYF IYQTLRA--- ---------- ------LKAL HSADVLHRDL
    380926 KPSGQ----- KVAIKKI-TP FDHSMFCLRT LREM-KLLRY FN---HENI- ------ISIL DIQKPRSYET FNE-----VY LIQELMET-- DMHRVIR-TQ ELSDDHCQYF IYQTLRA--- ---------- ------LKAM HSANVLHRDL
    263236                            MFCLRT LREM-KLLRY FN---HENI- ------ISIL DIQRPRNYEG FNE-----VY LIQELMET-- DMHRVIR-TQ DLSDDHCQYF IYQTLRA--- ---------- ------LKAM HSANVLHRDL
    154671 RPSGQ----- KVAIKKI-TP FDHSMFCLRT LREM-KLLRY FN---HENI- ------ISIL DIQKPRNYES FSE-----VY LIQELMET-- DMHRVIR-TQ ELSDDHCQYF IYQTLRA--- ---------- ------LKAM HTANVLHRDL
     67562 KPSGQ----- KVAIKKI-TP FDHSMFCLRT LREM-KLLRY FN---HENI- ------ISIL DIQKPRNFET FTE-----VY LIQELMET-- DMHRVIR-TQ ELSDDHCQYF IYQTLRA--- ---------- ------LKAM HSANVLHRDL
    226444                            MFCLRT LREM-KLLRY FN---HENI- ------ISIL DIQKPRNYDT FTE-----VY LIQELMET-- DMHRVIR-TQ ELSDDHCQYF IYQTLRA--- ---------- ------LKAM HSANVLHRDL
   1137209                            MFCLRT LREI-KLLRH FR---HENI- ------ISIL DILCPPSYDD FRE-----VY LVQELMET-- DLHRVIR-TQ QLSDDHCQYF IYQTLRA--- ---------- ------LKAL HSADVLHRDL
    AtMPK13 SETNE----- EVAIKKIANA FDNRVDAKRT LREI-KLLSH MD---HDNV- ------IKIK DIIELPEKER FED-----VY IVYELMDT-- DLHQIIRSTQ TLTDDHCQYF LYQILRG--- ---------- ------LKYI HSANVLHRDL
    AtMPK11 SETGE----- EVAIKKIGNA FGNIIDAKRT LREI-KLLKH MD---HDNV- ------IAII DIIRPPQPDN FND-----VH IVYELMDT-- DLHHIIRSNQ PLTDDHSRFF LYQLLRG--- ---------- ------LKYV HSANVLHRDL
    459393 LTTKDHR--- IVALKKIRLE AEDEGVPSTA IREI-SLLKE MNDPAIVRLL NIVHADGHKL YLVFEYLDLD LKKYMEALPV SQGGRGKALP EGAGAALATL GMGPLIVKKF LSQLISG--- ---------- ------TRYC HAHRVLHRDL
     69938                                                                                                                                                              MKYV HSASVIHRDL
```

```
   591714                                                                                                        MET-- DMHRVIRT-Q DLSDDHCQYF IYQTLRA--- ---------- ------LKAM HSANVLHRDL
   107905                                                                                    MKPKSIED FTE-----VY LIQELMET-- DMHRVIRT-Q DLSDDHCQYF IYQTLRA--- ---------- ------LKAM HSANVLHRDL
    99046 RRARA----- SPLRRSPTSS ARRSSLSARC ERS------S CC--STSEIT -----CLYDM DIPRPDN--F NE------CY LYEELMEC-- DLAAIIRSGQ PLTDAHFQSF IYQILCG--- ---------- ------LKYI HSANVLHRDL
   863830 TQTGE----- DVAIKKVTKV FEKAILAKRA LREV-KLLRH FS--GHENIT -----SILDM DITNVQE--F NE------IY LVQELMEA-- DLHQIIRSEQ PLTDAHFQYF IYQICRG--- ---------- ------LKYI HSANVLHRDL
   287494 TQTNE----- GVAIKKVTNV FSKKILAKRA LREI-KLLQH FR--GHRNIA -----CLYDM DIPRPDN--F NE------TY LYEELMEC-- DLAAIIRSGQ PLTDAHFQSF IYQILCG--- ---------- ------LKYI HSANVLHRDL
   662188 RRSGE----- GCAIKKITNI NTKRILTKRC LREI-R---- ----GHKNIT -----CLYDM DIVFQPNGNF DE------VY LYEELMEA-- DLHAIIRSGQ PLTDAHFQSF LYQTLCG--- ---------- ------LKYI HSANVLHRDL
   492594 AKTGE----- SCAIKKITNI NTKRILTKRC LREI-RLLHH FR--GHKNIT -----CLYDM DIVFQPNGNF DE------VY LYEELMEA-- DLHAIIRSGQ PLTDAHFQSF VYQTLCG--- ---------- ------LKYI HSANVLHRDI
   176887 RRSGE----- GCAIKKITNI NTKRILTKRC LREI-RLLHH FR--GHKNIT -----CLYDM DIVFQANMNF DE------VY LYEELMEA-- DLHAIIRSGQ PLTDAHFQSF LYQTLCG--- ---------- ------LKYI HSANVLHRDL
   618168  MALS----- GVAIKKVTNV FSKKILAKRA LREI-KLLQH FR--GHKNIT -----CLYDM DIPRPDN--F NE------TY LYEELMEC-- DLAAIIRSGQ PLTDAHFQSF IYQILCG--- ---------- ------LKYI HSANVLHRDL
   492643    M----- GVSLLRNMPK QVKAAQSRKL LTSIPRLLHH FR--GHKNIT -----CLYDM DIVFQPNGNF DE------VY LYEELMEA-- DLHAIIRSGQ PLTDAHFQSF VYQTLCG--- ---------- ------LKYI HSANVLHRDI
   125995                                                            MTDT DMGDPSSGPL TD-------G DFTELMEC-- DLAAIIRSGQ PLTDAHFQSF IYQILCG--- ---------- ------LKYI HSANVLHRDL
   122928                                                                                                    MEC-- DLAAIIRSGQ PLTDAHFQSF IYQILCG--- ---------- ------LKYI HSANVLHRDL
   154756                        MACLRT LREM-KLLRY FN---HENI- ------ISIL DIQKPRNYES FSE-----VY LIQELMET-- DMHRVIRT-Q ELSDDHCQYF IYQTLRA--- ---------- ------LKAM HTANVLHRDL
    68423
 OsMPK20-2
 Consensus .....      .......... .......... .... ..... ..  ......      ..... .......... ...     .. ........  .......... .......... .......                     .... ..........

          601                                                                                                                                                              750
   127228                                                        MSHSRR EEITNRLKQF NAG------- -DQYE--IID VVGEGAYGVV ----CSAVHK P--------- --TGQMVAIK RILP-FDHA- ----------
   287090                                                          MSRR EEIAARLKHF NGG------- -DQYD--VVD IVGEGAYGVV ----CSAVHK P--------- --TGQMVAIK RILP-FDHA- ----------
   186494                                                        MATSRR DEIAARLKHF NGG------- -DQYD--VID IVGEGAYGVV ----CSAVHK P--------- --TGQMVAIK RILP-FDHA- ----------
   260272                                                        MSSSRQ QELASRIQHF NAG------- -EQYE--VVD IVGEGAYGVV ----CSALHK P--------- --TGQTVAIK RIQP-FEHA- ----------
   119975        M YIIEILCTCV VYLYPRINRY SLFLHKFIRT YITHTD-IYI YIYIYSSHLY PSMAARLENF NAG------- -DDYK--IVD VIGEGAYGVV ----CSAIQQ S--------- --TGRKVAIK RILP-FDHA- ----------
     2147                                                           MGT RQALPRKFAF NVS------- -DQYE--IVD VVGEGAYGVV ----CSAIHK P--------- --SGQKVAIK KITP-FDHS- ----------
   692376                                                        MSRAPT GSGSSRKISF NVS------- -EQYD--IQD VVGEGAYGVV ----CSALHK P--------- --SGQKVAIK KITP-FDHS- ----------
   698785                                                        MSRAPA SGGSSRKISF NVS------- -EQYD--IQD VVGEGAYGVV ----CSALHK P--------- --SGQKVAIK KITP-FDHS- ----------
   468116                                                        MSRAPA GSGSSRRISF NVS------- -EQYD--IQD VVGEGAYGVV ----CSALHK P--------- --SGQKVAIK KITP-FDHS- ----------
   274271                                                        MSRG-T SSGGSRKISF NVS------- -EQYD--IQD VVGEGAYGVV ----CSAFHK P--------- --SGQKVAIK KITP-FDHS- ----------
   255486                                                        MSRAGQ AGGGGRKISF NVS------- -ENYD--IQD VVGEGAYGVV ----CSALHR P--------- --SGQKVAIK KITP-FDHS- ----------
   285227                                                        MSRQPN QSG-SRKISF NVS------- -EQYE--ISD VVGEGAYGVV ----CSALHK P--------- --SGQKVAIK KITP-FDHS- ----------
    18186                                                       MSRAQPP NPAGSRKISF NVS------- -EQYD--IQD VVGEGAYGVV ----CSAIHK P--------- --SGQKVAIK KITP-FDHS- ----------
   465108                                                       MAQGQNS THGGSRKISF NVS------- -DQYD--IQD VIGEGAYGVV ----CSALHK P--------- --SGQKVAIK KITP-FDHS- ----------
  1164585                                                      MSRSNPPN NASASRKISF NVS------- -EQYD--IQD VVGEGAYGVV ----CSAIHK P--------- --SGQKVAIK KITP-FDHS- ----------
    93851                                                     MSRTAQQQ QQQGSRKISF NVS------- -EQYD--IQD VVGEGAYGVV ----CSALHK P--------- --SGQKVAIK KISP-FDHS- ----------
   108267                                                     MSRQPQQQ QG-GSRKISF NVS------- -EQYD--IQD VVGEGAYGVV ----CSALHK P--------- --SGQKVAIK KITP-FDHS- ----------
    59022                                                           MVQ GQGGSRKISF NVS------- -DQYE--IQD IIGEGAYGVV ----CSAIHK P--------- --SGQKVAIK KITP-FDHS- ----------
   370137                                                        MSRAAG SSGSSRKISF NVS------- -EQYD--IQD VVGEGAYGVV ----CSALHK P--------- --SGQKVAIK KITP-FDHS- ----------
   532216                                                        MSRAAG SSGSSRKISF NVS------- -EQYD--IQD VVGEGAYGVV ----CSALHK P--------- --SGQKVAIK KITP-FDHS- ----------
    31240                                                        MSQRAP PSGSSRKISF NVS------- -EQYD--IQD VVGEGAYGVV ----CSAVHK P--------- --SQQKVAIK KITP-FDHS- ----------
   160345                                                         MSRTP MQGGSRKISF NVS------- -DQYD--IQD VVGEGAYGVV ----CSALHK P--------- --SGQKVAIK KITP-FDHS- ----------
   212060                                                               MQGGSRKISF NVS------- -DQYD--IQD VVGEGAYGVV ----CSALHK P--------- --SGQKVAIK KITP-FDHS- ----------
   327005                                                      MANRGGPP SSGGSRKISF NVS------- -EQYD--IQD VVGEGAYGVV ----CSALHK P--------- --SGQKVAIK KITP-FDHS- ----------
    48412                                                       MSRAPAQ QGSGSRKISF NVS------- -EQYD--IQD VVGEGAYGVV ----CSALHK P--------- --SGQKVAIK KITP-FDHS- ----------
   606069                                                       MSSRAPN PSTGSRKISF NVS------- -EQYD--IQD VVGEGAYGVV ----CSALHK P--------- --SGQKVAIK KITP-FDHS- ----------
   123724                                                        MSRAPN P-TGSRKISF NVS------- -EQYD--IQD VVGEGAYGVV ----CSALHK P--------- --SGQKVAIK KITP-FDHS- ----------
   342035                                                       MSRNAPN P-SGSRKISF NVS------- -EQYD--IQD VVGEGAYGVV ----CSAIHK P--------- --SGQKVAIK KITP-FDHS- ----------
  2135321                                                        MSNVQN R-GGARKISF NVS------- -EQYD--IQD VVGEGAYGVV ----CSAIHK P--------- --SGQKVAIK KITP-FDHS- ----------
   644393                                                        MSNAQH K-GGARKISF NVS------- -EQYD--IQD VVGEGAYGVV ----CSAVHK P--------- --SGQKVAIK KITP-FDHS- ----------
   784446                                                        MSNAQN R-GGARKISF NVS------- -EQYD--IQD VVGEGAYGVV ----CSAVHK P--------- --SGQKVAIK KITP-FDHS- ----------
   567834                                                        MSNAQQ K-GGARKISF NVS------- -EQYD--IQD VVGEGAYGVV ----CSAVHK P--------- --SGQKVAIK KITP-FDHS- ----------
   389770                                                        MSNAQQ K-GGARKISF NVS------- -EQYD--IQD VVGEGAYGVV ----CSAVHK P--------- --SGQKVAIK KITP-FDHS- ----------
    63860                                                         MSSAQ- R-GGARKISF NVS------- -EQYD--IQD VVGEGAYGVV ----CSAVHK P--------- --SGQKVAIK KITP-FDHS- ----------
   131603                                                         MSSAQ- R-GGARKISF NVS------- -EQYD--IQD VVGEGAYGVV ----CSAVHK P--------- --SGQKVAIK KITP-FDHS- ----------
   495859                                                         MVQQL PPGGPRKISF NVS------- -DQYE--IQD VIGEGAYGVV ----CSAIHK P--------- --SGQKVAIK KITP-FDHS- ----------
   434551                                                         MVQQL PPGGPRKISF NVS------- -DQYE--IQD VIGEGAYGVV ----CSAIHK P--------- --SGQKVAIK KITP-FDHS- ----------
   140828 PSRPPHFHAH HIPLATATSF TTYTLSTNNN NNNNNNNNNK NNKSLFPVSN VSGHVEMSSA QRGGARKISF NVS------- -EQYD--IQD VVGEGAYGVV ----CSAVHK P--------- --SGQKVAIK KITP-FDHS- ----------
    66435 AICVLCKRLG EFDPVNCCAI SVSSRSVSLG RSIPLIVVGS QRHRL----E VLTKYDIMSR HGSSSRKISF NVS------- -DQYE--IQD VIGEGAYGVV ----CSALHK P--------- --SGQKVAIK KITP-FDHS- ----------
   383913                      M DCLRPMLAFF PRILNANNRR KTKLLK-EPS KPSIMGQQGP PAGGSRKISF NVS------- -DQYE--IQD VIGEGAYGVV ----CSAIHK P--------- --SGQKVAIK KITP-FDHS- ----------
   323815                      M DCFRPLFLFF PRILNADSRR KAKALKAKPN KPAIMGQQGT PAGGSRKISF NVS------- -DQYE--IQD VIGEGAYGVV ----CSAIHK P--------- --SGQKVAIK KITP-FDHS- ----------
   396599 RSCAPHQPWF LSPAPISFPM DCLRRFLLRF LWMFPEDLEA NTQTPQPQRS NPVDMGQQNP PAGGSRKISF NVS------- -DQYE--IQD VIGEGAYGVV ----CSAIHK P--------- --SGQKVAIK KITP-FDHS- ----------
    74447                                                         MVQQ-P PSGGSRKISF NVS------- -DQYE--IQD VIGEGAYGVV ----CSAIHK P--------- --SGQKVAIK KITP-FDHS- ----------
    83363                                                        MGQQGT PAGGSRKISF NVS------- -DQYE--IQD VIGEGAYGVV ----CSAIHK P--------- --SGQKVAIK KITP-FDHS- ----------
    50905                                                           MSR HGSSSRKISF NVS------- -DQYE--IQD VVGEGAYGVV ----CSALHK P--------- --SGQKVAIK KITP-FDHS- ----------
   650405                                                            MS QRHGSRKISF NVS------- -DQYE--ILD VVGEGAYGVV ----CSALHK P--------- --SGQKVAIK KITP-FDHS- ----------
   493847                                                           KMS QRHGSRKISF NVS------- -DQYE--ILD VVGEGAYGVV ----CSALHK P--------- --SGQKVAIK KITP-FDHS- ----------
    95409                                                             M SKSSSRKVNF NVS------- -EHYD--IQD VVGEGAYGVV ----CSAIHK P--------- --SGQKVAIK KITP-FDHS- ----------
   835420                                                            MT ATTNPRARNF NVG------- -EQYE--ILD VVGEGAYGIV ----CSAIYK P--------- --TGQKVAIK KITP-FDHS- ----------
   343609                                                             M SSKSQRKLNF NVS------- -ENYE--VLD VVGEGAYGVV ----CSALHK S--------- --SGQKVAIK KITP-FDHS- ----------
    67554                                                M CRNNMISRML WVRVHMALCG KPI------- -RKDS--RSD VLITGS---- -----SALHK P--------- --SGQKVAIK KITP-FDHS- ----------
   216015                        MSSQLP QTPIESQVLQ TSQMPSAQVS AAPQMGQNVP SPSNPNAVAF NVS------- -DHYQ--ILE MVGQGAYGVV ----CSAIHK P--------- --TQQKVAIK KIEP-FERS- ----------
    52193                                                       MNI-EQP EQAPARQISF NVS------- -SHYQ--ILE IVGEGAYGIV ----CSAIHK P--------- --LNQKVAIK KIEP-FERS- ----------
    53916                                                      MNVNQQV QQSQPRQVSF NVS------- -DHYQ--IIE IVGEGAYGIV ----CSAIHK P--------- --SNQKVAIK KIEP-FERS- ----------
    83931                                                         MSSSG HQQEPRKISF NVS------- -DHYQ--ILD IVGEGAYGIV ----CSSIHK P--------- --SQQKVAIK KIVP-FERS- ----------
    51986              MKTENRPNHG VSLEKSNQPV SVPKQPPQPS QQASIPVQAA SQPPRQHQQS TSSTPRQISF NVS------- -SKYQ--ILD VVGEGAYGVV ----CSAIHK P--------- --SGQKVAIK KITP-FERN- ----------
    49589          MTTG QTLAYQAEIY NQAQAQAQAQ AQAQAQAQAQ AQAQAQAQAQ AQAQPRQISF NVS------- -DHYQ--IID VVGEGAYGVV ----CSAIHK P--------- --SGQKVAIK KITP-FERS- ----------
   862988                                              MPDT TAAQPQAKQR GHSHAASAA- QPA-PRKVRF NVG------- -TQYQ--VLD VVGEGAYGIV ----CSAVHR P--------- --SGRKVAIK KIAP-FDHS- ----------
   833500                                            MPENAQ QAPAAAQSKS KHGHAASQV- PPA-PRKVRF NVG------- -TQYQ--VLD VVGEGAYGIV ----CSAVHR P--------- --SGRKVAIK KIAP-FDHS- ----------
   810703                                        MPAEPTQPAA AAAQAPSTKA KHGHAASTA- QPA-PRKVRF NVG------- -TQYQ--VLD VVGEGAYGIV ----CSAVHR P--------- --SGRKVAIK KIAP-FDHS- ----------
   457677                                              MPEH AQQATAPSKS KHSHASSQA- QPA-PRKVRF NVG------- -TQYQ--VLD VVGEGAYGIV ----CSAVHR P--------- --SGRKVAIK KIAP-FDHS- ----------
   161161                               MTE QAQPAAASAA PAATTTQTKA KHSHAASQAA QPA-PRKVRF NVG------- -TNYQ--VLD VVGEGAYGIV ----CSAIHR P--------- --SGRKVAIK KIAP-FDHS- ----------
  1588194                                   MPDHSQTQAQ PAQAAAATKS KHSHAASQA- QPA-PRKVRF NVG------- -TQYQ--VLD VVGEGAYGIV ----CSAIHR P--------- --SGRKVAIK KIAP-FDHS- ----------
    98485                                            MS EKAQAAQSKS KHGHAASQA- QPA-PRKVRF NVG------- -TNYQ--VLD VVGEGAYGIV ----CSAVHK P--------- --SGRKVAIK KIAP-FDHS- ----------
  1443964 GRETRAIDRT AYSTFIFLSM PDTQAAPAAA PAAAPAAPPP AAPPPAAAKP KHGHAASQA- APA-PRKVRF NVG------- -TQYQ--VLD VVGEGAYGIV ----CSALHR P--------- --TGRKVAIK KIAP-FDHS- ----------
   864221 IEEGRTLLHE VAAKIDVLS- -SVLCPPINM PEINQQAPAA AAAP---AKS KHG-AASQP- PPA-PRKVRF NVG------- -TQYQ--VLD VVGEGAYGIV ----CSAVHR P--------- --SGRKVAIK KIAP-FEHS- ----------
   903905                                            MPP AENNSSSTKS KHGHAASQG- QPA-PRKVRF NVG------- -SQYQ--VLD VVGEGAYGIV ----CSAVHR P--------- --SGRKVAIK KIAP-FDHS- ----------
  1147795                                     MSTKTSS QAQPAAAAKS KHGHAASHA- QPA-PRKVRF NVGSKIEGFA GTQYQ--VLD VVGEGAYGIV ----CSAVYK P--------- --TGRKVAIK KIAP-FEHS- ----------
   622735                                            MADA-KKS KHAHAPAQG- QPA-PRKVRF NVG------- -TQYQ--VLD VVGEGAYGIV ----CSAVHR P--------- --SGRKVAIK KIMP-FDHS- ----------
   166786                                            MSDATKKS KHAHAASQAA QPA-PRKVRF NVG------- -TQYQ--VLD VVGEGAYGIV ----CSAVHR P--------- --SGRKVAIK KIMP-FDHS- ----------
  2641010                                        MSDQRQQARA KHGHAASTA- APA-PRKVRF NVG------- -TNYQ--VLD VVGEGAYGIV ----CSAIHR A--------- --SGRKVAIK KIAP-FDHS- ----------
    28679                                       MPSN MLGANAGTQS -KSRSQAQA- --A-PRKVRF NVG------- -TQYQ--VLD VVGEGAYGIV ----CSAIHR P--------- --SGRKVAIK KIAP-FDHS- ----------
   101027                                       MTDN AQQAPAQTQS SQAKSRSQA- --A-PRKVRF NVG------- -TQYQ--VLD VVGEGAYGIV ----CSAVHR P--------- --SGRKVAIK KIAP-FDHS- ----------
   564549                                            MSDPAAKT KHAASNNQ-- -PA-PRKVRF NVG------- -TQYQ--VLD VVGEGAYGIV ----CSAVHR P--------- --SGRKVAIK KIAP-FDHA- ----------
   227465                                 MADAAAATK DTKDAAASKA KHGASASQG- QSA-PRKVRF NVG------- -TQYQ--VLD VIGEGAYGIV ----CSALHR P--------- --SGRRVAIK KIAP-FEHS- ----------
    18095                                       MPETTTTTQQ QQPAKATKPA AAA-PRKVRF NVG------- -SQYQ--VLD VVGEGAYGIV ----CSALHK P--------- --SGRKVAIK KIAP-FDHS- ----------
   338545                                             MLHL MPS-RHQGRC DS-------- -TSYQ--VLD VVGEGAYGVV ----CSAVYK P--------- --TGRKVAIK KIAP-FEHS- ----------
   134728                                 MST ATDSSATAVA SSQPTSEKKA KHGHAASQA- QSA-PRKVRF NVG------- -TQYQ--VLD VVGEGAYGIV ----CSAVHR P--------- --SGRKVAIK KIAP-FEHS- ----------
   275037                                          MPE QASQPAAAKA KHSH-AASQA QPA-PRKVRF NVG------- -TQYQ--VLD VVGEGAYGIV ----CSAIHR P--------- --SGRKVAIK KIAP-FDHS- ----------
   503339                                        MPEINQ QAPAAAAAPA KSKHGAASQP PPA-PRKVRF NVG------- -TQYQ--VLD VVGEGAYGIV ----CSAVHR P--------- --SGRKVAIK KIAP-FEHS- ----------
    40745                      M AAAVANPIAA ATTQPAAAAP AAAAPATTGA AAPVVKQSST KSAEPRRVRF NVG------- -SKYH--VMD VIGEGAYGVV ----CSAVHR P--------- --TGQKVAIK KITP-FDHS- ----------
    43876 PVPGQSAPHD QPAAGP--SS EQGGVGASGG KLGEPGQGKP PAQEEQGQLA RDKPSSKKDD KK-EPRKIRF SVG------- -QKYQ--VQD VIGEGAYGVV ----CSAIHK A--------- --TGQRVAIK KVMP-FDHS- ----------
    14716 PVPGQSAPHD QPAAGP--SS EQGGVGASGG KLGEPGQGKP PAQEEQGQLA RDKPSSKKDD KK-EPRKIRF SVG------- -QKYQ--VQD VIGEGAYGVV ----CSAIHK A--------- --TGQRVAIK KVMP-FDHS- ----------
    15138 ALPPQPQPAP VASSSR--SN QADKGDGVAD PTGDKGSGEY SQSQEKGARA GQEQRGSGDV KKQEPRKVRF SVG------- -TKYA--VQD VIGEGAYGVV ----ASAVHR P--------- --TGQKVAIK KIEP-FDHS- ----------
    67488                                                      MSSNQQN QQNQQRRIKF SVG------- -TKYQ--VLD VIGEGAYGVV ----CSAIHR P--------- --SGHKVAIK KIEP-LMHQ- ----------
    28680                                            MTDSQQ AGTQSKSRSQ AQAAPRKVRF NVG------- -TQYQ--VLD VVGEGAYGIV ----CSAIHR P--------- --SGRKVAIK KIAP-FDHS- ----------
   177514                                                       MSS RKSSKPLPKF EIG------- -DDYQ--LLH AIGEGAYGTV ----AALHK P--------- --TGRQVAIK KILP-FDHT- ----------
    53880                                                       MSS RKASKPLPKF EIG------- -NDYQ--ILH AIGEGAYGTV ----AALHK P--------- --TGRQVAIK KILP-FDHT- ----------
   778168                                 MKMPH---- ----SSILVV T------IMS RKTQKPLPKF DTG------- -EDYQ--ILH AIGEGAYGTV ----VSATHK P--------- --TGRQVAIK KILP-FEHT- ----------
   388025                                              MTAAA A----PQAGN SKTKKPMPRF EIG------- -PDYQ--ILH SLGEGAYGTV ----AAALHR P--------- --SGRQVAIK KILP-FEHT- ----------
   581713                                              MTVST AQ-----APK PKSQKPIPKF DIG------- -PDYQ--ILH SLGEGAYGTV ----AAALHK P--------- --SGHNVAIK KILP-FDHS- ----------
   229317    MSYCSNL NIIHTRSESV LQTFCQSCLF LLALAQ---- ----TMTAAT QQGAGPAAPK PKAQKQVPRF DIG------- -PEYE--ILH SLGEGAYGTV ----AAAMHK P--------- --SGRQVAIK KILP-FDHT- ----------
   829657                                              MVAAP -------AFK VKVAKPLPRF DVG------- -PDYE--ILH CLGEGAYGTV ----VAASHK P--------- --TGRQVAIK KILP-FDHT- ----------
    70959                                                       MSR -KTQKPLPKF EIG------- -DDYR--VLH GLGEGAYGTV ----VAAVHK P--------- --TGREVAIK KVLP-FEHT- ----------
    32857                                                       MSR SKVAKPVPKF EVG------- -DDYR--MLH SLGEGAYGTV ----VAAVHK P--------- --TGREVAIK KVLP-FEHT- ----------
   561172 FLLLDQAPAL DLPRSARTVR PEVICKLAWL TSHINQLERN FLKWAQGRAP YSFASLIMSR -KAQKPLPPF HVG------- -DDYK--ILH GLGEGAYGTV ----VAALHK P--------- --TNREVAIK KLLP-FEHT- ----------
   573944                                                       MSR -KAQKPLPPF HLG------- -DDYK--ILH GLGEGAYGTV ----VAALHK P--------- --TNREVAIK KVLP-FEHT- ----------
   740770                                                     MSKSQ QKTAKPLPKF DVG------- -ADYK--LLH SLGEGAYGTV ----VAALHK P--------- --SGRQVAIK KVLP-FEHT- ----------
    16071                                      MSVLGMVST TIIDGPIHIY STWQFLLQDT QTC------- -AEIRSRVLN NLGEGAYGTV ----VSALHI P--------- --SGNQVAIK KVRP-FEHV- ----------
  1126092                                                        MP SKTHKPVPKF EVG------- -DDYR--VLN NLGEGAYGTV ----VSALHI P--------- --SGNQVAIK KVRP-FEHV- ----------
   478842                                                         M PRPSKNNTKF DLG------- -PDYK--VLN IIGEGAYGTV ----CSAVHI P--------- --TARNVAIK KVLP-FDHT- ----------
    46649                             M SKKVGK---- ---SLGKFNV GTNGQSLNPR PQLISIDPLR LPG------- -DDYK--ILF GLGEGAYGTV ----AAAVHK P--------- --TGRQVAIK KVLP-FEHT- ----------
   601377                                                      MTVR TSAKKPLPTF EVG------- -DDYR--ILH VLGEGAYGTV ----VAALHK P--------- --SGRQVAIK KVLP-FDHT- ----------
   866574                                                 MGLALEQL STMEEHMRQS LLA------- -SGYE--PLD LIGEGAYGVV ----CSALHM P--------- --TQRKVAIK RITP-FDHT- ----------
   521401                                                            MAR------- -SAHS--SLS TMDSGS-SIH ----SSALHM P--------- --TQRKVAIK RITP-FDHT- ----------
   850251                                                                 MSR-SIS SVRARTASYG ----TSALHM P--------- --TQRKVAIK RITP-FDHT- ----------
   832111                             M DPHQQLLVEP SGRRRSTC-- --------TD MSYDHRLDNF PLD------- -SAYR--VLE VIGEGAYGIV ----CSAIHQ P--------- --SQRKVAIK RITP-FDHS- ----------
   832110                                                       MSYDHRLDNF PLD------- -SAYR--VLE VIGEGAYGIV ----CSAIHQ P--------- --SQRKVAIK RITP-FDHS- ----------
   676666                                                         M SYTDLRLDSF CVD------- -GTYQ--VLD VIGEGAYGTV ----CSAIHI P--------- --SQRKVAIK RITP-FDHA- ----------
  1143376                                                        MSSDSRLDNF SVG------- -GTYR--VLE VIGEGAYGIV ----CSATHI S--------- --SQRKVAIK RITP-FDHT- ----------
   457401                                                         M GTLDHRLENF VVG------- -TVYQ--VLD VIGEGAYGIV ----CSAIHI P--------- --TQRKVAIK RITP-FDHS- ----------
   834255                                                          MDLSLEDF CAD------- -KSYR--ILE LIGEGAYGIV ----CSAIHH P--------- --SQRKVAIK RIAP-FDHS- ----------
   133042                                                          MADERMQSF VVG------- -SNYQ--VLD VIGEGAYGIV ----CSAMHL P--------- --SQRKVAIK RIAP-FDHS- ----------
    30507                                                          MSDERIATF VVG------- -SSYQ--VLD VIGEGAYGIV ----CSALHI P--------- --SQRKVAIK RITP-FDHT- ----------
   646041 KERRLRRRRT RLEDSEQAGD RSHPHPEPRA PASVGLYLSP SASAIAPS-- -------PVL AAMDDRKDSF VVG------- -SNYE--VLD VIGEGAYGIV ----CSALHI P--------- --SQRKVAIK RITP-FDHS- ----------
```

```
    570964  RARARAGAGA EKADASRSGT RRSGVCCYYP ALLVALLLL- ---LVLLL-- -------PSR PAMDDRMSSF VVG------- -SNYQ--VLD VIGEGAYGIV ----CSALHV P--------- --SQRKVAIK RITP-FDHS- ----------
    301734  HSEQRPIGRA ARHQKYSSLL KSLESPFSNR SLVSGVYLAS DCDHRNPALI RTFWLRAHSE TMDAARVENF IVG------- -HNYQ--LQE VIGEGAYGIV ----CSATHV P--------- --SQRKVAIK RITP-FDHS- ----------
    154378  GQDEPAVHEP TLKNLQGTWD RSFAVPFTVS ALAFSTLAIA HDDDTD---- ---------D METDSRLANF AVG------- -QNYS--LLD VIGEGAYGVV ----CSAVHV P--------- --SGRKVAIK RITP-FDHS- ----------
     90189                                                                         MAF IQS------- -SNYQ--PLD VIGEGAYGVV ----CSAVHV P--------- --TQRKVAIK RITP-FDHS- ----------
    448371                                                             MASTS SAPDERLLAF NPG------- -PNYE--VID LIGEGAYGIV ----CSAIHV P--------- --SQQRVAIK KISP-FDHS- ----------
    476779                                                             MDHTS SAPDERLLAF NPG------- -PNYE--VID LIGEGAYGIV ----CSAIHV P--------- --SQQRVAIK KISP-FDHS- ----------
     68819                                                             MDTTD TTIDHRLANF DVG------- -ANYQ--VLD VIGEGAYGVV ----VSALHT P--------- --SSRKVAIK RITP-FDHT- ----------
   2671102                                                                     MDERTTQF VIQ------- -SNYQ--PLD VIGEGAYGVV ----CSALHM P--------- --TQRKVAIK RITP-FDHS- ----------
    940143                                                           MATEVPA LDRDERIVGF AAG------- -SSYE--VVE LIGEGAYGIV ----CSAIHI P--------- --TQRKVAIK RITP-FDHS- ----------
    585786                                                           MEQSNEH NNVDERIIGF AAG------- -SNYD--VQE LIGEGAYGIV ----CSAVHI P--------- --TGRKVAIK RITP-FDHT- ----------
    349085                                         MSEL SGSQRAPA-- ----TRSSNS SVRGSQHFRL LLL------- -ISRL--FHS SLGEGAYGIV ----CSAIHI P--------- --TQRKVAIK RITP-FDHS- ----------
   1539717                                                                     MDDRLSGF NVG------- -DNYQ--IID IIGQSVHPLT ----LRIWVL SQRNTRGRSV RDSMRKVAIK RITP-FDHS- ----------
     24638                                                                        MAAFA LAH------- -QDYR--IID VIGEDLTLLL NH-GSSAEHT P--------- --SGRRVAIK KITP-FDHA- ----------
     75000                                                                                              MN TTGEGAYGVV ----CSAEHT P--------- --SGRRVAIK KITP-FDHA- ----------
   1245527                                                                        MDAAF LVG------- -HNYT--VLD VIGEGAYGVV ----CSAIHT P--------- --TQRKVAIK RITP-FDHS- ----------
    103018                                                               MAA AAPPAASRQF NVG------- -KDYQ--IVD CIGEGAYGVV ----VSAIHL P--------- --TQQKVAIK KITP-FDHS- ----------
     43883                                                                        MDRKF DIG------- -DDYK--VMD VVGEGAYGLV ----SSAVHL P--------- --TQSKVAVK RIQP-FDHS- ----------
     58913                                                                        MDRNF NVG------- -TDYT--VLD VVGEGAYGVV ----CSAVHE P--------- --SKQKVAIK KITP-FDHSS LSLFLPLTSL
     18326                                                                           MD NFS------- -VGDAYQVQD VIGEGAYGIV ----CSAVHL A--------- --SGRKVAIK RITP-FDHS- ----------
    301059                                                                                       MNTSHEYST RAGEGAYGIV ----CSAVHL A--------- --SGRKVAIK RITP-FDHS- ----------
     12410  GNGEMGNGDM GNGDLSAASL ARIVTILRAS ALVNLQRLSS PKKSAEQALK GEWHRVEGQH GWQESLIVIT PEW------- -QDRQAPSAS RQREGAYGIV ----CSAVHL A--------- --SGRKVAIK RITP-FDHS- ----------
     18327                                                                                                    MGEGAYGIV ----CSAVHL A--------- --SGRKVAIK RITP-FDHS- ----------
    184754                                                               MDG KVEEPRKIVF NIS------- -NQFQ--VQK IIGEGSYGIV ----SLAVHK P--------- --TGTKVAIK KIEP-FQRA- ----------
     99455                                                                     MGQKRITF NIS------- -TSFE--VQQ LLGEGAYGVV ----CLALHK P--------- --TGTKVAIK KIQP-FERS- ----------
    706256                                                                      MPKRIVY NIS------- -SDFQ--LKS LLGEGAYGVV ----CSATHK P--------- --TGEIVAIK KIEP-FDKP- ----------
    653342                                                                      MPKRIVY NIS------- -SDFQ--LKS LLGEGAYGVV ----CSATHK P--------- --TGEIVAIK KIEP-FDKP- ----------
    462249                                      MPSDTTT QQQPAAAAKS KHSHTASSTQ QQQ-PRKVRF NVG------- -THYA--VQD VVGEGAYGIV ----CSAVHR P--------- --TGRKVAIK KIAP-FDHS- ----------
    326973
     77855
    338563
    233520
     73610
    150924
    348809
    913771                                                                                                   MSSAKAHTA- ----SSALHS ---------- --TAQPAAKS PSRR-SRPS- ----------
    913918
    747578                                                                      MARTITF DIP------- -SQYK--LVD LIGEGAYGTV ----CSAIHK P--------- --SGIKVAIK KIQP-FSKK- ----------
    458977                                                                      MARTITF DIP------- -SQYK--LVD LIGEGAYGTV ----CSAIHK P--------- --SGIKVAIK KIQP-FSKK- ----------
     41459                                                           MVALTKK EKTSKPLPKF HVG------- -DDYQ--ILH SLGEGAYGTV ----VAAKHL P--------- --SDRQVAIK RILP-FEHT- ----------
   1397463                                                                MS SRKQKPLPPF HVG------- -DDYK--ILH GLGEGAYGTV ----VAALHK P--------- --TNREVAIK KVLP-FEHT- ----------
    149203
    102078                                                                  MKMSSRKVVF NIS------- -RSYE--TKQ VLGEGAYGVV ----ASAVHK P--------- --TGLTVAIK KIEP-FSKT- ----------
36766:44-3                                                                         MVVF NVS------- -QSFK--VVR VLGEGAYGIV ----SLAIHV P--------- --TGTQVAIK KIEP-FDKP- ----------
     54278                                         MIVN KFNRYNQLKN SNQQSRGSIN QSQPLRKVVF NVS------- -ESLK--VVK ILGEGAYGIV ----ALAIHT P--------- --TGTKVAIK RIEP-FERP- ----------
     29185                                                                                       MSGKYRVQD VIGEGAYGVV ----ASAVHR P--------- --TGQKVAIK KIAP-FDHS- ----------
    790328  GYFVAELMSL DRQEVKEAEV IRLLEAATRA AGM-CVERAG TVDSMPAAEE VERRFGDMGL ALEQLSTMEE HMR-Q--SLL ASGYE--PLD LIGEGAYG-- -------V-- ---------- ----RKVAIK RITP-FDHT- ----------
    765763  GYFVAELMSL DRQEVKEAEV IRLLEAATRA AGM-CVERAG TVDSMPAAEE VERRFG---- ----LSTMEE HMR-Q--SLL ASGYE--PLD LIGEGASA-- -------LHM P--------- -TQ-RKVAIK RITP-FDHT- ----------
   1491990  EDLWALANQC WAFEPGDRPK MKSNEGVSTF DYLHAILHRR ERDYWNSKWW FNRISHPLLS QLYRSTSPQK FVD-E--VET AERIQ--SVD M-QERLKK-- -------QQM E--------- -EM-RKVAIK RITP-FDHS- ----------
     32998  REGTSAGSVQ GEWECVEEGY IVEQEQQLQV TVIGSRGSCA DPLHYPAMSE KAQAAQSKSK HGHAASQAQP APR-KVRFNV GTNYQ--VLD VVGEGAYGIV ----CSAVHK P--------- -SG-RKVAIK KIAP-FDHS- ----------
   2601990  SCLKLITMCE CKRGRVLEGS RRHCEVQART VSAYTLRSDS NPLVWPSRGP RVRSLAC--- ---AASAAI- -------FVI QSNYQ--PLD VIGEGAYGVV ----CSALHM P--------- -TQ-RKVAIK RITP-FDHS- ----------
    262460  ELQKLEEENQ LRVEHLKGDD TVFEDLGASG KDYPLKSTWR QGTALRHRSP ASPAQHEKSN QYPTAGQAGG GGR-KISFNV SENYD--IQD VVGEGAYGVV ----CSALHR P--------- -SG-QKVAIK KITP-FDHS- ----------
     11717  SAAPYKSGVL AGRGASAYQN VGLEDEYNKA STRSSVAAGN GHGRLPRAAF GASSSSSGLF PMMRSQTHSF NAN-GQVFTL ETRYQ--LVK PIGNGAYGAV ----IAVKDV V--------- -DGGENLAVK KITNIFEDL- ----------
    532299  SVAPYKSAVL AGRGVSAYQS VGLEDEYTKA SSRSSVA--N GRGRLPRAAF GATTSSSGLL PMLRPQTHSF NAN-GQVFTL DTRYQ--LVK PIGNGAYGAV ----IAVKDV V--------- -NGGENLAVK KITNIFEDL- ----------
    551831  SVAPYKSAVL AGRGVAAYQS VGLEDEYTKA GSRSAVA--N GRGRLPRAAF GATTSSSGLF PTLRPQTHSF NAN-GQVFTL DTRYQ--LVK PIGNGAYGAV ----IAVKDV V--------- -NGGENLAVK KITNIFEDL- ----------
    301484  SVAPYKSAVL AGRGVSAYQS VGLEDEYTKA SSRSSVA--N GRGRLPRAAF GATTSSSGLL PMLRPQTHSF NAN-GQVFTL DTRYQ--LVK PIGNGAYGAV ----IAVKDV V--------- -NGGENLAVK KITNIFEDL- ----------
     42366  NTSTSAKKGA GARGKGTRSP RPPMEDSKTE LSDPAIANTN DSPSKDGVSA KKL------- ----PRRHSF HAL-NSTFVV DSEYQ--FVK ELGQG----- ---------- ---------- ----EGCAIK KITN-INTK- ----------
    476004  AMVPSKDGSF VTYGKQASED LKNEERSRAE AAKKLREKQR AD---EAAAA ANAANKPKYL PATRPGTQCF MTS-NTRFEI PSSFE--YVK TIGSGAYGVV ----ISAADA K--------- -TG-KTVAIK NIQRAFDDL- ----------
    558476  AMVPTKDGSF VTYGKQASED LKQEERSRAE AAKKLREKQR AD---EAAAA ANAANKPKYL PATRPGTQCF MTS-NTRFEI PSNFE--YVK TIGSGAYGVV ----ISATDS K--------- -SG-KTVAIK NIQRAFDDL- ----------
      9954  PASSSPVKSV GSAPLSSSRK LDG------- --SKSAAPVG ASDAVSGTRT A-AATT-VAA APARPGTYSF VVA-GTNFQI DEKYK--FIK VIGRGAYGVV ----ISAENA E--------- -TN-EKVAVK KISRAFEDL- ----------
     82136  PASSSPLKSV GSPPLSASKS LSAKGLPNSG RSSETSAPVG ASDAVSSTRK A-SATTAVAA PPARPGTYSF VVA-GTNFQI DEKYK--FIK VIGRGAYGVV ----ISAENT E--------- -TN-EKVAVK KISRAFEDL- ----------
    312415  PASSSPLKSV GSPPVSASKI LSAGGLPSSD RSSDTSAPVG ASDAVSSTRK A-SSTTTIAA PPARPGTYSF VVA-GTNFQI DEKYK--FIK VIGRGAYGVV ----ISAENA E--------- -TN-EKVAVK KISRAFEDL- ----------
    354114  HARRHGWANV KLSPEDTART QSAGAIVSAR NDDGDDTVHK QFPAGDGSSA GLPAATDAVI CKENADMHTF MVC-GAPFTI PRHYQ--LIK VCGRGAYGIV ----IAATNL R--------- -TG-GNVAIK KVIDCIWHP- ----------
     34134  VKAPERSVSK TPAKKRTSED GKHEESSSSH ASPGSSEGRR GRSRTRNSAA HHNHIDQPGH TTERQGYHSL MSSFGKVFHV EKRWK--LIR EMGSGAYGYV ----ISAVDE V--------- -SG-EPVAIK MITRVTQKI- ----------
     20005  MAPRGPSGMN ISQMHMGMGQ PQGGMQQQQQ QNSFQGLQWQ VYLEKLLNSL PILFDHVDRA IPLRPRLPLR HCV------- -SRCLR-SPF -VGGGSYGIV ----CSAEDT L--------- --RGRKVAIK KITDVFDDL- ----------
    518921                                                 MSIE PSRFGPDFHS VTVSR--DVF EVR------- -SHYVN-LRP -VGGGSYGIV ----CSAEDT L--------- --RGRKVAIK KITDVFDDL- ----------
    503206                                                 MAIE PSRYGPDFHC VTVSR--DVF EVR------- -SHYVN-LRP -VGGGSYGIV ----CSAEDT L--------- --RGRKVAIK KITDVFDDL- ----------
     10810                                                 MSIE PSRYGPDFHC VTVSR--DVF E--------- ---------- ---------- ---------- L--------- --RGRKVAIK KITDVFDDL- ----------
    AtMPK1                                              MATLVDP PNGIRNEGKH Y-FSMWQTLF EID------- -TKYMP-IKP -IGRGAYGVV ----CSSVNS D--------- --TNEKVAIK KIHNVYENR- ----------
    AtMPK2                                              MATPVDP PNGIRNQGKH Y-FSMWQTLF EID------- -TKYVP-IKP -IGRGAYGVV ----CSSVNR E--------- --SNERVAIK KIHNVFENR- ----------
    OsMPK7                                              MAMMVDP PNGMGNQGKH Y-YTMWQTLF EID------- -TKYVP-IKP -IGRGAYGIV ----CSSINR A--------- --TNEKVAIK KINNVFDNR- ----------
   OsMPK14                                              MAIMVDP PNGMGNQGKY Y-YSMWQTLF EID------- -TKYVP-IKP -IGRGAYGIV ----CSSINR E--------- --TNEKVAIK KIHNVFDNR- ----------
   AtMPK14                                              MAMLVDP PNGIRQEGKH Y-YTMWQTLF EID------- -TKYVP-IKP -IGRGAYGVV ----CSSINS E--------- --TNERVAIK KIHNVFENR- ----------
    AtMPK7                                              MAMLVEP PNGIKQQGKH Y-YSMWQTLF EID------- -TKYVP-IKP -IGRGAYGVV ----CSSINR E--------- --TNERVAIK KIHNVFENR- ----------
   AtMPK12                                         MSGE SSSGSTEHCI KVVPTHGGRY VQYNVYGQLF EVS------- -RKYVPPIRP -IGRGACGIV ----CAAVNS V--------- --TGEKVAIK KIGNAFDNI- ----------
    AtMPK4                                       MSAESC FGSSGDQSSS KGVATHGGSY VQYNVYGNLF EVS------- -RKYVPPLRP -IGRGAYGIV ----CAATNS E--------- --TGEEVAIK KIGNAFDNI- ----------
  OsMPK4-1                                       MDSSSG GAGGGGGAQI KGMGTHGGRY VLYNVYGNFF EVS------- -SKYAPPIRP -IGRGAYGIV ----CAAVNS E--------- --NGEEVAIK KIGNAFDNH- ----------
 OsMPK16-2                   MRME GGGGGGHGHH GGGGGGHGHH GGIGGGEAQI KGTLTHGGRY VQYNVYGNLF EVS------- -SKYVPPIRP -VGRGACGII ----CAVVNA Q--------- --TRQEVAIK KIGNAFDNQ- ----------
    AtMPK5                                       MAKEIE SATDLGDTNI KGVLVHGGRY FQYNVYGNLF EVS------- -NKYVPPIRP -IGRGAYGFV ----CAAVDS E--------- --THEEIAIK KIGKAFDNK- ----------
   AtMPK10                MEPTNDA ETLETQGEVT TAIWPSSQIL KT----TIDI PGTLSHDGRY IQYNLFGHIF ELP------- -AKYKPPIRP -IGRGACGIV ----CSAVDS E--------- --TNEKVAIK KITQVFDNT- ----------
    AtMPK6          MD GGSGQPAADT EMTEAPGGF- PAAAPSPQMP G-----IENI PATLSHGGRF IQYNIFGNIF EVT------- -AKYKPPIMP -IGKGAYGIV ----CSAMNS E--------- --TNESVAIK KIANAFDNK- ----------
    OsMPK6          MD AGAQPP--DT EMAEAGGGQQ PPAAAAAAGA GAGAGMMENI QATLSHGGRF IQYNIFGNVF EVT------- -AKYKPPILP -IGKGAYGIV ----CSALNS E--------- --TGEQVAIK KIANAFDNK- ----------
    AtMPK3                                          M NTGGGQYTDF PAVETHGGQF ISYDIFGSLF EIT------- -SKYRPPIIP -IGRGAYGIV ----CSVLDT E--------- --TNELVAMK KIANAFDNH- ----------
    OsMPK3                                             MDGAPVAEF RPTMTHGGRY LLYDIFGNKF EVT------- -NKYQPPIMP -IGRGAYGIV ----CSVMNF E--------- --TREMVAIK KIANAFNND- ----------
    206269                                                             MAEF VKLSIFGTVF EVTTRY---- -----VDLQP -VGMGAFGLV ----CSAKDQ L--------- --TGMNTAVK KIMKPFSAP- ----------
    154265                                                             MAEF VKLSIFGTVF EVTTRY---- -----VDLQP -VGMGAFGLV ----CSSKDQ L--------- --TGMNVAIK KIMKPFSAP- ----------
    168032       MVPSI YGNTDLIKAV RKSERDKERY FDRDNITMTM AMAMLIIIII KEDSKVMAEF VKLSIFGTVF EVTTRY---- -----IDLQP -VGMGAFGLV ----CSSKDQ L--------- --TGTSVAIK KIMKPFSAP- ----------
    307550        MSRV FLNTNYVKFH RTWFQIFVFF -----LFLFL FVCLSFFFIL K-----MAEF VKLSIFGTVF EVTTRY---- -----VDLQP -VGMGAFGLV ----CSAKDQ L--------- --TGMNVAIK KIMKPFSAP- ----------
    320251  ELIKKGETIK KYT---KELI ALGRTSVPED VAKLVSFLSS RDSDYITGQ- -TMVVDMAEF VRAQIFGTTF EITSRY---- -----TDLQP -VGMGAFGLV ----CSAKDQ L--------- --TNQAVAVK KIMKPFSTP- ----------
    356517  PFRQ---SIF DFSAPPNSFT ILP--FSPPE YLSLLTRSDE QLSHHIVAN- KRSAPTMAEF IRAQIFGTTF EITSRY---- -----SDLQP -VGMGAFGLV ----CSARDQ L--------- --TNQNVAVK KIMKPFSTP- ----------
    113434  PARQPSTTPF PSSVHPSVLI VLRVNFTAQT TPHPNQRNNE CRSSNSASSV PRSRWLVAGS LRVGCVGLDG KVTTRY---- -----VDLQP -VGMGAFGLV ----CSAKDQ L--------- --TGASVAVK KIMKPFSTP- ----------
     28887  RESLERFVRN PLRPDPQILR SLPLRLVSDR PPAPYPPPRT ADASIRLGPL STRPSRLSPS VLPRV----- PINGRL---- -----CQTQH -LWYSI---- ------RSNY L--------- --TGQSAAIK KIMKPFSTP- ----------
     16635  PLTWSTYAFA PPANELTRAV SREGHARKNL STNNPTHQQE TPPSFLLSYD YEYDTTMAEF IRAQIFGTCF EITSRY---- -----VDLQP -VGMGAFGLV ----CSAKDQ L--------- --TNQPVAIK KIMKPFSTP- ----------
      6923  VAKEVQQAST RDALLGGVSS STDDPYGTLD GATVSDRTRL LTGSQSLQD- STKRLQDSQR IALETEELGG DI---L---- -----RNLQP -VGMGAFGLV ----CSAKDQ L--------- --MGTSVAIK KIMKPFSTP- ----------
    807502                                                              MSF VKLSIFGTSF EVTTRY---- -----VDLQP -VGMGAFGLV ----CSAKDQ L--------- --TGASVAIK KIMKPFSTP- ----------
    129058                                                              MSF VKLSIFGTSF EVTTRY---- -----VDLQP -VGMGAFGLV ----CSAKDQ L--------- --TGSSVAIK KIMKPFSTP- ----------
    359241                                                              MSF VKLSIFGTSF EVTTRY---- -----VDLQP -VGMGAFGLV ----CSAKDQ L--------- --TGSSVAIK KIMKPFSTP- ----------
     53282                                                              MSF VKLSIFGTSF EVTTRY---- -----VDLQP -VGMGAFGLV ----CSAKDQ L--------- --KGTSVAIK KIMKPFSTP- ----------
    117789                                                              MSF VKLSIFGTSF EVTTRY---- -----VDLQP -VGMGAFGLV ----CSAKDQ L--------- --TGSSVAIK KIMKPFSTP- ----------
    170586                                                              MSF VKLSIFGTSF EVTTRY---- -----VDLQP -VGMGAFGLV ----CSAKDQ L--------- --TGASVAVK KIMKPFSTP- ----------
    162119                                                              MSF VKLSIFGTSF EVTTRY---- -----VDLQP -VGMGAFGLV ----CSAKDQ L--------- --TGSSVAIK KIMKPFSTP- ----------
    560244                                                              MSF VKLSIFGTSF EVTTRY---- -----VDLQP -VGMGAFGLV ----CSAKDQ L--------- --TGSSVAIK KIMKPFSTP- ----------
    499279                                                              MSF VKLSIFGTSF EVTTRY---- -----VDLQP -VGMGAFGLV ----CSAKDQ L--------- --TGSSVAIK KIMKPFSTP- ----------
    898207                                                             MSFF VKLSIFGTSF EVTDRY---- -----VDLQP -VGMGAFGLV ----CSAKDQ L--------- --TGASVAVK KIMKPFSTP- ----------
    500359                                                              MSF VKLSIFGTSF EVTTRY---- -----VDLQP -VGMGAFGLV ----CSAKDQ L--------- --TGAAVAVK KIMKPFSTP- ----------
    390416                                                              MSF VKLSIFGTSF EVTTRY---- -----VDLQP -VGMGAFGLV ----CSAKDQ L--------- --TGSSVAVK KIMKPFSTP- ----------
     61586                                                              MSF VKLSIFGTSF EVTTRY---- -----VDLQP -VGMGAFGLV ----CSAKDQ L--------- --TGSSVAIK KIMKPFSTP- ----------
     74488                                                              MSF VKLSIFGTSF EVTTRY---- -----VDLQP -VGMGAFGLV ----CSAKDQ L--------- --TGSSVAIK KIMKPFSTP- ----------
    155373                                                             MAQF AKLSIFGTVF EVTTRY---- -----VDLQP -VGMGAFGLV ----CSAKDE L--------- --TGQPVAIK KIMKPFSTP- ----------
     56185                                                             MADF VKLSIFGTVF EVTTRY---- -----VDLQP -VGMGAFGLV ----CSAKDE L--------- --TGQMTAIK KIMKPFSTP- ----------
     14909                                                             MADF VKLSIFGTVF EVTTRY---- -----VDLQP -VGMGAFGLV ----CSAKDE L--------- --TGQSAAIK KIMKPFSTP- ----------
    176707                                                                           M PLEATT---- -----LTIHP -ATPWNVNNL ----SSAKDQ L--------- --MGTSVAIK KIMKPFSTP- ----------
    176721                                                              MSF VKLSIFGTSF EVTTRY---- -----VDLQP -VGMGAFGLV ----CSAKDQ L--------- --MGTSVAIK KIMKPFSTP- ----------
    240717                                                                  MDADCLHRS- QVTTRY---- -----VDLQP -VGMGAFGLV ----CSAKDQ L--------- --MGTSVAIK KIMKPFSTP- ----------
     56428                                                 MCVR LSSDASVSTI VRTQIFGTSF EITSRY---- -----ADSNP -VGMGAFGLV ----CSARDQ L--------- --TGQAVAIK KIMKPFSTP- ----------
     97915                                                             MADF IRTQIFGTSF EITSRY---- -----ADSNP -VGMGAFGLV ----CSARDQ L--------- --TGQAVAIK KIMKPFSTP- ----------
    707387                                                             MAEF VRAQIFGTTF EITSRY---- -----TDLQP -VGMGAFGLV ----CSAKDQ L--------- --TSQAVAIK KIMKPFSTP- ----------
    504013                                                             MAEF VRAQIFGTTF EITSRY---- -----TDLQP -VGMGAFGLV ----CSAKDQ L--------- --TNQAVAIK KIMKPFSTP- ----------
     37843                                                             MAEF VRAQIFGTTF EITSRY---- -----TDLQP -VGMGAFGLV ----CSAKDQ L--------- --TGQAVAVK KIMKPFSTP- ----------
    507853                                                             MAEF VRAQIFGTTF EITSRY---- -----TDLQP -VGMGAFGLV ----CSAKDQ L--------- --TGQAVAVK KIMKPFSTP- ----------
    338747                                                             MAEF VRAQIFGTTF EITSRY---- -----TDLQP -VGMGAFGLV ----CSAKDQ L--------- --TSQAVAIK KIMKPFSTP- ----------
     49060                                                             MAEF VRAQIFGTTF EITSRY---- -----TDLQP -VGMGAFGLV ----CSAKDQ L--------- --TGQAVAVK KIMKPFSTP- ----------
    147551                                                             MAEF VRAQIFGTTF EITSRY---- -----TDLQP -VGMGAFGLV ----CSAKDQ L--------- --TGQAVAVK KIMKPFSTP- ----------
    286873                                                             MAEF VRAQIFGTTF EITSRY---- -----TDLQP -VGMGAFGLV ----CSAKDQ L--------- --TSQAVAIK KIMKPFSTP- ----------
    193904                                                             MAEF VRAQIFGTTF EITSRY---- -----TDLQP -VGMGAFGLV ----CSAKDQ L--------- --TSQAVAVK KIMKPFSTP- ----------
    189288                                                             MAEF VRAQIFGTTF EITSRY---- -----TDLQP -VGMGAFGLV ----CSAKDQ L--------- --TGQSVAVK KIMKPFSTP- ----------
    198339                                                             MAEF VRAQIFGTTF EITSRY---- -----TDLQP -VGMGAFGLV ----CSAKDQ L--------- --TSQPVAVK KIMKPFSTP- ----------
   2140409                                                             MAEF VRAQIFGTTF EITSRY---- -----TDLQP -VGMGAFGLV ----CSAKDQ L--------- --TNQNVAVK KIMKPFSTP- ----------
    642177                                                             MAEF VRAQIFGTTF EITSRY---- -----SDLQP -VGMGAFGLV ----CSAKDQ L--------- --TNQNVAIK KIMKPFSTP- ----------
    498471                                                             MAEF VRAQIFGTTF EITSRY---- -----SDLQP -VGMGAFGLV ----CSAKDQ L--------- --TNQSVAIK KIMKPFSTP- ----------
    784216                                                             MAEF VRAQIFGTTF EITSRY---- -----SDLQP -VGMGAFGLV ----CSAKDQ L--------- --TNQNVAIK KIMKPFSTP- ----------
    128268                                                             MAEF IRAQIFGTTF EITSRY---- -----SDLQP -VGMGAFGLV ----CSAKDQ L--------- --TNQNVAIK KIMKPFSTP- ----------
    246689                                                             MAEF IRAQIFGTTF EITSRY---- -----SDLQP -VGMGAFGLV ----CSARDQ L--------- --TNQNVAVK KIMKPFSTP- ----------
   1081594                                                             MAEF VRAQIFGTTF EITSRY---- -----SDLQP -VGMGAFGLV ----CSARDQ L--------- --TNQNVAVK KIMKPFSTP- ----------
```

```
 603877                                                              MAEF VRAQIFGTTF EITSRYAGPG AGGVYSDLQP -VGMGAFGLV ----CSAKDN L--------- --TGQNVAVK KIMKPFSTP- ----------
 140829                                                              MAEF VRAQIFGTTF EITSRY---- -----SDLQP -VGMGAFGLV WRVSSSAKDQ L--------- --TGQAVAVK KIMKPFSTP- ----------
 327107                                                              MAEF VRAQIFGTTF EITSRY---- -----TELQP -VGMGAFGLV ----CSAKDQ L--------- --TAQPVAVK KIMKPFSTP- ----------
 329694                                                              MAEF VRAQIFGTTF EITSRY---- -----TELQP -VGMGAFGLV ----CSAKDQ L--------- --TAQPVAVK KIMKPFSTP- ----------
 393439                                                              MAEF VRAQIFGTTF EITSRY---- -----TELQP -VGMGAFGLV ----CSAKDQ L--------- --TAQPVAVK KIMKPFSTP- ----------
  38942                                                              MAEF VRAQIFGTTF EITTRY---- -----TDLQP -VGMGAFGLV ----CSARDT L--------- --TGQPVAVK KIMKPFSTP- ----------
  87588                                                              MAEF IRAQIFGTCF EITSRY---- -----VDLQP -VGMGAFGLV ----CSAKDQ L--------- --TNQPVAIK KIMKPFSTP- ----------
 443298                                                              MAEF VRAQIFGTTF EITSRY---- -----SDLQP -VGMGAFGLV ----CSARDQ L--------- --TSQNVAIK KIMKPFSTP- ----------
  53587                                                              MAEF VRAQIFGTTF EITSRY---- -----TDLQP -VGMGAFGLV ----CSAKDQ L--------- --TGQAVAVK KIMKPFSTP- ----------
  70072                                                              MVDF VKLSIFGTVF EVTTRY---- -----VDLQP -VGMGAFGLV ----CSAKDQ L--------- --TNTSVAIK KIMKPFSTP- ----------
  13822                                                              MAEF VRAQIFGTTF EITSRY---- -----TDLQP -VGMGAFGLV ----CSAKDQ L--------- --TGHAVAIK KIMKPFGTP- ----------
  25364                                                              MADF IKLSIFGTVF EVTTRY---- -----VDLQP -VGMGAFGLV ----CSAKDQ L--------- --TGTNVAIK KIMKPFSTP- ----------
 116576                                                                         MGVL TILDRY---- -----TDLQP -VGMGAFGLV ----CSAKDQ L--------- --TSQPVAVK KIMKPFSTP- ----------
 103311                                                           MTSAGEF TRTQIFGTVF EITSRY---- -----TDLNP -VGMGAFGLV ----CSAMDK L--------- --TNQNVAIK KIMKPFSTS- ----------
  26173                                                           MSSDGEF TRTQIFGTVF EITNRY---- -----TDLNP -VGMGAFGLV ----CSAVDK L--------- --TGQNVAVK KIMKPFLTS- ----------
  55352                                                           MSSDGEF TRTQIFGTVF EITNRY---- -----TDLNP -VGMGAFGLV ----CSAVDK L--------- --TGQNVAVK KIMKPFSTA- ----------
 125207                                                           MTTNEEF IRTQIFGTVF EITNRY---- -----NDLNP -VGMGAFGLV ----CSATDT L--------- --TSQPVAIK KIMKPFSTA- ----------
  67307                                                           MTTNEEF IRTQIFGTVF EITNRY---- -----NDLNP -VGMGAFGLV ----CSATDT L--------- --TSQPVAIK KIMKPFSTA- ----------
 185764                                                            MSQEEF TRTQIFGTVF EITNRY---- -----GDLNP -VGMGAFGLV ----CSAKDK L--------- --TNQNVAIK KILKPFSTP- ----------
  99366                                                            MSQEEF TRTQIFGTVF EITNRY---- -----TDLNP -VGMGAFGLV ----CSAKDK L--------- --TNQNVAIK KIMKPFSTP- ----------
  50111                                                              MADF VNTSIFGTLF QITSRY---- -----DSLEP -VGMDS---- -----SARDQ L--------- --TNNSVAIK KIMKPFSTP- ----------
  87970                                                              MADF AKLSIFGTVF EVTTRY---- -----SDLQP -VGMGAFGLV ----CSASDQ L--------- --TATNVAIK KIMKPFSTP- ----------
 107532                  MSED AGVTSLPSGS APPIGNSISP GGPGFVMADF AKLSIFGTVF EVTTRY---- -----SDLQP -VGMGAFGLV ----CSASDQ L--------- --TATNVAIK KIMKPFSTP- ----------
 604754                                                                     MSISSRLE WVSLSSSSSS LWRLADGTGP WIVLGAFGLV ----CSAKDQ L--------- --TGAAVAVK KIMKPFSTP- ----------
 633130                                                               MSF VKLSIFGTSF EVTTRYVDLQ PVGMADGTGP WIVLGAFGLV ----CSAKDQ L--------- --TGAAVAVK KIMKPFSTP- ----------
 181927                                                              MPRF TRTQIFGTVF EITTRY---- -----VDLHP -VGMGASGLV ----CSAEDV L--------- --TGQSVAIK KVMKPFSTP- ----------
  69161                                                          MSQDDSSF VKLSVFGNVF QVTTRY---- -----TDLQP -IGMGAFGLL ----CSSIDQ K--------- --TSGPVAIK KVMKPFSAA- ----------
  35549                                                                                                       MGAFGLV ----CSARDQ L--------- --TGQPVAVK KIMKPFSTP- ----------
 230139                                                                           MW RPVSHE---- -----SDVSR -EATLTRLSN ----SSAKDQ L--------- --TAQPVAVK KIMKPFSTP- ----------
 193325                                                                                                       MGAFGLV ----CSAKDQ L--------- --TAQPVAVK KIMKPFSTP- ----------
 430049                                                                                                       MGAFGLV ----CSARDQ L--------- --TGQPVAVK KIMKPFSTP- ----------
 479652                                                              MAEF VRSQIFGTTF EITSRY---- -----TDLQP -VGMGAFGLV ----CSARDQ L--------- --TGQPVAVK KIMKPFSTP- ----------
 986652                                                                                MAEF---- -----VRAQI -FGTTFEITS ----SSAKDQ L--------- --TNQAVAVK KIMKPFSTP- ----------
 295607                                                                                   M---- -----FRPLP -LSPSASNKP ----SSAKDQ L--------- --TNQAVAIK KIMKPFSTP- ----------
 114099                                                                                                       MGAFGLV ----CSAKDQ L--------- --TSQAVAIK KIMKPFSTP- ----------
 265170                                                              MAEF VKLSIFGTVF EVTT------ ---------- ---------- ----SSAKDQ L--------- --TGTNVAVK KIMKPFSTP- ----------
  41804         MW LAYLRPTNPQ SLRGVKRPRA KDYTWKISGG PDASTYKTQH YITIYNMADF IRTQIFGTCF EITSRY---- -----VDLNP -VGMGAFGSV ----CSAKDQ L--------- --TGQNVAIK KIMKPFSTP- ----------
 500846                                                  MTTQV CSSAQLEMSF VKLSIFGTSF EVTTRY---- -----VDLQP -VGMGAFGLV ----CSAKDQ L--------- --TGSSVAIK KIMKPFSTP- ----------
 341622                                                                                                       MGAFGLV ----CSAKDQ L--------- --TNQAVAIK KIMKPFCTP- ----------
1545705                                                               MSF VKLSIFGTSF EVTTRY---- -----VDLQP -VGMGAFGLV ----CSAKDQ L--------- --TGSSVAIK KIMKPFSTP- ----------
  15276                                M LRYPAFVPGF SRTSAWTTTT ENACTSTMSF VKLSIFGTSF EVTTRY---- -----VDLQP -VGMGAFGLV ----CSAKDQ L--------- --TGSSVAIK KIMKPFSTP- ----------
 391543                                                              MAQF SQKTVMGTIF ETTQRY---- -----T-DWK PIGLGVSGLV ----CSARDQ I--------- --RHQTVAVK KLAEPFKTE- ----------
 346451                                               MMPTTQLT PPQGEGMAQF SQKTVMGTIF ETTQRL---- -----DLPQP PIGLGVSGLV ----CSARDQ I--------- --RHQTVAVK KLAEPFKTE- ----------
 449365                                        MSKS FTISQSSDNA PTPGQEMAQF TQKNIMGTLF ETTERY---- -----Q-HLE PVGMGISGLV ----CSARDQ I--------- --NHQTVAVK KLCEPFKTA- ----------
  54661 RAALSCSVHS AISDPDPPTN FPPPKAVTPS PQPPLLDLVY RDAQRGGRRK ENVATTQPTW SRCNLARVRF VVAP------ -----PSWRN SYGPRSLG-- -----PPSRS P--------- --PGTGPRAH RLMEDDRLT- ----------
 275309                                                              MVSF VGTNVMGASF EITDRY---- -----SDLEP -RGCGSSGMI ----CSARDS I--------- --SNSTVAIK KIISPFDTP- ----------
 389883                                                              MATF IKREIMGTTL ETTNRY---- -----SDLEA -RGVGVSGVI ----CSAKDC I--------- --TNQTVAIK KIARPFAST- ----------
  61624                                                                                                     MRNDRSTPY ----SSAEDQ F--------- --SGQRVAVK KIKKAFSSS- ----------
 173235                                                              MHKF MRAQIFGTTF ETTSRY---- -----SDLQP -VGTGTFSLV ----CSAEDQ F--------- --SGQRVAVK KIKKAFSSS- ----------
  35612                                                                                                                                                  MKPFSTP- ----------
  35597
 391476                                                            MLDVDL VKHNVGGCTF DTPSRY---- -----SNIQL -QGFGAYGIV ----CSLKDN L--------- --TSEVVAIK KIPDPFRSE- ----------
 335269                                                  MSAGV LSIPLVGVCA SHDTIHGLKA DS-KRY---- -----SNIQL -QGFGAYGIV ----CSLKDN L--------- --TSEVVAIK KIPDPFRSE- ----------
 377162                                         MS ILSSIMSAGV LSIPLVGVCA SHDTIHGLKA DS-KRY---- -----SNIQL -QGFGAYGIV ----CSLKDN L--------- --TSEVVAIK KIPDPFRSE- ----------
 271149                                                                                                                                                  MRMLSPS- ----------
  89784
 114108                                                                                                                                                 MLGKGPAH- ----------
 173207                                     MSRHYS VAIDGGP-EV FTIDTRY--R IVRKVGSGSY GTVCSAFDLH ADR------- ---------- ---------- ---------- -----YCAIK KVYRIFDK-- ----------
 127887                                  MTHDDTHCT ISLEGGR-ET FTVEKKY--K IIRRVGSGSY GTVCSAIELA TNDGNSLVAL SCHFYSNSPL K--------- ---------- -----VVAIK KCFRIFDK-- ----------
  29226                                  MVISDQYYS VAIEGGQ-ET FTIDKKY--R IIRKVGSGSY GTVCSAIHVE TNE------- ---------- ---------- ---------- -----VVAIK KCLRIFDR-- ----------
 167765 AQDFLFYFRI ISYYNSQ--- ---------- -QLLFKPVNN VSIDNGP-EK FTINKQY--K VIRKIGSGSY GSVCSALDVQ TNE------- ---------- ---------- ---------- -----VVAIK KCRHVFDK-- ----------
 150384                                  MTLSNDYYT VSIDNGP-EK FTINKQY--K IIRKIGSGSY GSVCSALDVQ TNE------- ---------- ---------- ---------- -----VVAIK KCRHVFDK-- ----------
  57923                                  MINPNDFYT VTIDSSNNET FTIHKQY--K IIRKVGAGSY GSVCSALNVN TNE------- ---------- ---------- ---------- -----VVAIK KCRHIFDK-- ----------
  57928
 230955 KSFNRFLFFL FSIQLGR--- ---------- -M----RYHE VEIDRGK-DR FTIRNKY--R FVRKIGSGAY GTVCSVVNIY TGD------- ---------- ---------- ---------- -----VIAIK KVHHIFDR-- ----------
 172033                                  M----RYHE VEIDRGK-DR FTIRNKY--R FVRKIGSGAY GTVCSVVNIY TGD------- ---------- ---------- ---------- -----VIAIK KVHHIFDR-- ----------
  40306                                     MIQIER HTYRVLNQDF -TIDRRF--Q VTKELGQGAY GIV------- ---------- ---------- ----CAARYT ES-------- -NQAVGVAIK KVTNIFSK-- ----------
 693957 ARLLSSCLSC RPARTA---- ---------- -ASMSDLHDR KVFKVFNQEF -IVDQRY--N VTKELGQGAY GIV------- ---------- ---------- ----CAATNN QT-------- -GE--GVAIK KITNVFSK-- ----------
 508525 PPLCTSTGAN APL--P---- ---------- -GSLRVRVQS RWLKVPDR-- --CADSG--N V--ELGQGAY GIV------- ---------- ---------- ----CAATNN QT-------- -GE--GVAIK KVTNVFSK-- ----------
 471145                                    MSGLDGR KVFKVFNQEF -IVDPRY--N VTKELGQGAY GIV------- ---------- ---------- ----CAATNN QT-------- -GE--GVAIK KVTNVFSK-- ----------
 480536                                         ML PRVRVQSRWL -KVPDRCADS GNVELGQGAY GIV------- ---------- ---------- ----CAATNN QT-------- -GE--GVAIK KVTNVFSK-- ----------
 471745                                    MSDLANR KVFKVFNQEF -IVDERY--N VTKELGQGAY GIV------- ---------- ---------- ----CAATNN QT-------- -GE--GVAIK KVTNVFSK-- ----------
 687176                                    MSELQGR KVFKVFNQEF -IVDERY--N VTKELGQGAY GIV------- ---------- ---------- ----CAATNN QT-------- -GE--GVAIK KVTNVFSK-- ----------
  34863                                  MGD--LQGR KVFKVFNQDF -IVDERY--T VTKELGQGAY GIV------- ---------- ---------- ----CAATNN QT-------- -GE--GVAIK KVTNVFSK-- ----------
  57153                                  MGDPSLQGR KVFKVFNQDF -IVDERY--T VTKELGQGAY GIV------- ---------- ---------- ----CAATNN QT-------- -GE--GVAIK KVTNVFSK-- ----------
 147961                                  MGD--LNGR KTFKVFNQEF -IVDERY--T VTKELGQGAY GIV------- ---------- ---------- ----CAATNA QT-------- -GE--GVAIK KITNVFSK-- ----------
 287354                                  MAD--LSGR KVFRVFNQDF -IVDERY--T VTKELGQGAY GIV------- ---------- ---------- ----CAATNA GT-------- -GE--GVAIK KVTNVFSK-- ----------
 281614                                  MTDPSLVGR KVFKVFNQDF -IVDERY--T VTKELGQGAY GIV------- ---------- ---------- ----CAASNN QT-------- -GE--GVAIK KVTNIFSK-- ----------
  29684 VAEHEDHAYQ HVYQIA---- ---------- -VKMADLQGR KVFKVFNQDF -IVDERY--T VTKELGQGAY GIV------- ---------- ---------- ----CAAVNN QT-------- -NE--GVAIK KVTNVFSK-- ----------
 144400                                    MADLVGR KIFKVFNQDF -IVDERY--T VTKELGQGAY GIV------- ---------- ---------- ----CAAVNT QT-------- -NE--GVAIK KVTNVFSK-- ----------
 136175                                    MADLVGR KIFKVFNQDF -IVDERY--T VTKELGQGAY GIVWYPLVLL ATAHLHVPCH MLIILQADIL FFPNSAAVNT QT-------- -NE--GVAIK KVTNVFSK-- ----------
 126818                                    MADLVGR KIFKVFNQDF -IVDERY--T VTKELGQGAY GIVWYPLVLW AAAHLHVSCH ISMI-QANLP FLLNSAAVNT QT-------- -NE--GVAIK KVTNVFSK-- ----------
2138682                                    MADLQGR KIFKVFNQDF -IVDERY--T VTKELGQGAY GIV------- ---------- ---------- ----CAAVNN QT-------- -NE--GVAIK KVTNVFSK-- ----------
  48096                                    MADLQGR KIFKVFNQDF -IVDERY--T VTKELGQGAY GIV------- ---------- ---------- ----CAAVNN QT-------- -NE--GVAIK KVTNVFSK-- ----------
 179022                                    MADLQGR KIFKVFNQDF -IVDERY--T VTKELGQGAY GIV------- ---------- ---------- ----CAAVNN QT-------- -SE--GVAIK KVTNVFSK-- ----------
 778216                                    MSDLQGR KIFKVFNQDF -IVDERY--T VTKELGQGAY GIV------- ---------- ---------- ----CAAVNN HT-------- -NE--GVAIK KVTNVFSK-- ----------
 665550                                    MADLVGR KVFKVFNQDF -IVDERY--T VTKELGQGAY GIV------- ---------- ---------- ----CAATNG QT-------- -NE--GVAIK KVTNVFSK-- ----------
 190738                                    MADLQGR KVFKVFNQDF -IVDERY--T VTKELGQGAY GIV------- ---------- ---------- ----CAATNT QT-------- -QE--GVAIK KVTNVFSK-- ----------
1107049                                    MADLQGR KVFKVFNQDF -VVDERY--T VTKELGQGAY GIV------- ---------- ---------- ----CAAVNN QT-------- -NE--GVAIK KVTNVFSK-- ----------
 504269                                    MTDLRGR KVFKVFNQDF -IVDERY--N VSKELGQGAY GIV------- ---------- ---------- ----CAASNI HT-------- -GE--GVAIK KVTNVFSK-- ----------
 432134                                    MADLRGR KVFKVFNQEF -VVDERY--N VSKELGQGAY GIV------- ---------- ---------- ----CAASNI HT-------- -GE--GVAIK KVTNVFSK-- ----------
  59068                                    MGELQGR KVFKVFNQDF -IVDERY--T VTKELGQGAY GIV------- ---------- ---------- ----CAATNN IT-------- -GE--GVAIK KVTNVFSK-- ----------
 336185                                    MADLQGR KVFKVFNQDF -IVNEQY--N VTKELGQGAY -VE------- ---------- ---------- ----SAATNI QT-------- -GE--GVAVK KVTNVFSK-- ----------
 343155                                    MADLQGR KVFKVFNQDF -IVNEQY--N VTKELGQGAY GIV------- ---------- ---------- ----CAATNI QT-------- -GE--GVAVK KVTNVFSK-- ----------
 326648                                    MADLQGR KVFKVFNQDF -IVNEQY--N VTKELGQGAY GIV------- ---------- ---------- ----CAATNS QT-------- -GE--GVAIK KVTNVFSK-- ----------
 425863                                    MADLQGR KVFKVFNQDF -IVNEQY--N VTKELGQGAY GIVW------ --------CV CDGTDELNPT SFSSSAATNT QT-------- -GE--GVAIK KVTNVFSK-- ----------
 436998                                    MSDLQGR KVFKVFNQDF -VVDERY--N VTKELGQGAY GIV------- ---------- ---------- ----CAAVNT QT-------- -NE--GVAIK KVTNVFSK-- ----------
 257317                                    MADLQGR KLFKVFNQDF -IVDERY--N VTKELGQGAY GIV------- ---------- ---------- ----CAATNT QT-------- -GE--GVAIK KVTNVFSK-- ----------
 998070                                    MADLQGR KVFKVFNQDF -IVDERY--N VTKELGQGAY GIV------- ---------- ---------- ----CAATNN GT-------- -GE--GVAIK KVTNVFSK-- ----------
  49449                                    MSDLSGR KIFKVFNQEF -IVDERY--T VTKELGQGAY GIV------- ---------- ---------- ----CAAQNN TS-------- -NE--GVAIK KVTNVFSK-- ----------
 498377                                    MADLQGR KIFKVFNQDF -IVDERY--T VTKELGQGAY GIV------- ---------- ---------- ----CAATNN QT-------- -GE--GVAIK KVTNVFSK-- ----------
 184715                                                              MSVT MLARSWAKEH TVL------- ---------- ---------- --SGSAASNI HT-------- -GE--GVAIK KVTNVFSK-- ----------
  51223                                     MDLQGR KVFKVFNQDF -IVDERY--T VTKELGQGAY ---------- ---------- ---------- -GIVCGATNV IT-------- -RE--GVAVK KVTNVFSK-- ----------
  66669                                     MDLQGR KIFKVFNQDF -IVDERY--T VTKELGQGAY ---------- ---------- ---------- -GIVCGATNV IT-------- -RE--GVAIK KVTNVFSK-- ----------
 649898                M---- ---------- -ALDGSLVGR KSFKVFNQDF -VVDERY--T VTKELGQGAY ---------- ---------- ---------- -GIVCAATNS AN-------- -GE--GVAIK KVTNVFSK-- ----------
 253817                M---- ---------- -SDTRSQRSL DKVPTVSSGM -FVDSKF--P AGLSQGASIQ TI-------- ---------- ------KLTL GGESSAATNS AN-------- -GE--GVAIK KVTNVFSK-- ----------
 618068                                                                                                                MAFAAVNN HT-------- -NE--GVAIK KVTNVFSK-- ----------
 108847                                     MAQNGR LVNKVFNQDF -VIDKRF--K ITKELGHGAY GIVCSAQYDD G--------- ---------- ---------S GS-------- -PGGNNVAIK KITNIFSK-- ----------
  33629                MNKDH KDGSVA---- ---------- -ATPNIYYGR SVNKVFNQDF -IIDKRF--K IVKELGHGAY GIVCSAKYDD GSGNDDITTN NNDAAG---- --------NS SS-------- -SDGSYVAIK KITNIFSK-- ----------
  54715                  MSS KTDDTG---- ---------- -AAAPIYYGR TVNKVYNQEF -IIDRRF--K IVKELGHGAY GIVCSAKYDD GT-----STT TTDSSN---- --------NS SS-------- -SDGSYVAIK KITNIFSK-- ----------
  88920                                   MATNSGHH TVFKVFNQDF -VVHDRY--Q ITKELGQGAY GLVVAAQNLD ---------- ---------- ---------- ---------- -SPQTPIAIK KVTNVFSK-- ----------
  51479                                       MSDR HTYKVFNQDF -TIDKRF--Q ITKELGHGAY GIVCAAKYTG ---------- ---------- ---------- -S-------- -TDGPSVAIK KVTNILSK-- ----------
  86943                                    MDVNEQY HKFKVFHQDF -VIDKRF--Q LIKELGHGAY GIVCSAKYIE ---------- ---------- ---------- -T-------- -PDGGSVAIK KVTNIFSK-- ----------
 100835                                     MTEPER HSFNVFHQDF -TIDKRF--E VIKELGHGAY GIVCSARYSE ---------- ---------- ---------- -T-------- -PDV-TVAIK KVTNIFSK-- ----------
 319719                                    MADKIER HTFKVFNQDF -SVDKRF--Q LIKEIGHGAY GIVCSARFAE ---------- ---------- ---------- -A-------- -AEDTTVAIK KVTNVFSK-- ----------
 822494                                    MADKIER HTFKVFNQDF -SVDKRF--Q LIKEIGHGAY GIVCSARFAE ---------- ---------- ---------- -A-------- -AEDTTVAIK KVTNVFSK-- ----------
 161919                                       MPGY QSFPVLNQQF -IVDKKF--Q FIREMGQGAY GVV------- ---------- ---------- ----CAAKDC ET-------- -NE--QVAIK KVCRIFEK-- ----------
 158631                                     MSNSGY QAFPVLNQQF -IVDKKF--Q FIREMGQGAY GVV------- ---------- ---------- ----CAAKDT DS-------- -DE--QVAIK KVCRIFEK-- ----------
 115223                                       MTNY QAFPVLNQQF -IVDKKY--Q FIREMGQGAY GVV------- ---------- ---------- ----CAAKDT ST-------- -GE--QVAIK KVCRIFEK-- ----------
 344665                                        MPY HAFPVLNQQF -LVDTKF--E FIRELGQGAY GVV------- ---------- ---------- ----CAAKNT ET-------- -GE--QVAIK KVTKVFEK-- ----------
 178050                                       MTIY QSFPVLNQQF -VVDKKY--Q FIREMGQGSY GVV------- ---------- ---------- ----CAAKDS ET-------- -DE--QVAIK KVCRVFEK-- ----------
 234927                                       MATH HSFPVLNQQF -IVDKKY--S FIRELGQGAY GVV------- ---------- ---------- ----CAATNT QT-------- -NE--QVAIK KVTKIFEK-- ----------
 328916              MAL---- ---------- -CGKVLVLYM ILFCVLFLAF YLFFYVY--E FDSHIIKKNF -FF------- ---------- ---------- ----SAARNT QN-------- -GE--GVAIK KVTNVFSK-- ----------
 273327                                                   MNL DLFFYVY--E FDSHIIKKNF -FF------- ---------- ---------- ----SAARNT QN-------- -GE--GVAIK KVTNVFSK-- ----------
 338804                                        MGR HGFPVLNQQF -LVDEKY--V FLRELGQGAY GVV------- ---------- ---------- ----CAARNT QN-------- -GE--GVAIK KVTNVFSK-- ----------
 527701 SYRCPSDPHP LPKHTSPSTL ISLRL----- ----SGTMAR HSFTALNSTF -VVDAQY--R MVKELGQGAY GCV------- ---------- ---------- ----IAAVHS ST-------- -GE--GCAIK KITNINTK-- ----------
  81317 SERRRYMGGP LPSIPLCTAQ LVSL------ ----SETMQR HTFTALNSTF -IVDSEY--Q FVKELGQGAY GCV------- ---------- ---------- ----VAARHR RS-------- -GE--GCAIK KITNINNK-- ----------
 831329                                       MLQR HSFTALNSTF -VVDSEY--Q FVKELGQGAY GCV------- ---------- ---------- ----VAARHR RS-------- -GE--GCAIK KITNINTK-- ----------
 676624                         MS------ ----RQPISR HTFTALNSTF -IVDGEY--Q FVKELGQGAY GCV------- ---------- ---------- ----VAARHR RS-------- -GE--GCAIK KITNINTK-- ----------
 826941                                        MHR HSFSALNSTF -VVDSEY--Q FVKELGQGAY GCV------- ---------- ---------- ----VAAKHR RS-------- -GE--GCAIK KITNINTK-- ----------
  77377                                        MQR HTFTALNSTF -VVDSEY--Q FVKELGQGAY GCV------- ---------- ---------- ----VAARHR RS-------- -GE--GCAIK KITNINTK-- ----------
  59915                                        MQR HSFHALNSTF -VVDSEY--Q FVKELGQGAY GCV------- ---------- ---------- ----VAATHR RT-------- -GE--GCAIK KITNINTK-- ----------
 160641                                       MPER HTFQALNSTF -VVDSEY--Q FVKELGQGAY GCV------- ---------- ---------- ----VAAKHR RS-------- -GE--GCAIK KITNINTK-- ----------
```

```
    108839  SYRSRFTPFP RCTQSISLVY LVIHLSLTSY YQHPSGTMQR HSFQALNSTF -VVDSEY--Q FVKELGQGAY GCV------- ---------- ---------- ----VAAKHR RS-------- -GE--GCAIK KITNINTK-- ----------
   1536187                                        MQR HSFNALNSTF -VVDSEY--Q FVKELGQGAY GCV------- ---------- ---------- ----VAAKHR RS-------- -GE--GCAIK KITNINTK-- ----------
    281708                                        MQR HSFTALNSTF -IVDSEY--Q FVKELGQGAY GCV------- ---------- ---------- ----VAAKHR RS-------- -GE--GCAIK KITNINTK-- ----------
    173791                                        MQR HSFTALNSTF -IVDSEY--Q FVKELGQGAY GCV------- ---------- ---------- ----VAAKHR RS-------- -GE--GCAIK KITNINTK-- ----------
     82703                                       MSSR HSFTALNSTF -VVDSEY--Q FVKELGQGAY GCV------- ---------- ---------- ----VAAKHR RS-------- -GE--GCAIK KITNINTK-- ----------
    139538                                       MMSR HQFTALNSVF -IVDSEY--Q FVKELGQGAY GCV------- ---------- ---------- ----VAARHR RS-------- -GE--GCAIK KITNINTK-- ----------
    479859                                        MQR HTFQALNSTF -YVDSEY--Q FTKELGQGAY GCV------- ---------- ---------- ----VAAKHR RS-------- -GE--GCAIK KITNINTK-- ----------
    621303                                       MLQR HQFTALNSVF -IVDAEY--E FVKELGQGAY GCV------- ---------- ---------- ----VAARHR RS-------- -GE--GCAIK KITNINTK-- ----------
     38569     MDYGSAR GTVVAVKTDQ LVSSA----- ----LEPTDR HQFTALNSVF -IVDAEY--E FVKELGQGAY GCV------- ---------- ---------- ----VAARHR RS-------- -GE--GCAIK KITNINTK-- ----------
    242842                                     MAQVNR TSFQALNQTF -FIDSEY--Q FVKELGQGAY GCV------- ---------- ---------- ----VAAKHK RT-------- -GE--GCAIK KITSINTK-- ----------
   2481011                                        MQR HTFTALNSTF -VVDSEY--Q FVKELGQGAY GCV------- ---------- ---------- ----VAARHR RT-------- -GE--GCAIK KITNINTK-- ----------
    169913                                        MQR HSFQALNSTF -VVDSEY--Q FVKELGQGAY GCV------- ---------- ---------- ----VSAKHR RT-------- -GE--GCAIK KITNINTK-- ----------
     71742                                        MER RSFVALSSTF -VVDHEY--Q FVKELGQGAY GCV------- ---------- ---------- ----VAAKHA KT-------- -GE--SCAIK KITNINTK-- ----------
     66635  APVASSSSAN QPPKTS---- ---------- ---------K HSFPVLGNDF -WVEREY--E FVKELGQGAY GIV------- ---------- ---------- ----CAAKNS RT-------- -GE--AVAIK KITKVFTK-- ----------
     53870  APVASSSSAN QPPKTS---- ---------- ---------K HSFPVLGNDF -WVEREY--E FVKELGQGAY GIV------- ---------- ---------- ----CAAKNS RT-------- -GE--AVAIK KITKVFTK-- ----------
     27246  TWMYISLFGA SILPSP---- ---------- ---TSTMLQR HSFTALNSTF -IVDSEY--Q FVKELGQGAY GCV------- ---------- ---------- ----VAARHR RS-------- -GE--GCAIK KITNINTK-- ----------
     88978  TFMTMGTFTR FWPTAS---- ---------- ---KVTVVGE PKYGRIISTF -VVDHEY--Q FVKELGQGAY GCV------- ---------- ---------- ----VAAKHA KT-------- -GE--SCAIK KITNINTK-- --------VG
   1091096  GRVGKETCAI TTIPPSFLPL PHLPPHRQHI LLAVTPSMQR HTFTALNSTF -IVDSEY--Q YVKELGQGAY GCV------- ---------- ---------- ----VAARHR RS-------- -GE--GCAIK KITNINTK-- ----------
     66856  GLIGD----V WKIPRS---- -----EERH- -----PAMQR HSFTALNSTF -VVDSEY--Q FVKELGQGAY GCV------- ---------- ---------- ----VAAKHR RS-------- -GE--G---- ---------- ----------
     20720  GGI----CSP PAGPES---- ----PRYRP- -----TMSSR HSFTALNSTF -VVDSEY--Q FVKELGQGAY GCV------- ---------- ---------- ----VAAKHR RS-------- -GE--GPSAH ARETTSAI-- ----------
    124539  PAIANTNDSP SKDGVS---- ---------- ---AKKLPRR HSFHALNSTF -VVDSEY--Q FVKELGQG-- ---------- ---------- ---------- ---------- ---------- --E--GCAIK KITNINTK-- ----------
    361187  PALIESRFAS SPPAAM---- ---------- ----ADLAGR KVFKVFNQDF -VVDERY--T VTKELGQGAY GIV------- ---------- ---------- ----CAAVNN QT-------- -NE--GVAIK KVTNVFSK-- ----------
    327531             MAN---- ---------- -MHAFDAQR- HAFKALQQTF -VVDQDY--T FHKELGQGAY GLV------- ---------- ---------- ----VAAKNR KT-------- -GE--GCAIK KITNIGTK-- ----------
      7086  TRLDDRNRQR DEQLKN---- ---------- -KSNKEKTRL NEEDPSLASG -VTYQDY--T FHKELGQGAY GLV------- ---------- ---------- ----VAAKNR KT-------- -GE--GCAIK KITNIGTK-- ----------
     28915  SADTKKNNFP TPPTMP---- ---------- ---------T HSFPVLNQQF -IVDTKY--Q FIREIGQGAY GVV------- ---------- ---------- ----CAAKNT QT-------- -GE--DVAIK KVTKVFEK-- ----------
    669145                                        MQR RTFTALNSTF -VVDADY--E FVKELGQGAY GCV------- ---------- ---------- ----VAAKHR KT-------- -GE--TVAIK KITNINTK-- --------VW
     46685                                 MD-LAAPRR HAFPVLNDQF -FVDEDY--E FIKELGQGAY GVV------- ---------- ---------- ----CAAKSK RT-------- -GE--SVAIK KVTKIFTK-- ----------
     53954                                 MDPIHNIPR HAFPVLSEPF -LVDETY--D FVKELGQGAY GVV------- ---------- ---------- ----CSARNK VS-------- -GD--QVAIK KVTKVFQK-- ----------
   1446322  ESVIDGILAS TPCWLR---- ---------- ----KFRMQR HSFTAINSTF -VVDSEY--L FVKELGQGAY GCV------- ---------- ---------- ----VAAKHR KS-------- -GE--GCAIK KITNINTK-- ----------
    351754                                      MMQQR HQFVALNSTF -IVDSEY--E FVKELGQGAY GCV------- ---------- ---------- ----VAARHR RS-------- -GE--GCAIK KITNINTK-- ----------
     65873  LVIIRYVTST VTVKMS---- ---------- -----QLQPR KHFQAFGTTY -AVQEGY--S LHKELGQGAY GCV------- ---------- ---------- ----VAGKHE AS-------- -GT--KLAIK KLRGVF---- ----------
     52469             MNAYPS---- ---------- -----QLQPR KHFQAFGTTY -AVQEGY--S LHKELGQGAY GCV------- ---------- ---------- ----VAGKHE AS-------- -GT--KLAIK KLRG------ ----------
     30002                 MS---- ---------- -----QLQPR KHFQAFGTTY -AVQEGY--S LHKELGQGAY GCV------- ---------- ---------- ----VAGKHE AS-------- ------VAIK KLRGVLCE-- --------IF
    154156                                        MGY HALSCFGQTF QVAEKY---E LVKLIGQGAY GIV------- ---------- ---------- ----CAAVNT RN-------- -GE--KCAIK KVNKLFER-- ----------
    166841                            MR SNLQQNSMGY HALSCFGQTF QVAEKY---E LVKLIGQGAY GIV------- ---------- ---------- ----CAAVNT RN-------- -GE--KCAIK KVNKLFER-- ----------
    238118                                        MKY HSLPCYGQSF EVYEKY---A FEREIGKGAY GIV------- ---------- ---------- ----CAAVNT KT-------- -GV--SCAIK KINKLFER-- ----------
    143069                                                  MKN---M LLKEKSERVH -TA------- ---------- ---------- ----YAAVNT KT-------- -GV--SCAIK KINKLFER-- ----------
    456995                                                                                                      MDVLSP PV-------- -TADQARVVR SRKSPTST-- ----------
    175880                                                                                                      MDASSP RN-------- -INAPAKDVR LRRLLAST-- ----------
     28309                                MATDTER CIFRAFGQDF -ILNKHF--H L--------- ---------- ---------- ---------- ---------T ES-------- -NEETHVAIR KIPNAFGN-- ----------
    767958                                MATDTER CIFRAFGQDF -ILNKHF--H LTGKIGRGSH SLI------- ---------- ---------- ----CSSTYT ES-------- -NEETHVAIR KIPNAFGN-- ----------
    126152                                      MTFFSDGY -CLFLEK--T KEGKIGRGSH SLI------- ---------- ---------- ----CSSTYT ES-------- -NEETHVAIR KIPNAFGN-- ----------
    613191
    198058                                                                                                                                MRGSPSK-- ----------
    123006
    178993                                                                                                           MA-------- -SSAPPSTTR PARASPSR-- ----------
    122967
    120501
     99018
    213678
    460523
    625621
    265871
     53464
    105414
    291876
     50744
    863837
     81355
    152896
   1196002
   1195972
     49917                                 MNQKRNDVD IKRTSFPAFG FLVGSRY--E IQEVLGHGTY GMV------- ---------- ---------- ----CSAIDK VE-------- -PSSPAIAIK RINNITRD-- ----------
    116823  KPSNILVNEN CDLKICDFGL ARIQDP---- ---------- ----QMTGYV STRYYRAPEI MLTW-QKYDV EV-------D IWSAGCIFAE MLEGKPLFPG K--------- ---------- DHVNQFSIIT ELLGTPPDD- ----------
    271167  KPSNILVNEN CDLKICDFGL ARIQDP---- ---------- ----QMTGYV STRYYRAPEI MLTW-QKYDV EV-------D IWSAGCIFAE MLEGKPLFPG K--------- ---------- DHVNQFSIIT ELLGTPPDD- ----------
    151286  ---------- ----ICDFGL ARIQDP---- ---------- ----QMTGYV STRYYRAPEI MLTW-QKYDV EV-------D IWSAGCIFAE MLEGKPLFPG K--------- ---------- DHVDQFSIIT ELLGTPPDD- ----------
     97820  KPSNILVNEN CDLKICDFGL ARIQDP---- ---------- ----QMTGYV STRYYRAPEI MLTW-QKYDV EV-------D IWSAGCIFAE MLEGKPLFPG K--------- ---------- DHVNQFSIIT ELLGTPPDD- ----------
    167647  KPSNILVNEN CDLKICDFGL ARIQDP---- ---------- ----QMTGYV STRYYRAPEI MLTW-QKYDV EV-------D IWSAGCIFAE MLEGKPLFPG K--------- ---------- DHVNQFSIIT ELLGTPPDD- ----------
    430394  KPSNILVNEN CDLKICDFGL ARIQDP---- ---------- ----QMTGYV STRYYRAPEI MLTW-QKYDV EV-------D IWSAGCIFAE MLEGKPLFPG K--------- ---------- DHVNQFSIIT ELLGTPPDD- ----------
    266211  KPSNILVNEN CDLKICDFGL ARIQDP---- ---------- ----QMTGYV STRYYRAPEI MLTW-QKYDV EV-------D IWSAGCIFAE MLEGKPLFPG K--------- ---------- DHVNQFSIIT ELLGTPPDD- ----------
    266223  KPSNILVNEN CDLKICDFGL ARIQDP---- ---------- ----QMTGYV STRYYRAPEI MLTW-QKYDV EV-------D IWSAGCIFAE MLEARIRCAS S--------- ---------- SRSP--GVRG NRLKTSSRT- ----------
    266189  KPSNILVNEN CDLKICDFGL ARIQDP---- ---------- ----QMTGYV STRYYRAPEI MLTW-QKYDV EV-------D IWSAGCIFAE MLEARIRCAS S--------- ---------- SRSP--GVRG NRLKTSSRT- ----------
    151328  KPSNILINEN CDLKICDFGL ARIQDP---- ---------- ----QMTGYV STRYYRAPEI MLTW-QKYDV EV-------D IWSAGCIFAE MLEGKPLFPG K--------- ---------- DHVDQFSIIT ELLGTPPDD- ----------
    800193  KPGNLLVNAD CELKICDFGL SRGFNS---- ------VPDE NAT-HLTEYV ATRWYRAPEI MLAF-RRYNT AI-------D VWSIGCILAE LLMGKPLFKG K--------- -------DY- --VDQLNKIL DVLGTPEEH- ----------
     22041  KPGNLLVNAD CELKICDFGL SRGFNS---- ------VPDE NAT-HLTEYV ATRWYRAPEI MLAF-RRYNT AI-------D VWSIGCILAE LLMGKPLFKG K--------- -------DY- --VDQLNKIL DVLGTPEEH- ----------
     22014  KPGNLLVNAD CELKICDFGL SRGFNS---- ------VPDE NAT-HLTEYV ATRWYRAPEI MLAF-RRYNT AI-------D VWSIGCILAE LLMGKPLFKG K--------- -------DY- --VDQLNKIL DVLGTPEEH- ----------
     22020  KPGNLLVNAD CELKICDFGL SRGFNS---- ------VPDE NAT-HLTEYV ATRWYRAPEI MLAF-RRYNT AI-------D VWSIGCILAE LLMGKPLFKG K--------- -------DY- --VDQLNKIL DVLGTPEEH- ----------
    448385  KPGNLLVNSD CELKICDFGL SRGFTQ---- ------ISGE NAT-HLTEYV ATRWYRAPEI MLAF-DRYST AI-------D VWSIGCILAE LLLGKPLFKG K--------- -------DY- --VDQLNKIL DVLGTPEEA- ----------
    122008  KPGNLLVNAD CELKICDFGL SRGFEQ---- ------RPDE HATTNMTEYV ATRWYRAPEI MLAF-RQYDT AI-------D VWSIGCILAE LLLGRPLFKG K--------- -------DY- --VDQLNKIL DVLGTPEDP- ----------
    578295  KPGNLLVNAD CELKICDFGL SRGFDA---- ------APEE YAT-QMTEYV ATRWYRAPEI MLAF-RRYNT AI-------D VWSIGCILAE LLLGRPLFKG K--------- -------DY- --VDQLNKIL DVLGSPDES- ----------
    139503  KPGNLLVNSD CELKICDFGL SRGFES---- ------RPDE YVS-HMTEYV ATRWYRAPEI MLAF-KRYDT AI-------D VWSIGCILAE LLTGKPLFKG K--------- -------DY- --VDQLNKIL DVLGTPSEA- ----------
   2468658  KPGNLLVNSD CELKICDFGL SRGYDA---- ------NPDE NTSHHLTEYV ATRWYRAPEI MLAF-RRYNT AI-------D VWSIGCILAE LLLGKPLFKG K--------- -------EWA YSVDQLNKIL DVLGHPDEF- ----------
   2215386  KPGNLLVNSD CELKICDFGL SRGYDA---- ------NPDE NTSHHLTEYV ATRWYRAPEI MLAF-RRYNT AI-------D VWSIGCILAE LLLGKPLFKG K--------- -------DY- --VDQLNKIL DVLGSPDEF- ----------
     88816  KPGNLLVNSD CELKICDFGL ARGFDA---- ------VPDE NQATHMTEYV ATRWYRAPEI MLAF-RRYNT AI-------D VWSIGCILAE LIMGKPLFKG K--------- -------DY- --VDQLNKIL DIVGTPPDE- ----------
     88949  KPGNLLVNSD CELKICDFGL ARGFDA---- ------VPDE NQATHMTEYV ATRWYRAPEI MLAF-RRYNT AI-------D VWSIGCILAE LIMGKPLFKG K--------- -------DY- --VDQLNKIL DIVGTPPDE- ----------
     88917  KPGNLLVNSD CELKICDFGL ARGFDA---- ------VPDE NQATHMTEYV ATRWYRAPEI MLAF-RRYNT AI-------D VWSIGCILAE LIMGKPLFKG K--------- -------DY- --VDQLNKIL DIVGTPPDE- ----------
    206606  KPGNLLVNAD CELKICDLGL SRAFERS--- ---TMPNQGE ESVSHLTEYV ATRWYRAPEI MLSF-KRYGT AI-------D VWSIGCILAE LLGSKPIFKG K--------- -------DY- --VDQLNLIL KVLGTPEER- ----------
    123647  KPGNLLVNAD CELKICDFGL SRGFNA---- ------LPDE NPT-MMTEYV ATRWYRAPEI MLAY-RRYDT AV-------D IWAVGCIAAE LMLGKPLFKG K--------- -------DY- --VDQLNKIL DILGTPEDE- ----------
    143931  KPGNLLVNAD CELKICDFGL SRGFNA---- ------LPDE NPT-MMTEYV ATRWYRAPEI MLAY-RRYDT AV-------D IWAVGCIAAE LMLGKPLFKG K--------- -------EL- -NFDQLNKIL DILGTPEDE- ----------
    165009  KPGNLLVNAD CELKICDFGL SRGFNS---- ------SPDE NST-MMTEYV ATRWYRAPEI MLAF-KGYNT AI-------D VWSIGCIMAE LMLGKPLFKG K--------- -------DY- --VDQLNKLL EVLGTPSNE- ----------
    580315  KPGNLLVNAD CELKICDFGL SRGFNS---- ------APDE NPT-LMTEYV ATRWYRAPEI MLVY-KAYNT AI-------D VWSIGCIMAE LIMGKPLFKG K--------- -------DY- --VDQLNKIL DVVGTPDNE- ----------
    492840  KPGNLLVNAD CELKICDFGL SRGFNS---- ------APDE NPT-LMTEYV ATRWYRAPEI MLVY-KAYNT AI-------D VWSIGCIMAE LIMGKPLFKG K--------- -------EY- --HYLI--FI DHVGTPDNE- ----------
    594182  KPGNLLVNAD CELKICDFGL SRGFNS---- ------APDD NPT-LMTEYV ATRWYRAPEI MLVY-KAYNT PI-------D VWAIGCIMAE LLTGKPLFKG K--------- -------DY- --VDQLNKIL DVLGTPDDA- ----------
    168238  KPGNLLVNAD CELKICDFGL SRGFNS---- ------APDE NPT-LMTEYV ATRWYRAPEI MLVY-KAYNT AI-------D VWSIGCIMAE LMMGKPLFKG K--------- -------DY- --VDQLNKIL DVLGSPEDA- ----------
    107302  KPGNLLVNAD CELKICDFGL SRGFNS---- ------APDE NPT-LMTEYV ATRWYRAPEI MLVY-KAYNT AI-------D VWSIGCIMAE LMMGKPLFKG K--------- -------DY- --VDQLNKIL DVLGSPEDA- ----------
     64718  KPGNLLVNAD CELKICDFGL ARGFEH---- ------HPDG NQ-TQLTEYV ATRWYRAPEL MLAF-KQYNT AI-------D VWSIGCIFAE LLLGKPLFKG K--------- -----DYV-- ---DQLNKIL EVLGTPDDE- ----------
     64830  KPGNLLVNAD CELKICDFGL ARGFEH---- ------HPDG NQ-TQLTEYV ATRWYRAPEL MLAF-KQYNT AI-------D VWSIGCIFAE LLLGKPLFKG K--------- -----DYV-- ---DQLNKIL EVLGTPDDE- ----------
   1154823  KPGNLLVNAD CELKICDFGL ARGFEH---- ------HPDG NQ-TQLTEYV ATRWYRAPEL MLAF-NR--- -V-------D VWSIGCIFAE LLLGKPLFKG K--------- -----ESVSI LTVDQLNKIL EVLGTPDDE- ----------
    433002  KPGNLLVNSD CELKICDFGL SRGFDA---- ------VPDE HTSGHLTEYV ATRWYRAPEI MLAF-RGYTT AI-------D VWSIGCILAE LLLGRPLFKG K--------- ---------- DYVDQVNKIL DVLGTPEES- ----------
    822334  KPGNLLVNSD CELKICDFGL SRGFDA---- ------VPDE HTSGHLTEYV ATRWYRAPEI MLAF-RGYTT AI-------D VWSIGCILAE LLLGRPLFKG K--------- ---------- EYLDQVNKIL DVLGTPEES- ----------
    511572  KPGNLLVNSD CELKICDFGL SRGYDS---- ------APDE QV-GHLTEYV ATRWYRAPEI MLAF-RGYTR AI-------D VWSIGCILAE LMLGRPLFKG K--------- ---------- DYVDQLNKIL DVLGTPEED- ----------
    688421  KPGNLLVNSD CELKICDFGL SRGFDS---- ------TPDE HIGSQLTEYV ATRWYRAPEI MLSF-RRYTT AI-------D VWSIGCILAE LLLGRPLFKG K--------- ---------- EYVDQLNKIF EVLGTPEES- ----------
     17778  KPGNLLVNSD CELKICDFGL SRGFDS---- ------TPDE HIGSQLTEYV ATRWYRAPEI MLSF-RRYTT ASKPNLLFND AFSRNCYLGG RYLRGKIMVG D------LDP PSCTIDECYL PTVDQLNKIF EVLGTPEES- ----------
   1336823  KPGNLLVNSD CELKVCDFGL ANTYEG---- -------NIS SHLLGLSCLP ATRWYRAPEI MLSF-STYTT AI-------- SRSIGCILAE LILGKPLFKG K--------- -----KLVSF NYLDQLNRIL DVLGSPDET- ----------
    605623  KPGNLLVNSD CELKICDFGL SRGFDA---- -------QSE DGVSHMTEYV ATRWYRAPEI MLAF-RFVCI FI-------D VWSIGCILAE LLLGKPMFKG K--------- -------DY- --VDQLNKIL DFVGTPSDE- ----------
    511468  KPGNLLVNSD CELKICDFGL SRGYDS---- ------APDE QVG-HLTEYV ATRWYRAPEI MLAF-RGYTR AI-------D VWSIGCILAE LMLGRPLFK- ---------- -------- --VDQLNKIL DVLGTPEED- ----------
     60300  KPGNLLVNSD CELKICDFGL SRGFDA---- ------LPDD DTH-RLTEYV ATRWYRAPEI ILAF-RRYNT AI-------D VWSIGCILAE LLTGKPLFKG K--------- ------DY-- --VDQLNKIL DVLGTPDEA- ----------
   2435698  KPGNLLVNSD CELKICDFGL SRGYDA---- ------NPDE NTSHHLTEYV ATRWYRAPEI MLAF-RRYNT AI-------D VWSIGCILAE LLLGKPLFKG K--------- ------EWAY -SVDQLNKIL DVLGHPDEF- ----------
   1222436  KPGNLLVNSD CELKICDFGL SRGFDA---- ------VPEE HTGGNLTEYV ATRWYRAPEI MLSF-RRYTT AI-------D VWSIGCIFGE LLMGRPLFKG K--------- ------EY-- --VDQLNKIF DVLGTPEEN- ----------
   1172380  KPGNLLVNSD CELKICDFGL SRGFDS---- ------SPDE YAN-HLTEYV ATRWYRAPEI MLSF-RRYTT SI-------D VWSIGCILAE LLLGRPLFKG K--------- ------DY-- --VDQLNKIL EVLGTPEER- ----------
   1151825  KPGNLLVNSD CELKICDFGL SRGFDS---- ------SPDE YAN-HLTEYV ATRWYRAPEI MLSF-RRYTT SI-------D VWSIGCILAE LLLGRPLFKG K--------- ------DYGQ QNIDQLNKIL EVLGTPEER- ----------
    410058  KPGNLLVNSD CELKICDFGL ARGFDD---- ------TASE ENASQLTEYV ATRWYRAPEI MLSF-RRYTK AI-------D VWSLGCIFGE LLLGKPLFKG K--------- ------DY-- --VDQLNKIL DVLGTPDEK- ----------
     57461  KPGNLLVNSD CELKICDFGL SRGFES---- ------RPDE YVS-HMTEYV ATRWYRAPEI MLAF-KRYDT AI-------D VWSIGCILAE LLTGKPLFKG K--------- ------DY-- --VDQLNKIL DVLGTPSEA- ----------
    311323  KPGNLLVNAD CELKICDFGL ARGFSLD--- -----PEENA ---GYMTEYV ATRWYRAPEI MLSF-QSYTK A--------- ----SCILAE LLGGKPFFKG R--------- ---------- DYVDQLNQIL HFLGTPSEA- ----------
    143102  KPGNLLVNAD CELKICDFGL ARGYSDN--- -----PESSA ---GFMTEYV ATRWYRAPEI MLSF-QNYTK AI-------D MWSVGCIFAE MLGGKPLFKG R--------- ---------- DYVDQLNQIL GILGTPDEE- ----------
   2750129  KPGNLLVNAD CELKICDFGL ARGYSSG--- -----TGSAS NNQGFMTEYV ATRWYRAPEV MLSF-ANYTT AI-------D VWSIGCILAE LLGGKPIFKG R--------- ---------- DYVDQLNQIL HYLGTPSED- ----------
   2702932  KPGNLLVNAD CELKICDFGL ARGYSSG--- -----TGSAS NNQGFMTEYV ATRWYRAPEV MLSF-ANYTT AI-------D VWSIGCILAE LLGGKPIFKG R--------- ---------- DYVDQLNQIL HYLGTPSED- ----------
    108010  KPSNLLLNAN CDLKLCDFGL ARSARPP--- -----PNVAN DSSTFMTEYV ATRWYRAPEV MLTF-KEYTR AI-------D MWSVGCVLAE MLSGKPLFPG R--------- ---------- DYHHQLSIIL DILGTPSID- ----------
   1754091  KPSNLLLNAN CDLKICDFGL ARSARPP--- -----PNVAN DSSTFMTEYV ATRWYRAPEV MLTF-KEYTR AI-------D IWSVGCVLAE MLSGKPLFPG R--------- ---------- DYHHQLSIIL DVLGTPSLD- ----------
    564944  KPSNLLLNAN CDLKVADFGL ARSVKTA--- -----EPSGT ETG-FMTEYV ATRWYRAPEI MLTF-KQYTK AI-------D IWSVGCILAE MLSGKPLFPG R--------- ---------- DYHHQLTLIL DLLGTPTLD- ----------
  AtMPK18   KPKNILANAN CKLKVCDFGL ARVAFN---- --------DT PTTVFWTDYV ATRWYRAPEL CGSFFSKYTP AI-------D VWSIGCIFAE VLTGKPLFPG K--------- ---------- SVVHQLELIT DLLGTPKSE- ----------
  AtMPK19   KPKNILANAN CKLKVCDFGL ARVSFN---- --------DT PTTVFWTDYV ATRWYRAPEL CGSFCSKYTP AI-------D IWSIGCIFAE VLTGKPLFPG K--------- ---------- SVVHQLDLIT DLLGTPKSE- ----------
   AtMPK20  KPKNILANAN CKLKICDFGL ARVAFN---- --------DT PTTIFWTDYV ATRWYRAPEL CGSFYSKYTP AI-------D IWSIGCIFAE VLMGKPLFPG K--------- ---------- NVVHQLDLMT DLLGTPSLD- ----------
 OsMPK20-1  KPKNILANSN CKLKICDFGL ARVAFN---- --------DT PTTIFWTDYV ATRWYRAPEL CGSFFSKYTP AI-------D IWSIGCIFAE VLTGKPLFPG K--------- ---------- NVVHQLDLMT DLLGTPSMD- ----------
 OsMPK20-4  KPKNILANAN CKLKICDFGL ARVAFN---- --------DT PTTVFWTDYV ATRWYRAPEL CGSFFTKYSP AI-------D IWSIGCIFAE ILTGKPLFPG K--------- ---------- NVVHQLDLMT DLLGTPSMD- ----------
 OsMPK20-5  KPKNILANAN CKLKICDFGL ARVAFN---- --------DT PTTVFWTDYV ATRWYRAPEL CGSFFSKYSP AI-------D TWSIGCIFAE ILTGKPLFPG K--------- ---------- NVVHQLDLMT DLLGTPSMD- ----------
 OsMPK20-3  KPKNILANAD CKLKICDFGL ARVAFT---- --------DA PTTIFWTDYV ATRWYRAPEL CGSFYSKYTP AI-------D IWSIGCIFAE VLIGKPLFPG K--------- ---------- NVVHQLDLIT DLLGTPSLD- ----------
   AtMPK16  KPKNILANAD CKLKICDFGL ARVAFN---- --------DT PTAIFWTDYV ATRWYRAPEL CGSFFSKYTP AI-------D IWSIGCIFAE LLTGKPLFPG K--------- ---------- NVVHQLDLMT DMLGTPSAE- ----------
 OsMPK16-1  KPKNILANAD CKLKICDFGL ARVAFS---- --------DT PTAIFWTDYI ATRWYRAPEL CGSFFSKYTP AI-------D IWSIGCIFAE LLTGRPLFPG K--------- ---------- NVVHQLDIIT DLLGTPSPE- ----------
   AtMPK17  KPKNILANAD CKIKICDLGL ARVSFT---- --------DS PSAVFWTDYV ATRWYRAPEL CGSFYSNYTP AI-------D MWSVGCIFAE MLTGKPLFPG K--------- ---------- NVVHQLELVT DLLGTPSPI- ----------
    AtMPK8  KPKNILANAD CKLKICDFGL ARVSFN---- --------DA PTAIFWTDYV ATRWYRAPEL CGSFFSKYTP AI-------D IWSVGCIFAE MLLGKPLFPG K--------- ---------- NVVHQLDLMT DFLGTPPPE- ----------
   AtMPK15  KPKNILANAD CKLKICDFGL ARVSFN---- --------DA PTAIFWTDYV ATRWYRAPEL CGSFFSKYTP AI-------D IWSVGCIFAE MLLGKPLFPG K--------- ---------- NVVHQLDIMT DFLGTPPPE- ----------
 OsMPK17-1  KPKNILANSD CKLKICDFGL ARASFN---- --------DA PSAIFWTDYV ATRWYRAPEL CGSFFSKYTP AI-------D IWSIGCIFAE LLTGRPLFPG K--------- ---------- NVVHQLDIIT DLLGTPSSE- ----------
```

```
      AtMPK9  KPKNILANSD CKLKICDFGL ARVSFN---- --------DA PSAIFWTDYV ATRWYRAPEL CGSFFSKYTP AI-------D IWSIGCIFAE MLTGKPLFPG K--------- ---------- NVVHQLDIMT DLLGTPPPE- ----------
    OsMPK21-1 KPKNILANAD CKLKVCDFGL ARVSFN---- --------DT PSAIFWTDYV ATRWYRAPEL CGSFFSKYTP AI-------D IWSVGCIFAE LLTGKPLFPG K--------- ---------- NVVHQLDLMT DLLGTPSAE- ----------
    OsMPK21-2 KPKNILANAD CKVKICDFGL ARVSFD---- --------DT PSAIFWTDYV ATRWYRAPEL CGSFFSKYTP AI-------D IWSVGCIFAE MLMGKPLFPG K--------- ---------- NVVHQLDLMT DLLGSPSGE- ----------
    OsMPK17-2 KPKNILANSD CKLKICDFGL ARVAFN---- --------DS PSTIFWTDYV ATRWYRAPEL CGSFFSKYTP AI-------D IWSIGCIFAE ILTGRPLFPG R--------- ---------- NVVHQLDLIT DLLGTPSSE- ----------
        30260 KPGNLLVNSD CELKICDFGL SRGFDA---- ------VPDE HTSGHLTEYV ATRWYRAPEI MLAF-RGYTT AI-------D VWSIGCIFAE LLLGRPLFKG K--------- ---------- ---------- ---------- ----------
       130090 KPSNILVNSD CRIKICDFGL ARHAN----- --------DR DLAEGLSEYV VTRWYRAPEL LLA--NAYTK AI-------D MWSIGCIFAE LLGRRIMFPG T--------- ---------- SYVDQLKVIV DIVGTPTTF- ----------
       599215 KPGNLLVNAD CELKICDFGL SRGFNS---- ------APDE NPT-LMTEYV ATRWYRAPEI MLVY------ ---------- ---------- ---------- ---------- ---------- NYVDQLNKIL DVVGTPDNE- ----------
       219362 KPGNILVSSR GVLKICDFGL ARAISGS--- ----PSNRSE EP---ITNYV ATRWYRAPEL ILRS-THYGK EV-------D MWAVGCILGE LYGRQPLMPG K--------- ---------- SSVHQLHEII KYLGSPPPH- ----------
        56051 KPGNILVTTQ GILKICDFGL ARGINPK--- ----YFKNRS AT---ITNYV ATRWYRAPEL ILST-KNYTK AV-------D IWAAGCILGE LFGRRPLFPG K--------- ---------- NSNEQIHELF KILGNPPLD- ----------
       139809 KPGNILVTTQ GILKICDFGL ARGINPK--- ----YFKNRS AT---ITNYV ATRWYRAPEL ILST-KNYTK AV-------D IWAAGCILGE LFGRRPLFPG K--------- ---------- NSNEQIHELF KILGNPPLD- ----------
         1060 KPGNILVTIQ GTLKICDFGL ARGINTK--- ----FITKGS KAS-QITNYV ATRWYRAPEL ILSK-KNYSK AV-------D MWAVGCILAE LYGRRPLFAG N--------- ---------- DLVHQITEIV KVLGSPSKG- ----------
       184799 KPGNILVTIQ GTLKICDFGL ARGINTK--- ----FITKGS KAS-QITNYV ATRWYRAPEL ILSK-KNYSK AV-------D MWAVGCILAE LYGRRPLFAG N--------- ---------- DSVHQITEIV KVLGSPSKG- ----------
        68008 KPGNILVTIQ GTLKICDFGL ARGISKE--- ----FLKQ-- KPH-SITNYV ATRWYRAPEL LLSK-KTYSK SV-------D MWAVGCIFAE LYGRRPIFIG N--------- ---------- DQIHQATEIV KILGSPPRD- ----------
        99901 KPGNILVTIQ GTLKICDFGL ARGISKE--- ----FLKQ-- KPH-SITNYV ATRWYRAPEL LLSK-KTYSK SV-------D MWAVGCIFAE LYGRRPIFIG N--------- ---------- DQIHQATEIV KILGSPPRD- ----------
       104502 KPGNILVTIQ GTLKICDFGL ARGISKE--- ----FLKQ-- KPH-SITNYV ATRWYRAPEL LLSK-KTYSK SV-------D MWAVGCIFAE LYGRRPIFIG N--------- ---------- DQIHQATEIV KILGSPPRD- ----------
       645859 KPGNILCTLN GCLKICDFGL ARGIHAG--- ----FFKCHS TVQPHITNYV ATRWYRAPEL LLSN-QPYSK SV-------D IWAVGCILAE FYARKPVFMG R--------- ---------- DSMHQIFEII KVLGTPDKD- ----------
       599559 KPGNILCTLN GCLKICDFGL ARGIHAG--- ----FFKCHS TVQPHITNYV ATRWYRAPEL LLSN-QPYSK SV-------D IWAVGCILAE FYARKPVFMG R--------- ---------- DSMHQIFEII KVLGTPDKD- ----------
   36518:4-24 KPGNILVTTQ GTLKICDFGL ARGVNPR--- ----YFKHRS S---QITNYV ATRWYRAPEL MLSE-KRYTK AV-------D LWAVGCILAE LYGRKPLFPG K--------- ---------- NQMDQIHCIV RVLGTPLPA- ----------
       178963 RAQDLRLWPR PRLLGRPGGE CRLHDR---- ---------- ----VCRDAL VPRPRDHAEL PELHQI---- ---------D VWSVGCILAE LLGGRPFFKG R--------- ---------- DYVDQLNQIL HILGTPNEE- ----------
       196817 KPGNLLVNAD CELKICDFGL ARGFS----- ------VDPE ENAGYMTEYV ATRWYRAPEI MLSF-QSYTK AI-------D VWSVGCILAE LLGGRPFFKG R--------- ---------- DYVDQLNQIL HILGTPNEE- ----------
       738557 KPGNLLVNAD CELKICDFGL ARGFS----- ------MDPE ENAGYMTEYV ATRWYRAPEI MLSF-QSYTK AI-------D VWSVGCILAE LLGGKPFFKG R--------- ---------- DYVDQLNQIL HYLGTPNEE- ----------
      1159263 KPGNLLVNAD CELKICDFGL ARGYTPGGGA ------SRAA GNQGFMTEYV ATRWYRAPEI MLSF-ANYTT AI-------D VWSVGCILAE LLGGKPIFKG R--------- ---------- DYVDQLNQIL HYLGTPSED- ----------
       176895 KPGNLLVNAD CELKICDFGL ARGFTPGGGT ------SKSA GNQGFMTEYV ATRWYRAPEI MLSF-ANYTT AI-------D VWSVGCILAE LLGGKPIFKG R--------- ---------- DYVDQLNQIL HYLGTPSED- ----------
       664529 KPGNLLVNAD CELKICDFGL ARGYTPGGGA ------SKSA GNQGFMTEYV ATRWYRAPEI MLSF-ANYTT AI-------D VWSIGCILAE LLGGKPIYKG R--------- ---------- DYVDQLNQIL HYLGTPSED- ----------
      1194920 KPGNLLVNAD CELKICDFGL ARGYAPGPGG GTASANQKAA GNQGFMTEYV ATRWYRAPEI MLSF-ANYTT AI-------D VWSVGCILAE LLGGKPIFKG R--------- ---------- DYVDQLNQIL HYLGTPSED- ----------
       432994 KPGNLLVNSD CELKICDFGL SRGFDA---- ------VPDE HTSGHLTEYV ATRWYRAPEI MLAF-RGYTT AI-------D VWSIGCIFAE LLLGRPLFKG K--------- ---------- DYGRSMLGLY SDSGSEAMI- ----------
        98579 KPGNLLVNAD CELKICDFGL SRGFDA---- ------LPDD D-THRLTEYV ATRWYRAPEI ILAF-RRYNT AI-------D VWSIGCIFAE LLTGKPLFKG K--------- ---------- E
        88826 KPGNLLVNSD CELKICDFGL ARGFDA---- ------VPDE NQATHMTEYV ATRWYRAPEI MLAF-RRYNT AI-------D VWSIGCILAE LIMGKPLFKG K--------- ---------- DYG---LSFR FPSSPFMFL- ----------
        54157 KPSNLLLNAN CDLKVCDFGL ARSVRTA--- -----EQ--- ET-GFMTEYV ATRWYRAPEI MLTF-KQYTK AI-------D VWSVGCILAE MLSGRPLFPG R--------- ---------- DYHHQLTLIL DVLGTPTLD- ----------
       914084 KPSNLLLNAN CDLKVCDFGL ARSVKTA--- -----EPSGT ET-GFMTEYV ATRWYRAPEI MLTF-KQYTK AI-------D VWSVGCILAE MLSGKPLFPG R--------- ---------- DYHHQLTLIL DVLGTPTLD- ----------
       503422 KPSNLLLNAN CDLKVCDFGL ARSVKTA--- -----EPSGT ET-GFMTEYV ATRWYRAPEI MLTF-KQYTK AI-------D VWSVGCILAE MLSGKPLFPG R--------- ---------- DYHHQLTLIL DVLGTPTLD- ----------
        95205 KPANLLLNAN CDLKVCDFGL ARSTRST--- -----NPGGK EA-GLMTEYV ATRWYRAPEI MLSF-KMYTK AI-------D IWAVGCILAE LINARPLFPG R--------- ---------- DYGHQLDLIL DVIGTPTLE- ----------
       229298 KPANLLLNAN CDLKVCDFGL ARSVKST--- -----TSGGK EV-GLMTEYV ATRWYRAPEI MLSF-KMYTK AV-------D MWAVGCILAE LLTTRPLFPG R--------- ---------- DYSHQLDLIL DVIGTPTLD- ----------
       455126 KPANLLLNAN CDLKVADFGL ARSVRTG--- -----LSNGA ES-GLMTEYV ATRWYRAPEI MLSF-KRYTK AI-------D VWSVGCILGE MLTGRPMFPG R--------- ---------- DYGHQLDLIL DVLGTPPLD- ----------
       256867 KPANILLNQN CDLKVCDFGL ARSVRTG--- -----RPAG- EV-GLMTEYV ATRWYRAPEI MLSF-KMYTK AI-------D VWSVGCILAE MLTGRPLFPG R--------- ---------- DYGHQLDLIL DVIGTPTLE- ----------
       419576 KPSNLLLNAN CDLKICDFGL ARSSIPS--- -----QNQE- GQ-GFLTEYV ATRWYRAPEI MLTF-KEYTK AI-------D VWSVGTVLAE MLSGRPLFPG R--------- ---------- DYHHQLTLIL DVLGTPSLD- ----------
        74409 KPSNLLLNAN CDLKVCDFGL ARSA--A--- -----STDD- NS-GFMTEYV ATRWYRAPEI MLTF-KEYTK AI-------D VWSVGCILAE MLSGKPLFPG K--------- ---------- DYHHQLTLIL DVLGTPTME- ----------
       521809 KPSNLLLNAN CDLKVCDFGL ARSA--A--- -----STED- NS-GFMTEYV ATRWYRAPEI MLTF-KEYTK AI-------D VWSVGCILAE MLSGKPLFPG K--------- ---------- DYHHQLTLIL DVLGTPTME- ----------
        54281 KPSNLLLNAN CDLKICDFGL ARIDAAK--- -----EGNGT EKINLLTEYV ATRWYRAPEL MLTS-SQYSK AI-------D IWSVGCILAE LFLGQPLFPG R--------- ---------- DYKHQLILIF EILGTPIGD- ----------
        48349 KPSNLLLNAN CDLKICDFGL ARIDAAK--- -----EGNGT EKINLLTEYV ATRWYRAPEL MLTS-SQYSK AI-------D IWSVGCILAE LFLGQPLFPG R--------- ---------- DYKHQLILIF EILGTPIGD- ----------
       594110 KPGNLLVNAD CELKICDFGL SRGFNS---- -------APD DNPTMMTEYV ATRWYRAPEI MLVY-KAYNT PI-------D VWSVGCIMAE LLTGKPLFKG K--------- ---------- DYVDQLNKIL DVLGTPDDA- ----------
        27538 -PSNLLLNAN CDLKLCDFGL ARSARPP--- -----PNVAN DSSTFMTEYV ATRWYRAPEV MLTF-KEYTR AI-------D MWSVGCVLAE MLSGTPMFPG RDYTAKHSAD TSLQPLKLGQ SDHDQLSLIL DVLGTPSID- ----------
        96623 KPSNLLLNAN CDLKLCDFGL ARSARPP--- -----PNVAN DSSTFMTEYV ATRWYRAPEV MLTF-KEYTR AI-------D IWSVGCVLAE MLSGKPLFPG RDY------- ---------- --HHQLSIIL DVLGTPSLD- ----------
       380926 KPSNLLLNAN CDLKVCDFGL ARSAASQ--- ---------E DNSGFMTEYV ATRWYRAPEI MLTF-KEYTK AI-------D VWSVGCILAE MLSGKPLFPG KDY------- ---------- --HHQLTLIL DVLGTPTME- ----------
       263236 KPSNLLLNAN CDLKVCDFGL ARSAAST--- ---------D DNSGFMTEYV ATRWYRAPEI MLTF-KEYTK AI-------D VWSVGCILAE MLSGKPLFPG KDY------- ---------- --HHQLTLIL DVLGTPTME- ----------
       154671 KPSNLLLNAN CDLKVCDFGL ARSAAST--- ---------E DNQGFMTEYV ATRWYRAPEI MLTF-KEYTK AN-------- ----GRLLRH QIATCA
        67562 KPSNLLLNAN CDLKVCDFGL ARSAAST--- ---------E DNSGFMTEYV ATRWYRAPEI MLTF-KEYTK AI-------D VWSVGCILAE MLSGKPLFPG KDCM
       226444 KPSNLLLNAN CDLKVCDFGL ARSAAS---- --------TE DNSGFMTEYV ATRWYRAPEI MLTF-KEYTK AI-------D VWSVGCILAE MLSGKPLFPG KDCKCPR--- ---------- --FQLL
      1137209 KPSNLLVNAN CDLKVCDFGL ARSARPP--- -----PNVAN DSSTFMTEYV ATRWYRAPEV MLTF-KEYTR AI-------D MWSVGCVLAE MLSGKPLFPG RDCTIFI--- ---------- --LLLHTLHL TQLASPVSA- ----------
       AtMPK13 KPSNLVLNTN CDLKICDFGL ART------- ---------S NETEIMTEYV VTRWYRAPEL LLNS-SEYTG AI-------D IWSVGCIFME ILRRETLFPG KDY------- ---------- --VQQLKLIT EVSKLKP
       AtMPK11 KPSNLLLNAN CDLKIGDFGL ART------- ---------K SETDFMTEYV VTRWYRAPEL LLNC-SEYTA AI-------D IWSVGCILGE IMTREPLFPG RDY------- ---------- --VQQLRLIT EVNFSLFHL- ----------
       459393 KPQNLLIDKE GNLKLADFGL ARAFGV---- ---------- -PLRTYTHEV VTLWYRSPEI LLGS-RQYST GV-------D MWSVGCIFAE MCTRKPLFPG D--------- ---------- SEIDEIFKIF RILGTPSEQ- ----------
        69938 KPGNLLVNSD CELKICDFGL SRGFTQ---- -------ISG ENATHLTEYV ATRWYRAPEI MLATTCQTDF VAV------D VWSIGCIFAE LLLGKPLFKG K--------- ---------- ---EYLNKIL DVLGTPEEA- ----------
       591714 KPSNLLLNAN CDLKVCDFGL ARSAAS---- --------QE DNSGFMTEYV ATRWYRAPEI MLTF-KEYTK AI-------D VWSVGCILAE MLSGKPLFPG KD-------- ---------- CKSQAFAMRA PALAQVYWP- ----------
       107905 KPSNLLLNAN CDLKICDLGL ARSSNS---- --------AD ENSGFMTEYV ATRWYRAPEI MLTF-KEYTK AI-------D VWSVGCILAE MLSGKPLFPG RD-------- ---------- YHHQ-LTLIL DVLGTPTMD- ----------
        99046 KPGNLLVNAD CELKICDFGL ARGFS----- ------MDPE ENAGYMTEYV ATRWYRAPEI MLSF-QSYTK ASM------C LILSVLLSHS TCRVRNPRPL QS-------- ---------- CLLSTRALYI DRLGHPHHP
       863830 KPGNLLVNAD CELKICDFGL ARGFS----- ------DDAE SNVGFMTEYV ATRWYRAPEI MLSF-QSYTK AST------S LYSQSAQDAN NPLSTVIPSR QS-------- ---------- PGD
       287494 KPGNLLVNAD CELKICDFGL ARGFS----- ------IDPE ENAGYMTEYV ATRWYRAPEI MLSF-QSYTK ASM------F SFAPLQRPLV VELFSNIMTS
       662188 KPGNLLVNAD CELKICDFGL ARGFTPGGG- -----ASKSA GNQGFMTEYV ATRWYRAPEI MLSF-ANYVG GFH------E IIPLRY
       492594 KPGNLLVNAD CELKICDFGL ARGYTPGDG- -----ASKSA GNQGFMTEYV ATRWYRAPEI MLSF-ANYVS ALR------S
       176887 KPGNLLVNAD CELKICDFGL ARGFTPGGG- -----TSKSA GNQGFMTEYV ATRWYRAPEI MLSF-ANYLC RPT------E PDPSLPRHAL
       618168 KPGNLLVNAD CELKIADFGL ARGFS----- ------IDPE ENAGYMTEYV ATRWYRAPEI MLSF-QNYTK AST------C PPRLALPIA
       492643 KPGNLLVNAD CELKICDFGL ARGYTPGDG- -----ASKSA GNQGFMTEYV ATRWYRAPEI MLSF-ANYVS ALR------S
       125995 KPGNLLVNAD CELKICDFGL ARGFSV---- -------DPE ENAGYMTEYV ATRWYRAPEI MLSF-QSYTK ASK------S SAEPAQCLSV RPSLTRVLPQ S--------- ---------- ----MYGLLA VSLPSCWVA- ----------
       122928 KPGNLLVNAD CELKICDFGL ARGFSI---- -------DPE ENAGYMTEYV ATRWYRAPEI MLSF-QNYTK AST------L FPGTFCQMEA RKLNKTAFPS R--------- ---------- ----RLVRRL HPRRAPGRA- ----------
       154756 KPSNLLLNAN CDLKVCDFGL ARSAAS---- --------TE DNQGFMTEYV ATRWYRAPEI MLTF-KEYTK AN-------- ----GRLLRH QIATCA
        68423
     OsMPK20-2
     Consensus .......... .......... .......... .......... .......... .......... .......... ......   . .......... ..g....... .   ..a... ..         ......vaik ki...f...

              751                                                                                                                                                         900
       127228 ---------- --MFCLRTLR EIKLLK---- -YFNH----E NI-------- ---------- ---ISILDIV KPRSY--DDF TEV-YL---- ---------- ----IQELME TDMHRVIRTQ D-----LSDD HCQYFTYQ-- ----------
       287090 ---------- --MFCLRTLR EIKLLK---- -YFNH----E NI-------- ---------- ---ISILDIM KPKSI--EDF TEV-YL---- ---------- ----IQELME TDMHRVIRTQ D-----LSDD HCQYFTYQ-- ----------
       186494 ---------- --MFCLRTLR EIKLLK---- -YFNH----E NI-------- ---------- ---ISILDIM KPKSI--DDF TEV-YL---- ---------- ----IQELME TDMHRVIRTQ D-----LSDD HCQYFTYQ-- ----------
       260272 ---------- --MFCLRTLR EIKLLK---- -YFNH----E NI-------- ---------- ---ISILDIV RPKSL--DEF NDV-YL---- ---------- ----IQELME TDMHRVIRTQ D-----LSDD HCQYFTYQ-- ----------
       119975 ---------- --MFCLRTLR EIKLLK---- -YFQH----E NI-------- ---------- ---VSILDIV KPATL--EDF TEV-YL---- ---------- ----IQELME TDMHRVIRTQ E-----LSED HCQYFTYQ-- ----------
         2147 ---------- --MFCLRTLR EMKLLK---- -YFNH----E NI-------- ---------- ---ISILDIQ RPSSF--ETF TEV-YL---- ---------- ----IQELME TDMHRVIRTQ T-----LSDD HCQYFIYQ-- ----------
       692376 ---------- --MFCLRTLR EMKLLR---- -YFNH----E NI-------- ---------- ---ISILDIQ KPRNY--ETF TEV-YL---- ---------- ----IQELME TDMHRVIRTQ E-----LSDD HCQYFIYQ-- ----------
       698785 ---------- --MFCLRTLR EMKLLR---- -YFNH----E NI-------- ---------- ---ISILDIQ KPRNY--ETF TEV-YL---- ---------- ----IQELME TDMHRVIRTQ E-----LSDD HCQYFIYQ-- ----------
       468116 ---------- --MFCLRTLR EMKLLR---- -YFNH----E NI-------- ---------- ---ISILDIQ KPRNF--ETF TEV-YL---- ---------- ----IQELME TDMHRVIRTQ E-----LSDD HCQYFIYQ-- ----------
       274271 ---------- --MFCLRTLR EMKLLR---- -YFNH----E NI-------- ---------- ---ISILDIQ RPRNY--ESF TEV-YL---- ---------- ----IQELME TDMHRVIRTQ E-----LSDD HCQYFIYQ-- ----------
       255486 ---------- --MFCLRTLR EMKLLR---- -YFNH----E NI-------- ---------- ---ISILDIQ KPRNY--ESF SEV-YL---- ---------- ----IQELME TDMHRVIRTQ E-----LSDD HCQYFIYQ-- ----------
       285227 ---------- --MFCLRTLR EMKLLR---- -YFNH----E NI-------- ---------- ---ISILDIQ RPRSF--ESF SEV-YL---- ---------- ----IQELME TDMHRVIRTQ E-----LSDD HCQYFIYQ-- ----------
        18186 ---------- --MFCLRTLR EMKLLR---- -YFNH----E NI-------- ---------- ---ISILDIQ KPRSY--ETF NEV-YL---- ---------- ----IQELME TDMHRVIRTQ D-----LSDD HCQYFIYQ-- ----------
       465108 ---------- --MFCLRTLR EMKLLR---- -YFNH----E NI-------- ---------- ---ISILDIQ KPRNY--DSF TEV-YL---- ---------- ----IQELME TDMHRVIRTQ D-----LSDD HCQYFIYQ-- ----------
      1164585 ---------- --MFCLRTLR EMKLLR---- -YFNH----E NI-------- ---------- ---ISILDIQ KPRSY--DSF NEV-YL---- ---------- ----IQELME TDMHRVIRTQ D-----LSDD HCQYFIYQ-- ----------
        93851 ---------- --MFALRTLR EMKLLR---- -YFNH----E NI-------- ---------- ---ISILDIQ KPKSY--ETF TEV-YL---- ---------- ----IQELME TDMHRVIRTQ E-----LSDD HCQYFIYQ-- ----------
       108267 ---------- --MFCLRTLR EMKLLR---- -YFNH----E NV-------- ---------- ---ISILDIQ KPRSY--ESF SEV-YL---- ---------- ----IQELME TDMHRVIRTQ E-----LSDD HCQYFIYQ-- ----------
        59022 ---------- --MFCLRTLR EMKLLR---- -YFNH----E NI-------- ---------- ---ISILDIQ KPRSY--ESF TEV-YL---- ---------- ----VQELME TDMHRVIRTQ D-----LSDD HCQYFIYQ-- ----------
       370137 ---------- --MFCLRTLR EMKLLR---- -YFNH----E NI-------- ---------- ---ISILDIQ KPRNY--DTF TEV-YL---- ---------- ----IQELME TDMHRVIRTQ E-----LSDD HCQYFIYQ-- ----------
       532216 ---------- --MFCLRTLR EMKLLR---- -YFNH----E NI-------- ---------- ---ISILDIQ KPRNY--DTF TEV-YL---- ---------- ----IQELME TDMHRVIRTQ E-----LSDD HCQYFIYQ-- ----------
        31240 ---------- --MFCLRTLR EMKLLR---- -YFNH----E NI-------- ---------- ---ISILDIQ KPRRF--EDF TEV-YL---- ---------- ----IQELME TDMHRVIRTQ E-----LSDD HCQYFIYQ-- ----------
       160345 ---------- --MFCLRTLR EMKLLR---- -YFNH----E NI-------- ---------- ---ISILDIQ RPRNY--ETF TEV-YL---- ---------- ----IQELME TDMHRVIRTQ D-----LSDD HCQYFIYQ-- ----------
       212060 ---------- --MFCLRTLR EMKLLR---- -YFNH----E NI-------- ---------- ---ISILDIQ KPRNY--ETF TEV-YL---- ---------- ----IQELME TDMHRVIRTQ D-----LSDD HCQYFIYQ-- ----------
       327005 ---------- --MFCLRTLR EMKLLR---- -YFNH----E NI-------- ---------- ---ISILDIQ KPRNF--ESF SEV-YL---- ---------- ----IQELME TDMHRVIRTQ E-----LSDD HCQYFIYQ-- ----------
        48412 ---------- --MFCLRTLR EMKLLR---- -YFNH----E NI-------- ---------- ---ISILDIQ KPRNY--ESF SEV-YL---- ---------- ----IQELME TDMHRVIRTQ E-----LSDD HCQYFIYQ-- ----------
       606069 ---------- --MFCLRTLR EMKLLR---- -YFNH----E NI-------- ---------- ---ISILDIQ KPRSY--ETF TEV-YL---- ---------- ----IQELME TDMHRVIRTQ D-----LSDD HCQYFIYQ-- ----------
       123724 ---------- --MFCLRTLR EMKLLR---- -YFNH----E NI-------- ---------- ---ISILDIQ KPRSY--ETF TEV-YL---- ---------- ----IQELME TDMHRVIRTQ D-----LSDD HCQYFIYQ-- ----------
       342035 ---------- --MFCLRTLR EMKLLR---- -YFNH----E NI-------- ---------- ---ISILDIQ KPRGY--DTF NEV-YL---- ---------- ----IQELME TDMHRVIRTQ D-----LSDD HCQYFIYQ-- ----------
      2135321 ---------- --MFCLRTLR EMKLLR---- -YFNH----E NI-------- ---------- ---ISILDIQ KPRSY--ETF NEV-YL---- ---------- ----IQELME TDMHRVIRTQ E-----LSDD HCQYFIYQ-- ----------
       644393 ---------- --MFCLRTLR EMKLLR---- -YFNH----E NI-------- ---------- ---ISILDIQ KPRSY--ETF NEV-YL---- ---------- ----IQELME TDMHRVIRTQ E-----LSDD HCQYFIYQ-- ----------
       784446 ---------- --MFCLRTLR EMKLLR---- -YFNH----E NI-------- ---------- ---ISILDIQ KPRSY--ETF NEV-YL---- ---------- ----IQELME TDMHRVIRTQ E-----LSDD HCQYFIYQ-- ----------
       567834 ---------- --MFCLRTLR EMKLLR---- -YFNH----E NI-------- ---------- ---ISILDIQ KPRSY--ETF NEV-YL---- ---------- ----IQELME TDMHRVIRTQ E-----LSDD HCQYFIYQ-- ----------
       389770 ---------- --MFCLRTLR EMKLLR---- -YFNH----E NI-------- ---------- ---ISILDIQ KPRSY--ETF NEV-YL---- ---------- ----IQELME TDMHRVIRTQ E-----LSDD HCQYFIYQ-- ----------
        63860 ---------- --MFCLRTLR EMKLLR---- -YFNH----E NI-------- ---------- ---ISILDIQ KPRSY--ETF NEV-YL---- ---------- ----IQELME TDMHRVIRTQ E-----LSDD HCQYFIYQ-- ----------
       131603 ---------- --MFCLRTLR EMKLLR---- -YFNH----E NI-------- ---------- ---ISILDIQ KPRSY--ETF NEV-YL---- ---------- ----IQELME TDMHRVIRTQ E-----LSDD HCQYFIYQ-- ----------
       495859 ---------- --MFCLRTLR EMKLLR---- -YFNH----E NI-------- ---------- ---ISILDIQ RPRSF--ENF NEV-YL---- ---------- ----IQELME TDMHRVIRTQ D-----LSDD HCQYFIYQ-- ----------
       434551 ---------- --MFCLRTLR EMKLLR---- -YFNH----E NI-------- ---------- ---ISILDIQ RPRSF--ENF NEV-YL---- ---------- ----IQELME TDMHRVIRTQ D-----LSDD HCQYFIYQ-- ----------
       140828 ---------- --MFCLRTLR EMKLLR---- -YFNH----E NI-------- ---------- ---ISILDIQ KPRSY--ETF NEV-YL---- ---------- ----IQELME TDMHRVIRTQ E-----LSDD HCQYFIYQ-- ----------
        66435 ---------- --MFCLRTLR EMKLLR---- -YFNH----E NI-------- ---------- ---ISILDIQ KPRNY--ESF TEV-YL---- ---------- ----IQELME TDMHRVIRTQ D-----LSDD HCQYFIYQ-- ----------
       383913 ---------- --MFCLRTLR EMKLLR---- -YFNH----E NI-------- ---------- ---ISILDIQ RPRNY--ESF TEV-YL---- ---------- ----IQELME TDMHRVIRTQ D-----LSDD HCQYFIYQ-- ----------
       323815 ---------- --MFCLRTLR EMKLLR---- -YFNH----E NI-------- ---------- ---ISILDIQ RPRNY--ESF TEV-YL---- ---------- ----IQELME TDMHRVIRTQ D-----LSDD HCQYFIYQ-- ----------
       396599 ---------- --MFCLRTLR EMKLLR---- -YFNH----E NI-------- ---------- ---ISILDIQ RPRNY--EGF NEV-YL---- ---------- ----IQELME TDMHRVIRTQ D-----LSDD HCQYFIYQ-- ----------
        74447 ---------- --MFCLRTLR EMKLLR---- -YFNH----E NI-------- ---------- ---ISILDIQ RPRNY--EGF NEV-YL---- ---------- ----IQELME TDMHRVIRTQ D-----LSDD HCQYFIYQ-- ----------
        83363 ---------- --MFCLRTLR EMKLLR---- -YFNH----E NI-------- ---------- ---ISILDIQ RPRNY--EGF TEV-YL---- ---------- ----IQELME TDMHRVIRTQ D-----LSDD HCQYFIYQ-- ----------
        50905 ---------- --MFCLRTLR EMKLLR---- -YFNH----E NI-------- ---------- ---ISILDIQ KPRNY--ESF TEV-YL---- ---------- ----IQELME TDMHRVIRTQ D-----LSDD HCQYFIYQ-- ----------
       650405 ---------- --MFCLRTLR EMKLLR---- -YFNH----E NI-------- ---------- ---ISILDIQ KPRDY--ESF SEV-YL---- ---------- ----IQELME TDMHRVIRTQ D-----LSDD HCQYFIYQ-- ----------
       493847 ---------- --MFCLRTLR EMKLLR---- -YFNH----E NI-------- ---------- ---ISILDIQ KPRDY--ESF SEV-YL---- ---------- ----IQELME TDMHRVIRTQ D-----LSDD HCQYFIYQ-- ----------
        95409 ---------- --MFCLRTLR EIKLLK---- -YFDH----E NI-------- ---------- ---ISILDVQ KPKSF--DSF CEV-YL---- ---------- ----IQELMD TDMHRVIRTQ D-----LSDD HCQYFIYQ-- ----------
       835420 ---------- --MFCLRTLR EIKLLK---- -YFNH----E NI-------- ---------- ---ISILDIV KPPSL--EAF QEV-YL---- ---------- ----IQELME TDMHRVIRTQ D-----LSDD HCQYFIYQ-- ----------
       343609 ---------- --MFCLRTLR EIKLLR---- -YFNH----E NI-------- ---------- ---IAILDIV KPESL--EKF TEV-YL---- ---------- ----IQELME TDMHRVIRTQ D-----LSDD HCQYFIYQ-- ----------
        67554 ---------- --MFCLRTLR EMKLLR---- -YFNH----E NI-------- ---------- ---ISILDIQ KPRNF--ETF TEV-YL---- ---------- ----IQELME TDMHRVIRTQ E-----LSDD HCQYFIYQ-- ----------
       216015 ---------- --MLCLRTLR ELKLLK---- -NFNH----E NI-------- ---------- ---ISILALQ RPSSY--ETF NEI-YL---- ---------- ----IQELME TDLHRVIRTQ K-----LSDD HIQYFIYQ-- ----------
        52193 ---------- --MLCLRTLR ELKLLK---- -HFNH----E NI-------- ---------- ---ISILAIQ RPVSY--EFF NEI-YL---- ---------- ----IQELME TDLHRVIRTQ K-----LTDD HIQYFIYQ-- ----------
        53916 ---------- --MLCLRTLR ELKLLK---- -HFNH----E NI-------- ---------- ---ISILAIQ RPASY--ETF NEI-YL---- ---------- ----IQELME TDLHRVIRTQ K-----LTDD HIQYFIYQ-- ----------
        83931 ---------- --MFCLRTLR ELKLLK---- -HFNH----E NI-------- ---------- ---ISILDIQ IPYSF--ESF NEV-YL---- ---------- ----IQELME TDLHRVIKTQ K-----LSDD HCQYFIYQ-- ----------
        51986 ---------- --MFCLRTLR EIKLLR---- -YFNH----E NI-------- ---------- ---IAILDVQ KPTNY--ELF SEV-YI---- ---------- ----IQELME TDMHKVIRTQ V-----LSDD HCQYFIYQ-- ----------
        49589 ---------- --MFCLRTLR EIKLLR---- -YFSH----E NI-------- ---------- ---ISILDIQ KPASF--GTF TEV-YL---- ---------- ----IQELME TDLHRVIRTQ D-----LSDD HCQYLIYQ-- ----------
       862988 ---------- --MFCLRTLR ELKLLK---- -FLSESGVSE NI-------- ---------- ---ISILDII KPPSL--EAF KEV-YL---- ---------- ----IQELME TDMHRVIRTQ D-----LSDD HAQYFIYQ-- ----------
       833500 ---------- --MFCLRTLR ELKLLK---- -FLSEAGVSE NI-------- ---------- ---ISILDII KPPSL--DAF KEV-YL---- ---------- ----IQELME TDMHRVIRTQ D-----LSDD HAQYFIYQ-- ----------
       810703 ---------- --MFCLRTLR ELKLLK---- -FLSEAGVSE NI-------- ---------- ---ISILDII KPSSI--DAF KEV-YL---- ---------- ----IQELME TDMHRVIRTQ D-----LSDD HAQYFIYQ-- ----------
       457677 ---------- --MFCLRTLR ELKLLK---- -FLSEAGVSE NI-------- ---------- ---ISILDII KPPSI--EAF KEV-YL---- ---------- ----IQELME TDMHRVIRTQ D-----LSDD HAQYFIYQ-- ----------
       161161 ---------- --MFCLRTLR ELKLLK---- -FLSEAGVSE NI-------- ---------- ---ISILDII KPRSI--EEF KEV-YL---- ---------- ----IQELME TDMHRVIRTQ D-----LSDD HAQYFIYQ-- ----------
```

```
   1588194  ---------- --MFCLRTLR ELKLLK---- -FLSEAGVSE NI-------- ---------- ---ISILDII KPPSI--EQF KEV-YL---- ---------- ----IQELME TDMHRVIRTQ D-----LSDD HAQYFIYQ-- ----------
     98485  ---------- --MFCLRTLR ELKLLK---- -FLSEAGVSE NI-------- ---------- ---ISILDII KPRSI--DDF KEV-YL---- ---------- ----IQELME TDMHRVIRTQ D-----LSDD HAQYFIYQ-- ----------
   1443964  ---------- --MFCLRTLR ELKLLK---- -FLSEAGVSE NI-------- ---------- ---ISIIDII KPPSI--EAF KEV-YL---- ---------- ----IQELME TDMHRVIRTQ D-----LSDD HAQYFIYQ-- ----------
    864221  ---------- --MFCLRTLR ELKLLK---- -FLSEAGVSE NI-------- ---------- ---ISILDII KPPSL--DAF KEV-YL---- ---------- ----IQELME TDMHRVIRTQ D-----LSDD HAQYFIYQ-- ----------
    903905  ---------- --MFCLRTLR ELKLLK---- -FLSESGVSE NI-------- ---------- ---ISILDII RPASL--EAF KEV-YL---- ---------- ----IQELME TDMHRVIRTQ D-----LSDD HAQYFIYQ-- ----------
   1147795  ---------- --MFCLRTLR ELKLLK---- -FLSEAGVSE NI-------- ---------- ---ISILDII KPPSI--EQF KEV-YL---- ---------- ----IQELME TDMHRVIRTQ D-----LSDD HAQYFIYQ-- ----------
    622735  ---------- --MFCLRTLR ELKLLK---- -FLSEAGVSE NI-------- ---------- ---ISILDIV KPPSL--DAF KEV-YL---- ---------- ----IQELME TDMHRVIRTQ D-----LSDD HAQYFVYQ-- ----------
    166786  ---------- --MFCLRTLR ELKLLK---- -FLSEAGVSE NI-------- ---------- ---ISILDII KPPSL--DAF KEV-YL---- ---------- ----IQELME TDMHRVIRTQ D-----LSDD HAQYFVYQ-- ----------
   2641010  ---------- --MFCLRTLR ELKLLK---- -FLSEAGVCE NI-------- ---------- ---ISILDII KPPSI--EQF KEV-YL---- ---------- ----IQELME TDMHRVIRTQ D-----LSDD HAQYFIYQ-- ----------
     28679  ---------- --MFCLRTLR ELKLLK---- -FLSEAGVSE NI-------- ---------- ---ISILDII KPPSL--EAF KEV-YL---- ---------- ----IQELME TDMHRVIRTQ D-----LSDD HAQYFVYQ-- ----------
    101027  ---------- --MFCLRTLR ELKLLK---- -FLSEAGVSE NI-------- ---------- ---ISILDII KPPSL--EAF KEV-YL---- ---------- ----IQELME TDMHRVIRTQ D-----LSDD HAQYFVYQ-- ----------
    564549  ---------- --MFCLRTLR ELKLLK---- -FLSECGASE NI-------- ---------- ---ISILDII RPPSL--EAF KEV-YI---- ---------- ----IQELME TDMHRVIRTQ D-----LSDD HAQYFVYQ-- ----------
    227465  ---------- --MFCLRTLR ELKLLK---- -FLSEAG--- -I-------- ---------- ---ISILDII KPPTI--EQF KEV-YL---- ---------- ----IQELME TDMHRVIRTQ D-----LSDD HAQYFVYQ-- ----------
     18095  ---------- --MFCLRTLR ELKLLK---- -FLSEAGVSE NI-------- ---------- ---ISILDLV KPPSL--EAF KEV-YL---- ---------- ----IQELME TDMHRVIRTQ D-----LSDD HAQYFIYQ-- ----------
    338545  ---------- --MFCLRTLR ELKLLK---- -FLSEAGVSE NI-------- ---------- ---ISILDII KPPSI--EQF KEV-YL---- ---------- ----IQELME TDMHRVIRTQ D-----LSDD HAQYFIYQ-- ----------
    134728  ---------- --MFCLRTLR ELKLLK---- -FLSEAGVSE NI-------- ---------- ---ISILDII KPPSL--EAF KEV-YL---- ---------- ----IQELME TDMHRVIRTQ D-----LSDD HAQYFTYQ-- ----------
    275037  ---------- --MFCLRTLR ELKLLK---- -FLSEAGVSE NI-------- ---------- ---ISILDII KPPSI--EQF KEV-YL---- ---------- ----IQELME TDMHRVIRTQ D-----LSDD HAQYFIYQ-- ----------
    503339  ---------- --MFCLRTLR ELKLLK---- -FLSEAGVSE NI-------- ---------- ---ISILDII KPPSL--DAF KEV-YL---- ---------- ----IQELME TDMHRVIRTQ D-----LSDD HAQYFIYQ-- ----------
     40745  ---------- --MFCLRTLR ELKLLK---- -YFQEQNVSE NI-------- ---------- ---ISIVDII RPPSI--EAF KEV-YL---- ---------- ----IQELME TDMHRVIRTQ D-----LSDD HCQYFIYQ-- ----------
     43876  ---------- --MFALRTLR ELKLLR---- -FFAEHQVSE NL-------- ---------- ---ITVIDLI KPLSY--DSF QEV-YL---- ---------- ----IQELME TDLYRVIRTQ P-----LSDD HVQYFVYQ-- ----------
     14716  ---------- --MFALRTLR ELKLLR---- -FFAEHQVSE NL-------- ---------- ---ITVIDLI KPLSY--DSF QEV-YL---- ---------- ----IQELME TDLYRVIRTQ P-----LSDD HVQYFVYQ-- ----------
     15138  ---------- --MFALRTLR ELKLLR---- -YFAEQQVSE NI-------- ---------- ---ISIVDIV KPTSF--DSF KEV-YL---- ---------- ----IQELME TDLYRVIKTQ T-----LSDD HVQYFVYQ-- ----------
     67488  ---------- --MFLLRTLR ELKLLK---- -YFQEQNVSE NI-------- ---------- ---ISVLDMI KPKDY--PNF KEV-YL---- ---------- ----IQELME TDLHRVIRTQ D-----LSDD HAQYFIYQ-- ----------
     28680  ---------- --MFCLRTLR ELKLLK---- -FLSEAG--- -I-------- ---------- ---ISILDII KPPSL--EAF KEV-YL---- ---------- ----IQELME TDMHRVIRTQ D-----LSDD HAQYFVYQ-- ----------
    177514  ---------- --LFALRTLR ELKLLK---- -FFSETCVNE NI-------- ---------- ---ISVLDII KPDSL--ESF KEI-YF---- ---------- ----IQELMQ TDLHRVIRTQ H-----LTDD HCQYFVYQ-- ----------
     53880  ---------- --LFCLRTLR ELKLLK---- -FFSETCVNE NI-------- ---------- ---ISILDII KPPTL--DSF KEV-YF---- ---------- ----IQELMQ TDLHRVIRTQ H-----LTDD HCQYFVYQ-- ----------
    778168  ---------- --LFCLRTLR ELKLLK---- -FFSDTCVNE NI-------- ---------- ---ISILDII KPPTI--DDF TEI-YF---- ---------- ----IQELMQ TDLHRVIRTQ H-----LTDD HCQYFIYQ-- ----------
    388025  ---------- --LFCLRTLR ELKLLK---- -FFSETCVNE NI-------- ---------- ---ISILDIV KPASL--EDF KEV-YF---- ---------- ----IQELMQ TDLHRVIRTQ S-----LSDD HCQYFVYQ-- ----------
    581713  ---------- --LFCLRTLR ELKLLK---- -FFSETCVNE NI-------- ---------- ---ISILDIV KPASL--EDF KEV-YF---- ---------- ----IQELMQ TDLHRVIRTQ P-----LSDD HCQYFVYQ-- ----------
    229317  ---------- --LFCLRTLR ELKLLK---- -FFSEACVNE NI-------- ---------- ---ISILDIV KPPSL--DDF KEV-YF---- ---------- ----IQELMQ TDLHRVIRTQ P-----LSDD HCQYFIYQ-- ----------
    829657  ---------- --LFCLRTLR ELKLLR---- -FFSETCVNE NI-------- ---------- ---ISILDIT RPPSL--EEF KEV-YF---- ---------- ----IQEFMQ TDLHRVIRTQ Q-----LTDD HCQYFVYQ-- ----------
     70959  ---------- --LFCLRTLR ELKLLK---- -FFSETCVNE NI-------- ---------- ---ITILDII KPPSL--EEF KEI-YF---- ---------- ----IQELMQ TDLHRVIRTQ Q-----LTDD HCQYFVYQ-- ----------
     32857  ---------- --LFCLRTLR ELKLLK---- -FFSETCVNE NI-------- ---------- ---ICILDII KPPTL--EEF TEI-YF---- ---------- ----IQELMQ TDLHRVIRTQ Q-----LTDD HCQYFVYQ-- ----------
    561172  ---------- --LFCLRTLR ELKLLK---- -FFSDSCVNE NI-------- ---------- ---ISILDIV KPPSL--DEF TEI-YF---- ---------- ----VQELMQ TDLHRVIRTQ Q-----LTDD HCQYFVYQ-- ----------
    573944  ---------- --LFCLRTLR ELKLLK---- -FFSDSCVNE NI-------- ---------- ---ISILDII KPPSL--DDF KEI-YF---- ---------- ----IQELMQ TDLHRVIRTQ Q-----LTDD HCQYFVYQ-- ----------
    740770  ---------- --LFCLRTLR ELKLLR---- -FFSDTCQNE NI-------- ---------- ---ISILSIV KPETL--DSF KEI-YF---- ---------- ----VQEFMQ TDLHRVIRSQ K-----LTDD HCQYFVYQ-- ----------
     16071  ---------- --LFCLRTLR ELKLLR---- -FFSEVCVNE NI-------- ---------- ---VSILDII KPPSY--DDF TEI-YF---- ---------- ----VQELMQ TDLHRVIRTQ I-----LTDD HCQYFIYQ-- ----------
   1126092  ---------- --LFCLRTLR ELKLLR---- -FFSEVCVNE NI-------- ---------- ---VSILDII KPPSY--DDF TEI-YF---- ---------- ----VQELMQ TDLHRVIRTQ I-----LTDD HCQYFIYQ-- ----------
    478842  ---------- --LFCLRTLR ELKLLK---- -FFAESSVNE NI-------- ---------- ---VAILDII KPSSM--DSF KEI-YF---- ---------- ----VQELMQ TDLHRVIKSQ H-----LSDD HCQYFIYQ-- ----------
     46649  ---------- --LFCLRTLR ELKLLR---- -LFAETCLNE NI-------- ---------- ---VSILSII KPPSL--DDF KEI-YF---- ---------- ----VQELMQ TDLHRVIRTQ L-----LSDD HCQYFVYQ-- ----------
    601377  ---------- --LFALRTLR ELKLLK---- -FFSQVVVNE NI-------- ---------- ---IAILDII KPPSF--DEF KEI-YF---- ---------- ----VQELMQ TDMHHVIRTQ H-----LSPD HCEYFTYQ-- ----------
    866574  ---------- --MFCLRTLR EIKLLR---- -HFHH----E NI-------- ---------- ---IAILDIL RPPSI--NQF NEV-YL---- ---------- ----VQELME TDLHRVIRTQ E-----LSDD HCQYFIYQ-- ----------
    521401  ---------- --MFCLRTLR EIKLLR---- -HFHH----E NI-------- ---------- ---IAILDIL RPPSI--NQF NEV-YL---- ---------- ----VQELME TDLHRVIRTQ E-----LSDD HCQYFIYQ-- ----------
    850251  ---------- --MFCLRTLR EIKLLR---- -HFHH----E NI-------- ---------- ---IAILDIL RPPSI--NQF NEV-YL---- ---------- ----VQELME TDLHRVIRTQ E-----LSDD HCQYFIYQ-- ----------
    832111  ---------- --MFCLRTLR EIKLLR---- -HFHH----E NI-------- ---------- ---ISILDIL RPPSF--EMF KEV-YL---- ---------- ----VQELME TDLHRVIRTQ N-----LSDD HCQYFIYQ-- ----------
    832110  ---------- --MFCLRTLR EIKLLR---- -HFHH----E NI-------- ---------- ---ISILDIL RPPSF--EMF KEV-YL---- ---------- ----VQELME TDLHRVIRTQ N-----LSDD HCQYFIYQ-- ----------
    676666  ---------- --LFCLRTLR EIKLLR---- -HFCH----E NI-------- ---------- ---ISILDIL QPLSF--ETF KEV-YL---- ---------- ----VQELME TDLHRVIRTQ K-----LSDD HCQYFIYQ-- ----------
   1143376  ---------- --MFCLRTLR EIKLLR---- -HFCH----E NI-------- ---------- ---ISILDIL QPPSF--ELF KEV-YL---- ---------- ----VQELME TDLHRVIRTQ K-----LSDD HCQYFIYQ-- ----------
    457401  ---------- --MFCLRTLR EIKLLR---- -HFRH----E NI-------- ---------- ---ISILDIL CPPSY--DDF REV-YL---- ---------- ----VQELME TDLHRVIRTQ Q-----LSDD HCQYFIYQ-- ----------
    834255  ---------- --MFCLRTLR EIKLLR---- -HFRH----E NI-------- ---------- ---ISILDIL QPPSF--DQF REV-YL---- ---------- ----VQELME TDLHRVIRTQ Q-----LSDD HCQYFIYQ-- ----------
    133042  ---------- --MFCLRTLR EIKLLR---- -HFHH----E NI-------- ---------- ---ISILDIL RPPSL--EDF KEV-YL---- ---------- ----VQELME TDLHRVIRTQ Q-----LSDD HCQYFIYQ-- ----------
     30507  ---------- --MFSLRTLR EIKLLR---- -HFHH----E NI-------- ---------- ---ISILDIL RPPSL--DEF KEV-YL---- ---------- ----VQELME TDLHRVIRTQ E-----LSDD HCQYFIYQ-- ----------
    646041  ---------- --MFCLRTLR EIKLLR---- -HFHH----E NI-------- ---------- ---ISILDIL RPPSL--QDF KEV-YL---- ---------- ----VQELME TDLHRVIRTQ H-----LSDD HCQYFIYQ-- ----------
    570964  ---------- --MFCLRTLR EIKLLR---- -HFHH----E NI-------- ---------- ---ISILDIL RPPSL--ADF KEV-YL---- ---------- ----VQELME TDLHRVIRTQ Q-----LSDD HCQYFIYQ-- ----------
    301734  ---------- --MFCLRTLR EIKLLR---- -HFHH----E NI-------- ---------- ---ISILDIL QPASV--EEF KEV-YL---- ---------- ----VQELME TDLHRVIRTQ T-----LSDD HCQYFIYQ-- ----------
    154378  ---------- --MFCLRTLR EIKLLR---- -HFHH----E NI-------- ---------- ---ISILDIL RPQNI--HDF REV-YL---- ---------- ----VQELME TDLHRVIRTQ T-----LSDD HCQYFIYQ-- ----------
     90189  ---------- --MFCLRTLR EIKLLR---- -HFHH----E NI-------- ---------- ---IAILDIL RPPSL--ADF TEV-YL---- ---------- ----VQELME TDLHRVIRTQ E-----LSDD HCQYFIYQ-- ----------
    448371  ---------- --MFCLRTLR EIKLLR---- -HFHH----E NI-------- ---------- ---ISILDIL RPDSL--EEF REV-YL---- ---------- ----VQELME TDMHRVIRTQ E-----LSDD HCQYFIYQ-- ----------
    476779  ---------- --MFCLRTLR EIKLLR---- -HFHH----E NI-------- ---------- ---ISILDIL RPDSL--EEF REV-YL---- ---------- ----VQELME TDMHRVIRTQ E-----LSDD HCQYFIYQ-- ----------
     68819  ---------- --MFSLRTLR EIRLLR---- -HFRH----E NI-------- ---------- ---IAILDIL RPPSY--ADF REV-YL---- ---------- ----VQELME TDLHRVIRTQ E-----LSDD HCQYFIYQ-- ----------
   2671102  ---------- --MFCLRTLR EIKLLR---- -HFHH----E NI-------- ---------- ---IAILDIL RPPSL--QDF TEV-YL---- ---------- ----VQELME TDLHRVIRTQ E-----LSDD HCQYFIYQ-- ----------
    940143  ---------- --MFCLRTLR EIKLLR---- -HFDH----E NI-------- ---------- ---ISILDIL RPPSL--NEF REV-YL---- ---------- ----VQELME TDMHRVIRTQ E-----LSDD HCQYFIYQ-- ----------
    585786  ---------- --MFCLRTLR EIKLLR---- -HFDH----E NI-------- ---------- ---IAILDIL RPPSL--AEF REV-YL---- ---------- ----VQELME TDMHRVIRTQ E-----LSDD HCQYFIYQ-- ----------
    349085  ---------- --MFCLRTLR EIKLLR---- -HFDH----E NI-------- ---------- ---ISILDIL RPPSL--NEF REV-YL---- ---------- ----VQELME TDMHRVIRTQ E-----LSDD HCQYFIYQVR RVPRLPFIIR
   1539717  ---------- --MFALRTLR EIKLLR---- -HFNH----E NI-------- ---------- ---ISILDIL RPHSM--ESF KEV-YL---- ---------- ----IQELME TDMHRVIRTQ E-----LSDD HCQYFIYQ-- ----------
     24638  ---------- --MFCLRTLR EIKLLR---- -HFRH----E NI-------- ---------- ---ISILDIL KPESF--EAF KEV-YL---- ---------- ----VQELME TDMHRVIRTQ E-----LSDD HCQYFIYQ-- ----------
     75000  ---------- --MFCLRTLR EIKLLR---- -HFRH----E NI-------- ---------- ---ISILDIL KPESF--EAF KEV-YL---- ---------- ----VQELME TDMHRVIRTQ E-----LSDD HCQYFIYQ-- ----------
   1245527  ---------- --MFALRTLR EIKLLR---- -HFHH----E NI-------- ---------- ---ISILDIL RPPSL--DDF KEV-YL---- ---------- ----VQELME TDLHRVIRTQ E-----LSDD HCQYFIYQ-- ----------
    103018  ---------- --MFCLRSLR EIKLLR---- -HFQH----E NI-------- ---------- ---ISILDII RPASI--DAF NEI-YL---- ---------- ----IQELME TDMHRVIRTQ E-----LSDD HCQYFLYQ-- ----------
     43883  ---------- --MFCLRTLR EIKLLR---- -WFAH----E NI-------- ---------- ---ISILDII KPSTY--EGF SEV-YL---- ---------- ----IQELME TDLHRVIRSQ E-----LSDD HCQYFLYQ-- ----------
     58913  CILLNLISID LIVFCLRTLR EIKLLR---- -WFSH----E NI-------- ---------- ---ISILDIV KPPSI--EEF SEV-YL---- ---------- ----IQELME TDMHRVIRTQ D-----LSDD HSQYFIYQ-- ----------
     18326  ---------- --MFCLRTLR EIKILK---- -HFNH----E NI-------- ---------- ---ISILDIL RPPNL--ESF TQV-YL---- ---------- ----VQELME TDMHRVIRTQ E-----LSDD HCQYFIYQ-- ----------
    301059  ---------- --MFCLRTLR EIKILK---- -HFNH----E NI-------- ---------- ---ISILDIL RPPNL--ESF TQV-YL---- ---------- ----VQELME TDMHRVIRTQ E-----LSDD HCQYFIYQ-- ----------
     12410  ---------- --MFCLRTLR EIKILK---- -HFNH----E NI-------- ---------- ---ISILDIL RPPNL--ESF TQV-YL---- ---------- ----VQ---- ---------- ---------- ---------- ----------
     18327  ---------- --MFCLRTLR EIKILK---- -HFNH----E NI-------- ---------- ---ISILDIL RPPNL--ESF TQV-YL---- ---------- ----VQELME TDMHRVIRTQ E-----LSDD HCQYFIYQ-- ----------
    184754  ---------- --LFCLRTLR EIKILK---- -YFKH----E NI-------- ---------- ---ITVFDIQ RPLDF--ENF NEV-YI---- ---------- ----IQELMQ TDLHKVISTQ A-----LTDD HCQYFIYQ-- ----------
     99455  ---------- --LFCVRTLR EIKLLK---- -HFKH----E NI-------- ---------- ---ISIFDIQ KPGSF--EDF KEV-YI---- ---------- ----IQELMQ TDLHRAIISQ R-----LTDD HVQYFIYQ-- ----------
    706256  ---------- --LFALRTLR EIKILK---- -HFKH----E NI-------- ---------- ---ITIFNIQ RPDSF--ENF NEV-YI---- ---------- ----IQELMQ TDLHRVISTQ M-----LSDD HIQYFIYQ-- ----------
    653342  ---------- --LFALRTLR EIKILK---- -HFKH----E NI-------- ---------- ---ITIFNIQ RPDSF--ENF NEV-YI---- ---------- ----IQELMQ TDLHRVISTQ M-----LSDD HIQYFIYQ-- ----------
    462249  ---------- --MFALRTLR ELKLLK---- -FLSEAGVSE NI-------- ---------- ---ISILDII KPASI--EQF KEV-YL---- ---------- ----IQELME TDMHRVIRTQ D-----LSDD HAQYFIYQ-- ----------
    326973             MFCLRTLR EMKLLR---- -YFNH----E NI-------- ---------- ---ISILDIQ KPRNF--ESF SEV-YL---- ---------- ----IQELME TDMHRVIRTQ E-----LSDD HCQYFIYQ-- ----------
     77855             MFCLRTLR EMKLLR---- -YFNH----E NI-------- ---------- ---ISILDIQ RPRNY--DSF TEV-YL---- ---------- ----IQELME TDMHRVIRTQ D-----LSDD HCQYFIYQ-- ----------
    338563             MFCLRTLR ELKLLK---- -FLSEAGVSE NI-------- ---------- ---ISILDII KPPSI--EQF KEV-YL---- ---------- ----IQELME TDMHRVIRTQ D-----LSDD HAQYFIYQ-- ----------
    233520             MFCLRTLR ELKLLK---- -FLSEAGVSE NI-------- ---------- ---ISILDII KPPSL--DAF KEV-YL---- ---------- ----IQELME TDMHRVIRTQ D-----LSDD HAQYFIYQ-- ----------
     73610             MFCLRTLR ELKLLK---- -FLSEAGVSE NI-------- ---------- ---ISILDLV KPPSL--EAF KEV-YL---- ---------- ----IQELME TDMHRVIRTQ D-----LSDD HAQYFIYQ-- ----------
    150924             MFCLRTLR ELKLLK---- -FLSEAGVSE NI-------- ---------- ---ISILDII KPPSL--EAF KEV-YL---- ---------- ----IQELME TDMHRVIRTQ D-----LSDD HAQYFVYQ-- ----------
    348809             MFCLRTLR ELKLLK---- -FLSESGVSE NI-------- ---------- ---ISILDII RPASL--EAF KEV-YL---- ---------- ----IQELME TDMHRVIRTQ D-----LSDD HAQYFIYQ-- ----------
    913771  ---------- --TTPCSVSA PSASSS---- -SSSSSARPV SI-------- ---------- ---ISIIDII KPPSI--EAF KEV-YL---- ---------- ----IQELME TDMHRVIRTQ D-----LSDD HAQYFIYQ-- ----------
    913918             MFCLRTLR ELKLLK---- -FLSEAG--- -I-------- ---------- ---ISIIDII KPPSI--EAF KEV-YL---- ---------- ----IQELME TDMHRVIRTQ D-----LSDD HAQYFIYQ-- ----------
    747578  ---------- --LFVTRTIR EIKLLR---- -YFHE---HE NI-------- ---------- ---ISILDKV RPVSI--DKL NAV-YL---- ---------- ----VEELME TDLQKVINNQ NSGFSTLSDD HVQYFTYQ-- ----------
    458977  ---------- --LFVTRTIR EIKLLR---- -YFHE---HE NI-------- ---------- ---ISILDKV RPVSI--DKL NAV-YL---- ---------- ----VEELME TDLQKVINNQ NSGFSTLSDD HVQYFVYQ-- ----------
     41459  ---------- --LFCLRTLR ELKLLNPHPS -VWHVRVHSR RLQIISILDI IKPPSLADFT EIYCENHMID QGPAC--ASD HVY-AV---- ---------- ----IQELMQ TDLHRVIRTQ Q-----LTDD HCQYFVYQ-- ----------
   1397463  ---------- --LFCLRTLR ELKLLK---- -FFSEMCVNE NVCVPSLSCQ GPPFNVSYVT LQIISILDIV KPPSY--DDF KEI-YF---- ---------- ----IQELMQ TDLHRVIRTQ Q-----LTDD HCQYFIYQ-- ----------
    149203                          MVS -VKAHTALSL PLFTSPPTER SPSRRSSPSS IPSSASLDII KPPSL--DDF KEI-YF---- ---------- ----IQELMQ TDLHRVIRTQ Q-----LTDD HCQYFVYQ-- ----------
    102078  ---------- --IVCLRTIR ELHLLN---F -FRNH----E NI-------- ---------- ---IGFYDVQ RPKDF--ESF NEV-YL---- ---------- ----IQEYMP TDLHKLNHSS D-----ISDK HIQYFIYQ-- ----------
 36766:44-3 ---------- --LFCLRTLR EIKLLS---R -LSNH----E NI-------- ---------- ---VQLYDVQ QPQDF--NSF KEV-YI---- ---------- ----IQEYMP TDLVNVIESQ I-----LSEE HVQYFTYQ-- ----------
     54278  ---------- --LFCLRTLR EIKLLS---K -FDNH----E NI-------- ---------- ---IKLYDVQ KPYNY--QMF HEV-YL---- ---------- ----IQEYMP SDLHNIIRTH T-----LTDQ HVQYFVYQ-- ----------
     29185  ---------- --MFALRTLR ELKLLR---- -YFAEQQVSE NIISI----- -----VDIRY EHCSFLVHLV RPGSLTISLD EKV-YL---- ---------- ----IQELME TDLYRVIKTQ T-----LSDD HVQYFVYQ-- ----------
    790328  ---------- --MFCLRTLR EIKLLR---- -HFHH----E NI-------- ---------- ---IAILDIL RPPSI--NQF NEV-YL---- ---------- ----VQELME TDLHRVIRT- QE----LSDD HCQYFIYQ-- ----------
    765763  ---------- --MFCLRTLR EIKLLR---- -HFHH----E NI-------- ---------- ---IAILDIL RPPSI--NQF NEV-YL---- ---------- ----VQELME TDLHRVIRT- QE----LSDD HCQYFIYQ-- ----------
   1491990  ---------- --MFALRTLR EIKLLR---- -HFNH----E NI-------- ---------- ---ISILDIL RPHSM--ESF KEV-YLIQVS TMPPSSTYCL QISMVQELME TDMHRVIRT- QE----LSDD HCQYFIYQ-- ----------
     32998  ---------- --MFCLRTLR ELKLLK---- -FLSEAGVSE NI-------- ---------- ---ISILDII KPRSI--DDF KEV-YL---- ---------- ----IQELME TDMHRVIRT- QD----LSDD HAQYFIYQ-- ----------
   2601990  ---------- --MFCLRTLR EIKLLR---- -HFHH----E NI-------- ---------- ---IAILDIL RPPSL--QDF TEV-YL---- ---------- ----VQELME TDLHRVIRT- QE----LSDD HCQYFIYQ-- ----------
    262460  ---------- --MFCLRTLR EMKLLR---- -YFNH----E NI-------- ---------- ---ISILDIQ KPRNY--ESF SEV-YL---- ---------- ----IQELME TDMHRVIRT- QE----LSDD HCQYFIYQ-- ----------
     11717  ---------- --VDAKRILR EVRLLG---- -HFKH----K NI-------- ---------- ---TRLLDLS PPPSR--KQF DDM-YI---- ---------- ----ITELME TDLHQVIYSM QP----MSDD HVKYFLYQ-- ----------
    532299  ---------- --VDAKRILR EVRLLG---- -HFSH----K NI-------- ---------- ---TRLLDLS PPPSR--KQF DDM-YI---- ---------- ----ITELME TDLHQVIYSM QP----MSDD HVKYFLYQ-- ----------
    551831  ---------- --VDAKRILR EVRLLG---- -HFNH----K NI-------- ---------- ---TRLLDLS PPPSR--KQF DDM-YI---- ---------- ----ITELME TDLHQVIYSM QP----MSDD HVKYFLYQ-- ----------
    301484  ---------- --VDAKRILR EVRLLG---- -HFSH----K NI-------- ---------- ---TRLLDLS PPPSR--KQF DDM-YI---- ---------- ----ITELME TDLHQVIYSM QP----MSDD HVKYFLYQ-- ----------
     42366  ---------- --ITCLYDM- ---------- ---------- ---------- ---------- -------DIV FEPD---GNF GEV-YI---- ---------- ----YEELME ADLHAIIRSG QP----LSDA HVQSFLYQ-- ----------
    476004  ---------- --TDAKRIVR EIKLMR---- -HLDH----K CV-------- ---------- ---LGVEDIF EPVAL--DKF EDV-YI---- ---------- ----VSQLMA TDLHRVIYSR HA----LSDE HIAFFMYQ-- ----------
    558476  ---------- --TDAKRIVR EIKLMR---- -HLNH----K CV-------- ---------- ---LGVEDIF EPVAL--DKF EDV-YI---- ---------- ----VSQLMA TDLHRVIYSR HA----LSDE HIAFFMYQ-- ----------
      9954  ---------- --VDAKRILR EIKLLQ---- -HFDH----E NV-------- ---------- ---ITILDLL PPPSL--AQF EDV-YI---- ---------- ----IADLME TDLHRIIYSR QP----LTDD HVQYFLYQ-- ----------
     82136  ---------- --VDAKRILR EIKLLQ---- -HFDH----E NV-------- ---------- ---ITIVDLL PPPSL--AQF EDV-YI---- ---------- ----IADLME TDLHRIIYSR QP----LTDD HVQYFLYQ-- ----------
    312415  ---------- --VDAKRILR EIKLLQ---- -HFDH----E NV-------- ---------- ---ITIVDLL PPPSL--AQF EDV-YI---- ---------- ----IADLME TDLHRIIYSR QP----LTDD HVQYFLYQ-- ----------
    354114  ---------- --HQLKQILR ECRLIR---- -HMAH----E NV-------- ---------- ---LSLLDLI PPPSY--TDF RDV-YM---- ---------- ----TVDLME MDLHRIIYSK EV----LRDD HIRYFLYQ-- ----------
     34134  ---------- --QLAKRALR ELTLLR---- -HFSH----E NI-------- ---------- ---TGLIDVD ISPN-----H DEI-YI---- ---------- ----FMEPME ADLHQIIKSG QS----LTVE HVQYFVYQ-- ----------
     20005  ---------- --TDAKRILR EMKLLR---- ----HLGVHE NIIN------ ---------- ----ILDVIL IPPNV--MDF QDI-YI---- ---------- ----VTDLME SDLERIISSS QP----LSDA HFQYFVYQ-- ----------
    518921  ---------- --TDAKRILR EMKLLR---- ----HLGVHE NIINVGCSR- ---------- ----ILDVIL IPPNV--MDF HDI-YI---- ---------- ----VTDLME SDLERIISSS QP----LSDA HFQYFLYQ-- ----------
    503206  ---------- --TDAKRILR EMKLLR---- ----HLGVHE NIIN------ ---------- ----ILDVIL IPPNV--MDF HDI-YI---- ---------- ----VTDLME SDLERIISSS QP----LSDA HFQYFLYQ-- ----------
     10810  ---------- --TDAKRILR EMKLLR---- ----HLGVHE NIIN------ ---------- ----ILDVIL IPPNV--MDF QDI-YI---- ---------- ----VTDLME SDLERIISSS QP----LSDA HFQYFLYQ-- ----------
    AtMPK1  ---------- --IDALRTLR ELKLLR---- ----HLR-HE NVI------- ---------- ----ALKDVM MPIHK--MSF KDV-YL---- ---------- ----VYELMD TDLHQIIKSS QV----LSND HCQYFLFQ-- ----------
    AtMPK2  ---------- --IDALRTLR ELKLLR---- ----HLR-HE NVV------- ---------- ----ALKDVM MANHK--RSF KDV-YL---- ---------- ----VYELMD TDLHQIIKSS QV----LSND HCQYFLFQ-- ----------
     OsMPK7 ---------- --VDALRTLR ELKLLR---- ----HLR-HE NVI------- ---------- ----ALKDIM MPVHR--RSF KDV-YL---- ---------- ----VYELMD TDLHQIIKSS QG----LSND HCQYFLFQ-- ----------
    OsMPK14 ---------- --VDALRTLR ELKLLR---- ----HLR-HE NVI------- ---------- ----ALKDIM MPVHR--RSF KDV-YL---- ---------- ----VYELMD TDLHQIIKSP QG----LSND HCQYFLFQ-- ----------
   AtMPK14  ---------- --IDALRTLR ELKLLR---- ----HVR-HE NVI------- ---------- ----SLKDVM LPTHR--YSF RDV-YL---- ---------- ----VYELMD SDLNQIIKSS QS----LSDD HCKYFLFQ-- ----------
    AtMPK7  ---------- --VDALRTLR ELKLLR---- ----HVR-HE NVI------- ---------- ----ALKDVM LPANR--SSF KDV-YL---- ---------- ----VYELMD TDLHQIIKSS QS----LSDD HCKYFLFQ-- ----------
   AtMPK12  ---------- --IDAKRTLR EIKLLR---- ----HMD-HE NVI------- ---------- ----TIKDIV RPPQR--DIF NDV-YI---- ---------- ----VYELMD TDLQRILRSN QT----LTSD QCRFLVYQ-- ----------
    AtMPK4  ---------- --IDAKRTLR EIKLLK---- ----HMD-HE NVI------- ---------- ----AVKDII RPPQR--ENF NDV-YI---- ---------- ----VYELMD TDLHQIIRSN QP----LTDD HCRFFLYQ-- ----------
   OsMPK4-1 ---------- --IDAKRTLR EIKLLR---- ----HMD-HE NII------- ---------- ----AIKDII RPPRR--DNF NDV-YI---- ---------- ----VSELMD TDLHQIIRSN QP----LTDD HCQYFLYQ-- ----------
  OsMPK16-2 ---------- --IDAKRTLR EIKLLR---- ----HMD-HD NVI------- ---------- ----SIKDII RPPRR--ENF NDV-YI---- ---------- ----VYELMD TDLHHLLRSN QP----LTDD HCQYFLYQ-- ----------
    AtMPK5  ---------- --VDAKRTLR EIKLLR---- ----HLE-HE NVV------- ---------- ----VIKDII RPPKK--EDF VDV-YI---- ---------- ----VFELMD TDLHQIIRSN QS----LNDD HCQYFLYQ-- ----------
   AtMPK10  ---------- --IEAKRTLR EIKLLR---- ----HFD-HE NIV------- ---------- ----AIRDVI LPPQR--DSF EDV-YI---- ---------- ----VNELME FDLYRTLKSD QE----LTKD HGMYFMYQ-- ----------
    AtMPK6  ---------- --IDAKRTLR EIKLLR---- ----HMD-HE NIV------- ---------- ----AIRDII PPPLR--NAF NDV-YI---- ---------- ----AYELMD TDLHQIIRSN QA----LSEE HCQYFLYQ-- ----------
```

```
   OsMPK6  ---------- --IDAKRTLR EIKLLR---- ----HMD-HE NIV------- ---------- ----AIRDII PPPQR--NSF NDV-YI---- ---------- ----AYELMD TDLHQIIRSN QA----LSEE HCQYFLYQ-- ----------
   AtMPK3  ---------- --MDAKRTLR EIKLLR---- ----HLD-HE NII------- ---------- ----AIRDVV PPPLR--RQF SDV-YI---- ---------- ----STELMD TDLHQIIRSN QS----LSEE HCQYFLYQ-- ----------
   OsMPK3  ---------- --MDAKRTLR EIKLLR---- ----HLD-HE NII------- ---------- ----GIRDVI PPPIP--QAF NDV-YI---- ---------- ----ATELMD TDLHHIIRSN QE----LSEE HCQYFLYQ-- ----------
   206269  ---------- --VLAKRTYR ELKLLK---- -ALRH----E N--------- ---------- --IISLSDIF ISP------L EDI-YF---- ---------- ----VTELLG TDLHRLL--Q SR---PLEKQ FIQYFLYQIL ----------
   154265  ---------- --VLAKRTYR ELKLLK---- -ALRH----E N--------- ---------- --IISLSDIF ISP------L EDI-YF---- ---------- ----VTELLG TDLHRLL--Q SR---PLEKQ FIQYFLYQIL ----------
   168032  ---------- --VLAKRTYR ELKLLK---- -ALRH----E N--------- ---------- --IISLSDIF ISP------L EDI-YF---- ---------- ----VTELLG TDLHRLL--Q SR---PLEKQ FIQYFLYQIL ----------
   307550  ---------- --VLAKRTYR ELKLLK---- -ALRH----E N--------- ---------- --IISLSDIF ISP------L EDI-YF---- ---------- ----VTELLG TDLHRLL--Q SR---PLEKQ FIQYFLYQIL ----------
   320251  ---------- --VLSKRTYR ELKLLK---- -HLRH----E N--------- ---------- --IISLSDIF ISP------L EDI-YF---- ---------- ----VTELLG TDLHRLL--T SR---PLEKQ FIQYFLYQIL ----------
   356517  ---------- --VLAKRTYR ELKLLK---- -HLRH----E N--------- ---------- --IISLSDIF ISP------L EDI-YF---- ---------- ----VTELLG TDLHRLL--T SR---PLEKQ FIQYFLYQIM ----------
   113434  ---------- --VLAKRTYR ELKLLK---- -HIQH----E N--------- ---------- --IISLSDVF ISP------L EDI-YF---- ---------- ----VTELLG TDLHRLL--T SR---PLEKQ FIQYFLYQIL ----------
    28887  ---------- --VLAKRTYR ELKLLK---- -HIQH----E N--------- ---------- --IISLSDIF ISP------L EDI-YF---- ---------- ----VTELLG TDLHRLL--T SR---PLEKQ FIQYFLYQIL ----------
    16635  ---------- --VLSKRTYR ELKLLK---- -HLQH----E N--------- ---------- --IISLSDIF ISP------L EDI-YF---- ---------- ----VTELLG TDLHRLL--T SR---PLEKQ FIQYFLYQIL ----------
     6923  ---------- --VLSKRTYR ELKLLK---- -HIQH----E N--------- ---------- --IIALSDVF ISP------L EDI-YF---- ---------- ----VTELLG TDLHRLL--T SR---PLEKQ FIQYFLYQIL ----------
   807502  ---------- --VLSKRTYR ELKLLK---- -HIQH----E N--------- ---------- --IISLSDVF ISP------L EDL-YF---- ---------- ----VTELLG TDLHRLL--T SR---PLEKQ FIQYFLYQIL ----------
   129058  ---------- --VLSKRTYR ELKLLK---- -HIQH----E N--------- ---------- --IISLSDVF ISP------L EDL-YF---- ---------- ----VTELLG TDLHRLL--T SR---PLEKQ FIQYFLYQIL ----------
   359241  ---------- --VLSKRTYR ELKLLK---- -HIQH----E N--------- ---------- --IISLSDVF ISP------L EDL-YF---- ---------- ----VTELLG TDLHRLL--T SR---PLEKQ FIQYFLYQIL ----------
    53282  ---------- --VLAKRTYR ELKLLK---- -HIQH----E N--------- ---------- --IISLNDVF ISP------L EDL-YF---- ---------- ----VTELLG TDLHRLL--S SR---PLEKQ FIQYFLYQIL ----------
   117789  ---------- --VLAKRTYR ELKLLK---- -HIQH----E N--------- ---------- --IISLSDVF ISP------L EDL-YF---- ---------- ----VTELLG TDLHRLL--T SR---PLEKQ FIQYFLYQIL ----------
   170586  ---------- --VLAKRTYR ELKLLK---- -HIQH----E N--------- ---------- --IISLSDVF ISP------L EDI-YF---- ---------- ----VTELLG TDLHRLL--T SR---PLEKQ FIQYFLYQIL ----------
   162119  ---------- --VLAKRTYR ELKLLK---- -HIQH----E N--------- ---------- --IISLSDVF ISP------L EDI-YF---- ---------- ----VTELLG TDLHRLL--T SR---PLEKQ FIQYFLYQIL ----------
   560244  ---------- --VLSKRTYR ELKLLK---- -HIQH----E N--------- ---------- --IISLSDVF ISP------L EDI-YF---- ---------- ----VTELLG TDLHRLL--T SR---PLEKQ FIQYFLYQIL ----------
   499279  ---------- --VLSKRTYR ELKLLK---- -HIQH----E N--------- ---------- --IISLSDVF ISP------L EDI-YF---- ---------- ----VTELLG TDLHRLL--T SR---PLEKQ FIQYFLYQIL ----------
   898207  ---------- --VLAKRTYR ELKLLK---- -HIQH----E N--------- ---------- --IISLSDVF IAP------L EDI-YF---- ---------- ----VTELLG TDLHRLL--T SR---PLEKQ FIQYFLYQIL ----------
   500359  ---------- --VLAKRTYR ELKLLK---- -HIQH----E N--------- ---------- --IISLSDVF ISP------L EDI-YF---- ---------- ----VTELLG TDLHRLL--T SR---PLEKQ FIQYFLYQIL ----------
   390416  ---------- --VLSKRTYR ELKLLK---- -HITH----E N--------- ---------- --IISLSDVF ISP------L EDI-YF---- ---------- ----VTELLG TDLHRLL--T SR---PLEKQ FIQYFLYQIL ----------
    61586  ---------- --VLSKRTYR ELKLLK---- -HIQH----E N--------- ---------- --IISLSDVF ISP------L EDL-YF---- ---------- ----VTELLG TDLHRLL--T SR---PLEKQ FIQYFLYQIL ----------
    74488  ---------- --VLSKRTYR ELKLLK---- -HITH----E N--------- ---------- --IISLSDVF ISP------L EDI-YF---- ---------- ----VTELLG TDLHRLL--T SR---PLEKQ FIQYFLYQIL ----------
   155373  ---------- --VLSKRTYR ELKLLK---- -HLKH----E N--------- ---------- --IISLSDIF ISP------L EDI-YF---- ---------- ----VTELLG TDLHRLL--T SR---PLDKQ FIQYFLYQIL ----------
    56185  ---------- --VLAKRTYR ELKLLK---- -HIQH----E N--------- ---------- --IISLSDIF ISP------L EDI-YF---- ---------- ----VTELLG TDLHRLL--T SR---PLEKQ FIQYFLYQIL ----------
    14909  ---------- --VLAKRTYR ELKLLK---- -HIQH----E N--------- ---------- --IISLSDIF ISP------L EDI-YF---- ---------- ----VTELLG TDLHRLL--T SR---PLEKQ FIQYFLYQIL ----------
   176707  ---------- --VLSKRTYR ELKLLK---- -HIQH----E N--------- ---------- --IIALSDVF ISP------L EDI-YF---- ---------- ----VTELLG TDLHRLL--T SR---PLEKQ FIQYFLYQIL ----------
   176721  ---------- --VLSKRTYR ELKLLK---- -HIQH----E N--------- ---------- --IIALSDVF ISP------L EDI-YF---- ---------- ----VTELLG TDLHRLL--T SR---PLEKQ FIQYFLYQIL ----------
   240717  ---------- --VLSKRTYR ELKLLK---- -HIQH----E N--------- ---------- --IIALSDVF ISP------L EDI-YF---- ---------- ----VTELLG TDLHRLL--T SR---PLEKQ FIQYFLYQIL ----------
    56428  ---------- --VLSKRTYR ESKLLK---- -HLRH----E N--------- ---------- --VISSSDIF ISP------L EDI-YF---- ---------- ----VTELLG TDLHRLL--T SR---PLEKQ FIQYFLYQIL ----------
    97915  ---------- --VLSKRTYR ESKLLK---- -HLRH----E N--------- ---------- --VISSSDIF ISP------L EDI-YF---- ---------- ----VTELLG TDLHRLL--T SR---PLEKQ FIQYFLYQIL ----------
   707387  ---------- --VLSKRTYR ELKLLK---- -HLRH----E N--------- ---------- --VISLSDIF ISP------L EDI-YF---- ---------- ----VTELLG TDLHRLL--T SR---PLEKQ FIQYFLYQIL ----------
   504013  ---------- --VLSKRTYR ELKLLK---- -HLKH----E N--------- ---------- --VISLSDIF ISP------L EDI-YF---- ---------- ----VTELLG TDLHRLL--T SR---PLEKQ FIQYFLYQIL ----------
    37843  ---------- --VLSKRTYR ELKLLK---- -HLRH----E N--------- ---------- --VISLSDIF ISP------L EDI-YF---- ---------- ----VTELLG TDLHRLL--T SR---PLEKQ FIQYFLYQIL ----------
   507853  ---------- --VLSKRTYR ELKLLK---- -HLRH----E N--------- ---------- --VISLSDIF ISP------L EDI-YF---- ---------- ----VTELLG TDLHRLL--T SR---PLEKQ FIQYFLYQIL ----------
   338747  ---------- --VLSKRTYR ELKLLK---- -HLRH----E N--------- ---------- --IISLSDIF ISP------L EDI-YF---- ---------- ----VTELLG TDLHRLL--T SR---PLEKQ FIQYFLYQIL ----------
    49060  ---------- --VLSKRTYR ELKLLK---- -HLKH----E N--------- ---------- --VISLSDIF ISP------L EDI-YF---- ---------- ----VTELLG TDLHRLL--T SR---PLEKQ FIQYFLYQIL ----------
   147551  ---------- --VLSKRTYR ELKLLK---- -HLKH----E N--------- ---------- --VISLSDIF ISP------L EDI-YF---- ---------- ----VTELLG TDLHRLL--T SR---PLEKQ FIQYFLYQIL ----------
   286873  ---------- --VLSKRTYR ELKLLK---- -HLRH----E N--------- ---------- --VISLSDIF ISP------L EDM-YV---- ---------- ----VTELLG TDLHRLL--T SR---PLEKQ FIQYFLYQIL ----------
   193904  ---------- --VLAKRTYR ELKLLK---- -HLKH----E N--------- ---------- --VISLSDIF ISP------L EDI-YF---- ---------- ----VTELLG TDLHRLL--T SR---PLEKQ FIQYFLYQIL ----------
   189288  ---------- --VLSKRTYR ELKLLK---- -HLRH----E N--------- ---------- --IISLSDIF ISP------L EDI-YF---- ---------- ----VTELLG TDLHRLL--T SR---PLEKQ FIQYFLYQIL ----------
   198339  ---------- --VLSKRTYR ELKLLK---- -HLRH----E N--------- ---------- --IISLSDIF ISP------L EDI-YF---- ---------- ----VTELLG TDLHRLL--T SR---PLEKQ FIQYFLYQIL ----------
  2140409  ---------- --VLAKRTYR ELKLLK---- -HLKH----E N--------- ---------- --VISLSDIF ISP------L EDI-YF---- ---------- ----VTELLG TDLHRLL--T SR---PLEKQ FIQYFLYQIM ----------
   642177  ---------- --VLAKRTYR ELKLLK---- -HLKH----E N--------- ---------- --VISLSDIF ISP------L EDI-YF---- ---------- ----VTELLG TDLHRLL--T SR---PLEKQ FIQYFLYQIM ----------
   498471  ---------- --VLAKRTYR ELKLLK---- -HLKH----E N--------- ---------- --VISLSDIF ISP------L EDI-YF---- ---------- ----VTELLG TDLHRLL--T SR---PLEKQ FIQYFLYQIM ----------
   784216  ---------- --VLAKRTYR ELKLLK---- -HLKH----E N--------- ---------- --VISLSDIF ISP------L EDI-YF---- ---------- ----VTELLG TDLHRLL--T SR---PLEKQ FIQYFLYQIM ----------
   128268  ---------- --VLAKRTYR ELKLLK---- -HLRH----E N--------- ---------- --VISLSDIF ISP------L EDI-YF---- ---------- ----VTELLG TDLHRLL--T SR---PLEKQ FIQYFLYQIM ----------
   246689  ---------- --VLAKRTYR ELKLLK---- -HLRH----E N--------- ---------- --IISLSDIF ISP------L EDI-YF---- ---------- ----VTELLG TDLHRLL--T SR---PLEKQ FIQYFLYQIM ----------
  1081594  ---------- --VLAKRTYR ELKLLK---- -HLRH----E N--------- ---------- --VISLSDIF ISP------L EDI-YF---- ---------- ----VTELLG TDLHRLL--T SR---PLEKQ FIQYFLYQIM ----------
   603877  ---------- --VLSKRTYR ELKLLK---- -HLKH----E N--------- ---------- --VISLSDIF ISP------L EDI-YF---- ---------- ----VTELLG TDLHRLL--T SR---PLEKQ FIQYFLYQIL ----------
   140829  ---------- --VLSKRTYR ELKLLK---- -HLKH----E N--------- ---------- --VISLSDIF ISP------L EDI-YF---- ---------- ----VTELLG TDLHRLL--T SR---PLEKQ FIQYFLYQIL ----------
   327107  ---------- --VLSKRTYR ELKLLK---- -HLRH----E N--------- ---------- --IISLSDIF ISP------L EDI-YF---- ---------- ----VTELLG TDLHRLL--T SR---PLEKQ FIQYFLYQIL ----------
   329694  ---------- --VLSKRTYR ELKLLK---- -HLRH----E N--------- ---------- --IISLSDIF ISP------L EDI-YF---- ---------- ----VTELLG TDLHRLL--T SR---PLEKQ FIQYFLYQIL ----------
   393439  ---------- --VLSKRTYR ELKLLK---- -HLRH----E N--------- ---------- --IISLSDIF ISP------L EDI-YF---- ---------- ----VTELLG TDLHRLL--T SR---PLEKQ FIQYFLYQIL ----------
    38942  ---------- --VLAKRTYR ELKLLK---- -HLRH----E N--------- ---------- -VIISLSDIF ISP------L EDI-YF---- ---------- ----VTELLG TDLHRLL--T SR---PLEKQ FVQYFLYQIL ----------
    87588  ---------- --VLSKRTYR ELKLLK---- -HLQH----E N--------- ---------- --IISLSDIF ISP------L EDI-YF---- ---------- ----VTELLG TDLHRLL--T SR---PLEKQ FIQYFLYQIL ----------
   443298  ---------- --VLAKRTYR ELKLLK---- -HLRH----E N--------- ---------- --VISLSDIF ISP------L EDI-YF---- ---------- ----VTELLG TDLHRLL--T SR---PLEKQ FIQYFLYQIM ----------
    53587  ---------- --VLSKRTYR ELKLLK---- -HLKH----E N--------- ---------- --VISLSDIF ISP------L EDI-YF---- ---------- ----VTELLG TDLHRLL--T SR---PLEKQ FIQYFLYQIL ----------
    70072  ---------- --VLAKRTYR ELKLLK---- -HIRH----E N--------- ---------- --IISLSDIF ISP------L EDI-YF---- ---------- ----VTELLG TDLHRLL--T SR---PLEKQ FIQYFLYQIL ----------
    13822  ---------- --VLSKRTYR ELKLLK---- -HLQH----E N--------- ---------- --VISLNDIF ISP------L EDI-YF---- ---------- ----VTELLG TDLHRLL--T SR---PLEKQ FIQYFLYQIL ----------
    25364  ---------- --VLSKRTYR ELKLLK---- -HLKH----E N--------- ---------- -VIISLSDIF ISP------L EDI-YF---- ---------- ----VTELLG TDLHRLL--T SR---PLEKQ FIQYFLYQIL ----------
   116576  ---------- --VLSKRTYR ELKLLK---- -HLRH----E N--------- ---------- --IISLSDIF ISP------L EDI-YF---- ---------- ----VTELLG TDLHRLL--T SR---PLEKQ FIQYFLYQIL ----------
   103311  ---------- --VLAKRTYR ELKLLK---- -HLRH----E N--------- ---------- --LITLDDIF LSP------L EDI-YF---- ---------- ----VTELQG TDLHRLL--T SR---PLEKQ FIQYFTYQIL ----------
    26173  ---------- --VLAKRTYR ELKLLK---- -HLKH----E N--------- ---------- --LITLDDIF LSP------L EDI-YF---- ---------- ----VNELQG TDLHRLL--T SR---PLEKQ FIQYFTYQIL ----------
    55352  ---------- --VLAKRTYR ELKLLK---- -HLKH----E N--------- ---------- --LITLDDIF LSP------L EDI-YF---- ---------- ----VNELQG TDLHRLL--T SR---PLEKQ FIQYFTYQIL ----------
   125207  ---------- --VLAKRTYR ELKLLK---- -HLRH----E N--------- ---------- --LICLQDIF LSP------L EDI-YF---- ---------- ----VTELQG TDLHRLL--Q TR---PLEKQ FVQYFLYQIL ----------
    67307  ---------- --VLAKRTYR ELKLLK---- -HLRH----E N--------- ---------- --LICLQDIF LSP------L EDI-YF---- ---------- ----VTELQG TDLHRLL--Q TR---PLEKQ FVQYFLYQIL ----------
   185764  ---------- --VLAKRTYR ELKLLK---- -HLRH----E N--------- ---------- --LITLEDIF LSP------L EDI-YF---- ---------- ----VTELQG TDLHRLL--T SR---PLEKQ FIQYFLYQIL ----------
    99366  ---------- --VLAKRTYR ELKLLK---- -HLRH----E N--------- ---------- --LITLEDIF LSP------L EDI-YF---- ---------- ----VNELQG TDLHRLL--T SR---PLEKQ FIQYFLYQII ----------
    50111  ---------- --VLSKRTYR ELKLLK---- -HIRH----E N--------- ---------- --IIGLSDIF ISP------S EDI-YF---- ---------- ----VTELLG TDLHRLL--T AR---PLEKQ FIQYFLYQIL ----------
    87970  ---------- --VLAKRTYR ELKLLK---- -HIRH----E N--------- ---------- --IISLSDIF ISP------L EDI-YF---- ---------- ----VTELLG TDLHRLL--T SR---PLEKQ FIQYFLYQIL ----------
   107532  ---------- --VLAKRTYR ELKLLK---- -HIRH----E N--------- ---------- --IISLSDIF ISP------L EDI-YF---- ---------- ----VTELLG TDLHRLL--T SR---PLEKQ FIQYFLYQIL ----------
   604754  ---------- --VLAKRTYR ELKLLK---- -HIQH----E N--------- ---------- --IISLSDVF ISP------L EDI-YF---- ---------- ----VTELLG TDLHRLL--T SR---PLEKQ FIQYFLYQIL ----------
   633130  ---------- --VLAKRTYR ELKLLK---- -HIQH----E N--------- ---------- --IISLSDVF ISP------L EDI-YF---- ---------- ----VTELLG TDLHRLL--T SR---PLEKQ FIQYFLYQIL LSKMQ-----
   181927  ---------- --VLSKRTYR ELKLLK---- -HLKH----E N--------- ---------- --IISLSDIF ISP------L DDI-YF---- ---------- ----VTELLG TDLHRLL--T FR---PLDKQ FVQYFLYQIL ----------
    69161  ---------- --VLAKRTYR ELKLLK---- -HLRH----E N--------- ---------- --IISLLDVF ISP------G EDI-YF---- ---------- ----ITELLG TDLHRLL--S SR---PLERQ FVQYFLYQML ----------
    35549  ---------- --VLSKRTYR ELKLLK---- -HIRH----E N--------- ---------- --IISLNDIF ISP------L EDI-YF---- ---------- ----VTELLG TDLHRLL--T MR---PLEKQ FIQYFLYQIL ----------
   230139  ---------- --VLSKRTYR ELKLLK---- -HLRH----E N--------- ---------- --IISLSDIF ISP------L EDI-YF---- ---------- ----VTELLG TDLHRLL--T SR---PLEKQ FIQYFLYQIL ----------
   193325  ---------- --VLSKRTYR ELKLLK---- -HLRH----E N--------- ---------- --IISLSDIF ISP------L EDI-YF---- ---------- ----VTELLG TDLHRLL--T SR---PLEKQ FIQYFLYQIL ----------
   430049  ---------- --VLSKRTYR ELKLLK---- -HLRH----E N--------- ---------- --IISLSDIF ISP------L EDI-YF---- ---------- ----VTELLG TDLHRLL--T SR---PLEKQ FIQYFLYQIL ----------
   479652  ---------- --VLSKRTYR ELKLLK---- -HLRH----E N--------- ---------- --IISLSDIF ISP------L EDI-YF---- ---------- ----VTELLG TDLHRLL--T SR---PLEKQ FIQYFLYQIL ----------
   986652  ---------- --VLSKRTYR ELKLLK---- -HLKH----E N--------- ---------- --VISLSDIF ISP------L EDI-YF---- ---------- ----VTELLG TDLHRLL--T SR---PLEKQ FIQYFLYQIL ----------
   295607  ---------- --VLSKRTYR ELKLLK---- -HLKH----E N--------- ---------- --VISLSDIF ISP------L EDI-YF---- ---------- ----VTELLG TDLHRLL--T SR---PLEKQ FIQYFLYQIL ----------
   114099  ---------- --VLSKRTYR ELKLLK---- -HLRH----E N--------- ---------- --VISLSDIF ISP------L EDM-YV---- ---------- ----VTELLG TDLHRLL--T SR---PLEKQ FIQYFLYQIL ----------
   265170  ---------- --VLSKRTYR ELKLLK---- -ALTH----E N--------- ---------- --IISLSDIF ISP------L EDI-YF---- ---------- ----VTELLG TDLHRLL--T SR---PLERQ FIQYFLYQIL ----------
    41804  ---------- --VLSKRTYR ELKLLK---- -HSKH----E N--------- ---------- --LISLSDIF ISP------S EDI-YF---- ---------- ----VTELLG TDLHRLL--T SR---PLERQ FIQYFLYQIL ----------
   500846  ---------- --VLSKRTYR ELKLLK---- -HIQH----E N--------- ---------- --IISLSDVF ISP------L EDL-YF---- ---------- ----VTELLG TDLHRLL--T SR---PLEKQ FIQYFLYQIL ----------
   341622  ---------- --VLSKRTYR ELKLLN---- -HLRH----E N--------- ---------- --VISLSNIF ISP------L EDI-YL---- ---------- ----VTELLR TDLQRLL--T SR---PLEKQ FIQYFLYQIL ----------
  1545705  ---------- --VLSKRTYR ELKLLK---- -HIQH----E N--------- ---------- --IISLSDVF ISP------L EDI-YF---- ---------- ----VTELLG TDLHRLL--T SR---PLEKQ FIQYFLYQIL ----------
    15276  ---------- --VLSKRTYR ELKLLK---- -HIQH----E N--------- ---------- --IISLSDVF ISP------L EDI-YF---- ---------- ----VTELLG TDLHRLL--T SR---PLEKQ FIQYFLYQIL ----------
   391543  ---------- --HIARHMFR EIKLLR---- -QLHH----E N--------- ---------- --IINLTDIF ISP------S EDI-YL---- ---------- ----VTELMA TDLNTIL--K AK---KVEDQ FAQYFMYQIM ----------
   346451  ---------- --HIARHMFR EIKLLR---- -QLHH----E N--------- ---------- --IINLTDIF ISP------S EDI-YL---- ---------- ----VTELMA TDLNTIL--K AK---KVEDQ FAQYFMYQIM ----------
   449365  ---------- --NIAKHMFR EVKLLK---- -RLQH----D N--------- ---------- --IHLNDIF ISP------S EEI-YL---- ---------- ----VTDLMA TDLHSLL--K AK---KVDNQ FTQYFLYQIM ----------
    54661  ---------- --V--DQIHG PAASGH---- -GCLW----A R--------- ---------- -LIISLSDIF ISP------L EDI-YF---- ---------- ----VTELLG TDLHRLL--T SR---PLEKQ FVQYFLYQIL ----------
   275309  ---------- --ALAKRTYR EVHLLS---- -HLRH----D N--------- ---------- --LINMTDIF ISP------S EDL-YI---- ---------- ----VTECIM TDLHHLLSAT SK---SLEGQ FTQFFTYQML ----------
   389883  ---------- --AVARRTYR EVHLIS---- -NLRH----D N--------- ---------- --LINMRDIF ISP------S EDL-YL---- ---------- ----VMDCVM TDLHQLIQSA KK---PLEGQ FIQFFTYQML ----------
    61624  ---------- --ASSKRTYR ELKLMR---- -HLKH----E N--------- ---------- --IISLIDVF ISP------L EDV-YI---- ---------- ----VTDLLD IDLQRLL--A LR---SLEKK FVQYFLYQIL ----------
   173235  ---------- --ASSKRTYR ELKLMR---- -HLKH----E N--------- ---------- --IISLIDVF ISP------L EDV-YI---- ---------- ----VTDLLD IDLQRLL--A LR---SLEKK FVQYFLYQIL ----------
    35612  ---------- --VLSKRTYR ELKLLK---- -HIRH----E N--------- ---------- --IISLNDIF ISP------L EDI-YF---- ---------- ----VTELLG TDLHRLL--T MR---PLEKQ FIQYFLYQIL ----------
    35597                                                                    MKILNDIF ISP------L EDI-YF---- ---------- ----VTELLG TDLHRLL--T MR---PLEKQ FIQYFLYQIL ----------
   391476  ---------- --EAAKRTFR EVKLLR---- -HLEH----D N--------- ---------- --VISLRDIF VSP------R DDI-YL---- ---------- ----VTDLMA TDLAHII--A SK---PIDDE FVKYICYQIM ----------
   335269  ---------- --EAAKRTFR EVKLLR---- -HLEH----D N--------- ---------- --VRAPLSAE PGP------K LTS-RL---- ---------- ----VTDLMA TDLAHII--A SK---PIDDE FVKYICYQIM ----------
   377162  ---------- --EAAKRTFR EVKLLR---- -HLEH----D N--------- ---------- --VISLRDIF VSP------R DDI-YL---- ---------- ----VTDLMA TDLAHII--A SK---PIDDE FVKYICYQIM ----------
   271149  ---------- --VISSFRRS RICMSE---- -AFR------ ---------- ---------- ------TSWW FTD------N ACS-YF---- ---------- ----VTELLG TDLHRLL--T SR---PLEKQ FIQYFLYQIL ----------
    89784                MYVGKR SCRPPS---- -RAHN----D S--------- ---------- -QLFRDRITW HRP------A PPA-YL---- ---------- ----AAS--G EAIYPVL--- SV---PDSGK FSICFIVLAL TC-VK-----
   114108  ---------- --QPGRRYQE DHEALQ---- -HPRP----L Q--------- ---------- ------ANIP RAE------A SQA-SE---- ---------- ----TRECHQ PQRYLHLPSR GH---VCRDR AARHGLAP-- ----------
   173207  ---------- -RLLTKRCLR EIKLL----- QHFN------ -NHPR----- ---------- --IIELFDMD IV---DVNNF NEI-YLVF-- ---------- ------NCMD ASLHDVIHSD K----PINTV QGQWILYQ-- ----------
   127887  ---------- -KLITKRCLR EIKLL----- QHFN------ -GHPR----- ---------- --IINLRALD IV---DYHDF NEV-YLIQ-- ---------- ------DCCD TTMADIIHSR Q----RLEPV HYQWFMYQ-- ----------
    29226  ---------- -KLVTKRCLR EIKLL----- QHLN------ -GHPR----- ---------- --IIGLRALD IV---DYSNF NEI-YLLQ-- ---------- ------ECCD TTMADVIHSK L----GLEPV HYQWFIYQ-- ----------
   167765  ---------- -KLITKRCLR EIKVL----- QHFN------ -GHPR----- ---------- --IIDLKALD IV---DPQNF NEV-YLIQ-- ---------- ------SCCD TTMADIIHSK L----ELEPV HYQWFMYQ-- ----------
   150384  ---------- -KLITKRCLR EIKVL----- QHFN------ -GHPR----- ---------- --IIDLKALD IV---DPQNF NEV-YLIQ-- ---------- ------SCCD TTMADIIHSK L----ELEPV HYQWFMYQ-- ----------
    57923  ---------- -KLITKRCLR EMKLL----- QHFN------ -GHPR----- ---------- --IIDLKSMD IV---DPERF NEV-YLIQ-- ---------- ------SCCD TTMADIIHSK L----QLEPV HYQWFMYQ-- ----------
    57928                       MKLL----- QHFN------ -GHPR----- ---------- --IIDLKSMD IV---DPERF NEV-YLIQ-- ---------- ------SCCD TTMADIIHSK L----QLEPV HYQWFMYQ-- ----------
   230955  ---------- -KLTTKRCLR EIKLL----- QHFN------ -GHPR----- ---------- --IIDIRELD IV---DHTNF NEI-YLAQ-- ---------- ------QCCD TTLADVIHSS Q----PLEEV HFQWFLYQ-- ----------
   172033  ---------- -KLTTKRCLR EIKLL----- QHFN------ -GHPR----- ---------- --IIDIRELD IV---DHTNF NEI-YLAQ-- ---------- ------QCCD TTLADVIHSS Q----PLEEV HFQWFLYQ-- ----------
    40306  ---------- -KILAKRALR ELKLL----- THFR------ -GHKN----- ---------- --ITCLYDMD I---VDTNNF NEI-YLYE-- ---------- ------ELME CDMHQIIRSQ Q----PLTDS HYQSFIYQ-- ----------
   693957  ---------- -KILAKRALR EIKLL----- QHFR------ -GHRN----- ---------- --ITCLYDMD I---PRPDNF NEA-YLYE-- ---------- ------ELME CDLAAIIRSG Q----PLTDA HFQSFIYQ-- ----------
   508525  ---------- -KILAKRALR EIKLL----- QHFR---EVL TSSVQ----- ---------- --ITCLYDMD I---PRPDNF NEC-YLYE-- ---------- ------ELME CDLAAIIRSG Q----PLTDA HFQSFIYQ-- ----------
   471145  ---------- -KILAKRALR EIKLL----- QHFR------ -DHRN----- ---------- --ITCLYDMD I---PRPDNF NEC-YLYE-- ---------- ------ELME CDLAAIIRSG Q----PLTDA HFQSFIYQ-- ----------
   480536  ---------- -KILAKRALR EIKLL----- QHFR------ -DHRN----- ---------- --ITCLYDMD I---PRPDNF NEC-YLYE-- ---------- ------ELME CDLAAIIRSG Q----PLTDA HFQSFIYQ-- ----------
   471745  ---------- -KILAKRALR EIKLL----- QHFR---GTD PVVSQ----- ---------- --ITCLYDMD I---PRPDNF NEC-YLYE-- ---------- ------ELME CDLAAIIRSG Q----PLTDA HFQSFIYQ-- ----------
   687176  ---------- -KILAKRALR EIKLL----- QHFR---GH- ---RN----- ---------- --ITCLYDMD I---PRPENF NEC-YLYE-- ---------- ------ELME CDLAAIIRSG Q----PLTDA HFQSFIYQ-- ----------
    34863  ---------- -KILAKRALR EIKLL----- QHFR------ -GHRN----- ---------- --ITCLYDMD I---PRPDNF NET-YLYE-- ---------- ------ELME CDLAAIIRSG Q----PLTDA HFQSFIYQ-- ----------
    57153  ---------- -KILAKRALR EIKLL----- QHFR------ -GHRN----- ---------- --ITCLYDMD I---PRPDNF NET-YLYE-- ---------- ------ELME CDLAAIIRSG Q----PLTDA HFQSFIYQ-- ----------
   147961  ---------- -KILAKRALR EVKLL----- QHFR------ -GHRN----- ---------- --ITCLYDLD I---PRMDNF NEC-YLYE-- ---------- ------ELME CDLAAIIRSG Q----PLTDA HFQSFIYQ-- ----------
   287354  ---------- -KILAKRALR EIKLL----- QHFR------ -GHRN----- ---------- --ITCLYDMD I---PKPDGF NET-YLYE-- ---------- ------ELME CDLAAIIRSG Q----PLTDA HFQSFIYQ-- ----------
   281614  ---------- -KILAKRALR EIKLL----- QHFR------ -EHRN----- ---------- --ITCLYDMD I---PRPDNF NEA-YLYE-- ---------- ------ELME CDLAAIIRSG Q----PLTDA HFQSFIYQ-- ----------
    29684  ---------- -KILAKRALR EIKLL----- QHFR------ -GHRN----- ---------- --ITCLYDMD I---PRPDNF NET-YLYE-- ---------- ------ELME CDLAAIIRSG Q----PLTDA HFQSFIYQ-- ----------
   144400  ---------- -KILAKRALR EIKLL----- QHFR------ -GHRN----- ---------- --ITCLYDMD I---PRPEMF NET-YLYE-- ---------- ------ELME CDLAAIIRSG Q----PLTDA HFQSFIYQ-- ----------
```

```
 136175 ---------- -KILAKRALR EIKLL----- QHFR------ -GHRN----- ---------- --ITCLYDMD I---PRPEMF NET-YLYE-- ---------- ------ELME CDLAAIIRSG Q----PLTDA HFQSFIYQ-- ----------
 126818 ---------- -KILAKRALR EIKLL----- QHFR------ -GHRN----- ---------- --ITCLYDMD I---PRPEMF NET-YLYE-- ---------- ------ELME CDLAAIIRSG Q----PLTDA HFQSFIYQ-- ----------
2138682 ---------- -KILAKRALR EIKLL----- QHFR------ -GHRN----- ---------- --ITCLYDMD I---PRPDNF NET-YLYE-- ---------- ------ELME CDLAAIIRSG Q----PLTDA HFQSFIYQ-- ----------
  48096 ---------- -KILAKRALR EIKLL----- QHFR------ -GHRN----- ---------- --ITCLYDMD I---PRPDNF NET-YLYE-- ---------- ------ELME CDLAAIIRSG Q----PLTDA HFQSFIYQ-- ----------
 179022 ---------- -KILAKRALR EIKLL----- QHFR------ -GHRN----- ---------- --ITCLYDMD I---PRPDNF NET-YLYE-- ---------- ------ELME CDLAAIVRSG Q----PLTDA HFQSFIYQ-- ----------
 778216 ---------- -KILAKRALR EIKLL----- QHFR------ -GHRN----- ---------- --ITCLYDMD I---PRPDNF NET-YLYE-- ---------- ------ELME CDLAAIIRSG Q----PLTDA HFQSFIYQ-- ----------
 665550 ---------- -KILAKRALR EIKLL----- QHFR------ -GHRN----- ---------- --ITCLYDMD I---PRPDNF NET-YLYEGI HFPAILVQWL ISA---ELME CDLAAIIRSG Q----PLTDA HFQSFIYQ-- ----------
 190738 ---------- -KILAKRALR EIKLL----- QHFR------ -GHRN----- ---------- --ITCLYDMD I---PRPDNF NET-YLYE-- ---------- ------ELME CDLAAIIRSG Q----PLTDA HFQSFIYQ-- ----------
1107049 ---------- -KILAKRALR EIKLL----- QHFR------ -GHRN----- ---------- --ITCLYDMD I---PRPDNF NET-YLYE-- ---------- ------ELME CDLAAIIRSG Q----PLTDA HFQSFIYQ-- ----------
 504269 ---------- -KILAKRALR EIKLL----- QHFR------ -GHRN----- ---------- --ITCLYDMD I---PRPDNF NET-YLYE-- ---------- ------ELME CDLAAIIRSG Q----PLTDA HFQSFIYQ-- ----------
 432134 ---------- -KILAKRALR EIKLL----- QHFR------ -GHRN----- ---------- --ITCLYDMD I---PRPDNF NET-YLYE-- ---------- ------ELME CDLAAIIRSG Q----PLTDA HFQSFIYQ-- ----------
  59068 ---------- -KILAKRALR EIKLL----- QHFR------ -GHRN----- ---------- --ITCLYDMD I---PRPDNF NET-YLYE-- ---------- ------ELME CDLAAIIRSG Q----PLTDA HFQSFIYQ-- ----------
 336185 ---------- -KILAKRALR EIKLL----- QHFR------ -GHRN----- ---------- --ITCLYDMD I---PRPDNF NET-YLYE-- ---------- ------ELME CDLAAIIRSG Q----PLTDA HFQSFIYQ-- ----------
 343155 ---------- -KILAKRALR EIKLL----- QHFR------ -GHRN----- ---------- --ITCLYDMD I---PRPDNF NET-YLYE-- ---------- ------ELME CDLAAIIRSG Q----PLTDA HFQSFIYQ-- ----------
 326648 ---------- -KILAKRALR EIKLL----- QHFR------ -GHRN----- ---------- --ITCLYDMD I---PRPDNF NET-YLYE-- ---------- ------ELME CDLAAIIRSG Q----PLTDA HFQSFIYQ-- ----------
 425863 ---------- -KILAKRALR EIKLL----- QHFR------ -GHRN----- ---------- --ITCLYDMD I---PRPDQF NET-YLYE-- ---------- ------ELME CDLAAIIRSG Q----PLTDA HFQSFIYQ-- ----------
 436998 ---------- -KILAKRALR EIKLL----- QHFR------ -GHRN----- ---------- --IACLYDMD I---PRPDNF NET-YLYE-- ---------- ------ELME CDLAAIIRSG Q----PLTDA HFQSFIYQ-- ----------
 257317 ---------- -KILAKRALR EIKLL----- QHFR------ -GHRN----- ---------- --ITCLYDMD I---PRPENF NET-YLYE-- ---------- ------ELME CDLAAIIRSG Q----PLTDA HFQSFIYQ-- ----------
 998070 ---------- -KILAKRALR EIKLL----- QHFR------ -GHRN----- ---------- --ITCLYDMD I---PRPDNF NEC-YLYE-- ---------- ------ELME CDLAAIIRSG Q----PLTDA HFQSFIYQ-- ----------
  49449 ---------- -KILAKRALR EIKLL----- QHFR------ -GHRN----- ---------- --ITCLYDMD I---PRPDNF NEC-YLYE-- ---------- ------ELME CDLAAIIRSG Q----PLTDA HFQSFIYQ-- ----------
 498377 ---------- -KILAKRALR EIKLL----- QHFR------ -GHRN----- ---------- --ITCLYDMD I---PRPDGF NET-YLYE-- ---------- ------ELME CDLAAIIRSG Q----PLTDA HFQSFIYQ-- ----------
 184715 ---------- -KILAKRALR EIKLL----- QHFR------ -GHRN----- ---------- --ITCLYDMD I---PRPDNF NET-YLYE-- ---------- ------ELME CDLAAIIRSG Q----PLTDA HFQSFIYQ-- ----------
  51223 ---------- -KILAKRALR EIKLL----- QHFRG----- --HRN----- ---------- --ITCLYDMD I---PRPENF NEV-YLYE-- ---------- ------ELME CDLAAIIRSG Q----PLTDA HFQSFIYQ-- ----------
  66669 ---------- -KILAKRALR EIKLL----- QHFR------ ---EL----- ---------- --MECDLAAI I---RRYSSS S--------- ---------- ---------- ----GNPTNG Q----PLTDA HFQSFIYQ-- ----------
 649898 ---------- -KILAKRALR ELKLL----- QHFRGMFDSL PTSDD----- ---------- --ITCLYDMD I---PKPDNF NEV-YLYE-- ---------- ------ELME CDLAAIIRSG Q----PLTDA HFQSFIYQ-- ----------
 253817 ---------- -KILAKRALR ELKLL----- QHFRG----- --HRN----- ---------- --ITCLYDMD I---PKPDNF NEV-YLYE-- ---------- ------ELME CDLAAIIRSG Q----PLTDA HFQSFIYQ-- ----------
 618068 ---------- -KILAKRALR EIKLL----- QHFR------ -GHRN----- ---------- --ITCLYDMD I---PRPDNF NET-YLYE-- ---------- ------ELME CDLAAIIRSG Q----PLTDA HFQSFIYQ-- ----------
 108847 ---------- -KILCKRALR EVKLL----- QFFR------ -GHKN----- ---------- --ITCLYDLD IVMDPVTGVF NDI-YLYE-- ---------- ------ELME CDMHQIIRSG Q----PLTDS HYQSFIYQ-- ----------
  33629 ---------- -KILCKRSLR ELKLL----- QFFR------ -GHKN----- ---------- --ITCLYDLD IIPNPLNGEF NEI-YLYE-- ---------- ------ELME CDMHQIIRSG Q----PLTDL HYQSFIYQ-- ----------
  54715 ---------- -KILCKRSLR ELKLL----- QFFR------ -GHKN----- ---------- --ITCLYDLD IIPNPLTGEF NEI-YLYE-- ---------- ------ELME CDMHQIIRSG Q----PLTDS HYQSFIYQ-- ----------
  88920 ---------- -SILTKRALR EIKLL----- RHFR------ -GHKN----- ---------- --ITCLYDME IVPDSRTGKW AEV-YLYE-- ---------- ------ECME ADLHAIIRSG Q----GLTDS HYQSFIYQ-- ----------
  51479 ---------- -KILCKRALR ELKLL----- NHFR------ -GHKN----- ---------- --ITCLYDMD IVD---PAHF NEV-YLYE-- ---------- ------ELME CDMHQIIRSG Q----PLTDA HYQSFVYQ-- ----------
  86943 ---------- -KILCKRALR ELKLL----- RHFR------ -GHKN----- ---------- --ITCLYDLD IIPDA-NMNF NEI-YLYE-- ---------- ------ELME CDMHQIIRSN Q----PLTDQ HYQSFVYQ-- ----------
 100835 ---------- -KILCKRALR ELKLL----- RHFR------ -GHKN----- ---------- --ITCLYDLD IVN---KNKF NEV-YLYE-- ---------- ------ELME CDMHQIIRSG Q----PLTDA HYQSFIYQ-- ----------
 319719 ---------- -TLLCKRSLR ELKLL----- RHFR------ -GHKN----- ---------- --ITCLYDMD IVFYP-DGSI NGL-YLYE-- ---------- ------ELME CDMHQIIKSG Q----PLTDA HYQSFTYQ-- ----------
 822494 ---------- -TLLCKRSLR ELKLL----- RHFR------ -GHKN----- ---------- --ITCLYDMD IVFYP-DGSI NGL-YLYE-- ---------- ------ELME CDMHQIIKSG Q----PLTDA HYQSFTYQ-- ----------
 161919 ---------- -TILAKRALR EVKLL----- KHFN------ -GHEN----- ---------- --ITSIIDMD I---VNLQDF NEI-YLVQ-- ---------- ------ELME ADLHQIIRSG Q----PLTDA HFQYFVYQ-- ----------
 158631 ---------- -TILAKRALR EVKLL----- KFFN------ -GHEN----- ---------- --ITSVLDMD I---VNLQDF NEI-YLVQ-- ---------- ------ELME ADLHQIIRSG Q----PLTDA HFQYFVYQ-- ----------
 115223 ---------- -TILAKRALR EVKLL----- KFFN------ -GHEN----- ---------- --ITSVLDMD I---VNLQDF NEI-YLVQ-- ---------- ------ELME ADLHQIIRSG Q----PLTDA HFQYFVYQ-- ----------
 344665 ---------- -NILAKRALR EVKLL----- RHFN------ -GHEN----- ---------- --ITSILDMD I---TNVQDF NEI-YLFQ-- ---------- ------ELME ADLHQIIRSE Q----PLTDA HFQYFIYQ-- ----------
 178050 ---------- -SILSKRALR EVKLL----- KHFN------ -GHEN----- ---------- --ITSIIDMD I---VNIHDF NEI-YLVQ-- ---------- ------ELME ADLHQIIRSG Q----PLTDS HFQYFIYQ-- ----------
 234927 ---------- -QILAKRALR EVKLL----- KHFN------ -GHEN----- ---------- --ITAILDMD I---HNVHDF NEI-YLVQ-- ---------- ------ELME ADLHQIIRSE Q----LLTDA HFQYFVYQ-- ----------
 328916 ---------- -TILTKRALR EVKLL----- QHFR------ -GHKN----- ---------- --ITCLFDMD I---VNPHDY NEI-YLYE-- ---------- ------ELME ADLHAIIRSG Q----PLTDA HYQSFTYQ-- ----------
 273327 ---------- -TILTKRALR EVKLL----- QHFR------ -GHKN----- ---------- --ITCLFDMD I---VNPHDY NEI-YLYE-- ---------- ------ELME ADLHAIIRSG Q----PLTDA HYQSFTYQ-- ----------
 338804 ---------- -TILTKRALR EVKLL----- QHFR------ -GHKN----- ---------- --ITCLFDMD I---VNPHDY NEI-YLYE-- ---------- ------ELME ADLHAIIRSG Q----PLTDA HYQSFTYQ-- ----------
 527701 ---------- -RILTKRCLR E-IKL---L- RHFR------ -GHKN----- ---------- --ITCLYDMD IVFRPD-GNF DEV-YLYE-- ---------- ------ELME ADLHAIIRSG Q----PLTDA HFQSFIYQ-- ----------
  81317 ---------- -AILTKRCLR E-IKL---L- HHFR------ -GHKN----- ---------- --ITCLYDMD IVFGPD-GDF DEV-YLYE-- ---------- ------ELME ADLHAIIRSG Q----PLSDA HFQSFLYQ-- ----------
 831329 ---------- -RILTKRCLR E-IRL---L- HHFR------ -GHKN----- ---------- --ITCLYDMD IVFQPN-GNF DEV-YLYE-- ---------- ------ELME ADLHAIIRSG Q----PLTDA HFQSFLYQ-- ----------
 676624 ---------- -RILTKRCLR E-IRL---L- HHFR------ -GHKN----- ---------- --ITCLYDMD IVFQAN-MNF DEV-YLYE-- ---------- ------ELME ADLHAIIRSG Q----PLTDA HFQSFLYQ-- ----------
 826941 ---------- -RILTKRCLR E-IRL---L- HHFR------ -GHKN----- ---------- --ITCLYDMD IVFQSS-GNF DEV-YLYE-- ---------- ------ELME ADLHAIIRSG Q----PLTDA HFQSFLYQ-- ----------
  77377 ---------- -RILTKRCLR E-IRL---L- HHFR------ -GHKN----- ---------- --ITCLYDMD IVFKPD-GNF DEV-YLYE-- ---------- ------ELME ADLHAIIRSQ Q----PLTDA HFQSFIYQ-- ----------
  59915 ---------- -RILTKRCLR E-IRL---L- HHFR------ -GHKN----- ---------- --ITCLYDMD IVFEPD-GNF GEV-YLYE-- ---------- ------ELME ADLHAIIRSG Q----PLSDA HFQSFLYQ-- ----------
 160641 ---------- -RILTKRCLR E-IKL---L- HHFR------ -GHKN----- ---------- --ITCLYDMD IVFRPD-GNF DEV-YLYE-- ---------- ------ELME ADLHAIIRSQ Q----PLSDA HFQSFIYQ-- ----------
 108839 ---------- -RILTKRCLR E-IKL---L- HHFR------ -GHKN----- ---------- --ITCLYDMD IVFRPD-GNF DEV-YLYE-- ---------- ------ELME ADLHAIIRSQ Q----PLSDA HFQSFIYQ-- ----------
1536187 ---------- -RILTKRCLR E-IKL---L- HHFR------ -GHKN----- ---------- --ITCLYDMD IVFHPD-GNF DEV-YLYE-- ---------- ------ELME ADLHAIIRSQ Q----PLSDA HFQSFIYQ-- ----------
 281708 ---------- -RILTKRCLR E-IKL---L- HHFR------ -GHKN----- ---------- --ITCLYDMD IVFHPD-GNF DEV-YLYE-- ---------- ------ELME ADLHAIIRSQ Q----PLSDA HFQSFIYQ-- ----------
 173791 ---------- -RILTKRCLR E-IKL---L- HHFR------ -GHKN----- ---------- --ITCLYDMD IVFHSD-GNF DEV-YLYE-- ---------- ------ELME ADLHAIIRSQ Q----PLSDA HFQSFIYQ-- ----------
  82703 ---------- -RILTKRCLR E-IKL---L- HHFR------ -GHKN----- ---------- --ITCLYDMD IVFRQD-GNF DEV-YLYE-- ---------- ------ELME ADLHAIIRSQ Q----PLSDA HFQSFIYQ-- ----------
 139538 ---------- -RILTKRCLR E-IRL---L- HHFR------ -GHKN----- ---------- --ITCLYDMD IVFQPN-GNF DEV-YLYE-- ---------- ------ELME ADLHAIIRSG Q----PLTDA HFQSFLYQ-- ----------
 479859 ---------- -RILTKRCLR E-IKL---L- HHFR------ -GHKN----- ---------- --ITCLYDMD IVFNSM-GDF DEV-YLYE-- ---------- ------ELME ADLHAIIRSG Q----PLSDA HFQSFLYQ-- ----------
 621303 ---------- -RILTKRCLR E-IRL---L- HHFR------ -GHKN----- ---------- --ITCLYDMD IVFQPN-GNF DEV-YLYE-- ---------- ------ELME ADLHAIVRSG Q----PLSDA HFQSFLYQ-- ----------
  38569 ---------- -RILTKRCLR E-IRL---L- HHFR------ -GHKN----- ---------- --ITCLYDMD IVFQPN-GNF DEV-YLYE-- ---------- ------ELME ADLHAIVRSG Q----PLSDA HFQSFLYQ-- ----------
 242842 ---------- -RILTKRCLR E-IKL---L- HHFR------ -GHKN----- ---------- --ITCLYDMD IVFEPN-GNF NEV-YLYE-- ---------- ------ELME ADLHAIIRSG Q----PLSDA HFQSFMYQ-- ----------
2481011 ---------- -RILTKRCLR E-IRL---L- HHFR------ -GHKN----- ---------- --ITCLYDMD IVFQPN-GNF DEV-YLYE-- ---------- ------ELME ADLHAIIRSG Q----PLTDA HFQSFLYQ-- ----------
 169913 ---------- -RILTKRCLR E-IKL---L- HHFR------ -RHKN----- ---------- --ITCLYDMD IVFDAS-GNF DEV-YLYE-- ---------- ------ELME ADLHAIIRSG Q----PLSDA HFQSFIYQ-- ----------
  71742 ---------- -RILTKRCLR E-IRL---L- HHFR------ -GHKN----- ---------- --ITCLYDMD IVFQPN-GNF DEV-YLYE-- ---------- ------ELME ADLHAIIRSG Q----PLTDA HFQSFVYQ-- ----------
  66635 ---------- -KILTKRALR E-LKL---L- RFFC------ -GHKN----- ---------- --ITCLYDMD IIDPHD---F QEV-YLYE-- ---------- ------ELME ADLHAIIRSG Q----PLSDA HFQSFIYQ-- ----------
  53870 ---------- -KILTKRALR E-LKL---L- RFFC------ -GHKN----- ---------- --ITCLYDMD IIDPHD---F QEV-Y--E-- ---------- ------ELME ADLHAIIRSG Q----PLSDA HFQSFIYQ-- ----------
  27246 --ASFLLT-- -YCTGATRMY I-PFSSRRR- MRST------ -DGVQ----- ---------- --ITCLYDMD IVFQPN-GNF DEV-YLYE-- ---------- ------ELME ADLHAIIRSG Q----PLTDA HFQSFLYQ-- ----------
  88978 LDAVIFTL-- -YATMNRTAD I-DEEMSPR- DQPL------ -QWAQ----- ---------- --ITCLYDMD IVFQPN-GNF DEV-YLYE-- ---------- ------ELME ADLHAIIRSG Q----PLTDA HFQSFVYQ-- ----------
1091096 ---------- -RILTKRCLR E-IRL---L- HHFR------ -GHKN----- ---------- --ITCLYDMD IVFQPN-GNF DEV-YLYE-- ---------- ------ELME ADLHAIIRSG Q----PLTDA HFQSFLYQ-- ----------
  66856 ---------- ---------- ----L---L- HHFR------ -GHKN----- ---------- --ITCLYDMD IVFHSD-GNF DEV-YLYE-- ---------- ------ELME ADLHAIIRSQ Q----PLSDA HFQSFIYQ-- ----------
  20720 ---------- -EDPHKEMFA R-DQTPPPL- SRTQ------ -EHCQ----- ---------- --ITCLYDMD IVFRQD-GNF DEV-YLYE-- ---------- ------ELME ADLHAIIRSQ Q----PLSDA HFQSFIYQ-- ----------
 124539 ---------- -RILTKRCLR E-IRL---L- HHFR------ -GHKN----- ---------- --ITCLYDMD IVFEPD-GNF GEV-YLYE-- ---------- ------ELME ADLHAIIRSG Q----PLSDA HFQSFLYQ-- ----------
 361187 ---------- -KILAKRALR E-IKL---L- QHFR------ -GHRN----- ---------- --ITCLYDMD IP-RPD--NF NET-YLYE-- ---------- ------ELME CDLAAIIRSG Q----PLTDA HFQSFIYQ-- ----------
 327531 ---------- -KILTKRCLR E-IRL---L- HHFR------ -GHKN----- ---------- --ITCLYDMD IVFEPN-NVF NEV-YLYE-- ---------- ------ELME ADLHAIIRSG Q----PLSDA HFQSFMYQ-- ----------
   7086 ---------- -KILTKRCLR E-IRL---L- HHFR------ -GHKN----- ---------- --ITCLYDMD IVFEPN-NVF NEV-YLYE-- ---------- ------ELME ADLHAI---- ---------- ---SFMYQ-- ----------
  28915 ---------- -AILAKRALR E-VKL---L- RHFS------ -GHEN----- ---------- --ITSILDMD ITNVQE---F NEI-YLVQ-- ---------- ------ELME ADLHQIIRSE Q----PLTDA HFQYFIYQ-- ----------
 669145 SRSVLGNR-- -TSHLKRTVE NPDEAMPAR- DQCT------ -YDAQ----- ---------- --ITCLYDMD IVFGSD-GNF DEV-YLYE-- ---------- ------ELME ADLHAIIRSG Q----PLSDA HFQSFIYQ-- ----------
  46685 ---------- -KILTKRALR E-LKL---L- HHFR------ -GHKN----- ---------- --ITCLYDMD II--SP-DTF TEV-YLYE-- ---------- ------ELME ADLHAIIRSG Q----PLSDA HFQSFIYQ-- ----------
  53954 ---------- -KILTKRALR E-LKL---L- HHFR------ -GHKN----- ---------- --ITCLYDLD LV--DP-VNF DSV-YLYE-- ---------- ------ECME ADLHAIIRSG Q----PLSDA HFQSFIYQ-- ----------
1446322 ---------- -RILMKRCLR E-ISL---L- VHFR------ -GHKNV-IEV VFLLVVDLPV CKITCLYDMD IVFQPN-GNF DEV-YLYE-- ---------- ------ELME ADLHAI---- ---------- ----------
 351754 ---------- -RILTKRCLR E-IRL---L- HHFR------ -GHKN----- ---------- --ITCLYDMD IVFQMN-GNF DEV-YLYE-- ---------- ------ELME ADLHAI---- ---------- ----------
  65873 ---------S KTILTKRALR EIK-----LL HHFR------ -GHKN----- ---------- --ITCLYDMD IV--DY-SNF NEV-YLYE-- ---------- ------ELME ADLHAIIRSG Q----PLTDQ HYQSFVYQ-- ----------
  52469 ---------- QTILTKRALR EIK-----LL HHFR------ -GHKN----- ---------- --ITCLYDMD IV--DY-SNF NEV-YLYE-- ---------- ------ELME ADLHAIIRSG Q----PLTDQ HYQSFVYQ-- ----------
  30002 FLLSYILTQQ QTILTKRALR EIKREATSVV RLSN------ -FDSK----- ---------- --ITCLYDMD IV--DY-SNF NEV-YLYE-- ---------- ------ELME ADLHAIIRSG Q----PLTDQ HYQSFVYQ-- ----------
 154156 ---------- -PILTKRALR ELKLL----- EHFN------ -GHKN----- ---------- --IIGLLDMD IV---DYSDF NEL-YLYQ-- ---------- ------ELME ADLHQIIRSG Q----PLTDE HYQSFIYQ-- ----------
 166841 ---------- -PILTKRALR ELKLL----- EHFN------ -GHKNVGREE VGYRRWLTRW MQIIGLLDMD IV---DYSDF NEL-YLYQ-- ---------- ------ELME ADLHQIIRSG Q----PLTDE HYQSFIYQ-- ----------
 238118 ---------- -PILTKRALR EVKLL----- EHFN------ -GHKN----- ---------- --IIGLYDID IV---DYNNF NEI-YLYQ-- ---------- ------ELME ADLHQIIQSR Q----PLTDE HFQSFIYQ-- ----------
 143069 ---------- -PILTKRALR EVKLL----- EHFN------ -GHKN----- ---------- --IIGLYDID IV---DYNNF NEI-YLYQ-- ---------- ------ELME ADLHQIIQSR Q----PLTDE HFQSFIYQ-- ----------
 456995 ---------- RR-------- ---LL----- HHFR------ -GHKN----- ---------- --ITCLYDMD IVFQPN-GNF DEV-YLYE-- ---------- ------ELME ADLHAIIRSG Q----PLTDA HFQSFLYQ-- ----------
 175880 ---------- LRILTKRCLR EIKLL----- HHFR------ -GHKN----- ---------- --ITCLYDMD IVFEPN-GNF NEV-YLYE-- ---------- ------ELME ADLHAIIRSG Q----PLSDA HFQSFMYQ-- ----------
  28309 ---------- -KLSCKRTLR ELKLL----- RHLR------ -GHPN----- ---------- --IVWLFDTD IVFYPN-GAL NGV-YLYE-- ---------- ------ELME CDLSQIIRSE Q----RLEDA HFQSFIYQ-- ----------
 767958 ---------- -KLSCKRTLR ELKLL----- RHLR------ -GHPN----- ---------- --IVWLFDTD IVFYPN-GAL NGV-YLYE-- ---------- ------ELME CDLSQIIRSE Q----RLEDA HFQSFIYQ-- ----------
 126152 ---------- -KLSCKRTLR ELKLL----- RHLR------ -GHPN----- ---------- --IVWLFDTD IVFYPN-GAL NGV-YLYE-- ---------- ------ELME CDLSQIIRSE Q----RLEDA HFQSFIYQ-- ----------
 613191                                                                          MD I---PRPDNF NET-YLYE-- ---------- ------ELME CDLAAIIRSG Q----PLTDA HFQSFIYQ-- ----------
 198058 ---------- -KSPMSSARR FWRSVLCARL SSFN------ -TSGAI---- ---------- --EISPVCTT W---TFPDPT TSTRLICM-- ---------- ------KLME CDLAAIIRSG Q----PLTDA HFQSFIYQ-- ----------
 123006                MFLAKR SWRSALCAKS SCSS------ -TSGAT---- ---------- --ITCLYDMD I---PRPDNF NET-YLYE-- ---------- ------ELME CDLAAIIRSG Q----PLTDA HFQSFIYQ-- ----------
 178993 ---------- -RSRMSSAKR SWPSAPCVRS SCCS------ -TSGATAIRV S--------- LQITCLYDMD I---PRPDNF NET-YLYE-- ---------- ------ELME CDLAAIVRSG Q----PLTDA HFQSFIYQ-- ----------
 122967                                                                          MT W---TFLDPI TSTRHIST-- ---------- ------RLME CDLAAIIRSG Q----PLTDA HFQSFIYQ-- ----------
 120501                                                                                                                      ME CDLAAIIRSG Q----PLTDA HFQSFIYQ-- ----------
  99018                MASSAR LPTTRRARLL QHFR------ -GHRN----- ---------- --ITCLYDMD I---PRPDNF NEC-YLYE-- ---------- ------ELME CDLAAIIRSG Q----PLTDA HFQSFIYQ-- ----------
 213678                MFLARR FSPSARSARS SYF------- -SISE----- ---------- --ITCLYDMD I---PRPDNF NEC-YLYE-- ---------- ------ELME CDLAAIIRSG Q----PLTDA HFQSFIYQ-- ----------
 460523                                                                          MD I---PRPDNF NEC-YLYE-- ---------- ------ELME CDLAAIIRSG Q----PLTDA HFQSFIYQ-- ----------
 625621                                                                          MD I---PKPDNF NEV-YLYE-- ---------- ------ELME CDLAAIIRSG Q----PLTDA HFQSFIYQ-- ----------
 265871                                                           M----- ---------- --ITCLYDMD I---PRPDNF NET-YLYE-- ---------- ------ELME CDLAAIIRSG Q----PLTDA HFQSFIYQ-- ----------
  53464                                                                                                                      ME CDLAAIIRSG Q----PLTDA HFQSFIYQ-- ----------
 105414                                                                          MD I---PRPDNF NET-YLYE-- ---------- ------ELME CDLAAIIRSG Q----PLTDA HFQSFIYQ-- ----------
 291876                                                                                                                      ME CDLAAIIRSG Q----PLTDA HFQSFIYQ-- ----------
  50744                                                                          MD PVT----GVF NDI-YLYE-- ---------- ------ELME CDMHQIIRSG Q----PLTDS HYQSFIYQ-- ----------
 863837                                                                          MD ITNV---QEF NEI-YLVQ-- ---------- ------ELME ADLHQIIRSE Q----PLTDA HFQYFIYQ-- ----------
  81355                                                                          MD IVFGPD-GDF DEV-YLYE-- ---------- ------ELME ADLHAIIRSG Q----PLSDA HFQSFLYQ-- ----------
 152896                                                                          MD IVFAPD-GNF DEV-YLYE-- ---------- ------ELME ADLHAIIRSQ Q----PLSDA HFQSFIYQ-- ----------
1196002                                                                          MD IVFQPN-GNF DEV-YLYE-- ---------- ------ELME ADLHAIIRSG Q----PLSDA HFQSFLYQ-- ----------
1195972                                                                     MSHVSVT VINSRS-QRL HTY-PRQE-- ---------- ------ELME ADLHAIIRSG Q----PLSDA HFQSFLYQ-- ----------
  49917 ---------- -IVLLRRAVR ELKLM----- RHLR------ -GHAN----- ---------- --IISLLDLD LV---DSKPY HGL-YCYQ-- ---------- ------ELMD CDLSKIIRSS I----QFTEI HIQSFIYK-- ----------
 116823 ---VIQT--- ICSENVS
 271167 ---VIHT--- IANPEIRSVS SQA
 151286 ---VIKT--- ICSETTLRFV KSLPKREPSH MEHDLAALST PSSY------ ---------- LSPLHCRLTT EAAYDLQMHR RLSFSEYTFR FAYGVA---- ----RFGW
  97820 ---VINT--- IASENP---- -TSSCEDATI CQVATQAGTP ASEE------ ---------- QVQECGPWCD RHARENARLR PEKEDNGHGG FGARIP---- ----RPLP
 167647 ---VINT--- IASENPLLGF YSCSSRILTC CSYRSSGAHA CLRP------ ---------- QEARNSRRGP GPRVPGPLSR PDRRAHRRGE IRLELQ---- ----RCRSAC RHMEDNDVSL QRSRRSPH
 430394 ---VINT--- IASENTLRFV RSLPKRERQP LKNKFRNADP PGVF------ ---------- MLGTFYSSGC
 266211 ---VINT--- IASENPSTFW SACSSSTPKS E
 266223 ---PIR---- ----RPSTFW SACSSSTPKS E
 266189 ---PIR---- ----RPSTFW SACSSSTPKS E
 151328 ---VIKT--- ICSETTLRFP ITIPQMSRKR NPNSTGHSMT LICR
 800193 ---IIKK--- IGSDKAQAYV --RSLPIKKT VPFQKLM-PN ADPQ------ ---------- ALDLLEKTLT FDPTWRITVC EALEHPWLSS YHE--P---- ----DDEPEC PEKFDK-W-K EVEKLE-TLE QFREALWNEI EDFR------
  22041 ---IIKK--- IGSDKAQAYV --RSLPIKKT VPFQKLM-PN ADPQ------ ---------- ALDLLEKTLT FDPTWRITVC EALEHPWLSS YHE--P---- ----DDEPEC PEKFDK-W-K EVEKLE-TLE QFREALWNEI EDFR------
  22014 ---IIKK--- IGSDKAQAYV --RSLPIKKT VPFQKLM-PN ADPQ------ ---------- ALDLLEKTLT FDPTWRITVC EALEHPWLSS YHE--P---- ----DDEPEC PEKFDK-W-K EVEKLE-TLE QFREALWNEI EDFR------
  22020 ---IIKK--- IGSDKAQAYV --RSLPIKKT VPFQKLM-PN ADPQ------ ---------- ALDLLEKTLT FDPTWRITVC EALEHPWLSS YHE--P---- ----DDEPEC PEKFDK-W-K EVEKLE-TLE QFREALWNEI EDFR------
 448385 ---VIRR--- IGSEKAQAYV --RSLPIKRA VPFQKHL-PS ADAQ------ ---------- AIDLLTKMLS FDPSTRITVP DALSHPWLSS YHD--V---- ----NDEPEC LQPFEK-W-H EIEQLE-TLE QFREALWNEI EDYR------
 122008 ---VITR--- IGSERAQAYI --RSLPVKKR VPFHKIV-PT ADSA------ ---------- ALDLLEKMLA FDPAARITVP DALAHPWLSS YHD--E---- ----QDEPEC PIPFEK-W-R YIETLE-TID QFRDALWNEI QEYR------
 578295 ---IVKR--- IGSDKAQAYV --RSLPYRKT VPFRKLM-PS ADPL------ ---------- ALDLLAKMLA FDPQDRITVP QALEHPWLAS YHD--V---- ----TDEPEC SVPYEK-W-R DIEKLE-TID QYREALWNEI EEYR------
```

```
    139503  ---VIKR--- IGSEKAQAYV --RTLPIKKK VPFRKLV-PS ADPQ------ ---------- ALDLLEKMLS FDPSGRITVA EALEHPWLSG YHD--V---- ----NDEPEC PTKFDR-W-R DIEKLE-TIE QFREALWKEI EDYR------
   2468658  ---VISR--- IGSEKAQAYV --RSLPYKPR VPFNKVI-PS ADSQ------ ---------- AVDLLTKMLA FDPSDRISVV DSLAHPYLSA YHD--V---- ----TDEPDC PRVFDR-W-R EVEELE-TID DFREALWNEI EDYR------
   2215386  ---VISR--- IGSEKAQAYV --RSLPYKPR VPFNKVI-PS ADSQ------ ---------- AVDLLTKMLA FDPSDRISVV DSLAHPYLSA YHD--V---- ----TDEPDC PRVFDR-W-R EVEELE-TID DFREALWNEI EDYR------
     88816  ---VLNR--- IHSDKARAYV --KSLAFRKP VPYKELM-PS ADPL------ ---------- ALDLLEKLLA FDPVDRITVP EALEHPWLAQ YHD--V---- ----GDEPDC PVPYEK-W-R DIEKLT-TIE EFREALWNEI EEYR------
     88949  ---VLNR--- IHSDKARAYV --RSLAFRKP VPYKELM-PS ADPL------ ---------- ALDLLEKLLA FDPVDRITVP EALEHPWLAQ YHD--V---- ----GDEPDC PVPYEK-W-R DIEKLT-TIE EFREALWNEI EEYR------
     88917  ---VLNR--- IHSDKARAYV --KSLAFRKP VPYKELM-PS ADPL------ ---------- ALDLLEKLLA FDPVDRITVP EALEHPWLAQ YHD--V---- ----GDEPDC PVPYEK-W-R DIEKLT-TIE EFREALWNEI EEYR------
    206606  ---TMQR--- IGSEKAQAYV --RSLPFKPK TKMSDHF-PN ADPL------ ---------- ALDLLEKMLA FDPSDRITVQ QALAHPYLEA YHE--I---- ----GDEPEA PILTEK-W-N ALEEIE-SDF EYRKAIWKEV YEFR------
    123647  ---VIKR--- IGSEKAQAYV --RSLPFKKA EPLSNVI-PN ADAE------ ---------- ALEFVEKLIY FDPSQRPNAE EALAHPWLST YHD--E---- ----NDEPTC PTVFDR-W-R QIEKLE-TLE EYREALVKEV RDCR------
    143931  ---VIKR--- IGSEKAQAYV --RSLPFKKA EPLSNVI-PN ADAE------ ---------- ALEFVEKLIY FDPSQRPNAE EALAHPWLST YHD--E---- ----NDEPTC PTVFDR-W-R QIEKLE-TLE EYREALVKEV RDCR------
    165009  ---TIER--- IGSSRAQAYV --RSLPFKKT VPFSKVL-PL ADAQ------ ---------- AVDLLTRMLT FDPAERITVS DALEHPWLAS YHD--V---- ----SDEPEC AEKFER-W-K TLEALE-TLE QYKETLYNEI QDYR------
    580315  ---VITR--- IASPKAQAYL --RSLPKKRK VPFEKII-PT ADPQ------ ---------- AVDLLTKLLI FDPSRRITVQ QALEHPWLSA YHD--V---- ----SDEPDC PEVFER-W-R DIEKLE-TLP QFRDAIWNEI QDYR------
    492840  ---VVTR--- IASPKAQAYL --RSLPYKKR VPFEKLI-PT ADPQ------ ---------- AVDLLTKLLV FDPASRITVQ QALEHPWLAA YHD--V---- ----SDEPSC PEVFER-W-R DIEKLE-TLP EFKEAIWNEI QDYR------
    594182  ---VVSR--- IASPKAQAYM --RSLPLKRK VPFEKLI-PT ADPQ------ ---------- AVDLLEKLLK FDPAERITVV DALEHPWLSA YHD--V---- ----SDEPEC PTKFER-W-R EIEELE-TLP QFRDAIWNEI QDYR------
    168238  ---VITR--- IASPKAQAYI --RSLPIKKR VPFEKLI-PT ADAQ------ ---------- AIDLLTKLLQ FDPAARITVA EALEHPWLSA YHD--V---- ----SDEPAC AAPFEG-W-R QIEELE-TLP QFREAIYNEI HDYR------
    107302  ---VITR--- IASPKAQAYI --RSLPIKKR VPFEKLI-PT ADAQ------ ---------- AIDLLTKLLQ FDPAARITVA EALEHPWLSA YHD--V---- ----SDEPAC AAPFEG-W-R QIEELE-TLP QFREAIYNEI HDYR------
     64718  ---VIRR--- IGSERAQAYI --RSLPFKKT VPFHKLL-PN ADPQ------ ---------- AIDLLAKMLT FDPAARMTVP EALEHPWLSS YHD--I---- ----DDEPSC PAKFTR-W-R EVEELE-TIE QFRDAIWKEI QDYR------
     64830  ---VIRR--- IGSERAQAYI --RSLPFKKT VPFHKLL-PN ADPQ------ ---------- AIDLLAKMLT FDPAARMTVP EALEHPWLSS YHD--I---- ----DDEPSC PAKFTR-W-R EVEELE-TIE QFRDAIWKEI QDYR------
   1154823  ---VIRR--- IGSERAQAYI --RSLPFKKT VPFHKLL-PN ADPQ------ ---------- AIDLLAKMLT FDPAARMTVP EALEHPWLSS YHD--I---- ----DDEPSC PAKFTR-W-R EVEELE-TIE QFRDAIWKEI QDYR------
    433002  ---IIHR--- IGSDKAQAYI --RSLPVKSG VPLRKLL-PT ADVQ------ ---------- ALDLLEKMIT FDPTSRIAVP DALAHPWLAS YHE--E---- ----TDEPEC PTKFER-W-R DIEKLE-TLE EFREALWKEI EDYR------
    822334  ---IIHR--- IGSDKAQAYI --RSLPVKSG VPLRKLL-PT ADVQ------ ---------- ALDLLEKMIT FDPTSRIAVP DALAHPWLAS YHE--E---- ----TDEPEC PTKFER-W-R DIEKLE-TLE EFREALWKEI EDYR------
    511572  ---VINR--- IGSDKAQLYI --RSLPVKKA IPLWKLL-PT ADFL------ ---------- ALDLLQKMLS FDPSARISVP DALAHPWLAS YHE--E---- ----TDEPDC PTTFEK-W-R DIEKLE-TLE DFRKALWEEI EDYR------
    688421  ---VIQR--- IGSDKAQIYV --RTLPVTKS VPFSRLL-PT ADYQ------ ---------- ALDLLEKMLS FDPLSRITVR EALSHPWLST YHD--D---- ----SDEPEC AKKFDK-W-R DIEKLE-TLE EFREAIWNEI QDYR------
     17778  ---VIQR--- IGSDKAQIYV --RTLPVTKS VPFSRLL-PT ADYQGNHVTF YCFARVDVGK ALDLLEKMLS FDPLSRITVR EALSHPWLST YHD--D---- ----SDEPEC AKKFDK-W-R DIEKLE-TLE EFREAIWNEI QDYR------
   1336823  ---IFDR--- ISAPKAKAYM --RSLPIRKA QPFCKVF-PK ADPQ------ ---------- AIDLLSKMIT WDPEHRLSVI EALEHPYVSN YHD--P---- ----DDEPSC PTKFDR-Y-K DHESLT-TLE EFRIAIWNEI QDFR------
    605623  ---VLKR--- IASDKARAYV --RSLPIRKK QPLNKVI-PN ADPQ------ ---------- ALDLLEKLLA FDPAARLTVE QAIAHPWLSA YHD--V---- ----NDEPTC PQNFDR-W-R DIEKLE-TLD DFRAALWAEI EDYR------
    511468  ---VINR--- IGSDKAQLYI --RSLPVKKA IPLWKLL-PT ADFL------ ---------- ALDLLQKMLS FDPSARISVP DALAHPWLAS YHE--E---- ----TDEPDC PTTFEK-W-R DIEKLE-TLE DFRKALWEEI EDYR------
     60300  ---VTKR--- IASDKAQAYV --RSLPFKPK KPLQTII-PS ADP------- ---------Q AIDLLTKMLA FDPVDRITVN EALEHPWLSS YHD--I---- ----KDEPVC DHKFEK-W-R QIEELE-TLD QFRDALWLEI EDYR------
   2435698  ---VISR--- IGSEKAQAYV --RSLPYKPR VPFNKVI-PS ADS------- ---------Q AVDLLTKMLA FDPSDRISVV DSLAHPYLSA YHD--V---- ----TDEPDC PRVFDR-W-R EVEELE-TID DFREALWNEI EDYR------
   1222436  ---VIQR--- IGSEKAQAYV --RSLPKIET VPLKCLI-PS ADL------- ---------Q AIDLLQKMLS FDPLSRITVH EALYHPWLAT YHE--E---- ----TDEPEC PNKFEK-W-K DIEKLE-TLE EFRQAIWNEI QDYR------
   1172380  ---VIQR--- IGSDKAQAYV --RSLPIKKA IPFRKLI-PT ADVL------ ---------- AIDLTKMLQ FDPSERINVI DALAHPWLAS YHD--V---- ----NDEPEC PEKFEK-W-R DIEKLE-TLD EFRDALWNEI EDYR------
   1151825  ---VIQR--- IGSDKAQAYV --RSLPIKKA IPFRKLI-PT ADVLGNFTLF NQRLFFTSTQ AIDLLTKMLQ FDPSERINVI DALAHPWLAS YHD--V---- ----NDEPEC PEKFEK-W-R DIEKLE-TLD EFRDALWNEI EDYR------
    410058  ---TMAR--- IGSPKAQAYV --RSLPLKKR RPFSKLL-PA AEP------- ---------E AVDLLTKLLT LDPDVRLTVV QALEHPWMSA YHEANV---- ----DDEPEC PTKFDR-W-R EQERLE-TFE ERRDALWTEI QEFR------
     57461  ---VIKR--- IGSEKAQAYV --RTLPIKKK VPFRKLV-PS ADP------- ---------Q AIDLLEKMLS FDPSGRITVA EALEHPWLSG YHD--V---- ----NDEPEC PTKFDR-W-R DIEKLE-TIE QFREALWKEI EDYR------
    311323  ---TLSR--- IGSPRAQEYV --RNLPYMPK IPFHTLF-PR ANPD------ ---------- AIDLLNKMLA FDPSSRIGVE EALEHRYLSI WHD--A---- ----TDEPNC PTKFDF-G-F EVV--D-DVS EMRHMILHEV RQFR------
    143102  ---TLRR--- VGSDRAQVYI --RSLPRMPR IPFQNLY-PR ANAL------ ---------- AIDLLNKLLE FDPSRRITVE EALAHPYLSA YHE--E---- ----DDEPSH NHTFDF-S-F EVV--D-SIV DMRRMIAQEV MSFK------
   2750129  ---TLRR--- VGSPRAQDYI --RSLPIKPR VPFATLF-PH ANPL------ ---------- AIDLLSKMLC FDPAKRISCE QALNHPYFQV WHD--P---- ----ADEPIC EAKFDF-G-F EEE--D-SIE GMKKLIIEEV KQFR------
   2702932  ---TLRR--- VGSPRAQDYI --RSLPIKPR VPFATLF-PH ANPL------ ---------- AIDLLSKMLC FDPAKRISCE QALNHPYFQV WHD--P---- ----ADEPIC EAKFDF-G-F EEE--D-SIE GMKKLIIEEV KQFR------
    108010  ---DFYA--- ISSLRSREYI --RALPFRKK KSFAQLF-PN ANPL------ ---------- AIDLLEKLLT FSPKRRIDVS EALRHPYLQP YHD--P---- ----QDEPTA DPIDPS-F-F DFD----FGD PMSKEDLKAI EGLSDWGIQG
   1754091  ---DFYA--- ITSQRSREYI --RALPFRKK KPFSTLF-PN ANPL------ ---------- AIDLMEKCLT FSPKRRIEVE DALKHPYLEA YHD--P---- ----QDEPTA EPLDPS-F-F DFD----NGD PLSKEQLKVM I-YEEIGVVG
    564944  ---EFYA--- ITTRRSRDYI --RALPFRKK KPFAQLF-PN ANPL------ ---------- AIDFLTRTLT FDPKKRITVE DALAHPYLEA YHD--P---- ----DDEPCA PPLDPE-F-F EFD--L-HKD DISREQLKEL L-YEEI----
    AtMPK18 ---TISG--- VRNDKARKYL --TEMRKKNP VTFSQKF-SK ADPL------ ---------- ALRLLQRLLA FDPKDRPTPA EALADPYFKG LSK--I---- ----EREPSS QQISKM-E-F EFERRRLTKD DIRELIYREI LEYH------
    AtMPK19 ---TIAG--- VRNEKARKYL --NEMRKKNL VPFSQKF-PN ADPL------ ---------- ALRLLQRLLA FDPKDRPTAE EALADPYFKC LAK--V---- ----EREPSC QPISKM-E-F EFERRRLTKD DIRELIYREI LEYH------
    AtMPK20 ---TISR--- VRNEKARRYL --TSMRKKPP IPFAQKF-PN ADPL------ ---------- SLKLLERLLA FDPKDRPTAE EALADPYFKG LAK--V---- ----EREPSC QPITKM-E-F EFERRKVTKE DIRELISREI LEYH------
  OsMPK20-1 ---TISR--- VRNDKARRYL --SSMRKKEP ILFSQKF-PS ADPL------ ---------- ALKLLQKLLA FDPKDRPTAE EALAHPYFKG LAK--V---- ----EREPSC QPITKM-E-F EFERRRVTKE DIRELIFREI LEYH------
  OsMPK20-4 ---TVTR--- IRNEKARRYL --SSMRKKQP VPFSERF-PK ADPA------ ---------- ALKLLQRLLA FDPKDRPTAE EALADPYFKG LAK--A---- ----EREPSC QPITKM-E-F EFERRKVTKE DVKELIFREI LEYH------
  OsMPK20-5 ---AISR--- IRNDKARRYL --SSMRRKQP VPFSEKF-PN VDPL------ ---------- ALKLLQRLLA FDPKDRPTAE EALADPYFKG LAK--V---- ----EREPSC QPISKM-E-F EFERRKVTKD DIKELIFREI LEYH------
  OsMPK20-3 ---AISQ--- VRNDKARKYL --TCMRKKQP ASFSHKF-LK ADPL------ ---------- ALQLLRKLLA FDPKDRPSAQ EALADPYFNG LAK--V---- ----EREPSC QPIPKM-E-F EFERRRATKE DIKELIFQEI LEYH------
    AtMPK16 ---AIGR--- VRNEKARRYL --SSMRKKKP IPFSHKF-PH TDPL------ ---------- ALRLLEKMLS FEPKDRPTAE EALADVYFKG LAK--V---- ----EREPSA QPVTKL-E-F EFERRRITKE DIRELIYRES LEYH------
  OsMPK16-1 ---TISR--- IRNEKARRYL --NSMRRKKP IPFTQKF-PN ADPL------ ---------- AMRLLERMLA FDPKDRPSAE EALADPYFKN IAN--V---- ----DREPSA QPITKL-E-F EFERRRITKE DIRELIYREI LEYH------
    AtMPK17 ---TLSR--- IRNEKARRYL --GNMRRKDP VPFTHKF-PN IDPV------ ---------- ALKLLQRLIA FDPKDRPSAE EALADPYFQG LAN--V---- ----DYEPSR QPISKL-E-F EFERRKLTRD DVRELMYREI LEYH------
     AtMPK8 ---SISR--- IRNEKARRYL --SSMRKKQP VPFSHKF-PK ADPL------ ---------- ALRLLERLLA FDPKDRASAE DALADPYFSG LSN--S---- ----EREPTT QPISKL-E-F DFERKKLVKD DVRELIYREI LEYH------
    AtMPK15 ---AISK--- IRNDKARRYL --GNMRKKQP VPFSKKF-PK ADPS------ ---------- ALRLLERLIA FDPKDRPSAE EALADPYFNG LSS--K---- ----VREPST QPISKL-E-F EFERKKLTKD DIRELIYREI LEYH------
  OsMPK17-1 ---TLSR--- IRNEKARRYL --STMRKKHA VPFSQKF-RN TDPL------ ---------- ALRLLERLLA FDPKDRSSAE EALADPYFAS LAN--V---- ----EREPSR HPISKL-E-F EFERRKLTKD DVRELIYREI LEYH------
     AtMPK9 ---AIAR--- IRNEKARRYL --GNMRRKPP VPFTHKF-PH ADPL------ ---------- ALHLLHRLLA FDPKDRPSAE EALADPYFYG LAN--V---- ----DREPST QPISKL-E-F EFERRKITKE DVRELIYREI LEYH------
  OsMPK21-1 ---SLAK--- IRNEKARRYL --SNMRKKPR VPFTKKF-PG VDPM------ ---------- ALHLLERLLA FDPKDRPSAE EALTDPYFNG LAN--S---- ----EREPIA QPISKL-E-F EFERKRLAKD DVRELIYREI LEYH------
  OsMPK21-2 ---TISR--- IRNEKARRYL --GNMRKKPR VPFSQKF-PG ADPM------ ---------- ALHLLERLLA FDPKDRPTAA EALTDPYFTG LAN--S---- ----EREPIA QPISKL-E-F EFERRKLAKD DVRELIYREI LEYH------
  OsMPK17-2 ---TLSR--- IRNENARGYL --TGMQRKHP IPFSHKF-HN ADPL------ ---------- ALRLLERLLA FDPKDRPTAE EALADPYFRG ISK--L---- ----EREPSR LPVSKF-E-F EFERRKLTKD DVREMIYREI LEYH------
     30260  ---------- ---DYAPAIL --SS------ --FYSDI-PK ---------- ---------- ALDLLEKMIT FDPTSRIAVP DALAHPWLAS YHE--E---- ----TDEPEC PTKFES-E-L EALSSLKRLG SCRAEVRTRC KQVP------
    130090  ---SFCDNPV ARRYAGRQFL --IQNPHVPK VDWAQIF-PE ANPD------ ---------- GLDLLDRLLQ FDPAKRITAA EALEHPYVSQ WRDPLL---- ----EQPCHA DVATKLTQ-F DYAQMSYDPQ TLKDLLYQEV MKAQ------
    599215  ---VVTR--- IASPKAQAYL --RSLPYKKR VPFEKLI-PT ADPQ------ ---------- AVDLLTKLLV FDPASRITVQ QALEHPWLAA YHD--V---- ----SDEPSC PEVFER-W-R DIEKLE-TLP EFKEAIWNEI QDYR------
    219362  ---ISIR--- RDWDLPG--- -----GERKA VNWSTVY-PF ASAD------ ---------- SLDLLGQLLK WEPTARLDVE QTIAHSFFKK IRD--I---- ----TCEPQS RS-VFN---F GKEENEDDLN KLHGLLKEEV AAFQ------
     56051  ---TVKK--- YNWKVEGLLW -----VKYKP VKWKSLY-PF APHE------ ---------- AINLLEKLLQ WDYKKRLEVE QILGHAFFRN TRN--A---- ----SDEPVS KE-LFD---F SFEGRANNIS QLKDLLELEV KHFK------
    139809  ---TVKK--- YNWKVEGLLW -----VKYKP VKWKSLY-PF APHE------ ---------- AINLLEKLLQ WDYKKRLEVE QILGHAFFRN TRN--A---- ----SDEPVS KE-LFD---F SFEGRANNIS QLKDLLELEV KHFK------
      1060  ---LVDR--- FSSKMAFDFF -GPPKPQYRA ISWDHIY-PF ASCD------ ---------- AHALMTNLLC WDAEKRYNVE RCLQDIFVSK CRD--L---- ----NNEPSC AR-PFD---F SFENQIESMN DLKKLLHEEV ESFK------
    184799  ---LVDR--- FSSKMAFDFF -GPPKPQYRA ISWDHIY-PF ASCD------ ---------- AHALMTNLLC WDAEKRYNVE RCLQDIFVSK CRD--L---- ----NNEPSC AR-PFD---F SFENQIESMN DLKKLLHEEV ESFK------
     68008  ---VLLK--- YGSGLIWELF -NSPNPQYKP IPWSQVY-PF ASKA------ ---------- AHDLLSHLIC WHSDSRYNVE EAISDPYLKS VRT--P---- ----DDEPIA RT-PFN---F EFETLATNLN DYKILIHEEV QQFK------
     99901  ---VLLK--- YGSGLIWELF -NSPNPQYKP IPWSQVY-PF ASKA------ ---------- AHDLLSHLIC WHSDSRYNVE EAISDPYLKS VRT--P---- ----DDEPIA RT-PFN---F EFETLATNLN DYKILIHEEV QQFK------
    104502  ---VLLK--- YGSGLIWELF -NSPNPQYKP IPWSQVY-PF ASKA------ ---------- AHDLLSHLIC WHSDSRYNVE EAISDPYLKS VRT--P---- ----DDEPIA RT-PFN---F EFETLATNLN DYKILIHEEV QQFK------
    645859  ---ILIK--- FGTIKAWNLG KNSNNPVYKK IPWSNIF-PF ASHE------ ---------- AINLIESLLH WDSTHRLNVE QAISHPFLNE VRK--P---- ----DDEPVC LQGPFD---F TYESELNSMS KLRDYLVEEV KNFK------
    599559  ---ILIK--- FGTIKAWNLG KNSNNPVYKK IPWSNIF-PF ASHE------ ---------- AINLIESLLH WDSTHRLNVE QAISHPFLNE VRK--P---- ----DDEPVC LQGPFD---F TYESELNSMS KLRDYLVEEV KNFK------
 36518:4-24 ---VINR--- NNWKFLL--- --PPRPQYRV SEWSQLY-PF ASKD------ ---------- GQELIESLLQ WDPEKRLSVH QAISHRFIEP VRD--S---- ----NSEALC E--VFD---F SFENRATTLG ELRELLEREV EMFK------
    178963  ---TLSR--- IGSPRAQEYV --RNLPYMAK KSFPSLF-PN ANPD------ ---------- ALDLLDRMLA FDPTRRISVE AALEHPYLAI WHD--A---- ----SDEPDC PTTFN----F DFEVVD-DVA EMRKMILDEV FRFR------
    196817  ---TLSR--- IGSPRAQEYV --RNLPFMPK KPFASLF-PN ANPD------ ---------- ALDLLDRMLA FDPSSRISVE QALEHPYLQI WHD--A---- ----SDEPDC PTTFDFDF-E VVE----DVG EMRKMILEER LAGS------
    738557  ---TLSR--- IGSPRAQDYV --RNLPYMQK VSFASLF-RQ ANPD------ ---------- ALDLLDRMLA FDPSSRISVE DALRHRYLHI WHD--A---- ----SDEPSC PTTFDFHF-E VVE----DIA EMKKMILAEA IYTK------
   1159263  ---TLRR--- VGSPRAQDYI --RSLPIKPR ISFATLY-PN ANPL------ ---------- AIDLLSQMLC FDPAKRISCE EALNHPYLQV WHD--P---- ----ADEPVC EKVPVVCR-H PLR----W
    176895  ---TLRR--- VGSPRAQDYI --RSLPIKPR VPFQTLY-PN ANPL------ ---------- AIDLLQQMLC FDPAKRISCE AALNHPYLQV WHD--P---- ----ADEPIC DTVSLISR-L KDK----VLK LS
    664529  ---T------ ---------- ---------- ---------- -NPL------ ---------- AIDLLAQMLC FDPAKRISCE QALNHPYFQV WHD--P---- ----ADEPVC ETKFDFGF-E EED----TIE GMKKLIVEEV TSFR------
   1194920  ---TLRR--- VGSPRVNFIF --QSA--VRV LTFYFIL-RP RIIF------ ---------- ALYQLSRASP FPLYTRMQIH LPSTYFPKCL HSI--P---- ----PNEFLA SKRSITHI-L LFG----MNR QTSPCATSPS TS-D------
    432994  ---STS
     98579
     88826  ---TFS
     54157  ---EFYA--- INSRRSRDYI --RALPFRKK RPFTAIY-PN ASAL------ ---------- AIDFLNKTLT FDPKKRLTVE EALQHPYLEA YHD--P---- ----EDEPVA PPLDEDFFAF DRQKDDISLI VFDFEVMRMR REGWIS
    914084  ---EFYA--- ITTRRSRDYI --RALPFRKK RPFAQLF-PN ASAL------ ---------- AVDFLTKTLT FDPKKRISVE MALCHPYLEA YHD--P---- ----DDEPVA PPLDPEFFEF DRTHLSSHTQ PAN
    503422  ---EFYA--- ITTRRSRDYI --RALPFRKK RPFAQIF-PN ASAL------ ---------- AVDFLTKTLT FDPKKRITVE EALAHPYLEA YHD--P---- ----DDEPVA PPLDPEFFEF DLHKDDISRE QLKELLYEEI MSFCPAPIT
     95205  ---EFYA--- ITSRRSRDYI --RALPIRKK RPFSAVF-PD ASPE------ ---------- ALDFLQKTLT FDPKKRLSVD QALEHPYLAA YHD--P---- ----EDEPAV APLDPEYFEF DYL--ELNKQ QLKELLYEEV QSFVPCI
    229298  ---EFYS--- ITSRRSRDYI --RALPIRKK KSFTTLF-PR ASQE------ ---------- AIDFLQKTLT FDQRKRMTVD EALEHPYLAT YHD--P---- ----EDEPVA PPLDPYFDF DEYKDDLTKA ELKELLYEEI MSFTPSI
    455126  ---EFYS--- ITSRRSRDYI --RALPIRRR KPMSQLF-PK ASPA------ ---------- AIDFLTKTLT FDPKKRLTVE ECLNHPYLEA YHD--P---- ----DDEPVV ESLEPEFFDF DFHKEEMSKD QLKELLYEEV TAFQSLL
    256867  ---EYAA--- ITSRRSRDYI --RALPIRKR KPFATLI-PS ASPA------ ---------- AIDFLSRTLT FDPKKRMTVD ECLRHPYLAP YYD--P---- ----ADEPCG PGLDENYFYF DLQKEELSKA QLKELLYNEV HSFTPAI
    419576  ---DFYA--- IQSHRSRDYL --RALPFNKR RPFSTIF-PN ANPL------ ---------- AIDLLDKCLT FNPKKRITVE EALAHPYLEP YHD--A---- ----DDEPNA DPLSIEFFAF DHSPTPLSRE ELKELIYEEI CH
     74409  ---DYYG--- IKSRRAREYI --RSLPFKKK IPFRALF-PK SNDL------ ---------- ALDLLEKMLA FNPAKRISVE DALRHPYLEP YHD--P---- ----EDEPTA PPIPEGFFDF DKNKDSLSKE QLKLLIYEEI MR
    521809  ---DYYG--- IKSRRAREYI --RSLPFKKK IPWKALF-PK TNDQ------ ---------- ALDLLERLLA FNPVKRITVE EALKHPYLEP YHD--P---- ----EDEPTA DPIPPEFFDF DKNKDNLTKE QLKVCQGDGR KFRRGNS
     54281  ---DLQS--- IKSKRAREYI --KSLPFYKK TPLRNLFGDH INPL------ ---------- GVDLVEKMLA FNTVNRITVE EALKHPYLRA FHV--P---- ----SDEPVA NPIPKDFFKF DLYKDQLSIS QLKMLLYEEI TKK
     48349  ---DLQS--- IKSKRAREYI --KSLPFYKK TPLRNLFGDH INPL------ ---------- GVDLVEKMLA FNTVNRITVE EALKHPYLRA FHV--P---- ----SDEPVA NPIPKDFFKF DLYKDQLSIS QLKMLLYEEI TKK
    594110  ---VVSR--- IASPKAQAYM --RSLPLKRK VPFEKLI-PT ADPQ------ ---------- GSSRPTSALI IMLMFIYSCG SS
     27538  ---DFYA--- ISSPRSREYI --RALPFRKR RDFKTMF-HG ANEL------ ---------- AIDFLEKCLT FSPKRRITAA EALRHPYFE- -----V---- ----NSLPAL PMLDLPLT-D AFDSPTTTRT TNLLLI
     96623  ---DFYA--- ITSQRSREYI --RALPFRKK KPFSTLF-PN ANPL------ ---------- AIDLMEKCIP RPSGRAYGRA AGSELL
    380926  ---DYYG--- IKSRRAREYI --RSLPFKKK --LLAFN-PV KRIT------ ---------- VEEALKHPYL EPYHDPDDEP TAPPIPEEF- -----F---- ----DFDKHK DNLSKEQLKQ LIYQEIMR
    263236  ---DYYG--- IKSRRAREYI --RSLPFKKK IPFRALF-PK SNDL------ ---------- ALDLSLISTR TRMR
    154671
     67562
    226444
   1137209  ---TVQV--- LCSF
    AtMPK13
    AtMPK11 ---TILF--- RFNLKKEH
    459393  ---EWP---- -GVTSLPDFK --PSFPKWQR SDLGSNVSSL DEE------- ---------- GLDLLDALLV YDPAGRISAK QALMHPYFID EP---V---- ----PQQPHS RTMVRNGFH
     69938  ---VIRR--- IGSEKAQAYV --RSLPIKRA VPFQKHL-PS ADAQ------ ---------- AIDLLTKMLS FDPSTRITVP DALSHPWLSS YHD--V---- ----NDEPEC LQPFE
    591714  ---ELTT--I PRPPPAYPHL --GRPRHPDD GGLLRHQVPP GS-------- ---------- GVHPLSALQE EGSLPNPVPQ DL
    107905  ---DFYG--I -KSRRVSEYM --NSVGMVCD SSYWHLG
     99046
    863830
    287494
    662188
    492594
    176887
    618168
    492643
    125995  ---SLSSKDA TMSTSSIRSC TSSAHHLNRH WLVSAVLVHR STCE------ -----ICHTC PRSPSTSSSP AQIQTLSTCS TACSHSTQAS ASASRK---- ----RLSTAT SPSGMMLLTS HLARRLSISH SRLSRTSARC DS
    122928  ---SL----- -LQRSRLRRP AQPDPAHPRY AQRGNPLAHR LP-------- ---------- PGAGIRPQPP LHGQEVFPVP VSQRQPGRAR PARSHA---- ----RLRPDP PHLGRGRPRA PVPGHLA-RR ERRARLPDDV QL
    154756
     68423
  OsMPK20-2
  Consensus     ....     ......r.lr e.kll..... ..f....... n....            .......d.. .........f ....y..... ...   .          ..elm. tdlh..i... ......l.d. h.qyf.yq.. ....

            901                                                                                                                                               1050
    127228  --------T LRALKAMHSA NVLHRDL--- ------KP-S NLLLNANCDL K--------- ---------- ---ICDLGLA R-------SS N-SAD----E NSGFMTEYVA TRWYRAPEIM LTFKEYTK-- ---------- ----------
    287090  --------T LRALKAMHSA NVLHRDL--- ------KP-S NLLLNANCDL K--------- ---------- ---ICDLGLA R-------SS N-SAD----E NSGFMTEYVA TRWYRAPEIM LTFKEYTK-- ---------- ----------
    186494  --------T LRALKAMHSA NVLHRDL--- ------KP-S NLLLNANCDL K--------- ---------- ---ICDLGLA R-------SA N-SAD----E NSGFMTEYVA TRWYRAPEIM LTFKEYTK-- ---------- ----------
    260272  --------T LRALKAMHSA NVLHRDL--- ------KP-S NLLLNANCDL K--------- ---------- ---ICDLGLA R-------SA N-SAD----E NSGFMTEYVA TRWYRAPEIM LTFKEYTK-- ---------- ----------
    119975  --------T LRALKAMHSA NILHRDL--- ------KP-S NLLLNANCDL K--------- ---------- ---ICDLGLA R-------SA N-SAD----E NSGFMTEYVA TRWYRAPEIM LTFKEYTK-- ---------- ----------
      2147  --------T LRALKAMHSA NVLHRDL--- ------KP-S NLLLNANCDL K--------- ---------- ---VCDFGLA R-------SA A-STD----D NQGFMTEYVA TRWYRAPEIM LTFKEYTK-- ---------- ----------
    692376  --------T LRALKAMHSA NVLHRDL--- ------KP-S NLLLNANCDL K--------- ---------- ---VCDFGLA R-------SA A-STE----D NSGFMTEYVA TRWYRAPEIM LTFKEYTK-- ---------- ----------
    698785  --------T LRALKAMHSA NVLHRDL--- ------KP-S NLLLNANCDL K--------- ---------- ---VCDFGLA R-------SA A-STE----D NSGFMTEYVA TRWYRAPEIM LTFKEYTK-- ---------- ----------
    468116  --------T LRALKAMHSA NVLHRDL--- ------KP-S NLLLNANCDL K--------- ---------- ---VCDFGLA R-------SA A-STE----D NSGFMTEYVA TRWYRAPEIM LTFKEYTK-- ---------- ----------
```

```
 274271  ---------T LRALKAMHSA NVLHRDL--- ------KP-S NLLLNANCDL K--------- ---------- ---VCDFGLA R-------SA A-STE----D NQGFMTEYVA TRWYRAPEIM LTFKEYTK-- ---------- ----------
 255486  ---------T LRALKAMHTA NVLHRDL--- ------KP-S NLLLNANCDL K--------- ---------- ---VCDFGLA R-------SA A-STE----D NQGFMTEYVA TRWYRAPEIM LTFKEYTK-- ---------- ----------
 285227  ---------T LRALKAMHSA NVLHRDL--- ------KP-S NLLLNANCDL K--------- ---------- ---VCDFGLA R-------SA A-STE----D NQGFMTEYVA TRWYRAPEIM LTFKEYTK-- ---------- ----------
  18186  ---------T LRALKAMHSA NVLHRDL--- ------KP-S NLLLNANCDL K--------- ---------- ---VCDFGLA R-------SA A-SQE----D NSGFMTEYVA TRWYRAPEIM LTFKEYTK-- ---------- ----------
 465108  ---------T LRALKAMHSA NVLHRDL--- ------KP-S NLLLNANCDL K--------- ---------- ---VCDFGLA R-------SA A-STD----D NSGFMTEYVA TRWYRAPEIM LTFKEYTK-- ---------- ----------
1164585  ---------T LRALKAMHSA NVLHRDL--- ------KP-S NLLLNANCDL K--------- ---------- ---VCDFGLA R-------SA A-SQE----D NSGFMTEYVA TRWYRAPEIM LTFKEYTK-- ---------- ----------
  93851  ---------T LRALKAMHSA NVLHRDL--- ------KP-S NLLLNANCDL K--------- ---------- ---VCDFGLA R-------SA A-STE----D NQGFMTEYVA TRWYRAPEIM LTFKEYTK-- ---------- ----------
 108267  ---------T LRALKAMHSA NVLHRDL--- ------KP-S NLLLNANCDL K--------- ---------- ---VCDFGLA R-------SA A-STE----D NQGFMTEYVA TRWYRAPEIM LTFKEYTK-- ---------- ----------
  59022  ---------T LRALKALHSA NVLHRDL--- ------KP-S NLLLNANCDL K--------- ---------- ---VCDFGLA R-------SA A-STD----D NSGFMTEYVA TRWYRAPEIM LTFKEYTK-- ---------- ----------
 370137  ---------T LRALKAMHSA NVLHRDL--- ------KP-S NLLLNANCDL K--------- ---------- ---VCDFGLA R-------SA A-STE----D NSGFMTEYVA TRWYRAPEIM LTFKEYTK-- ---------- ----------
 532216  ---------T LRALKAMHSA NVLHRDL--- ------KP-S NLLLNANCDL K--------- ---------- ---VCDFGLA R-------SA A-STE----D NSGFMTEYVA TRWYRAPEIM LTFKEYTK-- ---------- ----------
  31240  ---------T LRALKAMHSA NVLHRDL--- ------KP-S NLLLNANCDL K--------- ---------- ---VCDFGLA R-------SA A-STE----D NSGFMTEYVA TRWYRAPEIM LTFKEYTK-- ---------- ----------
 160345  ---------T LRALKAMHSA NVLHRDL--- ------KP-S NLLLNANCDL K--------- ---------- ---VCDFGLA R-------SA A-STE----D NSGFMTEYVA TRWYRAPEIM LTFKEYTK-- ---------- ----------
 212060  ---------T LRALKAMHSA NVLHRDL--- ------KP-S NLLLNANCDL K--------- ---------- ---VCDFGLA R-------SA A-STE----D NSGFMTEYVA TRWYRAPEIM LTFKEYTK-- ---------- ----------
 327005  ---------T LRALKAMHSA NVLHRDL--- ------KP-S NLLLNANCDL K--------- ---------- ---VCDFGLA R-------SA A-SME----D NSGFMTEYVA TRWYRAPEIM LTFKEYTK-- ---------- ----------
  48412  ---------T LRALKAMHSA NVLHRDL--- ------KP-S NLLLNANCDL K--------- ---------- ---VCDFGLA R-------SA A-STE----D NQGFMTEYVA TRWYRAPEIM LTFKEYTK-- ---------- ----------
 606069  ---------T LRALKAMHSA NVLHRDL--- ------KP-S NLLLNANCDL K--------- ---------- ---VCDFGLA R-------SA A-SQE----D NSGFMTEYVA TRWYRAPEIM LTFKEYTK-- ---------- ----------
 123724  ---------T LRALKAMHSA NVLHRDL--- ------KP-S NLLLNANCDL K--------- ---------- ---VCDFGLA R-------SA A-SQE----D NSGFMTEYVA TRWYRAPEIM LTFKEYTK-- ---------- ----------
 342035  ---------T LRALKAMHSA NVLHRDL--- ------KP-S NLLLNANCDL K--------- ---------- ---VCDFGLA R-------SA A-SQE----D NSGFMTEYVA TRWYRAPEIM LTFKEYTK-- ---------- ----------
2135321  ---------T LRALKAMHSA NVLHRDL--- ------KP-S NLLLNANCDL K--------- ---------- ---VCDFGLA R-------SA A-SQE----D NSGFMTEYVA TRWYRAPEIM LTFKEYTK-- ---------- ----------
 644393  ---------T LRALKAMHSA NVLHRDL--- ------KP-S NLLLNANCDL K--------- ---------- ---VCDFGLA R-------SA A-SQE----D NSGFMTEYVA TRWYRAPEIM LTFKEYTK-- ---------- ----------
 784446  ---------T LRALKAMHSA NVLHRDL--- ------KP-S NLLLNANCDL K--------- ---------- ---VCDFGLA R-------SA A-SQE----D NSGFMTEYVA TRWYRAPEIM LTFKEYTK-- ---------- ----------
 567834  ---------T LRALKAMHSA NVLHRDL--- ------KP-S NLLLNANCDL K--------- ---------- ---VCDFGLA R-------SA A-SQE----D NSGFMTEYVA TRWYRAPEIM LTFKEYTK-- ---------- ----------
 389770  ---------T LRALKAMHSA NVLHRDL--- ------KP-S NLLLNANCDL K--------- ---------- ---VCDFGLA R-------SA A-SQE----D NSGFMTEYVA TRWYRAPEIM LTFKEYTK-- ---------- ----------
  63860  ---------T LRALKAMHSA NVLHRDL--- ------KP-S NLLLNANCDL K--------- ---------- ---VCDFGLA R-------SA A-SQE----D NSGFMTEYVA TRWYRAPEIM LTFKEYTK-- ---------- ----------
 131603  ---------T LRALKAMHSA NVLHRDL--- ------KP-S NLLLNANCDL K--------- ---------- ---VCDFGLA R-------SA A-SQE----D NSGFMTEYVA TRWYRAPEIM LTFKEYTK-- ---------- ----------
 495859  ---------T LRALKAMHSA NVLHRDL--- ------KP-S NLLLNANCDL K--------- ---------- ---VCDFGLA R-------SA A-SSD----D NSGFMTEYVA TRWYRAPEIM LTFKEYTT-- ---------- ----------
 434551  ---------T LRALKAMHSA NVLHRDL--- ------KP-S NLLLNANCDL K--------- ---------- ---VCDFGLA R-------SA A-SSD----D NSGFMTEYVA TRWYRAPEIM LTFKEYTT-- ---------- ----------
 140828  ---------T LRALKAMHSA NVLHRDL--- ------KP-S NLLLNANCDL K--------- ---------- ---VCDFGLA R-------SA A-SQE----D NSGFMTEYVA TRWYRAPEIM LTFKEYTK-- ---------- ----------
  66435  ---------T LRALKAMHSA NVLHRDL--- ------KP-S NLLLNANCDL K--------- ---------- ---VCDFGLA R-------SA A-STD----D NSGFMTEYVA TRWYRAPEIM LTFKEYTK-- ---------- ----------
 383913  ---------T LRALKAMHSA NVLHRDL--- ------KP-S NLLLNANCDL K--------- ---------- ---VCDFGLA R-------SA A-STD----D NSGFMTEYVA TRWYRAPEIM LTFKEYTK-- ---------- ----------
 323815  ---------T LRALKAMHSA NVLHRDL--- ------KP-S NLLLNANCDL K--------- ---------- ---VCDFGLA R-------SA A-STD----D NSGFMTEYVA TRWYRAPEIM LTFKEYTK-- ---------- ----------
 396599  ---------T LRALKAMHSA NVLHRDL--- ------KP-S NLLLNANCDL K--------- ---------- ---VCDFGLA R-------SA A-STD----D NSGFMTEYVA TRWYRAPEIM LTFKEYTK-- ---------- ----------
  74447  ---------T LRALKAMHSA NVLHRDL--- ------KP-S NLLLNANCDL K--------- ---------- ---VCDFGLA R-------SA A-STD----D NSGFMTEYVA TRWYRAPEIM LTFKEYTK-- ---------- ----------
  83363  ---------T LRALKAMHSA NVLHRDL--- ------KP-S NLLLNANCDL K--------- ---------- ---VCDFGLA R-------SA A-STD----D NSGFMTEYVA TRWYRAPEIM LTFKEYTK-- ---------- ----------
  50905  ---------T LRALKAMHSA NVLHRDL--- ------KP-S NLLLNANCDL K--------- ---------- ---VCDFGLA R-------SA A-STD----D NSGFMTEYVA TRWYRAPEIM LTFKEYTK-- ---------- ----------
 650405  ---------T LRALKAMHSA NVLHRDL--- ------KP-S NLLLNANCDL K--------- ---------- ---VCDFGLA R-------SA A-STE----D NSGFMTEYVA TRWYRAPEIM LTFKEYTK-- ---------- ----------
 493847  ---------T LRALKAMHSA NVLHRDL--- ------KP-S NLLLNANCDL K--------- ---------- ---VCDFGLA R-------SA A-STE----D NSGFMTEYVA TRWYRAPEIM LTFKEYTK-- ---------- ----------
  95409  ---------T LRGLKALHSA NVLHRDL--- ------KP-S NLLLNANCDL K--------- ---------- ---ICDFGLA R-------SA A-STE----D NQGFMTEYVA TRWYRAPEIM LTFKEYTT-- ---------- ----------
 835420  ---------T LRALKCMHSA NVLHRDL--- ------KP-S NLLLNANCDL K--------- ---------- ---ICDFGLA R-------SA N-SND----E HSGFMTEYVA TRWYRAPEIM LTFKEYTK-- ---------- ----------
 343609  ---------T LRALKAMHSA NVLHRDL--- ------KP-S NLLLNANCDL K--------- ---------- ---VCDFGLA R-------SA N-SSD----E HSGFMTEYVA TRWYRAPEIM LTFKEYTK-- ---------- ----------
  67554  ---------T LRALKAMHSA NVLHRDL--- ------KP-S NLLLNANCDL K--------- ---------- ---VCDFGLA R-------SA A-STE----D NSGFMTEYVA TRWYRAPEIM LTFKEYTK-- ---------- ----------
 216015  ---------T LRALKALHSA NVLHRDL--- ------KP-S NLLLNANCDL K--------- ---------- ---ICDFGLA R-------SV A-SSE----D NFGFMTEYVA TRWYRAPEIM LTFQEYTT-- ---------- ----------
  52193  ---------T LRALKAMHLA NVLHRDL--- ------KP-S NLLLNSNCDL K--------- ---------- ---VCDFGLA R-------SI A-SSE----D NFGYMTEYVA TRWYRAPEIM LTFQEYTT-- ---------- ----------
  53916  ---------T LRALKAMHSA NVLHRDL--- ------KP-S NLLLNSNCDL K--------- ---------- ---VCDFGLA R-------SI A-SSE----D NFGFMTEYVA TRWYRAPEIM LTFQEYTT-- ---------- ----------
  83931  ---------T LRALKALHSA NVLHRDL--- ------KP-S NLLLNANCDL K--------- ---------- ---ICDFGLA R-------SV A-STE----D NFGFMTEYVS TRWYRAPEIM LTFQEYTT-- ---------- ----------
  51986  ---------T LRALKALHSA NILHRDL--- ------KP-S NLLLNANCDL K--------- ---------- ---VCDFGLA R-------SA A-GND----D NFGFMTEYVA TRWYRAPEIM LTFKEYTK-- ---------- ----------
  49589  ---------T LRALKALHSA NVLHRDL--- ------KP-S NLLLNANCDL K--------- ---------- ---VCDFGLS R-------ST A-STQ----D NFGFMTEYVA TRWYRAPEIM LTFKEYTK-- ---------- ----------
 862988  ---------T LRALKALHSA DVIHRDL--- ------KP-S NLLLNANCDL K--------- ---------- ---VCDFGLA R-------SV K-TAEP-SGT ETGFMTEYVA TRWYRAPEIM LTFKQYTK-- ---------- ----------
 833500  ---------T LRALKALHSA DVIHRDL--- ------KP-S NLLLNANCDL K--------- ---------- ---VCDFGLA R-------SV K-TAEP-SGT ETGFMTEYVA TRWYRAPEIM LTFKQYTK-- ---------- ----------
 810703  ---------T LRALKALHSA DVIHRDL--- ------KP-S NLLLNANCDL K--------- ---------- ---VCDFGLA R-------SV K-TAEP-SGT ETGFMTEYVA TRWYRAPEIM LTFKQYTK-- ---------- ----------
 457677  ---------T LRALKALHSA DVIHRDL--- ------KP-S NLLLNANCDL K--------- ---------- ---VCDFGLA R-------SV K-TAEP-SGT ETGFMTEYVA TRWYRAPEIM LTFKQYTK-- ---------- ----------
 161161  ---------T LRALKALHSA DVIHRDL--- ------KP-S NLLLNANCDL K--------- ---------- ---VCDFGLA R-------SV R-TAEP-SGT ETGFMTEYVA TRWYRAPEIM LTFKQYTK-- ---------- ----------
1588194  ---------T LRALKALHSA DVIHRDL--- ------KP-S NLLLNANCDL K--------- ---------- ---VCDFGLA R-------SV R-TAEP-SGT ETGFMTEYVA TRWYRAPEIM LTFKQYTK-- ---------- ----------
  98485  ---------T LRALKALHSA DVIHRDL--- ------KP-S NLLLNANCDL K--------- ---------- ---VCDFGLA R-------SV K-TAEP-SGT ETGFMTEYVA TRWYRAPEIM LTFKQYTK-- ---------- ----------
1443964  ---------T LRALKALHSA DVIHRDL--- ------KP-S NLLLNANCDL K--------- ---------- ---VCDFGLA R-------SV K-TAEP-SGT ETGFMTEYVA TRWYRAPEIM LTFKQYTK-- ---------- ----------
 864221  ---------T LRALKALHSA DVIHRDL--- ------KP-S NLLLNANCDL K--------- ---------- ---VCDFGLA R-------SV K-TAEP-SGT ETGFMTEYVA TRWYRAPEIM LTFKQYTK-- ---------- ----------
 903905  ---------T LRALKALHSA DVIHRDL--- ------KP-S NLLLNANCDL K--------- ---------- ---VADFGLA R-------SV K-TAEP-SGT ETGFMTEYVA TRWYRAPEIM LTFKQYTK-- ---------- ----------
1147795  ---------T LRALKALHSA DVIHRDL--- ------KP-S NLLLNANCDL K--------- ---------- ---VCDFGLA R-------SV K-TAEP-SGT ETGFMTEYVA TRWYRAPEIM LTFKQYTK-- ---------- ----------
 622735  ---------T LRALKALHSA DVIHRDL--- ------KP-S NLLLNANCDL K--------- ---------- ---VCDFGLA R-------SV K-TAEP-SGT ETGFMTEYVA TRWYRAPEIM LTFKQYTK-- ---------- ----------
 166786  ---------T LRALKALHSA DVIHRDL--- ------KP-S NLLLNANCDL K--------- ---------- ---VCDFGLA R-------SV K-TAEP-SGT ETGFMTEYVA TRWYRAPEIM LTFKQYTK-- ---------- ----------
2641010  ---------T LRALKALHSA DVIHRDL--- ------KP-S NLLLNANCDL K--------- ---------- ---VCDFGLA R-------SV K-TAEP-SGT ETGFMTEYVA TRWYRAPEIM LTFKQYTK-- ---------- ----------
  28679  ---------T LRALKALHSA DVIHRDL--- ------KP-S NLLLNANCDL K--------- ---------- ---VCDFGLA R-------SV R-TAEP-SGT ETGFMTEYVA TRWYRAPEIM LTFKQYTK-- ---------- ----------
 101027  ---------T LRALKALHSA DVIHRDL--- ------KP-S NLLLNANCDL K--------- ---------- ---VCDFGLA R-------SV K-TAEP-SGT ETGFMTEYVA TRWYRAPEIM LTFKQYTK-- ---------- ----------
 564549  ---------T LRALKALHSA DVIHRDL--- ------KP-S NLLLNANCDL K--------- ---------- ---VADFGLA R-------SV K-TAEP-SGT ETGFMTEYVA TRWYRAPEIM LTFKQYTK-- ---------- ----------
 227465  ---------T LRALKALHSA DVIHRDL--- ------KP-S NLLLNANCDL K--------- ---------- ---VCDFGLA R-------SV R-TAEP-SGT ETGFMTEYVA TRWYRAPEIM LTFKQYTK-- ---------- ----------
  18095  ---------T LRALKALHSA DVIHRDL--- ------KP-S NLLLNANCDL K--------- ---------- ---VCDFGLA R-------SV K-TAEP-SGT ETGFMTEYVA TRWYRAPEIM LTFKQYTK-- -VGIPHLVAA ARLKLIPGYN
 338545  ---------T LRALKALHSA DVIHRDL--- ------KP-S NLLLNANCDL K--------- ---------- ---VCDFGLA R-------SV K-TAEP-SGT ETGFMTEYVA TRWYRAPEIM LTFKQYTK-- ---------- ----------
 134728  ---------T LRALKALHSA DVIHRDL--- ------KP-S NLLLNANCDL K--------- ---------- ---VCDFGLA R-------SV K-TAEP-SGT ETGFMTEYVA TRWYRAPEIM LTFKQYTK-- ---------- ----------
 275037  ---------T LRALKALHSA DVIHRDL--- ------KP-S NLLLNANCDL K--------- ---------- ---VCDFGLA R-------SV K-TAEP-SGT ETGFMTEYVA TRWYRAPEIM LTFKQYTK-- ---------- ----------
 503339  ---------T LRALKALHSA DVIHRDL--- ------KP-S NLLLNANCDL K--------- ---------- ---VCDFGLA R-------SV K-TAEP-SGT ETGFMTEYVA TRWYRAPEIM LTFKQYTK-- ---------- ----------
  40745  ---------T LRALKALHSA DVIHRDL--- ------KP-S NLLLNANCDL K--------- ---------- ---VCDFGLA R-------SV R-TAEQ---- ETGFMTEYVA TRWYRAPEIM LTFKQYTK-- ---------- ----------
  43876  ---------T LRALKAMHSA NIIHRDL--- ------KP-S NLLLNANCDL K--------- ---------- ---VCDFGLA R-------SV L-TAEP-NGG ETGFMTEYVA TRWYRAPEIM LTFKQYTK-- ---------- ----------
  14716  ---------T LRALKAMHSA NIIHRDL--- ------KP-S NLLLNANCDL K--------- ---------- ---VCDFGLA R-------SV L-TAEP-NGG ETGFMTEYVA TRWYRAPEIM LTFKQYTK-- ---------- ----------
  15138  ---------T LRALKALHSA DVIHRDL--- ------KP-S NLLLNANCDL K--------- ---------- ---VCDFGLA R-------SV T-TAEP-NGG ETGFMTEYVA TRWYRAPEIM LTFKQYTK-- ---------- ----------
  67488  ---------T CRALKAMHSA DVIHRDL--- ------KP-S NLLLNANCDL K--------- ---------- ---VCDFGLA R-------ST Q-TAEP--GC ETGFMTEYVA TRWYRAPEIM LTFKQYTS-- ---------- ----------
  28680  ---------T LRALKALHSA DVIHRDL--- ------KP-S NLLLNANCDL K--------- ---------- ---VCDFGLA R-------SV R-TAEP-SGT ETGFMTEYVA TRWYRAPEIM LTFKQYTK-- ---------- ----------
 177514  ---------T LRALKSIHSA GIVHRDL--- ------KP-A NLLLNANCDL K--------- ---------- ---VCDFGLA R-------SV N-TSVP-PGK EVGIMTEYVA TRWYRAPEIM LSFKMYTK-- ---------- ----------
  53880  ---------T LRALKSIHSA GIVHRDL--- ------KP-A NLLLNANCDL K--------- ---------- ---VCDFGLA R-------SV K-TSVA-GGK EVGIMTEYVA TRWYRAPEIM LSFKMYTK-- ---------- ----------
 778168  ---------T LRALKSIHSA DIVHRDL--- ------KP-A NLLLNANCDL K--------- ---------- ---VCDFGLA R-------SV K-NSSA-NGK ETGLMTEYVA TRWYRAPEIM LSFKMYTK-- ---------- ----------
 388025  ---------V LRALKSIHSA DIVHRDL--- ------KP-A NLLLNANCDL K--------- ---------- ---VCDFGLA R-------SI N-STAP-GGK EMGLMTEYVA TRWYRAPEIM LSFKMYTK-- ---------- ----------
 581713  ---------T LRALKSIHSA DIVHRDL--- ------KP-A NLLLNANCDL K--------- ---------- ---VCDFGLA R-------SI K-STAP-GGK DMGLMTEYVA TRWYRAPEIM LSFKMYTK-- ---------- ----------
 229317  ---------T LRALKSIHSA DIVHRDL--- ------KP-A NLLLNANCDL K--------- ---------- ---VCDFGLA R-------SV K-STTS-GGK EVGLMTEYVA TRWYRAPEIM LSFKMYTK-- ---------- ----------
 829657  ---------I LRGLKSIHSA DIVHRDL--- ------KP-A NLLLNANCDL K--------- ---------- ---VCDFGLA R-------SV K-ATTP-GAK EAGLMTEYVA TRWYRAPEIM LSFKMYTK-- ---------- ----------
  70959  ---------T LRALKTMHSA DIVHRDL--- ------KP-A NLLLNANCDL K--------- ---------- ---VCDFGLA R-------ST R-STNP-GGK EAGLMTEYVA TRWYRAPEIM LSFKMYTK-- ---------- ----------
  32857  ---------T LRALKTMHSA DIVHRDL--- ------KP-A NLLLNANCDL K--------- ---------- ---VCDFGLA R-------ST R-STNP-GGK EAGLMTEYVA TRWYRAPEIM LSFKMYTK-- -VG---RAGY RHRRHLTNRN
 561172  ---------V LRALKTMHSA DLVHRDL--- ------KP-A NLLLNANCDL K--------- ---------- ---VCDFGLA R-------ST R-STNP-SGK EAGLMTEYVA TRWYRAPEIM LSFKMYTK-- ---------- ----------
 573944  ---------T LRALKTMHSA DIVHRDL--- ------KP-A NLLLNANCDL K--------- ---------- ---VCDFGLA R-------ST R-STNP-GGK EAGLMTEYVA TRWYRAPEIM LSFKMYTK-- ---------- ----------
 740770  ---------T LRALKTIHSA DIVHRDL--- ------KP-A NLLVNANCDL K--------- ---------- ---VCDFGLA R-------SV K-TSSAQGGK DIGMMTEYVA TRWYRAPEIM LSFKMYTK-- ---------- ----------
  16071  ---------V LRALKSIHSA DIVHRDL--- ------KP-A NLLLNSNCDL K--------- ---------- ---VCDFGLA R-------SV N-CTSP-DGK DAGLLTEYVA TRWYRAPEIM LSFKRYTK-- ---------- ----------
1126092  ---------V LRALKSIHSA DIVHRDL--- ------KP-A NLLLNSNCDL K--------- ---------- ---VCDFGLA R-------SV N-CTSP-DGK DAGLLTEYVA TRWYRAPEIM LSFKRYTK-- ---------- ----------
 478842  ---------T LRALRSVHSA DIVHRDL--- ------KP-A NLLLNANCDL K--------- ---------- ---VADFGLA R-------SV R-TGLS-NGA ESGLMTEYVA TRWYRAPEIM LSFKRYTK-- ---------- ----------
  46649  ---------T LRALLSIHTA DIVHRDL--- ------KP-S NLLVNANCDL K--------- ---------- ---VCDFGLA R-------SV K-SSSLMGGK DVGMMTEYVA TRWYRAPEIM LSFKMYTK-- ---------- ----------
 601377  ---------T LLAIKSMHDA DIVHRDL--- ------KP-A NILLNQNCDL K--------- ---------- ---VCDFGLA R-------SV R-T-----GR PAG--EEYVA TRWYRAPEIM LSFKMYTK-- ---------- ----------
 866574  ---------T LRALKALHSA DVLHRDL--- ------KP-S NLLLNANCDL K--------- ---------- ---ICDFGLA R-------SA RPPPNL-AND SNTFMTEYVA TRWYRAPEVM LTFKEYTR-- ---------- ----------
 521401  ---------T LRALKALHSA DVLHRDL--- ------KP-S NLLLNANCDL K--------- ---------- ---ICDFGLA R-------SA RPPPNL-AND SNTFMTEYVA TRWYRAPEVM LTFKEYTR-- ---------- ----------
 850251  ---------T LRALKALHSA DVLHRDL--- ------KP-S NLLLNANCDL K--------- ---------- ---ICDFGLA R-------SA RPPPNL-AND SNTFMTEYVA TRWYRAPEVM LTFKEYTR-- ---------- ----------
 832111  ---------T LRALKALHSA DVLHRDL--- ------KP-S NLLLNANCDL K--------- ---------- ---LCDFGLA R-------SA RPPPNV-AND SSTFMTEYVA TRWYRAPEVM LTFKEYTR-- ---------- ----------
 832110  ---------T LRALKALHSA DVLHRDL--- ------KP-S NLLLNANCDL K--------- ---------- ---LCDFGLA R-------SA RPPPNV-AND SSTFMTEYVA TRWYRAPEVM LTFKEYTR-- ---------- ----------
 676666  ---------T LRALKALHSA DVLHRDL--- ------KP-S NLLLNANCDL K--------- ---------- ---LCDFGLA R-------SA RPPPNV-AND SSTFMTEYVA TRWYRAPEVM LTFKEYTR-- ---------- ----------
1143376  ---------T LRALKALHSA DVLHRDL--- ------KP-S NLLLNANCDL K--------- ---------- ---VCDFGLA R-------SA RPPPNI-AND SSTFMTEYVA TRWYRAPEVM LTFKEYTR-- ---------- ----------
 457401  ---------T LRALKALHSA DVLHRDL--- ------KP-S NLLVNANCDL K--------- ---------- ---VCDFGLA R-------SA RPPPNV-AND SSTFMTEYVA TRWYRAPEVM LTFKEYTR-- ---------- ----------
 834255  ---------A LRALKALHSA DVLHRDL--- ------KP-S NLLLNANCDL K--------- ---------- ---VCDFGLA R-------SA RPPPNV-AND SSTFMTEYVA TRWYRAPEVM LTFKEYTR-- ---------- ----------
 133042  ---------T LRAIKALHSA DVLHRDL--- ------KP-S NLLLNANCDL K--------- ---------- ---LCDFGLA R-------SA RPPPNF-AND SSTFMTEYVA TRWYRAPEVM LTFKEYTR-- ---------- ----------
  30507  ---------T LRALKALHSA DVLHRDL--- ------KP-S NLLLNANCDL K--------- ---------- ---LCDFGLA R-------SA RPPPNF-AND SSTFMTEYVA TRWYRAPEVM LTFKEYTR-- ---------- ----------
 646041  ---------T LRALKALHSA DVLHRDL--- ------KP-S NLLLNANCDL K--------- ---------- ---LCDFGLA R-------SA RPPPNV-AND SSTFMTEYVA TRWYRAPEVM LTFKEYTR-- ---------- ----------
 570964  ---------T LRALKALHSA DVLHRDL--- ------KP-S NLLLNANCDL K--------- ---------- ---LCDFGLA R-------SA RPPPNV-AND SSTFMTEYVA TRWYRAPEVM LTFKEYTR-- ---------- ----------
 301734  ---------T LRALKALHSA DVLHRDL--- ------KP-S NLLLNANCDL K--------- ---------- ---LCDFGLA R-------SA RPPPN--LDD TSNFLTEYVA TRWYRAPEVM LTFKEYTR-- ---------- ----------
 154378  ---------T LRALKALHSA DVLHRDL--- ------KP-S NLLLNANCDL K--------- ---------- ---LCDFGLA R-------SA RPPPD--ADD SSNFLTEYVA TRWYRAPEVM LTFKEYTR-- ---------- ----------
  90189  ---------T LRALKALHSA DVLHRDL--- ------KP-S NLLLNANCDL K--------- ---------- ---LCDFGLA R-------SA RPPPNV-AND SSTFMTEYVA TRWYRAPEVM LTFKEYTR-- ---------- ----------
 448371  ---------T LRALKALHSA NVLHRDL--- ------KP-S NLLLNANCDL K--------- ---------- ---LCDFGLA R-------SS LPAPDANGNE ASTFMTEYVA TRWYRAPEVM LTFKEYTR-- ---------- ----------
 476779  ---------T LRALKALHSA NVLHRDL--- ------KP-S NLLLNANCDL KVSVLE---- ---------- ---LSHPTLS R-------SS LPAPDANGNE ASTFMTEYVA TRWYRAPEVM LTFKEYTR-- ---------- ----------
  68819  ---------T LRALKALHSA DVLHRDL--- ------KP-S NLLLNANCDL K--------- ---------- ---LCDFGLA R-------SA KPPPNV-AND SSTFLTEYVA TRWYRAPEVM LTFKEYTR-- ---------- ----------
2671102  ---------T LRALKALHSA DVLHRDL--- ------KP-S NLLLNANCDL K--------- ---------- ---LCDFGLA R-------SA RPPPNV--ED SSTFMTEYVA TRWYRAPEVM LTFKEYTR-- ---------- ----------
 940143  ---------T LRALKALHSA DVLHRDL--- ------KP-S NLLLNANCDL K--------- ---------- ---LCDFGLA R-------SA RPPPNV--DD TSTFMTEYVA TRWYRAPEVM LTFKEYTR-- ---------- ----------
 585786  ---------T LRALKALHSA DVLHRDL--- ------KP-S NLLLNANCDL K--------- ---------- ---LCDFGLA R-------SA RPPPNV--DP TITFMTEYVA TRWYRAPEVM LTFKEYTR-- ---------- ----------
 349085  RFSTLHPPQT LRALKALHSA DVLHRDL--- ------KP-S NLLLNANCDL K--------- ---------- ---LCDFGLA R-------SA RPPPNV--DD TSTFMTEYVA TRWYRAPEVM LTFKEYTR-- ---------- ----------
1539717  ---------T LRALKALHSA NVLHRDL--- ------KP-S NLLLNANCDL K--------- ---------- ---LCDFGLA R-------SS LPAANG-DDG SSTFMTEYVA TRWYRAPEVM LTFKEYTR-- ---------- ----------
  24638  ---------A LRGLKALHSA NVLHRDL--- ------KP-S NLLLNSNCDL K--------- ---------- ---VCDFGLA R-------SS QPPPDA-AND VSTFMTEYVA TRWYRAPEVM LTFKEYTR-- ---------- ----------
  75000  ---------A LRGLKALHSA NVLHRDL--- ------KP-S NLLLNSNCDL K--------- ---------- ---VCDFGLA R-------SS QPPPDA-AND VSTFMTEYVA TRWYRAPEVM LTFKEYTR-- ---------- ----------
1245527  ---------T LRALKALHSA DVLHRDL--- ------KP-S NLLLNANCDL K--------- ---------- ---LCDFGLA R-------SA RPPPNV--DD TSTFMTEYVA TRWYRAPEVM LTFKEYTR-- ---------- ----------
 103018  ---------T LRGLKALHSA AVLHRDL--- ------KP-S NLLLNANCDL K--------- ---------- ---ICDFGLA R-------SS IPSQN---QE GQGFLTEYVA TRWYRAPEIM LTFKEYTK-- ---------- ----------
  43883  ---------T LRGLKALHSA AVLHRDL--- ------KP-S NLLLNANCDL K--------- ---------- ---ICDFGLA R-------SA MSSET----E AVGFMTEYVA TRWYRAPEIM LTFKEYTF-- ---------- ----------
  58913  ---------T LRGLKALHSA AVLHRDL--- ------KP-S NLLLNSNCDL KVSMSEDREN TESDLIWFVL IHQICDFGLA R-------SA FMGEQ----E ATGFMTEYVA TRWYRAPEIM LTFKEYTK-- ---------- ----------
  18326  ---------T LRALKALHSA NILHRDL--- ------KP-S NLLLNANCDL K--------- ---------- ---LCDFGLA R-------SA HPPPGY-QDS AANFMTEYVA TRWYRAPEIF F-LESYSK-- ---------- ----------
 301059  ---------T LRALKALHSA NILHRDL--- ------KP-S NLLLNANCDL K--------- ---------- ---LCDFGLA R-------SA HPPPGY-QDS AANFMTEYVA TRWYRAPEIF F-LESYSK-- ---------- ----------
  12410  ---------T LRALKALHSA NILHRDL--- ------KP-S NLLLNANCDL K--------- ---------- -NQLCDFGLA R-------SA HPPPGY-QDS AANFMTEYVA TRWYRAPEIF F-LESYSK-- ---------- ----------
  18327  ---------T LRALKALHSA NILHRDL--- ------KP-S NLLLNANCDL K--------- ---------- ---LCDFGLA R-------SA HPPPGY-QDS AANFMTEYVA TRWYRAPEIF F-LESYSK-- ---------- ----------
 184754  ---------I LRALKMLHSA NVIHRDL--- ------KP-S NLLLNANCDL K--------- ---------- ---ICDFGLA R---IDAAKE GNGTEK---- -INLLTEYVA TRWYRAPEIM LTSSQYSK-- ---------- ----------
  99455  ---------I LRGLKALHSA NVIHRDL--- ------KP-S NLLLNANCDL K--------- ---------- ---ICDFGLA R---IDEQKQ GNYANSPENK GISMMTEYVA TRWYRAPEIM LTASKYTT-- ---------- ----------
 706256  ---------T LRAVKVLHGS NVIHRDL--- ------KP-S NLLINSNCDL K--------- ---------- ---VCDFGLA R---IIDESA ADNSEPTGQQ --SGMTEYVA TRWYRAPEVM LTSAKYSR-- ---------- ----------
 653342  ---------T LRAVKVLHGS NVIHRDL--- ------KP-S NLLINSNCDL K--------- ---------- ---VCDFGLA R---IIDESA ADNSEPTGQQ --SGMTEYVA TRWYRAPEVM LTSAKYSR-- ---------- ----------
 462249  ---------T LRALKALHSA DVIHRDL--- ------KP-S NLLLNANCDL K--------- ---------- ---VCDFGLA R-------SV KTAEP--SGT ETGFMTEYVA TRWYR----- ---------- ---------- ----------
```

```
     326973  ---------T LRALKAMHSA NVLHRDL--- ------KP-S NLLLNANCDL K--------- ---------- ---VCDFGLA R-------SA ASME-----D NSGFMTEYVA TRWYRAPEIM LTFKEYTK-- ---------- ----------
      77855  ---------T LRALKAMHSA NVLHRDL--- ------KP-S NLLLNANCDL K--------- ---------- ---VCDFGLA R-------SA ASTD-----D NSGFMTEYVA TRWYRAPEIM LTFKEYTK-- ---------- ----------
     338563  ---------T LRALKALHSA DVIHRDL--- ------KP-S NLLLNANCDL K--------- ---------- ---VCDFGLA R-------SV KTAEP--SGT ETGFMTEYVA TRWYRAPEIM LTFKQYTK-- ---------- ----------
     233520  ---------T LRALKALHSA DVIHRDL--- ------KP-S NLLLNANCDL K--------- ---------- ---VCDFGLA R-------SV KTAEP--SGT ETGFMTEYVA TRWYRAPEIM LTFKQYTK-- ---------- ----------
      73610  ---------T LRALKALHSA DVIHRDL--- ------KP-S NLLLNANCDL K--------- ---------- ---VCDFGLA R-------SV KTAEP--SGT ETGFMTEYVA TRWYRAPEIM LTFKQYTK-- ---------- ----------
     150924  ---------T LRALKALHSA DVIHRDL--- ------KP-S NLLLNANCDL K--------- ---------- ---VCDFGLA R-------SV KTAEP--SGT ETGFMTEYVA TRWYRAPEIM LTFKQYTK-- ---------- ----------
     348809  ---------T LRALKALHSA DVIHRDL--- ------KP-S NLLLNANCDL K--------- ---------- ---VADFGLA R-------SV KTAEP--SGT ETGFMTEYVA TRWYRAPEIM LTFKQYTK-- ---------- ----------
     913771  ---------T LRALKALHSA DVIHRDL--- ------KP-S NLLLNANCDL K--------- ---------- ---VCDFGLA R-------SV KTAEP--SGT ETGFMTEYVA TRWYRAPEIM LTFKQYTK-- ---------- ----------
     913918  ---------T LRALKALHSA DVIHRDL--- ------KP-S NLLLNANCDL K--------- ---------- ---VCDFGLA R-------SV KTAEP--SGT ETGFMTEYVA TRWYRAPEIM LTFKQYTK-- ---------- ----------
     747578  ---------I LRALKSIHSA QVIHRDI--- ------KP-S NLLLNSNCDL K--------- ---------- ---VCDFGLA R--------C LASSSDSRET LVGFMTEYVA TRWYRAPEIM LTFQEYTT-- ---------- ----------
     458977  ---------I LRALKSIHSA QVIHRDI--- ------KP-S NLLLNSNCDL K--------- ---------- ---VCDFGLA R--------C LASSSDSRET LVGFMTEYVA TRWYRAPEIM LTFQEYTT-- ---------- ----------
      41459  ---------I LRGLKTVHSA DVVHRDL--- ------KP-A NLLINANCDL K--------- ---------- ---ICDFGLA R-------SV KTSSVE-GPK DAGLMTEYVA TRWYRAPEIM LSFKMYTK-- ---------- ----------
    1397463  ---------T LRALKTMHSA DIVHRDL--- ------KP-A NLLLNANCDL K--------- ---------- ---VCDFGLA R-------ST R-STTP-GGK EAGLMTEYVA TRWYRAPEIM LSFKMYTK-- ---------- ----------
     149203  ---------T LRALKTMHSA DIVHRDL--- ------KP-A NLLLNANCDL K--------- ---------- ---VCDFGLA R-------ST R-STNP-GGK EAGLMTEYVA TRWYRAPEIM LSFKMYTK-- ---------- ----------
     102078  ---------I LRGLKTIHSA NVIHRDL--- ------KP-S NVLVNYNCDV K--------- ---------- ---ICDFGLA R---I-MGST MSPLE----- --SKLTEYVA TRWYRAPEIM LSQSMYTT-- ---------- ----------
 36766:44-3  ---------I LRGLKLIHSA GVIHRDL--- ------KP-S NILINENCDL K--------- ---------- ---ICDFGLA R---L-DVLH PNSSKIPKKG GISALTEYVA TRWYRAPEIM LNSAQYSS-- ---------- ----------
      54278  ---------I LKGLKLIHSA KVIHRDL--- ------KP-S NLLVNEECDL K--------- ---------- ---ICDFGLA R---L-DNQL YPNQELPNN- -ISSLTEYVA TRWYRAPEIM LNAANYSR-- ---------- ----------
      29185  ---------T LRALKALHSA DVIHRDL--- ------KP-S NLLLNANCDL K--------- ---------- ---VCDFGLA R-------SV TTAEP--NGG ETGFMTEYVA TRWYRAPEIM LT-------- ---------- ----------
     790328  ---------T LRALKALHSA DVLHRDL--- ------KP-S NLLLNANCDL K--------- ---------- ---ICDFGLA R-----SARP P---PNLAND SNTFMTEYVA TRWYRAPEVM LTFKEYTR-- ---------- ----------
     765763  ---------T LRALKALHSA DVLHRDL--- ------KP-S NLLLNANCDL K--------- ---------- ---ICDFGLA R-----SARP P---PNLAND SNTFMTEYVA TRWYRAPEVM LTFKEYTR-- ---------- ----------
    1491990  ---------T LRALKALHSA NVLHRDL--- ------KP-S NLLLNANCDL K--------- ---------- ---LCDFGLA R-----SSLP A---ANGDDG SSTFMTEYVA TRWYRAPEVM LTFKEYTR-- ---------- ----------
      32998  ---------T LRALKALHSA DVIHRDL--- ------KP-S NLLLNANCDL K--------- ---------- ---VCDFGLA R-----SVKT A---EP-SGT ETGFMTEYVA TRWYRAPEIM LTFKQYTK-- ---------- ----------
    2601990  ---------T LRALKALHSA DVLHRDL--- ------KP-S NLLLNANCDL K--------- ---------- ---LCDFGLA R-----SARP P---PN-VED SSTFMTEYVA TRWYRAPEVM LTFKEYTR-- ---------- ----------
     262460  ---------T LRALKAMHTA NVLHRDL--- ------KP-S NLLLNANCDL K--------- ---------- ---VCDFGLA R-----SAAS -------TED NQGFMTEYVA TRWYRAPEIM LTFKEYTK-- ---------- ----------
      11717  ---------M LCALHHIHSA GVLHRDM--- ------KP-S NILLNANCDL K--------- ---------- ---VCDFGLA R-----GGVG SSTWPQPVLS HPGELTEYVV TRWYRAPEIM LNCLQYST-- ---------- ----------
     532299  ---------M LCALHHIHSA GVLHRDM--- ------KP-S NILLNANCDL K--------- ---------- ---VCDFGLA R-----GGVG G----QEELL QPGELTEYVV TRWYRAPEIM LNCLHYTT-- ---------- ----------
     551831  ---------M LCALHHIHSA GVLHRDM--- ------KP-S NILLNANCDL K--------- ---------- ---VCDFGLA R-----GGVG G----QEELL QPGELTEYVV TRWYRAPEIM LNCLHYTT-- ---------- ----------
     301484  ---------M LCALHHIHSA GVLHRDM--- ------KP-S NILLNANCDL K--------- ---------- ---VCDFGLA R-----GGVG G----QEELL QPGELTEYVV TRWYRAPEIM LNCLHYTT-- ---------- ----------
      42366  ---------T LCGLKYIHSA NVLHRDL--- ------KP-G NLLVNADCEL K--------- ---------- ---ICDFGLA R-----GYTP GGG-ASRAAG NQGFMTEYVA TRWYRAPEIM LSFANYTT-- ---------- ----------
     476004  ---------M LCAMKYVHSA NVIHRDL--- ------KP-S NVLVNANCEL K--------- ---------- ---ICDFGLA R-----GVFP ---------E EELELTEYVV TRWYRAPEIM LGCMKYTR-- ---------- ----------
     558476  ---------M LCAMKYVHSA NVIHRDL--- ------KP-S NVLVNANCEL K--------- ---------- ---ICDFGLA R-----GVFP ---------E EELELTEYVV TRWYRAPEIM LGCMKYTR-- ---------- ----------
       9954  ---------I LRALKYIHSA NVLHRDL--- ------KP-S NLLLNSNC-- ---------- ---------- ---------- -------VTP --------EE DNMELTEYVV TRWYRAPEIM LSSREYTK-- ---------- ----------
      82136  ---------I LRALKYIHSA NVLHRDL--- ------KP-S NLLLNSNCDL K--------- ---------- ---VCDFGLS R-----GVAP --------EE DNMELTEYVV TRWYRAPEIM LSSREYTK-- ---------- ----------
     312415  ---------I LRALKYIHSA NVLHRDL--- ------KP-S NLLLNSNCDL K--------- ---------- ---VCDFGLS R-----GVAP --------EE DNMELTEYVV TRWYRAPEIM LSSREYTK-- ---------- ----------
     354114  ---------M LSGLHHMHQA GVLHRDL--- ------KP-S NLLINSDCQL K--------- ---------- ---ICDLGLA R-------SK --------EA EDVGMTEYVV TRWYRAPELL LGSA-YGE-- ---------- ----------
      34134  ---------I LRGLKYIHSA SVVHRDL--- ------KP-G NLLVNADCEL K--------- ---------- ---ICDFGLS R-----GFNA A------PDE NATMMTEYVA TRWYRAPEIM LAYRAYGT-- ---------- ----------
      20005  ---------I LRGMKFIHSG NVLHRDL--- ------KP-S NLLVNSNCDL S--------- ---------- ---ICDFGLA R---G----- ------VETA HNEDLTEYVV TRWYRAPELL TDCQNYND-- ---------- ----------
     518921  ---------I LRGMKFVHSG NVLHRDL--- ------KP-S NLLVNSNCDL S--------- ---------- ---ICDFGLA R---G----- ------VETA HNEDLTEYVV TRWYRAPELL TDCQNYND-- ---------- ----------
     503206  ---------I LRGMKFVHSG NVLHRDL--- ------KP-S NLLVNSNCDL S--------- ---------- ---ICDFGLA R---G----- ------VETA HNEDLTEYVV TRWYRAPELL TDCQNYND-- ---------- ----------
      10810  ---------I LRGMKFIHSG NVLHRDL--- ------KP-S NLLVNSNCDL S--------- ---------- ---ICDFGLA R---G----- ------VETA HNEDLTEYVV TRWYRAPELL TDCQNYND-- ---------- ----------
     AtMPK1  ---------L LRGLKYIHSA NILHRDL--- ------KP-G NLLVNANCDL K--------- ---------- ---ICDFGLA R--------- ------ASNT KGQFMTEYVV TRWYRAPELL LCCDNYGT-- ---------- ----------
     AtMPK2  ---------L LRGLKYIHSA NILHRDL--- ------KP-G NLLVNANCDL K--------- ---------- ---ICDFGLA R--------- ------TSNT KGQFMTEYVV TRWYRAPELL LCCDNYGT-- ---------- ----------
     OsMPK7  ---------L LRGLKYLHSA GILHRDL--- ------KP-G NLLVNANCDL K--------- ---------- ---ICDFGLA R--------- ------TNNT KGQFMTEYVV TRWYRAPELL LCCDNYGT-- ---------- ----------
    OsMPK14  ---------L LRGLKYLHSA EILHRDL--- ------KP-G NLLVNANCDL K--------- ---------- ---ICDFGLA R--------- ------TNSS KGQFMTEYVV TRWYRAPELL LCCDNYGT-- ---------- ----------
    AtMPK14  ---------L LRGLKYLHSA NILHRDL--- ------KP-G NLLVNANCDL K--------- ---------- ---ICDFGLA R--------- ------TY-- -EQFMTEYVV TRWYRAPELL LCCDNYGT-- ---------- ----------
     AtMPK7  ---------L LRGLKYLHSA NILHRDL--- ------KP-G NLLVNANCDL K--------- ---------- ---ICDFGLA R--------- ------TSQG NEQFMTEYVV TRWYRAPELL LCCDNYGT-- ---------- ----------
    AtMPK12  ---------L LRGLKYVHSA NILHRDL--- ------RP-S NVLLNSKNEL K--------- ---------- ---IGDFGLA R--------- ------TTS- DTDFMTEYVV TRWYRAPELL LNCSEYTA-- ---------- ----------
     AtMPK4  ---------L LRGLKYVHSA NVLHRDL--- ------KP-S NLLLNANCDL K--------- ---------- ---LGDFGLA R--------- ------TKS- ETDFMTEYVV TRWYRAPELL LNCSEYTA-- ---------- ----------
   OsMPK4-1  ---------L LRGLKYVHSA NVLHRDL--- ------KP-S NLFLNANCDL K--------- ---------- ---IADFGLA R--------- ------TTT- ETDLMTEYVV TRWYRAPELL LNCSQYTA-- ---------- ----------
  OsMPK16-2  ---------V LRGLKYVHSA NVLHRDL--- ------RP-S NLLLNAKCDL K--------- ---------- ---IGDFGLA R--------- ------TTN- ETDFMMEYVV TRWYRAPELL LNCSEYTA-- ---------- ----------
     AtMPK5  ---------I LRGLKYIHSA NVLHRDL--- ------KP-S NLLLNSNCDL K--------- ---------- ---ITDFGLA R--------- ------TTS- ETEYMTEYVV TRWYRAPELL LNSSEYTS-- ---------- ----------
    AtMPK10  ---------I LRGLKYIHSA NVLHRDL--- ------KP-S NLLLSTQCDL K--------- ---------- ---ICDFGLA R--------- ------ATP- ESNLMTEYVV TRWYRAPELL LGSSDYTA-- ---------- ----------
     AtMPK6  ---------I LRGLKYIHSA NVLHRDL--- ------KP-S NLLLNANCDL K--------- ---------- ---ICDFGLA R--------- ------VTS- ESDFMTEYVV TRWYRAPELL LNSSDYTA-- ---------- ----------
     OsMPK6  ---------I LRGLKYIHSA NVLHRDL--- ------KP-S NLLLNANCDL K--------- ---------- ---ICDFGLA R--------- ------TTS- ETDFMTEYVV TRWYRAPELL LNSSEYTA-- ---------- ----------
     AtMPK3  ---------L LRGLKYIHSA NIIHRDL--- ------KP-S NLLLNANCDL K--------- ---------- ---ICDFGLA R--------- ------PTS- ENDFMTEYVV TRWYRAPELL LNSSDYTA-- ---------- ----------
     OsMPK3  ---------I LRGLKYIHSA NVIHRDL--- ------KP-S NLLLNANCDL K--------- ---------- ---ICDFGLA R--------- ------PSS- ESDMMTEYVV TRWYRAPELL LNSTDYSA-- ---------- ----------
     206269  ---------- -RGLKYVHSA GVIHRDL--- ------KP-S NILINENCDL K--------- ---------- ---ICDFGLA R--------- ---------I QDPQMTGYVS TRYYRAPEIM LTWQKYDV-- ---------- ----------
     154265  ---------- -RGLKYVHSA GVIHRDL--- ------KP-S NILINENCDL K--------- ---------- ---ICDFGLA R--------- ---------I QDPQMTGYVS TRYYRAPEIM LTWQKYDV-- ---------- ----------
     168032  ---------- -RGLKYVHSA GVIHRDL--- ------KP-S NILINENCDL K--------- ---------- ---ICDFGLA R--------- ---------I QDPQMTGYVS TRYYRAPEIM LTWQKYDV-- ---------- ----------
     307550  ---------- -RGLKYVHSA GVIHRDL--- ------KP-S NILINENCDL K--------- ---------- ---ICDFGLA R--------- ---------I QDPQMTGYVS TRYYRAPEIM LTWQKYDV-- ---------- ----------
     320251  ---------- -RGLKYVHSA GVVHRDL--- ------KP-S NILVNENCDL K--------- ---------- ---ICDFGLA R--------- ---------I QDPQMTGYVS TRYYRAPEIM LTWQKYDV-- ---------- ----------
     356517  ---------- -RGLKYVHSA GVVHRDL--- ------KP-S NILVNENCDL K--------- ---------- ---ICDFGLA R--------- ---------I QDPQMTGYVS TRYYRAPEIM LTWQKYDV-- ---------- ----------
     113434  ---------- -RGLKYVHSA GVVHRDL--- ------KP-S NILVNENCDL K--------- ---------- ---ICDFGLA R--------- ---------I QDPQMTGYVS TRYYRAPEIM LTWQKYDV-- ---------- ----------
      28887  ---------- -RGLKYVHSA GVVHRDL--- ------KP-S NILVNENCDL K--------- ---------- ---ICDFGLA R--------- ---------L ADPQMTGYVS TRYYRAPEIM LTWQKYDV-- ---------- ----------
      16635  ---------- -RGLKYVHSA GVVHRDL--- ------KP-S NILVNENCDL K--------- ---------- ---ICDFGLA R--------- ---------I QDPQMTGYVS TRYYRAPEIM LTWQKYDV-- ---------- ----------
       6923  ---------- -RGLKYVHSA GVVHRDL--- ------KP-S NILVNENCDL K--------- ---------- ---ICDFGLA R--------- ---------I QDPQMTGYVS TRYYRAPEIM LTWQKYDV-- ---------- ----------
     807502  ---------- -RGLKYVHSA GVVHRDL--- ------KP-S NILVNENCDL K--------- ---------- ---ICDFGLA R--------- ---------I QDPQMTGYVS TRYYRAPEIM LTWQKYDV-- ---------- ----------
     129058  ---------- -RGLKYVHSA GVVHRDL--- ------KP-S NILVNENCDL K--------- ---------- ---ICDFGLA R--------- ---------I QDPQMTGYVS TRYYRAPEIM LTWQKYDV-- ---------- ----------
     359241  ---------- -RGLKYVHSA GVVHRDL--- ------KP-S NILVNENCDL K--------- ---------- ---ICDFGLA R--------- ---------I QDPQMTGYVS TRYYRAPEIM LTWQKYDV-- ---------- ----------
      53282  ---------- -RGLKYVHSA GVVHRDL--- ------KP-S NILVNENCDL R--------- ---------- ---ICDFGLA R--------- ---------I QDPQMTGYVS TRYYRAPEIM LTWQKYDV-- ---------- ----------
     117789  ---------- -RGLKYVHSA GVVHRDL--- ------KP-S NILVNENCDL K--------- ---------- ---ICDFGLA R--------- ---------I QDPQMTGYVS TRYYRAPEIM LTWQKYDV-- ---------- ----------
     170586  ---------- -RGLKYVHSA GVVHRDL--- ------KP-S NILVNENCDL K--------- ---------- ---ICDFGLA R--------- ---------I QDPQMTGYVS TRYYRAPEIM LTWQKYDV-- ---------- ----------
     162119  ---------- -RGLKYVHSA GVVHRDL--- ------KP-S NILINENCDL K--------- ---------- ---ICDFGLA R--------- ---------I QDPQMTGYVS TRYYRAPEIM LTWQKYDV-- ---------- ----------
     560244  ---------- -RGLKYVHSA GVVHRDL--- ------KP-S NILINENCDL K--------- ---------- ---ICDFGLA R--------- ---------L QDPQMTGYVS TRYYRAPEIM LTWQKYDV-- ---------- ----------
     499279  ---------- -RGLKYVHSA GVVHRDL--- ------KP-S NILINENCDL K--------- ---------- ---ICDFGLA R--------- ---------I QDPQMTGYVS TRYYRAPEIM LTWQKYDV-- ---------- ----------
     898207  ---------- -RGLKYVHSA GVVHRDL--- ------KP-S NILVNENCDL K--------- ---------- ---ICDFGLA R--------- ---------I QDPQMTGYVS TRYYRAPEIM LTWQKYDV-- ---------- ----------
     500359  ---------- -RGLKYVHSA GVVHRDL--- ------KP-S NILVNENCDL K--------- ---------- ---ICDFGLA R--------- ---------I QDPQMTGYVS TRYYRAPEIM LTWQKYDV-- ---------- ----------
     390416  ---------- -RGLKYVHSA GVVHRDL--- ------KP-S NILVNENCDL K--------- ---------- ---ICDFGLA R--------- ---------I QDPQMTGYVS TRYYRAPEIM LTWQKYDV-- ---------- ----------
      61586  ---------- -RGLKYVHSA GVVHRDL--- ------KP-S NILVNENCDL K--------- ---------- ---ICDFGLA R--------- ---------I QDPQMTGYVS TRYYRAPEIM LTWQKYDV-- ---------- ----------
      74488  ---------- -RGLKYVHSA GVVHRDL--- ------KP-S NILVNENCDL K--------- ---------- ---ICDFGLA R--------- ---------I QDPQMTGYVS TRYYRAPEIM LTWQKYDV-- ---------- ----------
     155373  ---------- -RGLKYLHSA GVVHRDL--- ------KP-S NILVNENCDL K--------- ---------- ---ICDFGLA R--------- ---------I QDPQMTGYVS TRYYRAPEIM LTWQKYDV-- ---------- ----------
      56185  ---------- -RGLKYVHSA GVVHRDL--- ------KP-S NILVNENCDL K--------- ---------- ---ICDFGLA R--------- ---------L ADPQMTGYVS TRYYRAPEIM LTWQKYDV-- ---------- ----------
      14909  ---------- -RGLKYVHSA GVVHRDL--- ------KP-S NILVNENCDL K--------- ---------- ---ICDFGLA R--------- ---------L ADPQMTGYVS TRYYRAPEIM LTWQKYDV-- ---------- ----------
     176707  ---------- -RGLKYVHSA GVVHRDL--- ------KP-S NILVNENCDL K--------- ---------- ---ICDFGLA R--------- ---------I QDPQMTGYVS TRYYRAPEIM LTWQKYDV-- ---------- ----------
     176721  ---------- -RGLKYVHSA GVVHRDL--- ------KP-S NILVNENCDL K--------- ---------- ---ICDFGLA R--------- ---------I QDPQMTGYVS TRYYRAPEIM LTWQKYDV-- ---------- ----------
     240717  ---------- -RGLKYVHSA GVVHRDL--- ------KP-S NILVNENCDL K--------- ---------- ---ICDFGLA R--------- ---------I QDPQMTGYVS TRYYRAPEIM LTWQKYDV-- ---------- ----------
      56428  ---------- -RGLKYVHSA GVIHRDL--- ------KP-S NILVNENCDL K--------- ---------- ---ICDFGLA R--------- ---------I QDPQMTGYVS TRYYRAPEIM LTWQKYDV-- ---------- ----------
      97915  ---------- -RGLKYVHSA GVIHRDL--- ------KP-S NILVNENCDL K--------- ---------- ---ICDFGLA R--------- ---------I QDPQMTGYVS TRYYRAPEIM LTWQKYDV-- ---------- ----------
     707387  ---------- -RGLKYVHSA GVVHRDL--- ------KP-S NILVNENCDL K--------- ---------- ---ICDFGLA R--------- ---------I QDPQMTGYVS TRYYRAPEIM LTWQKYDV-- ---------- ----------
     504013  ---------- -RGLKYVHSA GVVHRDL--- ------KP-S NILVNENCDL K--------- ---------- ---ICDFGLA R--------- ---------I QDPQMTGYVS TRYYRAPEIM LTWQKYDV-- ---------- ----------
      37843  ---------- -RGLKYVHSA GVVHRDL--- ------KP-S NILVNENCDL K--------- ---------- ---ICDFGLA R--------- ---------I QDPQMTGYVS TRYYRAPEIM LTWQKYDV-- ---------- ----------
     507853  ---------- -RGLKYVHSA GVVHRDL--- ------KP-S NILVNENCDL K--------- ---------- ---ICDFGLA R--------- ---------I QDPQMTGYVS TRYYRAPEIM LTWQKYDV-- ---------- ----------
     338747  ---------- -RGLKYVHSS GVVHRDL--- ------KP-S NILVNENCDL K--------- ---------- ---ICDFGLA R--------- ---------I QDPQMTGYVS TRYYRAPEIM LTWQKYDV-- ---------- ----------
      49060  ---------- -RGLKYVHSA GVVHRDL--- ------KP-S NILVNENCDL K--------- ---------- ---ICDFGLA R--------- ---------I QDPQMTGYVS TRYYRAPEIM LTWQKYDV-- ---------- ----------
     147551  ---------- -RGLKYVHSA GVVHRDL--- ------KP-S NILVNENCDL K--------- ---------- ---ICDFGLA R--------- ---------I QDPQMTGYVS TRYYRAPEIM LTWQKYDV-- ---------- ----------
     286873  ---------- -RGLKYVHSA GVVHRDL--- ------KP-S NILVNENCDL K--------- ---------- ---ICDFGLA R--------- ---------I QDPQMTGYVS TRYYRAPEIM LTWQKYDV-- ---------- ----------
     193904  ---------- -RGLKYVHSA GVVHRDL--- ------KP-S NILINENCDL K--------- ---------- ---ICDFGLA R--------- ---------I QDPQMTGYVS TRYYRAPEIM LTWQKYDV-- ---------- ----------
     189288  ---------- -RGLKYVHSA GVVHRDL--- ------KP-S NILVNENCDL K--------- ---------- ---ICDFGLA R--------- ---------I QDPQMTGYVS TRYYRAPEIM LTWQKYDV-- ---------- ----------
     198339  ---------- -RGLKYVHSA GVVHRDL--- ------KP-S NILINENCDL K--------- ---------- ---ICDFGLA R--------- ---------I QDPQMTGYVS TRYYRAPEIM LTWQKYDV-- ---------- ----------
    2140409  ---------- -RGLKYVHSA GVVHRDL--- ------KP-S NILVNENCDL K--------- ---------- ---ICDFGLA R--------- ---------I QDPQMTGYVS TRYYRAPEIM LTWQKYDV-- ---------- ----------
     642177  ---------- -RGLKYVHSA GVVHRDL--- ------KP-S NILVNENCDL K--------- ---------- ---ICDFGLA R--------- ---------I QDPQMTGYVS TRYYRAPEIM LTWQKYDV-- ---------- ----------
     498471  ---------- -RGLKYVHSA GVVHRDL--- ------KP-S NILVNENCDL K--------- ---------- ---ICDFGLA R--------- ---------I QDPQMTGYVS TRYYRAPEIM LTWQKYDV-- ---------- ----------
     784216  ---------- -RGLKYVHSA GVVHRDL--- ------KP-S NILVNENCDL K--------- ---------- ---ICDFGLA R--------- ---------I QDPQMTGYVS TRYYRAPEIM LTWQKYDV-- ---------- ----------
     128268  ---------- -RGLKYVHSA GVVHRDL--- ------KP-S NILVNENCDL K--------- ---------- ---ICDFGLA R--------- ---------I QDPQMTGYVS TRYYRAPEIM LTWQKYDV-- ---------- ----------
     246689  ---------- -RGLKYVHSA GVVHRDL--- ------KP-S NILVNENCDL K--------- ---------- ---ICDFGLA R--------- ---------I QDPQMTGYVS TRYYRAPEIM LTWQKYDV-- ---------- ----------
    1081594  ---------- -RGLKYVHSA GVVHRDL--- ------KP-S NILVNENCDL K--------- ---------- ---ICDFGLA R--------- ---------I QDPQMTGYVS TRYYRAPEIM LTWQKYDV-- ---------- ----------
     603877  ---------- -RGLKYVHSA GVVHRDL--- ------KP-S NILVNENCDL K--------- ---------- ---ICDFGLA R--------- ---------I QDPQMTGYVS TRYYRAPEIM LTWQKYDV-- ---------- ----------
     140829  ---------- -RGLKYVHSA GVVHRDL--- ------KP-S NILVNENCDL K--------- ---------- ---ICDFGLA R--------- ---------I QDPQMTGYVS TRYYRAPEIM LTWQKYDV-- ---------- ----------
     327107  ---------- -RGLKYVHSA GVVHRDL--- ------KP-S NILINENCDL K--------- ---------- ---ICDFGLA R--------- ---------I QDPQMTGYVS TRYYRAPEIM LTWQKYDV-- ---------- ----------
     329694  ---------- -RGLKYVHSA GVVHRDL--- ------KP-S NILINENCDL K--------- ---------- ---ICDFGLA R--------- ---------I QDPQMTGYVS TRYYRAPEIM LTWQKYDV-- ---------- ----------
     393439  ---------- -RGLKYVHSA GVVHRDL--- ------KP-S NILINENCDL K--------- ---------- ---ICDFGLA R--------- ---------I QDPQMTGYVS TRYYRAPEIM LTWQKYDV-- ---------- ----------
      38942  ---------- -RGLKYVHSA GVVHRDL--- ------KP-S NILINENCDL K--------- ---------- ---ICDFGLA R--------- ---------I QDPQMTGYVS TRYYRAPEIM LTWQKYDV-- ---------- ----------
      87588  ---------- -RGLKYVHSA GVVHRDL--- ------KP-S NILVNENCDL K--------- ---------- ---ICDFGLA R--------- ---------I QDPQMTGYVS TRYYRAPEIM LTWQKYDV-- ---------- ----------
     443298  ---------- -RGLKYVHSA GVVHRDL--- ------KP-S NILVNENCDL K--------- ---------- ---ICDFGLA R--------- ---------I QDPQMTGYVS TRYYRAPEIM LTWQKYDV-- ---------- ----------
      53587  ---------- -RGLKYVHSA GVVHRDL--- ------KP-S NILVNENCDL K--------- ---------- ---ICDFGLA R--------- ---------I QDPQMTGYVS TRYYRAPEIM LTWQKYDV-- ---------- ----------
      70072  ---------- -RGLKYVHSA GVVHRDL--- ------KP-S NILVNENCDL K--------- ---------- ---ICDFGLA R--------- ---------L QDPQMTGYVS TRYYRAPEIM LTWQKYDV-- ---------- ----------
      13822  ---------- -RGLKYVHSA GVVHRDL--- ------KP-S NILVNENCDL K--------- ---------- ---ICDFGLA R--------- ---------I QDPQMTGYVS TRYYRAPEIM LTWQKYDV-- ---------- ----------
      25364  ---------- -RGLKYVHSA GVVHRDL--- ------KP-S NILVNENCDL K--------- ---------- ---ICDFGLA R--------- ---------L QDPQMTGYVS TRYYRAPEIM LTWQKYDV-- ---------- ----------
     116576  ---------- -RGLKYVHSA GVVHRDL--- ------KP-S NILINENCDL K--------- ---------- ---ICDFGLA R--------- ---------I QDPQMTGYVS TRYYRAPEIM LTWQKYDV-- ---------- ----------
     103311  ---------- -RGLKYVHSA GVIHRDL--- ------KP-S NILINENCDL K--------- ---------- ---ICDFGLA R--------- ---------I QDPQMTGYVS TRYYRAPEIM LTWQKYDT-- ---------- ----------
      26173  ---------- -RGLKYIHSA GVIHRDL--- ------KP-S NILINENCDL K--------- ---------- ---ICDFGLA R--------- ---------I QDPQMTGYVS TRYYRAPEIM LTWQKYDT-- ---------- ----------
      55352  ---------- -RGLKYIHSA GVIHRDL--- ------KP-S NILINENCDL K--------- ---------- ---ICDFGLA R--------- ---------I QDPQMTGYVS TRYYRAPEIM LTWQKYDT-- ---------- ----------
     125207  ---------- -RGLKYVHSA GVIHRDL--- ------KP-S NILINENCDL K--------- ---------- ---ICDFGLA R--------- ---------I QDPQMTGYVS TRYYRAPEIM LTWQKYDV-- ---------- ----------
      67307  ---------- -RGLKYVHSA GVIHRDL--- ------KP-S NILINENCDL K--------- ---------- ---ICDFGLA R--------- ---------I QDPQMTGYVS TRYYRAPEIM LTWQKYDV-- ---------- ----------
     185764  ---------- -RGLKYVHSA GVIHRDL--- ------KP-S NILINENCDL K--------- ---------- ---ICDFGLA R--------- ---------I QDPQMTGYVS TRYYRAPEIM LTWQKYDV-- ---------- ----------
      99366  ---------- -RGLKYVHSA GVIHRDL--- ------KP-S NILINENCDL K--------- ---------- ---ICDFGLA R--------- ---------I QDPQMTGYVS TRYYRAPEIM LTWQKYDT-- ---------- ----------
      50111  ---------- -RGLKYVHSA GVVHRDL--- ------KP-S NILVNENCDL K--------- ---------- ---ICDFGLA R--------- ---------I QDPQMTGYVS TRYYRAPEIM LTWQKYDV-- ---------- ----------
      87970  ---------- -RGLKYVHSA GVVHRDL--- ------KP-S NILVNENCDL K--------- ---------- ---ICDFGLA R--------- ---------L QDPQMTGYVS TRYYRAPEIM LTWQKYDV-- ---------- ----------
     107532  ---------- -RGLKYVHSA GVVHRDL--- ---------- ---------- ---------- ---------- ---ICDFGLA R--------- ---------L QDPQMTGYVS TRYYRAPEIM LTWQKYDV-- ---------- ----------
     604754  ---------- -RGLKYVHSA GVVHRDL--- ------KP-S NILVNENCDL K--------- ---------- ---ICDFGLA R--------- ---------I QDPQMTGYVS TRYYRAPEIM LTWQKYDV-- ---------- ----------
     633130  ---------- -RGLKYVHSA GVVHRDLLPT KISFTQKP-S NILVNENCDL K--------- ---------- ---ICDFGLA R--------- ---------I QDPQMTGYVS TRYYRAPEIM LTWQKYDV-- ---------- ----------
     181927  ---------- -RGLKYVHSA GVIHRDL--- ------KP-R NILVNENCDL K--------- ---------- ---ICDFGLA R--------- ---------V LDYQMTGYVA TRYYRAPEIM LTWQKYDI-- ---------- ----------
      69161  ---------- -RALKFVHPA GVVHRDL--- ------KP-S NILINENCDL K--------- ---------- ---ICDFGLA R--------- ---------L QDPQMTGYVS TRYYRAPEIM LTWQEYDS-- ---------- ----------
```

```
  35549  ---------- -RGLKYVHSA GVVHRDL--- ------KP-S NILINENCDL K--------- ---------- ---ICDFGLA R--------- ---------I QDPQMTGYVS TRYYRAPEIM LTWQKYDV-- ---------- ----------
 230139  ---------- -RGLKYVHSA GVVHRDL--- ------KP-S NILINENCDL K--------- ---------- ---ICDFGLA R--------- ---------I QDPQMTGYVS TRYYRAPEIM LTWQKYDV-- ---------- ----------
 193325  ---------- -RGLKYVHSA GVVHRDL--- ------KP-S NILINENCDL K--------- ---------- ---ICDFGLA R--------- ---------I QDPQMTGYVS TRYYRAPEIM LTWQKYDV-- ---------- ----------
 430049  ---------- -RGLKYVHSA GVVHRDL--- ------KP-S NILINENCDL K--------- ---------- ---ICDFGLA R--------- ---------I QDPQMTGYVS TRYYRAPEIM LTWQKYDV-- ---------- ----------
 479652  ---------- -RGLKYVHSA GVVHRDL--- ------KP-S NILINENCDL K--------- ---------- ---ICDFGLA R--------- ---------I QDPQMTGYVS TRYYRAPEIM LTWQKYDV-- ---------- ----------
 986652  ---------- -RGLKYVHSA GVVHRDL--- ------KP-S NILVNENCDL K--------- ---------- ---ICDFGLA R--------- ---------I QDPQMTGYVS TRYYRAPEIM LTWQKYDV-- ---------- ----------
 295607  ---------- -RGLKYVHSA GVVHRDL--- ------KP-S NILVNENCDL K--------- ---------- ---ICDFGLA R--------- ---------I QDPQMTGYVS TRYYRAPEIM LTWQKYDV-- ---------- ----------
 114099  ---------- -RGLKYVHSA GVVHRDL--- ------KP-S NILVNENCDL K--------- ---------- ---ICDFGLA R--------- ---------I QDPQMTGYVS TRYYRAPEIM LTWQKYDV-- ---------- ----------
 265170  ---------- -RGLKYVHSA GVVHRDL--- ------KP-S NILVNENCDL K--------- ---------- ---ICDFGLA R--------- ---------I QDPQMTGYVS TRYYRAPEIM LTWQKYDV-- ---------- ----------
  41804  ---------- -RGLKYVHSA GVVHRDL--- ------KP-S NILINENCDL K--------- ---------- ---ICDFGLA R--------- ---------I QDPQMTGYVS TRYYRAPEIM LSWQKYDI-- ---------- ----------
 500846  ---------- -RGLKYVHSA GVVHRDL--- ------KP-S NILVNENCDL K--------- ---------- ---ICDFGLA R--------- ---------I QDPQMTGYVS TRYYRAPEIM LTWQKYDV-- ---------- ----------
 341622  ---------- -RGLKYVHSA GVIHRDL--- ------KP-S NILVNENCDL K--------- ---------- ---ICDFGLA R--------- ---------I QDPQMTGYVS TRYYRAPEIM LTWQKYDV-- ---------- ----------
1545705  ---------- -RGLKYVHSA GVVHRDL--- ------KP-S NILINENCDL K--------- ---------- ---ICDFGLA R--------- ---------I QDPQMTGYVS TRYYRAPEIM LTWQKYDV-- ---------- ----------
  15276  ---------- -RGLKYVHSA GVVHRDL--- ------KP-S NILINENCDL K--------- ---------- ---ICDFGLA R--------- ---------I QDPQMTGYVS TRYYRAPEIM LTWQKYDV-- ---------- ----------
 391543  ---------- -RGLKYLHSA GVVHRDL--- ------KP-S NILINENCDL K--------- ---------- ---ICDFGLA R--------- ---------V QEHNMTGYVS TRYYRAPEIM LTWRKYNE-- ---------- ----------
 346451  ---------- -RGLKYLHSA GVVHRDL--- ------KP-S NILINENCDL K--------- ---------- ---ICDFGLA R--------- ---------V QEHNMTGYVS TRYYRAPEIM LTWRKYNE-- ---------- ----------
 449365  ---------- -RGLKYIHSA GVVHRDL--- ------KP-S NILVNENCDL K--------- ---------- ---ICDFGLA R--------- ---------V QELQMTGYVA TRYYRAPEIM LTWRRYNE-- ---------- ----------
  54661  ---------- -RGLKYVHSA GVVHRDL--- ------KP-S NILINENCDL K--------- ---------- ---ICDFGLA R--------- ---------I QDPQMTGYVS TRYYRAPEIM LTWQKYDV-- ---------- ----------
 275309  ---------- -RGMKYIHSA GIIHRDL--- ------KP-S NILINDNCDL K--------- ---------- ---ICDFGLA R--------- ---------E QDAQMTGYVS TRYYRAPEIM LTWQKYTY-- ---------- ----------
 389883  ---------- -RGLKYIHSA GVIHRDL--- ------KP-S NLLINDNCDL K--------- ---------- ---ICDFGLA R--------- ---------E QDYQMTGYVT TRYYRAPEIM LTWQKYTH-- ---------- ----------
  61624  ---------- -RGLKYIHSA GVVHRDL--- ------KP-S NILINRNCDV K--------- ---------- ---ICDFGLA R--------- ---------I QDFEMTGYVS TRYYRAPETM LTWGNYGP-- ---------- ----------
 173235  ---------- -RGLKYIHSA GVVHRDL--- ------KP-S NILINRNCDV K--------- ---------- ---ICDFGLA R--------- ---------I QDFEMTGYVS TRYYRAPETM LTWGNYGP-- ---------- ----------
  35612  ---------- -RGLKYVHSA GVVHRDL--- ------KP-S NILINENCDL K--------- ---------- ---ICDFGLA R--------- ---------I QDPQMTGYVS TRYYRAPEIM LTWQKYDV-- ---------- ----------
  35597  ---------- -RGLKYVHSA GVVHRDL--- ------KP-S NILINENCDL K--------- ---------- ---ICDFGLA R--------- ---------I QDPQMTGYVS TRYYRAPEIM LTWQKYDV-- ---------- ----------
 391476  ---------- -RGLKYIHSA GVVHRDL--- ------KP-S NVLVDENCDI K--------- ---------- ---LCDFGLA R--------- ---------T VDTTMTGYVT MRHYRAPETM LTWQKYTV-- ---------- ----------
 335269  ---------- -RGLKYIHSA GVVHRDL--- ------KP-S NVLVDENCDI K--------- ---------- ---LCDFGLA R--------- ---------T VDTTMTGYVT MRHYRAPETM LTWQKYTV-- ---------- ----------
 377162  ---------- -RGLKYIHSA GVVHRDL--- ------KP-S NVLVDENCDI K--------- ---------- ---LCDFGLA R--------- ---------T VDTTMTGYVT MRHYRAPETM LTWQKYTV-- ---------- ----------
 271149  ---------- -RGLKYVHSA GVVHRDL--- ------KP-S NILVNENCDL K--------- ---------- ---ICDFGLA R--------- ---------I QDPQMTGYVS TRYYRAPEIM LTWQKYDV-- ---------- ----------
  89784  ---------- -RGLKYVHSA GVVHRDL--- ------KP-S NILINENCDL K--------- ---------- ---ICDFGLA R--------- ---------I QDPQMTGYVS TRYYRAPEIM LTWQKYDV-- ---------- ----------
 114108  ---------- --SAHLPSAR EAVHPIL--- -------S-L PDPLNENCDL K--------- ---------- ---ICDFGLA R--------- ---------I QDPQMTGYVS TRYYRAPEIM LTWQKYDV-- ---------- ----------
 173207  ---------M LSGLQYIHAA NVIHRDL--- ------KP-A NILVNVNCDI K--------- ---------- ---ICDFGMA REYR------ ----AQDGR- QSAFLTEYVT TRWYRAPEVM ISPNNYSKS- ---------- ----------
 127887  ---------L FSGLKYIHSA NVLHRDL--- ------KP-A NILVNENCDL R--------- ---------- ---ICDFGMA RGYS------ ----SQSSQL EAMNMTHYVV TRWYRAPEVM LSPNTYDKA- ---------- ----------
  29226  ---------I LSALKYIHSA HVLHRDL--- ------KP-A NILVNQNCDI R--------- ---------- ---ICDFGMA RGIV------ ----QPSSLH ETPSMTHYVV TRWYRAPEIM MSRNSYDKA- ---------- ----------
 167765  ---------L FSGLKYIHSA NVLHRDL--- ------KP-A NILVNENCDL R--------- ---------- ---ICDFGMA RGFA------ ----RSNAQI ESQYMTHYVV TRWYRSPEIM LSRKTYDKP- ---------- ----------
 150384  ---------L FSGLKYIHSA NVLHRDL--- ------KP-A NILVNENCDL R--------- ---------- ---ICDFGMA RGFA------ ----RSNAQI ESQYMTHYVV TRWYRSPEIM LSRKTYDKP- ---------- ----------
  57923  ---------L FSGLKYIHSA NVLHRDL--- ------KP-A NILVNENCDL R--------- ---------- ---ICDFGMA RGIA------ ----KPSAKV ELQNMTHYVV TRWYRAPEIM LSRNSYDKA- ---------- ----------
  57928  ---------L FSGLKYIHSA NVLHRDL--- ------KP-A NILVNENCDL R--------- ---------- ---ICDFGMA RGIA------ ----KPSAKV ELQNMTHYVV TRWYRAPEIM LSRNSYDKA- ---------- ----------
 230955  ---------I LTGLKYIHSA DVLHRDL--- ------KP-A NILVNQNCDI R--------- ---------- ---ICDFGMA RGHI------ -----DDQNY ESWNLTQYVS TRWYRAPEIM LSRNSYNKS- ---------- ----------
 172033  ---------I LTGLKYIHSA DVLHRDL--- ------KP-A NILVNQNCDI R--------- ---------- ---ICDFGMA RGHI------ -----DDQNY ESWNLTQYVS TRWYRAPEIM LSRNSYNKS- ---------- ----------
  40306  ---------I LCGLKYIHSA NVLHRDL--- ------KP-G NLLVNADCEL K--------- ---------- ---ICDFGLA RGFS------ -----ADPAE NAGFLTEYVA TRWYRAPEIM LSFQSYTKA- ---------- ----------
 693957  ---------I LCGLKYIHSA NVLHRDL--- ------KP-G NLLVNADCEL K--------- ---------- ---IADFGLA RGFS------ -----MDPEE NAGYMTEYVA TRWYRAPEIM LSFQSYTKA- ---------- ----------
 508525  ---------I LCGLKYIHSA NVLHRDL--- ------KP-G NLLVNADCEL K--------- ---------- ---IADFGLA RGFS------ -----MDPEE NAGYMTEYVA TRWYRAPEIM LSFQSYTKA- ---------- ----------
 471145  ---------I LCGLKYIHSA NVLHRDL--- ------KP-G NLLVNADCEL K--------- ---------- ---ICDFGLA RGFS------ -----MDPEE NAGYMTEYVA TRWYRAPEIM LSFQSYTKA- ---------- ----------
 480536  ---------I LCGLKYIHSA NVLHRDL--- ------KP-G NLLVNADCEL K--------- ---------- ---ICDFGLA RGFS------ -----MDPEE NAGYMTEYVA TRWYRAPEIM LSFQSYTKA- ---------- ----------
 471745  ---------I LCGLKYIHSA NVLHRDL--- ------KP-G NLLVNADCEL K--------- ---------- ---ICDFGLA RGFS------ -----MDPEE NAGYMTEYVA TRWYRAPEIM LSFQSYTKA- ---------- ----------
 687176  ---------I LCGLKYIHSA NVLHRDL--- ------KP-G NLLVNADCEL K--------- ---------- ---ICDFGLA RGFS------ -----MDPEE NAGYMTEYVA TRWYRAPEIM LSFQSYTKA- ---------- ----------
  34863  ---------I LCGLKYIHSA NVLHRDL--- ------KP-G NLLVNADCEL K--------- ---------- ---ICDFGLA RGFS------ -----VDPEE NAGYMTEYVA TRWYRAPEIM LSFQSYTKA- ---------- ----------
  57153  ---------I LCGLKYIHSA NVLHRDL--- ------KP-G NLLVNADCEL K--------- ---------- ---IADFGLA RGFS------ -----VDPEE NAGYMTEYVA TRWYRAPEIM LSFQSYTKA- ---------- ----------
 147961  ---------I LCGLKYIHSA NVLHRDL--- ------KP-G NLLVNADCEL K--------- ---------- ---IADFGLA RGFS------ -----IDPEE NAGYMTEYVA TRWYRAPEIM LSFQSYTKA- ---------- ----------
 287354  ---------I LCGLKYIHSA NVLHRDL--- ------KP-G NLLVNADCEL K--------- ---------- ---ICDFGLA RGFS------ -----VDPEE NAGYMTEYVA TRWYRAPEIM LSFQSYTKA- ---------- ----------
 281614  ---------I LCGLKYIHSA NVLHRDL--- ------KP-G NLLVNADCEL K--------- ---------- ---IADFGLA RGFS------ -----MDPEE NAGYMTEYVA TRWYRAPEIM LSFQSYTKA- ---------- ----------
  29684  ---------I LCGLKYIHSA NVLHRDL--- ------KP-G NLLVNADCEL K--------- ---------- ---ICDFGLA RGFS------ -----VDPEE NAGYMTEYVA TRWYRAPEIM LSFQSYTKA- ---------- ----------
 144400  ---------I LCGLKYIHSA NVLHRDL--- ------KP-G NLLVNADCEL K--------- ---------- ---ICDFGLA RGFS------ -----VDPEE NAGYMTEYVA TRWYRAPEIM LSFQNYTKAS MFLSLKQAVP LSDVSRLTLK
 136175  ---------I LCGLKYIHSA NVLHRDL--- ------KP-G NLLVNADCEL K--------- ---------- ---ICDFGLA RGFS------ -----VDPEE NAGYMTEYVA TRWYRAPEIM LSFQNYTKA- ---------- ----------
 126818  ---------I LCGLKYIHSA NVLHRDL--- ------KP-G NLLVNADCEL K--------- ---------- ---ICDFGLA RGFS------ -----VDPEE NAGYMTEYVA TRWYRAPEIM LSFQNYTKA- ---------- ----------
2138682  ---------I LCGLKYIHSA NVLHRDL--- ------KP-G NLLVNADCEL K--------- ---------- ---ICDFGLA RGFS------ -----IDPEE NAGYMTEYVA TRWYRAPEIM LSFQNYTKA- ---------- ----------
  48096  ---------I LCGLKYIHSA NVLHRDL--- ------KP-G NLLVNADCEL K--------- ---------- ---ICDFGLA RGFS------ -----IDPEE NAGYMTEYVA TRWYRAPEIM LSFQNYTKA- ---------- ----------
 179022  ---------I LCGLKYIHSA NVLHRDL--- ------KP-G NLLVNADCEL K--------- ---------- ---ICDFGLA RGFS------ -----VDPEE NAGYMTEYVA TRWYRAPEIM LSFQNYTKA- ---------- ----------
 778216  ---------I LCGLKYIHSA NVLHRDL--- ------KP-G NLLVNADCEL K--------- ---------- ---IADFGLA RGFS------ -----IDPEE NAGYMTEYVA TRWYRAPEIM LSFQNYTKA- ---------- ----------
 665550  ---------I LCGLKYIHSA NVLHRDL--- ------KP-G NLLVNADCEL K--------- ---------- ---ICDFGLA RGFS------ -----VDPEE NAGYMTEYVA TRWYRAPEIM LSFQSYTKA- ---------- ----------
 190738  ---------I LCGLKYIHSA NVLHRDL--- ------KP-G NLLVNADCEL K--------- ---------- ---ICDFGLA RGFS------ -----VDPEE NAGYMTEYVA TRWYRAPEIM LSFQSYTKA- ---------- ----------
1107049  ---------I LCGLKYIHSA NVLHRDL--- ------KP-G NLLVNADCEL K--------- ---------- ---ICDFGLA RGFS------ -----VDPEE NAGYMTEYVA TRWYRAPEIM LSFQSYTKA- ---------- ----------
 504269  ---------I LCGLKYIHSA NVLHRDL--- ------KP-G NLLVNADCEL K--------- ---------- ---IADFGLA RGFS------ -----IDPEE NAGYMTEYVA TRWYRAPEIM LSFQNYTKA- ---------- ----------
 432134  ---------I LCGLKYIHSA NVLHRDL--- ------KP-G NLLVNADCEL K--------- ---------- ---IADFGLA RGFS------ -----IDPEE NAGYMTEYVA TRWYRAPEIM LSFQNYTKA- ---------- ----------
  59068  ---------I LCGLKYIHSA NVLHRDL--- ------KP-G NLLVNADCEL K--------- ---------- ---ICDFGLA RGFS------ -----IDPEE NAGYMTEYVA TRWYRAPEIM LSFQNYTKA- ---------- ----------
 336185  ---------I LCGLKYIHSA NVLHRDL--- ------KP-G NLLVNADCEL K--------- ---------- ---ICDFGLA RGFS------ -----IDPEE NAGYMTEYVA TRWYRAPEIM LSFQNYTKA- ---------- ----------
 343155  ---------I LCGLKYIHSA NVLHRDL--- ------KP-G NLLVNADCEL K--------- ---------- ---ICDFGLA RGFS------ -----IDPEE NAGYMTEYVA TRWYRAPEIM LSFQSYTKA- ---------- ----------
 326648  ---------I LCGLKYIHSA NVLHRDL--- ------KP-G NLLVNADCEL K--------- ---------- ---ICDFGLA RGFS------ -----IDPEE NAGYMTEYVA TRWYRAPEIM LSFQSYTKA- ---------- ----------
 425863  ---------I LCGLKYIHSA NVLHRDL--- ------KP-G NLLVNADCEL K--------- ---------- ---ICDFGLA RGFS------ -----IDPEE NAGYMTEYVA TRWYRAPEIM LSFQSYTKA- ---------- ----------
 436998  ---------I LCGLKYIHSA NVLHRDL--- ------KP-G NLLVNADCEL K--------- ---------- ---ICDFGLA RGFS------ -----IDPEE NAGYMTEYVA TRWYRAPEIM LSFQSYTKA- ---------- ----------
 257317  ---------I LCGLKYIHSA NVLHRDL--- ------KP-G NLLVNADCEL K--------- ---------- ---ICDFGLA RGFS------ -----IDPEE NAGYMTEYVA TRWYRAPEIM LSFQSYTKA- ---------- ----------
 998070  ---------I LCGLKYIHSA NVLHRDL--- ------KP-G NLLVNADCEL K--------- ---------- ---ICDFGLA RGFS------ -----MDPEE NAGYMTEYVA TRWYRAPEIM LSFQSYTKA- ---------- ----------
  49449  ---------I LCGLKYIHSA NVLHRDL--- ------KP-G NLLVNADCEL K--------- ---------- ---ICDFGLA RGFS------ -----MDPEE NAGYMTEYVA TRWYRAPEIM LSFQSYTKA- ---------- ----------
 498377  ---------I LCGLKYIHSA NVLHRDL--- ------KP-G NLLVNADCEL K--------- ---------- ---ICDFGLA RGFS------ -----IDPEE NAGYMTEYVA TRWYRAPEIM LSFQSYTKA- ---------- ----------
 184715  ---------I LCGLKYIHSA NVLHRDL--- ------KP-G NLLVNADCEL K--------- ---------- ---IADFGLA RGFS------ -----IDPEE NAGYMTEYVA TRWYRAPEIM LSFQNYTKA- ---------- ----------
  51223  ---------I LCGLKYIHSA NVIHRDL--- ------KP-G NLLVNADCEL K--------- ---------- ---ICDFGLA RGFS------ -----ENPEE NAGYMTEYVA TRWYRAPEIM LSFQSYTKA- ---------- ----------
  66669  ---------I LCGLKYIHSA NVLHRDL--- ------KP-G NLLVNADCEL K--------- ---------- ---ICDFGLA RGFS------ -----ENPEE NAGYMTEYVA TRWYRAPEIM LSFQSYTKA- ---------- ----------
 649898  ---------I LCGLKYIHSA NVLHRDL--- ------KP-G NLLVNADCEL K--------- ---------- ---ICDFGLA RGFS------ -----VDPEE NAGYMTEYVA TRWYRAPEIM LSFLSYTKA- ---------- ----------
 253817  ---------I LCGLKYIHSA NVLHRDL--- ------KP-G NLLVNADCEL K--------- ---------- ---ICDFGLA RGFS------ -----VDPEE NAGYMTEYVA TRWYRAPEIM LSFLSYTKA- ---------- ----------
 618068  ---------I LCGLKYIHSA NVLHRDL--- ------KP-G NLLVNADCEL K--------- ---------- ---IADFGLA RGFS------ -----IDPEE NAGYMTEYVA TRWYRAPEIM LSFQNYTKA- ---------- ----------
 108847  ---------I LCGLKYIHSA DVLHRDL--- ------KP-G NLLVNVECDL K--------- ---------- ---ICDFGLA RGFS------ -----EDVEQ NSGFMTEYVA TRWYRAPEIM LSFSNYTKA- ---------- ----------
  33629  ---------V LCGLKYIHLA DVLHRDL--- ------KP-G NLLVNADCEL K--------- ---------- ---ICDFGLA RGFS------ -----ENPEQ NAGYMTEYVA TRWYRAPEIM LSFTNYSKA- ---------- ----------
  54715  ---------V LCGLKYIHSA DVLHRDL--- ------KP-G NLLVNADCEL K--------- ---------- ---ICDFGLA RGFS------ -----ENPEQ NSGFMTEYVA TRWYRAPEIM LSFSSYTKA- ---------- ----------
  88920  ---------L LCGLKYIHSA NVLHRDL--- ------KP-G NLLVNADCEL K--------- ---------- ---ICDFGLA RGYS------ -----SHPDT NAGFMTEYVA TRWYRAPEIM LSFQSYTKA- ---------- ----------
  51479  ---------I LTGLKYIHSA DVLHRDL--- ------KP-G NLLVNADCEL K--------- ---------- ---ICDFGLA RGYS------ -----SNEAK NAGFLTEYVA TRWYRAPEIM LSFQAYTKA- ---------- ----------
  86943  ---------I LCGLKYIHSA DVLHRDL--- ------KP-G NLLVNADCEL K--------- ---------- ---ICDFGLA RGYS------ -----NDPEK NAGYLTEYVA TRWYRAPEIM LSFQSYTKA- ---------- ----------
 100835  ---------I LCGLKYIHSA DVLHRDL--- ------KP-G NLLVNADCEL K--------- ---------- ---ICDFGLA RGYS------ -----SIPEK NAGFLTEYVA TRWYRAPEIM LSFQSYTKA- ---------- ----------
 319719  ---------I LCGLKYIHSA DVLHRDL--- ------KP-G NLLVNADCQL K--------- ---------- ---ICDFGLA RGYS------ -----ENPVE NSQFLTEYVA TRWYRAPEIM LSYQGYTKA- ---------- ----------
 822494  ---------I LCGLKYIHSA DVLHRDL--- ------KP-G NLLVNADCQL K--------- ---------- ---ICDFGLA RGYS------ -----ENPVE NSQFLTEYVA TRWYRAPEIM LSYQGYTKA- ---------- ----------
 161919  ---------I CRGLKYIHSA NVLHRDL--- ------KP-G NLLVNADCEL K--------- ---------- ---ICDFGLA RGYS------ -----SSPEG NAGFMTEYVA TRWYRAPEIM LSFQKYTKA- ---------- ----------
 158631  ---------I CRGLKYIHSA NVLHRDL--- ------KP-G NLLVNADCEL K--------- ---------- ---ICDFGLA RGYS------ -----ENDEH NVGFMTEYVA TRWYRSPEIM LSFQNYTKA- ---------- ----------
 115223  ---------I CRGLKYIHSA NVLHRDL--- ------KP-G NLLVNADCEL K--------- ---------- ---ICDFGLA RGYS------ -----DNPDY NAGFMTEYVA TRWYRAPEIM LSFQSYTKA- ---------- ----------
 344665  ---------I CRGLKYIHSA NVLHRDL--- ------KP-G NLLVNADCEL K--------- ---------- ---ICDFGLA RGFS------ -----DSPDH NAGFMTEYVA TRWYRAPEIM LSFQNYTKA- ---------- ----------
 178050  ---------L CRGLKYIHSA NVLHRDL--- ------KP-G NLLVNADCEL K--------- ---------- ---ICDFGLA RGYS------ -----PELDT TAGFMTEYVA TRWYRAPEIM LSFQNYTKA- ---------- ----------
 234927  ---------I CRGLKYIHSA NVLHRDL--- ------KP-G NLLVNADCEL K--------- ---------- ---ICDFGLA RGFS------ -----DSPDS NQGFMTEYVA TRWYRAPEIM LSFQNYTKA- ---------- ----------
 328916  ---------I LNGLKYIHSA NVLHRDL--- ------KP-G NLLVNADCEL K--------- ---------- ---ICDFGLA RGFS------ -----EDPAA NSGFMTEYVA TRWYRAPEIM LSFQSYTKA- ---------- ----------
 273327  ---------I LNGLKYIHSA NVLHRDL--- ------KP-G NLLVNADCEL K--------- ---------- ---ICDFGLA RGFS------ -----EDPAA NSGFMTEYVA TRWYRAPEIM LSFQSYTKA- ---------- ----------
 338804  ---------I LNGLKYIHSA NVLHRDL--- ------KP-G NLLVNADCEL K--------- ---------- ---ICDFGLA RGFS------ -----EDPAA NSGFMTEYVA TRWYRAPEIM LSFQSYTKA- ---------- ----------
 527701  ---------T LCGLKYIHSA NVLHRDL--- ------KP-G NLLVNADCEL K--------- ---------- ---ICDFGLA RGYSP----- -GGGTSKAAG NQGFMTEYVA TRWYRAPEIM LSFANYTTA- ---------- ----------
  81317  ---------T LCGLKYIHSA NVLHRDL--- ------KP-G NLLVNADCEL K--------- ---------- ---ICDFGLA RGY----T-- PGGGASRAAG NQGFMTEYVA TRWYRAPEIM LSFANYTTA- ---------- ----------
 831329  ---------T LCGLKYIHSA NVLHRDL--- ------KP-G NLLVNADCEL K--------- ---------- ---ICDFGLA RGF----T-- PGGGASKSAG NQGFMTEYVA TRWYRAPEIM LSFANYTTA- ---------- ----------
 676624  ---------T LCGLKYIHSA NVLHRDL--- ------KP-G NLLVNADCEL K--------- ---------- ---ICDFGLA RGF----T-- PGGGTSKSAG NQGFMTEYVA TRWYRAPEIM LSFANYTTA- ---------- ----------
 826941  ---------T LCGLKYIHSA NVLHRDL--- ------KP-G NLLVNADCEL K--------- ---------- ---ICDFGLA RGY----T-- PGGGASKSAG NQGFMTEYVA TRWYRAPEIM LSFANYTTA- ---------- ----------
  77377  ---------T LCGLKYIHSA NVLHRDL--- ------KP-G NLLVNADCEL K--------- ---------- ---ICDFGLA RGY----T-- PGGGTSKAAG NQGFMTEYVA TRWYRAPEIM LSFANYTTA- ---------- ----------
  59915  ---------T LCGLKYIHSA NVLHRDL--- ------KP-G NLLVNADCEL K--------- ---------- ---ICDFGLA RGYVY--T-- PGGGASRAAG NQGFMTEYVA TRWYRAPEIM LSFANYTTA- ---------- ----------
 160641  ---------T LCGLKYIHSA NVLHRDL--- ------KP-G NLLVNADCEL K--------- ---------- ---ICDFGLA RGYSP--G-- SGANQQRAAA NQGFMTEYVA TRWYRAPEIM LSFANYGPA- ---------- ----------
 108839  ---------T LCGLKYIHSA NVLHRDL--- ------KP-G NLLVNADCEL K--------- ---------- ---ICDFGLA RGYQP--G-- GGNNQSRAAA NQGFMTEYVA TRWYRAPEIM LSFANYGPA- ---------- ----------
1536187  ---------T LCGLKYIHSA NVLHRDL--- ------KP-G NLLVNADCEL K--------- ---------- ---ICDFGLA RGYSP--G-- GSNNQARGAA NQGFMTEYVA TRWYRAPEIM LSFANYGPA- ---------- ----------
 281708  ---------T LCGLKYIHSA NVLHRDL--- ------KP-G NLLVNADCEL K--------- ---------- ---ICDFGLA RGYSP--G-- GGNNNARGAA NQGFMTEYVA TRWYRAPEIM LSFANYGPA- ---------- ----------
 173791  ---------T LCGLKYIHSA NVLHRDL--- ------KP-G NLLVNADCEL K--------- ---------- ---ICDFGLA RGYSP--G-- SGSSQARGGA NQGFMTEYVA TRWYRAPEIM LSFANYGPA- ---------- ----------
  82703  ---------T LCGLKFIHSA NVLHRDL--- ------KP-G NLLVNADCEL K--------- ---------- ---ICDFGLA RGYSP--AAN GGTGAAKNAA NQGFMTEYVA TRWYRAPEIM LSFANYGPA- ---------- ----------
 139538  ---------T LCGLKYIHSA NVLHRDL--- ------KP-G NLLVNADCEL K--------- ---------- ---ICDFGLA RGFT------ PGSGASKSAG NQGFMTEYVA TRWYRAPEIM LSFANYTTA- ---------- ----------
 479859  ---------T LCGLKYIHSA NVLHRDL--- ------KP-G NLLVNADCEL K--------- ---------- ---ICDFGLA RGYTP--G-- AKPQNTRSTG NQGFMTEYVA TRWYRAPEIM LSFANYATA- ---------- ----------
 621303  ---------T LCGLKYIHSA NVLHRDL--- ------KP-G NLLVNADCEL K--------- ---------- ---ICDFGLA RGYTP--G-- GGTAASRGAP NLGLMTEYVA TRWYRAPEIM LSFANYSTA- ---------- ----------
  38569  ---------T LCGLKYIHSA NVLHRDL--- ------KP-G NLLVNADCEL K--------- ---------- ---ICDFGLA RGYTP--G-- GGTAASRGAP NLGLMTEYVA TRWYRAPEIM LSFANYSTA- ---------- ----------
 242842  ---------T LCGLKYIHSA NVLHRDL--- ------KP-G NLLVNADCEL K--------- ---------- ---ICDFGLA RGYTP--AG- GTAPPSKAAG NQGFMTEYVA TRWYRAPEIM LSFANYTTA- ---------- ----------
2481011  ---------T LCGLKYIHSA NVLHRDL--- ------KP-G NLLVNADCEL K--------- ---------- ---ICDFGLA RGY------- -SSGTGSASN NQGFMTEYVA TRWYRAPEVM LSFANYTTA- ---------- ----------
 169913  ---------T LCGLKYIHSA NVLHRDL--- ------KP-G NLLVNADCEL K--------- ---------- ---ICDFGLA RGF------- ------NQQQ RQGFMTEYVA TRWYRAPEIM LSFANYASA- ---------- ----------
  71742  ---------T LCGLKYIHSA NVLHRDI--- ------KP-G NLLVNADCEL K--------- ---------- ---ICDFGLA RGYTP----- -GDGASKSAG NQGFMTEYVA TRWYRAPEIM LSFANYTTA- ---------- ----------
  66635  ---------T LCGLRYIHSA NVLHRDL--- ------KP-G NLLVNADCEL K--------- ---------- ---ICDFGLA RGFDL--D-- ---NKGNDPN GGGLMTEYVA TRWYRAPEIM LSFQNYTTA- ---------- ----------
  53870  ---------T LCGLRYIHSA NVLHRDL--- ------KPAG NLLVNADCEL K--------- ---------- ---ICDFGLA RGFDL--D-- ---NKGNDPN GGGLMTEYVA TRWYRAPEIM LSFQNYTTA- ---------- ----------
  27246  ---------T LCGLKYIHSA NVLHRDL--- ------KP-G NLLVNADCEL K--------- ---------- ---ICDFGLA RGFTP--G-- GGASKSAG-- NQGFMTEYVA TRWYRAPEIM LSFANYTTA- ---------- ----------
  88978  ---------T LCGLKYIHSA NVLHRDI--- ------KP-G NLLVNADCEL K--------- ---------- ---ICDFGLA RGYTP--G-- DGASKSAG-- NQGFMTEYVA TRWYRAPEIM LSFANYTTA- ---------- ----------
1091096  ---------T LCGLKYIHSA NVLHRDL--- ------KP-G NLLVNADCEL K--------- ---------- ---ICDFGLA RGYTP--G-- GGASKSAG-- NQGFMTEYVA TRWYRAPEIM LSFANYTTA- ---------- ----------
  66856  ---------T LCGLKYIHSA NVLHRDL--- ------KP-G NLLVNADCEL K--------- ---------- ---ICDFGLA RGYSP--G-- SGSSQARGGA NQGFMTEYVA TRWYRAPEIM LSFANYGPA- ---------- ----------
  20720  ---------T LCGLKFIHSA NVLHRDL--- ------KP-G NLLVNADCEL K--------- ---------- ---ICDFGLA RGYSP--AAN GGTGAAKNAA NQGFMTEYVA TRWYRAPEIM LSFANYGPA- ---------- ----------
 124539  ---------T LCGLKYIHSA NVLHRDL--- ------KP-G NLLVNADCEL K--------- ---------- ---ICDFGLA RGYTP--G-- GGASRAAG-- NQGFMTEYVA TRWYRAPEIM LSFANYTTA- ---------- ----------
 361187  ---------I LCGLKYIHSA NVLHRDL--- ------KP-G NLLVNADCEL K--------- ---------- ---ICDFGLA RGFS------ -----VDPEE NAGYMTEYVA TRWYRAPEIM LSFQSYTKA- ---------- ----------
 327531  ---------I LCGLKYIHSA NVLHRDL--- ------KP-G NILLNADCEL K--------- ---------- ---ICDFGLA RGYTP-PGQN PGAPASKAAG NAGFMTEYVA TRWYRAPEIM LSFANYSTS- ---------- ----------
   7086  ---------I LCGLKYIHSA NVLHRDL--- ------KP-G NILLNADCEL K--------- ---------- ---ICDFGLA RGYTP-PGQN PGAPASKAAG NAGFMTEYVA TRWYRAPEIM LSFANYSTS- ---------- ----------
  28915  ---------I CRGLKYIHSA NVLHRDL--- ------KP-G NLLVNADCEL K--------- ---------- ---ICDFGLA RGFS------ -----DDAES NVGFMTEYVA TRWYRAPEIM LSFQSYTKA- ---------- ----------
 669145  ---------T LCGLKYIHSA NVLHRDL--- ------KP-G NLLVNADCEL K--------- ---------- ---ICDFGLA RGF------- ------NQQQ RQGFMTEYVA TRWYRAPEIM LSFANYASA- ---------- ----------
  46685  ---------T LCGLKYIHSA SVLHRDL--- ------KP-G NLLVNADCEL K--------- ---------- ---ICDFGLA RGFE------ -TDPALAQAS NQGFMTEYVA TRWYRAPEIM LSFQNYTTA- ---------- ----------
```

```
     53954  ---------T LCGLKYIHSA HVLHRDL--- ------KP-G NLLVNADCEL K--------- ---------- ---ICDFGLA RGFD------ -QAATDGQNN QQGFMTEYVA TRWYRAPEIM LSFANYTTA- ---------- ----------
   1446322  ---------I RSGQPL---- ----SDA--- ------HF-Q SFLYQTLCGL K--------- ---------- ---ICDFGLA RGYAPGPGGG TASANQKAAG NQGFMTEYVA TRWYRAPEIM LSFANYTTA- ---------- ----------
    351754  ---------T LCGLKYIHSA NVLHRDL--- ------KP-G NLLVNADCEL K--------- ---------- ---ICDFGLA RGYQPG-GNG KASGG---AP NQGFMTEYVA TRWYRAPEIM LSFANYTTA- ---------- ----------
     65873  ---------T LCGLKYIHSA DVIHRDL--- ------KP-G NLLVNADCEL K--------- ---------- ---ICDFGLA RGFE------ SDPLRAGLAG SAGFMTEYVA TRWYRAPEIM LSFANYSTS- ---------- ----------
     52469  ---------T LCGLKYIHSA DVIHRDL--- ------KP-G NLL------- ---------- ---------- ---ICDFGLA RGFE------ SDPLRAGLAG SAGFMTEYVA TRWYRAPEIM LSFANYSTS- ---------- ----------
     30002  ---------T LCGLKYIHSA DVIHRDL--- ------KP-G NLLVNADCEL K--------- ---------- ---ICDFGLA RGFE------ SDPLRAGLAG SAGFMTEYVA TRWYRAPEIM LSFANYSTS- ---------- ----------
    154156  ---------A CCGLKYIHSA NVLHRDL--- ------KP-C NLLVNTKCEL K--------- ---------- ---ICDFGLA RGYV------ --DAKGGQEG DVGFMTEYVA TRYYRAPEIM LSFRSYTKA- ---------- ----------
    166841  ---------A CCGLKYIHSA NVLHRDL--- ------KP-C NLLVNTKCEL K--------- ---------- ---ICDFGLA RGYV------ --DAKGGQEG DVGFMTEYVA TRYYRAPEIM LSFR------ ---------- ----------
    238118  ---------A CCGLKYIHSA NVLHRDL--- ------KP-S NLLVNTRCEL K--------- ---------- ---ICDFGLA RGYI------ --DPSSGQT- DAGFMTEYVA TRWYRAPEIM LGFKSYTKA- ---------- ----------
    143069  ---------A CCGLKYIHSA NVLHRDL--- ------KP-S NLLVNTRCEL K--------- ---------- ---ICDFGLA RGYI------ --DPSSGQT- DAGFMTEYVA TRWYRAPEIM LGFKSYTKA- ---------- ----------
    456995  ---------T LCGLKYIHSA NVLHRDL--- ------KP-G NLLVNADCEL K--------- ---------- ---ICDFGLA RGFTP---GS GA---SKSAG NQGFMTEYVA TRWYRAPEIM LSFANYTTA- ---------- ----------
    175880  ---------T LCGLKYIHSA NVLHRDL--- ------KP-G NLLVNADCEL K--------- ---------- ---ICDFGLA RGYTP---AG GTAPPSKAAG NQGFMTEYVA TRWYRAPEIM LSFANYTTA- ---------- ----------
     28309  ---------I LCALKYIHSA NVLHCDL--- ------KP-K NLLVNSDCQL K--------- ---------- ---ICNFGLS CSYS------ -----ENHKV NDGFIKGYIT SIWYKAPEIL LNYQECTKA- ---------- ----------
    767958  ---------I LCALKYIHSA NVLHCDL--- ------KP-K NLLVNSDCQL K--------- ---------- ---ICNFGLS CSYS------ -----ENHKV NDGFIKGYIT SIWYKAPEIL LNYQECTKA- ---------- ----------
    126152  ---------I LCALKYIHSA NVLHCDL--- ------KP-K NLLVNSDCQL K--------- ---------- ---ICNFGLS CSYS------ -----ENHKV NDGFIKGYIT SIWYKAPEIL LNYQECTKA- ---------- ----------
    613191  ---------I LCGLKYIHSA NVLHRDL--- ------KP-G NLLVNADCEL K--------- ---------- ---ICDFGLA RGFS------ -----VDPEE NAGYMTEYVA TRWYRAPEIM LSFQSYTKA- ---------- ----------
    198058  ---------I LCGLKYIHSA NVLHRDL--- ------KP-G NLLVNADCEL K--------- ---------- ---ICDFGLA RGFS------ -----VDPEE NAGYMTEYVA TRWYRAPEIM LSFQSYTKA- ---------- ----------
    123006  ---------I LCGLKYIHSA NVLHRDL--- ------KP-G NLLVNADCEL K--------- ---------- ---ICDFGLA RGFS------ -----IDPEE NAGYMTEYVA TRWYRAPEIM LSFQNYTKA- ---------- ----------
    178993  ---------I LCGLKYIHSA NVLHRDL--- ------KP-G NLLVNADCEL K--------- ---------- ---ICDFGLA RGFS------ -----VDPEE NAGYMTEYVA TRWYRAPEIM LSFQNYTKA- ---------- ----------
    122967  ---------I LCGLKYIHSA NVLHRDL--- ------KP-G NLLVNADCEL K--------- ---------- ---ICDFGLA RGFS------ -----IDPEE NAGYMTEYVA TRWYRAPEIM LSFQNYTKA- ---------- ----------
    120501  ---------I LCGLKYIHSA NVLHRDL--- ------KP-G NLLVNADCEL K--------- ---------- ---ICDFGLA RGFS------ -----VDPEE NAGYMTEYVA TRWYRAPEIM LSFQNYTKA- ---------- ----------
     99018  ---------I LCGLKYIHSA NVLHRDL--- ------KP-G NLLVNADCEL K--------- ---------- ---ICDFGLA RGFS------ -----MDPEE NAGYMTEYVA TRWYRAPEIM LSFQSYTKA- ---------- ----------
    213678  ---------I LCGLKYIHSA NVLHRDL--- ------KP-G NLLVNADCEL K--------- ---------- ---ICDFGLA RGFS------ -----MDPEE NAGYMTEYVA TRWYRAPEIM LSFQSYTKA- ---------- ----------
    460523  ---------I LCGLKYIHSA NVLHRDL--- ------KP-G NLLVNADCEL K--------- ---------- ---ICDFGLA RGFS------ -----MDPEE NAGYMTEYVA TRWYRAPEIM LSFQSYTKA- ---------- ----------
    625621  ---------I LCGLKYIHSA NVLHRDL--- ------KP-G NLLVNADCEL K--------- ---------- ---ICDFGLA RGFS------ -----VDPEE NAGYMTEYVA TRWYRAPEIM LSFLSYTKA- ---------- ----------
    265871  ---------I LCGLKYIHSA NVLHRDL--- ------KP-G NLLVNADCEL K--------- ---------- ---ICDFGLA RGFS------ -----IDPEE NAGYMTEYVA TRWYRAPEIM LSFQSYTKA- ---------- ----------
     53464  ---------I LCGLKYIHSA NVLHRDL--- ------KP-G NLLVNADCEL K--------- ---------- ---ICDFGLA RGFS------ -----IDPEE NAGYMTEYVA TRWYRAPEIM LSFQSYTKA- ---------- ----------
    105414  ---------I LCGLKYIHSA NVLHRDL--- ------KP-G NLLVNADCEL K--------- ---------- ---ICDFGLA RGFS------ -----LDPEE NAGYMTEYVA TRWYRAPEIM LSFQSYTKA- ---------- ----------
    291876  ---------I LCGLKYIHSA NVLHRDL--- ------KP-G NLLVNADCEL K--------- ---------- ---ICDFGLA RGFS------ -----LDPEE NAGYMTEYVA TRWYRAPEIM LSFQSYTKA- ---------- ----------
     50744  ---------I LCGLKYIHSA DVLHRDL--- ------KP-G NLLVNVECDL K--------- ---------- ---ICDFGLA RGFSED---- -------VEQ NSGFMTEYVA TRWYRAPEIM LSFSNYTKA- ---------- ----------
    863837  ---------I CRGLKYIHSA NVLHRDL--- ------KP-G NLLVNADCEL K--------- ---------- ---ICDFGLA RGFSDD---- -------AES NVGFMTEYVA TRWYRAPEIM LSFQSYTKA- ---------- ----------
     81355  ---------T LCGLKYIHSA NVLHRDL--- ------KP-G NLLVNADCEL K--------- ---------- ---ICDFGLA RGYTPGGG-- --AS--RAAG NQGFMTEYVA TRWYRAPEIM LSFANYTTA- ---------- ----------
    152896  ---------T LCGLKYIHSA NVLHRDL--- ------KP-G NLLVNADCEL K--------- ---------- ---ICDFGLA RGYSPGGN-- --NNNTRGAA NQGFMTEYVA TRWYRAPEIM LSFANYGPA- ---------- ----------
   1196002  ---------T LCGLKYIHSA NVLHRDL--- ------KP-G NLLVNADCEL K--------- ---------- ---ICDFGLA RGYAPGPGGG TASANQKAAG NQGFMTEYVA TRWYRAPEIM LSFANYTTA- ---------- ----------
   1195972  ---------T LCGLKYIHSA NVLHRDL--- ------KP-G NLLVNADCEL K--------- ---------- ---ICDFGLA RGYAPGPGGG TASANQKAAG NQGFMTEYVA TRWYRAPEIM LSFANYTTA- ---------- ----------
     49917  ---------I LCAVKYIHSA NILHRDL--- ------KP-G NILVNSIGEL K--------- ---------- ---ICDFGLA RSYRDS-STR PRSSLTGGSD EEFKITHYVA TRWYRAPELM MNYENYSKA- ---------- ----------
    116823
    271167
    151286
     97820
    167647
    430394
    266211
    266223
    266189
    151328
    800193  ---------- KEVRGMNLNM ADMT----NR TIDAAINASA QAYRTTEAPI A--------- ---------- ----PSETLP PAPVPAPSQI AFPLVPEPET IPETEVHVEP PTSVGTTAVV TDNHPLVETT ---------- ----------
     22041  ---------- KEVRGMNLNM ADMT----NR TIDAAINASA QAYRTTEAPI A--------- ---------- ----PSETLP PAPVPAPSQI AFPLVPEPET IPETEVHVEP PTSVGTTAVV TDNHPLVETT ---------- ----------
     22014  ---------- KEVRGMNLNM ADMT----NR TIDAAINASA QAYRTTEAPI A--------- ---------- ----PSETLP PAPVPAPSQI AFPLVPEPET IPETEVHVEP PTSVGTTAVV TDNHPLVETT ---------- ----------
     22020  ---------- KEVRGMNLNM ADMT----NR TIDAAINASA QAYRTTEAPI A--------- ---------- ----PSETLP PAPVPAPSQI AFPLVPEPET IPETEVHVEP PTSVGTTAVV TDNHPLVETT ---------- ----------
    448385  ---------- KEVRGMNVEV -------DYL GCSAGESRKR SVERMEGREM P--------- ---------- ----DSPETE PGTVGATQTQ ATDSEAKPPT INEPLDGGAN LGALPPVPEI AR-------- ---------- ----------
    122008  ---------- REVRSVVPDI ---LIG-GGS QPASLERPHR PPPHSQSPAV S--------- ---------- ----PAMARS TLPEVPEESS SASALGSPAK ITENGDKGNG QDAVGEAVEA PV-------- --PF-PA--- KEPDAPEA--
    578295  ---------- HEVRNTNFEM ---EWP-KMM TANSVERPHV EQQERTSTPP G--------- ---------- ----IVVEDE SIEEA-ETST AVEAGGANKS DYAKEDEKQG TESDTVTVGA PVVEDSAPIS TIPL-PGLER RDSMRPPT--
    139503  ---------- REVRMIALEM MDDEPA-PLR SPSVLSAVDE GGDKAQVEVV D--------- ---------- ----EEMVNA TRLEGVDECT QEKVEGQGQS EAQQED---- TETIPPLAEF PSKETSMEKR RNSG-SSTQG SSSLAYPTQR
   2468658  ---------- REVRGMPRHD PLSGVP-LRR STSRTESSSR SVYEDAEEIL V--------- ---------- ----PEVVKE ---EVAVDET TAPPSPQPAV RTVSEDTESQ QTVTTEIVEE PSPL------ ---------- ----------
   2215386  ---------- REVRGMPRHD PLSGVP-LRR STSRTESSSR SVYEDAEEIL V--------- ---------- ----PEVVKE ---EVAVDET TAPPSPQPAV RTVSEDTESQ QTVTTEIVEE PSPL------ ---------- ----------
     88816  ---------- HEVRRAVFDF SVPSWSVPSK SANTSVIRTE QQPETSDEGL P--------- ---------- ----DIVVGG TSTDTTQTAV EEPPEKDDEP PKDEGKIGHE QEASEATIIA GSTLVHTPSE MPALRPGIER KDSMRPPT--
     88949  ---------- HEVRRAVFDF SVPSWSVPSK SANTSVIRTE QQPETSDEGL P--------- ---------- ----DIVVGG TSTDTTQTAV EEPPEKDDEP PKDEGKIGHE QEASEATIIA GSTLVHTPSE MPALRPGIER KDSMRPPT--
     88917  ---------- HEVRRAVFDF SVPSWSVPSK SANTSVIRTE QQPETSDEGL P--------- ---------- ----DIVVGG TSTDTTQTAV EEPPEKDDEP PKDEGKIGHE QEASEATIIA GSTLVHTPSE MPALRPGIER KDSMRPPT--
    206606  ---------- LSVRSGAGGA GEDEMA--EE LGSEVSGDGD DEAESVEIVV Q--------- ---------- ----DADMPV EGQLVLPETA LKPDLEQPTE ATTTAVPSDP TETVTDTVER LTLVDSSALV DDADGPASPL ATLPRTPL--
    123647  ---------- REMRSLAATS ---------- ---------- -PISESRFAI P--------- ---------- ----SREE-- VAVKPVSESP EKQEAAKQLL SPELPATRTR RPTVSRKPSV EPSV------ ---PEDSVA- ----------
    143931  ---------- REMRSLAATS ---------- ---------- -PISESRFAI P--------- ---------- ----SREE-- VAVKPVSESP EKQEAAKQLL SPELPATRTR RPTVSRKPSV EPSV------ ---PEDSVA- ----------
    165009  ---------- REVRSVTEDK ---------- ---------- -PPSPSASPT R--------- ---------- ----ELERGL EEIREMAESM NTSSDAVFLA SPELPHAQQL HESLQQAVGI ERTP------ ---SERQAA- ----------
    580315  ---------- KEVRSIYYDA GDSPDGSPIP AEEPPETS-P PRSEGLMSPF E--------- ---------- ----L--KPS EDASRAQSPE SADA--HDVV SPVSTTLATV AETETPATEF PFTT------ ------RPD- ----------
    492840  ---------- KEVRSIYYDA QESPDGSPIP DHQPPETSLA PAAEELMSPF E--------- ---------- ----L--KPS AEVPRVQSPE STEP--LDVV SPSSIGLATV TETEAPSTEF PSTT------ ------RPD- ----------
    594182  ---------- KEVRSIYFDV DEPA-DRSPS IRRPREVRSP PPIEEGESPT A--------- ---------- ----V-EPPT EPSGRLSPTE IKRMHIHDLT SPETSTVTAL PDSSTATATA TTSTLSTTEG STQPERSAE- ----------
    168238  ---------- KEVRSIYFDV DEPAADRSPS IRRPREVLSP PPIEEGESPP P--------- ---------- ----LPESPT AAGSSTLSSE AAEAGKSKPV SPETSTLPLP TESSTTSTIA APGT----EG AIPRERPAE- ----------
    107302  ---------- KEVRSIYFD- ---------- ---------- -------SPP P--------- ---------- ----LPESPT AAGSSTLSSE AAEAGKSKPV SPETSTLPLP TESSTTSTIA APGT----EG AIPRERPAE- ----------
     64718  ---------- REVRTFPFEL ---------- ---------- ----SKERDR T--------- ---------- ----RSPD-- ---RRDPSAP EETPTTQFPS IPEQSETSLS EAEESMVSDA ALPR------ ---------- ----------
     64830  ---------- REVRTFPFEL ---------- ---------- ----SKERDR T--------- ---------- ----RSPD-- ---RRDPSAP EETPTTQFPS IPEQSETSLS EAEESMVSDA ALPR------ ---------- ----------
   1154823  ---------- REVRTFPFEL ---------- ---------- ----SKERDR T--------- ---------- ----RSPD-- ---RRDPSAP EETPTTQFPS IPEQSETSLS EAEESMVSDA ALPR------ ---------- ----------
    433002  ---------- MDVRGIKPEA ---------- ASSPRVVEEG QLPVSPSRSP G--------- ---------- ----QSITSP ---TRETNSK SLDASLPRVN SPEQEREAV- -DTLVPPTTA SEFL------ ---------- ----------
    822334  ---------- MDVRGIKPEA ---------- ASSPRVVEEG QLPVSPSRSP G--------- ---------- ----QSITSP ---TRETNSK SLDASLPRVN SPEQEREAV- -DTLVPPTTA SEFL------ ---------- ----------
    511572  ---------- MEVRGITREP ---------- AA-------- --PGPHENAR G--------- ---------- ----ETSDSP ---EQFLESQ ESVSALRRMD SPEVIRENTV SDSLAPPATM -DYF------ ---------- ----------
    688421  ---------- LDVRGLKEEE ---------- ASLPPVFEES NQP-SVMGSP A--------- ---------- ----RSVDKE LIGRKDTSKM SDMGSLPLVG SPERIEEGT- --ETAVGAVA PRMG------ ---------- ----------
     17778  ---------- LDVRGLKEEE ---------- ASLPPVFEES NQP-SVMGSP A--------- ---------- ----RSVDKE LIGRKDTSKM SDMGSLPLVG SPERIEEGT- --ETAVGAVA PRMG------ ---------- ----------
   1336823  ---------- HEVRSLHTGS ---------L TPSPVKEAWN EEPHGNLELE A--------- ---------- ----LSPTTE FSENIETHNV DSIPPLTEEF EKIPSSEAKQ IPSSDTKQQS SNAN------ ---------- ----------
    605623  ---------- REVRSIALEE DEHP-----G PLRSPSYMGS ASHSPTYTRV S--------- ---------- ----PSQPAP SQLPPTPEAH ASYLTAQPST SSSVTPHGSN IVSFPTTAEH SPEKP----- ---------- ----------
    511468  ---------- MEVRGITRE- ---------- ---------- --PAAPGPHE N--------- ---------- ----ARGETS DSPEQFLESQ ESVSALRRMD SPEVIRENTV SDSLAPPATM DYFR------ ---------- ----------
     60300  ---------- REVR
   2435698  ---------- REVRGMPRRR KEEK----RR KEA
   1222436  ---------- MEGRGLRLKD LRSL----LL KNRE
   1172380  ---------- MEVRGISRDG VGSD----AQ PDPSRSTNAV GEEPSTQPRN P--------- ---------- ----EKAPLT PAPVLVADSI TEKLENLRVD SPEEMREEDK GDADENLAPP AIDFL----- ---------- ----------
   1151825  ---------- MEVRGI
    410058  ---------- KEVR
     57461  ---------- REVR
    311323  ---SLVRAPL QQQQQRYAEQ QQQQLQQQQQ QQQQGSGQVP -IPDQFSRQS E--------- ---------- ----DPRPEE MNGVQGGSLE QELGYGLDAG RQMRLAIFWV LYQACLDSSV NRVYAAFHLL IASSSTLTPS AAKTLTQLAT
    143102  ---ASHQTTL QPPVNTG-AG LRRKESLSAQ DRETLERTTA QARTATAPAA N--------- ---------- ----NGAS-- -GASHQRNES VGTAAAGGAG SIAASAVTSH AMRHMETIVM AQYFILPKLL YITLYAVLGS AIPYLPLFYD
   2750129  ---AEVRAQA RAAGQIQRSG SDSNLGIPSR DEIMNSPVAE NYNGQEVPRS Q--------- ---------- ----SPVMED PSAALEHASR FGQNYLAKFG WDASKGLGAQ GEGRTSAIKV SQKLDMMGIG AAHTKDPNGI AWKQSKDYEN
   2702932  ---AEVRAQA RAAGQIQRSG SDSNLGIPSR DEIMNSPVAE NYNGQEVPRS Q--------- ---------- ----SPVMED PSAALEHASR FGQNYLAKFG WDASKGLGAQ GEGRTSAIKV SQKLDMMGIG AAHTKDPNGI AWKQSKDYEN
    108010  DGCPDEWRVS NVVHVDHAAS SF-SNPVQLQ -IPQFIYGGP PMLAPGGGAE V--------- ---------- ----LPAASL NNSWFTNNGF TPRFPNEDSG FDLLSILARV VNRPNPRISL GPVDLTCSFV VADVRRHDNP IIFCSPTFCA
   1754091  AGSMGKGMAM LFAENGLDVS LYDANPDNID DLPSVLQRG- --LAPDLHRR V--------- ---------- ----AGYKDL TEFMHSLGGA DADKLLVFS- IPHGSAADKV IEQIRGFLTA GDVILDGGNE YYENAKRRQD ALRPHGISYL
    564944  --MSFRPPPI SYCVDTHCAG SWPECVVCMR RALSSEQSG- ---ASAFPSE V--------- ---------- ----ELHSPK RS--HHPEGD RGPTHAASKL PPIMFDVPFF RFEPSPLVLD YATFKPIVES FFHDASVPDS TLQARARHLL
   AtMPK18  ---------- PQLLKDYMSG SEGSN--FVY PSAIGHLRQQ FTYLEENSSR N--------- ---------- --------GP VIPLERK-HA SLPRSTVHST VVH---STSQ PNLGATDSRR VSFEPSKNGA SSAGHPSTSA YPTKSIGPPP
   AtMPK19  ---------- PQLLKDYMN- SEGSS--FLY PSAIGHLRKQ FAYLEENSGK S--------- ---------- --------GP VIPPDRK-HA SLPRSAVHSS AVN---SNAQ PSLNASDSRR VSIEPSRNGV V----PSTSA YSTKPLGPPP
   AtMPK20  ---------- PQLLKDHMNG ADKAS--FLY PSAVDQFRRQ FAHLEENSGK T--------- ---------- --------GP VAPLERK-HA SLPRSTV--- -IH---STAV ARGGQPKLMN NTNT------ LNPETTQNIP FNHATIQAQQ
 OsMPK20-1  ---------- PQLLKDYING TERTT--FLY PSAVDQFRKQ FAHLEENGGN ---------- ---------- --------GP VIPMDRK-HT SLPRSTI--- -VH---STPI PAKEQPRIGP SRDKPSDEPY SNPREFDRFS GNAPRTSQAP
 OsMPK20-4  ---------- PQLLKDYMNG TEKTN--FLY PSALDNFRRQ FANLEENGGK N--------- ---------- --------GD AVPSDRK-HV SLPRTTT--- -VH---SAPI PPKDHQNI-- ---------- ---------- -----TSQVP
 OsMPK20-5  ---------- PQLLKDYMNG SENTS--FLY PSAVDNFRRQ FAILEENGGK S--------- ---------- --------GA L---DRK-HV SLPRATT--- -VH---STSI PPNEGLDA-- ---------- ---------- -----TSQVT
 OsMPK20-3  ---------- PQLLKEHISG TERPN--FHH LSVVDQFRKQ FTQVEENLNG S--------- ---------- --------GA AVSLQRK-HS SLPRSTI--- -VH---SAAI PAKDYKHVAS SSTK------ ------LAVD GSWNAQIQGV
   AtMPK16  ---------- PKMLKEYLDG SEPTN--FMY PSAVEHFKKQ FAYLEEHYKN G--------- ---------- --------TS HNPPERQQHA SLPRACVLYS DNNHPVAQQS SAEVTDGLSK CSIRDERPRG ADRNAQMPMS RIPINVPQTI
 OsMPK16-1  ---------- PKMLREFLEG TESTG--FMY PSAVDHFKKQ FAYLEEHYAK G--------- ---------- --------ST AAPPERQ-HN SLPRPCVVYS DNR----PQS TASVTEDLSR CLIRDNNLKS QD-SASVGAS RIP-------
   AtMPK17  ---------- PQMLQEYLQG EENINSHFLY PSGVDQFKQE FARLEEHNDD E--------- ---------- ----E---EH NSPPHQRKYT SLPRERVCSS EDEGSD--SV HAQSSSASVV FTPPQTPNTA TGLSSQKASQ VDKAATPVKR
    AtMPK8  ---------- PQMLEEYLRG GDQLS--FMY PSGVDRFKRQ FAHLEENQGK P--------- ---------- ----GAAGGG RSTALHRHHA SLPRERVPAP NGETAEE-SS DVERRAAAAV ASTLESEEAD N--------- -GG----GYS
   AtMPK15  ---------- PQMLEEYLRG GNQLS--FMY PSGVDRFRRQ FAHLEENQGP ---------- ---------- --------GG RSNALQRQHA SLPRERVPAS KNETVEERSN DIERRTTAAV ASTLDSPKAS QQ-AEGTENG GGG----GYS
 OsMPK17-1  ---------- PQMLQEYMKG GEQIS--FLY PSGVDRFKRQ FAHLEENYSK ---------- ---------- --------GE RGSPLQRKHA SLPRERVGVS KDGYNQQNTN DQERSADSVA RTTVSPPMSQ DA-QQHGSAG QNGVTSTDLS
    AtMPK9  ---------- PQMLQEYLRG GEQTS--FMY PSGVDRFKRQ FAHLEENYGK ---------- ---------- --------GE KGSPLQRQHA SLPRERVPAP KKENGSH-NH DIENRSIASL VTTLESPPTS QH-E--GSDY RNGTSQTGYS
 OsMPK21-1  ---------- PHMLQEYLRG GDQMS--FMY PSGVDRFKRQ FAHLEEGVSK ---------- ---------- --------GE KSSPQLRQNA SLPRERAIGN KHGDD---EY HAKLNVGEKP CHASVTDGIS KP-L------ --------MS
 OsMPK21-2  ---------- PQMMQKYLRG GDQSN--FLY PSGVDRFKRQ FAHLEEGVAQ ---------- ---------- --------GD KTSPQLRQHV SLPRERVVRN GDEPDPTADY CIKLHVGEQP GHSSVTDGLN KP-L------ --------LS
 OsMPK17-2  ---------- PQMLQEYIRG GEQIS--FLY PSGVDRFKRQ FAHLEENYSR ---------- ---------- --------GE RSTPLRRQHA SLPRERVCSS VDSNNQDSDN EERRAISSIA RTMISPPRSQ EKGKNRASAY PNGIINLNSN
     30260  ---------- NDIHTRKQDI ATVTITRTTT ACV
    130090  --------LS QQQQQETIAE
    599215  ---------K EAWTWVSTQR PDKNAPVTAR PLQTPIPTAP
    219362  ---------- TERNKRLV
     56051  ---------- DTCKVGLDG
    139809  ---------- DTCKVGLDG
      1060  ---------- SDRINFGKKL
    184799  ---------- SDRINFGKKL
     68008  ---------- IDRLGRLNAS QNEGGLSNLL NAN
     99901  ---------- IDRLGRLNAS QNEGGLSNLL NAN
    104502  ---------- IDRLGRLNAS QNEGGLSNLL NAN
    645859  ---------- TDLSSSSL
    599559  ---------- TDLSSSSL
36518:4-24  ---------- AERKLAEM
    178963  ---------- QHVRTAPGGG AGGIPGQQPV QGPVPIPAGG QPQQWTAEDP R--------- ---------- ----PQEYGS MQGLEAELGG R
    196817  ---------- TSLSISPG
    738557  ---------- LFLML
   1159263
    176895
    664529  ---------- AEVRAHARAA GQIRRQDSLP IPSRDEILNS PVQEHGHVHG G--------- ---------- ----TSNYTA PNRRASSPIM DDPSEELERE LAGTHLAGKK
   1194920  ---------- SKKKNQLKA
    432994
     98579
```

```
     88826
     54157
    914084
    503422
     95205
    229298
    455126
    256867
    419576
     74409
    521809
     54281
     48349
    594110
     27538
     96623
    380926
    263236
    154671
     67562
    226444
   1137209
   AtMPK13
   AtMPK11
    459393
     69938
    591714
    107905
     99046
    863830
    287494
    662188
    492594
    176887
    618168
    492643
    125995
    122928
    154756
     68423
  OsMPK20-2
  Consensus           . lr.lk..hsa .v.hrdl         kp . nll.n.ncdl k                     .cdfgla r......... .......... ....mteyva trwyrapeim l....y...

             1051                                                                                                                                                    1200
    127228   -AIDVWSVGC ILAEMLSGKP LFPGRD---- ---------- ---------- ---------- ---------- ---------- ---------- ---------- ---------- -YHHQLTLIL DVLG------ ---------- -----TPTMD
    287090   -AIDVWSVGC ILAEMLSGKP LFPGRD---- ---------- ---------- ---------- ---------- ---------- ---------- ---------- ---------- -YHHQLTLIL DVLG------ ---------- -----TPTMD
    186494   -AIDVWSVGC ILAEMLSGKP LFPGRD---- ---------- ---------- ---------- ---------- ---------- ---------- ---------- ---------- -YHHQLTLIL DVLG------ ---------- -----TPTMD
    260272   -AIDVWSVGC ILAEMLSGKP LFPGRD---- ---------- ---------- ---------- ---------- ---------- ---------- ---------- ---------- -YHHQLTLIL DVLG------ ---------- -----TPTMD
    119975   -AIDVWSVGC ILAELMSGKP LFPGRD---- ---------- ---------- ---------- ---------- ---------- ---------- ---------- ---------- -YHHQLTLIL DVLG------ ---------- -----TPTMD
      2147   -AIDMWSVGC ILAEMLSGKP LFPGKD---- ---------- ---------- ---------- ---------- ---------- ---------- ---------- ---------- -YHHQLTLIL DVLG------ ---------- -----TPTME
    692376   -AIDVWSVGC ILAEMLSGKP LFPGKD---- ---------- ---------- ---------- ---------- ---------- ---------- ---------- ---------- -YHHQLTLIL DVLG------ ---------- -----TPTME
    698785   -AIDVWSVGC ILAEMLSGKP LFPGKD---- ---------- ---------- ---------- ---------- ---------- ---------- ---------- ---------- -YHHQLTLIL DVLG------ ---------- -----TPTME
    468116   -AIDVWSVGC ILAEMLSGKP LFPGKD---- ---------- ---------- ---------- ---------- ---------- ---------- ---------- ---------- -YHHQLTLIL DVLG------ ---------- -----TPTME
    274271   -AIDVWSVGC ILAEMLSGKP LFPGKD---- ---------- ---------- ---------- ---------- ---------- ---------- ---------- ---------- -YHHQLTLIL DVLG------ ---------- -----TPTME
    255486   -AIDVWSVGC ILAEMLSGKP LFPGKD---- ---------- ---------- ---------- ---------- ---------- ---------- ---------- ---------- -YHHQLTLIL DVLG------ ---------- -----TPTME
    285227   -AIDVWSVGC ILAEMLSGKP LFPGKD---- ---------- ---------- ---------- ---------- ---------- ---------- ---------- ---------- -YHHQLTLIL DVLG------ ---------- -----TPTME
     18186   -AIDVWSVGC ILAEMLSGKP LFPGKD---- ---------- ---------- ---------- ---------- ---------- ---------- ---------- ---------- -YHHQLTLIL DVLG------ ---------- -----TPTME
    465108   -AIDVWSVGC ILAEMLSGKP LFPGKD---- ---------- ---------- ---------- ---------- ---------- ---------- ---------- ---------- -YHHQLTLIL DVLG------ ---------- -----TPTME
   1164585   -AIDVWSVGC ILAEMLSGKP LFPGKD---- ---------- ---------- ---------- ---------- ---------- ---------- ---------- ---------- -YHHQLTLIL DVLG------ ---------- -----TPTME
     93851   -AIDVWSVGC ILAEMLSGKP LFPGKD---- ---------- ---------- ---------- ---------- ---------- ---------- ---------- ---------- -YHHQLTLIL DVLG------ ---------- -----TPTME
    108267   -AIDVWSVGC ILAEMLSGKP LFPGKD---- ---------- ---------- ---------- ---------- ---------- ---------- ---------- ---------- -YHHQLTLIL DVLG------ ---------- -----TPTME
     59022   -AIDVWSVGC ILAEMLSGKP LFPGKD---- ---------- ---------- ---------- ---------- ---------- ---------- ---------- ---------- -YHHQLTLIL DVLG------ ---------- -----TPTME
    370137   -AIDVWSVGC ILAEMLSGKP LFPGKD---- ---------- ---------- ---------- ---------- ---------- ---------- ---------- ---------- -YHHQLTLIL DVLG------ ---------- -----TPTME
    532216   -AIDVWSVGC ILAEMLSGKP LFPGKD---- ---------- ---------- ---------- ---------- ---------- ---------- ---------- ------C--- KYHHQLTLIL DVLG------ ---------- -----TPTME
     31240   -AIDVWSVGC ILAEMLSGKP LFPGKD---- ---------- ---------- ---------- ---------- ---------- ---------- ---------- ---------- -YHHQLTLIL DVLG------ ---------- -----TPTME
    160345   -AIDVWSVGC ILAEMLSGKP LFPGKD---- ---------- ---------- ---------- ---------- ---------- ---------- ---------- ---------- -YHHQLTLIL DVLG------ ---------- -----TPTME
    212060   -AIDVWSVGC ILAEMLSGKP LFPGKD---- ---------- ---------- ---------- ---------- ---------- ---------- ---------- ---------- -YHHQLTLIL DVLG------ ---------- -----TPTME
    327005   -AIDVWSVGC ILAEMLSGKP LFPGKD---- ---------- ---------- ---------- ---------- ---------- ---------- ---------- ---------- -YHHQLTLIL DVLG------ ---------- -----TPTME
     48412   -AIDVWSVGC ILAEMLSGKP LFPGKD---- ---------- ---------- ---------- ---------- ---------- ---------- ---------- ---------- -YHHQLTLIL DVLG------ ---------- -----TPTME
    606069   -AIDVWSVGC ILAEMLSGKP LFPGKD---- ---------- ---------- ---------- ---------- ---------- ---------- ---------- ---------- -YHHQLTLIL DVLG------ ---------- -----TPTME
    123724   -AIDVWSIGC ILAEMLSGKP LFPGKD---- ---------- ---------- ---------- ---------- ---------- ---------- ---------- ---------- -YHHQLTLIL DVLG------ ---------- -----TPTME
    342035   -AIDVWSVGC ILAEMLSGKP LFPGKD---- ---------- ---------- ---------- ---------- ---------- ---------- ---------- ---------- -YHHQLTLIL DVLG------ ---------- -----TPTME
   2135321   -AIDVWSVGC ILAEMLSGKP LFPGKD---- ---------- ---------- ---------- ---------- ---------- ---------- ---------- ---------- -YHHQLTLIL DVLG------ ---------- -----TPTME
    644393   -AIDVWSVGC ILAEMLSGKP LFPGKD---- ---------- ---------- ---------- ---------- ---------- ---------- ---------- ---------- -YHHQLTLIL DVLG------ ---------- -----TPTME
    784446   -AIDVWSVGC ILAEMLSGKP LFPGKD---- ---------- ---------- ---------- ---------- ---------- ---------- ---------- ---------- -YHHQLTLIL DVLG------ ---------- -----TPTME
    567834   -AIDVWSVGC ILAEMLSGKP LFPGKD---- ---------- ---------- ---------- ---------- ---------- ---------- ---------- ---------- -YHHQLTLIL DVLG------ ---------- -----TPTME
    389770   -AIDVWSVGC ILAEMLSGKP LFPGKD---- ---------- ---------- ---------- ---------- ---------- ---------- ---------- ---------- -YHHQLTLIL DVLG------ ---------- -----TPTME
     63860   -AIDVWSVGC ILAEMLSGKP LFPGKD---- ---------- ---------- ---------- ---------- ---------- ---------- ---------- ------CMLS SYHHQLTLIL DVLG------ ---------- -----TPTME
    131603   -AIDVWSVGC ILAEMLSGKP LFPGKD---- ---------- ---------- ---------- ---------- ---------- ---------- ---------- ---------- -YHHQLTLIL DVLG------ ---------- -----TPTME
    495859   -AIDVWSVGC ILAEMLSGKP LFPGKD---- ---------- ---------- ---------- ---------- ---------- ---------- ---------- ---------- -YHHQLTLIL DVLG------ ---------- -----TPTME
    434551   -AIDVWSVGC ILAEMLSGKP LFPGKD---- ---------- ---------- ---------- ---------- ---------- ---------- ---------- ---------- -YHHQLTLIL DVLG------ ---------- -----TPTME
    140828   -AIDVWSVGC ILAEMLSGKP LFPGKD---- ---------- ---------- ---------- ---------- ---------- ---------- ---------- ---------- -YHHQLTLIL DVLG------ ---------- -----TPTME
     66435   -AIDVWSVGC ILAEMLSGKP LFPGKD---- ---------- ---------- ---------- ---------- ---------- ---------- ---------- ---------- -YHHQLTLIL DVLG------ ---------- -----TPTME
    383913   -AIDVWSVGC ILAEMLSGKP LFPGKD---- ---------- ---------- ---------- ---------- ---------- ---------- ---------- ---------- -YHHQLTLIL DVLG------ ---------- -----TPTME
    323815   -AIDVWSVGC ILAEMLSGKP LFPGKD---- ---------- ---------- ---------- ---------- ---------- ---------- ---------- ---------- -YHHQLTLIL DVLG------ ---------- -----TPTME
    396599   -AIDVWSVGC ILAEMLSGKP LFPGKD---- ---------- ---------- ---------- ---------- ---------- ---------- ---------- ---------- -YHHQLTLIL DVLG------ ---------- -----TPTME
     74447   -AIDVWSVGC ILAEMLSGKP LFPGKD---- ---------- ---------- ---------- ---------- ---------- ---------- ---------- ---------- -YHHQLTLIL DVLG------ ---------- -----TPTME
     83363   -AIDVWSVGC ILAEMLSGKP LFPGKD---- ---------- ---------- ---------- ---------- ---------- ---------- ---------- ---------- -YHHQLTLIL DVLG------ ---------- -----TPTME
     50905   -AIDVWSVGC ILAEMLSGKP LFPGKD---- ---------- ---------- ---------- ---------- ---------- ---------- ---------- ---------- -YHHQLTLIL DVLG------ ---------- -----TPTME
    650405   -AIDVWSVGC ILAEMLSGKP LFPGKD---- ---------- ---------- ---------- ---------- ---------- ---------- ---------- ---------- -YHHQLTLIL DVLG------ ---------- -----TPTME
    493847   -AIDVWSVGC ILAEMLSGKP LFPGKD---- ---------- ---------- ---------- ---------- ---------- ---------- ---------- ---------- -YHHQLTLIL DVLG------ ---------- -----TPTME
     95409   -AIDVWSVGC ILAEMLTGKP LFPGKD---- ---------- ---------- ---------- ---------- ---------- ---------- ---------- ---------- -YHHQLTLIL DVLG------ ---------- -----TPTME
    835420   -AIDVWSVGC ILAEMLSGKP LFPGRD---- ---------- ---------- ---------- ---------- ---------- ---------- ---------- ---------- -YHSQLNLIL DVLG------ ---------- -----TPTME
    343609   -AIDVWSVGC ILAEMLSGKP LFPGRD---- ---------- ---------- ---------- ---------- ---------- ---------- ---------- ---------- -YHHQLTLIL DVLG------ ---------- -----TPTME
     67554   -AIDVWSVGC ILAEMLSGKP LFPGKD---- ---------- ---------- ---------- ---------- ---------- ---------- ---------- ---------- -YHHQLTLIL DVLG------ ---------- -----TPTME
    216015   -AIDVWSVGC ILAEMLSGRP LFPGRD---- ---------- ---------- ---------- ---------- ---------- ---------- ---------- ---------- -YHNQLWLIM EVLG------ ---------- -----TPNSE
     52193   -AIDVWSVGC ILAEMLSGRP LFPGRD---- ---------- ---------- ---------- ---------- ---------- ---------- ---------- ---------- -YHNQLWLIM EVLG------ ---------- -----TPNME
     53916   -AIDVWSVGC ILAEMLTGKP LFPGRD---- ---------- ---------- ---------- ---------- ---------- ---------- ---------- ---------- -YHNQLWLIM EVLG------ ---------- -----TPNME
     83931   -AIDVWSVGC ILAEMLSGKP LFPGRD---- ---------- ---------- ---------- ---------- ---------- ---------- ---------- ---------- -YHNQLWLII EILG------ ---------- -----TPSME
     51986   -VIDIWSVGC ILAEMLSGKP LFPGKD---- ---------- ---------- ---------- ---------- ---------- ---------- ---------- ---------- -YHHQLTLIL NVLG------ ---------- -----TPSME
     49589   -AIDLWSVGC ILAEMIAGRP LFPGRD---- ---------- ---------- ---------- ---------- ---------- ---------- ---------- ---------- -YHHQLTLIL DILG------ ---------- -----TPVME
    862988   -AIDVWSVGC ILAEMLSGKP LFPGRD---- ---------- ---------- ---------- ---------- ---------- ---------- ---------- ---------- -YHHQLTLIL DVLG------ ---------- -----TPTLD
    833500   -AIDVWSVGC ILAEMLSGKP LFPGRD---- ---------- ---------- ---------- ---------- ---------- ---------- ---------- ---------- -YHHQLTLIL DVLG------ ---------- -----TPTLD
    810703   -AIDVWSVGC ILAEMLSGKP LFPGRD---- ---------- ---------- ---------- ---------- ---------- ---------- ---------- ---------- -YHHQLTLIL DVLG------ ---------- -----TPTLD
    457677   -AIDVWSVGC ILAEMLSGKP LFPGRD---- ---------- ---------- ---------- ---------- ---------- ---------- ---------- ---------- -YHHQLTLIL DVLG------ ---------- -----TPTLD
    161161   -AIDIWSVGC ILAEMLSGKP LFPGRD---- ---------- ---------- ---------- ---------- ---------- ---------- ---------- ---------- -YHHQLTLIL DVLG------ ---------- -----TPTLD
   1588194   -AIDIWSVGC ILAEMLSGKP LFPGRD---- ---------- ---------- ---------- ---------- ---------- ---------- ---------- ---------- -YHHQLTLIL DVLG------ ---------- -----TPTLD
     98485   -AIDIWSVGC ILAEMLSGKP LFPGRD---- ---------- ---------- ---------- ---------- ---------- ---------- ---------- ---------- -YHHQLTLIL DVLG------ ---------- -----TPTLD
   1443964   -AIDVWSVGC ILAEMLSGKP LFPGRD---- ---------- ---------- ---------- ---------- ---------- ---------- ---------- ---------- -YHHQLTLIL DVLG------ ---------- -----TPTLD
    864221   -AIDVWSVGC ILAEMLSGKP LFPGRD---- ---------- ---------- ---------- ---------- ---------- ---------- ---------- ---------- -YHHQLTLIL DVLG------ ---------- -----TPTLD
    903905   -AIDVWSVGC ILAEMLSGKP LFPGRD---- ---------- ---------- ---------- ---------- ---------- ---------- ---------- ---------- -YHHQLTLIL DVLG------ ---------- -----TPTLD
   1147795   -AIDVWSVGC ILAEMLSGKP LFPGRD---- ---------- ---------- ---------- ---------- ---------- ---------- ---------- ---------- -YHHQLTLIL DVLG------ ---------- -----TPSLD
    622735   -AIDVWSVGC ILAEMLSGKP LFPGRD---- ---------- ---------- ---------- ---------- ---------- ---------- ---------- ---------- -YHHQLTLIL DVLG------ ---------- -----TPTLD
    166786   -AIDVWSVGC ILAEMLSGKP LFPGRD---- ---------- ---------- ---------- ---------- ---------- ---------- ---------- ---------- -YHHQLTLIL DVLG------ ---------- -----TPTLD
   2641010   -AIDIWSVGC ILAEMLSGKP LFPGRD---- ---------- ---------- ---------- ---------- ---------- ---------- ---------- ---------- -YHHQLTLIL DVLG------ ---------- -----TPTLD
     28679   -AIDVWSVGC ILAEMLSGKP LFPGRD---- ---------- ---------- ---------- ---------- ---------- ---------- ---------- ---------- -YHHQLSLIL DVLG------ ---------- -----TPTLD
    101027   -AIDVWSVGC ILAEMLSSKP LFPGRD---- ---------- ---------- ---------- ---------- ---------- ---------- ---------- ---------- -YHHQLSLIL DVLG------ ---------- -----TPTLD
    564549   -AIDVWSVGC ILAEMLSGKP LFPGKD---- ---------- ---------- ---------- ---------- ---------- ---------- ---------- ---------- -YHHQLTLIL DVLG------ ---------- -----TPTLD
    227465   -AIDIWSVGC ILAEMLSGKP LFPGRD---- ---------- ---------- ---------- ---------- ---------- ---------- ---------- ---------- -YHHQLTLIL DVLG------ ---------- -----TPTLD
     18095   XAIDVWSVGC ILAEMLSGKP LFPGRD---- ---------- ---------- ---------- ---------- ---------- ---------- ---------- ---------- -YHHQLSLIL DVLG------ ---------- -----TPTLD
    338545   -AIDVWSVGC ILAEMLSGKP LFPGRD---- ---------- ---------- ---------- ---------- ---------- ---------- ---------- ---------- -YHHQLTLIL DVLG------ ---------- -----TPSLD
    134728   -AIDVWSVGC ILAEMLSGKP LFPGRD---- ---------- ---------- ---------- ---------- ---------- ---------- ---------- ---------- -YHHQLTLIL DVLG------ ---------- -----TPTLD
    275037   -AIDIWSVGC ILAEMLSGKP LFPGRD---- ---------- ---------- ---------- ---------- ---------- ---------- ---------- ---------- -YHHQLTLIL DVLG------ ---------- -----TPTLD
    503339   -AIDVWSVGC ILAEMLSGKP LFPGRD---- ---------- ---------- ---------- ---------- ---------- ---------- ---------- ---------- -YHHQLTLIL DVLG------ ---------- -----TPTLD
     40745   -AIDVWSVGC ILAEMLSGRP LFPGRD---- ---------- ---------- ---------- ---------- ---------- ---------- ---------- ---------- -YHHQLTLIL DVLG------ ---------- -----TPTLD
     43876   -AIDIWSVGC ICAEMLSGRP LFPGRD---- ---------- ---------- ---------- ---------- ---------- ---------- ---------- ---------- -YHHQLTLIL SVTG------ ---------- -----TPTID
     14716   -AIDIWSVGC ICAEMLSGRP LFPGRD---- ---------- ---------- ---------- ---------- ---------- ---------- ---------- ---------- -YHHQLTLIL SVTG------ ---------- -----TPTID
     15138   -AIDIWSVGC IAAEMLSGRP LFPGRD---- ---------- ---------- ---------- ---------- ---------- ---------- ---------- ---------- -YHNQLSLIL AVTG------ ---------- -----TPTLD
     67488   -AIDIWSVGC ILAEMLSGRP LFPGRD---- ---------- ---------- ---------- ---------- ---------- ---------- ---------- ---------- -YHHQLTLIL DILG------ ---------- -----TPTLD
     28680   -AIDVWSVGC ILAEMLSGKP LFPGRD---- ---------- ---------- ---------- ---------- ---------- ---------- ---------- ---------- -YHHQLSLIL DVLG------ ---------- -----TPTLD
    177514   -AIDIWAVGC VLAELLSGRP LFPGRD---- ---------- ---------- ---------- ---------- ---------- ---------- ---------- ---------- -YSHQLDLIL DIIGEYCPSL NVHRQKNNLF DLLLGTPTLD
     53880   -AIDIWAVGC VLAELLTGRP LFPGRD---- ---------- ---------- ---------- ---------- ---------- ---------- ---------- ---------- -YSHQLDLIL DVIG------ ---------- -----TPSLD
    778168   -AIDVWAVGC ILAELLTGRP LFPGRD---- ---------- ---------- ---------- ---------- ---------- ---------- ---------- ---------- -YGHQLDLIL DVIG------ ---------- -----TPTLD
```

```
     388025  -AVDVWAVGC ILAELLTGKP LFPGRD---- ---------- ---------- ---------- ---------- ---------- ---------- ---------- ---------- -YGHQLDLIL DVIG------ ---------- -----TPTLD
     581713  -AIDMWAVGC ILAELLTGKP LFPGRD---- ---------- ---------- ---------- ---------- ---------- ---------- ---------- ---------- -YGHQLDLIL DVIG------ ---------- -----TPTLD
     229317  -AVDMWAVGC ILAELLTTRP LFPGRD---- ---------- ---------- ---------- ---------- ---------- ---------- ---------- ---------- -YSHQLDLIL DVIG------ ---------- -----TPTLD
     829657  -AIDLWAVGC ILAEMLTGTP LFPGRD---- ---------- ---------- ---------- ---------- ---------- ---------- ---------- ---------- -YSHQLNLIL DIIG------ ---------- -----TPTLD
      70959  -AIDIWAVGC ILAELINARP LFPGRD---- ---------- ---------- ---------- ---------- ---------- ---------- ---------- ---------- -YGHQLDLIL DVIG------ ---------- -----TPTLE
      32857  QAIDIWAVGC ILAELLSGRP LFPGRD---- ---------- ---------- ---------- ---------- ---------- ---------- ---------- ---------- -YGHQLDLIL DVIG------ ---------- -----TPTLE
     561172  -AIDIWAVGC ILAELLNGRP LFPGRD---- ---------- ---------- ---------- ---------- ---------- ---------- ---------- ---------- -YGHQLDLIL DVIG------ ---------- -----TPTLE
     573944  -AIDIWAVGC ILAELLNGRP LFPGRD---- ---------- ---------- ---------- ---------- ---------- ---------- ---------- ---------- -YGHQLDLIL DVIG------ ---------- -----TPTLE
     740770  -AIDVWAVGC ILAEMLTGRP LFPGRD---- ---------- ---------- ---------- ---------- ---------- ---------- ---------- ---------- -YSHQLDLIL DIIG------ ---------- -----TPTLD
      16071  -AIDLWSVGC ILAELLNGKP LFPGRD---- ---------- ---------- ---------- ---------- ---------- ---------- ---------- ---------- -YSHQLDLIL DVIG------ ---------- -----TPTLE
    1126092  -AIDLWSVGC ILAELLNGKP LFPGRD---- ---------- ---------- ---------- ---------- ---------- ---------- ---------- ---------- -YSHQLDLIL DVIG------ ---------- -----TPTLE
     478842  -AIDVWSVGC ILGEMLTGRP MFPGRD---- ---------- ---------- ---------- ---------- ---------- ---------- ---------- ---------- -YGHQLDLIL DVLG------ ---------- -----TPPLD
      46649  -AIDMWAVGC ILAECLSGRP LFPGRD---- ---------- ---------- ---------- ---------- ---------- ---------- ---------- ---------- -YSHQLDLIL DVIG------ ---------- -----TPTIE
     601377  -AIDVWSVGC ILAEMLTGRP LFPGRD---- ---------- ---------- ---------- ---------- ---------- ---------- ---------- ---------- -YGHQLDLIL DVIG------ ---------- -----TPTLE
     866574  -AIDMWSVGC VLAEMLSGKP LFPGRDY--- ---------- ---------- ---------- ---------- ---------- ---------- ---------- ---------- --HHQLSLIL DTLG------ ---------- -----TPSID
     521401  -AIDMWSVGC VLAEMLSGKP LFPGRDY--- ---------- ---------- ---------- ---------- ---------- ---------- ---------- ---------- --HHQLSLIL DTLG------ ---------- -----TPSID
     850251  -AIDMWSVGC VLAEMLSGKP LFPGRDY--- ---------- ---------- ---------- ---------- ---------- ---------- ---------- ---------- --HHQLSLIL DTLG------ ---------- -----TPSID
     832111  -AIDMWSVGC VLAEMLSGTP MFPGRDY--- ---------- ---------- ---------- ---------- ---------- ---------- ---------- ---------- --HDQLSLIL DVLG------ ---------- -----TPSID
     832110  -AIDMWSVGC VLAEMLSGTP MFPGRDY--- ---------- ---------- ---------- ---------- ---------- ---------- ---------- ---------- --HDQLSLIL DVLG------ ---------- -----TPSID
     676666  -AIDMWSVGC VLAEMLSGKP LFPGRDY--- ---------- ---------- ---------- ---------- ---------- ---------- ---------- ---------- --HDQLSLIL DVLG------ ---------- -----TPSID
    1143376  -AIDMWSVGC VLAEMLSGKP LFPGRDY--- ---------- ---------- ---------- ---------- ---------- ---------- ---------- ---------- --HDQLSLIL DVLG------ ---------- -----TPSID
     457401  -AIDMWSVGC VLAEMLSGKP LFPGRDY--- ---------- ---------- ---------- ---------- ---------- ---------- ---------- ---------- --HDQLSIIL DVLG------ ---------- -----TPSID
     834255  -AIDMWSVGC VLAEMLSGKP LFPGRDY--- ---------- ---------- ---------- ---------- ---------- ---------- ---------- ---------- --HDQLSIIL DVLG------ ---------- -----TPSID
     133042  -AIDIWSVGC VLAEMLSGKP LFPGRDY--- ---------- ---------- ---------- ---------- ---------- ---------- ---------- ---------- --HHQLSIIL DVLG------ ---------- -----TPTLD
      30507  -AIDIWSVGC VLAEMLSGKP LFPGRDY--- ---------- ---------- ---------- ---------- ---------- ---------- ---------- ---------- --HHQLSIIL DVLG------ ---------- -----TPSID
     646041  -AIDIWSVGC VLAEMLSGKP LFPGRDY--- ---------- ---------- ---------- ---------- ---------- ---------- ---------- ---------- --HHQLSIIL DILG------ ---------- -----TPSLD
     570964  -AIDIWSVGC VLAEMLSGKP LFPGRDY--- ---------- ---------- ---------- ---------- ---------- ---------- ---------- ---------- --HHQLSIIL DILG------ ---------- -----TPSLD
     301734  -AIDIWSVGC VLAEMLSGKP LFPGRDY--- ---------- ---------- ---------- ---------- ---------- ---------- ---------- ---------- --HSQLSLIL DILG------ ---------- -----TPSLD
     154378  -AIDIWSVGC VLAEMISGKP LFPGRDY--- ---------- ---------- ---------- ---------- ---------- ---------- ---------- ---------- --HHQLSIIL DILG------ ---------- -----TPSLD
      90189  -AIDMWSVGC VLAEMLSGKP LFPGRDY--- ---------- ---------- ---------- ---------- ---------- ---------- ---------- ---------- --HHQLSIIL DILG------ ---------- -----TPSID
     448371  -AIDIWSVGC VLAEMLSGKA LFPGRDY--- ---------- ---------- ---------- ---------- ---------- ---------- ---------- ---------- --HHQLSLIL DILG------ ---------- -----TPSLD
     476779  -AIDIWSVGC VLAEMLSGKA LFPGRDY--- ---------- ---------- ---------- ---------- ---------- ---------- ---------- ---------- --HHQLSLIL DILG------ ---------- -----TPSLD
      68819  -AIDIWSVGC VLAEMLSGQP LFPGRDY--- ---------- ---------- ---------- ---------- ---------- ---------- ---------- ---------- --HHQLSLIL DVLG------ ---------- -----TPSID
    2671102  -AIDMWSVGC VLAEMLSGKP LFPGRDY--- ---------- ---------- ---------- ---------- ---------- ---------- ---------- ---------- --HHQLSIIL DILG------ ---------- -----TPSLD
     940143  -AIDIWSVGC ILAEMLSGKP LFPGRDY--- ---------- ---------- ---------- ---------- ---------- ---------- ---------- ---------- --HHQLSIIL DILG------ ---------- -----TPSLD
     585786  -AIDIWSVGC ILAEMLSGKP LFPGRDY--- ---------- ---------- ---------- ---------- ---------- ---------- ---------- ---------- --HHQLSLIL DILG------ ---------- -----TPSLD
     349085  -AIDIWSVGC ILAEMLSGKP LFPGRDY--- ---------- ---------- ---------- ---------- ---------- ---------- ---------- ---------- --HHQLSIIL DILG------ ---------- -----TPSLD
    1539717  -AIDIWSVGC VLAEMLSGKP LFPGRDY--- ---------- ---------- ---------- ---------- ---------- ---------- ---------- ---------- --HHQLSIIL DILG------ ---------- -----TPSLD
      24638  -AIDIWSIGC VLAEMLSGKP LFPGRDY--- ---------- ---------- ---------- ---------- ---------- ---------- ---------- ---------- --HDQLSLIL DILG------ ---------- -----TPSID
      75000  -AIDIWSIGC VLAEMLSGKP LFPGRDY--- ---------- ---------- ---------- ---------- ---------- ---------- ---------- ---------- --HDQLSLIL DILG------ ---------- -----TPSID
    1245527  -AIDIWSVGC VLAEMLSGKP LFPGRDCECA NFVRRAETNH DDASFILHSS LTDTQYVFLP VMSSLLVSNL DALPMSRPPA YLFNLSLLPL PTPHHRHPTP ALSYPHHLLA VDHHQLSLIL DILG------ ---------- -----TPTLD
     103018  -AIDVWSVGT VLAEMLSGRP LFPGRDY--- ---------- ---------- ---------- ---------- ---------- ---------- ---------- ---------- --HHQLTLIL DVLG------ ---------- -----TPSLD
      43883  -AIDVWSVGC ILAEMLNGKP LFPGRDY--- ---------- ---------- ---------- ---------- ---------- ---------- ---------- ---------- --HHQLTLIL GVLG------ ---------- -----TPSLD
      58913  -AIDVWSVGC ILAEMLNGKP LFPGRD---- ---------- ---------- ---------- ---------- ---------- ---------- ---------- ---------- -YHHQLTLIL DVLG------ ---------- -----TPSLD
      18326  -AIDLWSVGC ILAEMLSGKP LFPGRD---- ---------- ---------- ---------- ---------- ---------- ---------- ---------- ---------- -YHHQLSIIL DVLG------ ---------- -----TPSLD
     301059  -AIDLWSVGC ILAEMLSGKP LFPGRD---- ---------- ---------- ---------- ---------- ---------- ---------- ---------- ---------- -YHHQLSIIL DVLG------ ---------- -----TPSLD
      12410  -AIDLWSVGC ILAEMLSGKP LFPGRD---- ---------- ---------- ---------- ---------- ---------- ---------- ---------- ---------- -YHHQLSIIL DVLG------ ---------- -----TPSLD
      18327  -AIDLWSVGC ILAEMLSGKP LFPGRD---- ---------- ---------- ---------- ---------- ---------- ---------- ---------- ---------- -YHHQLSIIL DVLG------ ---------- -----TPSLD
     184754  -AIDIWSVGC ILAELFLGQP LFPGRD---- ---------- ---------- ---------- ---------- ---------- ---------- ---------- ---------- -YKHQLILIF EILG------ ---------- -----TPIGD
      99455  -AIDVWSVGC ILAELLMGNP LFPGKD---- ---------- ---------- ---------- ---------- ---------- ---------- ---------- ---------- -YRDQLIMIF KVLG------ ---------- -----TPTGE
     706256  -AMDVWSCGC ILAELFLRRP IFPGRD---- ---------- ---------- ---------- ---------- ---------- ---------- ---------- ---------- -YRHQLLLIF GIIG------ ---------- -----TPHSD
     653342  -AMDVWSCGC ILAELFLRRP IFPGRD---- ---------- ---------- ---------- ---------- ---------- ---------- ---------- ---------- -YRHQLLLIF GIIG------ ---------- -----TPHSD
     462249  ---------- --------KA LFPGRD---- ---------- ---------- ---------- ---------- ---------- ---------- ---------- ---------- -YHHQLTLIL DVLG------ ---------- -----TPTLD
     326973  -AIDVWSVGC ILAEMLSGKP LFPGKD---- ---------- ---------- ---------- ---------- ---------- ---------- ---------- ---------- -YHHQLTLIL DVLG------ ---------- -----TPTME
      77855  -AIDVWSVGC ILAEMLSGKP LFPGKD---- ---------- ---------- ---------- ---------- ---------- ---------- ---------- ---------- -YHHQLTLIL DVLG------ ---------- -----TPTME
     338563  -AIDVWSVGC ILAEMLSGKP LFPGRD---- ---------- ---------- ---------- ---------- ---------- ---------- ---------- ---------- -YHHQLTLIL DVLG------ ---------- -----TPSLD
     233520  -AIDVWSVGC ILAEMLSGKP LFPGRD---- ---------- ---------- ---------- ---------- ---------- ---------- ---------- ---------- -YHHQLTLIL DVLG------ ---------- -----TPTLD
      73610  -AIDVWSVGC ILAEMLSGKP LFPGRD---- ---------- ---------- ---------- ---------- ---------- ---------- ---------- ---------- -YHHQLSLIL DVLG------ ---------- -----TPTLD
     150924  -AIDVWSVGC ILAEMLSSKP LFPGRD---- ---------- ---------- ---------- ---------- ---------- ---------- ---------- ---------- -YHHQLSLIL DVLG------ ---------- -----TPTLD
     348809  -AIDVWSVGC ILAEMLSGKP LFPGRD---- ---------- ---------- ---------- ---------- ---------- ---------- ---------- ---------- -YHHQLTLIL DVLG------ ---------- -----TPTLD
     913771  -AIDVWSVGC ILAEMLSGKP LFPGRD---- ---------- ---------- ---------- ---------- ---------- ---------- ---------- ---------- -YHHQLTLIL DVLG------ ---------- -----TPTLD
     913918  -AIDVWSVGC ILAEMLSGKP LFPGRD---- ---------- ---------- ---------- ---------- ---------- ---------- ---------- ---------- -YHHQLTLIL DVLG------ ---------- -----TPTLD
     747578  -AMDIWSCGC ILAEMVSGKP LFPGRD---- ---------- ---------- ---------- ---------- ---------- ---------- ---------- ---------- -YHHQLWLIL EVLG------ ---------- -----TPSFE
     458977  -AMDIWSCGC ILAEMVSGKP LFPGRD---- ---------- ---------- ---------- ---------- ---------- ---------- ---------- ---------- -YHHQLWLIL EVLG------ ---------- -----TPSFE
      41459  -AIDIWSVGC ILAELLTGRP LFPGRD---- ---------- ---------- ---------- ---------- ---------- ---------- ---------- ---------- -YGHQLDLIL DVIG------ ---------- -----TPTLD
    1397463  -AIDIWAVGC ILAELLNGRP LFPGRD---- ---------- ---------- ---------- ---------- ---------- ---------- ---------- ---------- -YGHQLDLIL DVIG------ ---------- -----TPTLE
     149203  -AIDIWAVGC ILAELLNGRP LFPGRD---- ---------- ---------- ---------- ---------- ---------- ---------- ---------- ---------- -YGHQLDLIL DVIG------ ---------- -----TPTLE
     102078  -AVDLWSVGC MMAELYLGYP LFPGKD---- ---------- ---------- ---------- ---------- ---------- ---------- ---------- ---------- -YKHQLLLIF QYLG------ ---------- -----TPEGE
 36766:44-3  -EIDVWSVGC ILAEMLTYRP LFPGKD---- ---------- ---------- ---------- ---------- ---------- ---------- ---------- ---------- -YQNQLQLIL QVLG------ ---------- -----TPMGE
      54278  -AIDIWSVGC ILAEMFTYKP LFPGSD---- ---------- ---------- ---------- ---------- ---------- ---------- ---------- ---------- -YVHQLRLIF EIIG------ ---------- -----SPSEE
      29185  --IDIWSVGC IAAEMLSGRP LFPGRD---- ---------- ---------- ---------- ---------- ---------- ---------- ---------- ---------- -YHNQLSLIL AVTG------ ---------- -----TPTLD
     790328  -AIDMWSVGC VLAEMLSGKP LFPGRD---- ---------- ---------- ---------- ---------- ---------- ---------- ---------- ---------- -YHHQLSLIL DTLG------ ---------- -----TPSID
     765763  -AIDMWSVGC VLAEMLSGKP LFPGRD---- ---------- ---------- ---------- ---------- ---------- ---------- ---------- ---------- -YHHQLSLIL DTLG------ ---------- -----TPSID
    1491990  -AIDIWSVGC VLAEMLSGKP LFPGRD---- ---------- ---------- ---------- ---------- ---------- ---------- ---------- ---------- -YHHQLSIIL DILG------ ---------- -----TPSLD
      32998  -AIDIWSVGC ILAEMLSGKP LFPGRD---- ---------- ---------- ---------- ---------- ---------- ---------- ---------- ---------- -YHHQLTLIL DVLG------ ---------- -----TPTLD
    2601990  -AIDMWSVGC VLAEMLSGKP LFPGRD---- ---------- ---------- ---------- ---------- ---------- ---------- ---------- ---------- -YHHQLSIIL DILG------ ---------- -----TPSLD
     262460  -AIDVWSVGC ILAEMLSGKP LFPGKD---- ---------- ---------- ---------- ---------- ---------- ---------- ---------- ---------- -YHHQLTLIL DVLG------ ---------- -----TPTME
      11717  -AIDVWAVGC IFAEMLLREP LFPGND---- ---------- ---------- ---------- ---------- ---------- ---------- ---------- ---------- -YLHQLKLII KFLG------ ---------- -----TPKQE
     532299  -AIDVWAVGC IFAEMLLREP LFPGND---- ---------- ---------- ---------- ---------- ---------- ---------- ---------- ---------- -YLHQLKLII KFLG------ ---------- -----TPKQE
     551831  -AIDVWAVGC IFAEMLLREP LFPGND---- ---------- ---------- ---------- ---------- ---------- ---------- ---------- ---------- -YLHQLKLII KFLG------ ---------- -----TPKQE
     301484  -AIDVWAVGC IFAEMLLREP LFPGND---- ---------- ---------- ---------- ---------- ---------- ---------- ---------- ---------- -YLHQLKLII KFLG------ ---------- -----TPKQE
      42366  -AIDVWSIGC ILAELLGGKP IFKGRD---- ---------- ---------- ---------- ---------- ---------- ---------- ---------- ---------- -YVDQLNQIL HYLG------ ---------- -----TPSED
     476004  -EVDVWSMGC IFAEMMSRKP LFPGQD---- ---------- ---------- ---------- ---------- ---------- ---------- ---------- ---------- -YIDQLHLIM NALG------ ---------- -----APNDQ
     558476  -EVDVWSMGC IFAEMMSRKP LFPGQD---- ---------- ---------- ---------- ---------- ---------- ---------- ---------- ---------- -YIDQLHLIM NALG------ ---------- -----APNDQ
       9954  -AIDIWSTGC IFAELLGRTP LFPGDD---- ---------- ---------- ---------- ---------- ---------- ---------- ---------- ---------- -YIHQLQIIC DKIG------ ---------- -----TPCEE
      82136  -AIDIWSTGC IFAELLGRTP LFPGDD---- ---------- ---------- ---------- ---------- ---------- ---------- ---------- ---------- -YIHQLQIIC DKIG------ ---------- -----TPCEE
     312415  -AIDIWSTGC IFAELLGRTP LFPGDD---- ---------- ---------- ---------- ---------- ---------- ---------- ---------- ---------- -YIHQLQIIC DKIG------ ---------- -----TPCEE
     354114  -GVDLWAAGC IFAEMLGRKP LFPGET---- ---------- ---------- ---------- ---------- ---------- ---------- ---------- ---------- -YVHQLQLIM NVLG------ ---------- -----VPEEH
      34134  -AVDIWATGC IVAELMQGKP FFKGKE
      20005  -AVDVWAVGC IFAEMLRRRP FFTGRD---- ---------- ---------- ---------- ---------- ---------- ---------- ---------- ---------- -PSDQLHMII RVLG------ ---------- -----SPSEE
     518921  -AVDVWAIGC IFAEMLRRRP FFTGRD---- ---------- ---------- ---------- ---------- ---------- ---------- ---------- ---------- -PSDQLHMII RVLG------ ---------- -----SPTEE
     503206  -AVDVWAIGC IFAEMLRRRP FFTGRD---- ---------- ---------- ---------- ---------- ---------- ---------- ---------- ---------- -PSDQLHMII RVLG------ ---------- -----SPTEE
      10810  -AVDVWAVGC IFAEMLRRRP FFTGRD---- ---------- ---------- ---------- ---------- ---------- ---------- ---------- ---------- -PSDQLHMII RVLG------ ---------- -----SPSEE
     AtMPK1  -SIDVWSVGC IFAELLGRKP IFQGTE---- ---------- ---------- ---------- ---------- ---------- ---------- ---------- ---------- -CLNQLKLIV NILG------ ---------- -----SQREE
     AtMPK2  -SIDVWSVGC IFAELLGRKP VFPGTE---- ---------- ---------- ---------- ---------- ---------- ---------- ---------- ---------- -CLNQIKLII NILG------ ---------- -----SQREE
     OsMPK7  -SIDVWSVGC IFAELLGRKP IFPGTE---- ---------- ---------- ---------- ---------- ---------- ---------- ---------- ---------- -CLNQLKLIV NVLG------ ---------- -----TMSEA
    OsMPK14  -SIDVWSVGC IFAELLGRKP IFPGTE---- ---------- ---------- ---------- ---------- ---------- ---------- ---------- ---------- -CLNQLKLIV NVLG------ ---------- -----TMSES
    AtMPK14  -SIDVWSVGC IFAEILGRKP IFPGTE---- ---------- ---------- ---------- ---------- ---------- ---------- ---------- ---------- -CLNQLKLII NVVG------ ---------- -----SQQDW
     AtMPK7  -SIDVWSVGC IFAEILGRKP IFPGTE---- ---------- ---------- ---------- ---------- ---------- ---------- ---------- ---------- -CLNQLKLII NVVG------ ---------- -----SQQES
    AtMPK12  -AIDIWSVGC ILGEIMTGQP LFPGKD---- ---------- ---------- ---------- ---------- ---------- ---------- ---------- ---------- -YVHQLRLIT ELVG------ ---------- -----SPDNS
     AtMPK4  -AIDIWSVGC ILGETMTREP LFPGKD---- ---------- ---------- ---------- ---------- ---------- ---------- ---------- ---------- -YVHQLRLIT ELIG------ ---------- -----SPDDS
   OsMPK4-1  -AIDVWSVGC ILGEIVTRQP LFPGRD---- ---------- ---------- ---------- ---------- ---------- ---------- ---------- ---------- -YIQQLKLIT ELIG------ ---------- -----SPDDS
  OsMPK16-2  -AIDIWSVGC ILGEIVTREP LFPGKD---- ---------- ---------- ---------- ---------- ---------- ---------- ---------- ---------- -YVHQLRLIT ELIG------ ---------- -----SPDDS
     AtMPK5  -AIDVWSVGC IFAEIMTREP LFPGKD---- ---------- ---------- ---------- ---------- ---------- ---------- ---------- ---------- -YVHQLKLIT ELIG------ ---------- -----SPDGA
    AtMPK10  -AIDVWSVGC IFMEIMNREP LFPGKD---- ---------- ---------- ---------- ---------- ---------- ---------- ---------- ---------- -QVNQLRLLL ELIG------ ---------- -----TPSEE
     AtMPK6  -AIDVWSVGC IFMELMDRKP LFPGRD---- ---------- ---------- ---------- ---------- ---------- ---------- ---------- ---------- -HVHQLRLLM ELIG------ ---------- -----TPSEE
     OsMPK6  -AIDVWSVGC IFMELMDRKP LFPGRD---- ---------- ---------- ---------- ---------- ---------- ---------- ---------- ---------- -HVHQLRLLM ELIG------ ---------- -----TPNEA
     AtMPK3  -AIDVWSVGC IFMELMNRKP LFPGKD---- ---------- ---------- ---------- ---------- ---------- ---------- ---------- ---------- -HVHQMRLLT ELLG------ ---------- -----TPTES
     OsMPK3  -AIDVWSVGC IFMELINRQP LFPGRD---- ---------- ---------- ---------- ---------- ---------- ---------- ---------- ---------- -HMHQMRLIT EVIG------ ---------- -----TPTDD
     206269  -AVDIWSAGC IFAEMLEGNP LFPGKD---- ---------- ---------- ---------- ---------- ---------- ---------- ---------- ---------- -HVHQFSIIT ELLG------ ---------- -----TPPDD
     154265  -AVDIWSAGC IFAEMLEGKP LFPGKD---- ---------- ---------- ---------- ---------- ---------- ---------- ---------- ---------- -HVHQFSIIT ELLG------ ---------- -----TPNDD
     168032  -AVDIWSTGC IFAEMLEGKP LFPGKD---- ---------- ---------- ---------- ---------- ---------- ---------- ---------- ---------- -HVHQFSIIT ELLG------ ---------- -----TPPDD
     307550  -AVDIWSAGC IFAEMLEGKP LFPGKD---- ---------- ---------- ---------- ---------- ---------- ---------- ---------- ---------- -HVHQFTIIT ELLG------ ---------- -----TPPDD
     320251  -EVDIWSAGC IFAEMLEGKP LFPGKD---- ---------- ---------- ---------- ---------- ---------- ---------- ---------- ---------- -HVNQFSIIT ELLG------ ---------- -----TPPDD
     356517  -EVDIWSAGC IFAEMLEGKP LFPGKD---- ---------- ---------- ---------- ---------- ---------- ---------- ---------- ---------- -HVNQFSIIT ELLG------ ---------- -----TPPDD
     113434  -AVDIWSTGC IFAEMLEGKP LFPGKD---- ---------- ---------- ---------- ---------- ---------- ---------- ---------- ---------- -HVNQFSIIT ELLG------ ---------- -----TPPDD
      28887  -AVDIWSAGC IFAEMLEGRP LFPGKD---- ---------- ---------- ---------- ---------- ---------- ---------- ---------- ---------- -HVNQFSIIT ELLG------ ---------- -----TPPED
      16635  -EVDIWSAGC IFAEMLEGKP LFPGKD---- ---------- ---------- ---------- ---------- ---------- ---------- ---------- ---------- -HVNQFSIIT ELLG------ ---------- -----TPPDD
       6923  -AVDIWSTGC IFAEMLEGRP LFPGKD---- ---------- ---------- ---------- ---------- ---------- ---------- ---------- ---------- -HVHQFSIIT ELLG------ ---------- -----TPPDD
     807502  -AVDIWSTGC IFAEMLEGKP LFPGKD---- ---------- ---------- ---------- ---------- ---------- ---------- ---------- ---------- -HVNQFSIIT ELLG------ ---------- -----TPPDD
     129058  -AVDIWSTGC IFAEMLEGKP LFPGKD---- ---------- ---------- ---------- ---------- ---------- ---------- ---------- ---------- -HVNQFSIIT ELLG------ ---------- -----TPPDD
     359241  -AVDIWSTGC IFAEMLEGKP LFPGKD---- ---------- ---------- ---------- ---------- ---------- ---------- ---------- ---------- -HVNQFSIIT ELLG------ ---------- -----TPPDD
      53282  -AVDIWSTGC IFAEMLEGKP LFPGKD---- ---------- ---------- ---------- ---------- ---------- ---------- ---------- ---------- -HVNQFSIIT ELLG------ ---------- -----TPPDD
     117789  -AVDIWSAGC IFAEMLEGKP LFPGKD---- ---------- ---------- ---------- ---------- ---------- ---------- ---------- ---------- -HVNQFSIIT ELLG------ ---------- -----TPPDD
     170586  -AVDIWSTGC IFAEMLEGKP LFPGKD---- ---------- ---------- ---------- ---------- ---------- ---------- ---------- ---------- -HVNQFSIIT ELLG------ ---------- -----TPPDD
     162119  -AVDIWSAGC IFAEMLEGKP LFPGKD---- ---------- ---------- ---------- ---------- ---------- ---------- ---------- ---------- -HVHQFSIIT ELLG------ ---------- -----TPPED
     560244  -AVDIWSAGC IFAEMLEGKP LFPGKD---- ---------- ---------- ---------- ---------- ---------- ---------- ---------- ---------- -HVHQFSIIT ELLG------ ---------- -----TPPED
     499279  -AVDIWSAGC IFAEMLEGKP LFPGKD---- ---------- ---------- ---------- ---------- ---------- ---------- ---------- ---------- -HVHQFSIIT ELLG------ ---------- -----TPPED
     898207  -AVDIWSAGC IFAEMLEGKP LFPGKD---- ---------- ---------- ---------- ---------- ---------- ---------- ---------- ---------- -HVNQFSIIT ELLG------ ---------- -----TPPDD
     500359  -AVDIWSTGC IFAEMLEGKP LFPGKD---- ---------- ---------- ---------- ---------- ---------- ---------- ---------- ---------- -HVNQFQIIT ELLG------ ---------- -----TPPDD
     390416  -AVDIWSTGC IFAEMLEGKP LFPGKD---- ---------- ---------- ---------- ---------- ---------- ---------- ---------- ---------- -HVNQFSIIT ELLG------ ---------- -----TPPDD
      61586  -AVDIWSTGC IFAEMLEGKP LFPGKD---- ---------- ---------- ---------- ---------- ---------- ---------- ---------- ---------- -HVNQFSIIT ELLG------ ---------- -----TPPDD
      74488  -AVDIWSTGC IFAEMLEGKP LFPGKD---- ---------- ---------- ---------- ---------- ---------- ---------- ---------- ---------- -HVNQFSIIT ELLG------ ---------- -----TPGDD
```

```
 155373  -AVDIWSVGC IFAEMLEGKP LFPGKD---- ---------- ---------- ---------- ---------- ---------- ---------- ---------- ---------- -HVNQFSIIT ELLG------ ---------- -----TPPDD
  56185  -AVDIWSAGC IFAEMLEGRP LFPGKD---- ---------- ---------- ---------- ---------- ---------- ---------- ---------- ---------- -HVNQFSIIT ELLG------ ---------- -----TPPDE
  14909  -AVDIWSAGC IFAEMLEGRP LFPGKD---- ---------- ---------- ---------- ---------- ---------- ---------- ---------- ---------- -HVNQFSIIT ELLG------ ---------- -----TPPED
 176707  -AVDIWSTGC IFAEMLEGRP LFPGKD---- ---------- ---------- ---------- ---------- ---------- ---------- ---------- ---------- -HVHQFSIIT ELLG------ ---------- -----TPPDD
 176721  -AVDIWSTGC IFAEMLEGRP LFPGKD---- ---------- ---------- ---------- ---------- ---------- ---------- ---------- ---------- -HVHQFSIIT ELLG------ ---------- -----TPPDD
 240717  -AVDIWSTGC IFAEMLEGRP LFPGKD---- ---------- ---------- ---------- ---------- ---------- ---------- ---------- ---------- -HVHQFSIIT ELLG------ ---------- -----TPPDD
  56428  -EVDIWSAGC IFAEMLDGKP LFPGKD---- ---------- ---------- ---------- ---------- ---------- ---------- ---------- ---------- -HVNQFSIIT ELLG------ ---------- -----TPPDD
  97915  -EVDIWSAGC IFAEMLDGKP LFPGKD---- ---------- ---------- ---------- ---------- ---------- ---------- ---------- ---------- -HVNQFSIIT ELLG------ ---------- -----TPPDD
 707387  -EVDIWSAGC IFAEMLEGKP LFPGKD---- ---------- ---------- ---------- ---------- ---------- ---------- ---------- ---------- -HVNQFSIIT ELLG------ ---------- -----TPPDD
 504013  -EVDIWSAGC IFAEMLEGKP LFPGKD---- ---------- ---------- ---------- ---------- ---------- ---------- ---------- ---------- -HVNQFSIIT ELLG------ ---------- -----TPPDD
  37843  -EVDIWSAGC IFAEMLEGKP LFPGKD---- ---------- ---------- ---------- ---------- ---------- ---------- ---------- ---------- -HVNQFSIIT ELLG------ ---------- -----TPPDD
 507853  -EVDIWSAGC IFAEMLEGKP LFPGKD---- ---------- ---------- ---------- ---------- ---------- ---------- ---------- ---------- -HVNQFSIIT ELLG------ ---------- -----TPPDD
 338747  -EVDIWSAGC IFAEMLEGKP LFPGKD---- ---------- ---------- ---------- ---------- ---------- ---------- ---------- ---------- -HVNQFSIIT ELLG------ ---------- -----TPPPD
  49060  -EVDIWSAGC IFAEMLEGKP LFPGKD---- ---------- ---------- ---------- ---------- ---------- ---------- ---------- ---------- -HVNQFSIIT DLLG------ ---------- -----TPPDD
 147551  -EVDIWSAGC IFAEMLEGKP LFPGKD---- ---------- ---------- ---------- ---------- ---------- ---------- ---------- ---------- -HVNQFSIIT ELLG------ ---------- -----TPPDD
 286873  -EVDIWSAGC IFAEMLEGKP LFPGKD---- ---------- ---------- ---------- ---------- ---------- ---------- ---------- ---------- -HVNQFSIIT ELLG------ ---------- -----TPPDD
 193904  -EVDIWSVGC IFAEMLEGKP LFPGKD---- ---------- ---------- ---------- ---------- ---------- ---------- ---------- ---------- -HVHQFRIIT DLLG------ ---------- -----APPDD
 189288  -EVDIWSAGC IFAEMLEGKP LFPGKD---- ---------- ---------- ---------- ---------- ---------- ---------- ---------- ---------- -HVNQFSIIT ELLG------ ---------- -----TPPDD
 198339  -EVDIWSAGC IFAEMLEGKP LFPGKD---- ---------- ---------- ---------- ---------- ---------- ---------- ---------- ---------- -HVNQFSIIT ELLG------ ---------- -----TPPDD
2140409  -EVDIWSAGC IFAEMLEGKP LFPGKD---- ---------- ---------- ---------- ---------- ---------- ---------- ---------- ---------- -HVNQFSIIT ELLG------ ---------- -----TPPDD
 642177  -EVDIWSAGC IFAEMLEGKP LFPGKD---- ---------- ---------- ---------- ---------- ---------- ---------- ---------- ---------- -HVNQFSIIT ELLG------ ---------- -----TPPDD
 498471  -EVDIWSAGC IFAEMLEGKP LFPGKD---- ---------- ---------- ---------- ---------- ---------- ---------- ---------- ---------- -HVNQFSIIT ELLG------ ---------- -----TPPDD
 784216  -EVDIWSAGC IFAEMLEGKP LFPGKD---- ---------- ---------- ---------- ---------- ---------- ---------- ---------- ---------- -HVNQFSIIT ELLG------ ---------- -----TPPDD
 128268  -EVDIWSAGC IFAEMLEGKP LFPGKD---- ---------- ---------- ---------- ---------- ---------- ---------- ---------- ---------- -HVNQFSIIT ELLG------ ---------- -----TPPDD
 246689  -EVDIWSAGC IFAEMLEGKP LFPGKD---- ---------- ---------- ---------- ---------- ---------- ---------- ---------- ---------- -HVNQFSIIT ELLG------ ---------- -----TPPDD
1081594  -EVDIWSAGC IFAEMLEGKP LFPGKD---- ---------- ---------- ---------- ---------- ---------- ---------- ---------- ---------- -HVNQFSIIT ELLG------ ---------- -----TPPDD
 603877  -EVDIWSAGC IFAEMLEGKP LFPGKD---- ---------- ---------- ---------- ---------- ---------- ---------- ---------- ---------- -HVNQFSIIT ELLG------ ---------- -----TPPDD
 140829  -EVDIWSAGC IFAEMLEGKP LFPGKD---- ---------- ---------- ---------- ---------- ---------- ---------- ---------- ---------- -HVNQFSIIT ELLG------ ---------- -----TPPDD
 327107  -EVDIWSAGC IFAEMLEGKP LFPGKD---- ---------- ---------- ---------- ---------- ---------- ---------- ---------- ---------- -HVNQFSIIT ELLG------ ---------- -----TPPDD
 329694  -EVDIWSAGC IFAEMLEGKP LFPGKD---- ---------- ---------- ---------- ---------- ---------- ---------- ---------- ---------- -HVNQFSIIT ELLG------ ---------- -----TPPDD
 393439  -EVDIWSAGC IFAEMLEGKP LFPGKD---- ---------- ---------- ---------- ---------- ---------- ---------- ---------- ---------- -HVNQFSIIT ELLG------ ---------- -----TPPDD
  38942  -EVDIWSAGC IFAEMLEGKP LFPGKD---- ---------- ---------- ---------- ---------- ---------- ---------- ---------- ---------- -HVNQFSIIT ELLG------ ---------- -----TPPDD
  87588  -EVDIWSAGC IFAEMLEGKP LFPGKD---- ---------- ---------- ---------- ---------- ---------- ---------- ---------- ---------- -HVNQFSIIT ELLG------ ---------- -----TPPDD
 443298  -EVDIWSAGC IFAEMLEGKP LFPGKD---- ---------- ---------- ---------- ---------- ---------- ---------- ---------- ---------- -HVNQFSIIT ELLG------ ---------- -----TPPDD
  53587  -EVDIWSAGC IFAEMLEGKP LFPGKD---- ---------- ---------- ---------- ---------- ---------- ---------- ---------- ---------- -HVNQFSIIT ELLG------ ---------- -----TPPDD
  70072  -AVDIWSAGC IFAEMLEGKP LFPGKD---- ---------- ---------- ---------- ---------- ---------- ---------- ---------- ---------- -HVNQFSIIT ELLG------ ---------- -----TPPED
  13822  -EVDIWSAGC IFAEMLEGKP LFPGKD---- ---------- ---------- ---------- ---------- ---------- ---------- ---------- ---------- -HVNQFSIIT ELLG------ ---------- -----TPPDD
  25364  -AVDIWSAGC IFAEMLEGKP LFPGKD---- ---------- ---------- ---------- ---------- ---------- ---------- ---------- ---------- -HVNQFSIIT ELLG------ ---------- -----TPPDD
 116576  -EVDIWSAGC IFAEMLEGKP LFPGKD---- ---------- ---------- ---------- ---------- ---------- ---------- ---------- ---------- -HVNQFSIIT ELLG------ ---------- -----TPPDD
 103311  -EVDMWSVGC ILAEMIEGKP LFPGKD---- ---------- ---------- ---------- ---------- ---------- ---------- ---------- ---------- -HVHQFSIIT ELLG------ ---------- -----SPPPD
  26173  -EVDLWSVGC ILAEMIEGKP LFPGKD---- ---------- ---------- ---------- ---------- ---------- ---------- ---------- ---------- -HVHQFSIIT ELLG------ ---------- -----SPPPD
  55352  -EVDLWSVGC ILSEMIEGKP LFPGKD---- ---------- ---------- ---------- ---------- ---------- ---------- ---------- ---------- -HVHQFSIIT ELLG------ ---------- -----SPPQD
 125207  -EVDIWSAGC IFAEMIEGKP LFPGKD---- ---------- ---------- ---------- ---------- ---------- ---------- ---------- ---------- -HVHQFSIIT DLLG------ ---------- -----SPPKD
  67307  -EVDIWSAGC IFAEMIEGKP LFPGKD---- ---------- ---------- ---------- ---------- ---------- ---------- ---------- ---------- -HVHQFSIIT DLLG------ ---------- -----SPPKD
 185764  -EVDIWSAGC IFAEMIEGKP LFPGKD---- ---------- ---------- ---------- ---------- ---------- ---------- ---------- ---------- -HVHQFSIIT ELLG------ ---------- -----SPPED
  99366  -EVDIWSAGC IFAEMIEGKP LFPGKD---- ---------- ---------- ---------- ---------- ---------- ---------- ---------- ---------- -HVHQFSIIT ELLG------ ---------- -----TPPDD
  50111  -AVDIWSTGC IFAEMLEGKP LFPGKD---- ---------- ---------- ---------- ---------- ---------- ---------- ---------- ---------- -HVNQFSIIT ELLG------ ---------- -----TPPED
  87970  -AVDIWSAGC IFAEMLEGKP LFPGKD---- ---------- ---------- ---------- ---------- ---------- ---------- ---------- ---------- -HVNQFSIIT ELLG------ ---------- -----TPPDD
 107532  -AVDIWSAGC IFAEMLEGKP LFPGKD---- ---------- ---------- ---------- ---------- ---------- ---------- ---------- ---------- -HVNQFSIIT ELLG------ ---------- -----TPPDD
 604754  -AVDIWSTGC IFAEMLEGKP LFPGKD---- ---------- ---------- ---------- ---------- ---------- ---------- ---------- ---------- -HVNQFQIIT ELLG------ ---------- -----TPPDD
 633130  -AVDIWSTGC IFAEMLEGKP LFPGKD---- ---------- ---------- ---------- ---------- ---------- ---------- ---------- ---------- -HVNQFQIIT ELLG------ ---------- -----TPPDD
 181927  -EVDVWSAGC IFAEMLDGKP LFPGTD---- ---------- ---------- ---------- ---------- ---------- ---------- ---------- ---------- -HVNQFSIIT ELLG------ ---------- -----TPPDD
  69161  -AVDIWSVGC IFAEMIDGRP IFPGKD---- ---------- ---------- ---------- ---------- ---------- ---------- ---------- ---------- -HVHQLTVIT ELLG------ ---------- -----SPPED
  35549  -EVDIWSAGC IFAEMLEGKP LFPGKD---- ---------- ---------- ---------- ---------- ---------- ---------- ---------- ---------- -HVDQFSIIT ELLG------ ---------- -----TPPDD
 230139  -EVDIWSAGC IFAEMLEGKP LFPGKD---- ---------- ---------- ---------- ---------- ---------- ---------- ---------- ---------- -HVNQFSIIT ELLG------ ---------- -----TPPDD
 193325  -EVDIWSAGC IFAEMLEGKP LFPGKD---- ---------- ---------- ---------- ---------- ---------- ---------- ---------- ---------- -HVNQFSIIT ELLG------ ---------- -----TPPDD
 430049  -EVDIWSAGC IFAEMLEGKP LFPGKD---- ---------- ---------- ---------- ---------- ---------- ---------- ---------- ---------- -HVNQFSIIT ELLG------ ---------- -----TPPDD
 479652  -EVDIWSAGC IFAEMLEGKP LFPGKD---- ---------- ---------- ---------- ---------- ---------- ---------- ---------- ---------- -HVNQFSIIT ELLG------ ---------- -----TPPDD
 986652  -EVDIWSAGC IFAEMLEGKP LFPGKD---- ---------- ---------- ---------- ---------- ---------- ---------- ---------- ---------- -HVNQFSIIT ELLG------ ---------- -----TPPED
 295607  -EVDIWSAGC IFAEMLEGKP LFPGKD---- ---------- ---------- ---------- ---------- ---------- ---------- ---------- ---------- -HVNQFSIIT ELLG------ ---------- -----TPPDD
 114099  -EVDIWSAGC IFAEMLEGKP LFPGKD---- ---------- ---------- ---------- ---------- ---------- ---------- ---------- ---------- -HVNQFSIIT ELLG------ ---------- -----TPPDD
 265170  -AVDIWSAGC IFAEMLEGKP LFPGKD---- ---------- ---------- ---------- ---------- ---------- ---------- ---------- ---------- -HVHQFSIIT ELLG------ ---------- -----TPPDD
  41804  -EVDIWSAGC ILAEMLDGSP -----P---- ---------- ---------- ---------- ---------- ---------- ---------- ---------- ---------- -E-------- ---------- ---------- ---------D
 500846  -AVDIWSTGC IFAEMLEGKP LFPGKD---- ---------- ---------- ---------- ---------- ---------- ---------- ---------- ---------- -H-------- ---------- ---------- ---------D
 341622  -EVDIWSTGC IFAEMLKGKP LFPGKD---- ---------- ---------- ---------- ---------- ---------- ---------- ---------- ---------- -HVNQFSIIT KLLG------ ---------- -----TPPDD
1545705  -AVDIWSAGC IFAEMLEGKP LFPGKD---- ---------- ---------- ---------- ---------- ---------- ---------- ---------- ---------- -HVHQFSIIT ELLG------ ---------- -----TPPED
  15276  -AVDIWSAGC IFAEMLEGKP LFPGKD---- ---------- ---------- ---------- ---------- ---------- ---------- ---------- ---------- -HVHQFSIIT ELLG------ ---------- -----TPPED
 391543  -KVDIWSAGC IFAELLMGEP LFPGKN---- ---------- ---------- ---------- ---------- ---------- ---------- ---------- ---------- -HINQFCVIT DLLG------ ---------- -----SPPEE
 346451  -KVDIWSAGC IFAELLMGEP LFPGKN---- ---------- ---------- ---------- ---------- ---------- ---------- ---------- ---------- -HINQFCVIT DLLG------ ---------- -----SPPEE
 449365  -KVDIWSAGC IFAEMMTGRP LFLGKN---- ---------- ---------- ---------- ---------- ---------- ---------- ---------- ---------- -HIDQFCVIT QLLG------ ---------- -----SPPDN
  54661  -EVDIWSAGC IFAEMLEGKP LFPGKD---- ---------- ---------- ---------- ---------- ---------- ---------- ---------- ---------- -HVNQFSIIT ELLG------ ---------- -----TPPDD
 275309  -AVDMWSVGC ILAEMIIGKP LFPGKD---- ---------- ---------- ---------- ---------- ---------- ---------- ---------- ---------- -HVHQFKLIT EILG------ ---------- -----KPSEE
 389883  -AVDVWSVGC ILAEMLLGRV LLPGND---- ---------- ---------- ---------- ---------- ---------- ---------- ---------- ---------- -HVHQFTLIT EMLG------ ---------- -----KPPKE
  61624  -EVDIWSAGC IFAEMFTRKP LFPGTD---- ---------- ---------- ---------- ---------- ---------- ---------- ---------- ---------- -YINQFFLIT ELLG------ ---------- -----TPSED
 173235  -EVDIWSAGC IFAEMFTRKP LFPGTD---- ---------- ---------- ---------- ---------- ---------- ---------- ---------- ---------- -YINQFFLIT ELLG------ ---------- -----TPSED
  35612  -EVDIWSAGC IFAEMLEGKP LFPGKD---- ---------- ---------- ---------- ---------- ---------- ---------- ---------- ---------- -HVDQFSIIT ELLG------ ---------- -----TPPDD
  35597  -EVDIWSAGC IFAEMLEGKP LFPGKD---- ---------- ---------- ---------- ---------- ---------- ---------- ---------- ---------- -HVDQFSIIT ELLG------ ---------- -----TPPDD
 391476  -KVDMWSAGC ILAELIAGTP LFPGQS---- ---------- ---------- ---------- ---------- ---------- ---------- ---------- ---------- -HADQFSVIL NLLG------ ---------- -----SVPQS
 335269  -KVDMWSAGC ILAELIAGTP LFPGQS---- ---------- ---------- ---------- ---------- ---------- ---------- ---------- ---------- -HADQFSVIL NLLG------ ---------- -----SVPQS
 377162  -KVDMWSAGC ILAELIAGTP LFPGQS---- ---------- ---------- ---------- ---------- ---------- ---------- ---------- ---------- -HADQFSVIL NLLG------ ---------- -----SVPQS
 271149  -EVDIWSAGC IFAEMLEGKP LFPGKD---- ---------- ---------- ---------- ---------- ---------- ---------- ---------- ---------- -HVNQFSIIT ELLG------ ---------- -----TPPDD
  89784  -EVDIWSVGC IFAEMLEGKP LFPGKD---- ---------- ---------- ---------- ---------- ---------- ---------- ---------- ---------- -HVHQFRIIT DLLG------ ---------- -----APPDD
 114108  -EVDIWSAGC IFAEMLEGKP LFPGKD---- ---------- ---------- ---------- ---------- ---------- ---------- ---------- ---------- -HVNQFSIIT ELLG------ ---------- -----TPPDD
 173207  --IDVWSVGC IYGEILGRRV LFKGRD---- ---------- ---------- ---------- ---------- ---------- ---------- ---------- ---------- -YIDQLHKIL GILG------ ---------- -----LPESI
 127887  --IDMWSVGC IFAELLGRKV LFKGTD---- ---------- ---------- ---------- ---------- ---------- ---------- ---------- ---------- -YVDQLHKIV GILG------ ---------- -----LPEDT
  29226  --IDMWSVGC IFAELLGRKV LFKGND---- ---------- ---------- ---------- ---------- ---------- ---------- ---------- ---------- -YVDQLHKIV GILG------ ---------- -----LPEDT
 167765  --IDIWSVGC ILAELLGRKV LFRGTD---- ---------- ---------- ---------- ---------- ---------- ---------- ---------- ---------- -YVDQLHKIV GILG------ ---------- -----LPKDT
 150384  --IDIWSVGC ILAELLGRKV LFRGTD---- ---------- ---------- ---------- ---------- ---------- ---------- ---------- ---------- -YVDQLHKIV GILG------ ---------- -----LPKDT
  57923  --IDLWSVGC IFAELLGRKV LFKGTD---- ---------- ---------- ---------- ---------- ---------- ---------- ---------- ---------- -YVDQLHKIV GTLG------ ---------- -----LPKDT
  57928  --IDLWSVGC IFAELLGRKV LFKGTD---- ---------- ---------- ---------- ---------- ---------- ---------- ---------- ---------- -YVDQLHKIV GTLG------ ---------- -----LPKDT
 230955  --IDVWSLGC IFGEMLGRKV LFKGSD---- ---------- ---------- ---------- ---------- ---------- ---------- ---------- ---------- -YVDQLHKIV AILG------ ---------- -----LPQDT
 172033  --IDVWSLGC IFGEMLGRKV LFKGSD---- ---------- ---------- ---------- ---------- ---------- ---------- ---------- ---------- -YVDQLHKIV AILG------ ---------- -----LPQDT
  40306  --IDVWSVGC ILAELLGGKP FFKGHD---- ---------- ---------- ---------- ---------- ---------- ---------- ---------- ---------- -YVDQLNQIL RYLG------ ---------- -----TPS-S
 693957  --IDVWSVGC ILAELLGGKP FFKGRD---- ---------- ---------- ---------- ---------- ---------- ---------- ---------- ---------- -YVDQLNQIL HYLG------ ---------- -----TPN-E
 508525  --IDVWSVGC ILAELLGGKP FFKGRD---- ---------- ---------- ---------- ---------- ---------- ---------- ---------- ---------- -YVDQLNQIL HYLG------ ---------- -----TPN-E
 471145  --IDVWSVGC ILAELLGGKP FFKGRD---- ---------- ---------- ---------- ---------- ---------- ---------- ---------- ---------- -YVDQLNQIL HYLG------ ---------- -----TPN-E
 480536  --IDVWSVGC ILAELLGGKP FFKGRD---- ---------- ---------- ---------- ---------- ---------- ---------- ---------- ---------- -YVDQLNQIL HYLG------ ---------- -----TPN-E
 471745  --IDVWSVGC ILAELLGGKP FFKGRD---- ---------- ---------- ---------- ---------- ---------- ---------- ---------- ---------- -YVDQLNQIL HYLG------ ---------- -----TPN-E
 687176  --IDVWSVGC ILAELLGGKP FFKGRD---- ---------- ---------- ---------- ---------- ---------- ---------- ---------- ---------- -YVDQLNQIL HYLG------ ---------- -----TPN-E
  34863  --IDVWSVGC ILAELLGSKP FFKGRD---- ---------- ---------- ---------- ---------- ---------- ---------- ---------- ---------- -YVDQLNQIL HYLG------ ---------- -----TPS-E
  57153  --IDVWSVGC ILAELLGSKP FFKGRD---- ---------- ---------- ---------- ---------- ---------- ---------- ---------- ---------- -YVDQLNQIL HYLG------ ---------- -----TPS-E
 147961  --IDVWSVGC ILAELLGSKP FFKGRD---- ---------- ---------- ---------- ---------- ---------- ---------- ---------- ---------- -YVDQLNQIL HFLG------ ---------- -----TPS-E
 287354  --IDVWSVGC ILAELLGSKP FFKGRD---- ---------- ---------- ---------- ---------- ---------- ---------- ---------- ---------- -YVDQLNQIL HFLG------ ---------- -----TPS-E
 281614  --IDVWSVGC ILAELLGGKP FFKGRD---- ---------- ---------- ---------- ---------- ---------- ---------- ---------- ---------- -YVDQLNQIL RYLG------ ---------- -----TPS-E
  29684  --IDVWSVGC ILAELLGGRP FFKGRD---- ---------- ---------- ---------- ---------- ---------- ---------- ---------- ---------- -YVDQLNQIL HILG------ ---------- -----TPN-E
 144400  SSVDVWSVGC ILAELLGGRP FFKGRD---- ---------- ---------- ---------- ---------- ---------- ---------- ---------- ---------- -YVDQLNQIL HILG------ ---------- -----TPN-E
 136175  --IDVWSVGC ILAELLGGRP FFKGRD---- ---------- ---------- ---------- ---------- ---------- ---------- ---------- ---------- -YVDQLNQIL HILG------ ---------- -----TPN-E
 126818  --IDVWSVGC ILAELLGGRP FFKGRD---- ---------- ---------- ---------- ---------- ---------- ---------- ---------- ---------- -YVDQLNQIL HILG------ ---------- -----TPN-E
2138682  --IDVWSVGC ILAELLGGRP FFKGRD---- ---------- ---------- ---------- ---------- ---------- ---------- ---------- ---------- -YVDQLNQIL HILG------ ---------- -----TPN-E
  48096  --IDVWSVGC ILAELLGGRP FFKGRD---- ---------- ---------- ---------- ---------- ---------- ---------- ---------- ---------- -YVDQLNQIL HILG------ ---------- -----TPN-E
 179022  --IDVWSVGC ILAELLGGRP FFKGRD---- ---------- ---------- ---------- ---------- ---------- ---------- ---------- ---------- -YVDQLNQIL HILG------ ---------- -----TPN-E
 778216  --IDVWSVGC ILAELLGGRP FFKGRD---- ---------- ---------- ---------- ---------- ---------- ---------- ---------- ---------- -YVDQLNQIL HILG------ ---------- -----TPN-E
 665550  --IDVWSVGC ILAELLGGRP FFKGRD---- ---------- ---------- ---------- ---------- ---------- ---------- ---------- ---------- -YVDQLNQIL HILG------ ---------- -----TPN-E
 190738  --IDVWSVGC ILAELLGGRP FFKGRD---- ---------- ---------- ---------- ---------- ---------- ---------- ---------- ---------- -YVDQLNQIL HILG------ ---------- -----TPN-E
1107049  --IDVWSVGC ILAELLGGRP FFKGRD---- ---------- ---------- ---------- ---------- ---------- ---------- ---------- ---------- -YVDQLNQIL HILG------ ---------- -----TPN-E
 504269  --IDVWSVGC ILAELLGGRP FFKGRD---- ---------- ---------- ---------- ---------- ---------- ---------- ---------- ---------- -YVDQLNQIL HYLG------ ---------- -----TPN-E
 432134  --IDVWSVGC ILAELLGGRP FFKGRD---- ---------- ---------- ---------- ---------- ---------- ---------- ---------- ---------- -YVDQLNQIL HYLG------ ---------- -----TPN-E
  59068  --IDVWSVGC ILAELLGGRP FFKGRD---- ---------- ---------- ---------- ---------- ---------- ---------- ---------- ---------- -YVDQLNQIL HYLG------ ---------- -----TPS-E
 336185  --IDVWSVGC ILAELLGGRP FFKGRD---- ---------- ---------- ---------- ---------- ---------- ---------- ---------- ---------- -YVDQLNQIL HFLG------ ---------- -----TPN-E
 343155  --IDVWSVGC ILAELLGGRP FFKGRD---- ---------- ---------- ---------- ---------- ---------- ---------- ---------- ---------- -YVDQLNQIL HYLG------ ---------- -----TPN-E
 326648  --IDVWSVGC ILAELLGGRP FFKGRD---- ---------- ---------- ---------- ---------- ---------- ---------- ---------- ---------- -YVDQLNQIL HYLG------ ---------- -----TPN-E
 425863  --IDVWSVGC ILAELLGGRP FFKGRD---- ---------- ---------- ---------- ---------- ---------- ---------- ---------- ---------- -YVDQLNQIL HYLG------ ---------- -----TPN-E
 436998  --IDVWSVGC ILAELLGGRP FFKGRD---- ---------- ---------- ---------- ---------- ---------- ---------- ---------- ---------- -YVDQLNQIL HYLG------ ---------- -----TPA-E
 257317  --IDVWSVGC ILAELLGGRP FFKGRD---- ---------- ---------- ---------- ---------- ---------- ---------- ---------- ---------- -YVDQLNQIL HYLG------ ---------- -----TPN-E
 998070  --IDVWSVGC ILAELLGGKP FFKGRD---- ---------- ---------- ---------- ---------- ---------- ---------- ---------- ---------- -YVDQLNQIL HYLG------ ---------- -----TPN-E
  49449  --IDVWSVGC ILAELLGGKP FFKGRD---- ---------- ---------- ---------- ---------- ---------- ---------- ---------- ---------- -YVDQLNQIL HYLG------ ---------- -----TPN-S
 498377  --IDVWSVGC ILAELLGGKP FFKGRD---- ---------- ---------- ---------- ---------- ---------- ---------- ---------- ---------- -YVDQLNQIL HYLG------ ---------- -----TPN-E
 184715  --IDVWSVGC ILAELLGGRP FFKGRD---- ---------- ---------- ---------- ---------- ---------- ---------- ---------- ---------- -YVDQLNQIL HYLG------ ---------- -----TPN-E
  51223  --IDVWSVGC ILAELLGGRP FFKGRD---- ---------- ---------- ---------- ---------- ---------- ---------- ---------- ---------- -YVDQLNQIL HYLG------ ---------- -----TPN-E
  66669  --IDVWSVGC ILAELLGGRP FFKGRD---- ---------- ---------- ---------- ---------- ---------- ---------- ---------- ---------- -YVDQLNQIL HYLG------ ---------- -----TPN-E
 649898  --IDVWSVGC ILAELLGGRP FFKGRD---- ---------- ---------- ---------- ---------- ---------- ---------- ---------- ---------- -YVDQLNQIL HFLG------ ---------- -----TPN-E
 253817  --IDVWSVGC ILAELLGGRP FFKGRD---- ---------- ---------- ---------- ---------- ---------- ---------- ---------- ---------- -YVDQLNQIL HFLG------ ---------- -----TPN-E
 618068  --IDVWSVGC ILAELLGGRP FFKGRD---- ---------- ---------- ---------- ---------- ---------- ---------- ---------- ---------- -YVDQLNQIL HILG------ ---------- -----TPN-E
```

```
   108847  --IDIWSVGC ILGELLGGKP LFRGKD---- ---------- ---------- ---------- ---------- ---------- ---------- ---------- ---------- -YVDQLNQIL MVLG------ ---------- -----TPK-E
    33629  --IDIWSVGC ILAELLGGKP LFRGKD---- ---------- ---------- ---------- ---------- ---------- ---------- ---------- ---------- -YVDQLNQIL LVLG------ ---------- -----TPK-E
    54715  --IDIWSVGC ILAELLGGKP IFRGKD---- ---------- ---------- ---------- ---------- ---------- ---------- ---------- ---------- -YVDQLNQIL LILG------ ---------- -----TPP-E
    88920  --IDMWSVGC ILAELLGGKP FFKGRD---- ---------- ---------- ---------- ---------- ---------- ---------- ---------- ---------- -YVDQLNQIL HFLG------ ---------- -----TPD-E
    51479  --IDMWSVGC ILAELLGGKP LFKGKD---- ---------- ---------- ---------- ---------- ---------- ---------- ---------- ---------- -FVDQLNLIL NIVG------ ---------- -----TPN-E
    86943  --IDIWSVGC ILAELLGGKP IFRGKD---- ---------- ---------- ---------- ---------- ---------- ---------- ---------- ---------- -YVDQLNQIL YIVG------ ---------- -----TPK-E
   100835  --IDIWSVGC ILAELLGGKP IFKGKD---- ---------- ---------- ---------- ---------- ---------- ---------- ---------- ---------- -YVDQLNQIL YIVG------ ---------- -----TPP-E
   319719  --IDVWSAGC ILAEFLGGKP IFKGKD---- ---------- ---------- ---------- ---------- ---------- ---------- ---------- ---------- -YVNQLNQIL QVLG------ ---------- -----TPP-D
   822494  --IDVWSAGC ILAEFLGGKP IFKGKD---- ---------- ---------- ---------- ---------- ---------- ---------- ---------- ---------- -YVNQLNQIL QVLG------ ---------- -----TPP-D
   161919  --IDMWSVGC IFAEMLGGRP LFKGRD---- ---------- ---------- ---------- ---------- ---------- ---------- ---------- ---------- -YVDQLNQIL GILG------ ---------- -----TPD-E
   158631  --IDMWSVGC IFAEMLGGRP LFKGRD---- ---------- ---------- ---------- ---------- ---------- ---------- ---------- ---------- -YVDQLNQIL GILG------ ---------- -----TPD-E
   115223  --IDMWSVGC IFAEMLGGRP LFKGRD---- ---------- ---------- ---------- ---------- ---------- ---------- ---------- ---------- -YVDQLNQIL GILG------ ---------- -----TPD-E
   344665  --IDMWSVGC IFAEMLGGKP LFKGRD---- ---------- ---------- ---------- ---------- ---------- ---------- ---------- ---------- -YVDQLNQIL GILG------ ---------- -----TPD-E
   178050  --IDMWSVGC IFSEMLGGKP LFKGRD---- ---------- ---------- ---------- ---------- ---------- ---------- ---------- ---------- -YVDQLNQIL SILG------ ---------- -----TPD-E
   234927  --IDMWSVGC IFAEMLGGKP LFKGRD---- ---------- ---------- ---------- ---------- ---------- ---------- ---------- ---------- -YVDQLNQIL GILG------ ---------- -----TPD-E
   328916  --IDLWSVGC ILAELLGGRP LFKGRD---- ---------- ---------- ---------- ---------- ---------- ---------- ---------- ---------- -YVDQLNQIL QTLG------ ---------- -----TPS-D
   273327  --IDLWSVGC ILAELLGGRP LFKGRD---- ---------- ---------- ---------- ---------- ---------- ---------- ---------- ---------- -YVDQLNQIL QTLG------ ---------- -----TPS-D
   338804  --IDLWSVGC ILAELLGGRP LFKGRD---- ---------- ---------- ---------- ---------- ---------- ---------- ---------- ---------- -YVDQLNQIL QTLG------ ---------- -----TPS-D
   527701  --IDVWSIGC ILAELLGGKP IYKGRD---- ---------- ---------- ---------- ---------- ---------- ---------- ---------- ---------- -YVDQLNQIL HYLG------ ---------- -----TPP-E
    81317  --IDVWSVGC ILAELLGGKP IFKGRD---- ---------- ---------- ---------- ---------- ---------- ---------- ---------- ---------- -YVDQLNQIL HYLG------ ---------- -----TPS-E
   831329  --IDVWSVGC ILAELLGGKP IFKGRD---- ---------- ---------- ---------- ---------- ---------- ---------- ---------- ---------- -YVDQLNQIL HYLG------ ---------- -----TPS-E
   676624  --IDVWSVGC ILAELLGGKP IFKGRD---- ---------- ---------- ---------- ---------- ---------- ---------- ---------- ---------- -YVDQLNQIL HYLG------ ---------- -----TPS-E
   826941  --IDVWSIGC ILAELLGGKP IYKGRD---- ---------- ---------- ---------- ---------- ---------- ---------- ---------- ---------- -YVDQLNQIL HYLG------ ---------- -----TPS-E
    77377  --IDVWSVGC ILAELLGGKP IYKGRD---- ---------- ---------- ---------- ---------- ---------- ---------- ---------- ---------- -YVDQLNQIL HYLG------ ---------- -----TPS-E
    59915  --IDVWSIGC ILAELLGGKP IFKGRD---- ---------- ---------- ---------- ---------- ---------- ---------- ---------- ---------- -YVDQLNQIL HYLG------ ---------- -----TPS-E
   160641  --IDVWSVGC ILAELLAGKP IFKGRD---- ---------- ---------- ---------- ---------- ---------- ---------- ---------- ---------- -YVDQLNQIL HYLG------ ---------- -----TPS-E
   108839  --IDVWSVGC ILAELLSGKP IFKGRD---- ---------- ---------- ---------- ---------- ---------- ---------- ---------- ---------- -YVDQLNQIL HYLG------ ---------- -----TPS-E
  1536187  --IDVWSVGC ILAELLAGKP IFKGRD---- ---------- ---------- ---------- ---------- ---------- ---------- ---------- ---------- -YVDQLNQIL HYLG------ ---------- -----TPS-E
   281708  --IDVWSVGC ILAELLAGKP IFKGRD---- ---------- ---------- ---------- ---------- ---------- ---------- ---------- ---------- -YVDQLNQIL HYLG------ ---------- -----TPS-E
   173791  --IDVWSVGC ILAELLAGKP IFKGRD---- ---------- ---------- ---------- ---------- ---------- ---------- ---------- ---------- -YVDQLNQIL HYLG------ ---------- -----TPS-E
    82703  --IDVWSVGC ILAELLAGKP IFKGRD---- ---------- ---------- ---------- ---------- ---------- ---------- ---------- ---------- -YVDQLNQIL HYLG------ ---------- -----TPS-E
   139538  --IDVWSMGC ILAELLGGKP IFKGRD---- ---------- ---------- ---------- ---------- ---------- ---------- ---------- ---------- -YVDQLNQIL HYLG------ ---------- -----TPS-E
   479859  --IDVWSVGC ILAELLGGKP IFKGRD---- ---------- ---------- ---------- ---------- ---------- ---------- ---------- ---------- -YVDQLNQIL HYLG------ ---------- -----TPS-E
   621303  --IDIWSVGC ILAELLGGKP IFKGRD---- ---------- ---------- ---------- ---------- ---------- ---------- ---------- ---------- -YVDQLNQIL HYLG------ ---------- -----TPS-E
    38569  --IDIWSVGC ILAELLGGKP IFKGRD---- ---------- ---------- ---------- ---------- ---------- ---------- ---------- ---------- -YVDQLNQIL HYLG------ ---------- -----TPS-E
   242842  --IDVWSVGC ILAELLGGKP IFKGRD---- ---------- ---------- ---------- ---------- ---------- ---------- ---------- ---------- -YVDQLNQIL HYLG------ ---------- -----TPS-E
  2481011  --IDVWSIGC ILAELLGGKP IFKGRD---- ---------- ---------- ---------- ---------- ---------- ---------- ---------- ---------- -YVDQLNQIL HYLG------ ---------- -----TPS-E
   169913  --IDVWSVGC ILAELLAGKP IFKGRD---- ---------- ---------- ---------- ---------- ---------- ---------- ---------- ---------- -YVDQLNQIL HYLG------ ---------- -----TPS-E
    71742  --IDMWSVGC ILAELLGGKP IYKGKD---- ---------- ---------- ---------- ---------- ---------- ---------- ---------- ---------- -YVDQLNQIL HYLG------ ---------- -----TPS-E
    66635  --IDMWSVGC ILAELLGGRP IFKGKD---- ---------- ---------- ---------- ---------- ---------- ---------- ---------- ---------- -YVDQLNQII HYLG------ ---------- -----TPS-D
    53870  --IDMWSVGC ILAELLGGRP IFKGKD---- ---------- ---------- ---------- ---------- ---------- ---------- ---------- ---------- -YVDQLNQII HYLG------ ---------- -----TPS-D
    27246  --IDVWSVGC ILAELLGGKP IFKGRD---- ---------- ---------- ---------- ---------- ---------- ---------- ---------- ---------- -YVDQLNQIL HYLG------ ---------- -----TPS-E
    88978  --IDMWSVGC ILAELLGGKP IYKGKD---- ---------- ---------- ---------- ---------- ---------- ---------- ---------- ---------- -YVDQLNQIL HYLG------ ---------- -----TPS-E
  1091096  --IDVWSVGC ILAELLGGKP IYKGRD---- ---------- ---------- ---------- ---------- ---------- ---------- ---------- ---------- -YVDQLNQIL HYLG------ ---------- -----TPS-E
    66856  --IDVWSVGC ILAELLAGKP IFKGRD---- ---------- ---------- ---------- ---------- ---------- ---------- ---------- ---------- -YVDQLNQIL HYLG------ ---------- -----TPS-E
    20720  --IDVWSVGC ILAELLAGKP IFKGRD---- ---------- ---------- ---------- ---------- ---------- ---------- ---------- ---------- -YVDQLNQIL HYLG------ ---------- -----TPS-E
   124539  --IDVWSIGC ILAELLGGKP IFKGRD---- ---------- ---------- ---------- ---------- ---------- ---------- ---------- ---------- -YVDQLNQIL HYLG------ ---------- -----TPS-E
   361187  --IDVWSVGC ILAELLGGRP FFKGRD---- ---------- ---------- ---------- ---------- ---------- ---------- ---------- ---------- -YVDQLNQIL HILG------ ---------- -----TPN-E
   327531  --IDIWSVGC ILAECLGGKP IFRGRD---- ---------- ---------- ---------- ---------- ---------- ---------- ---------- ---------- -YVDQLSQIL NCLG------ ---------- -----TPK-E
     7086  --IDIWSVGC ILAECLGGKP IFRGRD---- ---------- ---------- ---------- ---------- ---------- ---------- ---------- ---------- -YVDQLSQIL NCLG------ ---------- -----TPK-E
    28915  --IDMWSVGC IFAEMLGGKP LFKGRD---- ---------- ---------- ---------- ---------- ---------- ---------- ---------- ---------- -YVDQLNQIL QILG------ ---------- -----TPD-E
   669145  --IDVWSVGC ILAELLAGKP IFKGRD---- ---------- ---------- ---------- ---------- ---------- ---------- ---------- ---------- -YVDQLNQIL HYLG------ ---------- -----TPS-E
    46685  --IDMWSVGC ILAELLGGRP IFKGRD---- ---------- ---------- ---------- ---------- ---------- ---------- ---------- ---------- -YVDQLNQIL HYLG------ ---------- -----TPS-E
    53954  --IDIWSVGC VLAELLGGRP IFKGKD---- ---------- ---------- ---------- ---------- ---------- ---------- ---------- ---------- -YIDQLNIVL HFLG------ ---------- -----TPS-D
  1446322  --IDVWSVGC ILAELLGGRP IFKGRD---- ---------- ---------- ---------- ---------- ---------- ---------- ---------- ---------- -YVDQLNQIL HYLG------ ---------- -----TPS-E
   351754  --IDVWSVGC ILAELLGGKP IFKGKD---- ---------- ---------- ---------- ---------- ---------- ---------- ---------- ---------- -YVDQLNQIL HYLG------ ---------- -----TPS-E
    65873  --IDIWSVGC ILAELLGGRP IFKGRD---- ---------- ---------- ---------- ---------- ---------- ---------- ---------- ---------- -YVDQLNQIL HVLG------ ---------- -----TPS-E
    52469  --IDIWSVGC ILAELLGGRP IFKGRD---- ---------- ---------- ---------- ---------- ---------- ---------- ---------- ---------- -YVDQLNQIL HVLG------ ---------- -----TPS-E
    30002  --IDIWSVGC ILAELLGGRP IFKGRD---- ---------- ---------- ---------- ---------- ---------- ---------- ---------- ---------- -YVDQLNQIL HVLG------ ---------- -----TPS-E
   154156  --IDMWSLGC IFAELLGSKP LFKGRD---- ---------- ---------- ---------- ---------- ---------- ---------- ---------- ---------- -FVDQLNQIL YILG------ ---------- -----TPD-D
   166841  --IDMWSLGC IFAELLGSKP LFKGRD---- ---------- ---------- ---------- ---------- ---------- ---------- ---------- ---------- -FVDQLNQIL YILG------ ---------- -----TPD-D
   238118  --IDMWSVGC VFAELLGGKP LFKGRH---- ---------- ---------- ---------- ---------- ---------- ---------- ---------- ---------- -FVDQLNQIL YVLG------ ---------- -----TPD-D
   143069  --IDMWSVGC VFAELLGGKP LFKGRH---- ---------- ---------- ---------- ---------- ---------- ---------- ---------- ---------- -FVDQLNQIL YVLG------ ---------- -----TPD-D
   456995  --IDVWSMGC ILAELLGGKP IFKGRD---- ---------- ---------- ---------- ---------- ---------- ---------- ---------- ---------- -YVDQLNQIL HYLG------ ---------- -----TPS-E
   175880  --IDVWSVGC ILAELLGGKP IFKGRD---- ---------- ---------- ---------- ---------- ---------- ---------- ---------- ---------- -YVDQLNQIL HYLG------ ---------- -----TPS-E
    28309  --VDIWSTGC ILAELLGRKP MFEGKD---- ---------- ---------- ---------- ---------- ---------- ---------- ---------- ---------- -YVDHLNHIL QILG------ ---------- -----TPP-E
   767958  --VDIWSTGC ILAELLGRKP MFEGKD---- ---------- ---------- ---------- ---------- ---------- ---------- ---------- ---------- -YVDHLNHIL QILG------ ---------- -----TPP-E
   126152  --VDIWSTGC ILAELLGRKP MFEGKD---- ---------- ---------- ---------- ---------- ---------- ---------- ---------- ---------- -YVDHLNHIL QILG------ ---------- -----TPP-E
   613191  --IDVWSVGC ILAELLGGRP FFKGRD---- ---------- ---------- ---------- ---------- ---------- ---------- ---------- ---------- -YVDQLNQIL HILG------ ---------- -----TPN-E
   198058  --IDVWSVGC ILAELLGGRP FFKGRD---- ---------- ---------- ---------- ---------- ---------- ---------- ---------- ---------- -YVDQLNQIL HILG------ ---------- -----TPN-E
   123006  --IDVWSVGC ILAELLGGRP FFKGRD---- ---------- ---------- ---------- ---------- ---------- ---------- ---------- ---------- -YVDQLNQIL HILG------ ---------- -----TPN-E
   178993  --IDVWSVGC ILAELLGGRP FFKGRD---- ---------- ---------- ---------- ---------- ---------- ---------- ---------- ---------- -YVDQLNQIL HILG------ ---------- -----TPN-E
   122967  --IDVWSVGC ILAELLGGRP FFKGRD---- ---------- ---------- ---------- ---------- ---------- ---------- ---------- ---------- -YVDQLNQIL HILG------ ---------- -----TPN-E
   120501  --IDVWSVGC ILAELLGGRP FFKGRD---- ---------- ---------- ---------- ---------- ---------- ---------- ---------- ---------- -YVDQLNQIL HILG------ ---------- -----TPN-E
    99018  --IDVWSVGC ILAELLGGKP FFKGRD---- ---------- ---------- ---------- ---------- ---------- ---------- ---------- ---------- -YVDQLNQIL HYLG------ ---------- -----TPN-E
   213678  --IDVWSVGC ILAELLGGKP FFKGRD---- ---------- ---------- ---------- ---------- ---------- ---------- ---------- ---------- -YVDQLNQIL HYLG------ ---------- -----TPN-E
   460523  --IDVWSVGC ILAELLGGKP FFKGRD---- ---------- ---------- ---------- ---------- ---------- ---------- ---------- ---------- -YVDQLNQIL HYLG------ ---------- -----TPN-E
   625621  --IDVWSVGC ILAELLGGRP FFKGRD---- ---------- ---------- ---------- ---------- ---------- ---------- ---------- ---------- -YVDQLNQIL HFLG------ ---------- -----TPN-E
   265871  --IDVWSVGC ILAELLGGRP FFKGRD---- ---------- ---------- ---------- ---------- ---------- ---------- ---------- ---------- -YVDQLNQIL HYLG------ ---------- -----TPN-E
    53464  --IDVWSVGC ILAELLGGRP FFKGRD---- ---------- ---------- ---------- ---------- ---------- ---------- ---------- ---------- -YVDQLNQIL HYLG------ ---------- -----TPN-E
   105414  --IDVWSVGC ILAELLGGKP FFKGRD---- ---------- ---------- ---------- ---------- ---------- ---------- ---------- ---------- -YVDQLNQIL HFLG------ ---------- -----TPS-E
   291876  --IDVWSVGC ILAELLGGKP FFKGRD---- ---------- ---------- ---------- ---------- ---------- ---------- ---------- ---------- -YVDQLNQIL HFLG------ ---------- -----TPS-E
    50744  --IDIWSVGC ILGELLGGKP LFRGKD---- ---------- ---------- ---------- ---------- ---------- ---------- ---------- ---------- -YVDQLNQIL MVLG------ ---------- -----TPK-E
   863837  --IDMWSVGC IFAEMLGGKP LFKGRD---- ---------- ---------- ---------- ---------- ---------- ---------- ---------- ---------- -YVDQLNQIL QILG------ ---------- -----TPD-E
    81355  --IDVWSVGC ILAELLGGKP IFKGRD---- ---------- ---------- ---------- ---------- ---------- ---------- ---------- ---------- -YVDQLNQIL HYLG------ ---------- -----TPS-E
   152896  --IDVWSVGC ILAELLAGKP IFKGRD---- ---------- ---------- ---------- ---------- ---------- ---------- ---------- ---------- -YVDQLNQIL HYLG------ ---------- -----TPS-E
  1196002  --IDVWSVGC ILAELLGGRP IFKGRD---- ---------- ---------- ---------- ---------- ---------- ---------- ---------- ---------- -YVDQLNQIL HYLG------ ---------- -----TPKCR
  1195972  --IDVWSVGC ILAELLGGRP IFKGRD---- ---------- ---------- ---------- ---------- ---------- ---------- ---------- ---------- -YVDQLNQIL HYLG------ ---------- -----TPKCR
    49917  --IDIWAVGC IFAEMLGRVP LFRGKN---- ---------- ---------- ---------- ---------- ---------- ---------- ---------- ---------- -QAHQMMEIS RVLG------ ---------- -----RPT-D
   116823
   271167
   151286
    97820
   167647
   430394
   266211
   266223
   266189
   151328
   800193  TTETIVERIE EPSKKLDSVI VEENQD---- -MRQMLSPSP EVYRQITTPT DPLINYARRS S--------- ---------- ---------- ---------- -----IMQPS RQGSTYNSPI PSAQNLSFIA SPEAIGGQVG GSSIAFPTQG
    22041  TTETIVERIE EPSKKLDSVI VEENQD---- -MRQMLSPSP EVYRQITTPT DPLINYARRS S--------- ---------- ---------- ---------- -----IMQPS RQGSTYNSPI PSAQNLSFIA SPEAIGGQVG GSSIAFPTQG
    22014  TTETIVERIE EPSKKLDSVI VEENQD---- -MRQMLSPSP EVYRQITTPT DPLINYARRS S--------- ---------- ---------- ---------- -----IMQPS RQGSTYNSPI PSAQNLSFIA SPEAIGGQVG GSSIAFPTQG
    22020  TTETIVERIE EPSKKLDSVI VEENQD---- -MRQMLSPSP EVYRQITTPT DPLINYARRS S--------- ---------- ---------- ---------- -----IMQPS RQGSTYNSPI PSAQNLSFIA SPEAIGGQVG GSSIAFPTQG
   448385  -SITPTDPVV TYARRSSILG ---------- ---------P SRQNSTFSS- -PMVQQQHLP S--------- ---------- ---------- ---------- -----YAEGP MHAEPG---- ---------- -----SVVSG GG-VVFPSHG
   122008  IAPMPVDPVV TYARRSSVLR SQSYSS---- Y----HAPSP RSRHQP-IP- -SFDGHYHPR T--------- ---------- ---------- ---------- -----GLSRV VNADAGEALE TAASTS---- -AGTAQPAVA GS-VPFPGSG
   578295  PADMANDPVV TYARRSSILQ AQYLQQ---- LQKQGNSVSP VRRTQPGMP- -VYAEESNLP T--------- ---------- ---------- ---------- -----GIANF YSSQRREGAP FPVPTPPPIV RAPSAGPSAT GS-IPFPSTG
   139503  IPAVTRDPLV SYARRTSMFG AHSNRG---- SLYESATGSP ---SDPYQP- -MSTGTHPTP T--------- ---------- ---------- ---------- -----RASSF IDDGSGVNFG GYALGSG--- HVKDSSTSTA GG-IAFPSMQ
  2468658  PAPPQTAPQM SEAARIAAMD AATLAP---- -----GVVAR RRTRSATTD- -PVVTYARRS S--------- ---------- ---------- ---------- -----ILQPR QSSMYGTQFP AAIEGLP--- -----STSEP AG-IAFPSQG
  2215386  PAPPQTAPQM SEAARIAAMD AATLAP---- -----GVVAR RRTRSATTD- -PVVTYARRS S--------- ---------- ---------- ---------- -----ILQPR QSSMYGTQFP AAIEGLP--- -----STSEP AG-IAFPSQG
    88816  PADMVNDPVV SYARRSSILH AQHAQQKFDL QHTASSSVSP TRRFSGIPA- -NYQDFEYMP GMTY------ ---------- ---------- ---------- -----YAPPS VGPQMREGMP TPPPMMASSI SSGASSTAGT NGMIPFPTTG
    88949  PADMVNDPVV SYARRSSILH AQHAQQKFDL QHTASSSVSP TRRFSGIPA- -NYQDFEYMP GMTY------ ---------- ---------- ---------- -----YAPPS VGPQMREGMP TPPPMMASSI SSGASSTAGT NGMIPFPTTG
    88917  PADMVNDPVV SYARRSSILH AQHAQQKFDL QHTASSSVSP TRRFSGIPA- -NYQDFEYMP GMTY------ ---------- ---------- ---------- -----YAPPS VGPQMREGMP TPPPMMASSI SSGASSTAGT NGMIPFPTTG
   206606  DPKPAAIPFP TTEDRPALHN RAPSSS---- -----STLRP VRSSGPLSSV DPYLTYARRS NSLI------ ---------- ---------- ---------- -----FTPAS SSQPHRRLYS EHVDRL---- -----APEDG AG----SGDD
   123647  -FPSTQDPVI AYHRRSLFGQ PSRTSS---- ---------- ---------- -AFSIHRDRT V--------- ---------- ---------- ---------- -----SPNVS EGQG------ ---------- --------AA SSTVAFPSTE
   143931  -FPSTQDPVI AYHRRSLFGQ PSRTSS---- ---------- ---------- -AFSIHRDRT V--------- ---------- ---------- ---------- -----SPNVS EGQG------ ---------- --------AA SSTVAFPSTE
   165009  PSAGERDLML AYQRRSSTFL PVRTSR---- ---------- ---------- -TNSIYSARS ---------- ---------- ---------- ---------- -------HEG GDAS------ ---------- --------QA ASTVAFPSQQ
   580315  PSSER-DSIP PYPRRSSFMR PSRTNS---- ---------- ---------- -TYSTYAARN R--------- ---------- ---------- ---------- -----HSIAY QPESRNSLER G--------- -------PSG ESTVAFPTQ-
   492840  PSSERGDSVA PYPRRPSFMR PSRTNS---- ---------- ---------- -MYSTYAARN R--------- ---------- ---------- ---------- -----HSIAY QPESRNSLER G--------- -------PDG ESSVAFPTQ-
   594182  RRSQIADPVY SYVRRSSFMQ PSRTSS---- ---------- ---------- -TYSQF--RN R--------- ---------- ---------- ---------- -----HSVAY PPEHRNSLER AAGVGAG--- -----TEAGG ESTIAFPTQ-
   168238  PRPQGGDPVY TYVRRSSFMQ PSRTSS---- ---------- ---------- -TYSTF--RS R--------- ---------- ---------- ---------- -----HSVAY PPENRSSLER AVGLGSGMPT GTGTGTSASG ESTIAFPTQP
   107302  PRPQGGDPVY TYVRRSSFMQ PSRTSS---- ---------- ---------- -TYSTF--RS R--------- ---------- ---------- ---------- -----HSVA- ---------- ---------- ---------G ESTIAFPTQP
    64718  GANTPTDPLL TYSRRSSFFG PRRRDS---- ---------- ---------- ----SYASSP YVRN------ ---------- ---------- ---------- -----QTLPP SRGEPAFVAE PGAMAP---- ------GTVG SNSVAFPSES
    64830  GANTPTDPLL TYSRRSSFFG PRRRDS---- ---------- ---------- ----SYASSP YVRN------ ---------- ---------- ---------- -----QTLPP SRGEPAFVAE PGAMAP---- ------GTVG SNSVAFPSES
  1154823  GANTPTDPLL TYSRRSSFFG PRRRDS---- ---------- ---------- ----SYASSP YVRN------ ---------- ---------- ---------- -----QTLPP SRGEPAFVAE PGAMAP---- ------GTVG SNSVAFPSES
   433002  RSTTPTDPVV TYARRSSILQ PARQSS---- ---------- ---------- ----TSAYNS ---------- ---------- ---------- ---------- ---------P LASTIQHALP TVFDGL---- ------PTTS SHGVAFPSET
   822334  RSTTPTDPVV TYARRSSILQ PARQSS---- ---------- ---------- ----TSAYNS ---------- ---------- ---------- ---------- ---------P LASTIQHALP TVFDGL---- ------PTTS SHGVAFPSET
   511572  RPCTPEDPVV NYARRSSILQ PQRQPS---- ---------- ---------- ----TSTYSS ---------- ---------- ---------- ---------- ---------P PASTTQHPFV PMFDGL---- ------PTSA S-GIAFPNEG
   688421  PTPTPTDPVV TYARRSSIMQ PPRQPS---- ---------- ---------- ----N--YNS ---------- ---------- ---------- ---------- ---------P SASASQHPWP SGTDGL---- ------PTNS S-VVPFPGQT
    17778  PTPTPTDPVV TYARRSSIMQ PPRQPS---- ---------- ---------- ----N--YNS ---------- ---------- ---------- ---------- ---------P SASASQHPWP SGTDGL---- ------PTNS S-VVPFPGQT
  1336823  -EEIIIDDLA SPRRRDPSTL VATTD----- ---------- ---------- -PIASYRRRS SILQ------ ---------- ---------- ---------- -----GGVSP HHSWGASQVQ HQIF------ ------PSFS QSESAGEGS-
   605623  AQDEMLPPAI QRLRHGRYAS VAD------- ---------- ---------- -PLMSYSRRT SIFS------ ---------- ---------- ---------- -----VPDNL RYHPQATGTP PSGLP----- ------RESN EAAYPFPTVN
   511468  -PCTPEDPVV NYARRSSILQ PQRQPS---- ---------- ---------- ----TSTYSS ---------- ---------- ---------- ---------- ---------P PASTTQHPFV PMFDGL---- -------PTS ASGIAFPNEG
    60300
```

```
    2435698
    1222436
    1172380  RATTPVDPVV TYARRSSIMQ PSRQPS---- ---------- -------AVN SPMMNSQQLP TIDG------ ---------- ---------- ---------- -----LPTMG NSSGIAF
    1151825
     410058
      57461
     311323  RPISLLNVAG ISS--FCVEY IALAAS---- SPISPPTP-I PAPCFIPPPT FAGMQTYTNP FTPT------ ---------- ---------- ---------- ---------- ----NTSTSP ALVLSSKT-- ----WRLLLF TAPAPAPAR-
     143102  ESLRLSSNQI GVV--LAIAP FVQSVACPVW TILADKKPSW HGPLMAILIA IGGTSVMTLM FLPN------ ---------- ---------- ---------- -----WLMRD TCANNPSCDP AVTKTDES-- ----STGLIF AAVLALIFA-
    2750129  LLARLNAAAA GET--YAPAT GVVAEG---- GFVKPTEEGE NANAAEVKEN AGEAKEKTKE ELKR------ ---------- ---------- ---------- -----EKKEK KRKRKEQEGE DVEKKSKK-- ----RKKDQS EGNTPTPVA-
    2702932  LLARLNAAAA GET--YAPAT GVVAEG---- GFVKPTEEGE NANAAEVKEN AGEAKEKTKE ELKR------ ---------- ---------- ---------- -----EKKEK KRKRKEQEGE DVEKKSKK-- ----RKKDQS EGNTPTPVA-
     108010  LTGYDESEVV GRNCRFLQAP NGAVTKGEQR RFTSPEAVSY MRKSLAADKE CQTSIVNYRK NGTA------ ---------- ---------- ---------- -----FINLV TVIPIPGGEQ GLPHEEHEVY YHVGFQVDLN EQPNAILDKL
    1754091  SMGVSGGYQS ARRGPSI-SP SGDRAAVER- --VLPLLKKF AAKDPKTGKP C---VANLGP GPCG------ ---------- ---------- ---------- -----HYVKM VHNGIEQGML GIVNETWEIL YKC-LHIDL- DTISSVYKKW
     564944  RVLEDPDASC YIYTPAWFEH NETRGPRPKR LTARRVFDAM YRTAADLGLP TGSRYVSACV LACG------ ---------- ---------- ---------- -----HLAEN VPRPYTFAQE --MAQSLSVL AVDVWAVRMW STLTPRPDR-
    AtMPK18  RVPPSGRPGR VVESSVSYEN ----GRNLKE AYFRSAVSSP HCYFRPNTMT NPENRNIEAS SFPP------ ---------- ---------- ---------- -----KPQNP VHQFSPTEPP AATTNQADVE TMNHPNPYFQ PQLPKTD---
    AtMPK19  RVP-SGKPGR VVESSVTYEN ----DRNLKE SSYDARTSYY RSTVLPPQTV SPNCYFLPNT MNQE------ ---------- ---------- ---------- -----KRSGT EAASQPKPQF VPTQCNSAKP AELNPNPYVQ SQ--------
    AtMPK20  RNLSAAKPST FMGPVAPFDN ----GRISRD AYDPRSFI-- RSTNLPFSQQ SAATVAMGKQ QERR------ ---------- ---------- ---------- -----TTMEP EKQARQISQY NRYAPDVAIN I-DNNPFIMA RTGMNKA---
  OsMPK20-1  QRVPTARPGR VVGPVLPYEN ----GA-TKD SYDARRLA-- MNSGYPPQQQ IPQAYGYYQI PGKS------ ---------- ---------- ---------- -----ACSEL SQAERYTLHQ QAYTCANSAT V-TDVALDMR APPFHLSGG-
  OsMPK20-4  QRIP-GRTGR GACPVIPFEN ----LS-AMG PYNQRRVV-- RNPVLPPATT NLSAYAYHRK SDSS------ ---------- ---------- ---------- -----ERELQ QELEKDRMRY QPSEHFMDAK VVSHMSHDLR ASSYYVSKA-
  OsMPK20-5  QRIPTARPGR TVGPVLPFEN ----PG-AAD PHSARRVV-- RNPMVPPAAA NKSGYSYNLK SDYS------ ---------- ---------- ---------- -----DRQHQ EELEKDRVQY RPAQHLMDAK VAPDTAPDIR SSQYYFTRSA
  OsMPK20-3  HANIAGEPST IVRPAVSSER ----SLAPTL QWQPNMTHFL NHALCYQNTV FSGSLLDATG PAQA------ ---------- ---------- ---------- -----IPRTT PYVDYRSGNL DLYQHHVSRE ---DVQSDTA TAQAHAASHG
    AtMPK16  QGAAVARPGK VVGSVLRYNN CGAATGVEAL EQQQRRMVRN PAAA------ --SQYPKRTQ PCKS------ ---------- ---------- ---------- -----NRGDE DCATAAEGPS RLKPNTQYIP QKVSAAQDTA MSRWY
  OsMPK16-1  QGAA-ARPGK AVGSVLRYGN C--STSAAEQ QYEQRRVVRN PAIAPNSSVP LGSSYPRRNQ TCKS------ ---------- ---------- ---------- -----ETGDV ERIDS----S QTGPPKPYVA NKLPATVDGR SGHW
    AtMPK17  SACLMRSDSI CASRCVGVSS AVS
     AtMPK8  ARNLMKSASI SGSKCIGVQS KTDK-EDTIA EEEDNETVAE LTDKVASLHN S
    AtMPK15  ARNLMKSSSI SGSKCIGVQS KTNI-EDSIV EEQD-ETVAV ---KVASLHN S
  OsMPK17-1  SRSYLKSASI SASKCVAVKD NKEP-EDDYI SEEMEGSVDG LSEQVSRMHS
     AtMPK9  ARSLLKSASI SASKCIGMKP RNKS-E---Y GESNNDTVDA LSQKVAALHT
  OsMPK21-1  ARSLLKSESI SASKCIGEKP KQDRDQEDSL TESMDETADE VSEKVAQLKT
  OsMPK21-2  ARNFLKSESI GASQCVVIKE KREKDEE-SM SEYMNEAADG VPHKIAQLKT
  OsMPK17-2  PKIYLKSASI SASTCIIRGN KGPK--ENGI SEDMEEVVYE LSDNVTRMLS
      30260
     130090
     599215
     219362
      56051
     139809
       1060
     184799
      68008
      99901
     104502
     645859
     599559
 36518:4-24
     178963
     196817
     738557
    1159263
     176895
     664529
    1194920
     432994
      98579
      88826
      54157
     914084
     503422
      95205
     229298
     455126
     256867
     419576
      74409
     521809
      54281
      48349
     594110
      27538
      96623
     380926
     263236
     154671
      67562
     226444
    1137209
    AtMPK13
    AtMPK11
     459393
      69938
     591714
     107905
      99046
     863830
     287494
     662188
     492594
     176887
     618168
     492643
     125995
     122928
     154756
      68423
  OsMPK20-2
  Consensus   .id.wsvgc i.ae.l.gkp lfpg.d                                                                                     ...ql..i. ..lg                    tp...

             1201                                                                                                                                                  1350
     127228  -DFY---GIK SRRARDYIRS LPFKKR---- ----IPFSRL F-P--NANPM ---------- ---------- --------AV DLLEKLLTFN PAKRITVEEA LKHPYLEPY- ----HDPDDE PDAP--PIHE SFFDFDKYKD QLTKEQLK--
     287090  -DFY---GIK SRRARDYIRS LPFKKR---- ----IPFSRL L-P--NANPL ---------- ---------- --------AV DLLEKLLTFN PEKRITVEEA LKHPYLEPY- ----HDPDDE PDAP--PIPD SFFDFDRYKE QLTKEQLK--
     186494  -DFY---GIK SRRARDYIRS LPFKKR---- ----IPFARL F-P--HANPM --------GK SLSFVPVIPS LMLTPSHIAV DLLEKLLTFN PTKRTTVEEA LKHPYLEPY- ----HDPDDE PDAP--PIPE SFFDFDRYKD QLTKEQLK--
     260272  -DFY---AIK SRRARDYIRG LPFKKR---- ----IPFSRL V-P--NANAM ---------- ---------- --------AL DLLEKLLTFN PVKRITVEEA LKHPYLEPY- ----HDPDDE PGAP--PIPE NFFDFDRYKD QLTKEQLK--
     119975  -DFY---GIK SRRARDYIRS LPFKKR---- ----IPFSRL F-P--TASPM ---------- ---------- --------AV DLLEKLLTFN PEKRITVAEA LEHPYLEPY- ----HDPNDE PDAP--PINE SFFDFDKYKD QLTKEQLK--
       2147  -DYY---AVK SARAREYIRS LPFKKR---- ----VPFASM FGA--DTNPL ---------- ---------- --------AI DLLEKMLTFN PSKRITVEDA LKHPYLEPY- ----HDPEDE PTAP--PIPE GFFDFDRRKD ELSKEQLK--
     692376  -DYY---GIK SRRAREYIRS LPFKKK---- ----IPWKAM F-P--KTSDL ---------- ---------- --------AL DLLERLLAFN PVKRITVEDA LKHPYLEPY- ----HDPDDE PTAD--PIPE EFFDFDKNKD NLTKEQLK--
     698785  -DYY---GIK SRRAREYIRS LPFKKK---- ----IPWKAM F-P--KTSDL ---------- ---------- --------AL DLLERLLAFN PVKRITVEDA LKHPYLEPY- ----HDPDDE PTAD--PIPE EFFDFDKNKD NLTKEQLK--
     468116  -DYY---GIK SRRAREYIRS LPFKKK---- ----IPWKVM F-P--RTSDL ---------- ---------- --------AL DLLERLLAFN PMKRITVEEA LKHPYLEPY- ----HDPDDE PTAD--PIPE EFFDFDKNKD TLTKEQLK--
     274271  -DYY---GIK SRRAREYIRS LPFKKK---- ----IPWKAM F-P--KTSDL ---------- ---------- --------AL DLLDKLLAFN PVKRITVEEA LKHPYLEPY- ----HDPEDE PNAD--PIPE EFFDFDKNKD NLSKEQLK--
     255486  -DYY---GIK SRRAREYIRS LPFKKK---- ----IPWRAM F-P--KTSDL ---------- ---------- --------AL DLLDKMLAFN PVKRITVEDA LRHPYLEPY- ----HDPDDE PSAE--PIPE EFFDFDKNKD SLSKEQLR--
     285227  -DYY---GIK SRRAREYIRS LPFKKK---- ----IPWKVM F-P--KTSDL ---------- ---------- --------AL DLLEKLLAFN PMKRISVEDA LKHPYLEPY- ----HDPDDE PGAD--PIPE EFFDFDKNKD TLSKEQLK--
      18186  -DYY---GIK SRRAREYIRS LPFKKK---- ----VPFRTL F-P--KTSDL ---------- ---------- --------AL DLLEKLLAFN PVKRITVEEA LKHPYLEPY- ----HDPDDE PTAP--PIPE EFFDFDKHKD NLSKDQLK--
     465108  -DYY---GIK SRRAREYIRS LPFKKK---- ----IPFKHL F-P--KTNDL ---------- ---------- --------AL DLLEKLLAFN PAKRITVEEA LRHPYLEPY- ----HDPDDE PTAD--PIPE EFFDFDKNKD ALSKEQLK--
    1164585  -DYY---GIK SRRAREYIRS LPFKKK---- ----VPFRTL F-P--KTSDL ---------- ---------- --------AL DLLEKLLAFN PVKRITVEDA LKHPYLEPY- ----HDPDDE PTAP--PIPE EFFDFDKHKD TLSKEQLK--
      93851  -DYY---GIK SRRAREYIRS LPFKKK---- ----IPWKAM F-P--KTSDL ---------- ---------- --------AL DLLEKLLAFN PTKRITVEEA LKHPYLEPY- ----HDPEDE PNAE--PIPE EFFDFDKNKD NLSKEQLK--
     108267  -DYY---GIK SRRAREYIRS LPFKKK---- ----IPWTAM F-P--KTSDL ---------- ---------- --------AL DLLEKLLAFN PAKRITVEEA LKHPYLEPY- ----HDPEDE PTAD--PIPE EFFDFDKNKD NLSKEQLK--
      59022  -DYY---GIK SRRAREYIRS LPFKKK---- ----IPFKAL F-P--KSNEL ---------- ---------- --------AL DLLEKLLAFN PAKRITVEEA LRHPYLEPY- ----HDPDDE PTAD--PIPE EFFDFDKNKD NLSKEQLK--
     370137  -DYY---GIK SRRAREYIRS LPFKKK---- ----IPWKAM F-P--KTSDL ---------- ---------- --------AL DLLERLLAFN PVKRITVEEA LKHPYLEPY- ----HDPDDE PTAD--PIPE EFFDFDKNKD NLTKEQLK--
     532216  -DYY---GIK SRRAREYIRS LPFKKK---- ----IPWKAM F-P--KTSDL ---------- ---------- --------AL DLLERLLAFN PVKRITVEEA LKHPYLEPY- ----HDPDDE PTAD--PIPE EFFDFDKNKD NLTKEQLK--
      31240  -DYY---GIK SRRAREYIRS LPFKKK---- ----IPWKAL F-P--KTNDQ ---------- ---------- --------AL DLLERLLAFN PVKRITVEEA LKHPYLEPY- ----HDPEDE PTAD--PIPP EFFDFDKNKD NLTKEQLK--
     160345  -DYY---GIK SRRAREYIRS LPFKKK---- ----IPFKVM F-P--QTNEL ---------- ---------- --------AL DLLEKLLAFN PAKRITVEEA LKHPYLEPY- ----HDPEDE PTAE--PIPE EFFDFDKNKD NLSKEELK--
     212060  -DYY---GIK SRRAREYIRS LPFKKK---- ----IPFKVM F-P--QTNEL ---------- ---------- --------AL DLLEKLLAFN PAKRITVEEA LKHPYLEPY- ----HDPEDE PTAE--PIPE EFFDFDKNKD NLSKEELK--
     327005  -DYY---GIK SRRAREYIRS LPFKKK---- ----IPWKVM F-P--KTSDL ---------- ---------- --------AL DLLERLLAFN PVKRITVEEA LKHPYLEPY- ----HDPEDE PTAE--PIPE EFFDFDKNKD NLTKEQLK--
      48412  -DYY---GIK SRRAREYIRS LPFKKK---- ----IPWKAM F-P--KTSDL ---------- ---------- --------AL DLLERLLAFN PVKRISVEDA LKHPYLEPY- ----HDPEDE PTAD--PIPE EFFDFDKSKD NLSKEQLK--
     606069  -DYY---GIK SRRAREYIRS LPFKKK---- ----VPFKQM F-P--KTSDL ---------- ---------- --------AL DLLEKLLAFN PVKRITVDEA LQHPYLEPY- ----HDPDDE PTAS--PIPE EFFDFDKNKD NLSKEQLK--
     123724  -DYY---GIK SRRAREYIRS LPFKKK---- ----VPFKNM F-P--KTSDL ---------- ---------- --------AL DLLEKLLAFN PVKRITVEEA LQHPYLEPY- ----HDPEDE PTAA--PIPE EFFDFDKNKD NLTKEQLK--
     342035  -DYY---GIK SRRAREYIRS LPFKKK---- ----VPFRTL F-P--KTSDL ---------- ---------- --------AL DLLEKLLAFN PVKRITVEEA LKHPYLEPY- ----HDPDDE PTAP--PIPE EFFDFDKHKD SLSKEQLK--
    2135321  -DYY---GIK SRRAREYIRS LPFKKK---- ----VPFRTL F-P--NTSEL ---------- ---------- --------AL DLLEKLLAFN PVKRITVEEA LKHPYLEPY- ----HDPDDE PTAP--PIPE EFFDFDKHKD NLTKEQLK--
     644393  -DYY---GIK SRRAREYIRS LPFKKK---- ----VPFRTL F-P--NTSEL ---------- ---------- --------AL DLLEKLLAFN PVKRITVEEA LKHPYLEPY- ----HDPDDE PTAP--PIPE EFFDFDKHKD NLSKEQLK--
     784446  -DYY---GIK SRRAREYIRS LPFKKK---- ----VPFRTL F-P--NTSDL ---------- ---------- --------AL DLLEKLLAFN PVKRITVEDA LKHPYLEPY- ----HDPDDE PTAP--PIPE EFFDFDKHKD NLSKEQLK--
     567834  -DYY---GIK SRRAREYIRS LPFKKK---- ----VPFRTL F-P--NTSDL ---------- ---------- --------AL DLLEKLLAFN PVKRITVEEA LQHPYLEPY- ----HDPEDE PTAP--PIPE EFFDFDKHKD NLSKEQLK--
     389770  -DYY---GIK SRRAREYIRS LPFKKK---- ----VPFRTL F-P--NTSDL ---------- ---------- --------AL DLLEKLLAFN PVKRITVEEA LQHPYLEPY- ----HDPEDE PTAP--PIPE EFFDFDKHKD NLSKEQLK--
      63860  -DYY---GIK SRRAREYIRS LPFKKK---- ----VPFRTL F-P--NTSEL ---------- ---------- --------AL DLLEKLLAFN PVKRITVEEA LKHPYLEPY- ----HDPDDE PTAP--PIPE EFFDFDKHKD NLSKEQLK--
     131603  -DYY---GIK SRRAREYIRS LPFKKK---- ----VPFRTL F-P--NTSEL ---------- ---------- --------AL DLLEKLLAFN PVKRITVEEA LKHPYLEPY- ----HDPDDE PTAP--PIPE EFFDFDKHKD NLSKEQLK--
     495859  -DYY---GIK SRRAREYIRS LPFKKK---- ----IPFKAL F-P--KSNDL ---------- ---------- --------AL DLLEKLLAFN PAKRITVDDA LRHPYLEPY- ----HDPDDE PTAP--PIPE GFFDFDKNKD ALSKEQLK--
```

```
    434551  -DYY---GIK SRRAREYIRS LPFKKK---- ----IPFKAL F-P--KSNDL ---------- ---------- --------AL DLLEKLLAFN PAKRITVDDA LRHPYLEPY- ----HDPDDE PTAP--PIPE GFFDFDKNKD ALSKEQLK--
    140828  -DYY---GIK SRRAREYIRS LPFKKK---- ----VPFRTL FNP--NTSEL ---------- ---------- --------AL DLLEKLLAFN PVKRITVEEA LKHPYLEPY- ----HDPDDE PTAP--PIPE EFFDFDKHKD NLSKEQLK--
     66435  -DYY---GIK SRRAREYIRS LPFKKR---- ----IPFSTM F-P--KTSPQ ---------- ---------- --------AL DLLEKLLAFN PAKRITVEEA LKHPYLEPY- ----HDPEDE PTAP--PIPD EFFDFDRRKD ELSKEQLK--
    383913  -DYY---GIK SRRAREYIRS LPFKKK---- ----IPFRAM F-P--KSNDM ---------- ---------- --------AL DLLEKLLAFN PAKRISVEDA LRHPYLEPY- ----HDPEDE PTAP--PIPD GFFDFDKNKD ALSKEQLK--
    323815  -DYY---GIK SRRAREYIRS LPFKKK---- ----IPFRAL F-P--KSNDT ---------- ---------- --------AL DLLEKLLAFN PAKRISVEDA LRHPYLEPY- ----HDPEDE PTAP--PIPD GFFDFDKNKD ALSKEQLK--
    396599  -DYY---GIK SRRAREYIRS LPFKKK---- ----IPFRAL F-P--KSNDL ---------- ---------- --------AL DLLEKLLAFN PAKRISVEDA LRHPYLEPY- ----HDPEDE PTAP--PIPD GFFDFDKNKD ALSKEQLK--
     74447  -DYY---GIK SRRAREYIRS LPFKKK---- ----IPFRAL F-P--KSNDL ---------- ---------- --------AL DLLEKLLAFN PAKRISVEDA LRHPYLEPY- ----HDPEDE PTAP--PIPE GFFDFDKNKD SLSKEQLK--
     83363  -DYY---GIK SRRAREYIRS LPFKKK---- ----IPFRAL F-P--KSNDT ---------- ---------- --------AL DLLEKLLAFN PAKRISVEDA LRHPYLEPY- ----HDPEDE PTAP--PIPD GFFDFDKNKD ALSKEQLK--
     50905  -DYY---GIK SRRAREYIRS LPFKKR---- ----IPFSTM F-P--KTSPQ ---------- ---------- --------AL DLLEKLLAFN PAKRITVEEA LKHPYLEPY- ----HDPEDE PTAP--PIPD EFFDFDRRKD ELSKEQLK--
    650405  -DYY---GIK SRRAREYIRS LPFKKR---- ----IPFATM F-P--KTSPQ ---------- ---------- --------AL DLLEKLLAFN PAKRITVEDA LKHPYLEPY- ----HDPEDE PTAP--PIPD EFFDFDRRKD DLSKEQLK--
    493847  -DYY---GIK SRRAREYIRS LPFKKR---- ----IPFATM F-P--KTSPQ ---------- ---------- --------AL DLLEKLLAFN PAKRITVEDA LKHPYLEPY- ----HDPEDE PTAP--PIPD EFFDFDRRKD DLSKEQLK--
     95409  -DYY---GIK SRRAREYIRS LPFRKR---- ----VPFSVL C-S--KANPL ---------- ---------- --------AI DMLEKLLTFN PLKRITVEEA LKHPYLEPY- ----HDPEDE PMAR--PIPE SFFDFDRVKE TMSKEQLK--
    835420  -DFY---GIK SRRARDYIRT LAFKRR---- ----IPFASM F-P--DANPL ---------- ---------- --------AL DLLEKFLTFN PAKRITVEEA LRHPYLEPY- ----HDPEDE PSMD--PIPE SFFDFDKHKD TLSKEQLK--
    343609  -DFY---GIK SRRARDYIRS LPFKKR---- ----IPFTHM F-P--HANPL ---------- ---------- --------AL DMLEKLLAFN PTKRITVEEA LKHPYLEPY- ----HDPEDE PSAD--PIPE SFFDFDKHKD QLSKEQLK--
     67554  -DYY---GIK SRRAREYIRS LPFKKK---- ----IPWKVM F-P--RTSDL ---------- ---------- --------AL DLLERLLAFN PMKRITVEEA LKHPYLEPY- ----HDPDDE PTAD--PIPE SFFDFDKHKD TLTKEQLK--
    216015  -DYY---NIK SKRAREYIRS LPFCKK---- ----VSFSEL FQS--IP--- ---------- ---------- ---NINPKAL DLLEQLLAFN PTKRITVDKA LKHPYLKLY- ----HDPNDE PMCE--PIPE DFFDFDRRKD ELSVEELK--
     52193  -DYY---NIK SKRAREYIRS LPFCKK---- ----IPFQDL FGN--INPN- ---------- ---------- --VQINPLAI DLLENLLIFN PAKRITVDDA LKHPYLKLY- ----HDPNDE PVSE--KIPE DFFDFDRKKD ELSIDDLK--
     53916  -DYY---NIK SKRAREYIRS LPFCKK---- ----VPFQVL FNN--PD--- ---------- ---------- ----INPLAL DLLENLLIFN PAKRITVDDA LKHPYLKLY- ----HDPNDE PISE--KIPE DFFDFDKRKD ELTVEELK--
     83931  -DYY---SIK SRRAKEYIKS LPLRKK---- ----VPFDKL FPN--A---- ---------- ---------- -----NPLAI DLLEKLLTFN PSKRIKVEDA LKHPYLKLY- ----HDPNDE PIAE--KIPE DFFDFDKKKD ELNIPELK--
     51986  -DYY---SIK SKRAREYIRS LPFRKK---- ----IPFSQI YPH--A---- ---------- ---------- -----NPLAL DLLEKLLTFN PARRLTAEDA LKHPYLKVY- ----HDPNDE PISP--SIPE DFFDFDKRKD ELTTSELK--
     49589  -DYY---GIN SKRAREYIRS LPFKKK---- ----VPFQQL FAN--LKRG- ---------- ---------- -GEQINPDAI DLLEKLLTFN PTKRISVDEA LKHPYVAAY- ----HDPEDE PMAN--PVDE AFFDFDKRKD DLSVSELK--
    862988  -EFY---AIT TRRSRDYIRA LPFRKR---- ----RPFAQI F-P--NASPL ---------- ---------- --------AV DFLTKTLTFD PKKRITVEDA LAHPYLEAY- ----HDPEDE PVAP--PLDP EFFDFDLHKD DISREQLK--
    833500  -EFY---AIT TRRSRDYIRA LPFRKR---- ----RPFAQL F-P--NANLL ---------- ---------- --------AV DFLTKTLTFD PKKRITVEEA LAHPYLEAY- ----HDPEDE PVAP--PLDP EFFEFDLHKD DISREQLK--
    810703  -EFY---AIT TRRSRDYIRA LPFRKR---- ----RPFAQL F-P--NANPL ---------- ---------- --------AV DFLTKTLTFD PKKRISVEDA LAHPYLEAY- ----HDPDDE PVAP--PLDP EFFDFDLHKD DISREQLK--
    457677  -EFY---AIT TRRSRDYIRA LPFRKR---- ----RPFAQL F-P--NANPL ---------- ---------- --------AV DFLTKTLTFD PKKRISVEDA LAHPYLEAY- ----HDPDDE PVAP--PLDP EFFEFDLHKD DISREQLK--
    161161  -EFY---AIT TRRSRDYIRA LPFRKR---- ----KPFAQL F-P--NANPL ---------- ---------- --------AV DFLTRSLTFD PKKRITVEEA LAHPYLEAY- ----HDPDDE PCAP--PLDP EFFDFDLHKD DISREQLK--
   1588194  -EFY---AIT TRRSRDYIRA LPFRKR---- ----KPFAQL F-P--NANPL ---------- ---------- --------AI DFLTRTLTFD PKKRITVEEA LAHPYLEAY- ----HDPEDE PCAP--PLDP EFFEFDLHKD DISREQLK--
     98485  -EFY---AIT TRRSRDYIRA LPFRKR---- ----KPFATL F-P--NASPL ---------- ---------- --------AV DFLTKSLTFD PKKRITVEEA LAHPYLEAY- ----HDPEDE PCAP--PLDP EFFEFDLHKD DISREQLK--
   1443964  -EFY---AIT TRRSRDYIRA LPFRKR---- ----RPFAQL F-P--NASAL ---------- ---------- --------AV DFLTKTLTFD PKKRISVEMA LCHPYLEAY- ----HDPEDE PVAP--PLDP EFFEFDLHKD DISREQLK--
    864221  -EFY---AIT TRRSRDYIRA LPFRKR---- ----RPFAQI F-P--NANPL ---------- ---------- --------AV DFLTKTLTFD PKKRITVEEA LAHPYLEAY- ----HDPDDE PVAP--PLDP EFFDFDLHKD DISREQLK--
    903905  -EFY---AIT TRRSRDYIRA LPFRKK---- ----RPFSVL F-P--NANPL ---------- ---------- --------AI DFLTKTLTFD PKKRITVEDA LAHPYLEAY- ----HDPEDE PVAP--PLDP EFFEFDLHKD DISREQLK--
   1147795  -EFY---AIT TRRSRDYIRA LPFRKK---- ----RPFAQL F-P--NANPL ---------- ---------- --------AV DFLTKTLTFD PKKRITVEDA LAHPYLEAY- ----HDPDDE PVAP--PLDP EFFEFDLHKD DISREQLK--
    622735  -EFY---AIT TRRSRDYIRA LPFRKK---- ----KPFATI F-P--NANPL ---------- ---------- --------AV DFLTKTLTFD PKKRITVEQA LAHPYLEAY- ----HDPDDE PVAP--PLDP EFFEFDLHKD DISREQLK--
    166786  -EFY---AIT TRRSRDYIRA LPFRKK---- ----KPFSQL F-P--NANPN ---------- ---------- --------AI DFLTKTLTFD PKKRITVEQA LAHPYLEAY- ----HDPDDE PVAP--PLDP EFFEFDLHKD DISREQLK--
   2641010  -EFY---AIS TRRSRDYIRA LPFKKK---- ----RSFATL F-P--NATPL ---------- ---------- --------AV DFLSRTLTFD PKKRITVEEA LEHPYLEAY- ----HDPEDE PVAP--PLDP EFFEFDLHKD DISREQLK--
     28679  -EFY---AIT TRRSRDYIRA LPFRKR---- ----KPFAQL F-P--NANSL ---------- ---------- --------AI DFLTKTLTFD PKKRLTVEEA LAHPYLEAY- ----HDPDDE PVAP--PLDP EFFEFDLHKD DISREQLK--
    101027  -EFY---AIT TRRSRDYIRA LPFRKR---- ----RPFAQL F-P--NATPL ---------- ---------- --------AI DFLTKTLTFD PKKRLTVEEA LAHPYLEAY- ----HDPDDE PVAP--PLDP EFFEFDLHKD DISREQLK--
    564549  -EFY---AIT TRRSRDYIRA LPFRKK---- ----KSFATL F-P--NASPL ---------- ---------- --------AV DFLTKTLTFD PKKRLTVEQA LAHPYLEAY- ----HDPEDE PCAP--PLDP EFFEFDLHKD DISREQLK--
    227465  -EFY---AIT TRRSRDYIRA LPFRKR---- ----RPFSAL F-P--NANPL ---------- ---------- --------AI DFLTQTLTFD PKKRITVEKA LAHPYLEAY- ----HDPDDE PVAP--PLDP DFFEFDLHKD DISREQLK--
     18095  -EFY---AIT TRRSRDYIRA LPFRKR---- ----KPFATL F-P--NANPL ---------- ---------- --------AI DFLTKTLTFD PKKRITVEEA LAHPYLEAY- ----HDPDDE PVAP--PLDP EFFEFDLHKD DISREQLK--
    338545  -EFY---AIT TRRSRDYIRA LPFRKK---- ----RPFAQL F-P--NANPL ---------- ---------- --------AV DFLTKTLTFD PKKRITVEDA LAHPYLEAY- ----HDPDDE PVAP--PLDP EFFEFDLHKD DISREQLK--
    134728  -EFY---AIT TRRSRDYIRA LPFRKK---- ----RPFAQL F-P--NASKE ---------- ---------- --------AV DFLTKTLTFD PKKRITVEDA LAHPYLEAY- ----HDPDDE PVAP--PLDP EFFEFDLESS
    275037  -EFY---AIT TRRSRDYIRA LPFRKK---- ----KPFAQL F-P--NANPL ---------- ---------- --------AI DFLTRTLTFD PKKRITVEEA LAHPYLEAY- ----HDPEDE PCAP--PLDP EFFEFDRECP RPYPHF
    503339  -EFY---AIT TRRSRDYIRA LPFRKR---- ----RPFAQI F-P--NANPL ---------- ---------- --------AV DFLTKTLTFD PKKRITVEEA LAHPYLEAY- ----HDPDDE PVAP--PLDP EFFEFDRNTQ GRYQPRTA--
     40745  -EFY---AIN SRRSRDYIRA LPFRKK---- ----RPFTAI Y-P--NASAL ---------- ---------- --------AI DFLNKTLTFD PKKRLTVEEA LQHPYLEAY- ----HDPEDE PVAP--PLDE DFFAFDRQKD DISREELK--
     43876  -EFY---RIN SRRSRDYLRA LPFQKR---- ----RNFAEL F-P--AASEQ ---------- ---------- --------AC DFLAKTLTFD PTKRLTAEQC LEHPYLAQY- ----HDPDDE PVAP--PLDP GFFDFDLEK- -LSREDLK--
     14716  -EFY---RIN SRRSRDYLRA LPFQKR---- ----RNFAEL F-P--AASEQ ---------- ---------- --------AC DFLAKTLTFD PTKRLTAEQC LEHPYLAQY- ----HDPDDE PVAP--PLDP GFFDFDLEK- -LSREDLK--
     15138  -EFY---KIN SRRSRDYLRA LPFTKK---- ----RDFAEL F-P--NASPA ---------- ---------- --------AV DFLTRTLTFD PTKRLTVEQC LEHPYVSAY- ----HDPEDE PTAP--PLPA SFFAFDKQKE AISKDQLK--
     67488  -EFY---AIS SRRSRDYIRA LPFRKK---- ----RSFSQL F-P--DASPE ---------- ---------- --------AV DFLTRTLTFS PKSRMSVEEC LSHPYLSAY- ----HDPDDE PSAP--PLDA SFFEFDHRKD VISKEELK--
     28680  -EFY---AIT TRRSRDYIRA LPFRKR---- ----KPFAQL F-P--NANSL ---------- ---------- --------AI DFLTKTLTFD PKKRLTVEEA LAHPYLEAY- ----HDPDDE PHC------V NSFAWTSHEG PLL
    177514  -EFY---AIT SRRSRDYIRA LPIRKR---- ----RPFATL F-P--HASKD ---------- ---------- --------AI DFLSKTLTFD PKKRLTVDEA LEHPYLSAY- ----HDPDDE PVVA--PLSP TYFEFDLHKE KLSKDQLK--
     53880  -EFY---AIT SRRSRDYIRA LPIRKR---- ----RPFATL F-P--HASKE ---------- ---------- --------AI DFLSKTLTFD PKKRMTVDEA LEHPYLSAY- ----HDPEDE PTVT--PLSP SYFDFDMHKE DLTKDQLK--
    778168  -EFY---AIT SRRSRDYIRA LPIRKR---- ----RPFTAL F-P--HASAE ---------- ---------- --------AI DFLSKTLTFD PKKRITVDEA LEHPYLSSY- ----HDPEDE PRAA--PLDP DYFEFDDHKE DLTKAELK--
    388025  -EFY---SIT SRRSRDYIRA LPIRKR---- ----KPFTQL F-P--KASAD ---------- ---------- --------AI DFLQKTLTFD PKKRLTVDEA LEHPYLAAY- ----HDPEDE PVVA--SMDP DYFDFDH--- DLTKAELK--
    581713  -EFY---SIT SRRSRDYIRA LPIRKR---- ----KSFTML F-P--KASPE ---------- ---------- --------AI DFLQKTLTFD QRKRMSVDEA LEHPYLATY- ----HDPEDE PVVA--SLDP DYFDFDHYKD DLTKAELK--
    229317  -EFY---SIT SRRSRDYIRA LPIRKR---- ----KSFTTL F-P--RASQE ---------- ---------- --------AI DFLQKTLTFD QRKRMTVDEA LEHPYLATY- ----HDPEDE PVAA--SLDP DYFDFDEYKD DLTKAELK--
    829657  -EFY---TIT SRRSRDYIRA LPIRKR---- ----RTFQSL F-P--KANPE ---------- ---------- --------AI DFLQRTLTFD PKKRLTVDEA LNHPYLAAY- ----HDPEDE PVVA--SLDP DYFDFDQFKD DLTKAELK--
     70959  -EFY---AIT SRRSRDYIRA LPIRKR---- ----RPFSAV F-P--DASPE ---------- ---------- --------AL DFLQKTLTFD PKKRLSVDQA LEHPYLSAY- ----HDPEDE PAVA--PLDP EYFEFDCGYP LYLTRSPS--
     32857  -EFY---GIT SRRSRDYIRA LPIRKR---- ----RPFSAL F-P--KASPE ---------- ---------- --------AL DFLQKTLTFD PKKRLTVDEA LEHPYLTQY- ----HDPEDE PAVA--PLDP EYFEFD--YL DLNKSQLK--
    561172  -EFY---GIT SRRSRDYIRA LPIRKR---- ----RPFAAM F-P--KASPE ---------- ---------- --------AI DFLQKTLTFD PKKRFTVDQA LDHPYLSAY- ----HDPEDE PAVP--SLDP EYFEFD--YQ ELTKDELK--
    573944  -EFY---GIT SRRSRDYIRA LPIRKR---- ----RPFTAL F-P--KASPE ---------- ---------- --------AI DFLQKTLTFD PKKRLTVDQA LDHPYLSAY- ----HDPEDE PAVP--SLDP EYFEFD--YK ELNKDELK--
    740770  -EFY---GIT SRRSRDYIRA LPIRKK---- ----RAFNAV F-P--KASPD ---------- ---------- --------AI DFLNRTLTFD PKKRMTVEES LQHPYLAAY- ----YDPSDE PTVH--SIDP DAFEFDDSRE PPSKAELK--
     16071  -EYH---SIT SRRSREYIRA LPIRKR---- ----RAFTTL F-P--KASTD ---------- ---------- --------AI DFLQKTLTFD PKKRITVDQA LQHPYLAAY- ----RDPGDE PSCA--AIDP DYFNFDTIKE DLTVEALK--
   1126092  -EYH---SIT SRRSREYIRA LPIRKR---- ----RAFTTL F-P--KASTD ---------- ---------- --------AI DFLQKTLTFD PKKRITVDQA LQHPYLAAY- ----RDPGDE PSCA--AIDP DYFNFDTIKE DLTVEALK--
    478842  -EFY---SIT SRRSRDYLRA LPIRRR---- ----KPMSQL F-P--KASPA ---------- ---------- --------AI DFLQKTLTFD PKKRLTVEEC LNHPYLEAY- ----HDPEDE PVVE--SLEP EFFDFDHKE EMSKDQLK--
     46649  -EFY---AIT SRRSRDYIRG LDIRKR---- ----RPFAAM F-P--NASPD ---------- ---------- --------AI DFLNRTLTFD PKKRMTVEEA IQHPYLALY- ----HDPDDE PTAT--SIDP DYFEFDG--- --NEKQLK--
    601377  -EYA---AIT SRRSRDYIRA LPIRKR---- ----KPFATL I-P--SASPA ---------- ---------- --------AI DFLSRTLTFD PKKRMTVDEC LRHPYLAPY- ----YDPADE PCGP--GLDE NYFYFDLQKE ELSKAQLK--
    866574  -DFY---AIS STRSREYLRA LPFRKR---- ----VNFHHV F-S--KANPQ ---------- ---------- --------AV DLMEKCLTFS PKRRITVEEA LQHPYLAPY- ----HDPQDE PTAA--PLDP SFFDFDNG-E PLGKEELK--
    521401  -DFY---AIS STRSREYLRA LPFRKR---- ----VNFHHV F-S--KANPQ ---------- ---------- --------AI DLMEKCLTFS PKRRITVEEA LQHPYLAPY- ----HDPQDE PTAA--PLDP SFFDFDNG-E PLGKEELK--
    850251  -DFY---AIS STRSREYLRA LPFRKR---- ----VNFHHV F-S--KANPQ ---------- ---------- --------AV DLMEKCLTFS PKRRITVEEA LQHPYLAPY- ----HDPQDE PTAA--PLDP SFFDFDNG-E PLGKEELK--
    832111  -DFY---AIS SPRSREYIRA LPFRRG---- ----RDFKTM F-H--GANEL ---------- ---------- --------AI DFLEKCLTFS PKRRITAAEA LRHPYFEPY- ----HDPDDE PVAD--LIDP SFFDFDNG-E PLEKEQLK--
    832110  -DFY---AIS SPRSREYIRA LPFRRG---- ----RDFKTM F-H--GANEL ---------- ---------- --------AI DFLEKCLTFS PKRRITAAEA LRHPYFEPY- ----HDPDDE PVAD--LIDP SFFDFDNG-E PLEKEQLKGE
    676666  -DFY---AIS SPRSREYIRA LPFRKG---- ----RDFGSM F-A--GANPL ---------- ---------- --------AI DFLEKCLTFS PRRRITVEEA LQHPYLEPY- ----HDPDDE PVAN--PIDP SFFDFENGDE PLEKEELK--
   1143376  -DFY---AIS SPRSREYIRA LPFRKG---- ----KDFSTM F-A--GTNPL ---------- ---------- --------AI DFLEKCLTFS PKRRITAVEA LQHPYFEPY- ----HDPDDE PIAD--PIDP SFFDFENGDE PLEKEELK--
    457401  -DFY---AIT SQRSRDYIRA LPFRKR---- ----RSFTQL F-P--GANPL ---------- ---------- --------AV DFLEKCLTFS PIRRIDVTEA LRHPYLEPY- ----HDPEDE PTAN--PIDP SFFDFDNG-D PVDKEDLK--
    834255  -DFY---AIS SQRSREYIRA LPFRKS---- ----KPFSQL F-P--AANPL ---------- ---------- --------AI DFLEKCLTFS PRRRITVVEA LQHPYFDPY- ----HDPDDE PMAD--PIDP SFFDFDNG-E ALEKEDLK--
    133042  -DFY---AIT SPRSREYIRA LPFRKK---- ----KPFSTL F-P--GANPL ---------- ---------- --------AL DLMDKCLTFS PKRRIDVDEA LKHPYLESY- ----HDPQDE PTAE--ALDP SFFDFDYG-E PLSKEQLK--
     30507  -DFY---AIT SPRSREYIRA LPFRRK---- ----KPFNTL F-P--GANTL ---------- ---------- --------AL DLMEKCLTFS PKRRIDVDEA LQHPYLQAY- ----HDPQDE PTAE--PLDP AFFDFDYG-T PLGREALK--
    646041  -DFY---AIT SQRSREYIRA LPFRKK---- ----KPFSQL F-P--GASPE ---------- ---------- --------AI DLMEKCLTFS PKRRIEVEEA LKHPYLAAY- ----HDPSDE PTAE--PLDP SFFDFDNG-D PLGKEELK--
    570964  -DFY---AIT SQRSREYIRA LPFRKK---- ----KPFSQL F-P--GANPQ ---------- ---------- --------AI DLMEKCLTFS PKRRIEVEEA LKHPYLEAY- ----HDPSDE PTAE--PLDP SFFDFDNG-D PLGKEDLK--
    301734  -DFY---AIT SQRSREYLRA LPFRKK---- ----KPFSQL F-P--NANPL ---------- ---------- --------AI DFLEKCLTFS PKRRIDVSEA LAHPYLESY- ----HDPEDE PTAP--PLDP SFFDFDGP-D SLGKEDLK--
    154378  -DFY---AIT SQRSREYIRA LPFRKK---- ----KPLDQL F-V--GANPL ---------- ---------- --------AV DLMEKCLTFS PKRRIDVSEA LEHPYLESY- ----HDPDDE PTAP--PLDP SFFDFDGS-E PLGKEDLK--
     90189  -DFY---AIS SLRSREYIRA LPFRKK---- ----KSFAQL F-P--NANPL ---------- ---------- --------AI DLLEKLLTFS PKRRIDVSEA LRHPYLQPY- ----HDPQDE PTAD--PIDP SFFDFDFG-D PMSKEDLK--
    448371  -DFY---AIT SPRSREYIRA LPFRKK---- ----KPFSQL F-P--NANPE ---------- ---------- --------AV DLLEKCLTFS PKRRIDVTEA LAHPYLKAY- ----HDPSDE PTAP--PLDP SFFDFDNG-T PLGKEELK--
    476779  -DFY---AIT SPRSREYIRA LPFRKK---- ----KPFSQL F-P--NANPE ---------- ---------- --------AV DLLEKCLTFS PKRRIDVTEA LAHPYLKAY- ----HDPSDE PTAP--PLDP SFFDFDNG-T PLGKEELK--
     68819  -DFY---AIT SQRSREYIRA LPFRKK---- ----KPFSQI F-P--KANPL ---------- ---------- --------AV DLLDKCLTFS PKTRIDVAGA LAHPYLAPY- ----HDPADE PTAE--PLDP SFFDFDTG-E PLGKERLKGA
   2671102  -DFY---AIT SQRSREYIRA LPFRKK---- ----KSFRYL F-P--DANPL ---------- ---------- --------AV DLLEQLLTFS PKRRIDVEQA LAHPYLAPY- ----HDPQDE PTAE--PLDP SFFSFDNG-E QMRKEDLKGM
    940143  -DFY---AIS SQRSREYIRA LPFRKK---- ----KPFSSL F-P--NANPL ---------- ---------- --------AI DLLEKCLTFS PKKRIQVDEA LQHPYLEPY- ----HDPMDE PTAP--PLDP SFFDFDNG-N PLSKEELKGT
    585786  -DFY---AIS SPRSREYIRV MPFRKK---- ----KPFATL F-P--KANPL ---------- ---------- --------AI DLMEKCLTFS PKKRIQVDEA LKHPYLAPY- ----HDPMDE PTAP--PLDP SFHFDNG-T PLSKEELK--
    349085  -DFY---AIS SPRSREYIRA LPFRKK---- ----KPFSSL F-P--NANPL ---------- ---------- --------AI DLLEKCLTFS PKKRIQVDEA LQHPYLEPY- ----HDPMDE PTAP--PLDP SFFDFDNG-N PLSKEELK--
   1539717  -DFY---AIT SQRSREYIRA LPFRKK---- ----KSFSTL F-P--GANPQ ---------- ---------- --------AI DLMEKCLTFS PKRRIDVHEA LAHPYLAPY- ----HDPMDE PTAA--PLDP SFFDFDNG-I QLDKEQLK--
     24638  -DFY---AIN STRSREYIRA LPFRKK---- ----RPFSQL F-P--KANPL ---------- ---------- --------AH DLLEKCLTFN PKRRIDVNDA LQHPYLASY- ----HDPNDE PVAL--PLDS SFFDFDNG-K QMDKDQLK--
     75000  -DFY---AIN STRSREYIRA LPFRKK---- ----RPFSQL F-P--KANPL ---------- ---------- --------AH DLLEKCLTFN PKRRIDVNDA LQHPYLASY- ----HDPNDE PVAL--PLDS SFFDFDNG-K QMDKDQLK--
   1245527  -DFY---AIT SPRSREYIRA LPFRKK---- ----KSFQQL F-P--GKNPL ---------- ---------- --------AI DFLEQCLTFS PKRRIQVEEA LQHPYLEPY- ----HDPADE PTAA--PLDP SFFDFDNG-V PLEKEELK--
    103018  -DFY---AIQ SHRSRDYIRA LPFNKR---- ----RPFSTI F-P--NANPL ---------- ---------- --------AI DLLDKCLTFN PKKRITVEEA LAHPYLEPY- ----HDADDE PNAD--PLSI EFFAFDNHSPT PLSREELK--
     43883  -DFY---AIG SQRSRDYLRA LPFQKK---- ----KPFQTL Y-P--NANPL ---------- ---------- --------AI DLLERCLTFN PKKRMSVVDA LSHPYLDPY- ----HDETDE PTAP--VLPP AFFSFDQQQ- -LDREQLK--
     58913  -DFY---AIS SHRSRDYLRA LPFKKK---- ----KPFSQL Y-P--NASNL ---------- ---------- --------AV DLLEKCLTFN PKKRITCEEA LKHPYLAAY- ----HDPDDE PDSP--PLDP SFHFDNGKE QLTREQLK--
     18326  -DFY---SIS SHRSREYIRA LPFRKK---- ----KPFAQL F-E--GKNPM ---------- ---------- --------AL DMLEQLLTFN PKTRMDVEGA LAHPYLEPY- ----HDPGDE PTAP--VLDP SFFDFDYQKD -ITKEQLK--
    301059  -DFY---SIS SHRSREYIRA LPFRKK---- ----KPFAQL F-E--GKNPM ---------- ---------- --------AL DMLEQLLTFN PKTRMDVEGA LAHPYLEPY- ----HDPGDE PTAP--VLDP SFFDFDYQKD -ITKEQLK--
     12410  -DFY---SIS SHRSREYIRA LPFRKK---- ----KPFAQL F-E--GKNPM ---------- ---------- --------AL DMLEQLLTFN PKTRMDVEGA LAHPYLEPY- ----HDPGDE PTAP--VLDP SFFDFDYQKD -ITKEQLK--
     18327  -DFY---SIS SHRSREYIRA LPFRKK---- ----KPFAQL F-E--GKNPM ---------- ---------- --------AL DMLEQLLTFN PKTRMDVEGA LAHPYLEPY- ----HDPGDE PTAP--YLSL KRSS-DRAV- -INKL
    184754  -DLQ---SIK SKRAREYIKS LPFYKK---- ----TPLRNL FGD--HINPL ---------- ---------- --------GV DLVEKMLAFN TVNRITVEEA LKHPYLRAF- ----HVPSDE PVAN--PIPK DFFKFDLYKD QLSISQLK--
     99455  -DYH---SIR SRRAKEYIKS LPFYKK---- ----VPFQKI L-P--YVNPL ---------- ---------- --------AI DLLSRMLTFN PSERITVVEA LEHPYLATY- ----HDPTDE PEAP--PIPQ EFFHFDKMKD QLSVSELK--
    706256  NDLR---CIE SPRAREYIKS LPMYPA---- ----APLEKM F-P--RVNPK ---------- ---------- --------GI DLLQRMLVFD PAKRITAKEA LEHPYLQTY- ----HDPNDE PEGE--PIPP SFFEFDHYKE ALTTKDLK--
    653342  NDLR---CIE SPRAREYIKS LPMYPA---- ----APLEKM F-P--RVNPK ---------- ---------- --------GI DLLQRMLVFD PAKRITAKEA LEHPYLQTY- ----HDPNDE PEGE--PIPP SFFEFDHYKE ALTTKDLK--
    462249  -EFY---AIT TRRSRDYIRA LPFRKK---- ----KPFSQI F-P--NASPL ---------- ---------- --------AV DFLTKTLTFD PKKRLTVEEA LAHPYLEAY- ----HDPEDE PVAP--PLDP EFFEFDRFRA RSARCELL--
    326973  -DYY---GIK SRRAREYIRS LPFKKK---- ----IPWKVM F-P--KTSDL ---------- ---------- --------AL DLLERLLAFN PVKRITVEEA LKHPYLEPY- ----HDPEDE PTAE--PIPE EFFDFDKNKD NLTKEQLK--
     77855  -DYY---GIK SRRAREYIRS LPFKKK---- ----IPFKHL F-P--KTNDL ---------- ---------- --------AL DLLEKLLAFN PAKRITVEEA LKHPYLEPY- ----HDPDDE PTAD--PIPE EFFDFDKNKD ALSKEQLK--
    338563  -EFY---AIT TRRSRDYIRA LPFRKR---- ----RPFAQL F-P--NANPL ---------- ---------- --------AV DFLTKTLTFD PKKRITVEDA LAHPYLEAY- ----HDPDDE PVAP--PLDP EFFEFDLHKD DISREQLK--
    233520  -EFY---AIT TRRSRDYIRA LPFRKR---- ----RPFAQL F-P--NANLL ---------- ---------- --------AV DFLTKTLTFD PKKRITVEEA LAHPYLEAY- ----HDPEDE PVAP--PLDP EFFEFDLHKD DISREQLK--
     73610  -EFY---AIT TRRSRDYIRA LPFRKR---- ----KPFATL F-P--NANPL ---------- ---------- --------AI DFLTKTLTFD PKKRITVEEA LAHPYLEAY- ----HDPEDE PVAP--PLDP EFFEFDLHKD DISREQLK--
    150924  -EFY---AIT TRRSRDYIRA LPFRKR---- ----RPFAQL F-P--NATPL ---------- ---------- --------AI DFLTKTLTFD PKKRLTVEEA LAHPYLEAY- ----HDPDDE PVAP--PLDP EFFEFDLHKD DISREQLK--
    348809  -EFY---AIT TRRSRDYIRA LPFRKK---- ----RPFSVL F-P--NANPL ---------- ---------- --------AI DFLTKTLTFD PKKRITVEDA LAHPYLEAY- ----HDPEDE PVAP--PLDP EFFEFDLHKD DISREQLK--
    913771  -EFY---AIT TRRSRDYIRA LPFRKR---- ----RPFAQL F-P--NASAL ---------- ---------- --------AV DFLTKTLTFD PKKRISVEMA LCHPYLEAY- ----HDPDDE PVAP--PLDP EFFEFDLHKD DISREQLK--
    913918  -EFY---AIT TRRSRDYIRA LPFRKR---- ----RPFAQL F-P--NASAL ---------- ---------- --------AV DFLTKTLTFD PKKRISVEMA LCHPYLEAY- ----HDPDDE PVAP--PLDP EFFEFDRTHL SSHTQPAN
    747578  -DFN---QIK SKRAKEYIAN LTMRPP---- ----LPWETV WSK--TDLNP ---------- ---------- -------DMI DLLDKMLQFN PDKRISAAEA LRHPYLAMY- ----HDPSDE PEYP--PLNL DDEFWKLDNK IMRPEEEEEV
    458977  -DFN---QIK SKRAKEYIAN LPMRPP---- ----LPWETV WSK--TDLNP ---------- ---------- -------DMI DLLDKMLQFN PDKRISAAEA LRHPYLAMY- ----HDPSDE PEYP--PLNL DDEFWKLDNK IMRPEEEEEV
     41459  -EFY---AIT SRRSRDYIRA LPFRKK---- ----RPFSTL F-P--KASPE ---------- ---------- --------AI DFLNRTLTFD PKKRATVEEA LEHPYLAAY- ----HDPEDE PTSG--TLDP EYFQFDRECL QVIAFGNGIS
   1397463  -EFY---GIT SRRSRDYIRA LPIRKR---- ----RPFTAL F-P--KASPE ---------- ---------- --------AI DFLQKTLTFD PKKRLTVDQA LEHPYLAAYA RREQHDPEDE PAVS--SLDP EYFEFDYK-- ----------
    149203  -EFY---GIT SRRSRDYIRA LPIRKR---- ----RPFTAL F-P--KASPE ---------- ---------- --------AI DFLQKTLTFD PKKRLTVDQA LDHPYLSAY- ----HDPEDE PAVP--SLDP EYFEFDCECP S---------
    102078  -DLE---CVK SPRARDYIQS LPRQRA---- ----VDVFSY FHN--HPRRI --------QR FG-------- -TATINPLGL DLMTRLLTFD PEKRITASEA LKHPYLEQY- ----HDPSDE PITK--VKLE PDYLDQKSKS DLSIDALK--
 36766:44-3  -DFH---SIK SARARSFIKS LKHQVR---- ----LDFADL CNN--HPSRL --------QR HG-------- -RRKISPQGI DLLERMLAFN PDKRISVDEA LSHPFVSRY- ----HDPNDE PTTT--PIAA EEFDFDVEKD KLDTMELK--
     54278  -DMH---IVK SHRAKNFLKS LPHREK---- ----LDFTEY INT--HPYRI --------AK HG-------- -INNVNPLGV DLLEKMLVFD PNKRITVEEA LAHPYLASY- ----HDPLDE PITS--PIPL EEFAFDINKD DLDKENLK--
     29185  -EFY---KIN SRRSRDYLRA LPFTKK---- ----RDFAEL F-P--NASPA ---------- ---------- --------AV DFLTRTLTFD PTKRLTVEQC LEHPYVSAY- ----HDPEDE PTAP--PLRQ
    790328  -DFY---AIS STRSREYLRA LPFRKR---- ----VNFHHV FSK--A---- ---------- ---------- -----NPQAV DLMEKCLTFS PKRRITVEEA LQHPYLAPY- ----HDPQDE PTAA--PLDP SFFDFD-NGE PLGKEELK--
    765763  -DFY---AIS STRSREYLRA LPFRKR---- ----VNFHHV FSK--A---- ---------- ---------- -----NPQAV DLMEKCLTFS PKRRITVEEA LQHPYLAPY- ----HDPQDE PTAA--PLDP SFFDFD-NGE PLGKEELK--
   1491990  -DFY---AIT SQRSREYIRA LPFRKK---- ----KSFSTL FPG--A---- ---------- ---------- -----NPQAI DLMEKCLTFS PKRRIDVHEA LAHPYLAPY- ----HDPMDE PTAA--PLDP SFFDFD-NGI QLDKEQLK--
     32998  -EFY---AIT TRRSRDYIRA LPFRKR---- ----KPFATL FPN--A---- ---------- ---------- -----SPLAV DFLTKSLTFD PKKRITVEEA LAHPYLEAY- ----HDPEDE PCAP--PLDP EFFEFDLHKD DISREQLK--
   2601990  -DFY---AIT SQRSREYIRA LPFRKK---- ----KSFRYL FPD--A---- ---------- ---------- -----NPLAV DLLEQLLTFS PKRRIDVEQA LAHPYLAPY- ----HDPQDE PTAE--PLDP SFFSFD-NGE QMRKEDLK--
    262460  -DYY---GIK SRRAREYIRS LPFKKK---- ----IPWRAM FPK--T---- ---------- ---------- -----SDLAL DLLDKMLAFN PVKRITVEDA LRHPYLEPY- ----HDPDDE PSAE--PIPE EFFDFDKNKD SLSKEQLR--
     11717  -DIS---FVK NTKALRFLTK LAISKP---- ----KKWRDV FAG--SGAE- ---------- ---------- --NSVSSDAM DLLSKMLMFN PEKRISVDAA LRHPYLATF- ----FDENDL ALSK--PFD- --FSFDLPDD QLSKDALI--
    532299  -DIG---FVK NTKALRFLTK LAISKP---- ----KKWHDV FAG--SGTE- ---------- ---------- --NAVSSEAI DLLSKMLLFN PEKRISVDTA LRHPYLATF- ----FDENDL VVSS--SFD- --FSFDLPDD QLSKDALI--
    551831  -DIG---FVK NTKAMRFLTK LAISKP---- ----KKWRDV FAG--SGTE- ---------- ---------- --NAVSSEAI DLLSKMLFFN PEKRISVDAA LRHPYLATF- ----FDENDL VVST--AFD- --FSFDLPDD QLSKDALI--
```

```
  301484  -DIG---FVK NTKALRFLTK LAISKP---- ----KKWHDV FAG--SGTE- ---------- ---------- --NAVSSEAI DLLSKMLLFN PEKRISVDTA LRHPYLATF- ----FDENDL VVSS--SFD- --FSFDLPDD QLSKDALI--
   42366  -TLR---RVG SPR------- ---------- ---------- ---------- ---------- ---------- ---------- -----MLCFD PAKRISCEDA LKHPYLQVW- ----HDPADE PVCE--KK-- --FDFSFEEE D-SIEGMK--
  476004  -ELY---FLT NARARKFMNA EFQKRG---- ----PNPTKP LAQ--MFTD- ---------- ---------- --S--PPDAL DLLQKMLVID PNKRISVDEA LAHPYLASI- ----RNVDDE TTAT--SS-- --FDFDFENE KLTKPVLQ--
  558476  -ELY---FLT NARARKFMNA EFQKRG---- ----PNPTKP LAH--MFTD- ---------- ---------- --S--PPDAL DLLQKMLVID PNKRISVDEA LAHPYLASI- ----RNMDDE TMAT--SS-- --FDFDFENE KLTKPVLQ--
    9954  -DLH---FVV SERAKRFMKN QPMRPG---- ----VPFSKL FPK--A---- ---------- ---------- -----TPEAV NLLQRMLVFD PVKRISVEEA LEHPYLASL- ----HNLEDE PVAD--SI-- --FSFDFEKE DLTESRLK--
   82136  -DLH---FVV SERAKRFMKN QPLRPG---- ----VPFARL FPK--A---- ---------- ---------- -----TPEAV DLLQRMLVFD PAKRISVEEA LEHPYLASL- ----HNLEDE PVAD--SF-- --FSFDFEKE DLTESRLK--
  312415  -DLH---FVV SERAKRFMKN QPMRPG---- ----VPFAKL FPK--A---- ---------- ---------- -----TPEAL DLLQRMLVFD PAKRISVEEA LEHPYLASL- ----HNLEDE PVAD--SS-- --FSFDFEKE DLTESRLK--
  354114  -SFKENPLAN KLKGRQLLSR TQAVAG---- ----IDTSTM FPN--A---- ---------- ---------- -----NPEGL DLLWKMLVFD VEKRITVEEA LRHPYLAMY- ----YDEERE NAPV--EK-- --FQ-SFDLD DLDESDLK--
   34134
   20005  EMAF----VP HEAAKRAILQ HGFYPK---- ----RPLIEF F-P--DANPL ---------- ---------- --------AV DLLSQMLKFN PAERISIVQA LAHPYLAQL- ----QNPADE PVC---AEPF NFDFERESLD -LGVEMPK--
  518921  EMSF----VP HEAAKRAILQ HGFYPK---- ----RPLIEF F-P--DANPL ---------- ---------- --------AV DLLSQMLKFN PAERISVVQA LAHPYLAQL- ----QNPADE PVC---AEPF NFDFERESLD -LGVEMPK--
  503206  EMSF----VP HEAAKRAILQ HGFYPK---- ----RPLIEF F-P--DANPL ---------- ---------- --------AV DLLSQMLKFN PAERISVVQA LAHPYLAQL- ----QNPADE PVC---AEPF NFDFERESLD -LGVEMPK--
   10810  EMAF----VP HEAAKRAILQ HGFYPK---- ----RPLIEF F-P--DANPL ---------- ---------- --------AV DLLSQMLKFN PAERISIVQA LAHPYLAQL- ----QNPADE PVC---AEPF NFDFERESLD -LGVEMPK--
  AtMPK1  DLEF----ID NPKAKRYIRS LPYSPG---- ----MSLSRL Y-P--GAHVL ---------- ---------- --------AI DLLQKMLVFD PSKRISVSEA LQHPYMAPL- ----YDPNAN PPA---QVPI D-LDVDED-- -LREEMIR--
  AtMPK2  DLEF----ID NPKAKRYIES LPYSPG---- ----ISFSRL Y-P--GANVL ---------- ---------- --------AI DLLQKMLVLD PSKRISVTEA LQHPYMAPL- ----YDPSAN PPA---QVPI D-LDVDEDED -LGAEMIR--
  OsMPK7  DIEF----ID NPKARKYIKT LPYTPG---- ----IPLTSM Y-P--QAHPL ---------- ---------- --------AI DLLQKMLVFD PSKRISVTEA LEHPYMSPL- ----YDPSAN PPA---QVPI D-LDIDEN-- -LGVDMIR--
 OsMPK14  DLEF----ID NPKARRYIKS LPYTPG---- ----VPLASM Y-P--HAHPL ---------- ---------- --------AI DLLQKMLIFD PTKRISVTEA LEHPYMSPL- ----YDPSAN PPA---QVPI D-LDIDEN-- -ISADMIR--
 AtMPK14  DLQF----ID NQKARRFIKS LPFSKG---- ----THFSHI Y-P--HANPL ---------- ---------- --------AI DLLQRMLVFD PTKRISVSDA LLHPYMEGL- ----LEPECN PSE---NVPV SSLEIDEN-- -MEGDMIR--
  AtMPK7  DIRF----ID NPKARRFIKS LPYSRG---- ----THLSNL Y-P--QANPL ---------- ---------- --------AI DLLQRMLVFD PTKRISVTDA LLHPYMAGL- ----FDPGSN PPA---HVPI S-LDIDEN-- -MEEPVIR--
 AtMPK12  SLGF----LR SDNARRYVRQ LPRYPK---- ----QQFAAR F-P--KMPTT ---------- ---------- --------AI DLLERMLVFD PNRRISVDEA LGHAYLSPH- ----HDVAKE PVC---STPF S-FDFEHPS- -CTEEHIK--
  AtMPK4  SLGF----LR SDNARRYVRQ LPQYPR---- ----QNFAAR F-P--NMSAG ---------- ---------- --------AV DLLEKMLVFD PSRRITVDEA LCHPYLAPL- ----HDINEE PVC---VRPF N-FDFEQPT- -LTEENIK--
 OsMPK4-1  SLGF----LR SDNARRYMKQ LPQYPR---- ----QDFRLR F-R--NMSAG ---------- ---------- --------AV DLLEKMLVFD PSRRITVDEA LHHPYLASL- ----HDINEE PTC---PAPF S-FDFEQPS- -FTEEHIK--
OsMPK16-2  SLGF----LR SDNARRYVRS LPQYPK---- ----QQFRAR F-P--TMSSG ---------- ---------- --------AM DLLERMLVFD PSKRITVDEA LCHPYLASL- ----HEIYDE PVC---PAPF S-FDFEQPS- -LTEEDIK--
  AtMPK5  SLEF----LR SANARKYVKE LPKFPR---- ----QNFSAR F-P--SMNST ---------- ---------- --------AI DLLEKMLVFD PVKRITVEEA LCYPYLSAL- ----HDLNDE PVC---SNHF S-FHFEDPS- -STEEEIK--
 AtMPK10  ELGS----L- SEYAKRYIRQ LPTLPR---- ----QSFTEK F-P--NVPPL ---------- ---------- --------AI DLVEKMLTFD PKQRISVKEA LAHPYLSSF- ----HDITDE PEC---SEPF N-FDLDEHP- -SEEQFR---
  AtMPK6  ELEF----L- NENAKRYIRQ LPPYPR---- ----QSITDK F-P--TVHPL ---------- ---------- --------AI DLIEKMLTFD PRRRITVLDA LAHPYLNSL- ----HDISDE PEC---TIPF N-FDFENHA- -LSEEQMK--
  OsMPK6  DLDF----V- NENARRYIRQ LPRHAR---- ----QSFPEK F-P--HVHPL ---------- ---------- --------AI DLVEKMLTFD PRQRITVEGA LAHPYLASL- ----HDISDE PVC---SSPF S-FDFEQHA- -LSEEQMK--
  AtMPK3  DLGF----TH NEDAKRYIRQ LPNFPR---- ----QPLAKL F-S--HVNPM ---------- ---------- --------AI DLVDRMLTFD PNRRITVEQA LNHQYLAKL- ----HDPNDE PIC---QKPF S-FEFEQQP- -LDEEQIK--
  OsMPK3  ELGF----IR NEDARKYMRH LPQYPR---- ----RTFASM F-P--RVQPA ---------- ---------- --------AL DLIERMLTFN PLQRITVEEA LDHPYLERL- ----HDIADE PIC---LEPF S-FDFEQKA- -LNEDQMK--
  206269  VIA----TIC SENTLRFVKN LPKRDP---- ----IPFSQR FAG---QDPE ---------- ---------- --------AI DLLDKMLTFD PRKRISAMEA LEHPYLSPY- ----HDPSDE PAAAD-KFDW SFN--EAELP VDTWKVMM--
  154265  LVA----TIC SENTLRFVKN LPKREP---- ----IPFSQR FAG---QDPQ ---------- ---------- --------AI DLLEKMLTFD PRQRITAEEA LSHPYLAPY- ----HDPTDE PSAPE-KFDW SFN--EADLP IDTWKVMM--
  168032  VIS----TIC SANTLRFVNN LPKREP---- ----VPFSQR FAG---QDPE ---------- ---------- --------AI DLLEKMLMFD PRKRITAEEA LSHPYLAPY- ----HDPSDE PAAPE-KFDW SFN--EADLP VDTWKVMM--
  307550  VIA----TIC SENTLRFVKN LPKRDP---- ----VPFSQR FVG---QDPQ ---------- ---------- --------AI DLLEKMLTFD PAKRITAEEA LSHPYLAPY- ----HDPTDE PVSPE-KFDW SFN--EADLP VDTWKVMM--
  320251  VIQ----TIC SENTLRFVQS LPKRER---- ----QPLKNK FKN---ADPQ ---------- ---------- --------AI DLLERMLVFD PRKRVRAGDA LADPYLAPY- ----HDPTDE PVADE-KFDW SFN--DADLP VDTWKIMM--
  356517  VIN----TIA SENTLRFVKS LPKRER---- ----QPLKNK FRN---ADPG ---------- ---------- --------AI DMLERMLVFD PKKRITATEA LAHEYLAPY- ----HDPTDE PVAEE-KFDW SFN--DADLP VDTWKIMIPD
  113434  VIE----TIA SENTLRFVQS LPKRER---- ----VPFSEK LRC---NDES ---------- ---------- --------AL DLLEKMLVFD PRKRIDATES LAHEYVAPY- ----HDPTDE PVANE-KFDW SFN--DAELP IDTWKVMM--
   28887  VIQ----TIC SENTLRFVQS LPKRER---- ----IPFAQK FRG---ADPL ---------- ---------- --------AL DLLEKMLVFD PRKRIDAGQA LAHEYLAPY- ----HDPSDE PVAPE-AFDW SFN--DADLP VDTWKIMM--
   16635  VIQ----TIC SENTLRFVQS LPKRER---- ----VPFSQK FRN---ADPL ---------- ---------- --------AI DLLEKMLVFD PRKRISAAEA LAHEYLAPY- ----HDPTDE PAAEQ-KFDW SFN--DADLP VDTWKVMM--
    6923  VIA----TIC SENTLRFVQS LPKRER---- ----VPFTQK FKT---NDPS ---------- ---------- --------AL DLLEKMLVFD PRKRITATEC LAEEYVAPY- ----HDPSDE PDAKE-KFDW SFN--DADLP VDTWKVMM--
  807502  VIE----TIC SENTLRFVQS LPKRER---- ----QPFSQK LRT---SDPD ---------- ---------- --------AL DLLERMLVFD PRKRIDAAES LAHPYVAPY- ----HDPTDE PVAAE-KFDW SFN--DADLP VDTWKVMM--
  129058  VIE----TIA SENTLRFVQS LPKRER---- ----VPFSQK LRT---TDKD GMSR------ ---------- --------SL DLLEEMLVFD PRKRIDAATS LAHDYVAPY- ----HDPTDE PVAAE-QFDW SFN--DADLP VDTWKVMM--
  359241  VIE----TIC SENTLRFVQS LPKRER---- ----VPFSQK LRT---KDED ---------- ---------- --------AL DLLEKMLVFD PRKRIDATES LAHEYVAPY- ----HDPTDE PVAVE-KFDW SFN--DADLP VDTWKVMM--
   53282  VIK----TIC SENTLRFVQS LPKRER---- ----VPFSQK LRT---TDPD ---------- ---------- --------AL DLLEKMLVFD PRKRIDATES LGHTYLAPY- ----HDPTDE PIAEL-KFDW SFN--DADLP VDTWKVMM--
  117789  VIQ----TIA SENTLRFVQS LPKRER---- ----VPLNQK LRC---NDPD ---------- ---------- --------AL DLLDKMLVFD PRKRITAAES LAHEYVAPY- ----HDPSDE PEASE-KFDW SFN--DADLP VDTWKVMM--
  170586  VIE----TIA SENTLRFVQS LPKRER---- ----VPFSEK LRC---NDES ---------- ---------- --------AL DLLEKMLVFD PRKRIDATES LAHEYVAPY- ----HDPTDE PVANE-KFDW SFN--DAELP IDTWKVMM--
  162119  VIQ----TIA SENTLRFVQS LPKRER---- ----VPFSQK LHS---NDPV ---------- ---------- --------AL DLLEKMLVFD PRKRITATES LEHEYVAPY- ----HDPTDE PEAAE-IFDW SFN--DADLP VDTWKVMM--
  560244  VIQ----TIA SENTLRFVQS LPKRER---- ----VPFSEK LSC---NDPV ---------- ---------- --------AI DLLEKMLVFD PRKRITATES LGHEYVAPY- ----HDPTDE PEAAE-KFDW SFN--DADLP VDTWKVMM--
  499279  VIQ----TIA SENTLRFVQS LPKRER---- ----VPFSEK LHC---NDPV ---------- ---------- --------AI DLLEKMLVFD PRKRITATES LAHEYVAPY- ----HDPTDE PEAAE-KFDW SFN--DADLP VDTWKVMM--
  898207  VIA----TIA SENTLRFVKS LPKRER---- ----VPFKQK LRC---ADES ---------- ---------- --------AL DLLEKMLVFD PRKRITATES LAHEYVAPY- ----HDPTDE PEAAE-KFDW SFN--DADLP VDTWKVMM--
  500359  VIA----TIA SENTLRFVQS LPKRDR---- ----QPFSQK LRT---ADDS ---------- ---------- --------AL DLLEKMLVFD PRKRITATEA LAHDYVSPY- ----HDATDE PESSE-KFDW SFN--DADLP VDTWKVMM--
  390416  VIQ----TIC SENTLRFVQS LPKRER---- ----VPFSQK LRT---ADHD ---------- ---------- --------AL DLLEKMLVFD PRKRITATES LSHEYVAPY- ----HDPTDE PLAAE-KFDW SFN--DADLS VDTWKVMM--
   61586  VIE----TIC SENTLRFVQS LPKRER---- ----VPFSQK LRT---TDPD ---------- ---------- --------AL DLLEKMLVFD PRKRINATEC LEHEYVAPY- ----HDPTDE PVAAE-KFDW SFN--DADLP VDTWKVMM--
   74488  VIK----TIC SENTLRFVQS LPKRER---- ----IPFNER FKT---QDPV ---------- ---------- --------AL DLLEKMLVFD PRQRIDATQA LGHEYVSPY- ----HDPTDE PVAPQ-AFDW SFN--DADLP VDTWKVMM--
  155373  VIK----TIG SENTLRFVQS LPKREK---- ----IPLTTK FPN---SDPV ---------- ---------- --------AL DLLEKMLVFD PKKRITAGDA LYHKYLEPY- ----HDPDDE PVAAE-VFDW SFN--DADLP VDTWKVMM--
   56185  VIQ----TIC SENTLRFVQS LPKRER---- ----VPFSQK FRG---ADPQ ---------- ---------- --------AL DLLEKMLVFD PRKRIDATQA LAHEYLAPY- ----HDPTDE PVAPE-AFDW SFN--DADLP VDTWKVMM--
   14909  VIQ----TIC SENTLRFVQS LPKRER---- ----IPFAQK FRG---ADPL ---------- ---------- --------AL DLLEKMLVFD PRKRIDAGQA LAHEYLAPY- ----HDPSDE PVAPE-AFDW SFN--DADLP VDTWKIMM--
  176707  VIA----TIC SENTLRFVQS LPKRER---- ----VPFTQK FKT---NDPS ---------- ---------- --------AL DLLEKMLVFD PRKRITATEC LAEEYVAPY- ----HDPSDE PDAKE-KFDW SFN--DADLP VDTWKVMM--
  176721  VIA----TIC SENTLRFVQS LPKRER---- ----VPFTQK FKT---NDPS ---------- ---------- --------AL DLLEKMLVFD PRKRITATEC LAEEYVAPY- ----HDPSDE PDAKE-KFDW SFN--DADLP VDTWKVMM--
  240717  VIA----TIC SENTLRFVQS LPKRER---- ----VPFTQK FKT---NDPS ---------- ---------- --------AI DLLEKMLVFD PRKRITATEC LAEEYVAPY- ----HDPSDE PDAKE-KFDW SFN--DADLP VDTWKVMM--
   56428  VIH----TIC SENTLRFVKS LPKRQR---- ----IPLSQK FRG---ADPS ---------- ---------- --------AI DLLEKMLVFD PRKRITAAEA LAHEYLAPY- ----HDPSDE PVAEE-KFDW SFNVLDADLP VDSWKVMMY-
   97915  VIH----TIC SENTLRFVKS LPKRQR---- ----IPLSQK FRG---ADPS ---------- ---------- --------AI DLLEKMLVFD PRKRITAAEA LAHEYLAPY- ----HDPSDE PVAEE-KFDW SFNVLDADLP VDSWKVMMY-
  707387  VIQ----TIC SENTLRFVQS LPKRER---- ----QPLVNK FKN---AEPA ---------- ---------- --------AI DLLENMLVFD PRKRVRAEQA LAHQYLAPY- ----HDPTDE PVAEE-KFDW SFN--DADLP VDTWKIMMY-
  504013  VIQ----TIC SENTLRFVQS LPKRER---- ----QPLVNK FKN---ADPA ---------- ---------- --------AI DLLENMLVFD PRKRVRAESA LAHPYLAPY- ----HDPTDE PVADE-KFDW SFN--DADLP VDTWKIMMY-
   37843  VIQ----TIC SENTLRFVQS LPKRER---- ----QPLANK FKN---ADPM ---------- ---------- --------AI DLLENMLVFD PKKRVRAGDA LAHPYLAPY- ----HDPSDE PVAEE-KFDW SFN--DADLP VDTWKIMMY-
  507853  VIQ----TIC SENTLRFVQS LPKRER---- ----QPLSNK FKN---ADPA ---------- ---------- --------AV DLLEKMLVFD PRKRIRAGDA LAHPYLSPY- ----HDPTDE PVAEE-KFDW SFN--DADLP VDTWKIMMY-
  338747  VIE----TIC SENTLRFVQS LPKRER---- ----IPLVNK FKN---AEPA ---------- ---------- --------AI DLLESMLVFD PRKRVKATEA LAHALYLAPY- ----HDPTDE PVADE-KFDW SFN--DADLP VDTWKIMMY-
   49060  VIS----TIC SENTLRFVQS LPKRER---- ----QPLKNK FKN---ADPQ ---------- ---------- --------AI ELLERMLVFD PRKRVKAGEA LADPYLSPY- ----HDPTDE PEAEE-KFDW SFN--DADLP VDTWKIMMY-
  147551  VIS----TIC SENTLRFVQS LPKRER---- ----QPLKNK FKN---ADPQ ---------- ---------- --------AI ELLERMLVFD PRKRVKAGEA LADPYLAPY- ----HDPTDE PEAEE-KFDW SFN--DADLP VDTWKIMMY-
  286873  VIQ----TIC SENTLRFVQS LPKRER---- ----QPLKNK FKN---ADPQ ---------- ---------- --------AI ELLERMLVFD PRKRVKAGEA LADQYLAPY- ----HDPTDE PVAEE-KFDW SFN--DADLP VDTWKIMMY-
  193904  VIN----TIC SENTLRFVQS LPKRER---- ----QPLKKK FKN---ADPQ ---------- ---------- --------AI ELLERILVFD PRKRIKAGEA LADPYLAPY- ----HDPTDE PEAEE-KFDW SFN--DADLP VDTWKIMMY-
  189288  VIQ----TIC SENTLRFVQS LPKRAR---- ----QPLTNK FKN---ADPL ---------- ---------- --------AI DLLERMLVFD PKKRISAEAG LAHEYLAPY- ----HDPTDE PAAEE-KFDW SFN--DADLP VDTWKIMMY-
  198339  VIK----TIC SENTLRFVQS LPKRER---- ----QPLANK FKN---ADPS ---------- ---------- --------AV DLLERMLVFD PKKRISAADG LAHEYLAPY- ----HDPTDE PVAEE-KFDW SFN--DADLP VDTWKIMMY-
 2140409  VIN----TIA SENTLRFVKS LPRRER---- ----QPLKNK FKN---ADPP ---------- ---------- --------AI DLLERMLVFD PKKRITAAEA LAHEYLAPY- ----HDPTDE PVAEE-KFDW SFN--DADLP VDTWKIMMY-
  642177  VIN----TIA SENTLRFVKS LPRRER---- ----QPLKNK FKN---ADPP ---------- ---------- --------AI DLLERMLVFD PKKRITAAEA LAHEYLAPY- ----HDPTDE PVAEE-KFDW SFN--DADLP VDTWKIMMY-
  498471  VIN----TIA SENTLRFVKS LPRRER---- ----QPLKNK FRN---ADAQ ---------- ---------- --------AI DLLERMLVFD PKKRVTAAEA LAHEYLAPY- ----HDPTDE PIAEE-KFDW SFN--DADLP VDTWKIMMY-
  784216  VIN----TIA SENTLRFVRS LPKRER---- ----QPLKNK FRN---ADPP ---------- ---------- --------AI DLLERMLVFD PRKRITAAEA LAHEYLAPY- ----HDPTDE PVAEE-KFDW SFN--DADLP VDTWKIMMY-
  128268  VIN----TIA SENTLRFVKS LPKRER---- ----QPLKNK FKN---ADSS ---------- ---------- --------AV DLLERMLVFD PKKRITATEA LSHEYLAPY- ----HDPTDE PVAEE-KFDW SFN--DADLP VDTWKIMMY-
  246689  VIN----TIA SENTLRFVKS LPKRER---- ----QPLKNK FRN---ADPS ---------- ---------- --------AI DMLERMLVFD PKKRITATEA LAHEYLAPY- ----HDPTDE PVAEE-KFDW SFN--DADLP VDTWKIMMY-
 1081594  VIN----TIA SENTLRFVKS LPKRER---- ----QPLRNK FKN---ADDS ---------- ---------- --------AV DLLERMLVFD PKKRITATEA LSHEYLAPY- ----HDPTDE PVAEE-KFDW SFN--DADLP VDTWKIMMY-
  603877  VIH----TIA SENTLRFVKS LPKRER---- ----QPLKDK FRN---ADPA ---------- ---------- --------AI DLLEDMLVFD PKKRVKATDA LAHEYLSPY- ----HDPTDE PSAEE-KFDW SFN--DADLP VDTWKIMMY-
  140829  VIH----TIA SENTLRFVQS LPKRER---- ----LPLKDR FKN---ADPL ---------- ---------- --------AI DLLERMLVFD PRKRVKASEA LAHEYLSPY- ----HDPTDE PVADE-KFDW SFN--DADLP VDTWKIMMY-
  327107  VIQ----TIC SENTLRFVKS LPQRER---- ----QPLANK FKN---ADAD ---------- ---------- --------AV DLLERMLVFN PKQRIQATDA LAHEYLAPY- ----HDPTDE PVAQE-KFDW SFN--DADLP VDTWKIMMY-
  329694  VIQ----TIC SENTLRFVQS LPQRER---- ----QPLANK FKN---ADAD ---------- ---------- --------AV DLLERMLVFD PKQRIQAPDA LAHEYLAPY- ----HDPTDE PVAQE-KFDW SFN--DADLP VDTWKIMMY-
  393439  VIQ----TIC SENTLRFVKS LPKRER---- ----QPLANK FKN---ADAD ---------- ---------- --------AV DLLEKMLVFN PKQRIQAPDA LAHEYLAPY- ----HDPTDE PVAQE-KFDW SFN--DADLP VDTWKIMMYV
   38942  VIQ----TIC SENTLRFVQS LPKRER---- ----QPLANK FKN---ADPT ---------- ---------- --------AV DLLERMLVFD PRKRVSAADA LAHEYLAPY- ----HDPTDE PVAEE-KFDW SFN--DADLP VDTWKIMVY-
   87588  VIQ----TIC SENTLRFVQS LPKRER---- ----VPFSQK FRN---ADPL ---------- ---------- --------AI DLLEKMLVFD PRKRISAAEA LAHEYLAPY- ----HDPTDE PAAEQ-KFDW SFN--DADLP VDTWKVMMY-
  443298  VIN----TIA SENTLRFVKS LPKRER---- ----QPLATK FKN---ADES ---------- ---------- --------AV DLLERMLVFD PRKRITAADA LAHEYLAPY- ----HDPTDE PVAEE-KFDW SFN--DADLP VDTWKIMMY-
   53587  VIS----TIC SENTLRFVQS LPKRER---- ----QPLKNK FKN---ADPQ ---------- ---------- --------AI ELLERMLVFD PQKRVKAGEA LADPYLAPY- ----HDPTDE PEAEE-KFDW SFN--DADLP VDTWKIMIF-
   70072  VIQ----TIC SENTLRFVQS LPKRER---- ----IPFSHK FRN---ADPL ---------- ---------- --------AL DLLERMLVFD PKKRITAAQA LAHEYLAPY- ----HDPTDE PIAEE-AFDW SFN--DADLP VDSWKVMMY-
   13822  VIH----TIC SENTLRFVQS LPKRDR---- ----VPLSQK FKT---ADPL ---------- ---------- --------AI DLLERMLVFD PRKRITAADA LAHGYLAPY- ----HDPTDE PVAEL-KFDW SFN--DADLP TDTWKVMMY-
   25364  VIQ----TIC SENTLRFVQS LPKREK---- ----VPFSQK FMN---AEPE ---------- ---------- --------AI DLLERMLVFD PRKRITAEEA LAHKYLEPY- ----HDESDE PIADE-PFDW SFN--DADLP VDEWKVKMY-
  116576  VIK----TIC SENTLRFVQS LPKRER---- ----QPLANK FKN---ADPS ---------- ---------- --------AV DLLERMLVFD PKKRISAADG LAHEYLAPY- ----HDPTDE PVAEE-KFDW SFN--DADLP VDTWKIMMY-
  103311  VIE----TIC SENTLRFVQS LPQRDP---- ----IPFGDR FASCTHVEPE ---------- ---------- --------AI DLLSKMLVFD PKKRITAADA LQHPYMEPY- ----HDPTDE PACES-KFDW SFN--DADLP VDTWRVMMY-
   26173  VID----TIC SENTLRFVQS LPHRDP---- ----IPFNER FAQCTHVEPE ---------- ---------- --------AI DLLAKMLIFD PKKRISAASA LTHPYMEPY- ----HDPTDE PICET-KFDW SFN--DADLP VDTWRVMMY-
   55352  VID----TIA SENTLRFVRS LPHREP---- ----IPFGER FASCTHVEPE ---------- ---------- --------AI DLLAKMLVFD PKKRISAAQA LTHPYMEPY- ----HDPTDE PICET-KFDW SFN--DAELP VDTWRVMMY-
  125207  VIN----TIC SENTLKFVTS LPHRDP---- ----IPFSER FKT---VEPD ---------- ---------- --------AV DLLEKMLVFD PKKRITAADA LAHPYLAPY- ----HDPTDE PVADA-KFDW HFN--DADLP VDTWRVMMY-
   67307  VIN----TIC SENTLKFVTS LPHRDP---- ----IPFSER FKT---VEPD ---------- ---------- --------AV DLLEKMLVFD PKKRITAADA LAHPYLAPY- ----HDPTDE PVADA-KFDW HFN--DADLP VDTWRVMMY-
  185764  VID----TIC SENTLRFVQS LPKREP---- ----IPLTER FKD---VEPD ---------- ---------- --------AV DLLSKMLVFD PRKRITAADA LAHPYLAPY- ----HDPTDE PVASE-KFDW SFN--DADLP IDTWRVMMY-
   99366  VID----TIA SENTLRFVKS LPKRDP---- ----IPLTER FKN---VEPD ---------- ---------- --------AV DLLEKMLVFD PKKRITAADV LGHPYLAPY- ----HDPTDE PVAEE-KFDW SFN--DADLP IDTWRVMMY-
   50111  VIQ----TIC SENTLRFVQS LPKRAK---- ----VPFSEK FKT---TDPL ---------- ---------- --------AL DLVEKMLSFD PRSRITASQA LSHPYLAPY- ----HDPNDE PVAAE-QFDW SFN--DADLP IDTWKVMMY-
   87970  VIA----TIC SENTLRFVQS LPKRER---- ----IPFHLK FKN---ADPL ---------- ---------- --------AL DLLERMLVFD PVKRITAAEA LGNEYLAPY- ----HDPTDE PVADE-PFDW SFN--DADLP VDTWRVMMY-
  107532  VIA----TIC SENTLRFVQS LPKRER---- ----IPFHLK FKN---ADPL ---------- ---------- --------AL DLLERMLVFD PVKRITAAEA LGNEYLAPY- ----HDPTDE PVADE-PFDW SFN--DADLP VDTWRVMMY-
  604754  VIA----TIA SENTLRFVQS LPKRDR---- ----VPFSQK LRT---ADDS ---------- ---------- --------AL DLLEKMLVFD PRKRITATEA LAHDYVSPY- ----HDATDE PESSE-KFDW SFN--DADLP VDTWKVMMY-
  633130  VIA----TIA SENTLRFVQS LPKRDR---- ----VPFSQK LRT---ADDS ---------- ---------- --------AL DLLEKMLVFD PRKRITATEA LAHDYVSPY- ----HDATDE PESSE-KFDW SFN--DADLP VDTWKVMMY-
  181927  VIY----SIA NKSTLKFVQS LPKHKR---- ----QALKNK FKG---ADPL ---------- ---------- --------AV DLLEQMLVFD PRKRICSSKA LTHKYLHSY- ----HEPLDE PVARG-KFDW SFS--DTNLP IDTWKIMI--
   69161  VIN----TIT SENTRRFVDA LPKREK---- ----IPFQQR FPN---ASEE ---------- ---------- --------EI DLLEKMLDFN PKERITAADA IQHPYLAPY- ----HDPSDE PVANE-RFDW SFN--DADLP VDQWKVMMY-
   35549  VIK----TIC SETTLRFVKS LPKREP---- ----QPLANK FKN---ADPD ---------- ---------- --------AI DLIERMLLFN PKKRISATEA LAHQYLAPY- ----HDPTDE PEAES-KFDW SFN--DADLP VDTWKIMMY-
  230139  VIQ----TIC SENTLRFVKS LPQRER---- ----QPLANK FKN---ADAD ---------- ---------- --------AV DLLERMLVFN PKQRIQATDA LAHEYLAPY- ----HDPTDE PVAQE-KFDW SFN--DADLP VDTWKIMMY-
  193325  VIQ----TIC SENTLRFVKS LPQRER---- ----QPLANK FKN---ADAD ---------- ---------- --------AV DLLERMLVFN PKQRIQAPDA LAHEYLAPY- ----HDPTDE PVAQE-KFDW SFN--DADLP VDTWKIMMY-
  430049  VIQ----TIC SENTLRFVQS LPKRER---- ----QNLANK FRN---ADAD ---------- ---------- --------AV DLLERMLVFD PKKRIRADEA LAHPYLAPY- ----HDPTDE PVASE-KFDW SFN--DADLP VDTWKIMMY-
  479652  VIQ----TIC SENTLRFVKS LPKRER---- ----QNLANK FRN---ADAD GMRHFGIPDL PQVPPIFTST KSLTSLFVAV DLLERMLVFD PKKRIRADEA LAHPYLAPY- ----HDPTDE PVASE-KFDW SFN--DADLP VDTWKIMMY-
  986652  VIK----TIC SENTLRFVQS LPKRER---- ----QPLANK FRN---ADAQ ---------- ---------- --------AV DLLERMLVFD PRKRVKAGEG LAHPYLAPY- ----HDPTDE PVADE-KFDW SFN--DADLP VDTWKIMMY-
  295607  VIQ----TIC SENTLRFVQS LPKRER---- ----QPLANK FKN---AEPA ---------- ---------- --------AI DLLEKMLVFD PKKRVRAEEA LAHEYLAPY- ----HDPTDE PVAEE-KFDW SFN--DADLP VDTWKIMMY-
  114099  VIQ----TIC SENTLRFVQS LPKRER---- ----QPLKNK FKN---ADPQ ---------- ---------- --------AI ELLEKMLVFD PRKRVKAGEA LADQYLAPY- ----HDPTDE PVAEE-KFDW SFN--DADLP VDTWKIMMY-
  265170  VVA----TIC SENTLRFVQN LPKRER---- ----IPFSQR FAE---QEPQ ---------- ---------- --------AI DLLEKMLVFD PRKRITATEA LAHPYLSPY- ----HDPSDE PAAPE-KFDW SFN--EADLP IDTWKVMMY-
   41804  VID----TIC SENTLRFVRS LPHRDP---- ----IPLSQK FKN---ADPE ---------- ---------- --------AI DLLEKMLVFD PKKRITATQA LSHPYSAPY- ----HDPEDE PFAAE-KFDW SFN--DADLP VDTWKVMMY-
  500846  VIE----TIA SENTLRFVQS LPKRER---- ----VPFSQK LRT---TDED ---------- ---------- --------AI DLLEKMLVFD PRKRIDAAMS LAHDYVAPY- ----HDPTDE PVAAE-QFDW SFN--DADLP VDTWKVMMY-
  341622  VIQ----TIC NENTLRFVQS LPKRER---- ----QPLVNK FKN---AESA ---------- ---------- --------AI DLLQNMLVFD PKKRVRAEQA LAHLYLAPY- ----HDPTDE PVAEE-KFDW SFD--DADFP VDTWKIIMY-
 1545705  VIQ----TIA SENTLRFVQS LPKRER---- ----VPFSEK LHC---NDPV ---------- ---------- --------AI DLLEKMLVFD PRKRITATES LAHEYVAPY- ----HDPTDE PEAAE-KF
   15276  VIQ----TIA SENTLRFVQS LPKRER---- ----VPFSEK LHC---NDPV ---------- ---------- --------AI DLLEKMLVFD PRKRITATES LAHEYVAPY- ----HDPTDE PEAAE-KF
  391543  VIA----NIT SENTLNFIKS LPKRER---- ----KPMSQL IPK---ANPD ---------- ---------- --------AV ALIDKMLQFD PNIRITAVQA VESPYLSPY- ----HDPNDE PVATE-TFDW SFL--EANLP ADIWKTIMY-
  346451  VIA----NIT SENTLNFIKS LPKRER---- ----KPMSQL IPK---ANPD ---------- ---------- --------AV ALIDKMLQFD PNIRITAVQA VESPYLSPY- ----HDPNDE PVATE-TFDW SFL--EANLP ADIWKTIMY-
  449365  VVA----NVT SQNTLNFVKS LPKRSR---- ----KSWATI IPT---ASRR ---------- ---------- --------AI NLLDKLLQFD PGKRCSAVDA LESQYLAPY- ----HDPEDE PAAPQ-SFDW SFL--EADLP ADVWKIIIY-
   54661  VIQ----TIC SEN------S LPKRER---- ----QPLANK FKN---ADPD ---------- ---------- --------AV DLLERMLVFD PRKRVSAADA LAHEYLAPY- ----HDPTDE PVAEE-KFDW SFN--DADLP VDTWKIMVY-
  275309  VMK----RIF SETLKRFVNS LPDTEP---- ----RGLASV VGD---ADPQ ---------- ---------- --------AM DLLGKMLQID PYKRITTEEA LKHPYVLTY- ----QDSDDE PVCKK-QLDW SLL--DSELS ATEWKNSMY-
  389883  VME----RVY SKTTLQFVQS LPETER---- ----RPLKSI LGD---ADPQ ---------- ---------- --------AV DLLEKMLDID PEKRITTTEA LAHPYVSTY- ----YDPEDE PVFDR-QLDW SLL--DSELS SEEWKTKM
   61624  VIC----TIN NQSTLKFIQS LPKRTK---- ----QPLGEK FKD---VELL ---------- ---------- --------AT DLMEEMLHFD TRRRIKASEA LSHKYLSQY- ----HDPTDE PVAVK-MFDW PFD--DSELT IDEWKFIM
  173235  VIC----TIN NQSTLKFIQS LPKRTK---- ----QPLGEK FKD---VELL ---------- ---------- --------AT DLMEEMLHFD TRRRIKASEA LSHKYLSQY- ----HDPTDE PVAVK-MFDW PFD--DSELT IDEWKFIMY-
   35612  VIK----TIC SETTLRFVKS LPKREP---- ----QPLANK FKN---ADPD ---------- ---------- --------AI DLIERMLLFN PKKRISATEA LAHQYLAPY- ----HDPTDE PEAES-KFDW SFN--DADLP VDTWKIMMY-
   35597  VIK----TIC SETTLRFVKS LPKREP---- ----QPLANK FKN---ADPD ---------- ---------- --------AI DLIERMLLFN PKKRISATEA LAHQYLAPY- ----HDPTDE PEAES-KFDW SFN--DADLP VDTWKIMMY-
  391476  VLQ----TIP SQNTSKYISS LPPRTK---- ----QSLAGV LKN---ASPD ---------- ---------- --------AI DLLEKLLVWD PNERLGAAAA LEHPYLQLY- ----HDATDE PVAKE-KFDW SSV--SKDRS LDHWKILTK-
  335269  VLQ----TIP SQNTSKYISS LPPRTK---- ----QSLAGV LKN---ASPD ---------- ---------- --------AI DLLEKLLVWD PNERLGAAAA LEHPYLQLY- ----HDATDE PVAKE-KFDW SSV--SKDRS LDHWKILTK-
  377162  VLQ----TIP SQNTSKYISS LPPRTK---- ----QSLAGV LKN---ASPD ---------- ---------- --------AI DLLEKLLVWD PNERLGAAAA LEHPYLQLY- ----HDATDE PVAKE-KFDW SSV--SKDRS LDHWKILTY-
```

```
 271149 VIH----TIA SENTLRFVQS LPKRER---- ----QPLKDK FRN---ADPA ---------- ---------- --------AI DLLEDMLVFD PKKRVKATDA LAHEYLSPY- ----HDPTDE PSAEE-KFDW SFN--DADLP VDTWKIMMY-
  89784 VIN----TIC SENTLRFVQS LPKRER---- ----QPLKDK FKN---ADPQ ---------- ---------- --------AI ELLERILVFD PRKRIKAGEA LADPYLAPY- ----HDPTDE PEAEE-KFDW SFN--DADLP VDTWKIMMYV
 114108 VIQ----TIC SENTLRFVQS LPKRER---- ----QPLKNK FKN---ADPQ ---------- ---------- --------AI ELLERMLVFD PRKRVKAGEA LADQYLAPY- ----HDPTDE PVAEE-KFDW SFN--DADLP VDTWKIMMY-
 173207 SFWD---PSE S--VATHLEQ LCTVNGQPPP -RHPIDFTSL FPD---CPTD ---------- ---------- --------GI DILQQLLQLD PHQRITVTDA IAHPFLAPF- ----GDPEEE SME---PGTC DFH-FEQYAN DEDLRQHV--
 127887 SFWD---KNA SEFVIEYIKN LRDGDGLPPP -KEPIDFSSL FPD---CPME ---------- ---------- --------GI DLLKNLLHLN PGLRITPDEA LAHPFVEQM- ----RDPADE VDC---PETF DFESFEYLQQ EDALRRSI--
  29226 SFWD---QNA SKSVVDYIKN LRDVNGEPPP -TEPIDFAAH FPE---CPPE ---------- ---------- --------GI DLLQRLLHLD PKRRINANEA LEHPYVALV- ----RDPPEE VEC---NLMF DFESFERIQD EDTLRQCI--
 167765 SFWD---SSS S--VTEYIRN LRDESGLPPP -EEPIDFSTL FPN---APPE ---------- ---------- --------AI HLLENLLTLD PSKRLTANEA LEHPFVAPV- ----RDPAEE IEC---ATLF DFESFEMIES EHVLRQCI--
 150384 SFWD---SSS S--VTEYIRN LRDESGLPPP -EEPIDFSTL FPN---APPE ---------- ---------- --------AI HLLENLLTLD PSKRLTANEA LEHPFVAPV- ----RDPAEE IEC---ATLF DFESFEMIES EHVLRQCI--
  57923 TFWD---SSS S--VTEYIRS LRDVNGNPPP -EEPIDFSLL FPN---CPPE ---------- ---------- --------GI DLLKQLLMLN PSKRITASEA LKHPFVASV- ----RDKSEE IEC---EKLF DFESFEMIES EHVLRQCI--
  57928 TFWD---SSS S--VTEYIRS LRDVNGNPPP -EEPIDFSLL FPN---CPPE ---------- ---------- --------GI DLLKQLLMLN PSKRITASEA LKHPFVASV- ----RDKSEE IEC---EKLF DFESFEMIES EHVLRQCI--
 230955 SFWN---TMA SPTVIEYIHN LRSPDGSPPP -TDPVDFQSL YPQ---CSTN ---------- ---------- --------GI NILTQMLHLD PAKRITVDQA LRHPYLADF- ----RDPGEE YDC---PRSF AFEPFEKVDS IDELRELI--
 172033 SFWN---TMA SPTVIEYIHN LRSPDGSPPP -TDPVDFQSL YPQ---CSTN ---------- ---------- --------GI NILTQMLHLD PAKRITVDQA LRHPYLADF- ----RDPGEE YDC---PRSF AFEPFEKVDS IDELRELI--
  40306 ETLS---RIG SQRAQEYVRS LPYMPR---- ----VPFSKL FPT---ANPQ ---------- ---------- --------AL DLLSRMLAFD PAERMTVQEA LEHPYLSVW- ----HDPADE PEC---PTTF DF-SFEAVDS VEDMREMI--
 693957 ETLS---RIG SPRAQDYVRN LPYMQK---- ----VQFASI FRQ---ANPD ---------- ---------- --------AL DLLDRMLAFD PSSRISVEEA LEHRYLHIW- ----HDASDE PTC---PTTF DF-QFEVVED IGEMKKMI--
 508525 ETLS---RIG SPRAQDYVRN LPYMPK---- ----IAFATL FRQ---ANPD ---------- ---------- --------AL DLLDRMLAFD PSSRISVEEA LEHRYLHIW- ----HDASDE PSC---PTTF DF-QFEVVED IQEMKHMI--
 471145 ETLS---RIG SPRAQDYVRN LPYMPK---- ----IAFATL FRQ---ANPD ---------- ---------- --------AL DLLDRMLAFD PSSRISVEEA LEHRYLHIW- ----HDASDE PSC---PTTF DF-QFEVVED IQEMKHMI--
 480536 ETLS---RIG SPRAQDYVRN LPYMPK---- ----IAFATL FRQ---ANPD ---------- ---------- --------AL DLLDRMLAFD PSSRISVEEA LEHRYLHIW- ----HDASDE PSC---PTTF DF-QFEVVED IQEMKHMI--
 471745 ETLS---RIG SPRAQDYVRN LPYMQK---- ----VSFSSL FRQ---ANPD ---------- ---------- --------AL DLLDLMLAFD PSSRISVEEA LEHRYLHIW- ----HDASDE PSC---PTTF DF-QFEVVEE IPEMKVMI--
 687176 ETLS---RIG SPRAQDYVRN LPYMQK---- ----VSFASL FRQ---ANPD ---------- ---------- --------AL DLLDRMLAFD PSSRISVEDA LEHRYLHIW- ----HDASDE PSC---PTTF DF-HFEVVED IAEMKKMI--
  34863 STLA---RIG SPRAQEYVRN LPYMQK---- ----IPFHTL FRN---ANPD ---------- ---------- --------AL DLLDRMLAFD PSQRIDVDEA LEHRYLSIW- ----HDASDE PNC---PTTF DF-GFEVVEE VPEMRQMI--
  57153 STLS---RIG SPRAQEYVRN LPYMSK---- ----IPFQQL FPR---ANPD ---------- ---------- --------AL DLLDRMLAFD PSQRIGVEEA LEHRYLAIW- ----HDASDE PPC---PTTF DF-GFEVVDE VPEMRQLI--
 147961 STLS---RIG SPRAQEYVRN LPYMQK---- ----IPFQTL FHR---ANPD ---------- ---------- --------AL DLLDRMLAFD PSQRISVEDA LEHRYLSIW- ----HDASDE PAC---PTPF DF-GFEVVDE VPEMRQMI--
 287354 STLA---RIG SPRAQEYVRN LPYMPK---- ----IPFNQL FPR---ANPD ---------- ---------- --------AL DLLDRMLAFD PSQRISVEEA LEHRYLAIW- ----HDASDE PPC---PTTF DF-AFETVED VGEMRLMI--
 281614 ATLS---RIG SPRAQDYVRN MAYMPR---- ----VPSSQQ FPR---ANPD ---------- ---------- --------AL DLLDRMLAFD PSERIKVEEA LAHPYLAIW- ----HDPSDE PSC---AVPF NF-AFEVVEE VSDMRQMI--
  29684 ETLS---RIG SPRAQEYVRN LPFMPK---- ----KPFASL FPN---ANPD ---------- ---------- --------AL DLLDRMLAFD PSSRISVEQA LEHPYLQIW- ----HDASDE PDC---PTTF DF-DFEVVED VGEMRKMI--
 144400 ETLS---RIG SPRAQEYVRN LPPMPK---- ----KSFQQL FPQ---ANPD ---------- ---------- --------AL DLLDRMLAFD PTSRISVEDA LKHPYLSIW- ----HDASDE PDC---PTPF NF-DFEVVDD VGEMRKMI--
 136175 ETLS---RIG SPRAQEYVRN LPPMPK---- ----KSFQQL FPQ---ANPD ---------- ---------- --------AL DLLDRMLAFD PTSRISVEDA LKHPYLSIW- ----HDASDE PDC---PTPF NF-DFEVVDD VGEMRKMI--
 126818 ETLS---RIG SPRAQEYVRN LPPMPK---- ----KSFQQL FPQ---ANPD ---------- ---------- --------AL DLLDRMLAFD PTSRISVEDA LKHPYLAIW- ----HDASDE PDC---PTPF NF-DFEVVDD VGEMRKMI--
2138682 ETLS---RIG SPRAQEYVRN LPYMSK---- ----KPFAQL FPN---ANPD ---------- ---------- --------AL DLLDRMLAFD PTRRISVEEA LEHPYLAIW- ----HDASDE PDC---PTTF NF-DFEVIDD VNEMRKMI--
  48096 ETLS---RIG SPRAQEYVRN LPYMAK---- ----KSFPSL FPN---ANPD ---------- ---------- --------AL DLLDRMLAFD PTRRISVEAA LEHPYLAIW- ----HDASDE PDC---PTTF NF-DFEVIDD VNEMRKMI--
 179022 ETLS---RIG SPRAQEYVRN LPYMAK---- ----KSFPSL FPN---ANPD ---------- ---------- --------AL DLLDRMLAFD PTRRISVEAA LEHPYLAIW- ----HDASDE PDC---PTTF NF-DFEVVDD VAEMRKMI--
 778216 ETLS---RIG SPRAQEYVRN LPFMAK---- ----KSFPSL FPN---ANPD ---------- ---------- --------AL DLLDRMLAFD PSRRISVEAA LEHPYLAIW- ----HDASDE PDC---PTTF NF-EFEVVED IDEMRKMI--
 665550 ETLS---RIG SPRAQEYVRN LPFMAK---- ----RPFPSL FPN---ANPD ---------- ---------- --------AL DLLNHMLAFD PSSRISVEEA LEHPYLQIW- ----HDASDE PGC---PTTF NF-DFEVVED VGEMRKMI--
 190738 ETLS---RIG SPRAQEYVRN LPFMAK---- ----RTFQSL FPN---ANPD ---------- ---------- --------AL DLLNHMLAFD PSSRISVETA LEHPYLHIW- ----HDASDE PGC---PTTF NF-DFEVVED VGEMRKMI--
1107049 ETLR---RIG SPRAQEYVRN LPFMPK---- ----KPFPAL FPD---ANPD ---------- ---------- --------AL DLLDKMLAFD PSQRISVEQA LEHPYLHIW- ----HDASDE PDC---PTTF NF-DFEVVED VGEMRKMI--
 504269 ETLR---RIG SPRAQDYVRN LPYMPK---- ----IAFQRL FPN---ANPD ---------- ---------- --------AL DLLDRMLAFD PSSRISVEDA LEHPYLQIW- ----HDASDE PTC---PTTF DF-RFEVVDD IHEMRKMI--
 432134 ETLR---RIG SPRAQDYVRN LPYMPK---- ----IAFQRL FPN---SNPD ---------- ---------- --------AL DLLDRMLAFD PSSRISVEDA LEHPYLQIW- ----HDASDE PTC---PTTF DF-RFEVVDD IHEMRKMI--
  59068 ETLS---RIG SPRAQEYVRN LPYMPK---- ----IPFERL FPN---ANPQ ---------- ---------- --------AL DLLDRMLAFD PSKRISVEEA LEHPYLQIW- ----HDASDE PTC---PTTF DF-HFEVIDD VQEMRKMI--
 336185 ETLA---RIG SPRAQEYVRN LPFMPK---- ----VPFKTL FPQ---ANPD ---------- ---------- --------AL DLLDRMLAFD PSSRISVEEA LEHPYLHIW- ----HDASDE PNC---PTTF DF-TFEVVDE VPDMRKMI--
 343155 ETLA---RIG SPRAQEYVRN LPFMPK---- ----VPFKQL FPQ---ANPD ---------- ---------- --------AL DLLDRMLAFD PSSRISVEEA LEHPYLHIW- ----HDASDE PNC---PTTF DF-HFEVVDE VPEMRKMI--
 326648 DTLA---RIG SPRAQEYVRN LPFMPK---- ----VPFKQL FPQ---ANPD ---------- ---------- --------AL DLLDRMLAFD PSSRISVEEA LEHPYLHIW- ----HDASDE PNC---PTTF DF-HFEVVDE VHDMRKMI--
 425863 ETLA---RIG SPRAQEYVRN LPFMPK---- ----IPFQRL FPN---ANPD ---------- ---------- --------AL DLLDRMLAFD PSSRISVEEA LEHPYLHIW- ----HDASDE PTC---PTTF DF-QFEVVDD VPEMRKMI--
 436998 ETLS---RIG SPRAQEYVRN LPFMPK---- ----IPFQRL FPQ---ANPD ---------- ---------- --------AL DLLDRMLAFD PSKRISVEEA LEHRYLHIW- ----HDASDE PSC---PTTF DF-HFEVVDD VGEMRKMI--
 257317 ETLS---RIG SPRAQEYVRN LPYMPK---- ----IPFTRL FPN---ANPD ---------- ---------- --------AL DLLDRMLAFD PSSRISVEDA LEHRYLHIW- ----HDASDE PSC---PTTF DF-AFEVVED VGDMRRMI--
 998070 ETLS---RIG SPRAQDYVRN LPYMQK---- ----VSFQAL FRN---ANPD ---------- ---------- --------AL DLLDRMLAFD PSSRISVEEA LEHRYLHIW- ----HDASDE PSC---PTTF DF-HFEVVEE INEMKKMI--
  49449 ETLS---RIG SPRAQEYVRN LPHMPK---- ----VSFQAL FRS---ANPD ---------- ---------- --------AL DLLDRMLAFD PSSRISVEEA LEHRYLHIW- ----HDASDE PAC---PTTF DF-HFEVVED IAEMKKMI--
 498377 ETLS---RIG SPRAQEYVRN LPYMQK---- ----VAFQSL FRT---ANPE ---------- ---------- --------AL DLLDKMLAFD PSRRISVEEA LEHKYLHIW- ----HDASDE PSC---LTTF DF-TFEVVED VAEMRKMI--
 184715 ETLR---RIG SPRAQDYVRN LPYMPK---- ----IAFQRL FPN---SNPD ---------- ---------- --------AL DLLDRMLAFD PSSRISVEDA LEHPYLQIW- ----HDASDE PTC---PTTF DF-RFEVVDD IHEMRKMI--
  51223 ETLC---RIG SARAQEYVRN LPFMPK---- ----APFSRL FPN---ANPD ---------- ---------- --------AL DLLDRMLAFD PSSRISVEEA LQHRYLSIW- ----HDASDE PDC---PTPF DF-SFESVNE VPEMRKMI--
  66669 ETLC---RIG SARAQEYVRN LPFMPK---- ----APFSRL FPN---ANPD ---------- ---------- --------AL DLLDRMLAFD PSSRISVEEA LQHRYLSIW- ----HDASDE PDC---PTPF DF-SFESVNE VPEMRKMI--
 649898 ETLS---RIG SPRAQEYVRN LPFMPK---- ----VPFQRL FPN---ANPD ---------- ---------- --------AL DLLDQMLAFD PSSRVSVEKA LEHRYLSIW- ----HDASDE PDC---ETTF DF-SFEVVED VPQMRKMI--
 253817 ETLS---RIG SPRAQEYVRN LPFMPK---- ----VPFQRL FPN---ANPD ---------- ---------- --------AL DLLDQMLAFD PSSRVSVEKA LEHRYLSIW- ----HDASDE PDC---ETTF DF-SFEVVED VPQMRKMI--
 618068 ETLS---RIG SPRAQEYVRN LPFMAK---- ----KSFPSL FPN---ANPD ---------- ---------- --------AL DLLDRMLAFD PSRRISVEAA LEHPYLAIW- ----HDASDE PDC---PTTF NF-EFEVVED IDEMRKMI--
 108847 SALT---KIG SPRAQNYVRS LPIMRK---- ----IHYREI LPN---ANPL ---------- ---------- --------AL DLLERMLAFD PHERITVEEA LNHPYLAVW- ----HDPQDE PES---KIKF DFKTFETVDD MESMKQLI--
  33629 ATLT---KIG SVRAQNYVRS LPLMKK---- ----VSYSEL FPN---ANPL ---------- ---------- --------AL DLLEKMLTLD PFERISVEEA LSHPYLAVW- ----HDPQDE PEC---QVKF NFKSFETVDN MDDMKQLI--
  54715 ATLT---RIG SHRAQNYVRS LPIMRK---- ----VSYKQL FPN---ANPL ---------- ---------- --------AL DLLEKMLALD PSERISVEDA LNHPYLEVW- ----HDPRDE PEC---QVKF DFKSFETVDS IEDMKQLI--
  88920 ETLS---RIG SPRAQQYVRN LPFMPR---- ----VPFTHV FPN---ANPL ---------- ---------- --------AL DLLSKLLTFD PAKRITVEEA LKHPYLSVW- ----HDPSDE PTC---SQEF DF-GFESVQD VDEMRQMI--
  51479 ETLS---RIG SPRAQDYVRS LPYMSP---- ----VDFVTL FPQ---ANPE ---------- ---------- --------AL DLLAKMLVFD PAQRITVDEA LQHPYLKIW- ----HDPADE PDC---ADKF DF-SFESVDD IAAMKQMI--
  86943 STLT---RIG SKRAQEYVRS LPLMAK---- ----VPFSGL FPN---ANSD ---------- ---------- --------AL DLLEKMLTLD PQERITVEDA LNHPYLKIW- ----HDPNDE PSC---HSKF DF-SFEVVDD MQLMKQMI--
 100835 ETLT---RIG SQRAQDYVRS LPFMPK---- ----IPFSKL YPE---ANPE ---------- ---------- --------AL DLLDRMLCLD PAKRITVEEA LTHPYLKVW- ----HDPADE PIC---ASKF DF-KFEEVDE LDTMRQMI--
 319719 ETLR---RIG SKNVQDYIHQ LGFIPK---- ----VPFVNL YPN---ANSQ ---------- ---------- --------AL DLLEQMLAFD PQKRITVDEA LEHPYLSIW- ----HDPADE PVC---SEKF EF-SFESVND MEDLKQMV--
 822494 ETLR---RIG SKNVQDYIHQ LGFIPK---- ----VPFVNL YPN---ANSQ ---------- ---------- --------AL DLLEQMLAFD PQKRITVDEA LEHPYLSIW- ----HDPADE PVC---SEKF EF-SFESVND MEDLKQMV--
 161919 ETLC---RVG SERAQVYIRS LTRMPK---- ----IPFQNL YPR---ASPL ---------- ---------- --------AI DLLNKLLTFD PAKRITVEEA LAHPYLSAY- ----HDEDDE PSH---TQTF DF-SFEVVES MEDMRKMI--
 158631 ETLC---RVG SERAQVYIRS LPRMPK---- ----IPFENL YPR---ANPM ---------- ---------- --------AI DLLNKLLEFD PSKRIDVDEA LAHPYLSAY- ----HDEDDE PTH---SSTF DF-SFEVVDS VDEMRRMI--
 115223 ETLR---RVG SERAQVYIRS LPRMPR---- ----IPFENL YPR---ANPL ---------- ---------- --------AI DLLNKLLEFD PSKRITVEEA LAHPYLSAY- ----HDEDDE PVH---TQTF DF-SFEVVDS IEEMSRMI--
 344665 ETLR---RVG SERAQIYISS LPKMPT---- ----IPFSQL YPK---ANPL ---------- ---------- --------AL DLLEKLLKFD PATRITVEQA LAHSYLAAY- ----HDEEDE PAH---EHMF DF-SFESVEL IEDMKKMI--
 178050 ETLQ---RVG SDRAQVYIRS LPKMPK---- ----VPFQNL YPK---ANPL ---------- ---------- --------AI DLLNKLLTFD PAKRMTVEEA LEHPYLSTY- ----HDEDDE PAH---QHNF DF-TFEILES LDDMRSMI--
 234927 ATLR---RVG SERAQVYIRS LPPMPR---- ----IPFEKL YPK---ANPL ---------- ---------- --------AI DLLNKLLCFD PALRITVEEA LAHPYLSAY- ----HDEDDE PVH---HQHF DF-SFESVEA MDDMKKLI--
 328916 ATLT---RIG STRAQEYIRN LPYMHK---- ----TTFQQL FPN---ANPL ---------- ---------- --------AI DLLERLLTFD PAQRITVEEA LEHPYLAVW- ----HDPNDE PVC---PEFF DF-SFETVDD PDQMRAMI--
 273327 ATLT---RIG STRAQEYIRN LPYMHK---- ----TTFQQL FPN---ANPL ---------- ---------- --------AI DLLERLLTFD PAQRITVEEA LEHPYLAVW- ----HDPNDE PVC---PEFF DF-SFETVDD PDQMRAMI--
 338804 ATLT---RIG STRAQEYIRN LPYMHK---- ----TTFQQL FPN---ANPL ---------- ---------- --------AI DLLERLLTFD PAQRITVEEA LEHPYLAVW- ----HDPNDE PVC---PEFF DF-SFETVDD PDQMRAMI--
 527701 ETLR---RVG SPRAQDYIRS LPIKPR---- ----VPFSTL FPR---ANPL ---------- ---------- --------AI DLLTQMLHFD PIKRISCEQA LNHPYFAVW- ----HDLADE PVC---PSTF HF-GFEEEDG IEGMKKLI--
  81317 DTLR---RVG SPRAQDYIRS LPIKPR---- ----ISFATL YPR---ANPL ---------- ---------- --------AI DLLSKMLCFD PAKRISCEEA LNHPYLQVW- ----HDPADE PVC---EKKF DF-SFEEEDN IDGMKKLI--
 831329 DTLR---RVG SPRAQDYIRS LPIKPR---- ----VPFATL YPH---ANPL ---------- ---------- --------AI DLLLQMLCFD PAKRISCEQA LNHPYLQVW- ----HDPADE PIC---ETKF DF-SFEEEDS IDGMKKLI--
 676624 DTLR---RVG SPRAQDYIRS LPIKPR---- ----VPFQTL YPH---ANPL ---------- ---------- --------AI DLLQQMLCFD PAKRISCEAA LNHPYLQVW- ----HDPADE PIC---DTKF DF-SFEEEDS IEGMKRLI--
 826941 DTLR---RVG SPRAQDYIRS LPIKPR---- ----IPFSTL YPH---ANPL ---------- ---------- --------AI DLLAQMLCFD PAKRISCEQA LNHPYLQVW- ----HDPADE PVC---ETKF DF-GFEEEDT IEGMKKLI--
  77377 DTLR---RVG SPRAQDYIRS LPIKPR---- ----IPFSTL FPG---ANPN ---------- ---------- --------AI DLLSRMLCFD PAKRLSCEEA LNHPYLQVW- ----HDPADE PTC---EAKF DF-SFEEEDS IEGMKKLI--
  59915 DTLR---RVG SPRAQDYIRS LPIKPR---- ----IPFQNL YPH---ANPL ---------- ---------- --------AI DLLSKMLCFD PAKRISCEDA LKHPYLQVW- ----HDPADE PVC---EKKF DF-SFEEEDS IEGMKKLI--
 160641 DTLR---RVG SPRAQDYIRS LPIKPR---- ----VPFSTL FPR---ANPL ---------- ---------- --------AI DLLSQLLNFD PAKRITCEQA LNHPYLAVW- ----HDPADE PVC---PSTF DF-GFEEEDS IEGMKRLI--
 108839 DTLR---RVG SPRAQDYIRS LPIRPR---- ----VPFSTL FPR---ANPL ---------- ---------- --------AI DLLSQLLNFD PAKRITCEQA LNHPYLQVW- ----HDPADE PTC---PTSF DF-GFEDEDS IEGMKRLI--
1536187 DTLR---RVG SPRAQDYIRS LPIKPR---- ----VPFSTL FPR---ANPL ---------- ---------- --------AI DLLSQLLHFD PAKRITCEQA LNHPYLAVW- ----HDPADE PVC---NSAF DF-GFEEEDT IEGMKRLI--
 281708 DTLR---RVG SPRAQDYIRS LPIKPR---- ----VPFQTL FPR---ANPL ---------- ---------- --------AI DLLAQLLHFD PAKRITCEQA LNHPYLAVW- ----HDPADE PVC---ASSF DF-GFEEEDS IEGMKRLI--
 173791 DTLR---RVG SPRAQDYIRS LPIKPR---- ----VPFSTL FPR---ANPL ---------- ---------- --------AI DLLAQLLHFD PAKRITCEQA LNHPYLAVW- ----HDPADE PTC---ASPF DF-GFEEEDS IEGMKRLI--
  82703 DTLR---RVG SPRAQDYIRS LPIKPR---- ----VPFQTL FPR---ANPL ---------- ---------- --------AI DLLSQLLHFD PARRITCEQA LNHPYLQVW- ----HDPADE PVC---PTPF DF-GFEEEDS IEGMKRLI--
 139538 ETLR---RVG SPRAQDYIRS LPIKPR---- ----VPFATL YPH---ANPM ---------- ---------- --------AI DLLSKMLCFD PSKRISCEQA LEHPYLQVW- ----HDPADE PVC---EAKF DF-KFEEEDS IDGMKRLI--
 479859 DTLR---RVG SPRAQDYIRS LPIKPR---- ----ILFQTL YPH---ANPL ---------- ---------- --------AL DLLGKLLAFD PAKRISCEEA LNHPYVQVW- ----HDPLDE PVC---DTPF DF-GFEEEDS IEGMKQLI--
 621303 DTLR---RVG SPRAQDYIRS LPIKPR---- ----IPFSTL YPN---ANPL ---------- ---------- --------AI DLLSKLLTFD PAKRISCEQA LAHPYFSVW- ----HDPSDE PIC---EKKF DF-SFEEEDS IEGMKNLI--
  38569 DTLR---RVG SPRAQDYIRS LPIKPR---- ----IPFSTL YPN---ANPL ---------- ---------- --------AI DLLSKLLTFD PAKRISCEQA LAHPYFSVW- ----HDPSDE PIC---EKKF DF-SFEEEDS IEGMKNLI--
 242842 ETLR---RVG SPRAQDYIRS LPIKPR---- ----IQFQTL YPN---ANPL ---------- ---------- --------AI DLLGKMLAFD PAKRISCEEA LNHPYFAVW- ----HDPNDE PVS---QSRF DF-AFEEEEN MDGMKRMI--
2481011 DTLR---RVG SPRAQDYIRS LPIKPR---- ----VPFATL FPH---ANPL ---------- ---------- --------AI DLLSKMLCFD PAKRISCEQA LNHPYFQVW- ----HDPADE PIC---EAKF DF-GFEEEDS IEGMKKLI--
 169913 DTLR---RVG SPRAQDYIRS LPIRPR---- ----VPFQQL FPQ---ANPQ ---------- ---------- --------AI DLLSKMLNFD PAKRITCEQA LEHPYLAVW- ----HDPSDE PVC---PTTF DF-DFEEEDS IEGMKKLI--
  71742 DALR---RVG SPRAQDYIRS LPIKPR---- ----VPFSTL FPN---ANPL ---------- ---------- --------AI DVLSLMLCFD PAKRMSCEQA LNHPYFQAW- ----HDVTDE PVC---ATKF DF-GFEEEDT IDGMKKLI--
  66635 QTLR---RVG SPRAQEYIRS LPYKAG---- ----IPFNLL YPH---ANPQ ---------- ---------- --------AL DLLARLLAFD PAERITCQDA LRHPYLSVW- ----YDPADI LTT---TTTF DF-AFENEDT IEGMKVLI--
  53870 QTLR---RVG SPRAQEYIRS LPYKAG---- ----IPFNLL YPH---ANPQ ---------- ---------- --------AL DLLARLLAFD PAERITCQDA LRHPYLSVW- ----YDPADI LTT---TTTF DF-AFENEDT IEGMKVLI--
  27246 DTLR---RVG SPRAQDYIRS LPIKPR---- ----VPFATL YPH---ANPL ---------- ---------- --------AI DLLLQMLCFD PAKRISCEQA LNHPYLQVW- ----HDPADE PIC---ETKF DF-SFEEEDS IDGMKKLI--
  88978 DALR---RVG SPRAQDYIRS LPIKPR---- ----VPFSTL FPN---ANPL ---------- ---------- --------AI DVLSLMLCFD PAKRMSCEQA LNHPYFQAW- ----HDVTDE P-------KF DF-GFEEEDT IDGMKKLI--
1091096 DTLR---RVG SPRAQDYIRS LPIKPR---- ----VPFSTL YPH---ANPL ---------- ---------- --------AI DLLSQMLCFD PAKRISCEQA LNHPYLQVW- ----HDPADE PIC---ESKF DF-SFEEEDS IEGMKKLI--
  66856 DTLR---RVG SPRAQDYIRS LPIKPR---- ----VPFSTL FPR---ANPL ---------- ---------- --------AI DLLAQLLHFD PAKRITCEQA LNHPYLAVW- ----HDPADE PTC---ASPF DF-GFEEEDS IEGMKRLI--
  20720 DTLR---RVG SPRAQDYIRS LPIKPR---- ----VPFQTL FPR---ANPL ---------- ---------- --------AI DLLSQLLHFD PARRITCEQA LNHPYLQVW- ----HDPADE PVC---PTPF DF-GFEEEDS IEGMKRLI--
 124539 DTLR---RVG SPRAQDYIRS LPIKPR---- ----IPFQNL YPH---ANPL ---------- ---------- --------AI DLLSKMLCFD PAKRISCEDA LKHPYLQVW- ----HDPADE PVC---EKKF DF-SFEEEDS IEGMKKLI--
 361187 ETLS---RIG SPRAQEYVRN LPFMAK---- ----KPFPQL FPN---ANPE ---------- ---------- --------AL DLLDRMLAFD PTSRITVEQA LEHPYLHIW- ----HDASDE PEC---KTTF NF-DFEVVDD VGEMRKMI--
 327531 DALR---RVG SPRAQDYIRS LPIKPR---- ----IPFQQL YPQ---ANPQ ---------- ---------- --------AI DLLGHMLCFD PAQRISCEDA LKHPYLTVW- ----HDPMDE PVC---QTKF DF-GFEEEET IEGMKKLI--
   7086 DALR---RVG SPRAQDYIRS LPIKPR---- ----IPFQQL YPQ---ANPQ ---------- ---------- --------AI DLLGHMLCFD PAQRISCEDA LKHPYLTVW- ----HDPMDE PVC---QTKF DF-GFEEEET IEGMKKLI--
  28915 ATLR---RVG SERAQAYIRS LPRMPT---- ----IHFQQL YPR---ASPL ---------- ---------- --------AI DLLEKLLNFD PAARITVEQA LAHPYLAAY- ----HEEDDE PSH---EKVF DF-SFEVTDR IDEMKRLI--
 669145 DTLR---RVG SPRAQDYIRS LPIKPR---- ----VPFQQL FPQ---ANPL ---------- ---------- --------AL DLLAKMLNFD PAKRITCDQA LEHPYLAVW- ----HDPSDE PSC---PTKF DF-GFESEES SEGMKKLI--
  46685 QTLR---RVG SPRAQDYIRS LPHKPG---- ----VPFDQL YPN---ANPL ---------- ---------- --------AL DLLSKLLSFD PSERITCNEA LRHPYLAVW- ----HDPADE IDC---QERF DF-GFEAEDS TEGMKRLI--
  53954 ATLR---RVG SPRAQDYIRS LPYKPG---- ----VPFVSL FPQ---ANPL ---------- ---------- --------AL DLVAKLLAFD PHERISCEDA LLHPYLSVW- ----HEPADE PTC---PTRF DF-GFESEDT IEGMRALI--
1446322 DTLR---RVG SPRAQDYIRS LPIKPR---- ----IPFSTL YPH---ANPL ---------- ---------- --------AI DLLSQMLAFD PAKRISCEQA LNHPYFAVW- ----HEPSDE PVC---DVPF DF-GFEEEES IEGMKKLI--
 351754 DTLR---RVG SPRAQDYIRS LPIKPR---- ----IPFSTL YPQ---ANPL ---------- ---------- --------AI DLLSKMLTFD PAKRISCEQA LAHPYLAVW- ----HDLSDE PIC---DAKF DF-SFEEEDT IEGMKRLI--
  65873 ETLR---RVG SPRAVEYIRS LPIKPR---- ----IPFERI YPK---ANPL ---------- ---------- --------AL DLLSKMLTFD PAKRITCDEA LKHPYLAVW- ----HDPTDE PSC---PERF DF-GFEVEDS LEGMKSAI--
  52469 ETLR---RVG SPRAVEYIRS LPIKPR---- ----IPFERI YPK---ANPL ---------- ---------- --------AL DLLSKMLTFD PAKRITCDEA LKHPYLAVW- ----HDPTDE PSC---PERF DF-GFEVEDS LEGMKSAI--
  30002 ETLR---RVG SPRAVEYIRS LPIKPR---- ----IPFERI YPK---ANPL ---------- ---------- --------AL DLLSKMLTFD PAKRITCDEA LKHPYLAVW- ----HDPTDE PSC---PERF DF-GFEVEDS LEGMKSAI--
 154156 DTLN---RIG SERAQTYIRG LERFAK---- ----IPLDQL YPN---ATET ---------- ---------- --------AL DLLEKLLTFD PMSRIGVEDA LDHPYFELF- ----HDPDDE PTH---FATV DF-SFESLNT IEEMKETI--
 166841 DTLN---RIG SERAQTYIRG LERFAK---- ----IPLDQL YPN---ATET ---------- ---------- --------AL DLLEKLLTFD PMSRIGVEDA LDHPYFELF- ----HDPDDE PTH---FATV DF-SFESLNT IEEMKETI--
 238118 ATLS---RVG SERAQIYIKS LEKFQK---- ----IPLSQL YPN---ASPT ---------- ---------- --------AL DLLEKLLTFD PSSRITVEEA LEHPYFELF- ----HDPNDE PSH---STLF DF-SFESLDT IEDMKETI--
 143069 ATLS---RVG SERAQIYIKS LEKFQK---- ----IPLSQL YPN---ASPT ---------- ---------- --------AL DLLEKLLTFD PSSRITVEEA LEHPYFELF- ----HDPNDE PSH---STLF DF-SFESLDT IEDMKETI--
 456995 ETLR---RVG SPRAQDYIRS LPIKPR---- ----VPFATL YPH---ANPM ---------- ---------- --------AI DLLSKMLCFD PSKRISCEQA LEHPYLQVW- ----HDPADE PVC---EAKF DF-KFEEEDS IDGMKRLI--
 175880 ETLR---RVG SPRAQDYIRS LPIKPR---- ----IQFQTL YPN---ANPL ---------- ---------- --------AI DLLGKMLAFD PAKRISCEEA LNHPYFAVW- ----HDPNDE PVS---QSRF DF-AFEEEEN MDGMKRMI--
  28309 ETLQ---EIA SQKVYNYIFQ FGNIPG---- ----RSFESI LPG---ANPE ---------- ---------- --------AL ELLKKMLEFD PKKRITVEDA LEHPYLSMW- ----HDIDEE FSC---QKTF RF-EFEHIES MAELGNEV--
 767958 ETLQ---EIA SQKVYNYIFQ FGNIPG---- ----RSFESI LPG---ANPE ---------- ---------- --------AL ELLKKMLEFD PKKRITVEDA LEHPYLSMW- ----HDIDEE FSC---QKTF RF-EFEHIES MAELGNEV--
 126152 ETLQ---EIA SQKVYNYIFQ FGNIPG---- ----RSFESI LPG---ANPE ---------- ---------- --------AL ELLKKMLEFD PKKRITVEDA LEHPYLSMW- ----HDIDEE FSC---QKTF RF-EFEHIES MAELGNEV--
 613191 ETLS---RIG SPRAQEYVRN LPFMPK---- ----KPFASL FPN---ANPD ---------- ---------- --------AL DLLDRMLAFD PSSRISVEQA LEHPYLQIW- ----HDASDE PDC---PTTF DF-DFEVVED VGEMRKMI--
 198058 ETLS---RIG SPRAQEYVRN LPFMAK---- ----KPFPQL FPN---ANPE ---------- ---------- --------AL DLLDRMLAFD PTSRITVEQA LEHPYLHIW- ----HDASDE PEC---KTTF NF-DFEVVDD VGEMRKMI--
 123006 ETLS---RIG SPRAQEYVRN LPYMAK---- ----KSFPSL FPN---ANPD ---------- ---------- --------AL DLLDRMLAFD PTRRISVEAA LEHPYLAIW- ----HDASDE PDC---PTTF NF-DFEVIDD VNEMRKMI--
 178993 ETLS---RIG SPRAQEYVRN LPYMAK---- ----KSFPSL FPN---ANPD ---------- ---------- --------AL DLLDRMLAFD PTRRISVEAA LEHPYLAIW- ----HDASDE PDC---PTTF NF-DFEVVDD VAEMRKMI--
 122967 ETLS---RIG SPRAQEYVRN LPYMAK---- ----KSFPSL FPN---ANPD ---------- ---------- --------AL DLLDRMLAFD PTRRISVEAA LEHPYLAIW- ----HDASDE PDC---PTTF NF-DFEVIDD VNEMRKMI--
 120501 ETLS---RIG SPRAQEYVRN LPPMPK---- ----KSFQQL FPQ---ANPD ---------- ---------- --------AL DLLDRMLAFD PTSRISVEDA LKHPYLAIW- ----HDASDE PDC---PTPF NF-DFEVVDD VGEMRKMI--
  99018 ETLS---RIG SPRAQDYVRN LPYMQK---- ----VSFASL FRQ---ANPD ---------- ---------- --------AL DLLDRMLAFD PSSRISVEEA LEHRYLHIW- ----HDASDE PSC---PTTF DF-QFEVVEE IPEMKTMI--
 213678 ETLS---RIG SPRAQDYVRN LPYMPK---- ----IAFATL FRQ---ANPD ---------- ---------- --------AL DLLDRMLAFD PSSRISVEEA LEHRYLHIW- ----HDASDE PSC---PTTF DF-QFEVVED IQEMKHMI--
 460523 ETLS---RIG SPRAQDYVRN LPYMQK---- ----VSFSSL FRQ---ANPD ---------- ---------- --------AL DLLDLMLAFD PSSRISVEEA LEHRYLHIW- ----HDASDE PSC---PTTF DF-QFEVVEE IPEMKVMI--
 625621 ETLS---RIG SPRAQEYVRN LPFMPK---- ----VPFQRL FPN---ANPD ---------- ---------- --------AL DLLDQMLAFD PSSRVSVEKA LEHRYLSIW- ----HDASDE PDC---ETTF DF-SFEVVED VPQMRKMI--
 265871 ETLA---RIG SPRAQEYVRN LPFMPK---- ----VPFKQL FPQ---ANPD ---------- ---------- --------AL DLLDRMLAFD PSSRISVEEA LEHPYLHIW- ----HDASDE PNC---PTTF DF-HFEVV-- ----------
  53464 ETLS---RIG SPRAQEYVRN LPYMPK---- ----IPFTRL FPN---ANPD ---------- ---------- --------AL DLLDRMLAFD PSSRISVEDA LEHRYLHIW- ----HDASDE PSC---PTTF DF-AFEVVED VGDMRRMI--
```

```
     105414  ATLS---RIG SPRAQEYVRN LPYMPK---- ----IPFHTL FPR---ANPD ---------- ---------- --------AL DLLDKMLAFD PSSRIGVEEA LEHRYLSIW- ----HDATDE PNC---PTKF DF-GFEVVDD VSEMRHMI--
     291876  ATLS---RIG SPRAQEYVRN LPYMPK---- ----IPFHTL FPR---ANPD ---------- ---------- --------AL DLLDKMLAFD PSSRIGVEEA LEHRYLSIW- ----HDATDE PNC---PTKF DF-GFEVVDD VSEMRHMI--
      50744  SALT---KIG SPRAQNYVRS LPIMRK---- ----IHYREI LPN---ANPL ---------- ---------- --------AL DLLERMLAFD PHERITVEEA LNHPYLAVW- ----HDPQDE PES---KIKF DFKTFETVDD MESMKQLI--
     863837  ATLR---RVG SERAQAYIRS LPRMPT---- ----IHFQQL YPR---ASPL ---------- ---------- --------AI DLLEKLLNFD PAARITVEQA LAHPYLAAY- ----HEEDDE PSH---EKVF DF-SFEVTDR IDEMKRLI--
      81355  DTLR---RVG SPRAQDYIRS LPIKPR---- ----ISFATL YPR---ANPL ---------- ---------- --------AI DLLSKMLCFD PAKRISCEEA LNHPYLQVW- ----HDPADE PVCEKVPVKF DF-SFEEEDN IDGMKKLI--
     152896  DTLR---RVG SPRAQDYIRS LPIKPR---- ----VPFQTL FPR---ANPL ---------- ---------- --------AI DLLAQLLHFD PAKRITCEQA LNHPYLAVW- ----HDPADE PTC---PSPF DF-GFEEEDS IEGMKRLI--
    1196002  PCSN---LLL YSQAQDYIRS LPIKPR---- ----IPFSTL YPH---ANPL ---------- ---------- --------AI DLLSQMLAFD PAKRISCEQA LNHPYFAVW- ----HEPSDE PVC---DVPF DF-GFEEEES IEGMKKLI--
    1195972  PCSN---LLL YSQAQDYIRS LPIKPR---- ----IPFSTL YPH---ANPL ---------- ---------- --------AI DLLSQMLAFD PAKRISCEQA LNHPYFAVW- ----HEPSDE PVC---DVPF DF-GFEEEES IEGMKKLI--
      49917  ADLD---RIA TPLSRNFFDI IKHPKGSKAK ATNGISFAHL YPE---ASTI ---------- ---------- --------AH DLLSRMLKYN PDKRATASEL LEHRYVELF- ----RQSYNK GSEPVCPKWM DD-SFEKA-S YDQLEVIL--
     116823
     271167
     151286
      97820
     167647
     430394
     266211
     266223
     266189
     151328
     800193  -------YVL PARSRTGSTA GGGEV----- ---TRRLLRT LSTVSIHESA ---------- -EGLAGG--- -----LAGIA PIGKFIVEPE -----TEADA PPSEMPRDFR VVDEEEEDEE REMTMRLAIG GGGKFHLP
      22041  -------YVL PARSRTGSTA GGGEV----- ---TRRLLRT LSTVSIHESA ---------- -EGLAGG--- -----LAGIA PIGKFIVEPE -----TEADA PPSEMPRDFR VVDEEEEDEE REMTMRLAIG EHTYNII
      22014  -------YVL PARSRTGSTA GGGEV----- ---TRRLLRT LSTVSIHESA ---------- -EGLAGG--- -----LAGIA PIGKFIVEPE -----TEADA PPSEMPRDFR VVDEEEEDEE REMTMRLAIG EHTYNII
      22020  -------YVL PARSRTGSTA GGGEV----- ---TRRLLRT LSTVSIHESA ---------- -EGLAGG--- -----LAGIA PIGKFIVEPE -----TEADA PPSEMPRDFR VVDEEEEDEE REMTMRLAIG EHTYNII
     448385  RTES---YIL PARSRTSSTP GGEV------ ---SRKLLRT LSTVSIHESI ---------- -EGLPGG--- -----LAGVA EIREYIVGKQ T----TEADA PPSEIPQEFG IDEGDEDEDG RDDSERDKNE SGKFRVG
     122008  YAD----YVM PARSRTMSTA TREVV----- ---SRKLLRT LSTVSIHETG ---------- -AGHPGG--- -----LAEIA PIGRYILEKR GPTEVTEADR PPSDLPGELG GTDEQKGAGE GGEEEEKEEK EGKSGKGKEK DKREIGKERR
     578295  GGS----FIV PARSRTASTV GGESMY---- --GTRKILRT LSTVSIHES- ---------- -MDSVGG--- -----LAAMG PIGQAIMERR E----TAADA PPSEMPREFD TVREE----E GEYRSFGTAS GSTSASVEGI SARPVGQEGR
     139503  QRDG---FIV PARSRTASMA TGYDV----- ---PRKLLRT LSTVSIYESL ---------- -GPLAGGGEG GGEGAASGVA PIAKIIRERR -----TEADA PPSEMPGDFQ IGVG------ GSDSESGESG DGK
    2468658  -------YVV PARSRTGSTV GGEPY----- ---TRKLLRT LSTVSIHERL ---------- -PAVALA--- -----RAQAI QEGKEAAESK -----SAGKD SGEDKEGKVE SEDVADPKAR LGRYIVEKSA TGADAPPSEM PREFDEVEKQ
    2215386  -------YVV PARSRTGSTV GGEPY----- ---TRKLLRT LSTVSIHERL ---------- -PAVALA--- -----RAQAI QEGKEAAESK -----SAGKD SGEDKEGKVE SEDVADPKAR LGRYIVEKSA TGADAPPSEM PREFDEVEKQ
      88816  GS-----YIV PARSRTASTV GAEGYFPGGS FIGPRKLLRT LSTVSIHESA ---------- -EGLAGG--- -----LAAMG PIGQAIINRE -----TAADA PPSEMPRDFG TVKEEEEQKT GGDGSGSSSA RSQKDKARNG GRRSGTASGN
      88949  GS-----YIV PARSRTASTV GAEGYFPGGS FIGPRKLLRT LSTVSIHESA ---------- -EGLAGG--- -----LAAMG PIGQAIINRE -----TAADA PPSEMPRDFG TVKEEEEQKT GGDGSGSSSA RSQKDKARNG GRRSGTASGN
      88917  GS-----YIV PARSRTASTV GAEGYFPGGS FIGPRKLLRT LSTVSIHESA ---------- -EGLAGG--- -----LAAMG PIGQAIINRE -----TAADA PPSEMPRDFG TVKEEEEQKT GGDGSGSSSA RSQKDKARNG GRRSGTASGN
     206606  DGEY---VVA MIRNRAPSTD AHSV------ ----GTLIRT LSTGGLNKLG ---------- -QQGAGA--- ---------- --GKDGVQPL -----TQADL PPSAMPVEFK NGRGTNGGGS K
     123647  -------YVV PGRHRTASMY TIGGSGSDIG AADVRRLLRT LSTVSIYESG ---------- -EGHVGG--- -----LADVA PIGKYIIQKD --------RT GDDALASEM- --PKEIRDEE GGSPSAQR-- --EKSGG--- DGKRLFRV
     143931  -------YVV PGRHRTASMY TIGGSGSDIG AADVRRLLRT LSTVSIYESG ---------- -EGHVGG--- -----LADVA PIGKYIIQKD --------RT GDDALASEM- --PKEIRDEE GGSPSAQR-- --EKSGG--- DGKRLFRV
     165009  QD-----YVV PARSRTGSLF MGEGS----- ----RKLLRT LSTVSIYESG ---------- -EGLAGG--- -----LADIA PIGRYIVEHD --------ET LPSEMPRELE SPPKEQKSPP NPGQNGQKTE SVEGSES--- GGRRECKA
     580315  -E-----YVV PARSRTASMF GD-AST---- ----RKLLRT LSTVSIYESG ---------- -EGLAGG--- -----LADIA PIGKYIVERD --------EA MPSEMPHDLD SHSQSPPQPK SGASTGSQKE G--------- -NEKRFHIQE
     492840  -E-----YVV PARSRTASMF GD-FSA---- ----RKLLRT LSTVSIYESG ---------- -EGLAGG--- -----LADIA PIGKYIVERD --------EA LPSGMPQELD S--QSPPQPK SGSSNGSRKD G--------- -EKR
     594182  -E-----YVV PARSRTASMF GDGTAT---- ----RRLLRT LSTVSIYESG ---------- -EGLAGG--- -----LADIA PIGKYIVERD --------EG LPSEMPQELE SPPQAQPQSQ THSSPKSQQS HSGKSS---- KDKARFHIHE
     168238  QE-----YVV PARTRTASMF GDGAAS---- ----RRLLRT LSTVSIYESG ---------- -EGFAGG--- -----LADIA PIGRYIVERD --------EG LPSEMPHELE SPPQPLALSQ TQS-PTSQGS GSGSGSEGSK KERARFHIQE
     107302  QE-----YVV PARTRTASMF GDGAAS---- ----RRLLRT LSTVSIYESG ---------- -EGFAGG--- -----LADIA PIGRYIVERD --------EG LPSEMPHELE SPPQ
      64718  -------YVV PARSRTISQ- -AAGMG---- -TFSRKVLRT LSTVSIHESV ---------- -DGLPAD--- -----LAHLA ---KYIKERD ----VSDADA PASEVPKDFD PIDENTGGNG GGGEGGDKCK FHIA
      64830  -------YVV PARSRTISQ- -AAGMG---- -TFSRKVLRT LSTVSIHESV ---------- -DGLPAD--- -----LAHLA ---KYIKERD ----VSDADA PASEVPKDFD PIDENTGGNG GGGEGGDKCK FHIA
    1154823  -------YVV PARSRTISQ- -AAGMG---- -TFSRKVLRT LSTVSIHESV ---------- -DGLPAD--- -----LAHLA ---KYIKERD ----VSDADA PASEVPKDFD PIDENTGGNG GGGEGGDKCK FHIA
     433002  -------YVL PARSRAAST- --AGGD---- -VSLRKLLRT LSTVSIHESV ---------- -EGLPCG--- -----LAGVA PIGQFITDQE -----SEADA PPSEIPIEFA -GKGGRGVYG DSGL
     822334  -------YVL PARSRAAST- --AGGD---- -VSLRKLLRT LSTVSIHESV ---------- -EGLPCG--- -----LAGVA PIGQFITDQE -----SEADA PPSEIPIEFA -GKGGRGVYG DSGL
     511572  -------YIL PARSRAASTT TAAGSE---- -IAVRKLLRT LSTVSIHESV ---------- -EGLPGG--- -----LAGVA PIGKFIVGQE -----SEADA PASEMPMEFS -IRTRRDGGK ELAI
     688421  -------YVV PVRSRAPS-- --AATD---- -VGARQLLRT LSTVSIHESS ---------- -EGLPGG--- -----LAAVA PIGKYIVART -----SGADA PPSEIPRDFG -VEESRNDEG H
      17778  -------YVV PVRSRAPS-- --AATD---- -VGARQLLRT LSTVSIHESS ---------- -EGLPGG--- -----LAAVA PIGKYIVART -----SGADA PPSEIPRDFG -VEESRNDEG H
    1336823  -------YIV PARSRAASMM GGQT------ -----QLLRT LSTFSIHEKV ---------- -D-------- ---------T ELGNLFSGQQ -----SAADA PPSSMPREFA NTDKENTEL
     605623  GGSG---YVL PARSRTASVI DMP------- ----RRLLRT LSTVSIYE
     511468  -------YIL PARSRAASTT TAAGSE---- -IAVRKLLRT LSTVSIHESV ---------- -EGLPGG--- -----LAGVA PIGKFIVGQE -----SEADA PASEMPMEFS IRTRRDGGKE LAI
      60300
    2435698
    1222436
    1172380
    1151825
     410058
      57461
     311323  ------RLPT STNTPFLNFP YFKLRACSAA LPSPNRLCTS ASSTSPSSKY ---------- -ARMANSSTP VRRRNAPPGK LILDRGKLAA CAAHTSSFHR RDWTSVTRPT TKSPISGTYR -------AER GVMDKSLLVR VRVPRREEDM
     143102  ------FFGQ PVSALVDSAV LKILGE-HKV LYGSQRLWGS ISNGA-NILL ---------- -VGLLISTMG INVAFYVFGV SVVCFIIIAL FTRFGGGNDS TLDSTLSQIN EESPLLKDQP RTTNYAIPPE QVEQNHTPVQ NSFFSALSQV
    2750129  ------EESS DDEPLPAPAP RPVLRRAHRS RHIAFKSMAT KDATA-VAEI ---------- -LGVAPTPSS LSSSASPMGT LTPTSDEPAT MEKLTRSTKS VADYFKERLA AKAAAKSGSS TPVPPSGSES AVEESTSREE SDAPRGGLGS
    2702932  ------EESS DDEPLPAPAP RPVLRRAHRS RHIAFKSMAT KDATA-VAEI ---------- -LGVAPTPSS LSSSASPMGT LTPTSDEPAT MEKLTRSTKS VADYFKERLA AKAAAKSGSS TPVPPSGSES AVEESTSREE SDAPRGGLGS
     108010  RDGSYMVNYT AAGGGAPQLP YPTRERKGHT PPQPVMSKGL KALLADRTFI ---------- -ESLPPSSTT VAPPSSEKAE SREASTSLST VLLSNTPDFM LVVSLKGLFL YVAPAVRRVL GYEPEELVGK AITDYCY-QH DIVPLTRELK
    1754091  EDEGELKRNF LFGIGADIC- ---RRHKEDG QPGHIVNEVQ DKVVQDADNS ---------- -EGTGFWTVM EAARRHVSAP TI-SSAHFFR IASADRAQRM EVVEALGQAT GAARKQQVDE KFKEEFIEDL RLAAYCACLA SFAQGMNLIA
     564944  -----IMVPN LGGVMKPELL --ARDAYTCL KTGQIDLSAP PAMSGEREDL ---------- -QCARICHMP LTEYHKEWLP E--TSQWVAD FYEHYLGVDI SAIPMLRAEN YLLLSPQSCE LFY-QWIWTL KPTEIPNRYQ VIDSRRENDG
    AtMPK18  -QLNNNTHMA IDAKLLQAQS QFGPAGAAAV AV---AAHRN IGTISYSAAS
    AtMPK19  ------HKVG IDAKLLHAQS QYGPAGAAAV AV---AAHRN IGAVGYGMS
    AtMPK20  --ENISDRII IDTNLLQATA GIGVAAAAAA AAPGGSAHRK VGAVRYGMSK ---------- -MY
  OsMPK20-1  PKSDSSERLA AETNLYTRSL N-GLAATAAG VA--ASAHRK VGVVPYGMSR ---------- -MY
  OsMPK20-4  -KSDVADRAA LQSNMMQGIG PFNGIAAVGG ------NYNK VSTVQYGVSR ---------- -MY
  OsMPK20-5  PRTDLTDRAA LQGSMLYGIA PFNGIAAVAG ------GYSK VGAVQYGVSR ---------- -MY
  OsMPK20-3  PVPAVSYSLP GTYRIT
    AtMPK16
  OsMPK16-1
    AtMPK17
     AtMPK8
    AtMPK15
  OsMPK17-1
     AtMPK9
  OsMPK21-1
  OsMPK21-2
  OsMPK17-2
      30260
     130090
     599215
     219362
      56051
     139809
       1060
     184799
      68008
      99901
     104502
     645859
     599559
 36518:4-24
     178963
     196817
     738557
    1159263
     176895
     664529
    1194920
     432994
      98579
      88826
      54157
     914084
     503422
      95205
     229298
     455126
     256867
     419576
      74409
     521809
      54281
      48349
     594110
      27538
      96623
     380926
     263236
     154671
      67562
     226444
    1137209
    AtMPK13
    AtMPK11
     459393
      69938
     591714
```

```
    107905
     99046
    863830
    287494
    662188
    492594
    176887
    618168
    492643
    125995
    122928
    154756
     68423
 OsMPK20-2
 Consensus  ....    .i. s.r...y... lp....         .pf... f..  .a.p.                            a. dll...l.fd p.kri....a l.hpyl...      hdp.de p.... .... .f..f..... .........

            1351                                                                                                                                                  1500
    127228  --RKFLFLLW FHCHS
    287090  --RKLP
    186494  --QMLYDEIA H
    260272  --QMLFDEIM H
    119975  --RRWKLNK
      2147  --CKWGKANC ATDMRAGIV
    692376  --LLIYQEIM R
    698785  --LLIYQEIM R
    468116  --VLIYQEIM R
    274271  --QLIYEEIM RS
    255486  --RLIYEEIL R
    285227  --RFIYDEIM R
     18186  --QLIYQEIM RDPIEP---- --PFPRPQNQ LNPPLTSHRS PAGESSSKHA LGADLLVLCR AQPRP--PAS DAGFLFAELP AAEEAAQIKT ILTTRGRGSR GCPSLRVTAG RLGLASQ
    465108  --QLAEKLQA PLRANYEHSS VIVVPCPDLR ESPFLLAHQG LSGDEKAADI GGQTHLYPRP MKDKTYQLGQ IAREMEMIVD GGPYPPPAVT FLGAGAGSYD LVGQNCEMVF NFGMRYADMC LLRSNNGRYV RIEPQTKDVL VENTDQHKWE
   1164585  --QLIYQEIM RGPTTMWTGL IRRFSTEESR DSGDFHAHAG GPALSNSSND G--VHDVFTP RQPRTLSPFR PPPLDPVVLH GYKDSTPPES RLLTPAVAEE IRTMVPERL- RISEDWRLVY SLAQNGTSLA TLYQQCR--- -------QYE
     93851  --RLIYEEIM RRPGITSQDA PTRTTFANVP DWMEPPMAVT AKRISDTIDW AEQQVRTVGN GDDDDDNEND LVALRERIAG AQAAQAQAQI QAQAQAQGGG EKL
    108267  --RLIYEEIM RDHFIA---A PMQYDRRN-- STLIDPASSP RKHFSPPISL GDGDIPICYD NDDDQDDSHD DSGAHGTLWK GRQCFSR
     59022  --SSRKTKLR EREGSL---- --ICPSGISQ RDILLPDSRS DQEVRESGIW LSYLCGHLGK REISRAPDWA GGVRLWRSFD
    370137  --RDYAVNAF EHAVSSYLTS LTDGRYEGPS ASIFHICVDL SRLV
    532216  --RDHAVNVF EPFNIIMLDS LHGSTWSTIR ARWVSITGIY PGFVGILASS
     31240  --GKKSCCIQ AGSDYLLYSQ CSFSRRSCDR FVSCYARSRS YA
    160345  --REYHPDNL LKETSLSPDT KSFVTSVDLP GDYAVNI
    212060  --REYHPDNL LKETSLSPDT KSFVTSVDLP GDYAVNI
    327005  --RKLRNPHL TNRFN
     48412  --RKRRINEI QCSANKPNQV
    606069  --ELIYQEIM R
    123724  --QLIYQEIM R
    342035  --QLIYQEIM R
   2135321  --QLIYQEIM SYASKTPATP ATFPESPMFD
    644393  --QLIYQEIM R
    784446  --QLIYQEIM R
    567834  --QLIYQEIM R
    389770  --QLIYQEIM R
     63860  --QLIYQEIM R
    131603  --QLIYQEIM R
    495859  --YLMYEEIM R
    434551  --YLMYEEIM R
    140828  --QLIYQEIM R
     66435  --TLIYEEIM RTTN
    383913  --LFIYEEIM R
    323815  --LFIYEEIM R
    396599  --VLIYEEIM R
     74447  --CEFAFILV LAAFSCLWID L
     83363  --REFNRFFL YEIYLLIHYS LYLRGNHALN LIDCFN
     50905  --TLIYEEIM RTTN
    650405  --STSFHGLL SCAD
    493847  --TLIFEEIM R
     95409  --GLIYEEVM GHGGGQA
    835420  --GLIYTEVT GTGVNFN
    343609  --QLIYDEIM RPI
     67554  --GMLIYARF DLN
    216015  --RMLYEEIM KPL
     52193  --KMLYEEIM KPL
     53916  --KMLYDEIM KPL
     83931  --KLLYDEVM KPLQYD
     51986  --AMIYNEVM TPL
     49589  --SMIFNEIM RP
    862988  --ELLYEEIM SFQPAPMT
    833500  --ELLYDEIM SFRPAPIT
    810703  --ELLYEEIM SFCPAPIT
    457677  --ELLYEEIM SFCPAPIT
    161161  --ELLYEEIM SFRPAPMS
   1588194  --ELLYEEIM SFRPPPMS
     98485  --ELLYEEIM SFRPPPMV
   1443964  --ELLYEEIM SFRPTMGA
    864221  --ELLYEEIM SFCPAPIT
    903905  --ELLYEEIM SYNPQPIT
   1147795  --ELLYEEIM SFRPTMQ
    622735  --ELLYEEIM SFNPVPMTAQ
    166786  --ELLYEEIM SFTPVVMTSS
   2641010  --ELLYDEIM SFNPAPIT
     28679  --DLLYQEIM SFRPAPIS
    101027  --ELLYEEIM SFRPAPIT
    564549  --ELLYEEIM SFHPQPIT
    227465  --ELLYEEIV TFKPTI
     18095  --ELLYEEIM SFRPAPIT
    338545  --ELLYEEIM SFRPTMQ
    134728
    275037
    503339  --QRAPL
     40745  --RLLFEEIE SQFQSSCIFY FALC
     43876  --RRMYDEVT GFQPLV
     14716  --RRMYDEVT GFQPLV
     15138  --ELLYEEVV AFQVSLLFLS FAILFEPVSL TMTSACFISL LSRGANALRP
     67488  --QLLFEEIM TFKPIC
     28680
    177514  --ELLYEEVL AFVPSINAPG ESHNANN
     53880  --ELLYEEVM SFQPSI
    778168  --GLLYEEVL SFVPSI
    388025  --ELLYEEIQ SFVPSI
    581713  --GLRPSTLL TSFLTAASRA SVRGNRLFHT VHLSV
    229317  --ELLYEEIM SFTPSI
    829657  --ELLYDEIM SFIPAI
     70959  --ELLYEEVQ SFVPCI
     32857  --ELLYDEVQ SFVPCI
    561172  --KRLYDEVI SFQSLI
    573944  --QLLYDEVM SFQSLI
    740770  --QLLYEEVI AFEPQQALAP LTSAWAVGSL PEASEVRPGK GSG
     16071  --TLLYEETI SFKSAL
   1126092  --TLLYEETI SFKSAL
    478842  --ELLYEEVT AFQSLL
     46649  --ELLYEEVM AMPSYI
    601377  --ELLYNEVH SFTPAI
    866574  --VLIYQEVT KPPSQMSAYL ASGYS
    521401  --VLIYQEVT KPPSQMSAYL ASGYS
    850251  --VLIYQEVT KPPSQMSAYL ASGYS
    832111  --ELIYNEVT SSRTQPPNSF LCMADATP
    832110  YAPIFYLVLG HDLYNSTCLS ITLINTTNRV DIQRSHLFKD SATELFLVYG RCHTITMFRM GVGTMTLEEL FWVMRYIWVE NFFNFMFATR CFLFSV
    676666  --VLIYKEIT SPRPQRAYPS MSEDER
   1143376  --VLIYKEIT ASRQDTSMDV DDGP
    457401  --RLIYAEVI AASPQAASLS VSEI
    834255  --VLIYKEIT NAQASQPSLL LAKDA
    133042  --VLIYEEVT RPRAQ
     30507  --GLIYEEVT RPGS
    646041  --VMIYEEVT KPRAQPAQ
    570964  --VLIYEEVT KPRPPPGTQ
```

```
     301734  --LLIYQEIT TPPPNHAYSS Q
     154378  --VLIYNEVM SAHQYPQQQQ IQIPSPGVPT NGGLNLMGSG S
      90189  --VLIYNEIM RPDTGPAPIT MMNNFPS
     448371  --LRIYEEVL QTPTYPIDAT AMQM
     476779  --LRIYEEVL QTPTYPIDAT AMQM
      68819  FSLSFLFRTP P-SPPLSLLH PCFRSLMSPD FFFVLFGFVF LPARRIDIRG GDEHASASAA GGYRGGCARG GDVVRGDQCS AVIYFLFLSI HENKSGFR
    2671102  SVPPSARR-- P-SADLRAWR LAYKGLLCDE YLLFFQDLGG MSADD
     940143  FTVFNPHARI DRLPPELIYQ EVTRSFATAS
     585786  ---------- -----ELIYE EVTRPLAPSG S
     349085  --ELIYQEVT RSFATAS
    1539717  --VLVYNEVT RPFPPRPIPP QQPQPS
      24638  --ILIYQEVM KPIPPQLEA
      75000  --ILIYQEVM KPIPPQLEA
    1245527  --VLIYEEIM KP
     103018  --ELIYEEVC H
      43883  --RLIYDEIT SPPPQHS
      58913  --QLIFNEVM N
      18326  --VLIFEEVI RPSNHQQAVM VDTPSTPVGP SAATPIAPNP DSAMALS
     301059  --VLIFEEVI RPSSHQQAVM VDTPSTPVGP SAATPIAPNP DSAMAIS
      12410  --VLIFEEVI RPSSHQQAVM VDTPSTPVGP SAATPIAPNP DSAMAIS
      18327
     184754  --MLLYEEIT KK
      99455  --KMLYDEIM SR
     706256  --KLIWNEIF S
     653342  --KLIWNEIF S
     462249  --AAPTKKVR NMSHFRNVPV RCIIRNVKLQ LEKFATLSNP DSGSESKYVL AQPNPTGYSG HIPPTLKLAP TVSMIFQGLL LLEKPTMLMK KM
     326973  --LLIYNEIM R
      77855  --QRLSSPPF LLLFGKGVYM ICIPFVSS
     338563  --ELLYEEIM SFRPTMQ
     233520  --ELLYDEIM SFRPAPIT
      73610  --ELLYEEIM SFRPAPIT
     150924  --ELLYEEIM SFRPAPIT
     348809  --ELLYEEIM SYNPQPIT
     913771  --ELLYEEIM SFRPTMGA
     913918
     747578  PIEMLKDMLY DELMKTME
     458977  PIEMLKDMLY DELMKTMEYR STRNAAS
      41459  DGRPVHKDEL SKADLKELLY EEVMREEETL VKTTKAHALK QCDQLVKDFA ACSEGRTISV AWACKDKWKG VQDCISQYTG PEAMTKVRAE YLRLRNEKIV QDVAAAGRAS GTGQANTRKH AERKVSANCL REVSQNSELG PTSGAAALMY
    1397463  --------EL NKEELKQLLY DEVMSFNSVI
     149203  ------SHSI PSLTHQTAVY LLVLRRADRS PRIDKELNKD ELKQLLYDEV MSFQSLI
     102078  --TYLYDAIL EMTRSRLK
 36766:44-3  --RQLYREIK QLRG
      54278  --RQIYNQII TAKRD
      29185
     790328  --VLIYQEVT KPPSQMSAYL ASGYS
     765763  --VLIYQEVT KPPSQMSAYL ASGYS
    1491990  --VDNIDGKL DPDVR-DRFV TTWFSVHDDM RWFFLREFAI ILRSSKPQTN AHLPENLLSI LERLTTMPTD VSELNEFWIP SFARAPPKAF LPPQEETEDA EAEDDWRTFF DTPDAPDTSA DQFVKGQSQR VYRLSTHQNP FDAKEPSPYI
      32998  --ELLYEEIM SFRPPPMV
    2601990  --VLIYEEVT RPQPFQLSEE A
     262460  --RLIYEEIL R
      11717  --DLLCEDIE QFHPPVPVSV APIPLNTAAS RFFRMGMTAS AS
     532299  --NLLCEDIE QFHPPVPTSI APLPLGAAAS RFFRMGMTAS AS
     551831  --NLLCEDIE QFHPPVPTSV TPMPLSAAAS RFFRMGMTAS AS
     301484  --NLLCEDIE QFHPPVPTSI APLPLGAAAS RFFRMGMTAS AS
      42366  --KLIVEEVN SFRAEVRAQ- ---------- --------AR AAGQVRKQDS LPIPSRD-EI ISSPVQEHAP RNGATSGYTG ARATAPEPIM DDPSAELEKE LSRQNLGK
     476004  --RLIWEEMR HFHPVDGEEP GVSATAG--- --------EG ENDSFSTTQA SNTPVTPVTP ATAEQDNTPS S----ENTVN ATTAEVEV-- STPATEEARP EDDGEAA--P SVDEKQSTNS DHKIVRTSVG SDNPTDAQTR QEAGEPAREV
     558476  --RLIWEEMR HFHPIEGEEP GVSSNAAA-- --------EG ENDSFATTQA SNTPVTPVTP ATAEQDNTSS SSSSSEATGT ATTAEVEVEV TTPATEEARP EDDGEASTRP TGDDKQSTNS DQKIVRTSVG SDNPTDAQTR QEAGEPAREV
       9954  --ELIFEEIL QIHPDAPRSP IKVPHAGLSS P----PTEQV LSPSTTRPHA QS
      82136  --ELIFEEIL KIHPDAPRSP LKTPSTGLTS P----PAEQV LSPSTTRSHG QD
     312415  --ELIFEEIL KIHPDAPRSP LKSPPAGLTS P----PAEQV LSPSTTRSHG QD
     354114  --ELMFKEIC HFHPE---EM VKRAKQQREN P----ETVEK LPPGWVKRES RSVPGKFYYS NPKRGISTWI KEEMD
      34134
      20005  --EELQRLVF QECMSIHQLE ARHMQ
     518921  --EELQRLVF QECMSIHQLE AHHMQ
     503206  --EELQRLVF QECMSIHQLE AHHMQ
      10810  --EELQRLVF QECMSIHQLE ARHMQ
     AtMPK1  --EMMWNEML HYHPQASTLN TEL
     AtMPK2  --ELMWKEMI HYHPEAATIN NNEVSEF
     OsMPK7  --EMMWQEML HYHPEVVAGV NM
    OsMPK14  --EMMWHEML HYHPEVVAAM SAR
    AtMPK14  --EMMWEEML HYLPRA
     AtMPK7  --EMMWNEML YYHPEAEISN A
    AtMPK12  --ELIYKESV KFNPDH
     AtMPK4  --ELIYRETV KFNPQDSV
   OsMPK4-1  --ELIWRESL AFNPDPPY
  OsMPK16-2  --EIIWREAL KFNPEPIH
     AtMPK5  --ELVWLESV KFNPLPSI
    AtMPK10  --ELIYCEAL AFNPETSND
     AtMPK6  --ELIYREAL AFNPEYQQ
     OsMPK6  --DLIYQEGL AFNPDYQ
     AtMPK3  --EMIYQEAI ALNPTYG
     OsMPK3  --QLIFNEAI EMNPNIRY
     206269  ---------- ---------- YSEILEFHNV DNVDGAQYLG TPLTGPKLEA QDAVAFQQ
     154265  ---------- ---------- YSEILDFHNV DNVDGSQFLG TPLTGPTMGN PDGATVDGNN VPTTSNNNFP AQQ
     168032  ---------- ---------- YSEILDFHNV DNVDGSQYLG TPLAGPV--- -DGQ------ --DTAYQQ
     307550  ---------- ---------- YSEILDFHNV DNIDGTQFLG TPLTGPTMEN PDGNI----- --DTSTFQQP SQQ
     320251  ---------- ---------- YSEILDYHNV DA-TNGEEVT NGE
     356517  KTGIRKVWSD QARLTSFDYR YSEILDYHNG ED-AGSAKDK PEEPGEERMG WFSGEESSGE DTWDTLTRTK SDEKT----- -RYHATRQGS GRGKVSDEQE
     113434  ---------- ---------- YSEILDFHQV SD-PGAADG- -EAPSGALAG P-EGQSRLGN GNVEHIGK
      28887  ---------- ---------- YSEILDFHNI ENTPDANEGP VAEQSETPLS PFNPTQTMAI QNQLTFLLLL LEIATFAILI VPLPFTWRRA LFKTIAESQI IAKVQYGLKI TFIFVALLFV DAVNQMLKIQ REKEVMHEQG TVQTDLRSQT
      16635  ---------- ---------- YSEILDFHNV DQTVEEGEAS GQLAGPQ
       6923  ---------- ---------- YSEILDFHQV GEGEGSEHAT VPITSMPNGE IPKGAVIAAQ PVPAQ
     807502  ---------- ---------- YSEILDFHQV --GDP----- -TDPNNQNYQ NYPNYQDPSM GLVQ
     129058  ---------- ---------- YSEILDFHQV --GET----- TTSSAGIPEG ALAGPEAPSA TPAQAK
     359241  ---------- ---------- YSEILDFHQV --GEA----- ITSAGGIPEG ALAGPQTTAA AVPPTE
      53282  ---------- ---------- YSEILDFHQV --GEQKPNDL TTTAGGVPEG ALAGPEEEAV PEAASPAEPK SE
     117789  ---------- ---------- YSEILDFHQV --GEA----- TTNSAGIPQG PLAGADDGYV PSGASATAQT T
     170586  ---------- ---------- YSEILDFHQV --SDP----- GAADGEAPSG ALAGPEGQSR LGNGNVEHIG K
     162119  ---------- ---------- YSEILDFHQV --GDS----- -NLTGSVPEG ALAAPEDPNV PHPEAAQIAA TAE
     560244  ---------- ---------- YSEILDFHQV --GDS----- -RLNGGVPEG ALAGPEDHAA TAAPAA
     499279  ---------- ---------- YSEILDFHQV --GDS----- -RLNGGVPEG ALAGP
     898207  ---------- ---------- YSEILDFHQI --GENALEGD SVPEGALSSE GMEEGKAPIA TIPAQDAPVP TGEVTAGGN
     500359  ---------- ---------- YSEILDFHQV --GDST---- LLDNNNIPEG SLAGP
     390416  ---------- ---------- YSEILDFHQV --DAS----- -VIDEAAPDG SLVG
      61586  ---------- ---------- YSEILGTS-- ---IFC-SRS LCVINPYSRP NHRFPPSWRD SNLVGRDTRG CSSGT
      74488  ---------- ---------- YSEILGEQKR NHGCSCRSLS LIVFAFFPRL VLADFHQIGD AALENADLAG APLGAMEGTP TAA
     155373  ---------- ---------- YSEILDFHHV --DEAQSNFM GDVQQQQVPG QAAQAPFA
      56185  ---------- ---------- YSEILDFHHI --EDSTDS-- GPVAGATPQQ QQA
      14909  ---------- ---------- YSEILDFHNI --ENTPDANE GPVAGAVPSP AA
     176707  ---------- ---------- YSEILDFHQV GEGEG----- -SEHATVPIT SMPNGEIPKG AVIAAQPVPA Q
     176721  ---------- ---------- YSEILDFHQV GEGEG----- -SEHATVPIT SMPNGEIPKG AVIAAQPVPA Q
     240717  ---------- ---------- YSEILDFHQV GEGEG----- -SEHATVPIT SMPNGEIPKG AVIAAQPVPA Q
      56428  ---------- ---------- -SEILDFHNI D--------G GVVSPE
      97915  ---------- ---------- -SEILDFHNI D--------G GVVSPE
     707387  ---------- ---------- -SEILDYHNV D--------A AQATEENSS
     504013  ---------- ---------- -SEILDYHNV D--------S QAPAEEN
      37843  ---------- ---------- -SEILDYHNV D--------A AGEMENGGS
     507853  ---------- ---------- -SEILDYHNI D--------S QGTTEVPPAE NGSS
     338747  ---------- ---------- -SEILDYHNV D--------A AQQAGE
      49060  ---------- ---------- -SEILDYHNV D--------S ANN----GEG QENGGA
     147551  ---------- ---------- -SEILDYHNV D--------Q GNDPTAAGGL TENGAA
     286873  ---------- ---------- -SEILDYHNV D--------S ANGEPDPHGE LTGAA
     193904  ---------- ---------- -SEILDYHNV E--------A SDVEGAAEDN EAVP
     189288  ---------- ---------- -SEILDYHNV D--------N QQA
     198339  ---------- ---------- -SEILDYHNV D--------G V
    2140409  ---------- ---------- -SEILDYHNV E--------A ASVGQMEDQQ YLAQ
     642177  ---------- ---------- -SEILDYHNV E--------A ASVAA-QDPQ FLAQ
     498471  ---------- ---------- -SEILDYHNA E--------A ANVAQ-HEQQ FLGQ
     784216  ---------- ---------- -TEILDYHNA E--------A GVAAHQLDEG QYGMAQ
     128268  ---------- ---------- -SEILDYHNV E--------A SGQMMFPDEV SISQ
     246689  ---------- ---------- -SEILDYHNV D--------T DMSQL--DEQ MNGQ
    1081594  ---------- ---------- -SEILDYHNI E--------G GVPNM--DEQ
     603877  ---------- ---------- -SEILDYHNV E--------T GGDNHVMQEF TNGQ
```

```
 140829  ---------- ---------- -SEILDYHNV E--------N DAIGQPVEGF SNGGQ
 327107  ---------- ---------- -SEILDFHNI E--------Q G-NEAGEALV QGAVQGDAGQ AFA
 329694  ---------- ---------- -SEILDFHNI E--------Q G-NEAGEALV QGAVQGDVGQ AFA
 393439  IRSLFLFLLL TLLRY----- -SEILDFHNI E--------Q G-NEAGQALM EGAVQGNNTQ AYA
  38942  ---------- ---------- -SEILDYHNI D--------Q TLPEAGQLPA DGAEGASQPV S
  87588  ---------- ---------- -SEILDFHNV D--------Q T-VEEGEASG QLAGPQ
 443298  ---------- ---------- -SEIKLQHVQ H--------L KLPHGGQGVM KEEGGSLLFT YWGTNNKVSK SKSLMGLSA- PVLLMCASLL CLIALT
  53587  ---------- ---------- -SISRQASEP S--------A VAGTLHQYTN EAMVVGYDLD ARGRYVASGA GVNHVARSGR PRCFMNRQVL CKLDRRKC
  70072  ---------- ---------- -SEILDFHQI E--------T PAETAEETPS CTLKGLSFGS RQRSKSAHGL SGWVQERQSM YISSPHDARS FSVEIRTHAR DMLVH
  13822  ---------- ---------- -SEILDYHNV
  25364  ---------- ---------- -HEILDFHNV D--------G QNYADNSEIQ KSFQ
 116576  ---------- ---------- -SEILDYHNV D--------G VAEAAA
 103311  ---------- ---------- -SEILDFHQM NGA--DPSSA VPTETGQVPV NEAGETSQV
  26173  ---------- ---------- -SEILDFHQI GGVGEEAGQS VTQEEIAHIQ QDGIQAPQQP QEQQVE
  55352  ---------- ---------- -SEILDFHQN HG-----GIE LQNQDSQQLS QDIVNVPQH- QDQQVELKYD
 125207  ---------- ---------- -SEILDFHRI GGSDGQIDIS ATFDDQVAAA TAAAAQAQAQ AQAQVQLNMA AHSHNGAGTT GNDHSDIAGG NKVSDHVAAN DTITDYGNQ- -----AIQYA NEFQQ
  67307  ---------- ---------- -SEILDFHRI GGSDGQIDIS ATFDDQVAAA TAAAAQAQAQ AQAQVQLNMA AHSHNGAGTT GNDHSDIAGG NKVSDHVAAN DTITDYGNQ- -----AIQYA NEFQQ
 185764  ---------- ---------- -SEILDFHQV DSGFDQSNSN DQQGQQQDEQ EVVKSIA
  99366  ---------- ---------- -SEILDFHQV DNNGGVH
  50111  ---------- ---------- -SEILDFHNI SQDGDILQPN SGEATENYVQ ANGAPFNV
  87970  ---------- ---------- -SEILDFHDV KEPQTEGLAA PAA
 107532  ---------- ---------- -SEILDFHDV KEPQTEGLAA PAA
 604754  ---------- ---------- -SEILDFHQV GDSTLLDNNN IPEGSLAGPG EDGNIVGAGG GVQSVSVASA PTQA
 633130  ---------- ---------- -SEILDFHQV GDSTLLDNNN IPEGSLAGPG EDGNIVGAGG GVQSVSVASA PTQA
 181927  ---------- ---------- --NTLRLELA PFGVQVILIV TGGVKSRITR TERVLDEDSV YLPISAEYGR RVYHPQEGAM PNEAYAASVA KQILKKNPPK --WFWQGNRA W----IIWFP DRFFPRSIWD WIFPSMFGLA RLRQILAHNK
  69161  ---------- ---------- -SEILDFHNV NVDSEQEMTP STTTAGAS
  35549  ---------- ---------- -SEILDFHNI DQGAEAETVE GALAA
 230139  ---------- ---------- -SEILDFHNI EQGNEAGEAL VQGAVQ-GDA GQAFA
 193325  ---------- ---------- -SEILDFHNI EQGNEAGEAL VQGAVQ-GDV GQAFA
 430049  ---------- ---------- -SEILDFHNI DQANEAGQAL VAGASHSGMA PQTYV
 479652  ---------- ---------- -SEILDFHNI DQANEAGQAL VAGASHSGMA PQTYV
 986652  ---------- ---------- -SEILDYHNI DQTQGEAENG GS
 295607  ---------- ---------- -SEILDYHNV DAAQATGEEN GNA
 114099  ---------- ---------- -SEILDYHNV DSANGEPDPH GELTGAA
 265170  ---------- ---------- -SEILDFHNV DNIWFTTNNI TREGTLWGSR HKNEQ
  41804  ---------- ---------- -SEILDFHNV DGGMVDINQQ LQPEQQQPAQ QQQEVEGENA ASYDGGLP
 500846  ---------- ---------- -SEILDFHQV GEPAPTEGAL AGPDASPPTN FPSASVSSIS SE
 341622  ---------- ---------- -SEILDYHNI DATQQSENGS
1545705
  15276
 391543  ---------- ---------- -GEVLGFHEQ MTESGQSGPM KTTSQLDGMD LG
 346451  ---------- ---------- -GEVLGFHEQ MTESGQSGPM KTTSQLDGMD LG
 449365  ---------- ---------- -AEVLGYHEQ A-----GGPF QLTGPFDGMD LG
  54661  ---------- ---------- -SEILDYHNI DQTLPEAGQL PADGAEGASQ PVS
 275309  ---------- ---------- -SEILDYHSG SCDWQYDHVP PTGKVQNMLA EEMGGTY
 389883
  61624
 173235  ---------- ---------- -SEIIDYHNV ASKP
  35612  ---------- ---------- -SEILDFHNI DQGAEAETVE GALAA
  35597  ---------- ---------- -SEILDFHNI DQGAEAETVE GALAA
 391476  ---------- ---------- -WKDHNTQGS IFTLWAHLNH DLLPAVFSDR VRVIGARPRR GSPIFEERVP DPHSRAVRAS YHQKSTKKED DHGPNHAKSP FPSASAFPVV E
 335269  ---------- ---------- -WKDHNTQGS IFTLWAHLNH DLLPAVFSDR VRVIGARPRR GSPIFEERVP DPHSRAVRAS YHQKSTKKED DHGPNHAKSP FPSASAFPVV E
 377162  ---------- ---------- -SEIMNHHNS QISEDLIDFQ SLLNE
 271149  ---------- ---------- -EQLYEYQDE TS
  89784  VPVSSRSSTL TGVR------ YSEILDYHNV EASDVEGAAE DNEAVP
 114108  ---------- ---------- -SEILDYHNV DSANGEPDPH GELTGAA
 173207  --------ID EIMRFRRKQW QLEQCLRS-- ---SSMHADM CCGVGTLGSS NIILRHQQQH QRHATMSLLD RTAIQWQYPT TDHTPPSPPP VDHQYDDT-- ---------- -SVLVTEEGS SMFSGGLVGE PEEMTAEEDA LLNQTAGMDV
 127887  --------VH EVMHSKGLLK GRNRRLLS-- ---VEPIDT- -----DMGAS NKRPRRRYTG SSISTPHSAS ATEAHQHAMA AVQQGREITV QDTEMD---- ---------- -----DDGGS GMITDRFVGE PEDMDEEDFK LMDSDDS-IR
  29226  --------ID AVKSTKPENT PSNRTESR-- ---TNSLQP- -----P-SEP HFQPQRRYTG SSISTPTTAS ATEAHLNAIA AVQQGKDFPI QESDNC---- ---------- -----AEGML -VGSNQFVGE PEDMDEDDIK LMDSDDS-LR
 167765  --------IE QVMQSKGADI SLHAPPLP-- ---LYRTST- -----ASSSS H-QTSRRYTG SSISTPSSAF TTEANLNAIQ LAQQGKDVTM ATGNTATA-- ---------- -AGNPQMTQF MVMSDQFVGE PEDMDEDDIK MMDSDESDIH
 150384  --------IE QVMQSKGADI SLHAPPLP-- ---LYRTST- -----ASSSS H-QTSRRYTG SSISTPSSAF TTEANLNAIQ LAQQGKDVTM ATGNTATA-- ---------- -AGNPQMTQF MVMSDQFVGE PEDMDEDDIK MMDSDESDIH
  57923  --------IE EVLKSKGMSD D------D-- ---VSRLAI- -----NHSAG H-QSHRRYTG SSISTPSSAS IAEANLNAIH AAQHGKEITI Q--NTA---- ---------- -----QDCHQ FIIGETFVRE PEDMDEDDIK QLDSDES-IG
  57928  --------IE EVLKSKGMSD D------D-- ---VSRLAI- -----NHSAG H-QSHRRYTG SSISTPSSAS IAEANLNAIH AAQHGKEITI QNTA------ ---------- -----QDCHQ FIIGETFVRE PEDMDEDDIK QLDSDES-IG
 230955  --------VK EVHAFEA--- ---------- ----NRSIS- -----SMSTG SSYSRRRYTG SSIATRRSTQ LAEANEQAAA SINENASL-- ---------- ---------- ---------- FTNQSHLVGE PEDMIESDGE EPPP------
 172033  --------VK EVHAFEA--- ---------- ----NRSIS- -----SMSTG SSYSRRRYTG SSIATRRSTQ LAEANEQAAA SINENASL-- ---------- ---------- ---------- FTNQSHLVGE PEDMIESDGE EPPP------
  40306  --------MQ EVMDFRKIVR QPVEMQKQ-M
 693957  --------LA EVNRFRHSVR VQPGQQGA-G ---QGQQP-- ---GQAVPIP NNYDRAGYE- ---DPRPHEA VVQQN-WQGG NDLERDLQ-G LDARGMR
 508525  --------LA EVNNFRRAVR MQPGQQAQ-G ---QGQQP-- ---PQPVPMP NNYDRSAYQ- ---DPRPHEV GAQGN-WEG- NDLERDLQ-G LDGRSMR--- ---------- ---Q
 471145  --------LA EVNNFRRAVR MQPGQQAQ-G ---QGQQP-- ---PQPVPMP NNYDRSAYQ- ---DPRPHEV GAQGN-WEG- NDLERDLQ-G LDGRSMR--- ---------- ---Q
 480536  --------LA EVNNFRRAVR MQPGQQAQ-G ---QGQQP-- ---PQPVPMP NNYDRSAYQ- ---DPRPHEV GAQGN-WEG- NDLERDLQ-G LDGRSMR--- ---------- ---Q
 471745  --------LA EVNRFRHEVR VQPGAGGA-G ---QAS-Q-- ---QPAVPIP NNYDRAGYE- ---DPRPQET MMQGGVWQG- NDLERDLQ-G LDARAMR
 687176  --------LA EVNRFRQLVR VQPGQQVQ-G ---QGQ-P-- ---GQAVPIP NNYDRGVYE- ---DPRPHEA MMQGG-WQG- SDLERDLQ-G LDARGMS--- ---------- ---NL
  34863  --------LQ EVRQFRQSVR APQQQQYS-Q ---GHQQG-- ---Q-QVPIP DGYD-RTHE- ---DPRPQEM GSTDPYG--- --LERELHDG LDAMR
  57153  --------LN EVRQFRHSVR APQSMPYS-Q ---QQIAN-- ---QTQVPIP EGYDPRAYE- ---DPRPQEM IQRGQ-G--- --LEGELAGG LDANMR
 147961  --------LD EVRRFRQSVR APQY--HS-S ---QQAPP-- ---QQQVPIP EHYDPRAFE- ---DPRPQEL GQGPAAGGQQ HDLERELQ-G LDQMRQ
 287354  --------LD EVKRFRQSVR TPQHLQYQ-Q ---QQGQQ-- ---PQHVPIP NQNYGAGYE- NGGDPRPQEM -QGHTAG--- -DLERELQGG LDMR
 281614  --------LL EVRNFREYVR APQQMQHP-Q ---NMHSG-- ---Q-QVPIP DNYSRQQHE- ---DPGPQEM GMKQSH---- -DLEGELQDG LDAHRRR
  29684  --------LE EVYRFRQVVR TVPGSGGG-Q H-AGQQGG-- ---ASQVPMP SGGATGQWR- -TEDPRPEEY M---GQQP-- GGLEADLAMG ADGRRSY--- ---------- ---RYLLSES DRNSDAYRRK EYSEEVTDCV VEDLSPAQQD
 144400  --------LD EVFRFRQLVR TVPG-GQG-Q G-GAQPQI-- ---PGQVPMP SDVHNQQWT- -AEDPRPHEY ----GAYN-- GGLEAELGGI QPWEGRL--- ---------- ---EKQASKA DREPFSMFGQ IPAEE---SM VSHAATSLTH
 136175  --------LD EVFRFRQLVR TVPG-GQG-Q G-GAQPQI-- ---PGQVPMP SDVHNQQWT- -AEDPRPHEY ----GAYN-- GGLEAELGGM
 126818  --------LD EVFRFRQLVR TVPG-GQG-Q G-GAQPQA-- ---PAQVPMP TDVHSQQWT- -AEDPRPHEY ----GAYN-- GGLEAELGGM
2138682  --------LE EVFRFRQLVR TVPGS-TP-H A-HAQPAA-- ---PGQVPMP SGGPNHQWK- -ADEPRPEEY ----G-TM-- QGLEAELGGR
  48096  --------LD EVFRFRQHVR TAPGAGGG-H L-PGQPPA-- ---PAQVPIP AGGPAQQWT- -AEDPRPQEY ----G-SM-- QGLEAELGGR
 179022  --------LD EVFRFRQHVR TAPGGGAG-G I-PGQQPV-- ---QGPVPIP AGGQPQQWT- -AEDPRPQEY ----G-SM-- QGLEAAAPLE DEWTVVD--- ---------- ---TEAI
 778216  --------LD EVFRFRQLVR TQPGQNPG-Q F--GAPAP-- ---AAPVPVP AANNPHQWT- -AEDPRPEEY ----GHQM-- QGLEAELGGR
 665550  --------LD EVSRFRQHVR IQP--GQQ-G Q-NQGPA--- ------VPIP QAG--GSWG- -AEDPRPQEA Y---GQSS-- QGLEQDLERG LDAMHG
 190738  --------LE EVQRFRQHVR IQP--GQQ-P A-APGPA--- ------VPIP QGG--GSWR- -AEDPRPQEA Y---GQGV-- TGLEQDLQGG LDAMHG
1107049  --------LD EVLRFRQLVR TAPASGNQ-G A-PQQIP--- ------VPMP SAG--GQWT- -AEDPRPQEY M---GQP--- NGLEQELQAG MDIRR
 504269  --------YN EVMNFRAVVR QQSQAQAA-A AAQQQQIA-- ---QTNVPIP EHSQ-GGWK- -GEEPKPQEA IAAGG-HM-- NDLESSLQRG MDVIH
 432134  --------YN EVMNFRAVVR QQSQAQAA-A A-QQQQIA-- ---QTNVPIP EHSQ-GGWK- -GEEPKPQEA IAAGG-HI-- NDLESSLQRG MDVIH
  59068  --------LD EVMRFRALVR QESQAQAA-A VAGQQQQM-- ---QQNVPIP ENTN-GAWK- -QEEPRPQEA LAAGGQHA-- NDLEASLHQG MDVH
 336185  --------LD EVVRFRATVR QQSQGHAA-G --QQQAAQ-- ---QTNVPIP D-QQ-GSWK- -TEDPRPQEA TAGGSYNP-- NDLESSLQRG MDMHHR
 343155  --------LD EVVRFRATVR QQSQAHAA-Q --QQQIAQ-- ---QTNVPIP DNQQ-GSWK- -TEDPRPQEA TAGGGYNP-- NDLESSLQRG MDVHH
 326648  --------LD EVNRFRATVR QQSQAHAA-Q --QQQNAQ-- ---QTNIPIP DNQQ-GPWK- -TEDPRPQEA TAGGGYNP-- NDLESSLQRG MDVHHR
 425863  --------LD EVIRFRATVR QQSQAHAT-Q --QQQMAQ-- ---QTNVPIP ENQQ-GGWK- -TEEPKPQEA AAGGGHM--- NDLEASLQRG MDVHH
 436998  --------LD EVINFRAHVR QQQQAAYQ-N --QQSQQV-- ---PQNVPIP EHNQ-GPWK- -QEDPRPQEA PLPGSVQ--- NDLEASLQRG SDFLH
 257317  --------LD EVMRFRQLVR VQPHAQGN-G ---NIAQA-- ---QTSVPIP DHT--GQWG- -HEDPRPQEA SAHGPAAL-- GANWGIMWPE SETSAKV--- ---------- ---RPLSVLY LPNTPAPARV LRLGSS
 998070  --------LQ EVNRFRQLVR VQPGAQVQ-H N-PQMSQG-- ---QSNVPIP EQYD--PRA- -YKDPRPEEM QHAMGMGN-- AGLEQELAGG MDAHMR
  49449  --------LA EVHRFRQMVR IQPTAS---- ---GMGPA-- ---QPSVPIP EHYD--PRA- -YENPRPQEA --GGGVSE-- MGLEQELQQG MDARLR
 498377  --------LQ EVQQFRHDVR KSPHAQV--- G-QAQQGQ-- ---QTNVPIP NNYD--PRS- -YEDPRPQE- --ASQNVN-- MGLEQELQGG LDARMH
 184715  --------YN EVMNFRAVVR QQSQAQAA-A A-QQQQIA-- ---QTNVPIP EHSQ-GGWK- -GEEPKPQEA IAAGGHI--- NDLESSLQRG MDVIH
  51223  --------LD EVTRFRTAVR THP--SPA-N L---NLAQ-- ---DVKMGIP EGHW-P---- -SEAPRPHET TGAVD----- DVLERELAGG LDAMQH
  66669  --------LD EVTRFRTAVR THP--SPA-N L---NLAQ-- ---DVKMGIP EGHW-P---- -SEAPRPHET TGAVD----- DVLERELAGG LDAMQH
 649898  --------LQ EVIKFRHEVR RQP--LPS-N LVDPNIAR-- ---KDSIGIP EHQW-AQQQ- RNEAPRPHEA HGDHG----- HELEDVLAGG LDAMHQ
 253817  --------LQ EVIKFRHEVR RQP--LPS-N LVDPNIAR-- ---KDSIGIP EHQW-AQQQ- RNEAPRPHEA HGDHG----- HELEDVLAGG LDAMHQ
 618068  --------LD EVFRFRQLVR TQPGQNPG-Q F--GAPAP-- ---AAPVPVP AANNPHQWT- -AEDPRPEEY GHQM------ QGLEAELGGR
 108847  --------IE EVKSFREFVR KPIQEQKQ-I QLQIQEKQ-- ---QQQQQRE ADQEQLRKK- LERERQQQQQ QQQQYIQHRE QQIQDQHQHQ QQQFQTQ--- ---------- --QDHQRFET SSKSIPGGEF SDQFGGIMTS NASVLNNTGL
  33629  --------ID EVKKFREFVR KPIHEQQQ-I QLQIQLQQ-- ---RQIEEQE AAAEAHR--- --KEQLLEQQ RQQQQQQQLQ DQLNVMDIVE TPAVHNQTYD AQMLHSAGAS GSSNSHLAHP NAPVNTNPEF SNNADVQYYQ SIPKPQELDE
  54715  --------IE EVKNFREFVR KPIEEQQQ-I QLQLQIQQ-- ---KQQQQQE EQERLQREH- EVKQAILQQQ AREAHLQEQR QQFEAMDISQ TPAPYDYL-- ---------- --NSAHSQNP NTTAVVNTDP SQLTENQYYQ -IPKPQELDE
  88920  --------LE EVVDFRKVVR MPPEQQAA-L AAKSREEK-- ---EKERELA RAREMERDE- TPKPVEDWMR EQQQYLTQQE QQQELSPPPA YAHPHRQY-- ---------- --QHQNTQPK RRDSTIDPSI AKRGYTREEP RPREVVDEAY
  51479  --------LN EVSEFRTAVR LPVAEQQQ-Q ILLQQQQQ-- ---EQEQQQI MLQQ------ --QQLQQQFD LQQQQIQQQQ IQQQLQQFQQ MQNQQLQ--- ---------- -----QQHLH NQHLQQNLHQ QHQQPEIISP PTDSLGIPSG
  86943  --------VE EVKSFREFVR KPIEEQH--- -LIQYNQN-- ---IVDQSQL QRPD------ --DDLNDQQQ QQQPQQPQSF DQFEQLASPA TYFNQQQ--- ---------- -----QQQQQ QQQLQGNF-- --VGNPNIIP NANDFIIDQ-
 100835  --------LD EVESFREFVR RPIEDQN--- -LELSNPQ-- ---AMEIEQP QQQI------ --PQQISQQD QIQDSFQFEL PQDQQQEAYL QQQQQQQ--- ---------- -----QQQYT QQEQQQIFNQ PGLFSPSFES PVGQIGIPQN
 319719  --------IQ EVQDFRLFVR QPLLEEQR-Q LQLQQQQQ-- ---QQQQQQQ QQQQ------ --QQQQQQQQ QQQQPSDVDN GNAAASEENY PKQMATS--- ---------- -----NSVAP QQESFGIHSQ NLPRHDADFP PRPQESMMEM
 822494  --------IQ EVQDFRLFVR QPLLEEQR-Q LQLQQQQQ-- ---QQQQQQQ QQQQ------ --QQQQQQQQ QQQQPSDVDN GNAAASEENY PKQMATS--- ---------- -----NSVAP QQESFGIHSQ NLPRHDADFP PRPQESMMEM
 161919  --------AQ EVMTFKAQKQ GLPPLQMG-- AANLKRKE-- ---SLSANDR EALERSRDV- AKDLDNNSHR HSSSIAASAA APVAQNRQDN MDVDA-L--- ---------- --ERELGGGI
 158631  --------AQ EVMSFKANKE SSDPRNAGNS GTNLKRRE-- ---SMSAQDR ETLAAAQAR- ATLEPSPAAD SGLAVAETAV NPPTATH--H MEVDD-L--- ---------- --ERELSGAI
 115223  --------AQ EVMTYKASKQ QSLQPTA-NT GANLRRRE-- ---SMSAQDR ETLAAAQAR- ATQEADNTDS GIAGVAETAV HH-RPIR--D MEVDE-L--- ---------- --ERELSGMV
 344665  --------AQ EVMSFKASKQ AALGLNR--- --QLGRKS-- ---SLSAPDR DSL--SQQR- QQNNNRNSMY EYGAASQHAA IPSA-----S MEVDEQL--- ---------- --EQELGGLV KENAHHLQ
 178050  --------IR EVMSFKDHKN GRSTPTLGPV AANLRRKD-- ---SMSAHDR ETLEKSKEV- AAQAKAAGVV EDTPDNPETT STSASKDAST STATANA--- ---------- --DTNANANA NAKAIAKANA AIAAASTADY SDTLPSSVPN
 234927  --------AQ EVMSFKASKQ AMLQPQLSP- GANLRRKD-- ---------- ------RQV- LGFSYKKNFV CQLYSNAHNC FSLSAQDREA LEKTKQA--- ---------- --AAAQGKST DHQYHHSQGV AATAGGNIQP PDNMPM----
 328916  --------AQ EVLNFRATVR RHQVPQLS-V ATGAKSTN-- ---LSKIPDR DTILNSPTV- VRGDPDALVA GHYQPTGLD- ---------S AVMSEDL--- ---------- --ERELSGGI
 273327  --------AQ EVLNFRATVR RHQVPQLS-V ATGAKSTN-- ---LSKIPDR DTILNSPTV- VRGDPDALVA GHYQPTGLD- ---------S AVMSEDL--- ---------- --ERELSGGI
 338804  --------AQ EVLNFRATVR RHQVPQLS-V ATGAKSTN-- ---LSKIPDR DTILNSPTV- VRGDPDALVA GHYQPTGLD- ---------S AVMSEDL--- ---------- --ERELSGGI
 527701  --------VE EVRSFRTEVR NQA------- -----KAS-- ----TQIRRQ --AFLFPVA- KKSSTLPFRN TL---PLMEL PL-VIPQATL LKGLLAR
  81317  --------VE EVNCFRAEVR AQA------- -----RAA-- ----GQIRRQ ------DS-- ---LPIPSRD EI---INSPV GE-HAAPNGH TSSYT--T-- ---------- ----SSRHPP ----SPVMDD PSAELERELA GTHLGRR
 831329  --------IE EVNSFRAEVR AQA------- -----RAA-- ----GQIRRQ ------DS-- ---LPIPSRE EI---ISSPV QE-YGPVPGA TSGFT--A-- ---------- ----PSQRPS ----SPVMDH PSDALEKELA GTHLGR
 676624  --------IE EVQSFRAEVR AQA------- -----RAT-- ----GQIKRQ ------DS-- ---LPIPSRD EI---ISSPV QE-YGPIHGA TSGYT--A-- ---------- ----PSQRPP ----SPVMDD PSDALEKELA GTHIGRR
 826941  --------VE EVTSFRAEVR AHA------- -----RAA-- ----GQIRRQ ------DS-- ---LPIPSRD EI---LNSPV QE-HGHVHGG TSNYT--A-- ---------- ----PNRRAS ----SPIMDD PSEELERELA GTHLAGKK
  77377  --------VE EVNSFRAEVR AQA------- -----RAA-- ----GQIRRQ ------DS-- ---LPIPTRD EI---LNSPM QE-H----GS TSGFT--V-- ---------- ----PHDRPH ----SPVMDD PSEALERELA GTHLGGMRR
  59915  --------VE EVNSFRAEVR AQA------- -----RAA-- ----GQVRKQ ------DS-- ---LPIPSRD EI---ISSPV QE-HAPRNGA TSGYT--G-- ---------- ----ARATAP ----EPIMDD PSAELEKELS RQNLGK
 160641  --------VE EVQSFRAEVR QQA------- -----RTG-- ---SGQIQRQ ------NS-- ---LPVPTRE DI---ENSPA AD--G----- --GYP--M-- ---------- ----QDQRAP ----SPVAED PSAELEKELA STHLGARRG
 108839  --------VE EVQSFRAEVR QQA------- -----RTG-- ----GAIRRQ ------DS-- ---LPIPSRE DI---QASPV TN--APLNGG TSGYT--L-- ---------- ----PEERTA ----SPVHED PSAELEKELA STHLGSRRS
```

```
  1536187  --------VE EVHAFRAEVR AQA------- -----RAA-- ----GQIRRQ ------ES-- ---LPMPSRD EI---MNSPV TD-QAPPNGA TSHYTGAA-- ---------- ----GEARPG ----SPILDD PSAELERELA NAHIARR
   281708  --------VE EVHAFRAEVR AQA------- -----RAA-- ----GQIRRQ ------ES-- ---LPMPTRD EI---LNSPV TD-QAPPNGA TSHYTGAA-- ---------- ----GEARPG ----SPIIDD PSAELERELA STHIARR
   173791  --------VE EVHAFRAEVR AQA------- -----RAA-- ----GQIRRQ ------ES-- ---LPMPSRD EI---LNSPV GD-NAAQNGA TSSYTGGN-- ---------- ----GEVRPG ----SPILDD PSAELERELA NAHIARR
    82703  --------VD EVHSFRAEVR AQA------- -----RAS-- ----GQIRRQ ------DST- TRSLPIPSRE EI---LSSPV LE-NAPPNGV TSSYTEPQ-- ---------- ----GPRGP- ----SPVMDD PSAELERELA GTHLGRA
   139538  --------IE EVNSFRAEVR AGV------- -----RAQ-- ----GQIRRQ ------DS-- ---LPIPTRD EL---ENS-- ---------- ASGY---A-- ---------- ----ELQRSP ----VPVMED PSAELERELA GTHLGGR
   479859  --------VE EVNKFRSLVR SQA------- -----RSG-- ---SGA-RRQ ------DS-- ---LPIPSRD DI---INSPV ND-IAPRDGA TSSYTH-V-- ---------- ----SDTVAN ----SPIIDD PSEELEKELE K-HL
   621303  --------VE EVNSFRAMT- ---------- ---------- ---------- ------GN-- ---LPMPSRD DI---ANSPV HE-YAPQDGA TSGYTAHP-- ---------- ----ADARAN GRHASPIMED PSADLERELA SVTGV
    38569  --------VE EVNSFRAMVR SQA------- -----RAS-- ----GQIRRQ ------DS-- ---LPMPSRD DI---ANSPV HE-YAPQDGA TSGYTAHP-- ---------- ----ADARAN GRHASPIMED PSADLERELA SVTGV
   242842  --------VE EVNAFRALVR NKT------- -----RSA-- -----QDKRA ------DGT- ---LPIPSRD DI---ISSPL TE-KAPANGA TSSFTAPG-- ---------- ----GAGPG- ----SPIMED PSEQLEKELA GTHLAGVPT
  2481011  --------IE EVKQFRAEVR AQA------- -----RAA-- ----GQIQRS ------GSD- S-NLGIPSRD EI---MNSPV AE-NYNGQEV PRSQ------ ---------- ---------- ----SPVMED PSAALERELA GK
   169913  --------VD EVNLFRQEVR AQA------- -----RAA-- ----SQGRRQ ------DT-- ---LPIPSHD DI---AASPI QE-DVPANGA TSGYYSGTNT YSYNPAGGQA GQQGAAGAGG QRPSSPVMDD PSAELERELA GTHIGRQ
    71742  --------VD EVASFRDVVR AQA------- -----RGA-- ----GQVRRQ ------DKF- VFGSDVTLIL FF---LHYIT FHVAFPFHPE TKFSTRLYRN MAPSMELHQV TRL-WTGRLL ALSWMSLANS SNVNL-RELV SLDRTRILFI
    66635  --------AE EVREFRAFVR EPI------- -----QEP-- ---VRPAKRQ E--------- --SLPIPSRE EI---INSPP SS-ALDGEGT SMAAG----- ---------- -----GRMIA EFAESPKDEH PWDGLQQDLA RTSL
    53870  --------AE EVREFRAFVR EPI------- -----QEP-- ---VRPAKRQ E--------- --SLPIPSRE EI---INSPP SS-ALDGEGT SMAAG----- ---------- -----GRMIA EFAESPKDEH PWDGLQQDLA RTSL
    27246  --------IE EVNSFRAEVR AQA------- -----RAA-- ---GQIRRQD ---------- --SLPIPSRE EI---ISSPV QE-YGPVPGA TSGFT----- ---------- -----APSQ- -RPSSPVMDH PSDALEKELA GTHLGR
    88978  --------VD EVASFRDVVR AQA------- -----RGA-- ---GQVRRQD ---------- --NLPIPSRD EI---LNSPI QE-HGPVHGA TSGYT----- ---------- -----ALDR- -QTSSPVMDE PSEQLERELA GTRFPGQN
  1091096  --------VQ EVNSFRAEVR AQA------- -----RAA-- ---GQIRRQD RDFDATPFP- PASLPIPTRD EI---INSPV QE-YGPVHGA TSGYT----- ---------- -----APSH- -RPPTPIMDD PSEELERELA GTHLGKR
    66856  --------VE EVHAFRAEVR AQA------- -----RAA-- ---GQIRRQE ---------- --SLPMPSRD EI---LNSPV GD-NAAQNGA TSSYT----- ---------- -----GGNGE VRPGSPILDD PSAELERELA NAHIARRCIS
    20720  --------VD EVHSFRAEVR AQA------- -----RAS-- ---GQIRRQD ---------- --SLPIPSRE EI---LSSPV LE-NAPPNGV TSSYT----- ---------- -----EPQGP -RGPSPVMDD PSAELERELA GTHLGRA
   124539  --------VE EVNSFRAEVR AQA------- -----RAA-- ---GQVRKQD ---------- --SLPIPSRD EI---ISSPV QE-HAPRNGA TSGYT----- ---------- -----GARA- -TAPEPIMDD PSAELEKELS RQNLGK
   361187  --------LE EVYRFRQFVR TVP------- -----GSA-- ---HAASQA- ---------- ------AQQA AA---AAAAA SS-QVPIPSG HGQWT----- ---------- -----ADE-- --PRPQEYLG QGNGLEADLA GGLDGRR
   327531  --------VE EVAQFRQEVR GQS------- -----RGE-- ---TGARRQD ---------- --TLPIPTRD EI---INSPV HE-DAPANGA TSAYT----- ---------- -----RPAEV -RPGSPMMED PAEQLDHELA ARG
     7086  --------VE EVAQFRQEVR GQS------- -----RGE-- ---TGARRQD ---------- --TLPIPTRD EI---INSPV HE-DAPANGA TSAYT----- ---------- -----RPAEV -RPGSPMMED PAEQLDHELA ARG
    28915  --------AQ EVMSFKASKE QAL------- -----GSQ-- ---NGGLRR- ---------- --AASLSAAD RN---ATTHA AR-DTPRASQ QIQEE----- ---------- -----PAMAQ QAPGGGHLES -AMEVDEDLE RELSGQAMQA
   669145  --------VD EVMLFRSEVR GQA------- -----RSN-- ---QGTRRGA E--------- --QLPVPSRD EI---ISSPI QQ-DAPANGA TGYYT----- ---------- -------QG NRVPSPVMDD PSADLERELE QTHLNRS
    46685  --------VE EVTSFRRMVR PNA------- -----TSR-- ---QNSGQGE ------QIR- REALPIPSRE VV---MGSPT TQ-DAPQDGG TSSAT----- ---------- -----AALNG AVNTSPVDDN PAEDLDAELV ARDRAAN
    53954  --------VQ EVEEFRRMVR PQM------- -----RPA-- ---PPPPRRQ ------DSL- PAPPPIPARD AL---GAHA- ------HEGY GAAYG----- ---------- -----RVPQH AGEEGPVEEV PGMELDRELE GQYHGKAR
  1446322  --------VE QVNSFRALVR SQA------- -----RAA-- ---GQARRQE ---------- --SLPIPTRD DI---ISSPV TE-HAPVNGA TSGYT----- ---------- -----ASGDL ARASSPIIDD PSEELERELA GTHLGQHV
   351754  --------VE EVNSFRTLVR SQA------- -----RAA-- ---GQVRRQD ---------- --SLPIPSRD EI---LNSPI QE-HAPKDAF DH-------- ---------- -----PSG-- ARPSSPIMDD PSAELDRELG TKKY
    65873  --------VE EVKSFRKKVR TPV------- -----NVQ-- ---QQQQAEQ QRLMEEQAA- AHIQAKEAKE SLPVPSREEL SGNETPKDEL TSSYT----- ---------- ----IPPQAN ANDGSYVLDD PSEELERELA STKI
    52469  --------VE EVKSFRKKVR TPV------- -----NVQ-- ---QQQQAEQ QRLMEEQAA- AHIQAKEAKE SLPVPSREEL SGNETPKDEL TSSYT----- ---------- ----IPPQAN ANDGSYVLDD PSEELERELA STKI
    30002  --------VE EVKSFRKKVR TPV------- -----NVQ-- ---QQQQAEQ QRLMEEQAA- AHIQAKEAKE SLPVPSREEL SGNETPKDEL TSSYT----- ---------- ----IPPQAN ANDGSYVLDD PSEELERELA STKI
   154156  --------ID EHYDTYIQAG TVQLA----- ---SQ
   166841  --------ID EVKMFKARKQ SLRLD----- ---MRGLR-- ---RQNSITT PTSKRARYN- SQVNEMGYAD QLITSPVGVD PNLERDLSGI I
   238118  --------IE EVKHFKARKH SLKID----- ---MKGLR-- ---RQHSITT PT----KAN- GSDCTFGYAN ELIASPVEVD PHLERDLSGL S
   143069  --------IE EVKHFKARKH SLKID----- ---MKGLR-- ---RQHSITT PT----KAN- GSDCTFGYAN ELIASPVEVD PHLERDLSGL S
   456995  --------IE EVNSFRAEVR AGVRAQG--- ---QIRRQ-- ---DRYVCL
   175880  --------VE EVNAFRALVR NKTRS-A--- ---QDKRA-- ---DG
    28309  --------IK EVFDFRKVVR KHPISG---- ---DSPSS-- ---SLSLEDA IPQEVVQVH- PSRKVLPSYS PEFSY----- ------PYQN LMGIS----- ---------- ----SNSFQG VNEKENTFKQ DTKHENSELL NGPTEPNITK
   767958  --------IK EVFDFRKVVR KHPISG---- ---DSPSS-- ---SLSLEDA IPQEVVQVH- PSRKVLPSYS PEFSYVSQLP SLTTTQPYQN LMGIS----- ---------- ----SNSFQG VNEKENTFKQ DTKHENSELL NGPTEPNITK
   126152  --------IK EVFDFRKVVR KHPISG---- ---DSPSS-- ---SLSLEDA IPQEVVQVH- PSRKVLPSYS PEFSYVSQLP SLTTTQPYQN LMGIS----- ---------- ----SNSFQG VNEKENTFKQ DTKHENSELL NGPTEPNITK
   613191  --------LE EVYRFRQVVR TVPGSGGGQH AGQQGGAS-- -----QVPMP SGGATGQWR- -TEDPRPEEY M-----GQQP GGLEADLAMG ADGRR
   198058  --------LE EVYRFRQFVR TVPGSAHAAS QAAQQAAAAA AAASSQVPIP SGHG--QWT- -ADEPRPQEY L-----GQGN G-LEADLAGG LDGRR
   123006  --------LD EVFRFRQHVR TAPGAGGGHL PGQPPAPA-- -----QVPIP AGGPAQQWT- -AEDPRPQEY ------GSMQ G-LEAELGGR
   178993  --------LD EVFRFRQHVR TAPGGGAGGI PGQQPVQG-- -----PVPIP AGGQPQQWT- -AEDPRPQEY ------GSMQ G-LEAELGGR
   122967  --------LD EVFRFRQHVR TAPGAGGGHL PGQPPAPA-- -----QVPIP AGGPAQQWT- -AEDPRPQEY ------GSMQ G-LEAELGGR
   120501  --------LD EVFRFRQLVR TVPG-GQGQG GAQPQAPA-- -----QVPMP TDVHSQQWT- -AEDPRPHEY ------GAYN GGLEAELGGM
    99018  --------LA EVNRFRHEVR VQPGANGQGQ AQQQAQA--- -----PVPIP NNY--DRAG- -YEDPRPQET MMQG--GWQA NDLERDLQ-G LDARGMR
   213678  --------LA EVNNFRRAVR MQPGQQAQGQ GQQPPQ---- -----PVPMP NNY--DRSA- -YQDPRPHEV GAQG--NWEG NDLERDLQ-G LDGRSMRQ
   460523  --------LA EVNRFRHEVR VQPGAGGAGQ ASQQPA---- ------VPIP NNY--DRAG- -YEDPRPQET MMQG--GVWQ VYLCPDSC
   625621  --------LQ EVIKFRHEVR RQPLPSNLVD PNIARKD--- -----SIGIP EHQWAQQQR- -NEAPRPHEA HGDH--GHEL EDVLAG---G LDAMHQ
   265871  ---------- ---RFRATVR QQSQAHAAQQ QQIAQQT--- -----NVPIP DNQ-QGSWK- -TEDPRPQEA TAGG--GYNP NDLESSLQRG MDVHH
    53464  --------LD EVMRFRQLVR VQPHAQGNGN IAQA-QT--- -----SVPIP DHT--GQWG- -HEDPRPQEA SAHGPAGGPA GGLEQELQGG LDAMH
   105414  --------LH EVRQFRSLVR APLQQQQQRY AEQQQQQLQQ QQQQQQQGSG QVPIPDQFSR QSEDPRPEEM N-----GVQG GSLEQELGYG LDAGRQMR
   291876  --------LH EVRQFRSLVR APLQQQQQRY AEQQQQQLQQ QQQQQQQGSG QVPIPDQFSR QSEDPRPEEM N-----GVQG GSLEQELGYG LDAGRQMR
    50744  --------IE EVKSFRE--- ---------- ---------F VRKPIQEQKQ IQLQIQEKQQ QQQQREADQE QL---RKNSK SIPGGEFSDQ FGGIM----- ---------- -----TSNAS VLNNTGLLTD PSQ-LRNEDN LQHYDAMPK-
   863837  --------AQ EVMSFKA--- ---------- ---------- -----SKEQA LGSQNGGLRR AASLSAADRN AT---THAAR DTPRASQQIQ EEPAM----- ---------- -----AQQAP GGGHLESAME VDEDLERELS GQAMQA
    81355  --------VE EVNCFRA--- ---------- ---------- ------EVRA QARAAGQIRR QDSLPIPSRD EI---I-NSP VGEHAAPNGH TSSYT----- ---------- -------TSS RHPPSPVMDD PSAELERELA GTHLGRR
   152896  --------VE EVHAFRA--- ---------- ---------- ------EVRA QARAAGQIRR QESLPMPTRD EI---L-SSP VTDQAPANGA TSHYT----- ---------- -----GATGE ARPGSPIMDD PSAELERELA STHIARR
  1196002  --------VE QVNSFRA--- ---------- ---------- ------LVRS QARAAGQARR QESLPIPTRD DI---I-SSP VTEHAPVNGA TSGYT----- ---------- -----ASGDL ARASSPIIDD PSEELERELA GTHLGQHV
  1195972  --------VE QVNSFRA--- ---------- ---------- ------LVRS QARAAGQARR QESLPIPTRD DI---I-SSP VTEHAPVNGA TSGYT----- ---------- -----ASGDL ARASSPIIDD PSEELERELA GTHLGQHV
    49917  --------RQ EVELFRKEVR VSSQPQ---- ---------- ---PLSTARI ASGTLTNRIS YSHERQNYRP NTASSFNQPS NSNKVQYQAM PPRRVQ---- ---------- ----SQSQSR SQATLPMRHH SFQQRALHRA ET
   116823
   271167
   151286
    97820
   167647
   430394
   266211
   266223
   266189
   151328
   800193
    22041
    22014
    22020
   448385
   122008  AAMFTV---- G
   578295  SADNTS---- GTPS-KKKEK RFLIF
   139503
  2468658  EFE
  2215386  EFE
    88816  GSASGTPSGN GLPTTKRKER RFILF
    88949  GSASGTPSGN GLPTTKRKER RFILF
    88917  GSASGTPSGN GLPTTKRKER RFILF
   206606
   123647
   143931
   165009
   580315
   492840
   594182
   168238
   107302
    64718
    64830
  1154823
   433002
   822334
   511572
   688421
    17778
  1336823
   605623
   511468
    60300
  2435698
  1222436
  1172380
  1151825
   410058
    57461
   311323  LGHEDEGR-- KVPWPRMNWL SDVRGAPGVR IVVGRVVV-- RSRSMGSSVS EGVVEERRLV AEGAGRGGRM EIDIVGEWLE LGCEGDVVEI GSAQPEG
   143102  PTDHSRPE-- QHP-SNYAFG PNLSRHDSVG SHTNTIYLED GDRNMDQMM- RVTTSMAAQD VQTEANELLL HTDGMPSLGL VLSRIPTVDT SLAAFASI-- ---------- --GQPDNMPP SKSTLTSPRV WTFLVTTLLF GIAYAMIAQF
  2750129  SRSRNEEE-- DAPRSGLGSR SGLGSGSGIG FGGGLGFSGS RPRDDDDSAP RGGLGSFARM FQMPSTAPTT ETGKGMAMFA ALSSSKVLSG EAAPAEEV-- ---------- --PVTETEKE RRKREKKEKK RKAEETQENE EKAAKKEKAA
  2702932  SRSRNEEE-- DAPRSGLGSR SGLGSGSGIG FGGGLGFSGS RPRDDDDSAP RGGLGSFARM FQMPSTAPTT ETGKGMAMFA ALSSSKVLSG EAAPAEEV-- ---------- --PVTETEKE RRKREKKEKK RKAEETQENE EKAAKKEKAA
   108010  ESSATPGSTN DLPHSTMPRT VDLLFRARTK AGDY-VWVEC RGRLHVEPGK GRKAIILSGR AMGMASMIWS SIVQGGGLNT SIRNTRAISR PEDEGVEV-- ---------- --EVVETERE FWGSLSKEGV LLVVGRGAKD VLGWAESEMQ
  1754091  RASSDEGWGV NLAECIQIWR EGCIIRSEYI ADLLQAAYEK EAGVMNTLTL QPVADEIKRT IPHLLKVVKY GLEWNAHIPT LSASLEYFKY CGGKHLPS-- ---------- --QFMEAELD YFGAHNYDLK SEGDGEVKKG EHHYEWKPA
   564944  DGGARDGDFV TFENHDPKGS QHILEPAELP SRDALLVHAA YTSVRHLSGF MDESKDF--- ---LDDPNFS FLGRTGERRT YVKECEAYSY TETEEDSL-- ---------- --EYLEKQ-S LISSASSQLF VEGDAEITGE SMSMTKMGCV
  AtMPK18
  AtMPK19
  AtMPK20
OsMPK20-1
OsMPK20-4
OsMPK20-5
OsMPK20-3
  AtMPK16
OsMPK16-1
  AtMPK17
   AtMPK8
  AtMPK15
OsMPK17-1
   AtMPK9
```
